# iTRAQ Quantitative Proteomic Comparison of Metastatic and Non-Metastatic Uveal Melanoma Tumors

**DOI:** 10.1371/journal.pone.0135543

**Published:** 2015-08-25

**Authors:** John W. Crabb, Bo Hu, John S. Crabb, Pierre Triozzi, Yogen Saunthararajah, Raymond Tubbs, Arun D. Singh

**Affiliations:** 1 Cole Eye Institute, Cleveland Clinic, Cleveland, Ohio, United States of America; 2 Lerner Research Institute, Cleveland Clinic, Cleveland, Ohio, United States of America; 3 Department of Ophthalmology, Cleveland Clinic Lerner College of Medicine of Case Western Reserve University, Cleveland Clinic, Cleveland, Ohio, United States of America; 4 Department of Molecular Medicine, Cleveland Clinic Lerner College of Medicine of Case Western Reserve University, Cleveland Clinic, Cleveland, Ohio, United States of America; 5 Department of Quantitative Health Sciences, Cleveland Clinic, Cleveland, Ohio, United States of America; 6 Taussig Cancer Institute, Cleveland Clinic, Cleveland, Ohio, United States of America; 7 Department of Molecular Pathology, Pathology & Laboratory Medicine Institute, Cleveland Clinic, Cleveland, Ohio, United States of America; University of Florida, UNITED STATES

## Abstract

**Background:**

Uveal melanoma is the most common malignancy of the adult eye. The overall mortality rate is high because this aggressive cancer often metastasizes before ophthalmic diagnosis. Quantitative proteomic analysis of primary metastasizing and non-metastasizing tumors was pursued for insights into mechanisms and biomarkers of uveal melanoma metastasis.

**Methods:**

Eight metastatic and 7 non-metastatic human primary uveal melanoma tumors were analyzed by LC MS/MS iTRAQ technology with Bruch’s membrane/choroid complex from normal postmortem eyes as control tissue. Tryptic peptides from tumor and control proteins were labeled with iTRAQ tags, fractionated by cation exchange chromatography, and analyzed by LC MS/MS. Protein identification utilized the Mascot search engine and the human Uni-Prot/Swiss-Protein database with false discovery ≤ 1%; protein quantitation utilized the Mascot weighted average method. Proteins designated differentially expressed exhibited quantitative differences (p ≤ 0.05, t-test) in a training set of five metastatic and five non-metastatic tumors. Logistic regression models developed from the training set were used to classify the metastatic status of five independent tumors.

**Results:**

Of 1644 proteins identified and quantified in 5 metastatic and 5 non-metastatic tumors, 12 proteins were found uniquely in ≥ 3 metastatic tumors, 28 were found significantly elevated and 30 significantly decreased only in metastatic tumors, and 31 were designated differentially expressed between metastatic and non-metastatic tumors. Logistic regression modeling of differentially expressed collagen alpha-3(VI) and heat shock protein beta-1 allowed correct prediction of metastasis status for each of five independent tumor specimens.

**Conclusions:**

The present data provide new clues to molecular differences in metastatic and non-metastatic uveal melanoma tumors. While sample size is limited and validation required, the results support collagen alpha-3(VI) and heat shock protein beta-1 as candidate biomarkers of uveal melanoma metastasis and establish a quantitative proteomic database for uveal melanoma primary tumors.

## Introduction

Uveal melanoma (UM) is the most common primary malignancy of the eye. The overall mortality rate is high (~40%) because this aggressive cancer metastasizes often before ophthalmic diagnosis [1, 2]. The survival rate has remained unchanged for several decades [3]; median survival time after detection of UM metastasis is about 9 months [4]. The most common sites of metastases are the liver (93%), lung (24%), and bone (16%), with the majority of patients exhibiting multiple sites [5]. UM originates in the capillary-rich uveal tract (iris, ciliary body, and choroid) and metastasize almost exclusively by the hematogenous route. Many differences exist between uveal and cutaneous melanoma, although there is a common embryologic origin of melanocytes [6]. The etiology of UM remains poorly understood. Mutations in GNAQ/11 [7, 8] and in the BRCA1-associated protein-1 (BAP1) [2, 9] and disruption of epigenetic regulators [10] have been suggested as possible events in UM metastasis. Most patients are now treated with plaque radiotherapy, rather than enucleation because of equivalent rates of metastasis [1]. However, improved survival rates have yet to be realized because undetectable micrometastases can occur and lie dormant for decades prior to diagnosis and treatment of primary tumors [11].

Several chromosomal abnormalities occur in UM (reviewed in [2]). The most common abnormalities include loss on chromosomes 1p, 3, 6q, 8p and 9p and gain on chromosomes 1q, 6p, and 8q [2]. High UM metastatic risk is most strongly associated with monosomy 3 but also with gain on 8q [2, 12]. Gain on chromosome 6p has been correlated with lower metastatic risk [13]. Genome-wide single-nucleotide polymorphism analysis [14], fluorescent *in situ* hybridization [15], and multiplex ligation-dependent probe amplification [13] are used to detect chromosomal irregularities in UM. These cytogenetic analyses all require tumor biopsies, commonly a fine needle aspirant biopsy. Such biopsies introduce additional diagnostic challenges because of the heterogeneity of UM primary tumors [16, 17]. Gene expression studies of UM tumors have led to the development of a validated gene profiling assessment test for UM metastasis risk [18, 19], however, this test also requires a tumor biopsies. Serum biomarkers with sufficient discriminatory accuracy to detect UM micrometastases would improve prognostic methods and enhance clinical patient care.

As an approach to better understanding mechanisms and biomarkers of UM metastasis, we have pursued global quantitative proteomic analyses of UM tumors. Several UM proteomics studies have been reported [reviewed in [20–22]], however only two have focused on primary UM tumor tissue [23, 24]. This is the first UM study to use LC MS/MS iTRAQ technology, proteomic methodology that offers more quantitative accuracy than 2D gel and label-free approaches [25]. This is also the first UM proteomics study to use Bruch’s membrane/choroid as normal control tissue. Bruch’s membrane is a stratified extracellular matrix separating the retinal pigment epithelium from the blood-bearing choroid portion of the uveal tract [26] where UM tumors originate. We report here global quantitative proteomic analysis of 15 UM primary tumors, including 8 metastatic and 7 non-metastatic tumors. Proteins identified in a training set of 5 metastatic and 5 non-metastatic tumors were used to successfully predict the metastatic status in a test set of 5 independent primary UM tumors. The results provide new clues to UM mechanisms and biomarkers and make available a quantitative proteomic database.

## Materials and Methods

### Ethics Statement

All human specimens used in this study were collected with adherence to the principles expressed in the Declaration of Helsinki. Human UM tumor tissue procedures in this study were approved by the Cleveland Clinic Institutional Review Board, study number 666, case 5608 entitled “Prognostication of Uveal Melanoma by Fine Needle Aspiration (FNA) and Fluorescence in Situ Hybridization (FISH)”. All tumor tissues were obtained with the informed written consent of the patients. Postmortem human control tissues used in this study complied with the policies of the Eye Bank Association of America and the Institutional Review Board of the Cleveland Clinic Foundation.

### Human Tissues

Human eyes from UM patients undergoing enucleation at the Cleveland Clinic were used to isolate ocular tumor specimens for proteomic analyses. Nine normal human postmortem eyes were obtained from the Cleveland Eye Bank, Cleveland OH and the National Disease Research Interchange, Philadelphia for isolation of control tissue. Criteria for normal postmortem eyes included adult donors over 40 years of age with no history of glaucoma, diabetic retinopathy, age-related macular degeneration, uveitis or ocular trauma. Normal eyes were from 5 females, 4 males, one black and 8 white donors, and ranged in age from 66–87 years (average ~77 years). Causes of death for the normal eye donors were primarily cardiovascular disease, but included subdural hematoma, Alzheimer’s disease, and breast cancer. For control tissue, the Bruch’s membrane/choroid complex was isolated from normal eyes by first removing the anterior segment, vitreous humor and retina, then brushing away the retinal pigment epithelium and finally separating the choroid/Bruch membrane complex from the posterior globe [27]. Cytogenetic analyses of UM tumor tissues by fluorescent *in situ* hybridization analyses for chromosomes 3 and 8 abnormalities were performed in the Department of Molecular Pathology, Cleveland Clinic, and by genome-wide single-nucleotide polymorphism analysis for chromosome 3 abnormalities in the Genomics Core Facility at the Cleveland Clinic.

### Sample Preparation

Protein was extracted from UM tumors and from normal control tissues in 100 mM triethylammonium bicarbonate containing 2% SDS. Soluble protein in tissue extracts was precipitated with four volumes of acetone and redissolved in 500 mM triethylammonium bicarbonate containing 0.1% SDS and quantified by the bicinchoninic acid assay [28]. Equal amounts (w/w) of 9 control specimen were pooled to form a single control reference sample. Individual tumor extracts and the pooled control sample were reduced with tris-(2-carboxyethyl) phosphine, cysteines alkylated with methyl methanethiosulfonate, and digested with trypsin [27, 29].

### iTRAQ labeling and SCX Chromatography

iTRAQ labeling with a 4-plex iTRAQ kit and chromatography methods were as previously described [27, 29–31]. In this study, tryptic digests of each of the 15 tumors (~100 μg/specimen) were labeled individually with iTRAQ tags 115, 116, or 117 and mixed individually with an equal amount of tryptic digest from the pooled control sample labeled with iTRAQ tag 114. Each individual tumor/control peptide mixture was fractionated by strong cation exchange (SCX) chromatography using an Ultimate 3000 LC system (LC Packings), a PolySulfoethyl A column (1.0 x 150 mm, 5 μm particle size, 200 Å pore size), a flow rate of 50 μl/min and a gradient of 0–600 mM KCl in 25% acetonitrile, 10 mM KH_2_PO_4_, pH 3. SCX chromatography was monitored by absorbance at 214 nm, and fractions (n = 90) were collected at 1 min intervals. Low absorbance, adjacent SCX fractions were combined and about 60 fractions per tumor specimen analyzed LC MS/MS.

### Protein Identification

SCX fractions were analyzed by LC MS/MS with a QTOF2 mass spectrometer equipped with a Cap LC system (Waters) as described [27, 29–31]. Protein identification utilized MASSLYNX 4.1 software (Waters), the Mascot search engine (Matrix Science, version 2.4.1), and the human Uni-Prot/Swiss-Protein sequence database (October 2014, 20,196 total sequences). Database search parameters were restricted to three missed tryptic cleavage sites, a precursor ion mass tolerance of 25 ppm, a fragment ion mass tolerance of 0.3 Da and a false discovery rate of ≤ 1%. Fixed protein modifications included N-terminal and epsilon-Lys iTRAQ modifications and *S*-methyl-Cys. Variable protein modifications included Met oxidation, Asn and Gln deamidation and iTRAQ Tyr. A minimum Mascot ion score of 20 was used for accepting peptide MS/MS spectra. Two unique peptides per protein were required for all protein identifications.

### Protein Quantitation

iTRAQ tags were quantified by the weighted average method using Mascot 2.4.1. Protein quantitation required a minimum of two unique peptides per identified protein, ion intensities ≥ 10 for all iTRAQ tags and Mascot peptide ion scores ≥ 20. Outlier peptides were eliminated using Dixon’s [32] and Rosner’s [33] methods. Protein ratios were determined in log space and transformed for reporting. For averaging results over multiple samples, protein ratios per sample were normalized to the protein median, then average protein ratios were calculated along with standard error of the mean (SEM) and *p* values (two-sided t-test for the null hypothesis that the protein mean = 0 in log space). Finally for reporting, average protein ratios were normalized to the protein mean. Proteins exhibiting average protein ratios above or below the mean by at least 1 standard deviation and *p* values ≤ 0.05 were considered significantly elevated or decreased. For individual samples, calculation of standard deviations and p-values required ≥ 3 unique peptides per protein; for average values, p-values were based on the presence of the protein in ≥ 3 samples.

### Bioinformatic Analyses

Bioinformatic analysis of pathways and protein molecular and cellular functions was performed with Ingenuity Pathways Analysis (release date 2014-09-22, Qiagen).

### Western Analysis

Western blot analysis of UM tumor and control Bruch’s/choroid control tissue lysates were performed using 12.5% acrylamide Criterion precast gels (1 mm x 7 cm x 13.5 cm, BioRad), SDS-PAGE, polyvinylidene fluoride membrane (Millipore), and chemiluminesence detection (GE Healthcare) [34, 35]. Chemiluminesence was detected with a Bio-RAD GS-710 densitometer. Prior to Western blot analysis, sample amounts applied to the SDS-PAGE (~10 μg) were equalized based on Coomassie blue staining intensities as described elsewhere [35]. Primary antibodies included anti-macrophage migration inhibitory factor (mouse monoclonal antibody (mAb), R&D Systems, Minneapolis, MN), anti-glyceraldehdye-3-phosphate dehydrogenase (mouse mAb, EMD Millipore), anti-glutathione S-transferase (rabbit polyclonal antibody (pAb), EMD Millipore), anti-lactate dehydrogenase (goat pAb, Abcam Inc.), anti-complement C9 (goat pAb, Abcam, Inc.), metalloproteinase inhibitor 3 (mouse mAb, Millipore Corporation), anti-A-kinase anchor protein 12 (mouse mAb, Abcam Inc.), and vitronectin (rabbit pAb, Abcam Inc.). Secondary antibodies were obtained from GE Healthcare and Santa Cruz Biotechnology.

### Experimental Design and Statistics

Each of the 15 tumor/control iTRAQ-labeled peptide mixtures was individually fractionated by SCX chromatography and analyzed by LC MS/MS. For data analysis, a training set of 10 tumors [metastatic samples UM 19, 21, 24, 28, 30 and non-metastatic samples UM 13, 20, 23, 25, 26] was defined to compare tumor type proteomes and to identify differentially expressed proteins. Proteins uniquely elevated or decreased in metastatic tumors and those present only in metastatic or non-metastatic tumors were identified in the results from the 10 sample training set. Proteins designated differentially expressed exhibited significant abundance differences in the training set (p ≤ 0.05, two sided t-test) without adjustment for multiple comparisons. Differentially expressed proteins were incorporated into logistic regression models with the individual proteins as predictors and the logistic models were used to classify metastatic status in a test set of 5 independent UM tumors (metastatic samples UM 09, 11, 12 and non-metastatic samples UM 02, 15). For multivariate predictions, dimension reductions were performed by applying principal component analysis (PCA) to the 17 differentially expressed proteins observed in all 10 samples of the training set. Multivariate classification of the 5 independent UM tumor samples in the test set utilized two principal components based on the loadings from PCA, with the probabilities of metastasis computed from a multivariate logistic model. All statistical analyses were conducted with R-studio (Boston, MA).

## Results

### Overview

**Fig 1:**
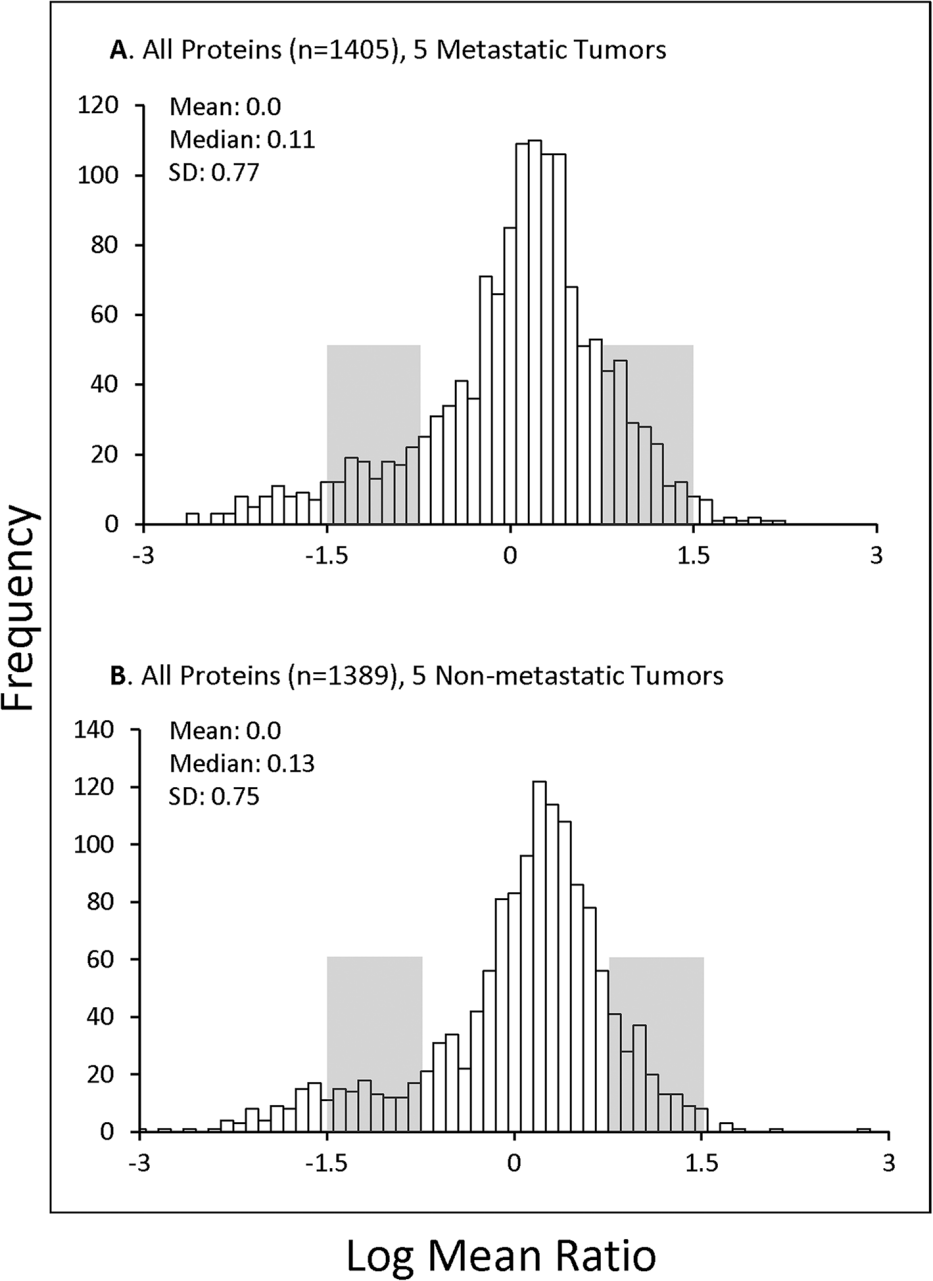
Distribution of Tumor Protein Ratios. The log_2_ mean distribution of protein ratios (Tumor/Control) are shown for proteins quantified in (A) 5 metastatic UM tumors, including 1405 quantified proteins (from S16 Table); and (B) 5 non-metastatic UM tumors, including 1389 quantified proteins (from S17 Table). Median, mean and SD values are indicated; protein ratios between 1–2 SD from the mean are shaded. The distribution of protein ratios is similar in both metastatic and non-metastatic tumors.

Over 1700 proteins were quantified with two or more peptides from 15 UM ocular tumors using LC MS/MS iTRAQ technology. Properties of the tumors analyzed are presented in Table 1, including demographic and clinical characteristics of the donors, metastasis and survival status, chromosome 3 status, tumor location, cell type and histopathology. Quantitative proteomics results from each sample are itemized in S1–S15 Tables, including protein ratios, standard deviation (SD), p values, number of unique peptides quantified, and percent sequence coverage for each protein. The average relative abundance of proteins quantified in 5 metastatic (n = 1405 proteins) and 5 non-metastatic (n = 1389 proteins) tumors are presented in S16 and S17 Tables, respectively. The distributions of average protein ratios are similar between metastatic and non-metastatic tumors and near-to-normal, although both tumor groups exhibit slightly more proteins decreased than increased relative to control tissue (Fig 1). Criteria for determining whether a protein was elevated or decreased included the average protein ratio and p value. Proteins exhibiting average ratios above or below the mean by at least 1 SD and p values ≤ 0.05 were considered significantly elevated or decreased, and are highlighted by color-coding throughout the supplementary tables. Quantitative proteomic results for each tumor specimen are summarized in S18 Table, including the number of proteins quantified per sample, as well as the number of significantly elevated and decreased proteins.

**Table 1:** Uveal Melanoma Tumor Specimens used for Proteomics Analysis.

Sample	Gender	Age (y)	Enucleation Date	Donor Status	Metastasis	Chromosome Status	Tumor Location	Tumor Cell Composition^c^
UM19	M	51	2007	Deceased	Yes	monosomy 3^b^	choroid	spindle > epithelioid
UM21	F	86	2008	Deceased	Yes	monosomy 3^b^	ciliary body, choroid	epitheloid > spindle
UM24	M	56	2005	Deceased	Yes	monosomy 3^a^	ciliary body, choroid	spindle > epithelioid
UM28	M	66	2008	Deceased	Yes	monosomy 3^b^	iris, ciliary body, choroid	spindle > epithelioid
UM30	M	75	2007	Deceased	Yes	monosomy 3^b^	ciliary body, choroid	spindle = epithelioid
UM13	F	79	2007	Deceased	No	monosomy 3^b^	iris,ciliary body, choroid	spindle
UM20	M	65	2007	Alive	No	disomy 3^a^	iris, ciliary body, choroid	spindle
UM23	M	52	2006	Alive	No	disomy 3^b^/8q amp^a^	ciliary body, choroid	spindle = epithelioid
UM25	F	68	2007	Alive	No	disomy 3^b^/8q amp^a^	choroid	spindle > epithelioid
UM26	F	76	2008	Alive	No	disomy 3^b^	choroid	spindle > epithelioid
UM02	F	82	2008	Alive	No	disomy 3^a^	ciliary body, choroid	epithelioid > spindle
UM09	F	78	2008	Deceased	Yes	disomy 3^b^	ciliary body, choroid	spindle > epithelioid
UM11	M	75	2008	Deceased	Yes	monosomy 3^b^	iris, ciliary body, choroid	epithelioid > spindle
UM12	F	73	2007	Deceased	Yes	monosomy 3^a^	iris, ciliary body, choroid	epithelioid > spindle
UM15	M	57	2007	Alive	No	monosomy 3^b^	iris, ciliary body, choroid	spindle = epithelioid

a. Karyotyping based on fluorescent in situ hybridization of chromosomes 3 and 8 (8q amp denotes 8q amplification).

b. Karyotyping based on genome-wide single-nucleotide polymorphism analysis.

c. Tumor cell type is shown; symbols reflect greater (>) or equal (=) relative amounts of the cell type.

### Independent Evidence Supporting the iTRAQ Protein Quantitation

**Fig 2:**
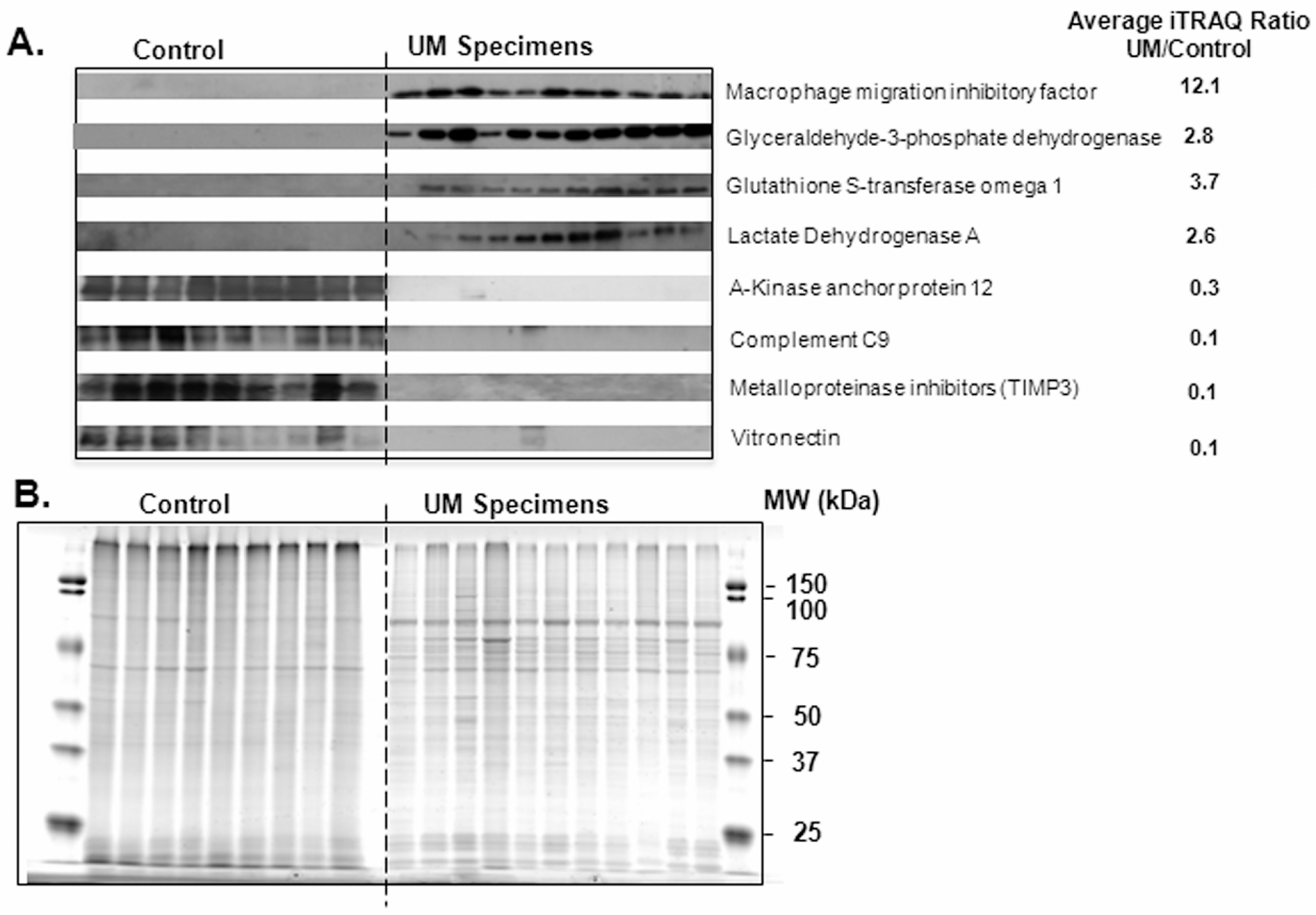
Western and SDS-PAGE Analysis. (A) Western blot analysis of the indicated 8 proteins in 11 UM tumors (samples 02, 09, 13, 15, 19, 20, 21, 23, 25, 26, 30) and 9 control Bruch’s membrane choroid specimens. The intensity of immunoreactivity in the Western blot supports the average iTRAQ ratios for these proteins. (B) Coomassie blue stained SDS-PAGE (~10 μg/lane) is shown with the same samples and amounts used for the Western analyses in panel A. These results support equal protein amounts per lane and show that UM tumor specimens and normal Bruch’s/choroid control samples exhibit different SDS-PAGE profiles.

Western blot analysis was used to independently evaluate the abundance of 8 proteins in 11 ocular tumors and 9 control samples. Immunoreactivity in the Western blots (Fig 2A) corroborate the iTRAQ quantitation and show that the primary tumor tissues, relative to the control, contain increased amounts of macrophage migration inhibitory factor, glyceraldehyde-3-phosphate dehydrogenase, glutathione S-transferase omega 1, and lactate dehydrogenase A and decreased amounts of A-kinase anchor protein 12, C9, metalloproteinase inhibitor 3, and vitronectin. As expected, UM tumor tissues exhibit different SDS-PAGE patterns than normal Bruch’s membrane/choriod control tissues (Fig 2B).

### Proteomic Comparison of Metastatic and Non-Metastatic Tumors

Both qualitative and quantitative proteomic comparisons were performed to identify proteins possibly associated with UM metastasis. First, a training set of 10 tumor samples was established and proteins in metastatic tumors (UM 19, 21, 24, 28, 30) were compared qualitatively with those in non-metastatic tumors (UM 13, 20, 23, 25, 26). Of the 1644 proteins detected in these 10 samples, 70% (1150 proteins) were present in both tumor types, supporting significant proteomic similarities. We identified 255 proteins only in metastatic tumors (S19 Table) and 239 proteins only in non-metastatic tumors (S20 Table), however the majority of these were detected in a single sample. Proteins found uniquely in three or more metastatic tumors are listed in Table 2; none of these 12 proteins were significantly elevated or decreased relative to the control.

**Table 2:** Proteins Detected only in Metastatic UM Tumors.

Uni-Prot	Protein	Sample Frequency	Average Ratio	SEM	P value
P07093	Glia-derived nexin	3	2.30	0.21	0.059
P02763	Alpha-1-acid glycoprotein 1 (orosomucoid 1)	3	1.99	0.48	0.289
P48426	Phosphatidylinositol 5-phosphate 4-kinase type-2 alpha	3	1.70	0.18	0.096
Q06787	Fragile X mental retardation protein 1	3	1.60	0.16	0.104
Q9Y383	Putative RNA-binding protein Luc7-like 2	3	1.41	0.21	0.242
Q15046	Lysine—tRNA ligase	3	1.36	0.24	0.330
P55145	Mesencephalic astrocyte-derived neurotrophic factor	3	1.17	0.11	0.281
Q9UBS4	DnaJ homolog subfamily B member 11	3	0.88	0.10	0.308
Q9UPN3	Microtubule-actin cross-linking factor 1, isoforms 1/2/3/5	4	0.87	0.13	0.379
P04114	Apolipoprotein B-100	3	0.65	0.53	0.502
Q9NVA2	Septin-11	3	0.60	0.18	0.106
P26583	High mobility group protein B2	5	0.55	0.17	0.025

Only proteins quantified in ≥ 3 metastatic tumor samples are shown among 1644 total proteins quantifed in 5 metastatic and 5 non-metastatic tumors. Average Ratio reflects UM/control. All proteins (n = 255) detected only in metastatic tumors are listed in S19 Table.

As a second approach to identifying metastasis associated proteins, we compared significantly elevated or decreased proteins in metastatic and non-metastatic tumors within the training set. The average results (S16 and S17 Tables) again revealed similarity, with the majority of the significantly altered proteins (70–78%) being altered in both metastatic and non-metastatic tumors. Nevertheless, we found 28 proteins significantly elevated (Table 3) and 30 proteins significantly decreased (Table 4) only in metastatic tumors. Fatty acid binding protein (FABP3) was the most significantly elevated (UM/control ratio = 4.9) and transducin gamma was the most significantly decreased (UM/control ratio = 0.07). Elevated serum proteins in Table 3 (eg, albumin and serotransferrin) may be associated with blood contamination of the tumor specimens.

**Table 3:** Proteins Significantly Elevated only in Metastatic UM Tumors.

Uni-Prot	Protein	Sample Frequency	Average Ratio	SEM	*p* value
P05413	Fatty acid-binding protein, heart	3	4.91	0.19	0.014
P23381	Tryptophan—tRNA ligase, cytoplasmic	5	4.42	0.15	0.001
P02768	Serum albumin	5	4.04	0.24	0.004
P52566	Rho GDP-dissociation inhibitor 2	4	3.74	0.29	0.020
Q8IV08	Phospholipase D3	4	3.64	0.12	0.002
P31146	Coronin-1A	4	3.31	0.25	0.018
Q9UBR2	Cathepsin Z	3	3.28	0.07	0.003
P61088	Ubiquitin-conjugating enzyme E2 N	3	3.02	0.07	0.004
P07686	Beta-hexosaminidase subunit beta	4	3.02	0.15	0.005
Q96C86	m7GpppX diphosphatase	4	2.93	0.13	0.004
P02787	Serotransferrin	5	2.91	0.21	0.007
P40121	Macrophage-capping protein	3	2.89	0.11	0.011
Q01105	Protein SET	3	2.79	0.14	0.019
P13693	Translationally-controlled tumor protein	3	2.70	0.13	0.016
P50395	Rab GDP dissociation inhibitor beta	4	2.66	0.15	0.008
Q9BRA2	Thioredoxin domain-containing protein 17	3	2.64	0.13	0.019
P11766	Alcohol dehydrogenase class-3	5	2.64	0.13	0.002
P13796	Plastin-2	4	2.62	0.18	0.012
P22234	Multifunctional protein ADE2	4	2.44	0.07	0.001
P17900	Ganglioside GM2 activator	3	2.38	0.13	0.021
P40967	Melanocyte protein PMEL	3	2.36	0.15	0.031
P33121	Long-chain-fatty-acid—CoA ligase 1	4	2.35	0.14	0.010
Q9H3G5	Probable serine carboxypeptidase CPVL	3	2.35	0.14	0.025
P07858	Cathepsin B	5	2.31	0.20	0.015
P60842	Eukaryotic initiation factor 4A-I	5	2.30	0.08	4.3E-04
O14818	Proteasome subunit alpha type-7	4	2.30	0.15	0.012
P07339	Cathepsin D	5	2.18	0.09	0.001
P14618	Pyruvate kinase PKM	5	2.16	0.11	0.002

Average ratio (UM/control), standard error of mean (SEM), and *p* values (t-test) are shown for proteins significantly elevated only in metastatic UM tumors in LC MS/MS iTRAQ analyses of 5 metastatic and 5 non-metastatic primary UM tumors.

**Table 4:** Proteins Significantly Decreased only in Metastatic UM Tumors.

Uni-Prot	Protein	Sample Frequency	Average Ratio	SEM	*p* value
P68371	Tubulin beta-4B chain	5	0.45	0.06	1.8E-04
O94905	Erlin-2	5	0.44	0.16	0.008
P05023	Sodium/potassium-transporting ATPase subunit alpha-1	5	0.44	0.11	0.002
P05556	Integrin beta-1	5	0.44	0.11	0.002
Q9HBL0	Tensin-1	4	0.42	0.27	0.048
P13987	CD59 glycoprotein	4	0.42	0.24	0.035
P06396	Gelsolin	5	0.42	0.18	0.009
P0C0L5	Complement C4-B	4	0.41	0.16	0.011
P43121	Cell surface glycoprotein MUC18	5	0.40	0.05	5.7E-05
P06899	Histone H2B type 1-J	5	0.40	0.18	0.006
O95865	N(G),N(G)-dimethylarginine dimethylaminohydrolase 2	4	0.40	0.17	0.012
P00738	Haptoglobin	5	0.40	0.11	0.001
P07305	Histone H1.0	4	0.39	0.26	0.036
P62873	Guanine nucleotide-binding protein G(I)/G(S)/G(T) subunit beta-1	3	0.37	0.05	0.002
O00159	Unconventional myosin-Ic	5	0.36	0.06	7.7E-05
O75369	Filamin-B	5	0.33	0.14	0.002
Q13509	Tubulin beta-3 chain	3	0.30	0.26	0.044
Q13425	Beta-2-syntrophin	3	0.30	0.06	0.002
P02511	Alpha-crystallin B chain	5	0.30	0.24	0.007
P02462	Collagen alpha-1(IV) chain	4	0.29	0.33	0.033
Q6NZI2	Polymerase I and transcript release factor	5	0.28	0.16	0.001
Q13642	Four and a half LIM domains protein 1	3	0.27	0.18	0.018
O43301	Heat shock 70 kDa protein 12A	4	0.26	0.05	0.000
P17661	Desmin	3	0.25	0.16	0.014
Q969G5	Protein kinase C delta-binding protein	4	0.23	0.19	0.005
P68366	Tubulin alpha-4A chain	3	0.21	0.13	0.007
Q13885	Tubulin beta-2A chain	3	0.16	0.33	0.032
Q9UBX5	Fibulin-5	4	0.15	0.26	0.005
Q16853	Membrane primary amine oxidase	4	0.12	0.08	1.1E-04
P63211	Guanine nucleotide-binding protein G(T) subunit gamma-T1	3	0.07	0.18	0.005

Average ratio (UM/control), standard error of mean (SEM), and p values (t-test) are shown for proteins significantly decreased only in metastatic tumors in LC MS/MS iTRAQ analyses of 5 metastatic and 5 non-metastatic primary UM tumors.

**Fig 3:**
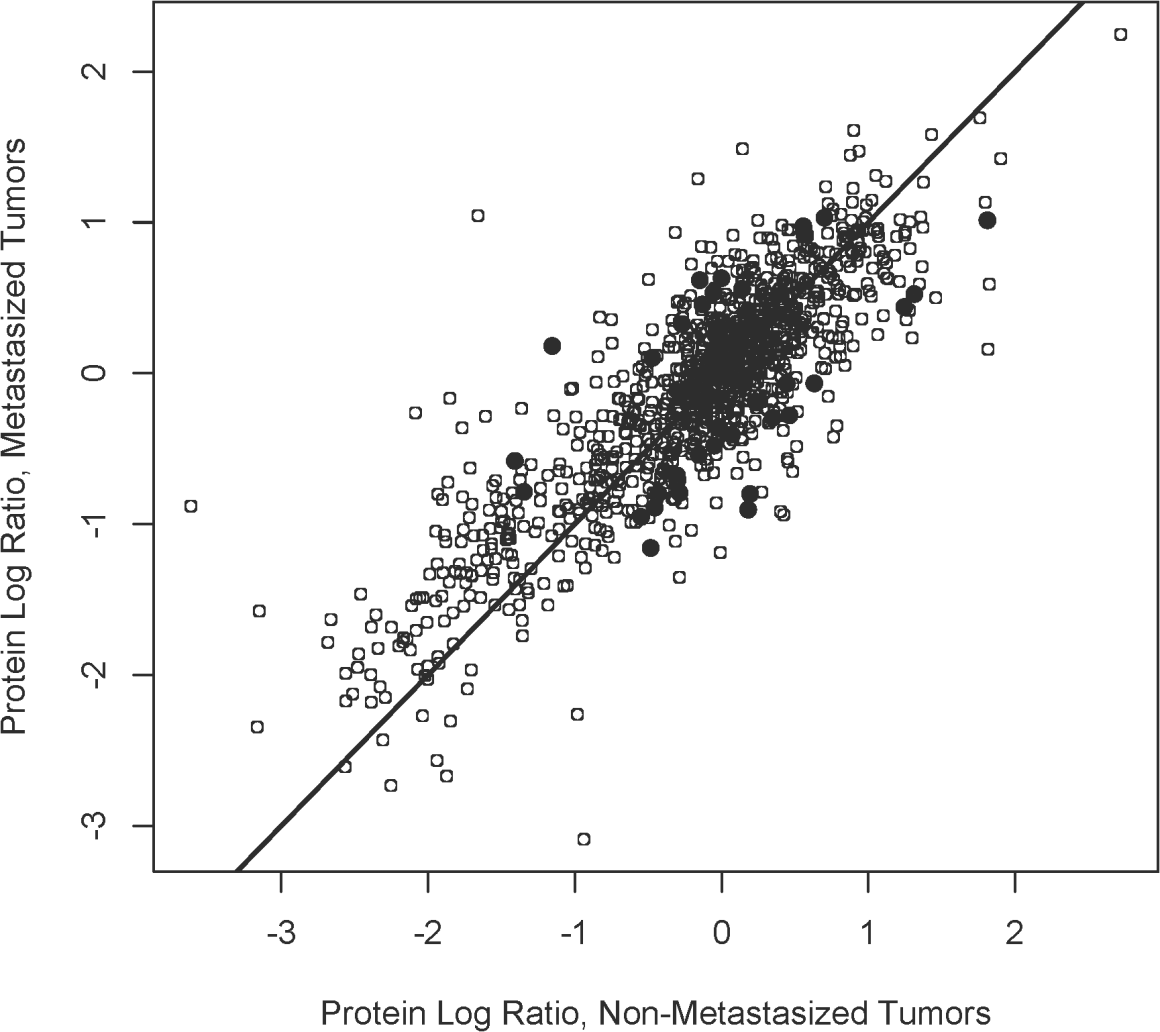
Identification of Differentially Expressed Proteins. Log ratios for proteins (n = 860) present in ≥ 2 specimens from both 5 metastatic and 5 non-metastatic tumors in the training set are plotted (o). Thirty-one proteins exhibiting quantitative differences between metastatic and non-metastatic tumors with p ≤ 0.05 (two sided t-test) were designated differentially expressed (●).

Finally, differentially expressed proteins were sought in the training set among 860 proteins present in ≥ 2 specimens from both metastatic and non-metastatic tumors (Fig 3). Thirty-one proteins were were designated differentially expressed (Table 5) based on quantitative differences with p ≤ 0.05 (two sided t-test). However, multiple bootstrap resampling could not statistically validate significant differences in protein levels (p ≤ 0.05) for any of the 31 proteins, therefore classification as differentially expressed must be considered tentative. A limitation of this study is the relatively small sample size. A larger sample size is required to validate differential expression status; power analysis suggests that 30 metastatic and 30 non-metastatic primary UM tumors would be sufficient to rigorously confirm about 30 differentially expressed proteins.

**Table 5:** Proteins Designated Differentially Expressed.

Uni-Prot	Protein	Frequency Mets	Frequency No Mets	Average Ratio	p-value
P12111	Collagen alpha-3(VI)	5	5	2.3	0.023
Q16698	2,4-dienoyl-CoA reductase, mitochondrial	5	4	2.2	0.041
Q02818	Nucleobindin-1	4	5	1.9	0.021
Q15365	Poly(rC)-binding protein 1	4	5	1.8	0.049
Q00765	Receptor expression-enhancing protein 5	4	5	1.8	0.029
P04179	Superoxide dismutase [Mn], mitochondrial	5	5	1.8	0.033
P40967	Melanocyte protein PMEL	3	5	1.5	0.035
P26599	Polypyrimidine tract-binding protein 1	5	4	1.5	0.041
P13693	Translationally-controlled tumor protein	3	4	1.4	0.039
Q9Y2X3	Nucleolar protein 58	5	4	1.4	0.007
Q8N5K1	CDGSH iron-sulfur domain-containing protein 2	5	5	1.3	0.043
P11142	Heat shock cognate 71 kDa protein	5	5	1.3	0.035
P30153	Serine/threonine-protein phosphatase 2A 65 kDa regulatory subunit A alpha	4	5	0.7	0.045
O43707	Alpha-actinin-4	5	5	0.7	0.049
Q8WUM4	Programmed cell death 6-interacting protein	5	3	0.7	0.005
Q9BRX8	Redox-regulatory protein FAM213A	3	5	0.7	0.044
P0C0S5	Histone H2A.Z	5	5	0.7	0.006
Q14344	Guanine nucleotide-binding protein subunit alpha-13	4	4	0.7	0.034
P62280	40S ribosomal protein S11	5	5	0.7	0.048
P05023	Sodium/potassium-transporting ATPase subunit alpha-1	5	5	0.7	0.008
Q99442	Translocation protein SEC62	3	4	0.7	0.030
P02545	Prelamin-A/C	5	5	0.6	0.044
P30041	Peroxiredoxin-6	5	5	0.6	0.006
P62805	Histone H4	5	5	0.6	0.025
P06899	Histone H2B type 1-J	5	5	0.5	0.021
Q92597	Protein NDRG1	3	3	0.5	0.026
P27816	Microtubule-associated protein 4	5	5	0.5	0.040
P00338	L-Lactate dehydrogenase A	5	5	0.5	0.011
P09211	Glutathione S-transferase P	5	5	0.5	0.044
P30086	Phosphatidylethanolamine-binding protein 1	5	5	0.5	0.049
P04792	Heat shock protein beta-1 (HSP 27)	5	5	0.4	0.019

Differentially expressed proteins, identified as shown in Fig 3, exhibited significant abundance differences (p ≤ 0.05, t-test) between 5 metastatic (Mets) and 5 non-metastatic (No Mets) primary tumors without adjustment for multiple comparisons. Average Ratio reflects Mets/No Mets.

### Prediction of Metastasis from Independent Proteomic data from UM Tumors

Each of the 31 proteins tentatively designated differentially expressed were used to develop prediction models to classify the metastatic status of five independent UM specimens, namely metastatic UM 09, 11, 12 and non-metastatic UM 02, 15. The majority of the 31 prediction models exhibited no discriminatory capability, however, logistic regression modeling with collagen alpha-3(VI) and heat shock protein beta-1 (Hsp beta-1) correctly classified the metastatic status of all five independent tumor samples in the test set. In additon, Sample UM15, which contained 29 of the 31 differentially expressed proteins, was correctly classified by 12 other proteins, namely mitochondrial 2,4-dienoyl-CoA reductase, nucleobindin-1, mitochondrial superoxide dismutase [Mn], polypyrimidine tract-binding protein 1, serine/threonine-protein phosphatase 2A regulatory subunit A, redox-regulatory protein FAM213A, histone H2A.Z, 40S ribosomal protein S11, histone H4, microtubule-associated protein 4, L lactate dehydrogenase A, and glutathione S-transferase P. Collagen alpha-3(VI) and Hsp beta-1 were the only proteins that provide accurate metastatic classification of samples UM 02, 09, 11 and 12, each of which contained 12–14 of the differentially expressed proteins. Multivariate prediction using principal component analysis also provided correct metastatic classification of sample UM15 but no other tumor specimen.

## Discussion

Toward a better understanding of mechanisms and biomarkers of UM metastasis, we quantified proteins in 15 primary UM tumors using LC MS/MS iTRAQ technology. Global proteomic analysis of 8 metastatic and 7 non-metastatic tumors allocated into a training set of 10 and and a test set of 5 tumors provided quantitation of over 1700 proteins. Protein quantitation by iTRAQ technology was independently corroborated by immunoblot analysis of eight proteins from tumor and control specimens. Comparative analyses of five metastatic and five non-metastatic specimens in the training set showed the majority of proteins in both tumor types were present in similar amounts but also suggested a number of proteomic differences between tumor types. For example, 12 proteins were unique to multiple metastatic specimens, 28 proteins were uniquely elevated and 30 proteins uniquely decreased in metastatic tumors, and 31 proteins were tentatively designated differentially expressed. Differentially expressed proteins could not be validated by multiple statistical comparisons, however, logistic regression modeling revealed that two of these proteins, namely collagen alpha-3(VI) and heat shock protein beta-1, correctly classified the metastatic status of five independent UM tumors in the test set. Overall, the results suggest insights to mechanisms of UM metastasis and provide a new quantitative proteomic database for comparison with previous UM protein and gene profiling studies and bioinformatic analysis.

### Previous Proteomic Profiling of UM

Previous UM proteomic investigations have emphasized *in vtro* studies [36–41], but also included UM primary tumor tissues [23, 24], UM aqueous humor [42], and UM sera [38]. Quantitative proteomic methods in previous UM studies have included stable isotope labeling with amino acids in culture [40, 41], 2D gel image analysis [23, 24, 37, 39] and label-free LC MS/MS methods [20]. All UM proteomic reports to-date, including the present study, are limited by relatively small sample sizes. Nevertheless, several proteins quantified in the present data warrant discussion in context with previous studies.

Heat shock protein beta-1, also known as heat shock protein 27 (Hsp27), was first reported decreased in UM monosomy 3 primary tumors using 2D gel methods [23]. Subsequently, immunohistochemical analyses demonstrated Hspβ1 to be reduced in monosomy 3 tumors relative to disomy 3 tumors [43]. Two other investigations suggest Hsp beta-1 to be differentially expressed in metastatic UM, an *in vtro* study of UM liver metastases [39], and a preliminary proteomic comparison of primary tumors introduced in a review article [20]. In the present study, Hsp beta-1 appeared to be differentially expressed, exhibited the lowest metastasis/no metastasis ratio (ratio = 0.4), and was an effective predictor of metastasis for five independent UM tumors. Together these studies suggest Hsp beta-1 as a candidate biomarker for UM metastasis.

The only other detailed proteomic study of UM primary tumors focused on 10 metastatic and 15 non-metastatic tumors, used 2D difference gel electrophoresis (2D DIGE), and reported 15 differentially expressed proteins [24]. Differential expression of these proteins was not validated by multiple statistical resampling, however notable similarities exist with the present data. Two proteins more abundant in metastatic tumors in the 2D DIGE data [24] were also significantly elevated only in metastatic tumors in the present study, namely FABP3 and beta-hexosaminidase. Higher levels of FABP3 in metastatic primary tumors has been supported by immunohistochemistry [24] and label-free LC MS/MS methods [20]. The alpha subunit of beta-hexosaminidase was elevated in the 2D-DIGE data [24], while we found the beta subunit of beta-hexosaminidase elevated, as was also reported for UM liver metastases [39]. Previously, tubulin beta and tubulin alpha 1aB were reported reduced in metastatic tumors [24]; in the present data, tubulin alpha and beta isoforms were significantly decreased only in metastatic UM tumors. Also of note, tubulin alpha and beta isoforms have been detected in the secretome of UM cell lines and tubulin autoantibodies detected in UM patient sera [38]. Translation initiation factors reflect another similarity among UM proteomic findings. Eukaryotic translation initiation factor 2–1 was decreased in metastatic tumors in the 2D-DIGE data [24], while eukaryotic translation initiation factor 5A was elevated in vitro in UM liver metastases [39], and in the present data, eukaryotic translation initiation factor 4A-1 was elevated only in metastatic tumors. Together these data suggest FABP3, beta-hexosaminidase subunits, tubulin isoforms and eukaryotic translation initiation factors warrant further evaluation for possible roles in UM metastasis. Three other proteins reported elevated in metastatic UM tumors include vimentin, protein DJ-1 and triosphosphate isomerase [20, 23, 24]. The present results differ in that protein DJ-1 and triosphosphate isomerase were found elevated in both metastatic and non-metastatic tumors while vimentin was not significantly changed in either.

The present findings also exhibit similaries with in vitro UM proteomic studies. Among the proteins we found significantly elevated only in metastatic tumors (Table 3), cathepsin Z and pyruvate kinase PKM were reported differentially expressed in vitro in UM liver metastases [39] and cathepsin Z, cathepsin B, cathepsin D, macrophage-capping protein, melanocyte protein PMEL were elevated in the secretome of primary UM cell lines [38]. In addition, autoantibodies to cathepsin D, macrophage-capping protein and melanocyte protein PMEL have been reported in sera from UM patients [38]. In the present results, melanocyte protein PMEL was designated differentially expressed and more abundant in metastatic than non-metastatic tumors. Among the proteins we found significantly decreased only in metastatic tumors (Table 4), alpha-crystallin B was reported differentially expressed in UM liver metastases [39], and cell surface glycoprotein MUC18 was found in the secretome of multiple UM cell lines [37, 38]. Glutathione S-transferase P, differentially expressed and less abundant in metastatic tumors in the present data (Table 5), was differentially expressed and decreased relative to the parent cell line in UM liver metastases [39] and detected in the secretome of multiple UM cell lines [38].

### Correlation with Gene Expression Profiling

Numerous UM gene expression profiling studies have suggested genetic discriminators of low and high UM metastatic risk. Recently a prospective multicenter study validated a UM gene expression assay for metastatic risk [18] by successfully classifying over 97% of 459 cases of choroidal and ciliary body UM [19]. Five of the proteins quantified in the present study are gene products or closely related products from this discriminatory set of 12 genes [18, 19], namely extracellular matrix protein 1, cadherin-1, leukotriene A4 hydrolase and related gene products eukaryotic translation initiation factor 4A and fragile X mental retardation protein 1. Genes encoding extracellular matrix protein 1 (*ECM1)* and cadherin-1 (*CDH1*) appear up-regulated in high metastatic risk tumors [18, 19], however we detected these proteins in only a few specimens and they were not significantly altered. The gene encoding leukotriene A4 hydrolase (*LTA4H*) appears down-regulated in high metastatic risk tumors [18, 19]; we detected this gene product in only one of eight metastatic tumors. Genes encoding eukaryotic translation initiation factor 1B (*EIF1B*) and fragile X mental retardation, autosomal homolog 1 (*FXR1*), both located on chromosome 3, appear down-regulated in high metastatic risk tumors [18, 19]. In our protein analyses, fragile X mental retardation protein 1, encoded by the related *FMR1* gene on chromosome X, was detected uniquely in three metastatic tumors but was not altered in abundance. We detected eukaryotic translation initiation factor 4A-1, encoded by the related *EIF4A1* gene on chromosome 17, in 5 metastatic and 4 non-metastatic tumors and to be significantly elevated only in metastatic tumors. Apparent protein and gene expression level differences of eukaryotic translation initiation factors [24, 39] and fragile X mental retardation protein 1 may in part reflect a compensatory response to the loss of *EIF1B* and *FXR1* on chromosome 3 in metastatic UM. However, protein and gene expression levels exhibit relatively low correlation (~20%) in mammals [44].

### Bioinformatic Clues to Mechanisms of UM Metastasis

Bioinformatic analyses of proteins detected uniquely in metasized UM primary tumors, or significantly elevated or decreased only in metastatic tumors or differentially expressed were pursued for possible clues to mechanisms of UM metastasis. The 12 proteins detected in 3 or more specimens in only metastatic UM tumors (Table 2) exhibit top molecular and cellular functions associated with cellular assembly, organization and morphology and have all been linked to multiple cancers. The liver is the most common site of UM metastases and liver cancer has been associated with several Table 2 proteins including putative RNA-binding protein Luc7-like 2, microtubule-actin cross-linking factor 1, and apolipoprotein B-100 (from the Catalogue of Somatic Mutations in Cancer, Welcome Trust Sanger Institute, Genome Research Limited, UK).

The 28 proteins elevated only in metastatic tumors (Table 3) exhibit top canonical pathways associated with chondroitin sulfate/dermatan sulfate degradation based on elevated ganglioside GM2 activator and beta-hexosaminidase beta subunit [45]. Top molecular and cellular functions of Table 3 proteins include cancer-related cellular development and cellular growth and proliferation, including proliferation of immune cells in the hematological system. Liver cancer has been linked with several Table 3 proteins [46–48], including tryptophan-tRNA ligase, phospholipase D3, ubiquitin-conjugating enzyme E2 N, beta-hexosaminidase beta subunit, macrophage-capping protein, Rab GDP dissociation inhibitor, multifunctional protein ADE2, melanocyte protein PMEL, long-chain-fatty-acid-CoA ligase 1, eukaryotic initiation factor 4A-1, cathepsin D and pyruvate kinase PKM (Catalogue of Somatic Mutations in Cancer). FABP3, the most abundant protein we found in metastatic tumors, has been implicated in renal cell carcinoma, esophageal adenocarcinoma [49, 50] and as a mammary-derived growth inhibitor [51].

The 30 proteins decreased only in metastatic tumors (Table 4) exhibit top canonical pathways associated with cell junction signaling and remodeling of epithelial adherens junctions based on gelsolin, integrin beta-1, tubulin beta-4B, tubulin beta-3, tubulin alpha-4a, and tubulin beta-2A. These cytoskeletal and cell-cell interaction proteins have been implicated in various cancers [52, 53], including all four detected isoforms of tubulin. Table 4 proteins associated with liver cancer include tubulin beta-4B, integrin beta-1, tensin-1, haptoglobin, unconventional myosin-1c, filamin-B, collagen alpha-1(IV), polymerase I and transcript release factor, four and a half LIM domains protein 1, and desmin.

The 31 proteins designated differentially expressed (Table 5) exhibit top molecular and cellular functions associated with cell death and survival, molecular transport, and cellular assembly and organization. Differentially expressed collagen alpha-3(VI) and Hsp beta-1 were the only promising predictors of metastasis in this study. Over expression of type VI collagen has been correlated with breast, ovarian, gastric and pancreatic cancers [54–58]. We found collagen alpha-3(VI) significantly more abundant in metastatic than non-metastatic UM tumors. Alternative splicing of the collagen alpha-3(VI) gene has been suggested to have a mechanistic role in pancreatic cancer [57], and elevated serum protein levels to possibly offer biomarker potential [58]. Hsp beta-1 was more abundant in non-metastatic UM tumors in the present study. Small heat shock proteins like Hsp beta-1 and alpha-crystallin B (Table 4) undergo physiology-dependent phosphorylation, oligomerization, and proteolysis that induce structural changes and alter the activity of binding partners [59–61]. Heat shock proteins interact with multiple binding partners and appear to function in carcinogenesis but mechanisms remain unresolved [59, 60]. Hsp beta-1 has been suggested to serve as a switch between tumor dormancy and tumor growth in breast cancer [62], mediated through interactions with vascular endothelial growth factor [61, 63]. Whether Hsp beta-1 plays a functional role in UM micrometastasis that lie dormant for decades remains to be determined.

## Conclusions

The present results expand the proteins known to be associated with human UM primary tumors to over 1700, and establish the largest quantitative proteomic database currently available from UM tissues. Although limited in sample size to 8 metastatic and 7 non-metastatic tumors, this global proteomic study provides clues to mechanisms and biomarkers of UM metastasis. Notable similarities with previous UM proteomic and gene profiling findings suggest Hsp beta-1, FABP3, beta-hexosaminidase beta subunit, tubulin isoforms, eukaryotic translation initiation factors and fragile X mental retardation protein 1 warrant further investigation for possible roles in UM metastasis. Logistic regression modeling with collagen alpha-3(VI) and Hsp beta-1 quantitative proteomic data provided the correct metastatic classification of 5 independent tumors, suggesting these proteins as candidate biomarkers of UM metastasis.

## Supporting Information

S1 Table Relative Protein Abundance: Sample UM19. (PDF)

S2 Table Relative Protein Abundance: Sample UM21. (PDF)

S3 Table Relative Protein Abundance: Sample UM24. (PDF)

S4 Table Relative Protein Abundance: Sample UM28. (PDF)

S5 Table Relative Protein Abundance: Sample UM30. (PDF)

S6 Table Relative Protein Abundance: Sample UM13. (PDF)

S7 Table Relative Protein Abundance: Sample UM20. (PDF)

S8 Table Relative Protein Abundance: Sample UM23. (PDF)

S9 Table Relative Protein Abundance: Sample UM25. (PDF)

S10 Table Relative Protein Abundance: Sample UM26. (PDF)

S11 Table Relative Protein Abundance: Sample UM02. (PDF)

S12 Table Relative Protein Abundance: Sample UM09. (PDF)

S13 Table Relative Protein Abundance: Sample UM11. (PDF)

S14 Table Relative Protein Abundance: Sample UM12. (PDF)

S15 Table Relative Protein Abundance: Sample UM15. (PDF)

S16 Table Average Relative Protein Abundance: Metastatic Tumors (Samples UM19, UM21, UM24, UM28, UM30). (PDF)

S17 Table Average Relative Protein Abundance: Non-Metastatic Tumors (Samples UM13, UM20, UM23, UM25, UM26). (PDF)

S18 Table Overall Summary of Quantitative Proteomic Results. (PDF)

S19 Table Relative Abundance: Proteins Detected Only in Metastatic UM Tumors. (PDF)

S20 Table Relative Abundance: Proteins Detected Only in Non-Metastatic UM Tumors. (PDF)
