# Supplementary material for: iTRAQ Quantitative Proteomic Comparison of Metastatic and Non-Metastatic Uveal Melanoma Tumors
Source: PLoS One. 2015 Aug 25;10(8):e0135543. doi: 10.1371/journal.pone.0135543 (PMC4549237; doi:10.1371/journal.pone.0135543)
Supplement: S1 Table — (PDF) [file pone.0135543.s001.pdf]

Supplementary Table S1
Relative Protein Abundance: Sample UM19, Metastatic
Total Proteins Quantified = 899; LogMedian Protein Ratio =0.13; LogMeanProtein Ratio = 0; Standard Deviation = 0.97

Uni-Prot Accession	Protein	Ratio UM/Control	Standard Deviation	*p* value	Unique Peptides	% Sequence Coverage
P23381	Tryptophan--tRNA ligase, cytoplasmic	13.98	0.162	3.0E-09	13	25.9
P52566	Rho GDP-dissociation inhibitor 2	10.32	0.151	2.7E-07	4	25.4
P62937	Peptidyl-prolyl cis-trans isomerase A	8.13	0.085	0.0E+00	7	38.2
P31146	Coronin-1A	6.73	0.256	8.0E-04	6	15.2
P32455	Interferon-induced guanylate-binding protein 1	6.58	0.214	9.2E-04	9	15.0
Q9UL46	Proteasome activator complex subunit 2	6.45	0.111	2.2E-05	5	22.6
P18463	HLA class I histocompatibility antigen, B-37 alpha chain	5.99	0.269	2.7E-03	3	12.4
P42224	Signal transducer and activator of transcription 1-alpha/beta	5.64	0.098	2.4E-13	17	25.2
P60174	Triosephosphate isomerase	5.56	0.128	1.0E-04	10	38.5
P30086	Phosphatidylethanolamine-binding protein 1	5.50	0.088	3.1E-06	9	55.6
P19971	Thymidine phosphorylase	5.48	0.216	3.0E-04	8	21.8
P29401	Transketolase	5.36	0.122	6.3E-05	9	14.6
P40121	Macrophage-capping protein	5.33	0.354	1.5E-02	4	16.7
P00558	Phosphoglycerate kinase 1	5.30	0.104	2.2E-11	14	28.8
P78417	Glutathione S-transferase omega-1	5.23	0.127	7.8E-08	13	46.5
P16401	Histone H1.5	5.17	0.085	8.4E-04	4	14.6
P40925	Malate dehydrogenase, cytoplasmic	5.13	0.153	1.6E-05	6	22.2
Q03519	Antigen peptide transporter 2	5.03	0.071	4.4E-06	5	8.6
P23528	Cofilin-1	4.97	0.121	3.9E-05	6	33.1
P06737	Glycogen phosphorylase, liver form	4.96	0.089	8.9E-16	24	27.0
P30043	Flavin reductase (NADPH)	4.86	0.220	1.1E-02	3	18.9
P52565	Rho GDP-dissociation inhibitor 1	4.81	0.173	2.3E-05	5	22.5
P06733	Alpha-enolase	4.58	0.070	3.6E-11	14	36.2
Q9UI08	Ena/VASP-like protein	4.38	0.290	1.2E-02	4	13.9
P13796	Plastin-2	4.29	0.142	8.8E-07	10	20.7
Q06323	Proteasome activator complex subunit 1	4.27	0.100	9.5E-08	9	41.0
P04439	HLA class I histocompatibility antigen, A-3 alpha chain	4.23	0.344	2.8E-02	3	11.5
Q9BV36	Melanophilin	4.13	0.191	1.4E-02	3	6.7
Q01105	Protein SET	4.08	0.152	4.4E-03	3	14.1
P07195	L-lactate dehydrogenase B chain	4.07	0.068	6.0E-09	7	21.3
Q8IV08	Phospholipase D3	3.95	0.153	3.8E-03	4	8.4
P28838	Cytosol aminopeptidase	3.94	0.133	4.5E-08	17	36.2
P15531	Nucleoside diphosphate kinase A	3.91	0.107	1.6E-05	6	43.4
Q04760	Lactoylglutathione lyase	3.87	0.280	9.4E-03	3	14.7
P07858	Cathepsin B	3.84	0.089	4.7E-06	5	15.9
P04080	Cystatin-B	3.82	0.171	1.3E-02	3	39.8
Q13510	Acid ceramidase	3.76	0.155	2.7E-05	6	12.2
Q9Y2S2	Lambda-crystallin homolog	3.75	0.220	2.3E-02	7	22.3
O15533	Tapasin	3.67	0.146	7.5E-04	3	6.7
P04406	Glyceraldehyde-3-phosphate dehydrogenase	3.64	0.064	2.2E-10	8	30.4
Q03518	Antigen peptide transporter 1	3.64	0.303	2.2E-02	5	7.2
P06865	Beta-hexosaminidase subunit alpha	3.61	0.166	2.3E-02	3	5.1
P50395	Rab GDP dissociation inhibitor beta	3.60	0.145	7.6E-04	6	16.4
P63241	Eukaryotic translation initiation factor 5A-1	3.60	0.229	1.5E-02	5	30.5
O14818	Proteasome subunit alpha type-7	3.53	0.146	2.8E-03	6	23.8
P20618	Proteasome subunit beta type-1	3.48	0.161	6.5E-05	4	21.6
P06744	Glucose-6-phosphate isomerase	3.44	0.249	5.6E-03	7	14.7
P61204	ADP-ribosylation factor 3	3.37	0.176	5.5E-03	3	17.7
Q96KP4	Cytosolic non-specific dipeptidase	3.34	0.093	3.0E-05	6	16.0
P08758	Annexin A5	3.31	0.054	1.3E-15	10	31.3
P63104	14-3-3 protein zeta/delta	3.28	0.112	1.3E-05	5	26.5
P14618	Pyruvate kinase PKM	3.27	0.083	1.0E-09	12	25.0
Q86UX7	Fermitin family homolog 3	3.26	0.279	2.0E-02	4	9.1
P60842	Eukaryotic initiation factor 4A-I	3.25	0.110	4.9E-05	5	14.5
O15400	Syntaxin-7	3.24	0.081	1.6E-04	4	17.2
P23526	Adenosylhomocysteinase	3.21	0.195	4.6E-04	8	21.1
Q13185	Chromobox protein homolog 3	3.19	0.213	1.7E-02	3	18.0
P40967	Melanocyte protein PMEL	3.17	0.143	9.6E-06	3	4.7
O75874	Isocitrate dehydrogenase [NADP] cytoplasmic	3.17	0.133	2.2E-03	5	11.8
P07686	Beta-hexosaminidase subunit beta	3.16	0.140	4.9E-04	5	7.7
P08670	Vimentin	3.12	0.038	0.0E+00	25	50.9
Q99497	Protein DJ-1	3.10	0.124	1.0E-03	5	24.3
Q9Y3Z3	Deoxynucleoside triphosphate triphosphohydrolase SAMHD1	3.08	0.067	5.0E-09	9	18.1
P02768	Serum albumin	3.07	0.039	0.0E+00	37	52.7
P07900	Heat shock protein HSP 90-alpha	3.02	0.049	2.8E-11	9	10.2
P09960	Leukotriene A-4 hydrolase	2.96	0.063	5.3E-03	3	4.3
P36543	V-type proton ATPase subunit E 1	2.96	0.147	3.6E-03	3	15.5
P18669	Phosphoglycerate mutase 1	2.94	0.067	1.5E-08	6	37.0
P01903	HLA class II histocompatibility antigen, DR alpha chain	2.94	0.086	3.7E-06	4	21.3
P08238	Heat shock protein HSP 90-beta	2.92	0.112	1.1E-04	4	5.4
Q13838	Spliceosome RNA helicase DDX39B	2.89	0.183	2.7E-03	7	20.1
P28066	Proteasome subunit alpha type-5	2.82	0.118	1.5E-05	3	21.2
P19338	Nucleolin	2.81	0.074	2.1E-07	8	10.8
P16152	Carbonyl reductase [NADPH] 1	2.79	0.206	5.5E-03	4	20.2
P13639	Elongation factor 2	2.77	0.102	7.9E-06	11	12.7
Q07955	Serine/arginine-rich splicing factor 1	2.74	0.147	6.9E-03	3	8.1
Q96AG4	Leucine-rich repeat-containing protein 59	2.74	0.138	9.4E-04	4	13.7
O75083	WD repeat-containing protein 1	2.70	0.093	9.1E-07	4	6.8
P06753	Tropomyosin alpha-3 chain	2.70	0.093	2.2E-07	4	13.0
O95336	6-phosphogluconolactonase	2.70	0.190	1.1E-02	4	20.9
P22087	rRNA 2'-O-methyltransferase fibrillarin	2.64	0.218	4.6E-02	3	9.7
Q9NRV9	Heme-binding protein 1	8.76	NA	NA	2	13.8
P06454	Prothymosin alpha	7.05	NA	NA	2	21.6
Q15181	Inorganic pyrophosphatase	6.67	NA	NA	2	10.0
O75347	Tubulin-specific chaperone A	5.91	NA	NA	2	16.7
Q9UHB6	LIM domain and actin-binding protein 1	5.69	0.644	2.0E-01	3	6.6
Q9BQE5	Apolipoprotein L2	5.46	NA	NA	2	6.5
P61088	Ubiquitin-conjugating enzyme E2 N	5.38	NA	NA	2	13.8
O75368	SH3 domain-binding glutamic acid-rich-like protein	4.98	NA	NA	2	20.2
P13686	Tartrate-resistant acid phosphatase type 5	4.96	NA	NA	2	5.8
P55008	Allograft inflammatory factor 1	4.95	NA	NA	2	15.6
P30273	High affinity immunoglobulin epsilon receptor subunit gamma	4.65	NA	NA	2	19.8
P20042	Eukaryotic translation initiation factor 2 subunit 2	4.65	NA	NA	2	9.9
P31939	Bifunctional purine biosynthesis protein PURH	4.48	NA	NA	2	4.4
P49863	Granzyme K	4.18	NA	NA	2	10.6
P12955	Xaa-Pro dipeptidase	4.13	NA	NA	2	3.7
P07108	Acyl-CoA-binding protein	4.04	0.320	8.9E-02	3	50.6
P10599	Thioredoxin	4.02	NA	NA	2	20.0
P11766	Alcohol dehydrogenase class-3	3.93	NA	NA	2	7.0
Q13637	Ras-related protein Rab-32	3.78	NA	NA	2	10.2
Q9UBR2	Cathepsin Z	3.71	0.333	8.6E-02	3	9.6
P30040	Endoplasmic reticulum resident protein 29	3.45	NA	NA	2	7.7
P57729	Ras-related protein Rab-38	3.44	NA	NA	2	8.1
P13693	Translationally-controlled tumor protein	3.36	NA	NA	2	15.7
P53999	Activated RNA polymerase II transcriptional coactivator p15	3.35	NA	NA	2	18.9
Q9Y2Q3	Glutathione S-transferase kappa 1	3.23	NA	NA	2	11.5
Q9BRA2	Thioredoxin domain-containing protein 17	3.18	NA	NA	2	19.5
Q01518	Adenylyl cyclase-associated protein 1	3.16	NA	NA	2	5.7

P04233	HLA class II histocompatibility antigen gamma chain	3.13	0.271	8.9E-02	3	13.9
P17900	Ganglioside GM2 activator	3.12	NA	NA	2	9.3
P07737	Profilin-1	3.09	0.126	9.2E-02	8	65.0
Q9Y2X3	Nucleolar protein 58	3.01	NA	NA	2	6.4
O14556	Glyceraldehyde-3-phosphate dehydrogenase, testis-specific	3.01	NA	NA	2	6.9
O00115	Deoxyribonuclease-2-alpha	2.99	NA	NA	2	6.4
P22234	Multifunctional protein ADE2	2.93	NA	NA	2	4.5
P17096	High mobility group protein HMG-I/HMG-Y	2.86	NA	NA	2	23.4
Q6IAN0	Dehydrogenase/reductase SDR family member 7B	2.85	NA	NA	2	7.1
Q9Y2W1	Thyroid hormone receptor-associated protein 3	2.84	NA	NA	2	2.4
P07741	Adenine phosphoribosyltransferase	2.81	NA	NA	2	12.8
Q15942	Zyxin	2.79	NA	NA	2	5.4
P46776	60S ribosomal protein L27a	2.75	NA	NA	2	14.2
P61769	Beta-2-microglobulin	2.69	NA	NA	2	16.8
Q00796	Sorbitol dehydrogenase	2.67	NA	NA	2	2.8
P02787	Serotransferrin	2.63	0.071	1.6E-12	20	30.5
Q9H2U2	Inorganic pyrophosphatase 2, mitochondrial	2.63	NA	NA	2	7.2
P50453	Serpin B9	2.60	NA	NA	2	8.5
P06748	Nucleophosmin	2.57	0.115	1.8E-05	6	21.8
Q15631	Translin	2.57	NA	NA	2	11.4
P55786	Puromycin-sensitive aminopeptidase	2.53	0.228	2.6E-02	5	7.0
P07339	Cathepsin D	2.52	0.098	2.1E-05	7	18.0
P63279	SUMO-conjugating enzyme UBC9	2.52	NA	NA	2	14.6
E9PAV3	Nascent polypeptide-associated complex subunit alpha, muscle-specific form	2.49	NA	NA	2	1.3
P25786	Proteasome subunit alpha type-1	2.47	0.193	1.8E-02	5	16.0
P25788	Proteasome subunit alpha type-3	2.46	0.072	1.4E-03	3	10.2
P58546	Myotrophin	2.45	NA	NA	2	25.4
P07910	Heterogeneous nuclear ribonucleoproteins C1/C2	2.44	0.085	1.3E-07	7	22.5
P22314	Ubiquitin-like modifier-activating enzyme 1	2.43	0.089	7.7E-04	6	6.8
P62750	60S ribosomal protein L23a	2.40	0.116	1.4E-03	5	27.6
Q63HN8	E3 ubiquitin-protein ligase RNF213	2.39	NA	NA	2	0.5
Q5SNT6	WASH complex subunit FAM21B	2.36	NA	NA	2	2.1
P05107	Integrin beta-2	2.36	0.197	4.1E-03	5	7.4
P15121	Aldose reductase	2.35	NA	NA	2	9.2
P11310	Medium-chain specific acyl-CoA dehydrogenase, mitochondrial	2.33	0.224	7.1E-02	3	7.4
P31153	S-adenosylmethionine synthase isoform type-2	2.31	NA	NA	2	7.6
P09211	Glutathione S-transferase P	2.28	0.174	3.3E-02	7	40.5
Q99729	Heterogeneous nuclear ribonucleoprotein A/B	2.27	NA	NA	2	6.6
Q06787	Fragile X mental retardation protein 1	2.25	NA	NA	2	4.3
P46926	Glucosamine-6-phosphate isomerase 1	2.25	0.036	1.2E-05	3	10.0
P09429	High mobility group protein B1	2.24	0.086	1.6E-03	6	27.0
P30101	Protein disulfide-isomerase A3	2.23	0.054	2.5E-10	15	28.1
O00154	Cytosolic acyl coenzyme A thioester hydrolase	2.23	0.194	2.7E-02	4	15.8
P40926	Malate dehydrogenase, mitochondrial	2.23	0.054	7.9E-08	10	32.0
Q9HC38	Glyoxalase domain-containing protein 4	2.23	0.215	1.5E-01	4	11.8
O00299	Chloride intracellular channel protein 1	2.23	NA	NA	2	7.5
P04179	Superoxide dismutase [Mn], mitochondrial	2.22	0.060	4.6E-04	6	23.4
Q9BZZ5	Apoptosis inhibitor 5	2.22	NA	NA	2	3.4
P10619	Lysosomal protective protein	2.21	0.115	2.2E-02	4	7.7
Q7Z6Z7	E3 ubiquitin-protein ligase HUWE1	2.21	NA	NA	2	0.7
Q9GZY8	Mitochondrial fission factor	2.21	NA	NA	2	6.4
Q14103	Heterogeneous nuclear ribonucleoprotein D0	2.16	NA	NA	2	6.8
Q9NYL4	Peptidyl-prolyl cis-trans isomerase FKBP11	2.16	NA	NA	2	10.4
O15145	Actin-related protein 2/3 complex subunit 3	2.16	0.085	5.2E-02	3	15.7
P46940	Ras GTPase-activating-like protein IQGAP1	2.15	0.112	4.3E-06	11	7.7
P30044	Peroxiredoxin-5, mitochondrial	2.15	0.061	1.7E-03	3	16.8
Q15907	Ras-related protein Rab-11B	2.14	0.135	1.0E-02	5	22.5
P26641	Elongation factor 1-gamma	2.13	0.097	1.7E-03	4	8.9
P49189	4-trimethylaminobutyraldehyde dehydrogenase	2.13	NA	NA	2	3.8
P62158	Calmodulin	2.12	0.096	4.5E-04	4	29.5
P38606	V-type proton ATPase catalytic subunit A	2.12	0.137	1.7E-03	8	15.1
P23284	Peptidyl-prolyl cis-trans isomerase B	2.11	0.075	8.9E-05	10	38.9
P61160	Actin-related protein 2	2.11	0.094	5.6E-05	7	23.9
Q12906	Interleukin enhancer-binding factor 3	2.10	0.094	1.0E-02	6	8.5
P30740	Leukocyte elastase inhibitor	2.10	0.552	9.1E-02	5	20.6
Q15046	Lysine--tRNA ligase	2.08	NA	NA	2	3.7
P31948	Stress-induced-phosphoprotein 1	2.07	0.085	1.3E-05	9	13.3
P09525	Annexin A4	2.07	0.712	7.1E-03	9	21.6
Q9UNH7	Sorting nexin-6	2.06	0.691	3.2E-01	3	3.9
P33121	Long-chain-fatty-acid--CoA ligase 1	2.06	0.088	2.1E-02	3	5.7
Q9UKM9	RNA-binding protein Raly	2.05	0.034	2.5E-06	3	12.7
P51159	Ras-related protein Rab-27A	2.05	0.164	2.6E-02	3	15.4
Q96C19	EF-hand domain-containing protein D2	2.04	NA	NA	2	6.3
Q13488	V-type proton ATPase 116 kDa subunit a isoform 3	2.04	NA	NA	2	3.5
O43399	Tumor protein D54	2.04	NA	NA	2	9.2
Q9BR76	Coronin-1B	2.03	NA	NA	2	3.9
P14550	Alcohol dehydrogenase [NADP(+)]	2.03	0.582	6.5E-01	3	8.0
P54819	Adenylate kinase 2, mitochondrial	2.03	0.110	1.5E-02	3	15.5
P18621	60S ribosomal protein L17	2.02	0.196	5.7E-02	3	18.5
Q9NYU2	UDP-glucose:glycoprotein glucosyltransferase 1	2.02	0.192	1.0E-02	5	2.8
P52907	F-actin-capping protein subunit alpha-1	2.01	0.079	6.3E-05	3	18.5
P37837	Transaldolase	2.01	0.113	2.9E-02	6	17.5
P11216	Glycogen phosphorylase, brain form	2.01	0.122	5.9E-04	7	10.3
Q9H3N1	Thioredoxin-related transmembrane protein 1	2.00	NA	NA	2	7.5
Q9Y6C9	Mitochondrial carrier homolog 2	1.99	NA	NA	2	7.3
P21283	V-type proton ATPase subunit C 1	1.99	NA	NA	2	4.5
Q5T1M5	FK506-binding protein 15	1.98	NA	NA	2	2.1
O43852	Calumenin	1.97	0.216	7.7E-02	3	9.5
Q15084	Protein disulfide-isomerase A6	1.96	0.114	5.8E-03	7	20.2
P59998	Actin-related protein 2/3 complex subunit 4	1.95	0.057	7.0E-04	3	16.1
P31949	Protein S100-A11	1.94	0.117	2.9E-02	3	34.3
P02766	Transthyretin	1.94	NA	NA	2	12.9
P60900	Proteasome subunit alpha type-6	1.94	0.344	2.4E-01	3	13.0
Q92688	Acidic leucine-rich nuclear phosphoprotein 32 family member B	1.92	0.317	9.4E-02	3	10.0
P10644	cAMP-dependent protein kinase type I-alpha regulatory subunit	1.92	NA	NA	2	5.2
P51149	Ras-related protein Rab-7a	1.91	0.093	4.5E-03	3	16.9
O75396	Vesicle-trafficking protein SEC22b	1.91	0.050	2.8E-02	6	34.4
O43776	Asparagine--tRNA ligase, cytoplasmic	1.91	NA	NA	2	5.7
P27797	Calreticulin	1.91	0.173	6.4E-04	10	20.1
Q9BS26	Endoplasmic reticulum resident protein 44	1.90	0.085	4.1E-04	5	13.1
Q00765	Receptor expression-enhancing protein 5	1.90	0.080	1.4E-02	3	10.6
Q02818	Nucleobindin-1	1.89	0.181	1.4E-02	3	8.9
Q9NP81	Serine--tRNA ligase, mitochondrial	1.88	NA	NA	2	6.9
Q9P2E9	Ribosome-binding protein 1	1.87	0.092	2.3E-04	8	7.0
P62263	40S ribosomal protein S14	1.87	0.126	3.1E-03	4	36.4
P39687	Acidic leucine-rich nuclear phosphoprotein 32 family member A	1.87	0.301	1.1E-01	4	14.5
Q13576	Ras GTPase-activating-like protein IQGAP2	1.86	0.190	2.6E-01	3	1.9
Q99798	Aconitate hydratase, mitochondrial	1.86	0.100	6.9E-04	8	13.2
P30504	HLA class I histocompatibility antigen, Cw-4 alpha chain	1.86	NA	NA	2	7.7
P07602	Prosaposin	1.86	0.147	9.6E-02	4	6.9
P61604	10 kDa heat shock protein, mitochondrial	1.85	0.133	1.4E-03	5	49.0
P01859	Ig gamma-2 chain C region	1.85	0.088	5.2E-03	3	12.0
P21281	V-type proton ATPase subunit B, brain isoform	1.85	0.087	1.6E-03	6	11.7
P26038	Moesin	1.84	0.097	5.3E-04	9	15.3
P14317	Hematopoietic lineage cell-specific protein	1.84	0.108	8.0E-03	5	12.6

P30042	ES1 protein homolog, mitochondrial	1.83	NA	NA	2	11.2
P30837	Aldehyde dehydrogenase X, mitochondrial	1.82	0.095	4.1E-04	5	14.1
Q86VP6	Cullin-associated NEDD8-dissociated protein 1	1.80	0.126	1.9E-01	4	3.3
Q99523	Sortilin	1.80	0.073	3.1E-02	4	6.3
P08575	Receptor-type tyrosine-protein phosphatase C	1.80	0.071	1.7E-04	8	7.1
P15311	Ezrin	1.79	0.084	6.5E-04	6	8.7
P00338	L-lactate dehydrogenase A chain	1.79	0.262	3.3E-03	9	25.0
P54136	Arginine--tRNA ligase, cytoplasmic	1.79	0.071	1.1E-03	4	7.4
Q5SSJ5	Heterochromatin protein 1-binding protein 3	1.79	0.572	2.6E-01	3	6.1
Q12905	Interleukin enhancer-binding factor 2	1.77	0.212	1.0E-02	3	9.5
Q13423	NAD(P) transhydrogenase, mitochondrial	1.77	0.099	2.4E-04	5	5.2
P28065	Proteasome subunit beta type-9	1.77	0.329	4.7E-01	3	14.2
P60981	Destrin	1.76	0.078	1.7E-02	3	17.0
Q14974	Importin subunit beta-1	1.76	0.029	1.1E-02	4	7.2
Q9H3G5	Probable serine carboxypeptidase CPVL	1.76	0.083	8.5E-03	3	6.9
Q9UKV3	Apoptotic chromatin condensation inducer in the nucleus	1.76	NA	NA	2	1.7
P13797	Plastin-3	1.75	NA	NA	2	4.0
Q5VTE0	Putative elongation factor 1-alpha-like 3	1.75	0.127	1.3E-03	9	19.9
P37802	Transgelin-2	1.73	0.240	6.2E-02	3	14.6
P99999	Cytochrome c	1.73	0.104	1.4E-02	3	24.8
O14773	Tripeptidyl-peptidase 1	1.72	0.108	1.6E-03	4	8.7
Q9ULV4	Coronin-1C	1.72	NA	NA	2	4.9
P48735	Isocitrate dehydrogenase [NADP], mitochondrial	1.72	0.119	3.8E-04	7	15.3
P25398	40S ribosomal protein S12	1.71	0.081	2.9E-04	3	22.0
O75390	Citrate synthase, mitochondrial	1.71	0.143	2.8E-01	5	12.2
Q04637	Eukaryotic translation initiation factor 4 gamma 1	1.71	NA	NA	2	1.9
P30533	Alpha-2-macroglobulin receptor-associated protein	1.69	NA	NA	2	6.2
P26599	Polypyrimidine tract-binding protein 1	1.68	0.184	8.4E-03	4	5.8
Q86UE4	Protein LYRIC	1.68	NA	NA	2	4.0
O43488	Aflatoxin B1 aldehyde reductase member 2	1.68	NA	NA	2	5.3
P10412	Histone H1.4	1.67	0.078	2.5E-03	5	15.5
O15143	Actin-related protein 2/3 complex subunit 1B	1.67	0.163	8.1E-03	5	17.5
P07237	Protein disulfide-isomerase	1.66	0.069	3.4E-03	12	23.2
P62330	ADP-ribosylation factor 6	1.66	NA	NA	2	14.9
Q8NC51	Plasminogen activator inhibitor 1 RNA-binding protein	1.66	NA	NA	2	6.9
P78347	General transcription factor II-I	1.65	0.273	2.3E-01	3	3.4
Q06830	Peroxiredoxin-1	1.65	0.071	1.5E-04	9	47.7
P62424	60S ribosomal protein L7a	1.65	0.083	9.8E-02	4	16.9
Q5JRX3	Presequence protease, mitochondrial	1.65	NA	NA	2	2.4
O76021	Ribosomal L1 domain-containing protein 1	1.64	NA	NA	2	4.3
P17643	5,6-dihydroxyindole-2-carboxylic acid oxidase	1.64	0.178	2.7E-02	6	13.6
P49591	Serine--tRNA ligase, cytoplasmic	1.64	0.102	1.1E-03	3	7.0
P49755	Transmembrane emp24 domain-containing protein 10	1.64	NA	NA	2	7.3
O15144	Actin-related protein 2/3 complex subunit 2	1.63	0.101	1.2E-02	3	9.0
Q14165	Malectin	1.63	NA	NA	2	5.5
Q00839	Heterogeneous nuclear ribonucleoprotein U	1.63	0.181	2.6E-02	8	10.9
P00352	Retinal dehydrogenase 1	1.62	0.065	5.0E-06	8	17.0
P63000	Ras-related C3 botulinum toxin substrate 1	1.62	NA	NA	2	15.1
Q15691	Microtubule-associated protein RP/EB family member 1	1.62	0.167	7.0E-02	3	9.0
P36578	60S ribosomal protein L4	1.62	0.083	9.4E-04	5	13.8
Q8N5K1	CDGSH iron-sulfur domain-containing protein 2	1.60	0.204	3.9E-02	5	31.9
P52597	Heterogeneous nuclear ribonucleoprotein F	1.60	NA	NA	2	7.0
P04075	Fructose-bisphosphate aldolase A	1.59	0.053	4.0E-06	12	42.3
P38159	RNA-binding motif protein, X chromosome	1.59	1.019	5.0E-01	3	9.0
P08865	40S ribosomal protein SA	1.59	NA	NA	2	9.5
P15153	Ras-related C3 botulinum toxin substrate 2	1.59	NA	NA	2	14.6
Q96D96	Voltage-gated hydrogen channel 1	1.58	NA	NA	2	9.5
P09669	Cytochrome c oxidase subunit 6C	1.57	NA	NA	2	22.7
P62857	40S ribosomal protein S28	1.57	NA	NA	2	30.4
Q14247	Src substrate cortactin	1.57	NA	NA	2	3.5
Q99873	Protein arginine N-methyltransferase 1	1.57	NA	NA	2	6.4
Q9NQP4	Prefoldin subunit 4	1.56	NA	NA	2	17.9
P62851	40S ribosomal protein S25	1.56	0.067	1.1E-03	4	24.0
Q86V81	THO complex subunit 4	1.56	NA	NA	2	8.2
P30048	Thioredoxin-dependent peroxide reductase, mitochondrial	1.55	0.062	1.5E-03	4	19.5
Q07960	Rho GTPase-activating protein 1	1.55	0.140	1.9E-01	4	9.3
P46778	60S ribosomal protein L21	1.54	NA	NA	2	13.8
Q92890	Ubiquitin fusion degradation protein 1 homolog	1.54	NA	NA	2	7.5
Q14152	Eukaryotic translation initiation factor 3 subunit A	1.53	0.149	3.2E-02	5	4.8
P61978	Heterogeneous nuclear ribonucleoprotein K	1.53	0.104	4.9E-04	14	33.7
Q01130	Serine/arginine-rich splicing factor 2	1.53	NA	NA	2	10.9
Q15366	Poly(rC)-binding protein 2	1.52	NA	NA	2	10.1
P09622	Dihydrolipoyl dehydrogenase, mitochondrial	1.52	0.153	2.5E-01	6	12.8
Q9Y4I1	Unconventional myosin-Va	1.52	NA	NA	2	1.0
A1L0T0	Acetolactate synthase-like protein	1.52	0.161	1.4E-01	3	6.0
P62136	Serine/threonine-protein phosphatase PP1-alpha catalytic subunit	1.52	0.186	8.9E-02	3	9.1
Q16531	DNA damage-binding protein 1	1.51	0.112	5.7E-02	4	3.4
P51812	Ribosomal protein S6 kinase alpha-3	1.51	0.234	2.7E-01	3	5.0
P14868	Aspartate--tRNA ligase, cytoplasmic	1.51	0.097	8.9E-03	4	10.0
Q08211	ATP-dependent RNA helicase A	1.51	0.047	1.6E-03	5	4.4
P51991	Heterogeneous nuclear ribonucleoprotein A3	1.51	0.072	1.4E-03	5	14.6
P13667	Protein disulfide-isomerase A4	1.50	0.214	5.6E-02	6	9.5
P61019	Ras-related protein Rab-2A	1.50	0.154	7.2E-02	3	18.4
Q08379	Golgin subfamily A member 2	1.49	NA	NA	2	2.0
P27348	14-3-3 protein theta	1.49	0.045	2.6E-02	4	15.9
P61158	Actin-related protein 3	1.49	0.157	3.8E-02	6	17.7
P51810	G-protein coupled receptor 143	1.49	0.132	1.6E-01	3	9.9
P01857	Ig gamma-1 chain C region	1.49	0.048	4.7E-06	4	17.9
P29692	Elongation factor 1-delta	1.48	0.068	1.4E-02	3	13.9
P62191	26S protease regulatory subunit 4	1.48	NA	NA	2	5.2
O00160	Unconventional myosin-If	1.47	NA	NA	2	1.5
P62258	14-3-3 protein epsilon	1.46	0.081	2.2E-03	7	30.6
P61026	Ras-related protein Rab-10	1.46	NA	NA	2	9.5
Q9Y6N5	Sulfide:quinone oxidoreductase, mitochondrial	1.46	0.077	8.3E-04	5	12.2
P43686	26S protease regulatory subunit 6B	1.46	0.096	7.3E-02	3	8.4
Q07666	KH domain-containing, RNA-binding, signal transduction-associated protein 1	1.46	NA	NA	2	5.2
P29728	2'-5'-oligoadenylate synthase 2	1.46	0.176	1.9E-01	3	5.7
P62249	40S ribosomal protein S16	1.45	NA	NA	2	15.8
Q99536	Synaptic vesicle membrane protein VAT-1 homolog	1.45	0.070	3.5E-03	9	27.0
P11586	C-1-tetrahydrofolate synthase, cytoplasmic	1.45	0.060	5.8E-04	6	7.6
Q3SY69	Mitochondrial 10-formyltetrahydrofolate dehydrogenase	1.45	NA	NA	2	2.3
P26373	60S ribosomal protein L13	1.45	0.139	1.7E-02	3	15.2
P12270	Nucleoprotein TPR	1.45	0.045	1.7E-02	4	2.4
P09651	Heterogeneous nuclear ribonucleoprotein A1	1.45	0.076	2.8E-03	6	20.7
P30519	Heme oxygenase 2	1.44	0.111	6.5E-02	4	18.7
O95292	Vesicle-associated membrane protein-associated protein B/C	1.44	0.235	2.5E-01	3	18.9
P21796	Voltage-dependent anion-selective channel protein 1	1.44	0.101	2.1E-03	8	33.2
Q9Y608	Leucine-rich repeat flightless-interacting protein 2	1.43	NA	NA	2	2.8
Q09028	Histone-binding protein RBBP4	1.43	NA	NA	2	8.2
Q1KMD3	Heterogeneous nuclear ribonucleoprotein U-like protein 2	1.43	0.162	7.3E-02	4	5.2
P14314	Glucosidase 2 subunit beta	1.43	1.244	1.8E-01	9	16.3
P17931	Galectin-3	1.41	0.090	2.0E-03	7	30.8
P26640	Valine--tRNA ligase	1.40	NA	NA	2	2.1
Q9Y266	Nuclear migration protein nudC	1.40	NA	NA	2	9.1
Q99805	Transmembrane 9 superfamily member 2	1.39	0.260	2.2E-01	3	4.2

P22626	Heterogeneous nuclear ribonucleoproteins A2/B1	1.39	0.117	3.5E-03	12	31.7
P11142	Heat shock cognate 71 kDa protein	1.39	0.143	7.6E-03	10	16.7
Q92556	Engulfment and cell motility protein 1	1.39	0.218	2.1E-01	3	5.0
Q9UHX1	Poly(U)-binding-splicing factor PUF60	1.39	NA	NA	2	3.2
O95881	Thioredoxin domain-containing protein 12	1.38	NA	NA	2	14.0
P17844	Probable ATP-dependent RNA helicase DDX5	1.38	0.150	4.6E-02	4	7.7
Q13217	DnaJ homolog subfamily C member 3	1.38	NA	NA	2	3.8
P09874	Poly [ADP-ribose] polymerase 1	1.38	0.136	1.7E-02	7	7.7
Q15029	116 kDa U5 small nuclear ribonucleoprotein component	1.38	0.090	1.5E-01	3	3.7
Q13596	Sorting nexin-1	1.37	0.056	1.5E-01	3	7.1
Q9NYU1	UDP-glucose:glycoprotein glucosyltransferase 2	1.37	NA	NA	2	1.1
P30050	60S ribosomal protein L12	1.37	0.131	9.5E-02	6	54.5
Q5JTV8	Torsin-1A-interacting protein 1	1.37	NA	NA	2	4.5
P62906	60S ribosomal protein L10a	1.37	0.173	3.9E-02	6	27.6
P30084	Enoyl-CoA hydratase, mitochondrial	1.36	0.290	1.3E-01	4	13.1
Q9Y4W6	AFG3-like protein 2	1.36	0.099	1.2E-01	4	4.6
P13010	X-ray repair cross-complementing protein 5	1.36	0.110	1.2E-01	6	11.1
P19367	Hexokinase-1	1.36	0.110	2.2E-02	5	6.1
P50454	Serpin H1	1.36	0.334	2.8E-01	4	11.7
P05155	Plasma protease C1 inhibitor	1.36	NA	NA	2	4.6
P35998	26S protease regulatory subunit 7	1.36	0.114	1.8E-01	3	8.3
P62826	GTP-binding nuclear protein Ran	1.35	NA	NA	2	13.9
P15880	40S ribosomal protein S2	1.35	0.099	4.2E-02	4	16.0
P05387	60S acidic ribosomal protein P2	1.34	0.081	1.3E-02	5	32.2
P51572	B-cell receptor-associated protein 31	1.34	0.144	9.7E-02	5	22.0
P27635	60S ribosomal protein L10	1.34	0.081	2.9E-01	3	15.9
P16070	CD44 antigen	1.34	0.214	5.4E-01	8	10.0
O60506	Heterogeneous nuclear ribonucleoprotein Q	1.34	3.880	5.3E-01	3	5.6
P50213	Isocitrate dehydrogenase [NAD] subunit alpha, mitochondrial	1.34	0.132	4.8E-02	4	11.5
Q15121	Astrocytic phosphoprotein PEA-15	1.34	NA	NA	2	17.7
P36776	Lon protease homolog, mitochondrial	1.34	NA	NA	2	2.4
Q92597	Protein NDRG1	1.34	NA	NA	2	7.6
P0CW22	40S ribosomal protein S17-like	1.33	NA	NA	2	15.6
P62241	40S ribosomal protein S8	1.33	0.122	6.4E-02	4	15.4
O60749	Sorting nexin-2	1.33	NA	NA	2	5.0
P22059	Oxysterol-binding protein 1	1.32	NA	NA	2	2.9
Q16666	Gamma-interferon-inducible protein 16	1.32	0.028	4.2E-02	3	4.1
P60953	Cell division control protein 42 homolog	1.32	NA	NA	2	11.0
P62753	40S ribosomal protein S6	1.31	0.182	1.1E-01	4	16.5
Q99614	Tetratricopeptide repeat protein 1	1.31	NA	NA	2	10.3
P14854	Cytochrome c oxidase subunit 6B1	1.30	0.156	2.2E-01	3	24.4
Q9NQ39	Putative 40S ribosomal protein S10-like	1.30	NA	NA	2	13.6
Q96TC7	Regulator of microtubule dynamics protein 3	1.30	0.188	1.2E-01	3	7.7
P61421	V-type proton ATPase subunit d 1	1.30	0.091	3.9E-02	3	7.7
P34932	Heat shock 70 kDa protein 4	1.29	0.037	8.2E-03	6	7.5
Q16836	Hydroxyacyl-coenzyme A dehydrogenase, mitochondrial	1.29	NA	NA	2	6.1
P43490	Nicotinamide phosphoribosyltransferase	1.29	0.439	6.0E-01	4	7.3
P00441	Superoxide dismutase [Cu-Zn]	1.28	NA	NA	2	13.0
Q13162	Peroxiredoxin-4	1.28	NA	NA	2	8.9
P12956	X-ray repair cross-complementing protein 6	1.27	0.120	7.6E-02	7	11.0
P11021	78 kDa glucose-regulated protein	1.27	0.043	8.1E-05	22	30.7
O75367	Core histone macro-H2A.1	1.27	1.860	1.7E-01	8	22.0
P11387	DNA topoisomerase 1	1.26	NA	NA	2	2.7
O95202	LETM1 and EF-hand domain-containing protein 1, mitochondrial	1.26	0.263	2.3E-01	3	5.3
Q9Y4L1	Hypoxia up-regulated protein 1	1.26	NA	NA	2	2.2
P54727	UV excision repair protein RAD23 homolog B	1.26	0.148	7.8E-02	4	9.3
Q92945	Far upstream element-binding protein 2	1.26	0.311	3.9E-01	4	7.5
P31040	Succinate dehydrogenase [ubiquinone] flavoprotein subunit, mitochondrial	1.26	0.108	1.9E-01	6	12.3
P23246	Splicing factor, proline- and glutamine-rich	1.25	0.780	2.0E-01	5	8.5
P08195	4F2 cell-surface antigen heavy chain	1.25	0.107	3.5E-02	5	9.7
P26368	Splicing factor U2AF 65 kDa subunit	1.25	NA	NA	2	3.8
P84103	Serine/arginine-rich splicing factor 3	1.24	NA	NA	2	18.9
P38117	Electron transfer flavoprotein subunit beta	1.24	6.341	3.2E-01	6	20.0
P08237	ATP-dependent 6-phosphofructokinase, muscle type	1.23	NA	NA	2	3.6
P47756	F-actin-capping protein subunit beta	1.23	0.095	2.9E-02	7	24.5
Q9NZ08	Endoplasmic reticulum aminopeptidase 1	1.23	NA	NA	2	1.6
O75306	NADH dehydrogenase [ubiquinone] iron-sulfur protein 2, mitochondrial	1.23	NA	NA	2	4.3
Q14558	Phosphoribosyl pyrophosphate synthase-associated protein 1	1.22	NA	NA	2	8.4
P61353	60S ribosomal protein L27	1.22	NA	NA	2	22.1
P62979	Ubiquitin-40S ribosomal protein S27a	1.22	0.331	4.8E-01	8	41.7
Q15393	Splicing factor 3B subunit 3	1.22	NA	NA	2	2.5
P78371	T-complex protein 1 subunit beta	1.22	0.136	1.9E-01	7	14.8
P27816	Microtubule-associated protein 4	1.22	0.290	3.6E-01	4	4.3
P38646	Stress-70 protein, mitochondrial	1.22	0.183	3.0E-02	11	19.7
Q9H0U4	Ras-related protein Rab-1B	1.22	NA	NA	2	10.9
O00231	26S proteasome non-ATPase regulatory subunit 11	1.21	0.658	3.9E-01	6	16.1
P17858	ATP-dependent 6-phosphofructokinase, liver type	1.21	0.471	2.7E-01	4	4.6
Q15008	26S proteasome non-ATPase regulatory subunit 6	1.21	NA	NA	2	5.9
Q96GK7	Fumarylacetoacetate hydrolase domain-containing protein 2A	1.21	NA	NA	2	9.6
P02652	Apolipoprotein A-II	1.20	0.182	4.2E-01	3	19.0
Q9UBS4	DnaJ homolog subfamily B member 11	1.20	NA	NA	2	6.4
P40763	Signal transducer and activator of transcription 3	1.20	0.655	4.1E-01	6	11.2
Q15293	Reticulocalbin-1	1.20	0.039	4.7E-02	4	11.2
Q8N163	Cell cycle and apoptosis regulator protein 2	1.19	NA	NA	2	3.0
P18124	60S ribosomal protein L7	1.19	0.109	6.4E-02	4	12.1
P60866	40S ribosomal protein S20	1.19	0.519	5.0E-01	3	25.2
P13073	Cytochrome c oxidase subunit 4 isoform 1, mitochondrial	1.19	0.116	3.8E-01	4	25.4
Q9NSE4	Isoleucine--tRNA ligase, mitochondrial	1.19	0.106	2.4E-01	3	4.2
P08134	Rho-related GTP-binding protein RhoC	1.19	0.209	3.2E-01	3	10.4
P39019	40S ribosomal protein S19	1.19	1.729	7.2E-01	6	31.0
Q6UVK1	Chondroitin sulfate proteoglycan 4	1.19	NA	NA	2	1.4
Q14697	Neutral alpha-glucosidase AB	1.18	0.247	1.9E-01	16	20.2
Q16698	2,4-dienoyl-CoA reductase, mitochondrial	1.18	NA	NA	2	9.3
B5ME19	Eukaryotic translation initiation factor 3 subunit C-like protein	1.18	0.089	8.3E-02	5	5.3
P05388	60S acidic ribosomal protein P0	1.18	0.151	3.1E-01	4	12.0
Q6IAA8	Ragulator complex protein LAMTOR1	1.18	NA	NA	2	16.8
Q14683	Structural maintenance of chromosomes protein 1A	1.17	0.385	3.4E-01	3	2.8
P35606	Coatomer subunit beta'	1.17	14.906	6.8E-01	3	3.2
P01834	Ig kappa chain C region	1.17	0.221	2.3E-01	3	46.2
P10809	60 kDa heat shock protein, mitochondrial	1.17	0.112	2.2E-01	12	24.1
P29590	Protein PML	1.16	0.169	4.8E-01	5	6.6
P02746	Complement C1q subcomponent subunit B	1.16	12.416	4.4E-01	3	15.0
Q9ULA0	Aspartyl aminopeptidase	1.16	NA	NA	2	5.1
P11717	Cation-independent mannose-6-phosphate receptor	1.16	NA	NA	2	1.0
P52272	Heterogeneous nuclear ribonucleoprotein M	1.15	0.375	1.7E-01	7	11.9
O75844	CAAX prenyl protease 1 homolog	1.15	NA	NA	2	3.8
P11940	Polyadenylate-binding protein 1	1.14	0.356	3.0E-01	7	12.3
Q7L5N1	COP9 signalosome complex subunit 6	1.14	NA	NA	2	8.0
O00303	Eukaryotic translation initiation factor 3 subunit F	1.14	NA	NA	2	7.3
O43837	Isocitrate dehydrogenase [NAD] subunit beta, mitochondrial	1.14	NA	NA	2	5.5
O75643	U5 small nuclear ribonucleoprotein 200 kDa helicase	1.14	NA	NA	2	0.9
Q02218	2-oxoglutarate dehydrogenase, mitochondrial	1.14	0.462	1.6E-01	8	9.4
Q86UP2	Kinectin	1.13	0.088	1.2E-01	6	5.7
P00505	Aspartate aminotransferase, mitochondrial	1.13	0.037	6.4E-02	5	13.5
P21912	Succinate dehydrogenase [ubiquinone] iron-sulfur subunit, mitochondrial	1.13	0.404	3.5E-01	3	11.1

P49773	Histidine triad nucleotide-binding protein 1	1.13	NA	NA	2	26.2
P23396	40S ribosomal protein S3	1.13	0.163	1.6E-01	6	24.7
Q99714	3-hydroxyacyl-CoA dehydrogenase type-2	1.12	NA	NA	2	6.9
P08571	Monocyte differentiation antigen CD14	1.12	NA	NA	2	5.3
Q99460	26S proteasome non-ATPase regulatory subunit 1	1.12	NA	NA	2	2.3
P49411	Elongation factor Tu, mitochondrial	1.12	0.338	5.9E-02	10	23.5
P31930	Cytochrome b-c1 complex subunit 1, mitochondrial	1.11	0.292	2.8E-01	5	14.0
O00483	NADH dehydrogenase [ubiquinone] 1 alpha subcomplex subunit 4	1.11	NA	NA	2	22.2
P63261	Actin, cytoplasmic 2	1.11	0.070	3.5E-01	5	16.8
P30049	ATP synthase subunit delta, mitochondrial	1.10	NA	NA	2	13.7
P50991	T-complex protein 1 subunit delta	1.10	1.666	4.7E-01	6	14.8
Q92841	Probable ATP-dependent RNA helicase DDX17	1.10	NA	NA	2	3.0
Q13200	26S proteasome non-ATPase regulatory subunit 2	1.09	0.315	5.2E-01	4	5.2
P55072	Transitional endoplasmic reticulum ATPase	1.09	0.089	1.9E-01	11	16.4
P46782	40S ribosomal protein S5	1.09	NA	NA	2	8.3
P43304	Glycerol-3-phosphate dehydrogenase, mitochondrial	1.08	NA	NA	2	3.4
Q9NSD9	Phenylalanine--tRNA ligase beta subunit	1.08	NA	NA	2	3.1
P20700	Lamin-B1	1.08	0.544	7.5E-01	8	14.7
P06576	ATP synthase subunit beta, mitochondrial	1.08	0.238	2.7E-01	9	20.8
P50990	T-complex protein 1 subunit theta	1.07	0.231	2.6E-01	11	21.4
P11177	Pyruvate dehydrogenase E1 component subunit beta, mitochondrial	1.07	NA	NA	2	4.2
Q99733	Nucleosome assembly protein 1-like 4	1.07	NA	NA	2	7.7
Q92973	Transportin-1	1.07	NA	NA	2	3.2
Q15233	Non-POU domain-containing octamer-binding protein	1.07	0.130	3.4E-01	3	8.1
P55884	Eukaryotic translation initiation factor 3 subunit B	1.07	NA	NA	2	2.3
Q15413	Ryanodine receptor 3	1.07	NA	NA	2	0.3
P47985	Cytochrome b-c1 complex subunit Rieske, mitochondrial	1.06	NA	NA	2	8.0
P46777	60S ribosomal protein L5	1.06	0.415	7.9E-01	3	11.1
Q99623	Prohibitin-2	1.06	0.762	5.2E-01	5	19.4
P39656	Dolichyl-diphosphooligosaccharide--protein glycosyltransferase 48 kDa subunit	1.06	0.155	4.8E-01	5	10.5
Q9UNM6	26S proteasome non-ATPase regulatory subunit 13	1.06	NA	NA	2	4.8
P78527	DNA-dependent protein kinase catalytic subunit	1.06	0.623	8.0E-01	10	2.8
P61247	40S ribosomal protein S3a	1.05	1.378	6.8E-01	4	17.0
Q9Y3U8	60S ribosomal protein L36	1.05	0.375	7.1E-01	4	30.5
Q9Y262	Eukaryotic translation initiation factor 3 subunit L	1.05	NA	NA	2	3.4
Q13011	Delta(3,5)-Delta(2,4)-dienoyl-CoA isomerase, mitochondrial	1.05	NA	NA	2	5.5
Q14980	Nuclear mitotic apparatus protein 1	1.05	0.827	5.7E-01	6	4.0
Q8N1G4	Leucine-rich repeat-containing protein 47	1.04	NA	NA	2	4.3
Q86WV6	Stimulator of interferon genes protein	1.04	NA	NA	2	5.8
P33176	Kinesin-1 heavy chain	1.04	0.323	8.3E-01	4	4.9
P07384	Calpain-1 catalytic subunit	1.04	1.498	9.3E-01	3	4.8
Q02878	60S ribosomal protein L6	1.04	1.367	9.0E-01	7	24.7
Q14847	LIM and SH3 domain protein 1	1.04	NA	NA	2	8.8
P48643	T-complex protein 1 subunit epsilon	1.04	1.023	6.9E-01	9	15.5
P62913	60S ribosomal protein L11	1.03	0.244	8.1E-01	3	16.9
Q9NR28	Diablo homolog, mitochondrial	1.03	NA	NA	2	8.8
Q96I24	Far upstream element-binding protein 3	1.03	NA	NA	2	3.7
P22695	Cytochrome b-c1 complex subunit 2, mitochondrial	1.03	0.334	8.4E-01	5	14.3
Q9Y383	Putative RNA-binding protein Luc7-like 2	1.02	NA	NA	2	4.3
Q96AE4	Far upstream element-binding protein 1	1.02	NA	NA	2	2.5
Q02543	60S ribosomal protein L18a	1.02	NA	NA	2	10.2
Q96QK1	Vacuolar protein sorting-associated protein 35	1.02	NA	NA	2	2.6
P49368	T-complex protein 1 subunit gamma	1.02	0.351	7.2E-01	7	16.1
P53618	Coatomer subunit beta	1.02	NA	NA	2	2.1
P18859	ATP synthase-coupling factor 6, mitochondrial	1.02	0.891	9.0E-01	3	26.9
O00203	AP-3 complex subunit beta-1	1.01	0.460	9.6E-01	3	3.6
Q9H4M9	EH domain-containing protein 1	1.01	NA	NA	2	4.5
P02790	Hemopexin	1.01	0.203	8.9E-01	4	7.6
Q9NTJ5	Phosphatidylinositide phosphatase SAC1	1.01	NA	NA	2	3.4
P27824	Calnexin	1.00	0.230	9.8E-01	12	22.8
P35573	Glycogen debranching enzyme	1.00	NA	NA	2	1.6
Q99832	T-complex protein 1 subunit eta	1.00	0.337	9.8E-01	6	12.3
P35580	Myosin-10	1.00	49.586	9.9E-01	23	12.6
P07919	Cytochrome b-c1 complex subunit 6, mitochondrial	1.00	NA	NA	2	15.4
P36871	Phosphoglucomutase-1	0.99	NA	NA	2	3.0
Q9UHD8	Septin-9	0.99	0.480	9.7E-01	5	9.7
Q86U42	Polyadenylate-binding protein 2	0.99	NA	NA	2	5.6
P29966	Myristoylated alanine-rich C-kinase substrate	0.99	1.544	9.9E-01	6	31.6
P40227	T-complex protein 1 subunit zeta	0.99	0.126	8.6E-01	3	6.0
Q16352	Alpha-internexin	0.99	NA	NA	2	5.2
O75947	ATP synthase subunit d, mitochondrial	0.99	0.502	9.3E-01	5	28.6
P36957	Dihydrolipoyllysine-residue succinyltransferase component of 2-oxoglutarate dehydrogenase complex, mitochondrial	0.98	3.194	8.2E-01	4	10.2
P48047	ATP synthase subunit O, mitochondrial	0.98	0.301	8.8E-01	4	26.3
Q9Y230	RuvB-like 2	0.98	0.194	8.2E-01	3	8.6
Q14258	E3 ubiquitin/ISG15 ligase TRIM25	0.98	NA	NA	2	3.8
Q9P0L0	Vesicle-associated membrane protein-associated protein A	0.98	NA	NA	2	8.4
Q9UIJ7	GTP:AMP phosphotransferase AK3, mitochondrial	0.97	3.550	8.6E-01	4	18.9
Q16891	Mitochondrial inner membrane protein	0.97	0.160	6.9E-01	10	15.2
P62318	Small nuclear ribonucleoprotein Sm D3	0.97	NA	NA	2	24.6
Q00325	Phosphate carrier protein, mitochondrial	0.97	0.438	7.9E-01	5	13.8
P26583	High mobility group protein B2	0.97	NA	NA	2	12.9
P14866	Heterogeneous nuclear ribonucleoprotein L	0.97	1.461	8.3E-01	6	12.6
P82909	28S ribosomal protein S36, mitochondrial	0.97	NA	NA	2	25.2
P62277	40S ribosomal protein S13	0.97	4.701	7.9E-01	5	27.8
Q93050	V-type proton ATPase 116 kDa subunit a isoform 1	0.96	NA	NA	2	2.9
O15173	Membrane-associated progesterone receptor component 2	0.96	0.320	8.3E-01	4	21.5
P30041	Peroxiredoxin-6	0.96	0.619	7.0E-01	6	20.1
Q15041	ADP-ribosylation factor-like protein 6-interacting protein 1	0.96	NA	NA	2	7.9
O60664	Perilipin-3	0.96	NA	NA	2	7.8
P50914	60S ribosomal protein L14	0.96	NA	NA	2	10.7
P08574	Cytochrome c1, heme protein, mitochondrial	0.96	NA	NA	2	7.1
P16615	Sarcoplasmic/endoplasmic reticulum calcium ATPase 2	0.96	0.334	4.6E-01	9	10.1
P27708	CAD protein	0.95	NA	NA	2	1.2
Q08378	Golgin subfamily A member 3	0.95	NA	NA	2	2.4
Q99653	Calcineurin B homologous protein 1	0.95	NA	NA	2	10.3
P62269	40S ribosomal protein S18	0.95	0.409	7.5E-01	5	23.0
O43678	NADH dehydrogenase [ubiquinone] 1 alpha subcomplex subunit 2	0.95	0.838	8.9E-01	3	20.2
Q9BRX8	Redox-regulatory protein FAM213A	0.95	NA	NA	2	8.7
P14625	Endoplasmin	0.95	0.823	5.2E-01	17	22.5
P02763	Alpha-1-acid glycoprotein 1	0.95	5.798	6.8E-01	3	18.9
O75964	ATP synthase subunit g, mitochondrial	0.95	NA	NA	2	27.2
P20674	Cytochrome c oxidase subunit 5A, mitochondrial	0.94	1.205	8.4E-01	4	24.7
O15511	Actin-related protein 2/3 complex subunit 5	0.94	NA	NA	2	11.9
Q9UHQ9	NADH-cytochrome b5 reductase 1	0.94	0.796	7.2E-01	3	9.8
P42765	3-ketoacyl-CoA thiolase, mitochondrial	0.94	0.406	8.9E-01	4	14.9
P04632	Calpain small subunit 1	0.94	NA	NA	2	6.3
Q15075	Early endosome antigen 1	0.94	0.685	5.3E-01	3	2.9
P63244	Guanine nucleotide-binding protein subunit beta-2-like 1	0.94	0.199	8.5E-01	4	12.0
O00232	26S proteasome non-ATPase regulatory subunit 12	0.94	NA	NA	2	4.4
Q16795	NADH dehydrogenase [ubiquinone] 1 alpha subcomplex subunit 9, mitochondrial	0.94	0.448	6.4E-01	3	10.1
P60891	Ribose-phosphate pyrophosphokinase 1	0.93	NA	NA	2	5.7
P17987	T-complex protein 1 subunit alpha	0.93	0.072	2.9E-01	5	9.5
P0CG05	Ig lambda-2 chain C regions	0.93	0.445	7.1E-01	3	46.2
Q13151	Heterogeneous nuclear ribonucleoprotein A0	0.93	1.152	6.2E-01	3	7.5
P24539	ATP synthase F(0) complex subunit B1, mitochondrial	0.92	0.934	6.2E-01	3	13.3

P02647	Apolipoprotein A-I	0.92	0.865	3.2E-01	8	28.1
P04843	Dolichyl-diphosphooligosaccharide--protein glycosyltransferase subunit 1	0.92	0.162	3.0E-01	11	19.3
Q14203	Dynactin subunit 1	0.92	0.071	2.2E-01	3	3.1
P28161	Glutathione S-transferase Mu 2	0.91	NA	NA	2	7.3
Q12907	Vesicular integral-membrane protein VIP36	0.91	NA	NA	2	6.2
P53621	Coatomer subunit alpha	0.91	0.073	1.8E-01	3	2.5
P32969	60S ribosomal protein L9	0.91	0.419	5.6E-01	3	14.6
P49257	Protein ERGIC-53	0.91	0.179	5.9E-01	3	9.6
O43390	Heterogeneous nuclear ribonucleoprotein R	0.91	0.181	4.6E-01	6	11.5
Q05193	Dynamin-1	0.91	0.087	3.6E-01	3	3.1
P39023	60S ribosomal protein L3	0.90	0.722	5.5E-01	3	8.9
P40429	60S ribosomal protein L13a	0.90	NA	NA	2	9.4
Q9Y3I0	tRNA-splicing ligase RtcB homolog	0.89	0.454	6.4E-01	4	8.9
P63208	S-phase kinase-associated protein 1	0.89	1.422	7.4E-01	3	20.9
P35232	Prohibitin	0.89	0.234	8.7E-02	6	23.5
Q6NUK1	Calcium-binding mitochondrial carrier protein SCaMC-1	0.88	0.528	6.9E-01	3	5.2
P47755	F-actin-capping protein subunit alpha-2	0.87	NA	NA	2	12.2
Q13228	Selenium-binding protein 1	0.87	NA	NA	2	4.4
Q99442	Translocation protein SEC62	0.87	NA	NA	2	4.3
P83731	60S ribosomal protein L24	0.87	NA	NA	2	10.8
Q9HDC9	Adipocyte plasma membrane-associated protein	0.87	0.183	3.8E-01	3	7.7
P00390	Glutathione reductase, mitochondrial	0.87	0.232	3.8E-01	3	9.8
O15260	Surfeit locus protein 4	0.87	NA	NA	2	8.6
Q00059	Transcription factor A, mitochondrial	0.87	0.277	4.7E-01	3	7.7
Q01813	ATP-dependent 6-phosphofructokinase, platelet type	0.86	NA	NA	2	3.2
P46781	40S ribosomal protein S9	0.86	1.101	6.6E-01	3	9.8
P49748	Very long-chain specific acyl-CoA dehydrogenase, mitochondrial	0.86	0.119	1.8E-01	5	7.6
P54652	Heat shock-related 70 kDa protein 2	0.85	NA	NA	2	4.4
P02774	Vitamin D-binding protein	0.85	NA	NA	2	3.4
P55084	Trifunctional enzyme subunit beta, mitochondrial	0.85	0.106	5.7E-02	5	10.8
O95865	N(G),N(G)-dimethylarginine dimethylaminohydrolase 2	0.85	NA	NA	2	9.1
P24534	Elongation factor 1-beta	0.85	NA	NA	2	7.1
Q9UQE7	Structural maintenance of chromosomes protein 3	0.85	0.099	2.4E-01	3	2.3
Q8IVF2	Protein AHNAK2	0.85	NA	NA	2	0.4
P35637	RNA-binding protein FUS	0.85	0.129	1.3E-01	4	10.6
P50402	Emerin	0.84	NA	NA	2	9.8
P56556	NADH dehydrogenase [ubiquinone] 1 alpha subcomplex subunit 6	0.84	NA	NA	2	15.6
O14950	Myosin regulatory light chain 12B	0.84	NA	NA	2	12.2
P01625	Ig kappa chain V-IV region Len	0.84	NA	NA	2	21.1
P04844	Dolichyl-diphosphooligosaccharide--protein glycosyltransferase subunit 2	0.84	NA	NA	2	4.1
O43865	Putative adenosylhomocysteinase 2	0.83	0.259	3.5E-01	4	7.0
O14735	CDP-diacylglycerol--inositol 3-phosphatidyltransferase	0.83	NA	NA	2	9.9
P50151	Guanine nucleotide-binding protein G(I)/G(S)/G(O) subunit gamma-10	0.83	NA	NA	2	26.5
P05362	Intercellular adhesion molecule 1	0.82	0.176	2.1E-01	4	10.5
P40939	Trifunctional enzyme subunit alpha, mitochondrial	0.82	0.131	3.9E-02	14	23.1
Q9H3Z4	DnaJ homolog subfamily C member 5	0.82	NA	NA	2	8.1
P62244	40S ribosomal protein S15a	0.82	NA	NA	2	12.3
P09496	Clathrin light chain A	0.81	0.550	7.2E-01	3	8.9
P16435	NADPH--cytochrome P450 reductase	0.81	NA	NA	2	3.0
Q14764	Major vault protein	0.81	0.325	3.2E-01	4	5.0
P11279	Lysosome-associated membrane glycoprotein 1	0.81	NA	NA	2	4.1
P47813	Eukaryotic translation initiation factor 1A, X-chromosomal	0.80	NA	NA	2	13.9
P25705	ATP synthase subunit alpha, mitochondrial	0.80	0.112	9.1E-03	15	31.3
P10606	Cytochrome c oxidase subunit 5B, mitochondrial	0.80	1.013	5.1E-01	3	15.5
P09543	2',3'-cyclic-nucleotide 3'-phosphodiesterase	0.80	0.260	3.5E-01	5	11.4
P51648	Fatty aldehyde dehydrogenase	0.80	NA	NA	2	4.5
Q14108	Lysosome membrane protein 2	0.80	NA	NA	2	4.6
O75489	NADH dehydrogenase [ubiquinone] iron-sulfur protein 3, mitochondrial	0.80	NA	NA	2	9.1
Q6UXV4	Apolipoprotein O-like	0.80	NA	NA	2	11.6
P00367	Glutamate dehydrogenase 1, mitochondrial	0.79	0.168	1.1E-01	4	8.8
P48681	Nestin	0.79	NA	NA	2	1.5
P21266	Glutathione S-transferase Mu 3	0.79	NA	NA	2	9.3
Q86VB7	Scavenger receptor cysteine-rich type 1 protein M130	0.79	0.191	2.1E-01	3	2.9
P01860	Ig gamma-3 chain C region	0.78	0.181	2.4E-01	3	6.4
P67870	Casein kinase II subunit beta	0.78	NA	NA	2	10.2
P67936	Tropomyosin alpha-4 chain	0.78	0.413	1.9E-01	8	25.0
O43707	Alpha-actinin-4	0.78	0.061	2.3E-04	18	23.9
P62701	40S ribosomal protein S4, X isoform	0.78	0.227	4.8E-01	3	13.7
P24752	Acetyl-CoA acetyltransferase, mitochondrial	0.78	0.097	1.2E-01	5	16.2
P45880	Voltage-dependent anion-selective channel protein 2	0.78	0.084	4.9E-03	4	17.0
Q14204	Cytoplasmic dynein 1 heavy chain 1	0.77	0.086	3.0E-03	24	6.0
P35221	Catenin alpha-1	0.77	NA	NA	2	3.4
P58876	Histone H2B type 1-D	0.77	NA	NA	2	7.9
P36542	ATP synthase subunit gamma, mitochondrial	0.77	NA	NA	2	7.0
Q16695	Histone H3.1t	0.76	0.087	5.3E-02	4	19.1
Q7KZF4	Staphylococcal nuclease domain-containing protein 1	0.76	0.796	4.6E-01	5	7.4
Q92499	ATP-dependent RNA helicase DDX1	0.76	0.377	3.3E-01	3	4.2
O94919	Endonuclease domain-containing 1 protein	0.75	0.147	7.0E-02	3	8.4
Q9Y2J2	Band 4.1-like protein 3	0.75	NA	NA	2	3.3
P61225	Ras-related protein Rap-2b	0.74	NA	NA	2	10.9
P04839	Cytochrome b-245 heavy chain	0.74	NA	NA	2	1.9
Q9BSJ8	Extended synaptotagmin-1	0.73	0.222	1.2E-01	4	4.0
Q9BQE3	Tubulin alpha-1C chain	0.73	0.231	6.3E-02	3	8.7
Q6DD88	Atlastin-3	0.72	0.119	1.7E-01	5	9.2
Q53GQ0	Estradiol 17-beta-dehydrogenase 12	0.72	0.064	1.9E-02	3	11.2
Q9NP97	Dynein light chain roadblock-type 1	0.72	NA	NA	2	29.2
Q13561	Dynactin subunit 2	0.72	0.319	1.5E-01	6	19.0
O00264	Membrane-associated progesterone receptor component 1	0.71	0.142	2.3E-02	4	16.4
Q9NQC3	Reticulon-4	0.71	0.062	5.6E-04	3	2.7
P63092	Guanine nucleotide-binding protein G(s) subunit alpha isoforms short	0.71	0.266	2.5E-01	3	12.2
P00918	Carbonic anhydrase 2	0.70	NA	NA	2	10.8
P01023	Alpha-2-macroglobulin	0.70	0.110	1.4E-01	5	4.5
P14927	Cytochrome b-c1 complex subunit 7	0.69	NA	NA	2	15.3
O94979	Protein transport protein Sec31A	0.69	0.557	3.2E-01	3	2.6
Q27J81	Inverted formin-2	0.69	NA	NA	2	1.6
P02545	Prelamin-A/C	0.69	0.046	3.8E-10	30	47.3
Q9P0M6	Core histone macro-H2A.2	0.69	NA	NA	2	8.6
Q02252	Methylmalonate-semialdehyde dehydrogenase [acylating], mitochondrial	0.68	NA	NA	2	4.9
Q12797	Aspartyl/asparaginyl beta-hydroxylase	0.68	0.121	1.4E-01	3	2.9
P05091	Aldehyde dehydrogenase, mitochondrial	0.68	0.073	3.3E-03	3	6.0
A0FGR8	Extended synaptotagmin-2	0.68	NA	NA	2	3.0
P01009	Alpha-1-antitrypsin	0.67	0.103	1.4E-03	9	22.5
P09382	Galectin-1	0.67	0.110	5.7E-04	6	46.7
P68871	Hemoglobin subunit beta	0.67	0.056	3.3E-04	5	44.9
P62280	40S ribosomal protein S11	0.67	0.571	4.4E-01	3	17.1
Q07065	Cytoskeleton-associated protein 4	0.66	0.049	1.5E-03	4	8.6
P10515	Dihydrolipoyllysine-residue acetyltransferase component of pyruvate dehydrogenase complex, mitochondrial	0.66	NA	NA	2	2.6
P17612	cAMP-dependent protein kinase catalytic subunit alpha	0.66	0.285	2.4E-01	3	6.8
Q9Y639	Neuroplastin	0.65	0.237	2.3E-01	3	9.3
O60313	Dynamin-like 120 kDa protein, mitochondrial	0.65	0.416	3.2E-01	5	6.8
P04040	Catalase	0.65	0.068	2.9E-01	3	7.2
Q8NBS9	Thioredoxin domain-containing protein 5	0.64	0.187	2.3E-01	5	10.6
Q9Y490	Talin-1	0.64	0.049	1.1E-08	26	14.0
P46939	Utrophin	0.64	NA	NA	2	0.9
P35579	Myosin-9	0.63	0.039	2.9E-15	63	28.4

P55060	Exportin-2	0.63	NA	NA	2	2.3
Q9H223	EH domain-containing protein 4	0.63	0.070	9.3E-02	3	5.0
P09497	Clathrin light chain B	0.63	0.113	1.3E-01	4	15.3
P00387	NADH-cytochrome b5 reductase 3	0.62	0.072	4.3E-03	6	22.3
Q15149	Plectin	0.62	0.049	8.4E-12	52	12.6
Q9UHG3	Prenylcysteine oxidase 1	0.62	0.094	2.6E-02	4	8.5
P60660	Myosin light polypeptide 6	0.62	0.069	2.3E-07	9	58.3
Q96CW1	AP-2 complex subunit mu	0.61	NA	NA	2	4.6
P29992	Guanine nucleotide-binding protein subunit alpha-11	0.61	0.120	2.1E-01	3	11.1
Q16181	Septin-7	0.61	0.034	8.0E-04	7	20.6
P13861	cAMP-dependent protein kinase type II-alpha regulatory subunit	0.61	0.080	3.9E-02	4	13.9
Q9Y277	Voltage-dependent anion-selective channel protein 3	0.61	NA	NA	2	7.4
O75915	PRA1 family protein 3	0.61	NA	NA	2	9.6
P61163	Alpha-centractin	0.60	0.169	2.5E-02	3	8.5
P50995	Annexin A11	0.60	0.177	4.2E-02	4	7.5
O75131	Copine-3	0.60	NA	NA	2	4.3
O60716	Catenin delta-1	0.60	NA	NA	2	2.3
P54920	Alpha-soluble NSF attachment protein	0.59	0.648	2.4E-01	5	18.6
P50502	Hsc70-interacting protein	0.59	0.348	1.8E-01	4	9.8
O94760	N(G),N(G)-dimethylarginine dimethylaminohydrolase 1	0.59	NA	NA	2	9.1
O94905	Erlin-2	0.58	0.141	1.1E-02	5	16.2
O95782	AP-2 complex subunit alpha-1	0.58	NA	NA	2	2.0
Q15019	Septin-2	0.58	0.091	4.1E-03	6	20.5
Q14956	Transmembrane glycoprotein NMB	0.57	NA	NA	2	3.3
P62805	Histone H4	0.57	0.066	9.1E-10	7	52.4
P23634	Plasma membrane calcium-transporting ATPase 4	0.57	0.316	2.6E-01	3	3.3
Q99572	P2X purinoceptor 7	0.57	NA	NA	2	3.5
Q9HD20	Manganese-transporting ATPase 13A1	0.55	0.723	2.2E-01	3	3.2
Q16270	Insulin-like growth factor-binding protein 7	0.55	NA	NA	2	10.3
P35611	Alpha-adducin	0.55	NA	NA	2	4.5
P35613	Basigin	0.55	NA	NA	2	8.3
Q14254	Flotillin-2	0.54	0.187	7.5E-02	3	7.5
P08133	Annexin A6	0.54	0.052	1.9E-13	27	40.4
P12111	Collagen alpha-3(VI) chain	0.54	0.055	2.1E-11	39	13.8
P30153	Serine/threonine-protein phosphatase 2A 65 kDa regulatory subunit A alpha isoform	0.54	0.142	8.6E-02	3	4.9
P0C0S5	Histone H2A.Z	0.53	NA	NA	2	18.8
P49458	Signal recognition particle 9 kDa protein	0.53	NA	NA	2	22.1
Q9UPN3	Microtubule-actin cross-linking factor 1, isoforms 1/2/3/5	0.53	NA	NA	2	0.4
Q8WUM4	Programmed cell death 6-interacting protein	0.53	0.123	3.0E-02	3	3.7
P17655	Calpain-2 catalytic subunit	0.52	NA	NA	2	2.6
P04899	Guanine nucleotide-binding protein G(i) subunit alpha-2	0.52	0.154	2.6E-03	4	14.6
P08107	Heat shock 70 kDa protein 1A/1B	0.51	0.055	1.1E-06	10	19.5
Q05682	Caldesmon	0.51	0.133	2.1E-02	4	6.6
O43491	Band 4.1-like protein 2	0.50	0.120	1.3E-03	8	10.7
O14495	Lipid phosphate phosphohydrolase 3	0.50	NA	NA	2	3.9
P07099	Epoxide hydrolase 1	0.50	0.051	7.6E-07	8	16.3
P35222	Catenin beta-1	0.50	0.556	2.4E-01	4	6.4
Q00610	Clathrin heavy chain 1	0.50	0.086	5.7E-10	26	17.1
Q14192	Four and a half LIM domains protein 2	0.50	NA	NA	2	7.9
P09936	Ubiquitin carboxyl-terminal hydrolase isozyme L1	0.50	0.915	3.7E-01	5	28.3
Q03591	Complement factor H-related protein 1	0.49	NA	NA	2	9.4
Q15836	Vesicle-associated membrane protein 3	0.49	NA	NA	2	24.0
P68371	Tubulin beta-4B chain	0.49	NA	NA	2	7.2
Q99584	Protein S100-A13	0.49	0.181	1.8E-02	3	30.6
P06396	Gelsolin	0.49	0.161	4.8E-04	11	17.6
Q13418	Integrin-linked protein kinase	0.49	NA	NA	2	4.4
O00468	Agrin	0.49	NA	NA	2	1.1
P69905	Hemoglobin subunit alpha	0.49	0.107	1.4E-05	6	62.0
Q09666	Neuroblast differentiation-associated protein AHNAK	0.48	0.042	0.0E+00	77	12.5
P04217	Alpha-1B-glycoprotein	0.48	NA	NA	2	4.8
P04792	Heat shock protein beta-1	0.47	0.134	2.0E-03	6	31.2
P42167	Lamina-associated polypeptide 2, isoforms beta/gamma	0.47	0.216	2.7E-01	4	10.8
P13987	CD59 glycoprotein	0.47	NA	NA	2	15.6
P12814	Alpha-actinin-1	0.47	0.047	4.5E-07	10	14.1
Q9HBL0	Tensin-1	0.47	NA	NA	2	2.1
P54709	Sodium/potassium-transporting ATPase subunit beta-3	0.47	0.147	5.5E-02	3	13.6
P0C0L5	Complement C4-B	0.46	0.139	6.1E-03	4	1.9
P12109	Collagen alpha-1(VI) chain	0.46	0.102	1.4E-04	11	12.4
P23229	Integrin alpha-6	0.45	NA	NA	2	2.1
Q07954	Prolow-density lipoprotein receptor-related protein 1	0.45	0.138	5.3E-05	9	2.6
Q02952	A-kinase anchor protein 12	0.44	0.099	9.0E-05	21	15.7
P80723	Brain acid soluble protein 1	0.44	0.069	3.4E-05	7	63.4
Q03252	Lamin-B2	0.43	0.056	1.2E-07	14	24.3
P09493	Tropomyosin alpha-1 chain	0.43	0.521	7.2E-02	5	12.0
Q14344	Guanine nucleotide-binding protein subunit alpha-13	0.42	0.226	4.8E-02	3	9.0
Q9NR12	PDZ and LIM domain protein 7	0.41	NA	NA	2	4.8
P01024	Complement C3	0.41	0.158	5.9E-04	17	10.5
P20073	Annexin A7	0.41	0.290	5.9E-01	3	8.6
P00167	Cytochrome b5	0.40	0.197	5.4E-02	4	42.5
P00738	Haptoglobin	0.40	0.100	4.2E-04	4	11.3
Q9NZN4	EH domain-containing protein 2	0.39	0.267	2.9E-02	4	7.4
P68032	Actin, alpha cardiac muscle 1	0.39	0.281	9.5E-03	7	28.9
P0C0S8	Histone H2A type 1	0.39	0.411	1.3E-01	3	28.5
O75955	Flotillin-1	0.38	NA	NA	2	4.9
P06899	Histone H2B type 1-J	0.38	NA	NA	2	7.9
P62873	Guanine nucleotide-binding protein G(I)/G(S)/G(T) subunit beta-1	0.37	NA	NA	2	9.4
Q16563	Synaptophysin-like protein 1	0.36	NA	NA	2	10.0
P10301	Ras-related protein R-Ras	0.35	NA	NA	2	10.6
Q5JRA6	Melanoma inhibitory activity protein 3	0.34	NA	NA	2	1.2
Q96CX2	BTB/POZ domain-containing protein KCTD12	0.34	NA	NA	2	5.8
Q00577	Transcriptional activator protein Pur-alpha	0.33	0.300	1.5E-01	3	6.8
P04216	Thy-1 membrane glycoprotein	0.31	NA	NA	2	15.5
P43121	Cell surface glycoprotein MUC18	0.30	0.330	9.9E-02	3	5.6
P61626	Lysozyme C	0.28	NA	NA	2	12.8
P16157	Ankyrin-1	0.27	NA	NA	2	1.5
P50895	Basal cell adhesion molecule	0.26	0.231	7.3E-02	3	7.8
Q6UXB8	Peptidase inhibitor 16	0.26	NA	NA	2	4.3
P41222	Prostaglandin-H2 D-isomerase	0.25	NA	NA	2	12.1
P08311	Cathepsin G	0.25	NA	NA	2	10.6
O43242	26S proteasome non-ATPase regulatory subunit 3	0.24	NA	NA	2	3.0
O94875	Sorbin and SH3 domain-containing protein 2	0.24	0.562	1.4E-01	3	4.6
P07197	Neurofilament medium polypeptide	0.23	NA	NA	2	2.9
P12277	Creatine kinase B-type	0.22	NA	NA	2	4.2
P05186	Alkaline phosphatase, tissue-nonspecific isozyme	0.21	0.956	3.5E-01	3	5.2
P07305	Histone H1.0	0.21	NA	NA	2	9.3
P22105	Tenascin-X	0.20	0.162	9.0E-02	6	1.4
Q9BXN1	Asporin	0.20	NA	NA	2	2.9
Q7Z7G0	Target of Nesh-SH3	0.19	NA	NA	2	2.0
Q969G5	Protein kinase C delta-binding protein	0.18	NA	NA	2	7.3
Q13361	Microfibrillar-associated protein 5	0.17	NA	NA	2	13.3
P07360	Complement component C8 gamma chain	0.17	0.426	2.1E-01	3	23.8
Q6UWY5	Olfactomedin-like protein 1	0.17	NA	NA	2	6.5
Q92777	Synapsin-2	0.16	NA	NA	2	4.1
P02462	Collagen alpha-1(IV) chain	0.16	0.494	1.3E-01	3	2.5
P24844	Myosin regulatory light polypeptide 9	0.16	NA	NA	2	12.2

P26006	Integrin alpha-3	0.14	NA	NA	2	2.5
Q01995	Transgelin	0.14	NA	NA	2	12.9
Q13885	Tubulin beta-2A chain	0.14	NA	NA	2	4.7
Q16853	Membrane primary amine oxidase	0.14	NA	NA	2	2.6
P58166	Inhibin beta E chain	0.13	NA	NA	2	5.7
P43320	Beta-crystallin B2	0.12	0.390	1.9E-01	3	15.6
P21246	Pleiotrophin	0.11	NA	NA	2	9.5
P10643	Complement component C7	0.11	0.436	1.3E-01	3	5.3
Q13509	Tubulin beta-3 chain	0.10	NA	NA	2	4.7
Q9UBX5	Fibulin-5	0.09	NA	NA	2	3.6
P05164	Myeloperoxidase	0.08	0.502	9.1E-02	3	5.1
P10745	Retinol-binding protein 3	0.07	NA	NA	2	1.5
Q03135	Caveolin-1	0.06	NA	NA	2	13.5
P03973	Antileukoproteinase	0.06	NA	NA	2	18.9
P23946	Chymase	0.06	0.427	7.3E-02	3	16.2
P63211	Guanine nucleotide-binding protein G(T) subunit gamma-T1	0.05	NA	NA	2	17.6
P32119	Peroxiredoxin-2	0.38	0.074	3.5E-06	4	18.2
O94832	Unconventional myosin-Id	0.37	0.180	2.6E-02	4	4.4
Q9NZM1	Myoferlin	0.36	0.073	1.4E-04	6	3.4
P12110	Collagen alpha-2(VI) chain	0.35	0.180	3.6E-03	8	10.0
Q01082	Spectrin beta chain, non-erythrocytic 1	0.35	0.041	0.0E+00	61	30.3
P07942	Laminin subunit beta-1	0.35	0.075	1.9E-03	5	2.9
P05023	Sodium/potassium-transporting ATPase subunit alpha-1	0.35	0.068	9.3E-10	22	23.2
P41219	Peripherin	0.34	0.330	9.1E-03	11	23.2
O75369	Filamin-B	0.33	0.163	8.3E-04	5	2.9
P06727	Apolipoprotein A-IV	0.33	0.123	1.5E-04	9	23.7
Q13813	Spectrin alpha chain, non-erythrocytic 1	0.32	0.039	0.0E+00	73	34.6
P05556	Integrin beta-1	0.32	0.173	3.0E-03	4	6.5
P18206	Vinculin	0.29	0.116	1.5E-07	13	14.8
P21333	Filamin-A	0.29	0.045	0.0E+00	52	26.3
P22413	Ectonucleotide pyrophosphatase/phosphodiesterase family member 1	0.28	0.256	2.7E-02	5	5.3
P14555	Phospholipase A2, membrane associated	0.27	0.216	2.8E-02	3	11.1
P04083	Annexin A1	0.27	0.054	0.0E+00	13	40.8
P26447	Protein S100-A4	0.27	0.135	1.2E-03	3	27.7
P07355	Annexin A2	0.25	0.055	0.0E+00	21	47.8
P08572	Collagen alpha-2(IV) chain	0.23	0.294	3.4E-03	6	4.4
Q16555	Dihydropyrimidinase-related protein 2	0.23	0.157	7.0E-05	9	19.2
P46821	Microtubule-associated protein 1B	0.22	0.326	1.7E-02	4	2.1
P27105	Erythrocyte band 7 integral membrane protein	0.22	0.134	3.0E-03	7	25.3
P36269	Gamma-glutamyltransferase 5	0.22	0.187	1.5E-04	5	10.6
Q16363	Laminin subunit alpha-4	0.21	0.173	6.7E-03	4	2.6
P02654	Apolipoprotein C-I	0.21	0.103	6.6E-03	3	24.1
O00159	Unconventional myosin-Ic	0.21	0.256	3.5E-02	5	4.9
P02751	Fibronectin	0.21	0.084	7.9E-10	12	6.8
P60903	Protein S100-A10	0.20	0.103	4.3E-07	5	36.1
P00450	Ceruloplasmin	0.20	0.102	3.6E-04	6	6.2
O15230	Laminin subunit alpha-5	0.20	0.155	2.9E-03	14	5.0
P01011	Alpha-1-antichymotrypsin	0.20	0.114	3.9E-07	6	12.5
P11047	Laminin subunit gamma-1	0.20	0.233	2.2E-04	12	8.0
P00747	Plasminogen	0.19	0.168	3.9E-06	6	7.3
P39059	Collagen alpha-1(XV) chain	0.19	0.166	9.9E-04	5	3.8
P04196	Histidine-rich glycoprotein	0.19	0.245	2.8E-03	3	5.3
P98160	Basement membrane-specific heparan sulfate proteoglycan core protein	0.19	0.087	9.5E-13	30	8.8
P02549	Spectrin alpha chain, erythrocytic 1	0.19	0.262	1.2E-02	7	4.0
P08294	Extracellular superoxide dismutase [Cu-Zn]	0.19	0.412	2.8E-02	3	15.4
Q9BXM0	Periaxin	0.18	0.464	1.5E-02	7	3.5
Q9Y6C2	EMILIN-1	0.18	0.254	1.1E-03	7	9.2
Q14112	Nidogen-2	0.18	0.187	1.2E-06	11	8.4
Q05707	Collagen alpha-1(XIV) chain	0.17	0.449	2.4E-02	8	5.1
P02679	Fibrinogen gamma chain	0.16	0.167	4.9E-05	11	23.8
P55268	Laminin subunit beta-2	0.16	0.166	8.0E-05	12	7.3
P21980	Protein-glutamine gamma-glutamyltransferase 2	0.16	0.083	5.9E-10	14	20.7
P01031	Complement C5	0.16	0.274	5.2E-04	8	4.7
P02511	Alpha-crystallin B chain	0.16	0.055	2.4E-05	5	29.1
Q6NZI2	Polymerase I and transcript release factor	0.16	0.133	1.5E-03	5	15.6
P11166	Solute carrier family 2, facilitated glucose transporter member 1	0.15	0.063	2.3E-05	3	5.5
P01871	Ig mu chain C region	0.15	0.161	4.9E-05	9	23.9
P14543	Nidogen-1	0.15	0.200	1.2E-06	10	8.2
P02649	Apolipoprotein E	0.14	0.113	1.1E-05	12	40.1
P04275	von Willebrand factor	0.14	0.176	2.9E-07	10	4.1
P11277	Spectrin beta chain, erythrocytic	0.14	0.183	6.1E-03	9	5.1
Q2UY09	Collagen alpha-1(XXVIII) chain	0.13	0.395	4.2E-02	3	2.3
P01008	Antithrombin-III	0.13	0.134	5.0E-05	5	11.4
P35749	Myosin-11	0.13	0.162	7.9E-11	30	17.1
P51888	Prolargin	0.13	0.120	1.0E-14	10	29.3
Q12805	EGF-containing fibulin-like extracellular matrix protein 1	0.13	0.327	3.2E-02	3	5.7
P39060	Collagen alpha-1(XVIII) chain	0.12	0.224	3.3E-05	6	3.6
P02675	Fibrinogen beta chain	0.12	0.161	1.3E-04	9	24.8
Q14195	Dihydropyrimidinase-related protein 3	0.12	0.159	2.1E-06	9	22.5
P02749	Beta-2-glycoprotein 1	0.12	0.190	1.2E-03	3	10.4
Q15661	Tryptase alpha/beta-1	0.11	0.174	2.8E-08	4	17.1
P02686	Myelin basic protein	0.10	0.226	6.4E-05	3	11.2
P02760	Protein AMBP	0.10	0.207	2.6E-06	4	19.0
P22748	Carbonic anhydrase 4	0.09	0.231	1.2E-04	6	23.1
P35625	Metalloproteinase inhibitor 3	0.09	0.227	6.4E-06	5	24.6
P15088	Mast cell carboxypeptidase A	0.09	0.254	6.4E-03	4	8.4
P35555	Fibrillin-1	0.09	0.077	0.0E+00	47	18.7
P02671	Fibrinogen alpha chain	0.08	0.186	1.0E-05	8	10.6
P02730	Band 3 anion transport protein	0.08	0.287	5.0E-03	8	9.8
P02743	Serum amyloid P-component	0.08	0.290	3.5E-05	5	20.6
P51884	Lumican	0.08	0.108	5.1E-13	10	30.2
P10909	Clusterin	0.08	0.111	2.2E-15	15	26.1
P04004	Vitronectin	0.08	0.150	2.8E-12	10	17.6
P07585	Decorin	0.07	0.149	1.4E-05	7	19.5
P21810	Biglycan	0.07	0.123	2.0E-11	7	19.0
P21926	CD9 antigen	0.06	0.203	7.4E-05	3	9.6
P02748	Complement component C9	0.06	0.170	7.5E-06	8	14.5
P22352	Glutathione peroxidase 3	0.06	0.160	1.0E-06	3	11.5
P20774	Mimecan	0.06	0.206	2.5E-08	8	25.8
P08123	Collagen alpha-2(I) chain	0.03	0.167	1.2E-05	3	3.7
P25189	Myelin protein P0	0.03	0.248	3.2E-08	7	27.4

Brown denotes change ≥ 2 standard deviations (SD) from the mean, yellow denotes change ≥ 1 SD and green highlights p values ≤ 0.05. NA, not applicable, n<3 unique peptides.
