# Supplementary material for: iTRAQ Quantitative Proteomic Comparison of Metastatic and Non-Metastatic Uveal Melanoma Tumors
Source: PLoS One. 2015 Aug 25;10(8):e0135543. doi: 10.1371/journal.pone.0135543 (PMC4549237; doi:10.1371/journal.pone.0135543)
Supplement: S2 Table — (PDF) [file pone.0135543.s002.pdf]

Supplementary Table S2
Relative Protein Abundance: Sample UM21, Metastatic
Total Proteins Quantified = 834; Log Median Protein Ratio = 0.135; Log Mean ProteinRatio = 0; Standard Deviation = 0.86

Uni-Prot Accession	Protein	Ratio UM/Control	Standard Deviation	*p* value	Unique Peptides	% Sequence Coverage
P16403	Histone H1.2	7.53	0.087	3.8E-04	7	17.8
P04004	Vitronectin	6.35	0.135	3.5E-12	9	18.8
P26006	Integrin alpha-3	6.32	0.051	3.9E-04	3	3.4
P07900	Heat shock protein HSP 90-alpha	5.46	0.090	6.6E-08	12	13.4
P08865	40S ribosomal protein SA	5.30	0.066	2.1E-06	6	19.7
Q9UH65	Switch-associated protein 70	5.19	0.099	1.5E-03	5	6.2
P04899	Guanine nucleotide-binding protein G(i) subunit alpha-2	5.10	0.098	3.0E-04	3	9.3
P40925	Malate dehydrogenase, cytoplasmic	4.93	0.127	3.3E-06	3	10.5
P19338	Nucleolin	4.69	0.064	6.9E-11	13	17.2
P21980	Protein-glutamine gamma-glutamyltransferase 2	4.57	0.131	1.7E-11	12	15.4
Q99798	Aconitate hydratase, mitochondrial	4.50	0.078	5.9E-03	7	10.6
P06727	Apolipoprotein A-IV	4.49	0.056	5.7E-04	10	20.2
P08572	Collagen alpha-2(IV) chain	4.32	0.116	8.1E-07	6	4.4
Q16555	Dihydropyrimidinase-related protein 2	4.30	0.070	6.6E-09	7	14.5
P61160	Actin-related protein 2	4.21	0.071	7.4E-03	3	9.9
P06748	Nucleophosmin	4.00	0.060	2.7E-06	6	17.7
P30837	Aldehyde dehydrogenase X, mitochondrial	3.94	0.103	6.5E-03	6	16.1
Q00839	Heterogeneous nuclear ribonucleoprotein U	3.82	0.111	5.0E-02	8	8.6
P25189	Myelin protein P0	3.75	0.176	2.1E-07	7	27.4
Q9Y2X3	Nucleolar protein 58	3.68	0.172	1.9E-02	3	8.1
Q12905	Interleukin enhancer-binding factor 2	3.67	0.062	3.2E-06	4	12.3
Q00610	Clathrin heavy chain 1	3.63	0.049	9.3E-10	20	12.7
P14543	Nidogen-1	3.60	0.126	5.9E-08	8	6.7
P31948	Stress-induced-phosphoprotein 1	3.54	0.095	9.5E-07	9	14.2
Q9BXM0	Periaxin	3.53	0.249	2.3E-02	5	1.8
P35555	Fibrillin-1	3.52	0.050	0.0E+00	39	14.0
P50895	Basal cell adhesion molecule	3.52	0.131	1.4E-03	4	9.2
P07741	Adenine phosphoribosyltransferase	3.50	0.043	3.4E-02	3	13.9
Q02818	Nucleobindin-1	3.50	0.083	2.5E-03	4	11.1
P16070	CD44 antigen	3.42	0.158	4.3E-07	6	7.4
P00450	Ceruloplasmin	3.31	0.139	6.9E-06	6	9.3
P62736	Actin, aortic smooth muscle	3.26	0.219	3.9E-04	6	21.0
P07686	Beta-hexosaminidase subunit beta	3.19	0.080	1.1E-05	8	12.6
P98160	Basement membrane-specific heparan sulfate proteoglycan core protein	3.15	0.059	0.0E+00	30	8.2
Q02878	60S ribosomal protein L6	3.13	0.079	3.0E-03	4	13.9
Q99536	Synaptic vesicle membrane protein VAT-1 homolog	3.03	0.113	7.3E-04	11	34.9
Q15661	Tryptase alpha/beta-1	2.86	0.138	2.2E-09	4	17.1
P04040	Catalase	2.85	0.055	1.9E-04	3	6.3
P35580	Myosin-10	2.84	0.076	7.6E-04	21	11.3
Q06830	Peroxiredoxin-1	2.64	0.082	1.1E-03	6	32.2
P51991	Heterogeneous nuclear ribonucleoprotein A3	2.61	0.105	9.8E-03	5	14.3
P30086	Phosphatidylethanolamine-binding protein 1	2.59	0.162	6.5E-04	8	43.3
P26373	60S ribosomal protein L13	2.59	0.052	2.1E-07	5	24.2
P04843	Dolichyl-diphosphooligosaccharide--protein glycosyltransferase subunit 1	2.57	0.077	2.2E-03	9	16.8
P13861	cAMP-dependent protein kinase type II-alpha regulatory subunit	2.54	0.074	7.6E-03	6	18.1
P01024	Complement C3	2.52	0.131	1.5E-06	13	7.9
P30101	Protein disulfide-isomerase A3	2.45	0.041	6.4E-07	14	28.9
O14556	Glyceraldehyde-3-phosphate dehydrogenase, testis-specific	2.44	0.313	9.6E-04	3	9.6
P13489	Ribonuclease inhibitor	2.39	0.184	7.2E-03	6	13.4
P62987	Ubiquitin-60S ribosomal protein L40	6.34	0.695	5.0E-01	8	50.8
P43304	Glycerol-3-phosphate dehydrogenase, mitochondrial	6.16	NA	NA	2	3.4
P31930	Cytochrome b-c1 complex subunit 1, mitochondrial	5.21	0.099	2.5E-01	3	7.7
P51149	Ras-related protein Rab-7a	4.58	0.351	8.1E-01	4	18.8
P04217	Alpha-1B-glycoprotein	4.51	NA	NA	2	4.8
P61353	60S ribosomal protein L27	4.50	NA	NA	2	22.1
Q16698	2,4-dienoyl-CoA reductase, mitochondrial	4.49	0.058	1.2E-01	4	14.3
P22413	Ectonucleotide pyrophosphatase/phosphodiesterase family member 1	4.41	0.429	8.9E-02	3	2.9
Q9BZZ5	Apoptosis inhibitor 5	4.39	NA	NA	2	4.8
P39748	Flap endonuclease 1	4.24	NA	NA	2	4.5
Q01995	Transgelin	4.09	0.221	4.3E-01	3	14.4
P62318	Small nuclear ribonucleoprotein Sm D3	4.07	NA	NA	2	24.6
P30048	Thioredoxin-dependent peroxide reductase, mitochondrial	3.93	0.176	2.8E-01	4	19.5
O75643	U5 small nuclear ribonucleoprotein 200 kDa helicase	3.75	NA	NA	2	1.2
P48426	Phosphatidylinositol 5-phosphate 4-kinase type-2 alpha	3.75	NA	NA	2	6.4
Q15365	Poly(rC)-binding protein 1	3.74	0.253	3.8E-01	4	13.5
Q05682	Caldesmon	3.72	0.382	4.3E-01	4	6.6
Q14980	Nuclear mitotic apparatus protein 1	3.68	0.435	5.3E-01	7	4.0
Q12797	Aspartyl/asparaginyl beta-hydroxylase	3.64	0.440	8.3E-01	6	6.9
P0C0L5	Complement C4-B	3.50	NA	NA	2	1.0
P41219	Peripherin	3.39	0.400	4.7E-01	9	17.9
Q8NBJ7	Sulfatase-modifying factor 2	3.35	NA	NA	2	4.3
Q99584	Protein S100-A13	3.27	0.304	1.4E-01	5	41.8
P69905	Hemoglobin subunit alpha	3.24	0.265	4.0E-01	6	58.5
P45880	Voltage-dependent anion-selective channel protein 2	3.17	0.121	4.2E-01	6	24.5
P26599	Polypyrimidine tract-binding protein 1	3.12	NA	NA	2	3.6
O00567	Nucleolar protein 56	3.12	0.155	7.0E-02	4	6.9
P16157	Ankyrin-1	2.96	NA	NA	2	1.5
O00483	NADH dehydrogenase [ubiquinone] 1 alpha subcomplex subunit 4	2.94	0.169	1.7E-01	3	32.1
P08621	U1 small nuclear ribonucleoprotein 70 kDa	2.88	NA	NA	2	4.6
P22695	Cytochrome b-c1 complex subunit 2, mitochondrial	2.88	0.190	2.9E-01	3	7.9
P14868	Aspartate--tRNA ligase, cytoplasmic	2.84	0.565	8.7E-01	3	7.8
P0CW22	40S ribosomal protein S17-like	2.78	NA	NA	2	15.6
Q13177	Serine/threonine-protein kinase PAK 2	2.78	NA	NA	2	5.9
P43243	Matrin-3	2.77	0.480	3.5E-01	3	4.8
P30085	UMP-CMP kinase	2.70	NA	NA	2	10.7
Q14165	Malectin	2.70	0.074	2.0E-01	3	7.9
Q92890	Ubiquitin fusion degradation protein 1 homolog	2.68	NA	NA	2	7.5
P07360	Complement component C8 gamma chain	2.61	NA	NA	2	15.8
Q9Y4F1	FERM, RhoGEF and pleckstrin domain-containing protein 1	2.60	NA	NA	2	1.9
P52907	F-actin-capping protein subunit alpha-1	2.59	NA	NA	2	10.5
Q9NX63	Coiled-coil-helix-coiled-coil-helix domain-containing protein 3, mitochondrial	2.59	NA	NA	2	9.3
Q7L5N1	COP9 signalosome complex subunit 6	2.55	NA	NA	2	9.5
P62158	Calmodulin	2.50	NA	NA	2	22.1
Q00765	Receptor expression-enhancing protein 5	2.50	NA	NA	2	10.1
P80723	Brain acid soluble protein 1	2.50	NA	NA	2	9.7
P27635	60S ribosomal protein L10	2.49	0.254	2.4E-01	4	19.2
P43490	Nicotinamide phosphoribosyltransferase	2.47	0.471	5.9E-01	4	7.5
P82909	28S ribosomal protein S36, mitochondrial	2.47	NA	NA	2	24.3
P06576	ATP synthase subunit beta, mitochondrial	2.44	0.537	7.5E-01	12	29.1
P08237	ATP-dependent 6-phosphofructokinase, muscle type	2.43	0.655	5.2E-01	4	5.6
O94911	ATP-binding cassette sub-family A member 8	2.42	NA	NA	2	1.2
Q8IY95	Transmembrane protein 192	2.41	NA	NA	2	8.5

Q16363	Laminin subunit alpha-4	2.41	NA	NA	2	1.4
Q7KZF4	Staphylococcal nuclease domain-containing protein 1	2.38	0.466	7.0E-01	3	3.5
P62081	40S ribosomal protein S7	2.38	NA	NA	2	8.8
O75396	Vesicle-trafficking protein SEC22b	2.37	0.308	4.7E-01	3	16.7
P05413	Fatty acid-binding protein, heart	2.36	0.167	2.6E-04	5	33.8
P02748	Complement component C9	2.36	0.172	4.0E-03	6	11.3
P13073	Cytochrome c oxidase subunit 4 isoform 1, mitochondrial	2.35	0.531	6.4E-01	3	19.5
P07910	Heterogeneous nuclear ribonucleoproteins C1/C2	2.32	0.151	1.1E-03	5	16.3
P46779	60S ribosomal protein L28	2.32	0.229	1.6E-01	3	19.0
P27105	Erythrocyte band 7 integral membrane protein	2.31	0.122	1.8E-03	5	17.0
Q9UHQ9	NADH-cytochrome b5 reductase 1	2.30	0.438	6.7E-01	4	15.1
P42858	Huntingtin	2.30	NA	NA	2	0.5
P62424	60S ribosomal protein L7a	2.27	NA	NA	2	6.4
P30049	ATP synthase subunit delta, mitochondrial	2.25	NA	NA	2	13.7
Q12805	EGF-containing fibulin-like extracellular matrix protein 1	2.22	NA	NA	2	3.9
P61626	Lysozyme C	2.22	NA	NA	2	12.8
P05198	Eukaryotic translation initiation factor 2 subunit 1	2.22	NA	NA	2	7.0
P24941	Cyclin-dependent kinase 2	2.22	0.514	1.7E-01	3	11.7
O75131	Copine-3	2.20	0.142	2.6E-02	4	7.6
P11142	Heat shock cognate 71 kDa protein	2.19	0.068	6.6E-05	10	13.8
P02549	Spectrin alpha chain, erythrocytic 1	2.17	0.180	2.9E-03	5	2.5
O14980	Exportin-1	2.15	NA	NA	2	2.2
Q9C0E8	Protein lunapark	2.15	NA	NA	2	3.7
Q01844	RNA-binding protein EWS	2.15	NA	NA	2	2.4
P08294	Extracellular superoxide dismutase [Cu-Zn]	2.13	0.197	5.6E-02	3	15.4
P61247	40S ribosomal protein S3a	2.12	0.211	1.9E-01	5	20.5
O43491	Band 4.1-like protein 2	2.09	0.204	3.2E-02	3	3.1
P27348	14-3-3 protein theta	2.07	0.109	7.7E-03	4	14.3
Q14152	Eukaryotic translation initiation factor 3 subunit A	2.07	0.097	6.0E-02	5	4.1
P22626	Heterogeneous nuclear ribonucleoproteins A2/B1	2.06	0.052	2.1E-05	12	31.7
P61026	Ras-related protein Rab-10	2.05	0.475	9.5E-01	3	12.5
P16615	Sarcoplasmic/endoplasmic reticulum calcium ATPase 2	2.04	0.070	3.1E-02	6	6.0
P51810	G-protein coupled receptor 143	2.03	0.064	6.7E-06	4	11.1
P23526	Adenosylhomocysteinase	2.01	0.196	6.9E-05	7	16.2
O00571	ATP-dependent RNA helicase DDX3X	2.01	NA	NA	2	3.2
O43301	Heat shock 70 kDa protein 12A	2.01	0.148	8.5E-04	3	5.5
P10155	60 kDa SS-A/Ro ribonucleoprotein	2.01	NA	NA	2	3.2
P62917	60S ribosomal protein L8	2.00	NA	NA	2	8.9
P22234	Multifunctional protein ADE2	2.00	NA	NA	2	4.9
P30464	HLA class I histocompatibility antigen, B-15 alpha chain	2.00	0.313	1.3E-01	4	16.0
P19971	Thymidine phosphorylase	1.99	3.632	6.1E-01	3	8.3
P46926	Glucosamine-6-phosphate isomerase 1	1.98	0.086	2.5E-04	5	16.3
Q86VP6	Cullin-associated NEDD8-dissociated protein 1	1.97	0.309	7.3E-02	4	3.5
Q9BSJ8	Extended synaptotagmin-1	1.96	NA	NA	2	2.2
O75369	Filamin-B	1.96	0.184	2.5E-03	7	4.0
P20700	Lamin-B1	1.94	0.990	5.6E-01	5	8.2
P62753	40S ribosomal protein S6	1.94	0.089	1.9E-01	4	15.3
P61604	10 kDa heat shock protein, mitochondrial	1.93	0.128	3.9E-03	3	27.5
P05556	Integrin beta-1	1.93	0.161	1.3E-02	7	9.0
Q9UMS4	Pre-mRNA-processing factor 19	1.92	NA	NA	2	6.7
P46781	40S ribosomal protein S9	1.92	0.525	3.8E-01	4	18.6
P02654	Apolipoprotein C-I	1.91	NA	NA	2	24.1
Q53GQ0	Estradiol 17-beta-dehydrogenase 12	1.91	NA	NA	2	8.3
P42765	3-ketoacyl-CoA thiolase, mitochondrial	1.91	0.227	8.6E-01	3	9.1
O95298	NADH dehydrogenase [ubiquinone] 1 subunit C2	1.90	NA	NA	2	16.8
Q99538	Legumain	1.90	NA	NA	2	5.1
Q15819	Ubiquitin-conjugating enzyme E2 variant 2	1.90	0.143	4.2E-02	4	24.8
P14618	Pyruvate kinase PKM	1.90	0.068	7.3E-13	11	23.2
Q15393	Splicing factor 3B subunit 3	1.90	NA	NA	2	2.1
O76003	Glutaredoxin-3	1.89	NA	NA	2	3.9
P17900	Ganglioside GM2 activator	1.89	NA	NA	2	7.8
Q07666	KH domain-containing, RNA-binding, signal transduction-associated protein 1	1.88	0.112	7.4E-02	4	7.9
P14314	Glucosidase 2 subunit beta	1.88	0.140	1.1E-02	5	8.7
P53990	IST1 homolog	1.87	0.217	4.5E-02	5	10.7
P54652	Heat shock-related 70 kDa protein 2	1.86	0.125	1.9E-02	9	15.0
P04406	Glyceraldehyde-3-phosphate dehydrogenase	1.85	0.067	1.4E-13	9	30.7
P10321	HLA class I histocompatibility antigen, Cw-7 alpha chain	1.84	NA	NA	2	6.3
P57729	Ras-related protein Rab-38	1.84	0.128	1.5E-05	4	17.1
P33121	Long-chain-fatty-acid--CoA ligase 1	1.83	0.130	2.0E-02	4	6.6
Q02543	60S ribosomal protein L18a	1.82	NA	NA	2	10.2
Q6UVK1	Chondroitin sulfate proteoglycan 4	1.82	NA	NA	2	1.2
Q01105	Protein SET	1.82	0.161	1.1E-03	6	21.4
P39019	40S ribosomal protein S19	1.82	0.033	1.4E-03	5	25.5
Q14624	Inter-alpha-trypsin inhibitor heavy chain H4	1.81	0.240	3.7E-02	4	4.1
P78371	T-complex protein 1 subunit beta	1.81	0.134	7.4E-02	7	12.1
P26640	Valine--tRNA ligase	1.81	NA	NA	2	2.1
P04179	Superoxide dismutase [Mn], mitochondrial	1.80	2.468	9.0E-01	5	17.6
P10644	cAMP-dependent protein kinase type I-alpha regulatory subunit	1.80	0.069	2.7E-02	4	12.1
P35998	26S protease regulatory subunit 7	1.80	NA	NA	2	5.1
Q8WVC6	Dephospho-CoA kinase domain-containing protein	1.80	NA	NA	2	10.0
P07585	Decorin	1.80	0.065	1.9E-04	3	8.1
P25705	ATP synthase subunit alpha, mitochondrial	1.80	0.672	1.6E-01	11	22.1
P25786	Proteasome subunit alpha type-1	1.79	0.206	2.7E-03	5	20.2
P55072	Transitional endoplasmic reticulum ATPase	1.78	0.113	1.5E-01	13	17.0
Q99832	T-complex protein 1 subunit eta	1.78	0.285	7.6E-01	5	10.5
Q9HBL0	Tensin-1	1.78	0.036	1.7E-04	3	2.1
P02743	Serum amyloid P-component	1.78	0.186	4.6E-07	6	23.3
P31150	Rab GDP dissociation inhibitor alpha	1.77	NA	NA	2	5.4
Q92905	COP9 signalosome complex subunit 5	1.77	NA	NA	2	6.9
P54819	Adenylate kinase 2, mitochondrial	1.76	0.085	2.9E-04	5	24.3
P0C0S8	Histone H2A type 1	1.74	0.164	1.6E-01	3	28.5
P09211	Glutathione S-transferase P	1.73	0.153	4.5E-01	4	26.7
P01857	Ig gamma-1 chain C region	1.73	0.072	8.2E-03	4	18.5
P16930	Fumarylacetoacetase	1.73	NA	NA	2	5.3
P01903	HLA class II histocompatibility antigen, DR alpha chain	1.72	NA	NA	2	9.8
P23381	Tryptophan--tRNA ligase, cytoplasmic	1.71	0.189	5.0E-03	5	9.6
P24844	Myosin regulatory light polypeptide 9	1.70	NA	NA	2	12.2
Q96C19	EF-hand domain-containing protein D2	1.70	NA	NA	2	6.3
P15311	Ezrin	1.70	NA	NA	2	2.6
P18669	Phosphoglycerate mutase 1	1.70	0.087	6.5E-05	5	33.1
Q9NY33	Dipeptidyl peptidase 3	1.69	NA	NA	2	3.5
Q8N5K1	CDGSH iron-sulfur domain-containing protein 2	1.69	0.302	3.4E-01	3	20.7
P29966	Myristoylated alanine-rich C-kinase substrate	1.67	0.487	6.4E-01	4	15.7
Q86UE4	Protein LYRIC	1.67	NA	NA	2	4.0
Q96JB5	CDK5 regulatory subunit-associated protein 3	1.66	NA	NA	2	3.0
P02686	Myelin basic protein	1.66	NA	NA	2	7.2
P11586	C-1-tetrahydrofolate synthase, cytoplasmic	1.65	0.049	1.5E-04	7	9.2
O14735	CDP-diacylglycerol--inositol 3-phosphatidyltransferase	1.65	NA	NA	2	9.9
P04275	von Willebrand factor	1.65	0.119	4.4E-07	11	4.2

P50402	Emerin	1.65	NA	NA	2	9.8
P08238	Heat shock protein HSP 90-beta	1.64	0.132	1.7E-03	7	10.1
Q969X5	Endoplasmic reticulum-Golgi intermediate compartment protein 1	1.64	NA	NA	2	7.9
O43399	Tumor protein D54	1.64	NA	NA	2	11.7
P48047	ATP synthase subunit O, mitochondrial	1.64	0.113	4.4E-02	3	16.0
O15400	Syntaxin-7	1.64	0.119	6.2E-06	4	17.2
P61421	V-type proton ATPase subunit d 1	1.63	NA	NA	2	5.4
P10909	Clusterin	1.63	0.103	1.6E-15	14	28.5
P01860	Ig gamma-3 chain C region	1.63	24.364	7.2E-01	3	6.4
P50914	60S ribosomal protein L14	1.62	NA	NA	2	10.7
P00167	Cytochrome b5	1.62	0.208	3.9E-02	3	32.1
P00338	L-lactate dehydrogenase A chain	1.61	0.109	1.6E-07	7	17.8
Q15008	26S proteasome non-ATPase regulatory subunit 6	1.61	NA	NA	2	5.9
P60866	40S ribosomal protein S20	1.61	0.120	8.0E-02	3	25.2
P83731	60S ribosomal protein L24	1.61	NA	NA	2	13.4
P11177	Pyruvate dehydrogenase E1 component subunit beta, mitochondrial	1.61	NA	NA	2	4.2
Q9NYU2	UDP-glucose:glycoprotein glucosyltransferase 1	1.61	0.039	1.1E-01	3	2.2
O75915	PRA1 family protein 3	1.59	NA	NA	2	9.6
P18206	Vinculin	1.59	0.108	1.7E-06	11	10.1
P09429	High mobility group protein B1	1.59	0.148	5.4E-02	5	21.9
O94832	Unconventional myosin-Id	1.58	0.403	4.0E-01	4	4.2
P43686	26S protease regulatory subunit 6B	1.57	NA	NA	2	6.0
P11021	78 kDa glucose-regulated protein	1.56	1.263	1.0E+00	17	26.1
P21912	Succinate dehydrogenase [ubiquinone] iron-sulfur subunit, mitochondrial	1.56	19.919	5.1E-01	5	17.5
P36578	60S ribosomal protein L4	1.56	0.180	1.1E-01	7	15.2
P51858	Hepatoma-derived growth factor	1.56	0.189	3.0E-02	4	18.3
Q10567	AP-1 complex subunit beta-1	1.55	NA	NA	2	2.4
P53597	Succinyl-CoA ligase [ADP/GDP-forming] subunit alpha, mitochondrial	1.55	NA	NA	2	6.9
P51884	Lumican	1.55	0.069	0.0E+00	7	20.4
P07737	Profilin-1	1.54	0.154	4.5E-06	4	26.4
P37837	Transaldolase	1.54	0.082	9.3E-03	5	13.1
P09497	Clathrin light chain B	1.54	NA	NA	2	8.3
O60568	Procollagen-lysine,2-oxoglutarate 5-dioxygenase 3	1.53	NA	NA	2	3.9
P09936	Ubiquitin carboxyl-terminal hydrolase isozyme L1	1.53	0.906	3.6E-01	4	21.5
P49458	Signal recognition particle 9 kDa protein	1.52	NA	NA	2	22.1
O14745	Na(+)/H(+) exchange regulatory cofactor NHE-RF1	1.52	NA	NA	2	8.7
Q16891	Mitochondrial inner membrane protein	1.52	1.413	6.9E-01	10	13.9
P07602	Prosaposin	1.52	0.132	1.7E-01	4	6.9
P62244	40S ribosomal protein S15a	1.52	0.089	1.7E-01	3	18.5
P40763	Signal transducer and activator of transcription 3	1.51	9.412	8.5E-01	3	4.4
P07942	Laminin subunit beta-1	1.51	0.116	5.1E-04	3	2.1
P06396	Gelsolin	1.50	0.147	7.2E-03	9	10.2
Q07954	Prolow-density lipoprotein receptor-related protein 1	1.50	0.060	4.4E-06	14	3.8
P55809	Succinyl-CoA:3-ketoacid coenzyme A transferase 1, mitochondrial	1.50	0.161	9.0E-02	4	6.5
P25398	40S ribosomal protein S12	1.49	0.075	7.4E-04	3	25.0
P49327	Fatty acid synthase	1.48	NA	NA	2	1.0
P06744	Glucose-6-phosphate isomerase	1.47	0.162	1.4E-04	6	10.8
Q92896	Golgi apparatus protein 1	1.47	0.606	8.3E-01	3	3.0
Q06323	Proteasome activator complex subunit 1	1.47	0.116	1.8E-04	6	26.1
O75367	Core histone macro-H2A.1	1.47	0.107	3.3E-02	7	23.9
Q05707	Collagen alpha-1(XIV) chain	1.47	0.164	7.9E-05	7	4.1
P08107	Heat shock 70 kDa protein 1A/1B	1.47	0.063	4.7E-04	8	15.3
P50502	Hsc70-interacting protein	1.47	NA	NA	2	5.1
O75390	Citrate synthase, mitochondrial	1.47	0.111	8.5E-03	5	11.8
Q9P2E9	Ribosome-binding protein 1	1.46	0.078	5.2E-04	9	7.4
P61204	ADP-ribosylation factor 3	1.46	NA	NA	2	10.5
Q13185	Chromobox protein homolog 3	1.46	NA	NA	2	10.9
P14854	Cytochrome c oxidase subunit 6B1	1.46	0.083	1.9E-01	3	24.4
Q15084	Protein disulfide-isomerase A6	1.46	0.042	3.2E-05	5	12.5
P02042	Hemoglobin subunit delta	1.46	0.026	6.3E-02	3	30.6
P28066	Proteasome subunit alpha type-5	1.46	NA	NA	2	12.0
Q07955	Serine/arginine-rich splicing factor 1	1.45	0.126	4.8E-03	3	8.5
P42566	Epidermal growth factor receptor substrate 15	1.45	NA	NA	2	2.8
Q16695	Histone H3.1t	1.44	0.086	3.4E-04	3	14.7
P13796	Plastin-2	1.44	NA	NA	2	3.0
P13987	CD59 glycoprotein	1.44	NA	NA	2	15.6
O75347	Tubulin-specific chaperone A	1.44	1.129	2.3E-01	3	26.9
Q08211	ATP-dependent RNA helicase A	1.43	0.120	3.0E-02	6	5.7
P16219	Short-chain specific acyl-CoA dehydrogenase, mitochondrial	1.43	NA	NA	2	4.6
Q14956	Transmembrane glycoprotein NMB	1.43	4.826	2.0E-01	4	7.5
P08123	Collagen alpha-2(I) chain	1.43	NA	NA	2	2.4
P00747	Plasminogen	1.43	0.215	2.3E-02	5	9.4
O15455	Toll-like receptor 3	1.43	NA	NA	2	2.0
B5ME19	Eukaryotic translation initiation factor 3 subunit C-like protein	1.43	NA	NA	2	2.1
Q13263	Transcription intermediary factor 1-beta	1.43	NA	NA	2	1.9
P63000	Ras-related C3 botulinum toxin substrate 1	1.43	0.044	2.4E-02	4	24.5
P17844	Probable ATP-dependent RNA helicase DDX5	1.42	0.014	6.9E-03	3	4.7
P11047	Laminin subunit gamma-1	1.42	0.103	3.4E-09	12	7.8
Q9NZM1	Myoferlin	1.42	0.076	1.9E-04	8	4.4
P02652	Apolipoprotein A-II	1.42	NA	NA	2	17.0
P13667	Protein disulfide-isomerase A4	1.42	0.225	4.8E-01	5	8.5
P16401	Histone H1.5	1.42	0.395	6.1E-02	3	9.7
Q96AG4	Leucine-rich repeat-containing protein 59	1.42	0.224	2.6E-02	4	12.7
Q15435	Protein phosphatase 1 regulatory subunit 7	1.42	NA	NA	2	5.3
Q86UP2	Kinectin	1.42	0.356	1.2E-01	8	7.3
P05388	60S acidic ribosomal protein P0	1.41	0.053	8.6E-02	3	10.7
P39687	Acidic leucine-rich nuclear phosphoprotein 32 family member A	1.40	0.165	2.3E-01	4	12.9
P35232	Prohibitin	1.40	0.062	3.2E-03	7	25.4
P26639	Threonine--tRNA ligase, cytoplasmic	1.40	NA	NA	2	2.9
P61158	Actin-related protein 3	1.40	0.082	1.5E-02	6	16.3
P09496	Clathrin light chain A	1.39	0.525	7.0E-01	3	8.9
P11310	Medium-chain specific acyl-CoA dehydrogenase, mitochondrial	1.39	0.159	2.0E-01	3	7.4
P55145	Mesencephalic astrocyte-derived neurotrophic factor	1.39	NA	NA	2	11.0
P09622	Dihydrolipoyl dehydrogenase, mitochondrial	1.39	NA	NA	2	3.3
P31040	Succinate dehydrogenase [ubiquinone] flavoprotein subunit, mitochondrial	1.38	0.059	3.2E-02	3	4.8
O60716	Catenin delta-1	1.37	NA	NA	2	2.0
P09382	Galectin-1	1.37	0.074	4.9E-02	5	34.8
Q96QK1	Vacuolar protein sorting-associated protein 35	1.37	NA	NA	2	2.3
P50990	T-complex protein 1 subunit theta	1.36	0.269	7.3E-01	12	22.3
P14866	Heterogeneous nuclear ribonucleoprotein L	1.36	0.248	2.4E-01	4	8.5
P61225	Ras-related protein Rap-2b	1.36	0.075	7.1E-02	3	16.4
Q15019	Septin-2	1.36	0.145	3.3E-02	5	21.9
Q14697	Neutral alpha-glucosidase AB	1.36	0.162	9.1E-02	10	11.8
Q8TAQ2	SWI/SNF complex subunit SMARCC2	1.36	0.069	4.6E-02	3	2.4
P02765	Alpha-2-HS-glycoprotein	1.36	NA	NA	2	3.5
P22105	Tenascin-X	1.35	NA	NA	2	0.8
Q9UHG3	Prenylcysteine oxidase 1	1.35	0.060	1.4E-02	4	8.1
Q9BPX5	Actin-related protein 2/3 complex subunit 5-like protein	1.35	NA	NA	2	20.3
Q16531	DNA damage-binding protein 1	1.34	NA	NA	2	1.5

P62826	GTP-binding nuclear protein Ran	1.34	NA	NA	2	9.7
P21399	Cytoplasmic aconitate hydratase	1.34	NA	NA	2	2.2
O95299	NADH dehydrogenase [ubiquinone] 1 alpha subcomplex subunit 10, mitochondrial	1.34	1.972	4.2E-01	3	7.9
P39656	Dolichyl-diphosphooligosaccharide--protein glycosyltransferase 48 kDa subunit	1.33	0.341	3.1E-01	3	7.2
Q8N1G4	Leucine-rich repeat-containing protein 47	1.32	0.198	1.6E-01	5	10.8
P49189	4-trimethylaminobutyraldehyde dehydrogenase	1.32	NA	NA	2	2.6
P0CG05	Ig lambda-2 chain C regions	1.31	NA	NA	2	23.6
Q9UJS0	Calcium-binding mitochondrial carrier protein Aralar2	1.31	NA	NA	2	3.3
P27797	Calreticulin	1.31	0.490	7.6E-01	4	8.6
P17643	5,6-dihydroxyindole-2-carboxylic acid oxidase	1.31	0.113	6.7E-03	7	11.5
P46940	Ras GTPase-activating-like protein IQGAP1	1.31	0.093	2.2E-05	17	11.1
P21283	V-type proton ATPase subunit C 1	1.30	NA	NA	2	3.9
P00387	NADH-cytochrome b5 reductase 3	1.30	0.164	1.1E-01	7	22.3
P62241	40S ribosomal protein S8	1.29	NA	NA	2	5.8
Q9Y2S2	Lambda-crystallin homolog	1.29	NA	NA	2	6.3
P47756	F-actin-capping protein subunit beta	1.29	0.326	5.0E-01	3	15.9
P50454	Serpin H1	1.28	0.088	5.6E-01	4	10.0
Q9NSE4	Isoleucine--tRNA ligase, mitochondrial	1.28	2.135	7.5E-01	4	5.9
Q9HDC9	Adipocyte plasma membrane-associated protein	1.28	0.235	3.7E-01	3	7.9
P68871	Hemoglobin subunit beta	1.28	0.086	2.7E-02	4	38.8
P01009	Alpha-1-antitrypsin	1.27	0.154	2.5E-01	8	22.0
P62263	40S ribosomal protein S14	1.27	0.073	1.8E-03	3	29.8
O95292	Vesicle-associated membrane protein-associated protein B/C	1.27	0.284	4.6E-02	4	19.3
P13671	Complement component C6	1.27	NA	NA	2	2.7
Q13200	26S proteasome non-ATPase regulatory subunit 2	1.27	NA	NA	2	2.3
O15230	Laminin subunit alpha-5	1.27	0.127	4.2E-05	10	3.2
Q1KMD3	Heterogeneous nuclear ribonucleoprotein U-like protein 2	1.27	NA	NA	2	2.5
Q9Y4I1	Unconventional myosin-Va	1.27	0.103	6.1E-03	7	3.8
P52272	Heterogeneous nuclear ribonucleoprotein M	1.26	0.081	9.2E-03	8	13.4
Q03135	Caveolin-1	1.26	NA	NA	2	13.5
P12956	X-ray repair cross-complementing protein 6	1.26	0.132	2.3E-02	8	13.5
P67936	Tropomyosin alpha-4 chain	1.26	0.230	1.4E-02	8	25.8
Q9GZS3	WD repeat-containing protein 61	1.26	NA	NA	2	7.5
Q92945	Far upstream element-binding protein 2	1.26	0.119	4.9E-03	7	12.0
Q9Y230	RuvB-like 2	1.26	0.078	5.8E-02	3	6.7
P07099	Epoxide hydrolase 1	1.26	0.128	7.4E-03	6	13.6
P22059	Oxysterol-binding protein 1	1.26	0.134	9.4E-02	3	4.0
O00299	Chloride intracellular channel protein 1	1.26	NA	NA	2	6.2
Q9Y4L1	Hypoxia up-regulated protein 1	1.26	0.183	3.8E-01	4	4.1
P68366	Tubulin alpha-4A chain	1.26	NA	NA	2	4.2
Q15181	Inorganic pyrophosphatase	1.25	0.090	3.6E-03	4	13.5
Q9UBQ7	Glyoxylate reductase/hydroxypyruvate reductase	1.25	NA	NA	2	4.6
Q9BS40	Latexin	1.25	NA	NA	2	12.2
Q3SY69	Mitochondrial 10-formyltetrahydrofolate dehydrogenase	1.25	NA	NA	2	2.3
P61769	Beta-2-microglobulin	1.24	NA	NA	2	10.9
Q92841	Probable ATP-dependent RNA helicase DDX17	1.24	0.114	4.7E-01	3	5.3
P40429	60S ribosomal protein L13a	1.24	0.307	8.1E-01	5	14.8
Q14195	Dihydropyrimidinase-related protein 3	1.24	0.122	4.2E-05	8	17.0
P38606	V-type proton ATPase catalytic subunit A	1.24	NA	NA	2	3.2
P11413	Glucose-6-phosphate 1-dehydrogenase	1.23	NA	NA	2	3.7
O94875	Sorbin and SH3 domain-containing protein 2	1.23	0.596	1.5E-01	3	3.6
P24752	Acetyl-CoA acetyltransferase, mitochondrial	1.23	0.200	7.9E-02	3	8.0
P00352	Retinal dehydrogenase 1	1.23	NA	NA	2	3.6
P21281	V-type proton ATPase subunit B, brain isoform	1.22	3.080	4.9E-01	3	7.4
P23946	Chymase	1.22	0.421	8.3E-03	4	21.1
Q15907	Ras-related protein Rab-11B	1.22	0.183	5.9E-02	3	12.8
P07358	Complement component C8 beta chain	1.22	NA	NA	2	2.0
P02774	Vitamin D-binding protein	1.22	NA	NA	2	3.4
O95782	AP-2 complex subunit alpha-1	1.22	NA	NA	2	1.9
P62873	Guanine nucleotide-binding protein G(I)/G(S)/G(T) subunit beta-1	1.21	NA	NA	2	6.8
P20774	Mimecan	1.21	0.152	2.3E-10	7	21.8
P04844	Dolichyl-diphosphooligosaccharide--protein glycosyltransferase subunit 2	1.21	0.038	1.1E-02	3	5.4
P29590	Protein PML	1.21	NA	NA	2	2.4
P05141	ADP/ATP translocase 2	1.21	0.097	7.6E-03	4	13.1
P30405	Peptidyl-prolyl cis-trans isomerase F, mitochondrial	1.21	NA	NA	2	7.7
P61981	14-3-3 protein gamma	1.21	NA	NA	2	9.7
Q13423	NAD(P) transhydrogenase, mitochondrial	1.20	0.136	2.6E-02	7	7.4
Q969G5	Protein kinase C delta-binding protein	1.20	0.070	7.4E-03	3	11.5
P22087	rRNA 2'-O-methyltransferase fibrillarin	1.20	0.158	5.0E-03	4	14.6
O43852	Calumenin	1.20	0.088	2.3E-04	7	22.5
P04080	Cystatin-B	1.19	0.072	9.5E-06	4	52.0
O15145	Actin-related protein 2/3 complex subunit 3	1.19	NA	NA	2	9.6
P02790	Hemopexin	1.19	0.141	1.2E-01	3	8.9
Q13011	Delta(3,5)-Delta(2,4)-dienoyl-CoA isomerase, mitochondrial	1.19	0.122	1.7E-01	4	10.4
P09417	Dihydropteridine reductase	1.19	NA	NA	2	12.3
P62750	60S ribosomal protein L23a	1.18	0.149	5.5E-03	4	20.5
P02675	Fibrinogen beta chain	1.18	0.102	9.8E-06	8	21.2
Q9UHL4	Dipeptidyl peptidase 2	1.18	0.231	2.3E-03	4	11.4
Q9NX24	H/ACA ribonucleoprotein complex subunit 2	1.18	NA	NA	2	19.0
Q13217	DnaJ homolog subfamily C member 3	1.18	NA	NA	2	3.6
P12270	Nucleoprotein TPR	1.18	0.171	4.0E-01	4	2.5
Q12906	Interleukin enhancer-binding factor 3	1.18	0.119	3.3E-03	6	8.2
P36269	Gamma-glutamyltransferase 5	1.18	0.145	7.9E-03	4	7.3
P54136	Arginine--tRNA ligase, cytoplasmic	1.17	NA	NA	2	3.3
Q9Y376	Calcium-binding protein 39	1.17	NA	NA	2	5.9
Q8NBJ5	Procollagen galactosyltransferase 1	1.16	NA	NA	2	4.0
P49411	Elongation factor Tu, mitochondrial	1.16	0.089	1.8E-02	8	19.5
Q9UBX5	Fibulin-5	1.16	0.179	1.3E-03	3	7.1
Q16181	Septin-7	1.16	0.070	2.9E-04	6	16.2
P27708	CAD protein	1.16	NA	NA	2	0.9
P22314	Ubiquitin-like modifier-activating enzyme 1	1.16	0.138	4.9E-03	7	7.3
P48643	T-complex protein 1 subunit epsilon	1.16	0.136	8.7E-02	8	13.9
P13674	Prolyl 4-hydroxylase subunit alpha-1	1.16	NA	NA	2	4.7
Q13425	Beta-2-syntrophin	1.15	0.066	6.9E-03	3	5.9
O95881	Thioredoxin domain-containing protein 12	1.15	NA	NA	2	14.0
Q9UJZ1	Stomatin-like protein 2, mitochondrial	1.15	NA	NA	2	8.7
Q9UKM9	RNA-binding protein Raly	1.14	0.050	3.5E-03	4	17.0
P07305	Histone H1.0	1.14	NA	NA	2	8.8
O00154	Cytosolic acyl coenzyme A thioester hydrolase	1.14	NA	NA	2	8.2
O75489	NADH dehydrogenase [ubiquinone] iron-sulfur protein 3, mitochondrial	1.14	0.328	6.2E-01	4	18.9
P20042	Eukaryotic translation initiation factor 2 subunit 2	1.14	NA	NA	2	9.9
P42704	Leucine-rich PPR motif-containing protein, mitochondrial	1.13	0.116	2.4E-02	3	2.2
Q15293	Reticulocalbin-1	1.13	0.184	2.0E-01	6	14.2
Q6NUK1	Calcium-binding mitochondrial carrier protein SCaMC-1	1.12	0.219	1.9E-01	3	5.0
P17987	T-complex protein 1 subunit alpha	1.12	0.177	2.6E-01	8	15.5
Q9Y6C2	EMILIN-1	1.12	0.241	7.8E-04	5	6.1
P15121	Aldose reductase	1.12	0.266	3.8E-02	5	16.1
O75368	SH3 domain-binding glutamic acid-rich-like protein	1.12	NA	NA	2	21.9
P54709	Sodium/potassium-transporting ATPase subunit beta-3	1.12	0.105	5.7E-02	3	13.6

P11766	Alcohol dehydrogenase class-3	1.12	0.597	2.2E-01	3	7.2
P36957	Dihydrolipoyllysine-residue succinyltransferase component of 2-oxoglutarate dehydrogenase complex, mitochondrial	1.12	0.169	7.7E-01	5	11.5
Q07960	Rho GTPase-activating protein 1	1.12	0.172	6.5E-03	5	10.7
Q9BTV4	Transmembrane protein 43	1.11	NA	NA	2	6.3
P63241	Eukaryotic translation initiation factor 5A-1	1.11	0.117	3.9E-04	6	33.1
P01031	Complement C5	1.11	0.280	6.5E-02	6	3.6
Q02252	Methylmalonate-semialdehyde dehydrogenase [acylating], mitochondrial	1.11	0.164	3.7E-01	3	5.8
P00488	Coagulation factor XIII A chain	1.10	NA	NA	2	2.6
P07919	Cytochrome b-c1 complex subunit 6, mitochondrial	1.10	NA	NA	2	15.4
P31939	Bifunctional purine biosynthesis protein PURH	1.10	0.200	8.9E-03	3	5.2
P49748	Very long-chain specific acyl-CoA dehydrogenase, mitochondrial	1.09	3.977	5.0E-01	6	9.3
P07954	Fumarate hydratase, mitochondrial	1.09	NA	NA	2	3.7
P30533	Alpha-2-macroglobulin receptor-associated protein	1.09	0.132	2.6E-02	4	10.1
P51159	Ras-related protein Rab-27A	1.09	NA	NA	2	10.9
Q9Y4W6	AFG3-like protein 2	1.08	0.106	1.8E-02	4	5.0
P19823	Inter-alpha-trypsin inhibitor heavy chain H2	1.08	0.175	4.5E-02	3	3.4
Q92599	Septin-8	1.08	NA	NA	2	6.0
P58166	Inhibin beta E chain	1.08	NA	NA	2	6.6
Q14247	Src substrate cortactin	1.08	0.319	4.8E-02	4	8.9
P62906	60S ribosomal protein L10a	1.08	0.073	7.0E-04	7	33.6
P52565	Rho GDP-dissociation inhibitor 1	1.07	0.140	1.6E-07	4	22.5
P06703	Protein S100-A6	1.07	NA	NA	2	16.7
P62714	Serine/threonine-protein phosphatase 2A catalytic subunit beta isoform	1.07	NA	NA	2	6.1
P05107	Integrin beta-2	1.06	0.121	8.1E-02	3	4.0
P06454	Prothymosin alpha	1.06	0.365	2.8E-03	3	21.6
P49755	Transmembrane emp24 domain-containing protein 10	1.06	NA	NA	2	9.6
P61978	Heterogeneous nuclear ribonucleoprotein K	1.06	0.079	8.3E-04	13	29.6
Q8IV08	Phospholipase D3	1.06	0.076	2.0E-05	4	8.4
P13010	X-ray repair cross-complementing protein 5	1.06	0.271	5.1E-01	4	7.8
P60660	Myosin light polypeptide 6	1.06	0.056	7.9E-11	8	58.3
P36871	Phosphoglucomutase-1	1.05	0.253	1.5E-01	3	4.8
P26583	High mobility group protein B2	1.05	NA	NA	2	12.9
Q15046	Lysine--tRNA ligase	1.05	2.738	7.9E-01	3	5.5
P39059	Collagen alpha-1(XV) chain	1.05	0.100	3.1E-06	5	3.5
Q9Y3Z3	Deoxynucleoside triphosphate triphosphohydrolase SAMHD1	1.04	0.053	9.5E-03	3	5.0
Q9BS26	Endoplasmic reticulum resident protein 44	1.04	0.062	1.7E-03	4	10.1
P20810	Calpastatin	1.04	0.387	2.4E-01	3	8.2
P09543	2',3'-cyclic-nucleotide 3'-phosphodiesterase	1.04	0.136	1.2E-02	6	11.9
P09669	Cytochrome c oxidase subunit 6C	1.04	0.243	4.7E-01	3	32.0
P00738	Haptoglobin	1.03	0.085	5.0E-04	5	12.3
P12111	Collagen alpha-3(VI) chain	1.03	0.042	1.8E-11	36	11.7
Q15233	Non-POU domain-containing octamer-binding protein	1.03	NA	NA	2	4.9
Q13557	Calcium/calmodulin-dependent protein kinase type II subunit delta	1.03	NA	NA	2	4.6
P62829	60S ribosomal protein L23	1.03	NA	NA	2	12.9
P62277	40S ribosomal protein S13	1.03	0.262	7.5E-02	6	34.4
Q99714	3-hydroxyacyl-CoA dehydrogenase type-2	1.02	NA	NA	2	8.4
P02511	Alpha-crystallin B chain	1.02	0.108	5.0E-05	5	29.1
Q15691	Microtubule-associated protein RP/EB family member 1	1.02	0.614	6.4E-01	3	7.8
O00159	Unconventional myosin-Ic	1.02	0.087	1.2E-02	6	5.4
P60174	Triosephosphate isomerase	1.02	0.107	2.5E-04	12	51.4
Q06787	Fragile X mental retardation protein 1	1.02	NA	NA	2	2.7
O75964	ATP synthase subunit g, mitochondrial	1.01	NA	NA	2	27.2
P30084	Enoyl-CoA hydratase, mitochondrial	1.01	NA	NA	2	8.6
Q14699	Raftlin	1.01	0.325	2.6E-02	3	7.3
Q9H4M9	EH domain-containing protein 1	1.00	NA	NA	2	3.6
P08758	Annexin A5	1.00	0.048	0.0E+00	19	59.7
O60763	General vesicular transport factor p115	1.00	0.200	3.1E-01	3	2.6
Q5JTV8	Torsin-1A-interacting protein 1	1.00	NA	NA	2	4.3
P19367	Hexokinase-1	1.00	0.186	6.0E-02	5	5.5
Q08722	Leukocyte surface antigen CD47	1.00	NA	NA	2	5.9
P05164	Myeloperoxidase	0.99	NA	NA	2	3.0
P24534	Elongation factor 1-beta	0.99	0.106	3.0E-03	4	12.9
P61106	Ras-related protein Rab-14	0.99	0.143	5.8E-01	3	23.7
P49257	Protein ERGIC-53	0.99	0.106	9.0E-02	3	7.8
Q02978	Mitochondrial 2-oxoglutarate/malate carrier protein	0.99	NA	NA	2	6.7
Q9NTJ5	Phosphatidylinositide phosphatase SAC1	0.99	NA	NA	2	4.1
Q8IZP0	Abl interactor 1	0.98	NA	NA	2	4.9
P09012	U1 small nuclear ribonucleoprotein A	0.98	NA	NA	2	7.4
Q9NYF8	Bcl-2-associated transcription factor 1	0.98	NA	NA	2	4.2
Q71UM5	40S ribosomal protein S27-like	0.98	NA	NA	2	22.6
P55268	Laminin subunit beta-2	0.98	0.077	4.8E-10	13	8.2
P50213	Isocitrate dehydrogenase [NAD] subunit alpha, mitochondrial	0.98	0.111	1.1E-01	3	10.1
P20674	Cytochrome c oxidase subunit 5A, mitochondrial	0.98	0.050	1.0E-01	3	14.7
O75436	Vacuolar protein sorting-associated protein 26A	0.97	NA	NA	2	5.5
P29692	Elongation factor 1-delta	0.97	NA	NA	2	6.4
P23284	Peptidyl-prolyl cis-trans isomerase B	0.97	0.070	8.6E-08	10	38.9
P46939	Utrophin	0.97	0.101	4.5E-02	5	1.8
P54920	Alpha-soluble NSF attachment protein	0.97	0.170	1.8E-01	3	10.8
O75306	NADH dehydrogenase [ubiquinone] iron-sulfur protein 2, mitochondrial	0.96	0.285	4.8E-01	3	6.7
P36543	V-type proton ATPase subunit E 1	0.96	0.133	1.8E-02	3	10.6
Q15843	NEDD8	0.96	NA	NA	2	17.3
O60749	Sorting nexin-2	0.96	NA	NA	2	5.0
Q13162	Peroxiredoxin-4	0.96	0.203	3.0E-01	3	12.9
P50395	Rab GDP dissociation inhibitor beta	0.96	0.107	3.3E-06	6	16.6
Q27J81	Inverted formin-2	0.96	NA	NA	2	1.6
P29992	Guanine nucleotide-binding protein subunit alpha-11	0.95	0.265	2.0E-01	3	8.6
P43121	Cell surface glycoprotein MUC18	0.95	0.246	3.9E-02	3	5.7
P17858	ATP-dependent 6-phosphofructokinase, liver type	0.95	0.356	5.6E-01	4	5.3
P23246	Splicing factor, proline- and glutamine-rich	0.94	0.209	3.7E-02	4	6.2
P13804	Electron transfer flavoprotein subunit alpha, mitochondrial	0.94	NA	NA	2	6.0
O95202	LETM1 and EF-hand domain-containing protein 1, mitochondrial	0.94	0.106	5.2E-01	3	4.3
P04114	Apolipoprotein B-100	0.93	NA	NA	2	0.4
O14773	Tripeptidyl-peptidase 1	0.93	0.057	3.0E-04	3	6.2
P55209	Nucleosome assembly protein 1-like 1	0.93	NA	NA	2	7.7
P40939	Trifunctional enzyme subunit alpha, mitochondrial	0.93	0.114	2.1E-01	5	6.6
Q13243	Serine/arginine-rich splicing factor 5	0.92	NA	NA	2	5.9
Q92688	Acidic leucine-rich nuclear phosphoprotein 32 family member B	0.92	0.169	8.6E-03	5	17.1
P04632	Calpain small subunit 1	0.92	0.516	9.2E-01	3	11.2
Q9UHD8	Septin-9	0.92	0.374	7.0E-01	3	5.6
P07858	Cathepsin B	0.92	0.186	5.0E-04	4	13.0
P21796	Voltage-dependent anion-selective channel protein 1	0.92	0.071	3.3E-06	6	19.8
P13639	Elongation factor 2	0.91	0.161	9.3E-05	12	12.8
Q8IVF2	Protein AHNAK2	0.91	0.117	1.0E-05	8	0.5
P15088	Mast cell carboxypeptidase A	0.91	0.223	7.6E-04	5	10.1
Q14108	Lysosome membrane protein 2	0.90	NA	NA	2	4.6
P10599	Thioredoxin	0.90	0.126	1.6E-04	3	32.4
P01834	Ig kappa chain C region	0.90	NA	NA	2	34.9
P61586	Transforming protein RhoA	0.89	NA	NA	2	9.8
Q8NC56	LEM domain-containing protein 2	0.89	NA	NA	2	3.6

Q01085	Nucleolysin TIAR	0.89	NA	NA	2	4.8
P31153	S-adenosylmethionine synthase isoform type-2	0.89	0.841	7.6E-01	3	9.4
P27816	Microtubule-associated protein 4	0.89	0.292	6.6E-01	5	4.9
P02671	Fibrinogen alpha chain	0.88	0.093	3.2E-03	8	11.3
Q03252	Lamin-B2	0.88	0.057	4.8E-09	14	24.3
P54727	UV excision repair protein RAD23 homolog B	0.88	0.302	4.2E-01	5	10.0
P05091	Aldehyde dehydrogenase, mitochondrial	0.88	0.255	2.3E-01	3	6.0
Q9NZN4	EH domain-containing protein 2	0.88	NA	NA	2	3.5
P30042	ES1 protein homolog, mitochondrial	0.88	0.040	3.6E-03	3	13.1
Q9Y6N5	Sulfide:quinone oxidoreductase, mitochondrial	0.88	0.116	4.1E-02	5	14.2
P42224	Signal transducer and activator of transcription 1-alpha/beta	0.88	NA	NA	2	2.8
P08571	Monocyte differentiation antigen CD14	0.88	NA	NA	2	5.3
P30043	Flavin reductase (NADPH)	0.87	NA	NA	2	16.0
O00410	Importin-5	0.87	NA	NA	2	1.3
Q9UBS4	DnaJ homolog subfamily B member 11	0.87	NA	NA	2	5.9
P35625	Metalloproteinase inhibitor 3	0.87	0.091	1.3E-08	4	15.6
P05186	Alkaline phosphatase, tissue-nonspecific isozyme	0.87	0.219	1.5E-02	4	7.1
P09493	Tropomyosin alpha-1 chain	0.87	0.337	4.3E-02	5	12.0
P21589	5'-nucleotidase	0.87	0.159	3.4E-01	6	12.0
P51572	B-cell receptor-associated protein 31	0.86	0.111	4.9E-02	8	28.9
P26447	Protein S100-A4	0.86	0.132	5.1E-05	3	27.7
P68371	Tubulin beta-4B chain	0.86	0.065	2.9E-05	3	10.6
O00231	26S proteasome non-ATPase regulatory subunit 11	0.86	0.104	2.8E-01	4	8.5
P62136	Serine/threonine-protein phosphatase PP1-alpha catalytic subunit	0.86	NA	NA	2	6.1
Q96KP4	Cytosolic non-specific dipeptidase	0.85	0.082	4.2E-04	5	13.9
P14927	Cytochrome b-c1 complex subunit 7	0.85	1.891	8.4E-01	3	19.8
Q9Y3I0	tRNA-splicing ligase RtcB homolog	0.84	NA	NA	2	4.6
P55795	Heterogeneous nuclear ribonucleoprotein H2	0.84	0.678	9.8E-01	5	10.2
Q9BUF5	Tubulin beta-6 chain	0.84	0.113	2.1E-05	4	9.0
P02649	Apolipoprotein E	0.83	0.118	1.1E-05	16	49.5
P50991	T-complex protein 1 subunit delta	0.83	0.167	3.3E-01	5	12.4
Q9NQC3	Reticulon-4	0.83	NA	NA	2	2.3
P11166	Solute carrier family 2, facilitated glucose transporter member 1	0.83	0.132	5.6E-04	3	5.5
Q9UIJ7	GTP:AMP phosphotransferase AK3, mitochondrial	0.82	0.294	9.0E-01	3	15.0
P22748	Carbonic anhydrase 4	0.82	0.257	4.1E-04	6	16.3
P32119	Peroxiredoxin-2	0.82	NA	NA	2	8.6
Q14376	UDP-glucose 4-epimerase	0.82	NA	NA	2	6.9
P52566	Rho GDP-dissociation inhibitor 2	0.81	NA	NA	2	15.4
P17540	Creatine kinase S-type, mitochondrial	0.81	NA	NA	2	5.5
Q6NZI2	Polymerase I and transcript release factor	0.81	0.102	3.5E-04	6	19.2
P59998	Actin-related protein 2/3 complex subunit 4	0.80	0.055	6.8E-03	3	16.1
O15144	Actin-related protein 2/3 complex subunit 2	0.80	0.295	3.5E-01	4	12.0
Q93050	V-type proton ATPase 116 kDa subunit a isoform 1	0.80	NA	NA	2	3.0
Q04760	Lactoylglutathione lyase	0.80	NA	NA	2	9.8
P30519	Heme oxygenase 2	0.80	NA	NA	2	10.1
O60313	Dynamin-like 120 kDa protein, mitochondrial	0.80	NA	NA	2	2.6
Q02952	A-kinase anchor protein 12	0.80	0.151	3.5E-04	9	5.9
Q9NYL4	Peptidyl-prolyl cis-trans isomerase FKBP11	0.80	NA	NA	2	10.4
P38117	Electron transfer flavoprotein subunit beta	0.80	0.006	5.7E-02	3	11.8
Q96C86	m7GpppX diphosphatase	0.80	NA	NA	2	8.3
Q9NZ08	Endoplasmic reticulum aminopeptidase 1	0.79	NA	NA	2	2.2
Q9H2U2	Inorganic pyrophosphatase 2, mitochondrial	0.79	0.257	4.0E-01	3	9.0
P51665	26S proteasome non-ATPase regulatory subunit 7	0.79	NA	NA	2	6.2
P23528	Cofilin-1	0.79	0.169	1.6E-03	7	42.2
P25788	Proteasome subunit alpha type-3	0.79	NA	NA	2	4.7
P55735	Protein SEC13 homolog	0.79	NA	NA	2	6.8
P55786	Puromycin-sensitive aminopeptidase	0.79	0.225	6.6E-02	3	3.5
Q16658	Fascin	0.78	NA	NA	2	6.1
P30153	Serine/threonine-protein phosphatase 2A 65 kDa regulatory subunit A alpha isoform	0.78	0.048	1.5E-01	3	5.4
O00264	Membrane-associated progesterone receptor component 1	0.78	0.070	4.4E-02	3	15.9
P01023	Alpha-2-macroglobulin	0.78	0.074	1.1E-03	10	7.6
Q15029	116 kDa U5 small nuclear ribonucleoprotein component	0.78	0.168	1.7E-01	3	3.7
O94979	Protein transport protein Sec31A	0.77	0.150	2.7E-01	5	4.9
Q13642	Four and a half LIM domains protein 1	0.77	NA	NA	2	8.0
Q8WWM9	Cytoglobin	0.77	NA	NA	2	8.4
P48444	Coatomer subunit delta	0.77	NA	NA	2	4.1
P30041	Peroxiredoxin-6	0.77	0.059	5.7E-06	8	28.6
Q92499	ATP-dependent RNA helicase DDX1	0.77	NA	NA	2	2.6
P08559	Pyruvate dehydrogenase E1 component subunit alpha, somatic form, mitochondrial	0.77	NA	NA	2	5.4
P14209	CD99 antigen	0.77	NA	NA	2	9.7
Q14103	Heterogeneous nuclear ribonucleoprotein D0	0.76	NA	NA	2	6.8
O14495	Lipid phosphate phosphohydrolase 3	0.76	NA	NA	2	7.1
P02679	Fibrinogen gamma chain	0.75	0.093	7.9E-05	10	25.2
P21926	CD9 antigen	0.75	NA	NA	2	7.5
P15586	N-acetylglucosamine-6-sulfatase	0.75	0.196	4.0E-03	3	4.7
Q92820	Gamma-glutamyl hydrolase	0.74	NA	NA	2	7.9
P42167	Lamina-associated polypeptide 2, isoforms beta/gamma	0.74	NA	NA	2	5.5
P35222	Catenin beta-1	0.74	0.109	1.2E-02	6	8.6
P63220	40S ribosomal protein S21	0.74	NA	NA	2	22.9
P12955	Xaa-Pro dipeptidase	0.73	0.102	2.6E-04	4	7.3
P08133	Annexin A6	0.73	0.040	0.0E+00	28	43.2
P09525	Annexin A4	0.73	0.149	1.9E-03	8	22.9
Q14764	Major vault protein	0.73	0.128	3.4E-02	3	3.7
O15173	Membrane-associated progesterone receptor component 2	0.72	0.166	2.2E-02	3	17.9
P01008	Antithrombin-III	0.72	0.251	3.1E-03	3	6.9
O43242	26S proteasome non-ATPase regulatory subunit 3	0.71	NA	NA	2	3.2
Q9Y6M9	NADH dehydrogenase [ubiquinone] 1 beta subcomplex subunit 9	0.71	NA	NA	2	15.6
O94874	E3 UFM1-protein ligase 1	0.71	NA	NA	2	2.6
Q9BR76	Coronin-1B	0.71	NA	NA	2	3.9
P63244	Guanine nucleotide-binding protein subunit beta-2-like 1	0.71	0.363	9.7E-01	3	9.5
P04216	Thy-1 membrane glycoprotein	0.71	NA	NA	2	15.5
P46776	60S ribosomal protein L27a	0.71	NA	NA	2	14.2
P14550	Alcohol dehydrogenase [NADP(+)]	0.71	0.243	1.9E-01	4	11.4
O94826	Mitochondrial import receptor subunit TOM70	0.70	NA	NA	2	2.6
P10606	Cytochrome c oxidase subunit 5B, mitochondrial	0.70	0.131	4.8E-02	6	31.0
Q9UBR2	Cathepsin Z	0.69	NA	NA	2	7.3
P35749	Myosin-11	0.69	0.137	1.6E-09	22	11.7
O75083	WD repeat-containing protein 1	0.68	0.136	8.3E-05	6	9.7
P31146	Coronin-1A	0.68	0.218	1.3E-01	3	8.0
P30622	CAP-Gly domain-containing linker protein 1	0.68	0.722	7.9E-01	3	2.7
Q9NP81	Serine--tRNA ligase, mitochondrial	0.68	NA	NA	2	6.9
P40121	Macrophage-capping protein	0.68	NA	NA	2	5.7
O94905	Erlin-2	0.68	NA	NA	2	5.0
O95336	6-phosphogluconolactonase	0.67	0.206	3.0E-02	3	15.1
P07197	Neurofilament medium polypeptide	0.67	0.165	9.0E-03	6	6.9
Q92973	Transportin-1	0.66	0.444	2.5E-01	3	4.6
Q13510	Acid ceramidase	0.66	0.085	2.3E-11	6	12.4
Q13228	Selenium-binding protein 1	0.66	NA	NA	2	4.2
P53621	Coatomer subunit alpha	0.66	1.141	8.4E-01	6	5.1

Q9NTZ6	RNA-binding protein 12	0.65	NA	NA	2	3.3
O43390	Heterogeneous nuclear ribonucleoprotein R	0.65	0.740	5.0E-01	4	6.5
Q9P0K7	Ankycorbin	0.65	NA	NA	2	1.8
P35579	Myosin-9	0.65	0.042	4.7E-14	51	25.8
Q9Y3U8	60S ribosomal protein L36	0.64	0.084	2.5E-02	4	30.5
P00367	Glutamate dehydrogenase 1, mitochondrial	0.64	0.117	2.2E-02	6	10.8
P18124	60S ribosomal protein L7	0.64	0.117	2.5E-01	5	17.3
P06737	Glycogen phosphorylase, liver form	0.64	0.099	5.0E-05	20	23.7
P50995	Annexin A11	0.64	0.082	8.1E-04	6	11.1
P01876	Ig alpha-1 chain C region	0.64	NA	NA	2	4.5
Q02218	2-oxoglutarate dehydrogenase, mitochondrial	0.63	0.093	1.3E-01	6	6.7
P0C0S5	Histone H2A.Z	0.63	NA	NA	2	18.8
Q16795	NADH dehydrogenase [ubiquinone] 1 alpha subcomplex subunit 9, mitochondrial	0.63	NA	NA	2	6.4
P61088	Ubiquitin-conjugating enzyme E2 N	0.62	NA	NA	2	13.8
P55084	Trifunctional enzyme subunit beta, mitochondrial	0.62	0.077	1.0E-02	8	12.9
Q13838	Spliceosome RNA helicase DDX39B	0.62	NA	NA	2	5.1
P62280	40S ribosomal protein S11	0.61	0.152	1.4E-01	3	14.6
Q9NVA2	Septin-11	0.60	NA	NA	2	4.9
P02760	Protein AMBP	0.60	NA	NA	2	7.7
Q04917	14-3-3 protein eta	0.60	NA	NA	2	9.8
P78347	General transcription factor II-I	0.59	0.349	2.1E-01	3	2.9
P02751	Fibronectin	0.59	0.094	3.4E-07	8	4.4
P02545	Prelamin-A/C	0.59	0.644	5.4E-01	34	41.1
P29401	Transketolase	0.58	0.131	7.2E-05	9	12.2
Q6P2Q9	Pre-mRNA-processing-splicing factor 8	0.58	0.648	7.9E-01	3	1.4
Q96AE4	Far upstream element-binding protein 1	0.58	0.257	4.6E-01	3	4.5
P28331	NADH-ubiquinone oxidoreductase 75 kDa subunit, mitochondrial	0.58	0.766	8.5E-01	3	4.5
Q5SSJ5	Heterochromatin protein 1-binding protein 3	0.57	NA	NA	2	3.4
P00505	Aspartate aminotransferase, mitochondrial	0.57	0.086	6.2E-03	6	16.7
Q92522	Histone H1x	0.57	NA	NA	2	11.7
Q14683	Structural maintenance of chromosomes protein 1A	0.56	0.077	6.5E-02	3	2.8
Q5VTE0	Putative elongation factor 1-alpha-like 3	0.56	0.074	9.4E-05	11	26.6
P08670	Vimentin	0.56	0.039	0.0E+00	28	59.4
P40926	Malate dehydrogenase, mitochondrial	0.56	0.107	2.0E-07	8	28.7
P05023	Sodium/potassium-transporting ATPase subunit alpha-1	0.55	0.096	6.4E-06	15	16.2
Q9NSD9	Phenylalanine--tRNA ligase beta subunit	0.55	0.464	5.8E-01	3	4.9
Q53EL6	Programmed cell death protein 4	0.53	NA	NA	2	4.7
P20618	Proteasome subunit beta type-1	0.53	0.174	5.2E-03	3	15.4
P02787	Serotransferrin	0.53	0.099	9.2E-05	14	21.3
P02768	Serum albumin	0.52	0.034	0.0E+00	37	54.0
P05387	60S acidic ribosomal protein P2	0.52	0.087	1.9E-03	5	32.2
P15880	40S ribosomal protein S2	0.52	NA	NA	2	7.8
P06753	Tropomyosin alpha-3 chain	0.52	0.102	1.3E-04	4	13.0
P57053	Histone H2B type F-S	0.51	NA	NA	2	7.9
Q8WUM4	Programmed cell death 6-interacting protein	0.50	0.246	1.2E-01	4	4.5
Q5JRX3	Presequence protease, mitochondrial	0.50	NA	NA	2	2.4
P14136	Glial fibrillary acidic protein	0.50	NA	NA	2	5.6
O75947	ATP synthase subunit d, mitochondrial	0.49	0.324	3.1E-01	5	22.4
P07437	Tubulin beta chain	0.49	0.058	1.5E-06	3	9.9
P23396	40S ribosomal protein S3	0.49	0.099	5.0E-02	7	24.7
O94919	Endonuclease domain-containing 1 protein	0.48	NA	NA	2	5.8
Q9H3G5	Probable serine carboxypeptidase CPVL	0.47	NA	NA	2	3.2
P17096	High mobility group protein HMG-I/HMG-Y	0.47	NA	NA	2	23.4
P40227	T-complex protein 1 subunit zeta	0.47	NA	NA	2	4.1
O15143	Actin-related protein 2/3 complex subunit 1B	0.47	NA	NA	2	6.5
P48735	Isocitrate dehydrogenase [NADP], mitochondrial	0.46	NA	NA	2	4.2
P46777	60S ribosomal protein L5	0.46	0.846	3.0E-01	4	15.5
P12814	Alpha-actinin-1	0.46	0.062	5.9E-07	8	12.3
Q53H82	Beta-lactamase-like protein 2	0.45	0.289	1.6E-01	3	12.5
P06899	Histone H2B type 1-J	0.45	NA	NA	2	7.9
P62701	40S ribosomal protein S4, X isoform	0.45	0.366	9.3E-01	3	9.1
P60981	Destrin	0.45	NA	NA	2	13.3
P38646	Stress-70 protein, mitochondrial	0.45	0.137	2.4E-02	8	13.5
Q13561	Dynactin subunit 2	0.44	0.109	1.7E-01	5	10.7
P49207	60S ribosomal protein L34	0.43	NA	NA	2	12.8
P23634	Plasma membrane calcium-transporting ATPase 4	0.43	NA	NA	2	2.0
O43707	Alpha-actinin-4	0.43	0.080	3.4E-03	15	19.8
A4D1P6	WD repeat-containing protein 91	0.42	NA	NA	2	4.3
Q15942	Zyxin	0.41	NA	NA	2	5.2
Q99873	Protein arginine N-methyltransferase 1	0.41	NA	NA	2	7.5
P11940	Polyadenylate-binding protein 1	0.38	0.073	6.2E-02	4	7.4
Q15631	Translin	0.38	NA	NA	2	8.8
P43307	Translocon-associated protein subunit alpha	0.37	NA	NA	2	6.6
P34932	Heat shock 70 kDa protein 4	0.36	0.309	3.7E-01	8	11.3
P55060	Exportin-2	0.35	0.205	1.0E-01	3	3.4
O95865	N(G),N(G)-dimethylarginine dimethylaminohydrolase 2	0.35	0.204	3.1E-01	3	11.2
Q08380	Galectin-3-binding protein	0.34	NA	NA	2	4.8
P18859	ATP synthase-coupling factor 6, mitochondrial	0.33	0.965	7.2E-01	3	30.6
Q14974	Importin subunit beta-1	0.33	0.216	9.7E-02	3	5.4
P07384	Calpain-1 catalytic subunit	0.31	0.085	3.0E-01	5	7.7
Q14203	Dynactin subunit 1	0.31	0.114	1.4E-01	3	3.3
Q14558	Phosphoribosyl pyrophosphate synthase-associated protein 1	0.31	NA	NA	2	8.4
P35637	RNA-binding protein FUS	0.30	0.521	1.0E+00	3	5.3
O75477	Erlin-1	0.29	NA	NA	2	7.2
Q9ULA0	Aspartyl aminopeptidase	0.29	NA	NA	2	6.1
Q15121	Astrocytic phosphoprotein PEA-15	0.28	NA	NA	2	16.9
P26196	Probable ATP-dependent RNA helicase DDX6	0.28	NA	NA	2	6.8
O14979	Heterogeneous nuclear ribonucleoprotein D-like	0.27	0.214	1.4E-01	3	4.5
P47985	Cytochrome b-c1 complex subunit Rieske, mitochondrial	0.26	NA	NA	2	8.0
P10809	60 kDa heat shock protein, mitochondrial	0.26	0.269	8.0E-02	12	20.9
P17661	Desmin	0.26	NA	NA	2	4.0
Q6DD88	Atlastin-3	0.26	0.129	5.9E-02	3	5.9
P31949	Protein S100-A11	0.23	NA	NA	2	17.1
Q99733	Nucleosome assembly protein 1-like 4	0.23	NA	NA	2	7.7
Q99623	Prohibitin-2	0.21	0.126	1.6E-01	8	28.1
P14317	Hematopoietic lineage cell-specific protein	0.21	NA	NA	2	4.3
P17931	Galectin-3	0.21	0.402	4.0E-01	5	25.6
P32969	60S ribosomal protein L9	0.20	0.403	9.4E-01	4	15.1
P04440	HLA class II histocompatibility antigen, DP beta 1 chain	0.20	NA	NA	2	7.8
O95302	Peptidyl-prolyl cis-trans isomerase FKBP9	0.20	NA	NA	2	4.0
P52815	39S ribosomal protein L12, mitochondrial	0.20	NA	NA	2	5.6
Q9BQE3	Tubulin alpha-1C chain	0.18	NA	NA	2	6.7
O43809	Cleavage and polyadenylation specificity factor subunit 5	0.18	NA	NA	2	13.2
P35221	Catenin alpha-1	0.17	0.173	2.5E-01	6	7.4
Q8NBS9	Thioredoxin domain-containing protein 5	0.17	0.088	6.5E-02	7	15.5
P17612	cAMP-dependent protein kinase catalytic subunit alpha	0.16	NA	NA	2	4.3
Q00325	Phosphate carrier protein, mitochondrial	0.16	0.307	5.7E-01	6	16.0
P27824	Calnexin	0.15	0.490	5.8E-01	8	15.4
P60033	CD81 antigen	0.14	NA	NA	2	11.9

P16152	Carbonyl reductase [NADPH] 1	0.14	NA	NA	2	7.6
P62249	40S ribosomal protein S16	0.14	NA	NA	2	15.8
P62913	60S ribosomal protein L11	0.14	0.103	5.6E-02	3	16.9
P28838	Cytosol aminopeptidase	0.14	0.217	4.1E-01	7	17.0
Q00796	Sorbitol dehydrogenase	0.14	NA	NA	2	4.8
A0FGR8	Extended synaptotagmin-2	0.13	NA	NA	2	2.8
P39023	60S ribosomal protein L3	0.12	0.332	6.3E-01	3	6.7
Q16853	Membrane primary amine oxidase	0.12	NA	NA	2	2.4
P62269	40S ribosomal protein S18	0.12	0.202	5.5E-01	6	38.8
P30044	Peroxiredoxin-5, mitochondrial	0.11	0.177	2.0E-01	3	16.8
O00203	AP-3 complex subunit beta-1	0.11	NA	NA	2	2.7
Q13151	Heterogeneous nuclear ribonucleoprotein A0	0.10	3.349	7.5E-01	3	7.5
O14950	Myosin regulatory light chain 12B	0.10	NA	NA	2	12.2
P17980	26S protease regulatory subunit 6A	0.10	NA	NA	2	5.7
Q07065	Cytoskeleton-associated protein 4	0.08	0.245	2.0E-01	6	12.5
P14625	Endoplasmin	0.07	0.134	7.7E-02	14	17.2
P60842	Eukaryotic initiation factor 4A-I	0.04	NA	NA	2	6.4
P07339	Cathepsin D	0.41	0.117	1.3E-05	5	11.7
P04083	Annexin A1	0.39	0.106	4.1E-09	11	30.6
Q13813	Spectrin alpha chain, non-erythrocytic 1	0.39	0.026	0.0E+00	60	25.9
P02730	Band 3 anion transport protein	0.37	0.144	1.2E-04	6	7.1
Q09666	Neuroblast differentiation-associated protein AHNAK	0.37	0.038	5.6E-11	74	11.8
Q99572	P2X purinoceptor 7	0.37	0.156	2.8E-02	5	10.6
P21333	Filamin-A	0.37	0.047	6.7E-16	48	22.8
O14818	Proteasome subunit alpha type-7	0.36	0.099	3.4E-04	3	11.3
Q13435	Splicing factor 3B subunit 2	0.36	0.401	4.3E-02	3	5.1
P62805	Histone H4	0.36	0.028	2.1E-14	7	52.4
Q14204	Cytoplasmic dynein 1 heavy chain 1	0.35	0.065	4.0E-05	25	5.9
P04075	Fructose-bisphosphate aldolase A	0.34	0.122	2.7E-03	13	43.1
P26038	Moesin	0.34	0.098	1.5E-03	10	17.0
P78417	Glutathione S-transferase omega-1	0.33	0.124	2.4E-10	9	31.1
P07195	L-lactate dehydrogenase B chain	0.30	0.113	3.3E-04	8	26.3
P22352	Glutathione peroxidase 3	0.29	0.068	2.1E-12	3	11.5
P06865	Beta-hexosaminidase subunit alpha	0.29	0.123	5.0E-06	10	18.7
Q15149	Plectin	0.28	0.030	4.9E-14	51	12.0
P13797	Plastin-3	0.28	0.089	3.9E-04	6	9.8
O60506	Heterogeneous nuclear ribonucleoprotein Q	0.28	0.155	2.7E-02	3	5.6
P11277	Spectrin beta chain, erythrocytic	0.28	0.235	2.2E-02	5	3.3
Q9Y490	Talin-1	0.27	0.052	6.8E-10	31	14.8
P04792	Heat shock protein beta-1	0.26	0.056	9.5E-06	6	27.8
Q14112	Nidogen-2	0.26	0.147	4.6E-07	9	7.3
P26641	Elongation factor 1-gamma	0.25	0.153	9.1E-03	5	10.5
P49591	Serine--tRNA ligase, cytoplasmic	0.25	0.073	2.5E-03	3	9.9
P60709	Actin, cytoplasmic 1	0.25	0.051	1.3E-03	6	24.8
P99999	Cytochrome c	0.24	0.115	2.3E-04	3	24.8
P21266	Glutathione S-transferase Mu 3	0.24	0.137	1.8E-03	3	12.4
A1L0T0	Acetolactate synthase-like protein	0.23	0.044	8.0E-03	4	6.0
Q15063	Periostin	0.23	0.161	1.9E-02	5	7.4
P15531	Nucleoside diphosphate kinase A	0.23	0.108	4.0E-10	7	52.6
P00558	Phosphoglycerate kinase 1	0.22	0.104	1.8E-07	13	29.7
P12109	Collagen alpha-1(VI) chain	0.21	0.075	5.0E-06	11	11.5
P08195	4F2 cell-surface antigen heavy chain	0.20	0.104	2.1E-03	3	5.9
P13760	HLA class II histocompatibility antigen, DRB1-4 beta chain	0.19	0.142	1.8E-02	5	20.7
P02647	Apolipoprotein A-I	0.18	0.138	6.7E-03	7	24.3
P49368	T-complex protein 1 subunit gamma	0.17	0.056	2.0E-02	8	19.1
Q99497	Protein DJ-1	0.16	0.221	1.3E-03	6	28.0
P62258	14-3-3 protein epsilon	0.16	0.116	3.7E-04	9	34.9
P12110	Collagen alpha-2(VI) chain	0.16	0.095	2.8E-04	8	7.9
P07237	Protein disulfide-isomerase	0.16	0.111	2.6E-03	11	18.5
P09874	Poly [ADP-ribose] polymerase 1	0.15	0.100	4.8E-02	5	5.6
P60903	Protein S100-A10	0.15	0.199	9.0E-03	4	41.2
P09651	Heterogeneous nuclear ribonucleoprotein A1	0.13	0.049	9.9E-07	4	13.2
P07355	Annexin A2	0.13	0.041	0.0E+00	23	54.6
P78527	DNA-dependent protein kinase catalytic subunit	0.12	0.058	2.0E-04	17	3.7
P51888	Prolargin	0.12	0.112	0.0E+00	12	32.2
P62851	40S ribosomal protein S25	0.12	0.068	3.7E-05	5	29.6
Q01082	Spectrin beta chain, non-erythrocytic 1	0.11	0.038	0.0E+00	45	23.1
P01871	Ig mu chain C region	0.11	0.116	3.8E-05	7	18.8
P49773	Histidine triad nucleotide-binding protein 1	0.10	0.373	2.2E-02	4	42.9
P62937	Peptidyl-prolyl cis-trans isomerase A	0.10	0.092	0.0E+00	7	35.2
Q15582	Transforming growth factor-beta-induced protein ig-h3	0.10	0.080	3.7E-02	3	4.7
P01011	Alpha-1-antichymotrypsin	0.10	0.102	1.4E-06	6	10.6
P11216	Glycogen phosphorylase, brain form	0.09	0.119	1.1E-02	7	10.3
P06733	Alpha-enolase	0.08	0.100	2.1E-06	12	36.4
P63104	14-3-3 protein zeta/delta	0.06	0.107	6.3E-03	6	29.8
P21810	Biglycan	0.05	0.102	5.5E-12	7	20.1
P39060	Collagen alpha-1(XVIII) chain	0.04	0.093	5.5E-08	9	5.8
P18621	60S ribosomal protein L17	0.04	0.081	2.7E-03	4	19.0

Brown denotes change ≥ 2 standard deviations (SD) from the mean, yellow denotes change ≥ 1 SD and green highlights p values ≤ 0.05. NA, not applicable, n<3 unique peptides.
