# Supplementary material for: iTRAQ Quantitative Proteomic Comparison of Metastatic and Non-Metastatic Uveal Melanoma Tumors
Source: PLoS One. 2015 Aug 25;10(8):e0135543. doi: 10.1371/journal.pone.0135543 (PMC4549237; doi:10.1371/journal.pone.0135543)
Supplement: S3 Table — (PDF) [file pone.0135543.s003.pdf]

Supplementary Table S3
Relative Protein Abundance: Sample UM24, Metastatic
Total Proteins Quantified = 723; LogMedian Protein Ratio = 0.05; LogMean Protein Ratio = 0; Standard Deviation = 0.81

Uni-Prot Accession	Protein	Ratio UM/Control	Standard Deviation	*p* value	Unique Peptides	% Sequence Coverage
P02768	Serum albumin	14.89	0.033	0.0E+00	48	64.7
P68871	Hemoglobin subunit beta	9.54	0.070	0.0E+00	6	55.8
P69905	Hemoglobin subunit alpha	8.80	0.067	0.0E+00	7	63.4
P00734	Prothrombin	8.75	0.227	5.9E-03	7	14.6
P02042	Hemoglobin subunit delta	8.17	0.108	1.6E-08	7	59.2
P02787	Serotransferrin	7.41	0.063	0.0E+00	30	42.7
P62937	Peptidyl-prolyl cis-trans isomerase A	7.29	0.082	3.1E-12	10	60.0
Q00796	Sorbitol dehydrogenase	6.14	0.214	3.4E-03	3	4.8
P02765	Alpha-2-HS-glycoprotein	5.87	0.173	7.6E-06	5	12.0
P02774	Vitamin D-binding protein	5.40	0.115	9.6E-08	15	26.8
P02652	Apolipoprotein A-II	5.28	0.115	3.1E-03	4	21.0
P59665	Neutrophil defensin 1	5.13	0.135	4.9E-05	3	20.2
P43652	Afamin	4.85	0.152	2.7E-02	4	6.7
P23528	Cofilin-1	4.81	0.205	4.7E-03	5	33.7
P01857	Ig gamma-1 chain C region	4.79	0.063	1.6E-15	4	18.8
P00558	Phosphoglycerate kinase 1	4.63	0.089	4.3E-06	11	24.9
P06702	Protein S100-A9	4.37	0.197	1.1E-03	3	30.7
P07737	Profilin-1	4.13	0.126	5.8E-06	5	39.3
Q8IV08	Phospholipase D3	3.91	0.168	2.6E-03	3	6.3
P02790	Hemopexin	3.90	0.106	2.3E-08	5	13.2
P09601	Heme oxygenase 1	3.87	0.209	4.8E-02	4	17.0
P05164	Myeloperoxidase	3.75	0.077	4.6E-14	13	17.7
P78417	Glutathione S-transferase omega-1	3.74	0.164	9.9E-04	6	24.5
P01876	Ig alpha-1 chain C region	3.73	0.130	1.9E-04	7	21.5
P08574	Cytochrome c1, heme protein, mitochondrial	3.60	0.127	8.4E-05	5	22.2
P00915	Carbonic anhydrase 1	3.59	0.257	1.7E-02	3	11.5
P02647	Apolipoprotein A-I	3.59	0.083	1.6E-10	10	39.3
Q13838	Spliceosome RNA helicase DDX39B	3.54	0.111	7.5E-03	4	9.6
P08758	Annexin A5	3.48	0.054	0.0E+00	14	46.3
P13639	Elongation factor 2	3.45	0.102	1.4E-04	8	11.5
P05109	Protein S100-A8	3.44	0.152	3.2E-05	5	39.8
P01042	Kininogen-1	3.36	0.101	1.3E-03	8	11.6
P05107	Integrin beta-2	3.26	0.136	1.0E-03	9	12.1
P21796	Voltage-dependent anion-selective channel protein 1	3.26	0.066	1.3E-06	10	42.8
P01834	Ig kappa chain C region	3.23	0.072	3.3E-04	4	65.1
P35232	Prohibitin	3.17	0.064	4.0E-11	12	48.9
P01009	Alpha-1-antitrypsin	3.13	0.075	1.7E-08	19	49.3
P84077	ADP-ribosylation factor 1	3.13	0.155	8.6E-04	3	16.0
P05155	Plasma protease C1 inhibitor	3.05	0.132	1.4E-03	5	12.0
P36955	Pigment epithelium-derived factor	3.04	0.103	1.1E-04	8	23.4
P13796	Plastin-2	3.02	0.160	2.4E-03	3	4.6
P33121	Long-chain-fatty-acid--CoA ligase 1	2.93	0.072	4.4E-03	6	9.6
P04406	Glyceraldehyde-3-phosphate dehydrogenase	2.90	0.044	6.7E-16	10	29.0
P62826	GTP-binding nuclear protein Ran	2.86	0.123	4.8E-02	3	13.9
P08238	Heat shock protein HSP 90-beta	2.79	0.103	2.2E-03	6	8.3
P07858	Cathepsin B	2.77	0.157	1.2E-03	3	10.6
P02751	Fibronectin	2.76	0.050	3.6E-15	31	19.1
P14618	Pyruvate kinase PKM	2.74	0.067	8.8E-10	15	33.1
P19823	Inter-alpha-trypsin inhibitor heavy chain H2	2.66	0.115	1.8E-02	3	3.8
P45880	Voltage-dependent anion-selective channel protein 2	2.65	0.082	3.9E-05	7	24.5
P08195	4F2 cell-surface antigen heavy chain	2.54	0.113	1.6E-04	8	16.5
Q99623	Prohibitin-2	2.54	0.073	1.7E-09	8	29.4
P07339	Cathepsin D	2.53	0.148	2.0E-05	8	21.8
P06733	Alpha-enolase	2.52	0.091	5.3E-03	11	33.2
P01023	Alpha-2-macroglobulin	2.52	0.051	6.7E-07	14	11.5
P02792	Ferritin light chain	2.50	0.184	2.8E-02	3	13.1
Q8NBJ5	Procollagen galactosyltransferase 1	2.46	0.299	4.0E-02	3	5.0
P60842	Eukaryotic initiation factor 4A-I	2.41	0.181	4.6E-02	4	11.8
O75083	WD repeat-containing protein 1	2.37	0.237	1.1E-02	3	7.6
Q15063	Periostin	2.35	0.124	1.7E-03	9	15.2
P07900	Heat shock protein HSP 90-alpha	2.31	0.080	1.4E-04	9	12.4
P00747	Plasminogen	2.30	0.088	3.1E-06	13	17.2
Q08211	ATP-dependent RNA helicase A	2.28	0.147	5.0E-03	4	3.9
P16401	Histone H1.5	2.28	0.123	2.9E-03	3	14.6
P19367	Hexokinase-1	2.25	0.098	1.4E-04	10	11.9
Q9P2R7	Succinyl-CoA ligase [ADP-forming] subunit beta, mitochondrial	7.32	NA	NA	2	3.2
P27169	Serum paraoxonase/arylesterase 1	6.83	NA	NA	2	5.4
P02763	Alpha-1-acid glycoprotein 1	5.46	NA	NA	2	11.9
P30043	Flavin reductase (NADPH)	5.31	NA	NA	2	14.1
P17213	Bactericidal permeability-increasing protein	5.01	NA	NA	2	3.5
P01859	Ig gamma-2 chain C region	4.45	NA	NA	2	7.7
P0CG05	Ig lambda-2 chain C regions	4.43	NA	NA	2	27.4
P22087	rRNA 2'-O-methyltransferase fibrillarin	3.71	0.242	8.1E-02	3	10.9
P31146	Coronin-1A	3.54	NA	NA	2	5.9
Q8NBJ7	Sulfatase-modifying factor 2	3.34	NA	NA	2	10.0
Q96C86	m7GpppX diphosphatase	3.19	NA	NA	2	7.1
P24158	Myeloblastin	3.17	NA	NA	2	7.8
P16152	Carbonyl reductase [NADPH] 1	3.09	NA	NA	2	10.5
P07093	Glia-derived nexin	3.08	NA	NA	2	6.5
Q16658	Fascin	3.07	NA	NA	2	5.3
P40967	Melanocyte protein PMEL	3.02	NA	NA	2	3.3
P11766	Alcohol dehydrogenase class-3	2.98	0.399	8.8E-02	3	7.0
P41218	Myeloid cell nuclear differentiation antigen	2.91	0.286	4.2E-01	3	7.1
P23381	Tryptophan--tRNA ligase, cytoplasmic	2.85	NA	NA	2	3.2
P30044	Peroxiredoxin-5, mitochondrial	2.76	NA	NA	2	10.3
P52566	Rho GDP-dissociation inhibitor 2	2.75	NA	NA	2	12.4
Q15029	116 kDa U5 small nuclear ribonucleoprotein component	2.71	NA	NA	2	2.0
Q92688	Acidic leucine-rich nuclear phosphoprotein 32 family member B	2.71	NA	NA	2	6.8
Q13098	COP9 signalosome complex subunit 1	2.68	NA	NA	2	5.7
P07919	Cytochrome b-c1 complex subunit 6, mitochondrial	2.65	NA	NA	2	35.2
Q9Y2X3	Nucleolar protein 58	2.64	NA	NA	2	5.3
Q13303	Voltage-gated potassium channel subunit beta-2	2.60	NA	NA	2	5.7
P04440	HLA class II histocompatibility antigen, DP beta 1 chain	2.59	NA	NA	2	7.8
P08246	Neutrophil elastase	2.59	NA	NA	2	6.4
P31948	Stress-induced-phosphoprotein 1	2.53	NA	NA	2	4.1
P01625	Ig kappa chain V-IV region Len	2.53	NA	NA	2	21.1
P23526	Adenosylhomocysteinase	2.51	NA	NA	2	6.5
Q9Y5X3	Sorting nexin-5	2.50	NA	NA	2	5.4
P62158	Calmodulin	2.49	NA	NA	2	22.1
P02786	Transferrin receptor protein 1	2.43	NA	NA	2	3.2

O43776	Asparagine--tRNA ligase, cytoplasmic	2.42	NA	NA	2	5.7
Q1KMD3	Heterogeneous nuclear ribonucleoprotein U-like protein 2	2.38	0.375	1.7E-01	3	3.1
P01620	Ig kappa chain V-III region SIE	2.37	NA	NA	2	31.2
P63241	Eukaryotic translation initiation factor 5A-1	2.36	0.298	5.4E-02	4	27.9
Q99523	Sortilin	2.36	NA	NA	2	2.6
O00299	Chloride intracellular channel protein 1	2.32	0.472	1.6E-01	4	24.9
P12429	Annexin A3	2.29	NA	NA	2	6.2
P51810	G-protein coupled receptor 143	2.27	NA	NA	2	5.7
P22234	Multifunctional protein ADE2	2.25	NA	NA	2	3.3
P09211	Glutathione S-transferase P	2.24	0.183	9.3E-02	7	48.1
O14556	Glyceraldehyde-3-phosphate dehydrogenase, testis-specific	2.23	0.075	4.0E-02	3	10.3
Q12906	Interleukin enhancer-binding factor 3	2.23	0.110	7.9E-03	6	8.9
P35613	Basigin	2.22	NA	NA	2	8.3
P20702	Integrin alpha-X	2.20	0.341	2.8E-01	3	2.8
P61626	Lysozyme C	2.19	NA	NA	2	12.8
Q7Z7H5	Transmembrane emp24 domain-containing protein 4	2.17	NA	NA	2	9.7
P06727	Apolipoprotein A-IV	2.15	0.065	2.6E-10	17	41.9
P00751	Complement factor B	2.13	0.175	1.7E-02	5	5.4
P06748	Nucleophosmin	2.12	0.076	2.4E-04	5	15.0
P62241	40S ribosomal protein S8	2.12	NA	NA	2	9.1
Q6PIU2	Neutral cholesterol ester hydrolase 1	2.10	NA	NA	2	4.7
Q00341	Vigilin	2.10	NA	NA	2	1.7
P50454	Serpin H1	2.09	0.083	3.7E-04	6	17.7
P22695	Cytochrome b-c1 complex subunit 2, mitochondrial	2.09	0.083	1.6E-05	5	15.0
P11279	Lysosome-associated membrane glycoprotein 1	2.08	NA	NA	2	4.1
P43490	Nicotinamide phosphoribosyltransferase	2.08	NA	NA	2	3.1
P51149	Ras-related protein Rab-7a	2.06	0.064	1.3E-06	6	32.9
P26599	Polypyrimidine tract-binding protein 1	2.04	0.399	2.3E-01	3	3.6
Q9UKM9	RNA-binding protein Raly	2.03	NA	NA	2	7.2
Q16610	Extracellular matrix protein 1	2.03	NA	NA	2	4.8
Q07960	Rho GTPase-activating protein 1	2.02	0.219	1.1E-01	3	5.0
P54136	Arginine--tRNA ligase, cytoplasmic	2.00	0.182	3.6E-02	4	7.4
Q9UPN3	Microtubule-actin cross-linking factor 1, isoforms 1/2/3/5	1.99	NA	NA	2	0.5
P50213	Isocitrate dehydrogenase [NAD] subunit alpha, mitochondrial	1.99	NA	NA	2	6.0
Q15907	Ras-related protein Rab-11B	1.97	0.131	4.4E-02	3	12.4
P46063	ATP-dependent DNA helicase Q1	1.96	NA	NA	2	3.5
P16070	CD44 antigen	1.96	0.279	2.3E-01	6	8.8
Q00839	Heterogeneous nuclear ribonucleoprotein U	1.96	0.152	1.1E-02	8	9.0
P11215	Integrin alpha-M	1.95	0.385	2.8E-01	3	3.0
P10809	60 kDa heat shock protein, mitochondrial	1.95	0.080	7.0E-04	7	12.6
P52272	Heterogeneous nuclear ribonucleoprotein M	1.94	0.103	7.0E-05	9	14.0
P78527	DNA-dependent protein kinase catalytic subunit	1.93	0.100	9.5E-04	12	3.3
Q13423	NAD(P) transhydrogenase, mitochondrial	1.93	0.144	1.1E-03	9	9.2
P31930	Cytochrome b-c1 complex subunit 1, mitochondrial	1.92	0.072	1.3E-03	6	17.7
Q6UW68	Transmembrane protein 205	1.92	NA	NA	2	10.6
P08603	Complement factor H	1.92	0.224	4.0E-02	5	4.2
Q9NVH1	DnaJ homolog subfamily C member 11	1.92	NA	NA	2	4.8
P60174	Triosephosphate isomerase	1.89	0.033	7.8E-04	5	20.3
O15371	Eukaryotic translation initiation factor 3 subunit D	1.89	NA	NA	2	3.5
Q14974	Importin subunit beta-1	1.86	0.053	2.4E-02	4	7.2
O00160	Unconventional myosin-If	1.86	NA	NA	2	2.1
O15118	Niemann-Pick C1 protein	1.86	NA	NA	2	1.2
Q08945	FACT complex subunit SSRP1	1.85	NA	NA	2	2.0
P46940	Ras GTPase-activating-like protein IQGAP1	1.84	0.136	1.3E-03	10	6.3
P26641	Elongation factor 1-gamma	1.84	0.128	3.4E-02	6	14.4
Q08380	Galectin-3-binding protein	1.83	0.148	8.1E-03	5	11.6
P22314	Ubiquitin-like modifier-activating enzyme 1	1.82	0.222	7.4E-02	4	3.9
P01860	Ig gamma-3 chain C region	1.79	NA	NA	2	3.2
O43390	Heterogeneous nuclear ribonucleoprotein R	1.78	0.107	3.2E-02	5	6.5
P63104	14-3-3 protein zeta/delta	1.78	0.114	4.8E-02	4	22.9
Q16698	2,4-dienoyl-CoA reductase, mitochondrial	1.75	0.274	1.9E-01	3	11.3
P61421	V-type proton ATPase subunit d 1	1.75	0.102	2.1E-03	5	12.8
P49755	Transmembrane emp24 domain-containing protein 10	1.75	0.363	1.3E-01	4	22.4
P62847	40S ribosomal protein S24	1.75	NA	NA	2	19.5
P07814	Bifunctional glutamate/proline--tRNA ligase	1.75	NA	NA	2	1.9
P02654	Apolipoprotein C-I	1.73	0.081	4.0E-04	4	34.9
P19338	Nucleolin	1.73	0.097	7.7E-03	7	10.0
P04040	Catalase	1.72	0.112	6.9E-02	5	11.0
Q8IUX7	Adipocyte enhancer-binding protein 1	1.71	0.146	1.7E-02	5	6.4
P29401	Transketolase	1.70	0.232	3.0E-01	5	6.3
O15144	Actin-related protein 2/3 complex subunit 2	1.70	0.040	6.5E-05	4	12.7
Q5VTE0	Putative elongation factor 1-alpha-like 3	1.70	0.051	3.4E-06	12	27.9
P04114	Apolipoprotein B-100	1.70	0.276	1.0E-01	3	0.6
Q9NX63	Coiled-coil-helix-coiled-coil-helix domain-containing protein 3, mitochondrial	1.67	NA	NA	2	9.3
P06576	ATP synthase subunit beta, mitochondrial	1.67	0.064	1.1E-07	15	35.0
Q9UJZ1	Stomatin-like protein 2, mitochondrial	1.66	NA	NA	2	10.4
P04080	Cystatin-B	1.66	0.065	5.2E-03	3	39.8
P59998	Actin-related protein 2/3 complex subunit 4	1.65	1.427	4.1E-01	3	16.1
P07910	Heterogeneous nuclear ribonucleoproteins C1/C2	1.65	0.094	2.4E-03	9	26.1
P08237	ATP-dependent 6-phosphofructokinase, muscle type	1.65	0.218	1.4E-01	4	6.8
P21912	Succinate dehydrogenase [ubiquinone] iron-sulfur subunit, mitochondrial	1.65	0.088	8.0E-03	3	11.4
P02675	Fibrinogen beta chain	1.64	0.057	4.0E-04	14	31.6
Q15181	Inorganic pyrophosphatase	1.64	NA	NA	2	6.6
Q9Y4W6	AFG3-like protein 2	1.63	0.143	4.5E-01	3	3.5
P23284	Peptidyl-prolyl cis-trans isomerase B	1.63	0.061	6.1E-05	11	43.5
P02746	Complement C1q subcomponent subunit B	1.62	NA	NA	2	8.7
O75390	Citrate synthase, mitochondrial	1.62	0.079	2.6E-03	6	13.7
P02671	Fibrinogen alpha chain	1.61	0.066	5.2E-04	11	14.8
P04217	Alpha-1B-glycoprotein	1.61	0.242	2.3E-01	3	6.3
P31040	Succinate dehydrogenase [ubiquinone] flavoprotein subunit, mitochondrial	1.61	0.162	1.4E-02	5	10.2
P11142	Heat shock cognate 71 kDa protein	1.61	0.074	6.6E-05	10	12.1
P02452	Collagen alpha-1(I) chain	1.60	12.316	5.7E-01	3	2.7
Q99497	Protein DJ-1	1.59	0.276	3.4E-01	3	20.6
O95302	Peptidyl-prolyl cis-trans isomerase FKBP9	1.58	NA	NA	2	3.7
P09651	Heterogeneous nuclear ribonucleoprotein A1	1.57	0.089	2.8E-04	10	33.1
P01034	Cystatin-C	1.57	0.084	1.3E-02	3	30.8
P51572	B-cell receptor-associated protein 31	1.57	0.105	1.3E-02	6	25.6
P04196	Histidine-rich glycoprotein	1.56	0.105	4.5E-02	6	9.3
P15531	Nucleoside diphosphate kinase A	1.55	0.054	1.8E-03	4	31.6
P09525	Annexin A4	1.55	0.083	6.4E-04	8	22.6
P05090	Apolipoprotein D	1.53	0.151	1.3E-01	3	15.9
P43243	Matrin-3	1.52	NA	NA	2	3.4
O00231	26S proteasome non-ATPase regulatory subunit 11	1.52	0.070	4.6E-02	4	9.5
P52565	Rho GDP-dissociation inhibitor 1	1.52	NA	NA	2	15.2
P63000	Ras-related C3 botulinum toxin substrate 1	1.51	0.103	1.3E-01	3	17.2
P06737	Glycogen phosphorylase, liver form	1.51	0.183	5.9E-02	6	8.1
Q12905	Interleukin enhancer-binding factor 2	1.50	0.163	3.8E-02	4	13.8

P52597	Heterogeneous nuclear ribonucleoprotein F	1.50	0.515	3.4E-01	3	7.0
P14868	Aspartate--tRNA ligase, cytoplasmic	1.49	0.164	4.9E-02	5	13.4
Q13162	Peroxiredoxin-4	1.49	NA	NA	2	8.9
O60506	Heterogeneous nuclear ribonucleoprotein Q	1.49	NA	NA	2	2.9
P05388	60S acidic ribosomal protein P0	1.49	0.104	2.9E-02	4	13.6
P13010	X-ray repair cross-complementing protein 5	1.48	0.258	7.0E-02	6	8.1
Q9Y6C9	Mitochondrial carrier homolog 2	1.48	0.096	5.8E-02	3	16.5
P38606	V-type proton ATPase catalytic subunit A	1.48	0.111	5.5E-02	6	9.2
P30484	HLA class I histocompatibility antigen, B-46 alpha chain	1.47	NA	NA	2	9.4
Q5SSJ5	Heterochromatin protein 1-binding protein 3	1.47	2.614	4.2E-01	4	7.8
P19827	Inter-alpha-trypsin inhibitor heavy chain H1	1.46	0.114	3.0E-02	3	4.8
O43175	D-3-phosphoglycerate dehydrogenase	1.46	NA	NA	2	5.1
P09874	Poly [ADP-ribose] polymerase 1	1.45	NA	NA	2	2.3
P14406	Cytochrome c oxidase subunit 7A2, mitochondrial	1.45	NA	NA	2	27.7
O15143	Actin-related protein 2/3 complex subunit 1B	1.45	NA	NA	2	6.7
B5ME19	Eukaryotic translation initiation factor 3 subunit C-like protein	1.45	NA	NA	2	2.3
Q13151	Heterogeneous nuclear ribonucleoprotein A0	1.45	0.124	2.6E-02	5	19.3
P02679	Fibrinogen gamma chain	1.45	0.130	1.8E-02	17	40.2
P30050	60S ribosomal protein L12	1.44	NA	NA	2	17.0
O75533	Splicing factor 3B subunit 1	1.44	NA	NA	2	1.7
Q93050	V-type proton ATPase 116 kDa subunit a isoform 1	1.44	0.158	1.6E-02	4	5.0
Q13185	Chromobox protein homolog 3	1.44	0.067	4.7E-02	3	19.7
P05141	ADP/ATP translocase 2	1.43	0.236	2.3E-01	4	9.1
P43304	Glycerol-3-phosphate dehydrogenase, mitochondrial	1.43	NA	NA	2	3.2
Q9UHX1	Poly(U)-binding-splicing factor PUF60	1.42	NA	NA	2	5.4
P83731	60S ribosomal protein L24	1.42	NA	NA	2	11.5
Q14108	Lysosome membrane protein 2	1.42	0.168	1.2E-01	4	10.5
P17987	T-complex protein 1 subunit alpha	1.42	0.397	2.2E-01	3	5.6
P01024	Complement C3	1.41	0.215	2.2E-02	23	14.6
P37837	Transaldolase	1.41	0.157	1.4E-01	4	11.3
Q9BVK6	Transmembrane emp24 domain-containing protein 9	1.40	NA	NA	2	8.1
P54920	Alpha-soluble NSF attachment protein	1.39	0.529	4.6E-01	3	10.5
P22626	Heterogeneous nuclear ribonucleoproteins A2/B1	1.39	0.056	1.0E-04	12	31.4
P61160	Actin-related protein 2	1.39	0.080	5.5E-02	3	10.7
Q07955	Serine/arginine-rich splicing factor 1	1.39	NA	NA	2	8.5
O15511	Actin-related protein 2/3 complex subunit 5	1.38	0.098	2.4E-01	3	17.2
Q99805	Transmembrane 9 superfamily member 2	1.38	NA	NA	2	3.9
P46459	Vesicle-fusing ATPase	1.38	NA	NA	2	3.1
P39656	Dolichyl-diphosphooligosaccharide--protein glycosyltransferase 48 kDa subunit	1.38	0.125	2.0E-02	5	10.7
Q9Y6N5	Sulfide:quinone oxidoreductase, mitochondrial	1.38	1.616	1.9E-01	5	13.6
P21281	V-type proton ATPase subunit B, brain isoform	1.37	0.090	1.4E-02	4	10.4
Q15365	Poly(rC)-binding protein 1	1.37	0.020	2.6E-01	4	11.2
P12956	X-ray repair cross-complementing protein 6	1.37	0.103	9.9E-03	9	16.1
Q13510	Acid ceramidase	1.37	NA	NA	2	5.1
P06744	Glucose-6-phosphate isomerase	1.37	NA	NA	2	4.3
P07602	Prosaposin	1.37	0.474	2.1E-01	5	8.4
Q13200	26S proteasome non-ATPase regulatory subunit 2	1.37	0.153	1.3E-01	3	3.4
P10599	Thioredoxin	1.36	0.360	7.4E-02	4	39.0
P51665	26S proteasome non-ATPase regulatory subunit 7	1.36	NA	NA	2	9.6
P36543	V-type proton ATPase subunit E 1	1.36	NA	NA	2	7.5
Q9Y394	Dehydrogenase/reductase SDR family member 7	1.35	NA	NA	2	8.8
P14625	Endoplasmin	1.35	0.062	4.6E-05	16	21.9
P26196	Probable ATP-dependent RNA helicase DDX6	1.34	NA	NA	2	5.8
P04075	Fructose-bisphosphate aldolase A	1.34	0.071	8.5E-03	14	44.5
P30533	Alpha-2-macroglobulin receptor-associated protein	1.33	0.178	2.3E-01	3	8.1
Q14697	Neutral alpha-glucosidase AB	1.33	0.101	2.9E-02	9	9.7
P61158	Actin-related protein 3	1.33	4.630	1.9E-01	3	10.3
P46977	Dolichyl-diphosphooligosaccharide--protein glycosyltransferase subunit STT3A	1.33	NA	NA	2	2.3
P53618	Coatomer subunit beta	1.32	NA	NA	2	3.4
P50995	Annexin A11	1.32	0.330	1.2E-01	5	9.3
P08311	Cathepsin G	1.32	0.093	1.4E-02	4	18.8
P30041	Peroxiredoxin-6	1.32	0.257	1.9E-01	5	21.9
P35998	26S protease regulatory subunit 7	1.31	0.301	2.3E-01	3	8.5
Q15582	Transforming growth factor-beta-induced protein ig-h3	1.31	0.217	8.8E-02	7	10.7
Q07020	60S ribosomal protein L18	1.31	NA	NA	2	13.8
Q14152	Eukaryotic translation initiation factor 3 subunit A	1.31	NA	NA	2	1.9
Q9UNM6	26S proteasome non-ATPase regulatory subunit 13	1.31	0.061	9.0E-02	3	8.8
P46939	Utrophin	1.30	1.567	6.0E-01	3	1.1
P27824	Calnexin	1.30	0.317	9.3E-02	10	18.8
O75367	Core histone macro-H2A.1	1.29	0.495	1.8E-01	8	26.3
Q92841	Probable ATP-dependent RNA helicase DDX17	1.29	0.205	1.2E-01	5	7.3
Q9HB90	Ras-related GTP-binding protein C	1.29	NA	NA	2	5.5
P62136	Serine/threonine-protein phosphatase PP1-alpha catalytic subunit	1.29	0.240	4.8E-01	3	8.5
Q9BS26	Endoplasmic reticulum resident protein 44	1.28	0.168	6.3E-02	5	9.9
O43242	26S proteasome non-ATPase regulatory subunit 3	1.28	0.130	1.9E-01	3	4.5
P14866	Heterogeneous nuclear ribonucleoprotein L	1.28	0.305	2.4E-01	4	8.3
Q15233	Non-POU domain-containing octamer-binding protein	1.27	NA	NA	2	4.9
P11177	Pyruvate dehydrogenase E1 component subunit beta, mitochondrial	1.27	NA	NA	2	4.2
O43852	Calumenin	1.26	0.441	2.7E-01	3	14.0
P23396	40S ribosomal protein S3	1.26	0.138	3.8E-02	6	19.8
P00403	Cytochrome c oxidase subunit 2	1.26	NA	NA	2	7.5
Q13263	Transcription intermediary factor 1-beta	1.26	NA	NA	2	2.4
P21266	Glutathione S-transferase Mu 3	1.25	NA	NA	2	9.8
Q8NBQ5	Estradiol 17-beta-dehydrogenase 11	1.25	NA	NA	2	8.7
P07237	Protein disulfide-isomerase	1.25	0.206	1.3E-01	11	18.5
P11021	78 kDa glucose-regulated protein	1.25	0.084	3.8E-03	25	41.1
O00303	Eukaryotic translation initiation factor 3 subunit F	1.25	NA	NA	2	8.1
P0CW22	40S ribosomal protein S17-like	1.25	NA	NA	2	16.3
Q86UP2	Kinectin	1.24	11.418	3.3E-01	5	4.7
Q7L5N1	COP9 signalosome complex subunit 6	1.23	NA	NA	2	6.7
P31943	Heterogeneous nuclear ribonucleoprotein H	1.23	NA	NA	2	6.7
Q00325	Phosphate carrier protein, mitochondrial	1.23	0.292	2.3E-01	6	14.4
P55084	Trifunctional enzyme subunit beta, mitochondrial	1.23	0.116	3.1E-02	10	19.0
P63244	Guanine nucleotide-binding protein subunit beta-2-like 1	1.22	0.082	2.6E-01	3	9.8
P31949	Protein S100-A11	1.22	NA	NA	2	17.1
P02776	Platelet factor 4	1.22	0.797	7.7E-01	3	26.7
P28482	Mitogen-activated protein kinase 1	1.22	NA	NA	2	5.0
P31689	DnaJ homolog subfamily A member 1	1.21	NA	NA	2	4.8
Q8TCT9	Minor histocompatibility antigen H13	1.21	NA	NA	2	5.8
Q8N5K1	CDGSH iron-sulfur domain-containing protein 2	1.21	0.201	2.1E-01	3	23.7
P32969	60S ribosomal protein L9	1.21	NA	NA	2	5.7
Q9Y265	RuvB-like 1	1.21	0.339	5.4E-01	3	11.2
P08575	Receptor-type tyrosine-protein phosphatase C	1.20	NA	NA	2	1.7
P40939	Trifunctional enzyme subunit alpha, mitochondrial	1.20	0.124	1.1E-01	16	22.9
P08697	Alpha-2-antiplasmin	1.20	0.292	6.8E-01	4	11.4
P18124	60S ribosomal protein L7	1.19	0.534	3.5E-01	4	15.7
Q16891	Mitochondrial inner membrane protein	1.19	0.141	4.7E-01	6	9.6

Q16270	Insulin-like growth factor-binding protein 7	1.18	0.305	2.6E-01	3	10.6
P39023	60S ribosomal protein L3	1.18	0.002	7.7E-04	4	10.7
P62829	60S ribosomal protein L23	1.18	NA	NA	2	12.9
P08134	Rho-related GTP-binding protein RhoC	1.18	0.204	4.6E-01	4	17.6
P54709	Sodium/potassium-transporting ATPase subunit beta-3	1.17	0.084	2.4E-01	5	24.0
P60866	40S ribosomal protein S20	1.16	NA	NA	2	19.3
P42765	3-ketoacyl-CoA thiolase, mitochondrial	1.16	NA	NA	2	4.5
P61978	Heterogeneous nuclear ribonucleoprotein K	1.16	0.307	4.2E-01	12	28.1
P61026	Ras-related protein Rab-10	1.15	0.087	2.1E-01	3	15.0
P46781	40S ribosomal protein S9	1.15	2.251	6.7E-01	3	11.3
P62280	40S ribosomal protein S11	1.14	0.237	5.9E-01	3	21.5
P62906	60S ribosomal protein L10a	1.14	0.130	2.5E-01	4	22.1
P04843	Dolichyl-diphosphooligosaccharide--protein glycosyltransferase subunit 1	1.13	0.118	2.4E-01	8	14.8
P01903	HLA class II histocompatibility antigen, DR alpha chain	1.13	0.293	4.4E-01	4	21.3
O75964	ATP synthase subunit g, mitochondrial	1.13	NA	NA	2	27.2
Q96QK1	Vacuolar protein sorting-associated protein 35	1.13	NA	NA	2	2.6
P11413	Glucose-6-phosphate 1-dehydrogenase	1.13	0.772	8.3E-01	3	7.2
P62249	40S ribosomal protein S16	1.12	0.024	9.0E-02	5	34.2
P02749	Beta-2-glycoprotein 1	1.12	0.253	2.5E-01	8	34.8
Q14258	E3 ubiquitin/ISG15 ligase TRIM25	1.12	NA	NA	2	3.8
P08865	40S ribosomal protein SA	1.11	20.731	5.3E-01	7	30.5
P24539	ATP synthase F(0) complex subunit B1, mitochondrial	1.11	NA	NA	2	7.8
P18077	60S ribosomal protein L35a	1.11	NA	NA	2	14.5
P40227	T-complex protein 1 subunit zeta	1.11	0.200	5.5E-01	4	8.1
Q9BV36	Melanophilin	1.11	NA	NA	2	4.8
P25398	40S ribosomal protein S12	1.11	NA	NA	2	13.6
O14980	Exportin-1	1.11	NA	NA	2	2.2
P62701	40S ribosomal protein S4, X isoform	1.11	1.095	7.2E-01	3	9.1
Q9NVI7	ATPase family AAA domain-containing protein 3A	1.10	NA	NA	2	3.9
P25786	Proteasome subunit alpha type-1	1.10	2.271	7.7E-01	4	11.8
P11216	Glycogen phosphorylase, brain form	1.10	2.639	5.0E-01	6	8.4
Q86VP6	Cullin-associated NEDD8-dissociated protein 1	1.10	NA	NA	2	1.5
P17858	ATP-dependent 6-phosphofructokinase, liver type	1.10	0.480	4.4E-01	4	8.7
O60313	Dynamin-like 120 kDa protein, mitochondrial	1.10	1.050	8.2E-01	4	4.8
P17643	5,6-dihydroxyindole-2-carboxylic acid oxidase	1.09	0.182	5.0E-01	4	8.0
P54652	Heat shock-related 70 kDa protein 2	1.09	NA	NA	2	3.9
Q9NZ08	Endoplasmic reticulum aminopeptidase 1	1.09	NA	NA	2	2.2
Q02878	60S ribosomal protein L6	1.08	1.047	6.1E-01	6	19.4
O14773	Tripeptidyl-peptidase 1	1.08	0.076	1.9E-01	3	7.5
O95202	LETM1 and EF-hand domain-containing protein 1, mitochondrial	1.07	0.219	6.7E-01	3	5.0
P30101	Protein disulfide-isomerase A3	1.07	0.190	4.4E-01	14	29.3
P04844	Dolichyl-diphosphooligosaccharide--protein glycosyltransferase subunit 2	1.07	NA	NA	2	4.1
Q02218	2-oxoglutarate dehydrogenase, mitochondrial	1.07	0.126	7.6E-01	10	12.0
P25705	ATP synthase subunit alpha, mitochondrial	1.07	0.142	4.3E-01	14	29.5
P38646	Stress-70 protein, mitochondrial	1.06	0.582	6.1E-01	12	22.5
Q9UBQ0	Vacuolar protein sorting-associated protein 29	1.06	NA	NA	2	12.1
Q99536	Synaptic vesicle membrane protein VAT-1 homolog	1.06	0.127	7.4E-01	10	32.6
P09382	Galectin-1	1.06	0.853	5.7E-01	5	43.7
P01011	Alpha-1-antichymotrypsin	1.05	0.188	5.4E-01	9	22.2
P42704	Leucine-rich PPR motif-containing protein, mitochondrial	1.05	4.782	9.4E-01	5	4.0
Q16401	26S proteasome non-ATPase regulatory subunit 5	1.05	NA	NA	2	5.6
P00338	L-lactate dehydrogenase A chain	1.04	0.417	7.3E-01	4	10.8
P61247	40S ribosomal protein S3a	1.04	0.664	7.4E-01	5	22.3
P13667	Protein disulfide-isomerase A4	1.04	0.364	8.4E-01	5	8.4
P48444	Coatomer subunit delta	1.04	NA	NA	2	4.1
P23246	Splicing factor, proline- and glutamine-rich	1.03	1.587	7.9E-01	6	10.2
P48643	T-complex protein 1 subunit epsilon	1.03	0.930	7.2E-01	5	9.4
P10515	Dihydrolipoyllysine-residue acetyltransferase component of pyruvate dehydrogenase complex, mitochondrial	1.03	0.546	9.5E-01	4	7.0
P40926	Malate dehydrogenase, mitochondrial	1.03	0.879	7.7E-01	7	22.2
P11940	Polyadenylate-binding protein 1	1.02	6.560	9.3E-01	6	11.8
P12111	Collagen alpha-3(VI) chain	1.02	0.916	5.8E-01	48	18.0
P07384	Calpain-1 catalytic subunit	1.02	0.162	7.9E-01	3	5.5
P49368	T-complex protein 1 subunit gamma	1.02	2.531	8.3E-01	7	16.1
Q9HDC9	Adipocyte plasma membrane-associated protein	1.02	NA	NA	2	5.0
P49257	Protein ERGIC-53	1.01	0.323	8.3E-01	5	13.3
P29692	Elongation factor 1-delta	1.01	NA	NA	2	6.4
Q99798	Aconitate hydratase, mitochondrial	1.01	7.893	8.8E-01	5	8.2
P24752	Acetyl-CoA acetyltransferase, mitochondrial	1.01	2.630	9.9E-01	5	11.5
P38159	RNA-binding motif protein, X chromosome	1.00	0.093	1.0E+00	3	7.4
P78371	T-complex protein 1 subunit beta	0.99	0.482	9.8E-01	5	12.7
P18669	Phosphoglycerate mutase 1	0.99	NA	NA	2	15.4
Q9P2R3	Ankyrin repeat and FYVE domain-containing protein 1	0.99	NA	NA	2	2.8
O95292	Vesicle-associated membrane protein-associated protein B/C	0.99	NA	NA	2	13.6
Q5JPE7	Nodal modulator 2	0.99	NA	NA	2	2.2
P30086	Phosphatidylethanolamine-binding protein 1	0.99	0.357	8.8E-01	6	31.0
P30040	Endoplasmic reticulum resident protein 29	0.98	NA	NA	2	8.4
P51991	Heterogeneous nuclear ribonucleoprotein A3	0.98	0.684	8.1E-01	6	19.6
Q9NZM1	Myoferlin	0.98	0.388	8.5E-01	9	4.9
P49207	60S ribosomal protein L34	0.97	NA	NA	2	12.8
P01008	Antithrombin-III	0.97	0.507	8.7E-01	10	23.3
P07195	L-lactate dehydrogenase B chain	0.97	NA	NA	2	6.6
P46777	60S ribosomal protein L5	0.97	0.577	8.5E-01	4	16.8
P02788	Lactotransferrin	0.97	NA	NA	2	3.8
Q15084	Protein disulfide-isomerase A6	0.97	0.101	6.6E-01	6	16.6
Q9UHQ9	NADH-cytochrome b5 reductase 1	0.97	0.403	7.3E-01	4	15.1
Q7L576	Cytoplasmic FMR1-interacting protein 1	0.97	NA	NA	2	2.0
O75643	U5 small nuclear ribonucleoprotein 200 kDa helicase	0.97	NA	NA	2	1.0
P49591	Serine--tRNA ligase, cytoplasmic	0.96	1.346	8.4E-01	4	8.6
P12109	Collagen alpha-1(VI) chain	0.96	0.704	7.0E-01	14	17.0
O43760	Synaptogyrin-2	0.96	NA	NA	2	8.9
Q9Y2Q3	Glutathione S-transferase kappa 1	0.96	1.810	7.2E-01	3	15.5
O75396	Vesicle-trafficking protein SEC22b	0.95	0.477	8.7E-01	3	18.1
P28331	NADH-ubiquinone oxidoreductase 75 kDa subunit, mitochondrial	0.95	NA	NA	2	5.1
O94979	Protein transport protein Sec31A	0.95	NA	NA	2	2.1
Q06323	Proteasome activator complex subunit 1	0.95	1.494	8.4E-01	4	17.3
P36957	Dihydrolipoyllysine-residue succinyltransferase component of 2-oxoglutarate dehydrogenase complex, mitochondrial	0.95	0.151	7.7E-01	5	12.8
P62269	40S ribosomal protein S18	0.95	0.118	5.5E-01	4	17.8
P52907	F-actin-capping protein subunit alpha-1	0.95	NA	NA	2	13.3
P42025	Beta-centractin	0.95	NA	NA	2	5.1
P62913	60S ribosomal protein L11	0.94	1.314	9.1E-01	3	16.9
P55209	Nucleosome assembly protein 1-like 1	0.94	NA	NA	2	7.7
P05387	60S acidic ribosomal protein P2	0.94	1.105	9.3E-01	3	25.2
P50991	T-complex protein 1 subunit delta	0.94	0.971	6.6E-01	5	11.3
P05198	Eukaryotic translation initiation factor 2 subunit 1	0.94	NA	NA	2	7.0
Q9Y4L1	Hypoxia up-regulated protein 1	0.93	0.508	6.6E-01	6	6.4
P36542	ATP synthase subunit gamma, mitochondrial	0.93	NA	NA	2	7.0
P50990	T-complex protein 1 subunit theta	0.92	0.098	2.0E-01	8	13.7

Q9NSE4	Isoleucine--tRNA ligase, mitochondrial	0.92	NA	NA	2	3.1
P0DME0	Protein SETSIP	0.92	1.362	7.6E-01	3	14.9
P49189	4-trimethylaminobutyraldehyde dehydrogenase	0.92	NA	NA	2	4.7
P48047	ATP synthase subunit O, mitochondrial	0.91	0.163	5.8E-01	4	26.3
P13073	Cytochrome c oxidase subunit 4 isoform 1, mitochondrial	0.91	1.742	6.2E-01	3	20.1
Q14254	Flotillin-2	0.91	0.358	8.5E-01	5	10.5
P12110	Collagen alpha-2(VI) chain	0.91	0.166	2.0E-01	11	13.2
P07358	Complement component C8 beta chain	0.91	1.690	8.4E-01	3	5.2
P36578	60S ribosomal protein L4	0.91	0.040	1.3E-01	5	12.6
Q00610	Clathrin heavy chain 1	0.90	0.324	1.5E-01	31	21.3
P99999	Cytochrome c	0.90	NA	NA	2	18.1
P26038	Moesin	0.89	0.187	5.6E-01	6	9.9
P62263	40S ribosomal protein S14	0.89	0.365	6.5E-01	3	29.8
P30049	ATP synthase subunit delta, mitochondrial	0.89	NA	NA	2	13.7
P04792	Heat shock protein beta-1	0.89	0.472	2.7E-01	9	51.2
Q9UGP8	Translocation protein SEC63 homolog	0.89	NA	NA	2	3.2
Q99832	T-complex protein 1 subunit eta	0.88	0.103	4.0E-01	4	9.6
O75947	ATP synthase subunit d, mitochondrial	0.88	2.495	7.8E-01	4	22.4
Q14956	Transmembrane glycoprotein NMB	0.87	0.852	7.3E-01	3	6.5
Q9Y277	Voltage-dependent anion-selective channel protein 3	0.87	0.217	3.1E-01	4	12.4
P55884	Eukaryotic translation initiation factor 3 subunit B	0.87	NA	NA	2	3.1
Q9P2E9	Ribosome-binding protein 1	0.86	0.135	1.8E-01	5	4.6
Q9UBS4	DnaJ homolog subfamily B member 11	0.86	NA	NA	2	7.5
O75489	NADH dehydrogenase [ubiquinone] iron-sulfur protein 3, mitochondrial	0.86	0.031	4.0E-02	3	9.5
P62753	40S ribosomal protein S6	0.85	0.146	3.7E-01	4	16.5
P47756	F-actin-capping protein subunit beta	0.85	0.171	1.8E-01	3	11.6
Q14165	Malectin	0.85	NA	NA	2	5.5
P09622	Dihydrolipoyl dehydrogenase, mitochondrial	0.85	2.279	7.0E-01	4	9.2
P27797	Calreticulin	0.84	0.720	5.2E-01	5	12.2
P00450	Ceruloplasmin	0.84	0.185	1.9E-01	11	14.3
Q10567	AP-1 complex subunit beta-1	0.84	0.141	2.1E-01	4	3.1
Q07065	Cytoskeleton-associated protein 4	0.84	0.176	1.3E-01	4	8.8
Q13011	Delta(3,5)-Delta(2,4)-dienoyl-CoA isomerase, mitochondrial	0.84	1.481	7.6E-01	3	9.5
P51608	Methyl-CpG-binding protein 2	0.84	NA	NA	2	5.1
P15880	40S ribosomal protein S2	0.83	0.096	1.6E-01	3	13.7
Q9Y4F1	FERM, RhoGEF and pleckstrin domain-containing protein 1	0.83	NA	NA	2	2.6
P27635	60S ribosomal protein L10	0.83	0.703	4.7E-01	3	15.0
P14314	Glucosidase 2 subunit beta	0.82	0.094	7.7E-02	6	10.0
P06753	Tropomyosin alpha-3 chain	0.82	0.196	3.1E-01	3	12.6
P60709	Actin, cytoplasmic 1	0.81	0.151	2.0E-02	7	26.1
Q92499	ATP-dependent RNA helicase DDX1	0.81	NA	NA	2	4.1
P06703	Protein S100-A6	0.81	NA	NA	2	16.7
P08133	Annexin A6	0.81	0.105	6.2E-04	34	56.2
P08107	Heat shock 70 kDa protein 1A/1B	0.81	0.212	3.2E-01	8	11.5
Q7KZF4	Staphylococcal nuclease domain-containing protein 1	0.81	0.023	2.5E-02	3	3.7
P24534	Elongation factor 1-beta	0.81	0.394	4.8E-01	3	13.8
Q99714	3-hydroxyacyl-CoA dehydrogenase type-2	0.80	NA	NA	2	7.7
O95782	AP-2 complex subunit alpha-1	0.79	NA	NA	2	3.7
Q14203	Dynactin subunit 1	0.78	NA	NA	2	2.3
Q07954	Prolow-density lipoprotein receptor-related protein 1	0.78	0.077	3.5E-02	5	1.7
P04632	Calpain small subunit 1	0.78	0.226	2.2E-01	3	11.2
Q14103	Heterogeneous nuclear ribonucleoprotein D0	0.77	NA	NA	2	9.3
P38117	Electron transfer flavoprotein subunit beta	0.77	NA	NA	2	11.4
P16615	Sarcoplasmic/endoplasmic reticulum calcium ATPase 2	0.77	0.163	6.1E-02	7	8.1
P48735	Isocitrate dehydrogenase [NADP], mitochondrial	0.77	0.086	4.7E-02	6	15.3
Q07666	KH domain-containing, RNA-binding, signal transduction-associated protein 1	0.77	0.200	2.4E-01	4	7.9
Q15165	Serum paraoxonase/arylesterase 2	0.76	NA	NA	2	7.9
P16157	Ankyrin-1	0.76	0.149	7.9E-02	4	3.1
Q15293	Reticulocalbin-1	0.76	0.436	5.4E-01	4	13.0
Q12797	Aspartyl/asparaginyl beta-hydroxylase	0.76	0.105	1.0E-01	5	4.7
P35579	Myosin-9	0.76	0.052	4.4E-06	69	34.9
Q96CW1	AP-2 complex subunit mu	0.75	0.225	6.3E-01	3	9.4
Q14204	Cytoplasmic dynein 1 heavy chain 1	0.75	0.082	4.2E-03	24	6.4
Q15149	Plectin	0.75	0.072	1.5E-03	45	10.9
P20700	Lamin-B1	0.75	0.262	1.7E-01	5	8.2
P02649	Apolipoprotein E	0.75	0.124	1.3E-02	15	49.2
P08571	Monocyte differentiation antigen CD14	0.74	0.214	1.9E-01	3	7.5
P07437	Tubulin beta chain	0.74	0.131	8.5E-02	4	16.0
P16403	Histone H1.2	0.74	0.149	2.5E-01	4	15.5
P09429	High mobility group protein B1	0.74	0.055	8.2E-02	3	17.2
P15311	Ezrin	0.74	0.081	2.0E-01	3	4.9
P02549	Spectrin alpha chain, erythrocytic 1	0.74	0.108	1.0E-02	11	5.7
P15144	Aminopeptidase N	0.73	0.440	8.2E-01	3	4.2
P27105	Erythrocyte band 7 integral membrane protein	0.73	0.192	1.2E-01	5	21.2
P09543	2',3'-cyclic-nucleotide 3'-phosphodiesterase	0.73	0.479	3.8E-01	5	11.2
Q07021	Complement component 1 Q subcomponent-binding protein, mitochondrial	0.72	NA	NA	2	11.0
P17931	Galectin-3	0.72	0.110	5.6E-03	6	28.4
Q14980	Nuclear mitotic apparatus protein 1	0.72	3.126	5.2E-01	3	2.2
P62851	40S ribosomal protein S25	0.72	0.123	4.7E-02	3	16.0
Q9NVA2	Septin-11	0.72	0.400	2.5E-01	3	7.2
Q14764	Major vault protein	0.71	0.330	2.6E-01	5	6.5
P39019	40S ribosomal protein S19	0.71	0.157	1.0E-01	3	18.6
P09936	Ubiquitin carboxyl-terminal hydrolase isozyme L1	0.71	NA	NA	2	11.7
P55072	Transitional endoplasmic reticulum ATPase	0.71	0.150	7.2E-02	10	13.4
P02730	Band 3 anion transport protein	0.70	0.097	1.9E-02	11	16.9
P04179	Superoxide dismutase [Mn], mitochondrial	0.70	0.142	1.2E-01	5	17.1
O75131	Copine-3	0.70	0.088	1.2E-02	3	6.3
Q53GQ0	Estradiol 17-beta-dehydrogenase 12	0.69	0.448	4.6E-01	3	12.5
P32119	Peroxiredoxin-2	0.69	0.141	3.5E-02	4	18.2
P50914	60S ribosomal protein L14	0.69	NA	NA	2	10.2
P11277	Spectrin beta chain, erythrocytic	0.69	0.151	4.3E-03	16	11.6
P06396	Gelsolin	0.69	0.076	7.8E-04	12	18.4
P00740	Coagulation factor IX	0.69	NA	NA	2	3.3
P30048	Thioredoxin-dependent peroxide reductase, mitochondrial	0.68	0.257	1.0E-01	3	14.1
Q9NY15	Stabilin-1	0.68	NA	NA	2	1.3
O15173	Membrane-associated progesterone receptor component 2	0.67	0.092	4.5E-02	4	22.0
P00367	Glutamate dehydrogenase 1, mitochondrial	0.67	0.170	5.9E-02	5	9.9
Q7Z7G0	Target of Nesh-SH3	0.67	NA	NA	2	3.2
Q99584	Protein S100-A13	0.67	0.095	2.2E-02	4	41.8
Q9UBI6	Guanine nucleotide-binding protein G(I)/G(S)/G(O) subunit gamma-12	0.66	NA	NA	2	41.7
P62277	40S ribosomal protein S13	0.66	0.404	3.4E-01	3	17.9
P05362	Intercellular adhesion molecule 1	0.65	NA	NA	2	5.3
P30042	ES1 protein homolog, mitochondrial	0.65	NA	NA	2	9.3
Q9BS40	Latexin	0.64	NA	NA	2	12.2
P62424	60S ribosomal protein L7a	0.64	NA	NA	2	6.4
Q15019	Septin-2	0.64	0.087	3.1E-02	5	18.6
O60504	Vinexin	0.64	NA	NA	2	5.2

Q99460	26S proteasome non-ATPase regulatory subunit 1	0.63	NA	NA	2	2.7
Q9NVD7	Alpha-parvin	0.63	0.231	2.0E-01	3	9.4
Q9P0L0	Vesicle-associated membrane protein-associated protein A	0.63	NA	NA	2	10.0
Q96D15	Reticulocalbin-3	0.63	NA	NA	2	8.8
O00468	Agrin	0.63	0.118	3.1E-02	6	4.4
P50502	Hsc70-interacting protein	0.62	0.289	2.2E-01	3	7.9
Q9NQC3	Reticulon-4	0.62	0.178	1.4E-01	3	2.7
P29590	Protein PML	0.61	NA	NA	2	3.3
Q9NZN4	EH domain-containing protein 2	0.61	NA	NA	2	5.2
O43865	Putative adenosylhomocysteinase 2	0.61	NA	NA	2	3.6
P35580	Myosin-10	0.61	0.093	6.0E-03	12	8.0
P13861	cAMP-dependent protein kinase type II-alpha regulatory subunit	0.61	0.152	2.2E-01	3	11.4
O95831	Apoptosis-inducing factor 1, mitochondrial	0.60	NA	NA	2	5.2
P60033	CD81 antigen	0.60	NA	NA	2	11.9
Q16181	Septin-7	0.60	0.067	3.6E-03	6	16.2
P08572	Collagen alpha-2(IV) chain	0.60	0.134	6.9E-03	8	5.8
P62917	60S ribosomal protein L8	0.60	0.251	1.5E-01	3	13.2
P50402	Emerin	0.60	NA	NA	2	9.8
Q13418	Integrin-linked protein kinase	0.60	NA	NA	2	5.3
P67936	Tropomyosin alpha-4 chain	0.60	0.205	1.7E-02	8	27.4
P40429	60S ribosomal protein L13a	0.60	NA	NA	2	9.9
P00738	Haptoglobin	0.59	0.168	7.4E-02	6	18.5
Q8WUM4	Programmed cell death 6-interacting protein	0.59	0.346	2.3E-01	3	3.2
P84103	Serine/arginine-rich splicing factor 3	0.59	NA	NA	2	14.0
P11166	Solute carrier family 2, facilitated glucose transporter member 1	0.59	0.092	7.2E-03	3	7.1
P11047	Laminin subunit gamma-1	0.58	0.110	1.2E-03	16	10.4
Q9BZZ2	Sialoadhesin	0.58	NA	NA	2	1.3
P26373	60S ribosomal protein L13	0.57	0.336	3.6E-01	3	15.2
P07942	Laminin subunit beta-1	0.57	0.200	3.0E-02	4	3.1
Q71U36	Tubulin alpha-1A chain	0.56	0.234	2.9E-02	4	13.1
P62244	40S ribosomal protein S15a	0.56	NA	NA	2	13.1
P05556	Integrin beta-1	0.56	0.148	1.7E-02	5	7.5
P02462	Collagen alpha-1(IV) chain	0.56	0.293	5.7E-02	4	3.3
P68371	Tubulin beta-4B chain	0.56	0.128	3.8E-03	3	9.9
Q9UHG3	Prenylcysteine oxidase 1	0.56	NA	NA	2	4.4
P08670	Vimentin	0.55	0.039	5.3E-15	27	55.4
P39060	Collagen alpha-1(XVIII) chain	0.55	0.124	8.2E-05	8	5.5
P09619	Platelet-derived growth factor receptor beta	0.55	NA	NA	2	2.9
O15230	Laminin subunit alpha-5	0.55	0.101	8.6E-05	16	5.9
P42167	Lamina-associated polypeptide 2, isoforms beta/gamma	0.55	0.307	1.2E-01	4	10.1
P60660	Myosin light polypeptide 6	0.55	0.040	1.4E-04	9	58.3
Q9UHD8	Septin-9	0.55	0.221	3.8E-01	4	8.9
O75915	PRA1 family protein 3	0.55	0.399	2.7E-01	3	19.7
P04083	Annexin A1	0.55	0.036	7.4E-04	14	46.0
Q08722	Leukocyte surface antigen CD47	0.54	NA	NA	2	5.9
P61604	10 kDa heat shock protein, mitochondrial	0.54	0.110	5.3E-02	3	29.4
P22413	Ectonucleotide pyrophosphatase/phosphodiesterase family member 1	0.54	NA	NA	2	2.7
Q08431	Lactadherin	0.53	NA	NA	2	7.5
Q13509	Tubulin beta-3 chain	0.53	0.072	6.3E-03	3	8.9
P05023	Sodium/potassium-transporting ATPase subunit alpha-1	0.53	0.075	2.7E-04	11	12.4
Q9Y2J2	Band 4.1-like protein 3	0.53	NA	NA	2	3.3
Q6DD88	Atlastin-3	0.53	0.191	2.9E-02	4	7.2
Q06830	Peroxiredoxin-1	0.53	0.094	1.5E-05	6	32.2
P23634	Plasma membrane calcium-transporting ATPase 4	0.53	NA	NA	2	2.8
P20073	Annexin A7	0.52	0.088	3.9E-02	4	11.7
P13671	Complement component C6	0.52	0.277	1.9E-01	4	6.5
P10606	Cytochrome c oxidase subunit 5B, mitochondrial	0.52	0.205	2.2E-02	3	15.5
P17612	cAMP-dependent protein kinase catalytic subunit alpha	0.52	0.068	7.1E-02	3	6.8
O94905	Erlin-2	0.51	0.087	8.3E-04	6	16.8
Q9Y490	Talin-1	0.51	0.071	4.5E-07	24	12.4
P12814	Alpha-actinin-1	0.50	0.131	2.5E-03	10	14.3
Q03252	Lamin-B2	0.50	0.080	3.4E-06	15	24.2
P02545	Prelamin-A/C	0.50	0.028	0.0E+00	30	41.0
P04216	Thy-1 membrane glycoprotein	0.50	0.194	6.6E-02	3	24.2
P26447	Protein S100-A4	0.49	0.138	1.8E-02	3	27.7
Q13561	Dynactin subunit 2	0.48	NA	NA	2	8.2
O60814	Histone H2B type 1-K	0.48	NA	NA	2	7.9
Q9BSJ8	Extended synaptotagmin-1	0.48	0.408	3.0E-01	3	3.0
Q9P0M6	Core histone macro-H2A.2	0.48	NA	NA	2	5.4
Q16363	Laminin subunit alpha-4	0.47	0.211	2.1E-02	8	4.8
P49411	Elongation factor Tu, mitochondrial	0.47	0.189	3.2E-02	5	13.3
P35222	Catenin beta-1	0.47	NA	NA	2	4.0
P55268	Laminin subunit beta-2	0.47	0.085	1.1E-07	14	8.8
P0C0S5	Histone H2A.Z	0.46	NA	NA	2	18.8
P59768	Guanine nucleotide-binding protein G(I)/G(S)/G(O) subunit gamma-2	0.46	NA	NA	2	42.3
P98160	Basement membrane-specific heparan sulfate proteoglycan core protein	0.45	0.045	9.4E-12	33	10.1
Q12805	EGF-containing fibulin-like extracellular matrix protein 1	0.45	NA	NA	2	3.9
Q8WXH0	Nesprin-2	0.45	NA	NA	2	0.3
P08294	Extracellular superoxide dismutase [Cu-Zn]	0.45	NA	NA	2	12.1
Q9BT22	Chitobiosyldiphosphodolichol beta-mannosyltransferase	0.44	NA	NA	2	3.9
O60716	Catenin delta-1	0.44	NA	NA	2	2.3
P35611	Alpha-adducin	0.44	NA	NA	2	6.0
P07357	Complement component C8 alpha chain	0.44	NA	NA	2	7.0
P26583	High mobility group protein B2	0.44	NA	NA	2	12.9
O75369	Filamin-B	0.43	0.174	6.9E-02	7	3.3
Q93052	Lipoma-preferred partner	0.43	NA	NA	2	4.2
P00352	Retinal dehydrogenase 1	0.43	NA	NA	2	2.0
P05091	Aldehyde dehydrogenase, mitochondrial	0.43	NA	NA	2	4.3
P07360	Complement component C8 gamma chain	0.43	0.199	5.9E-02	3	23.3
O14786	Neuropilin-1	0.42	NA	NA	2	3.1
P43121	Cell surface glycoprotein MUC18	0.41	NA	NA	2	3.4
P23142	Fibulin-1	0.41	NA	NA	2	3.3
Q9Y240	C-type lectin domain family 11 member A	0.40	NA	NA	2	7.7
Q14118	Dystroglycan	0.40	NA	NA	2	2.5
Q14344	Guanine nucleotide-binding protein subunit alpha-13	0.40	0.242	2.0E-01	3	7.7
Q2UY09	Collagen alpha-1(XXVIII) chain	0.40	NA	NA	2	1.8
P29966	Myristoylated alanine-rich C-kinase substrate	0.39	0.345	4.2E-01	3	12.3
Q9BTV4	Transmembrane protein 43	0.39	NA	NA	2	6.3
P55060	Exportin-2	0.37	NA	NA	2	2.3
Q9Y3I0	tRNA-splicing ligase RtcB homolog	0.37	NA	NA	2	4.6
P09493	Tropomyosin alpha-1 chain	0.36	0.516	1.4E-01	3	8.8
Q96CX2	BTB/POZ domain-containing protein KCTD12	0.36	NA	NA	2	7.4
O14950	Myosin regulatory light chain 12B	0.35	NA	NA	2	12.2
P54289	Voltage-dependent calcium channel subunit alpha-2/delta-1	0.34	NA	NA	2	2.1
O43301	Heat shock 70 kDa protein 12A	0.34	NA	NA	2	2.8
Q92522	Histone H1x	0.33	NA	NA	2	11.7
Q96HC4	PDZ and LIM domain protein 5	0.32	NA	NA	2	3.5

P27816	Microtubule-associated protein 4	0.29	NA	NA	2	2.0
O94832	Unconventional myosin-Id	0.28	NA	NA	2	2.4
Q05682	Caldesmon	0.28	NA	NA	2	3.7
P46821	Microtubule-associated protein 1B	0.27	0.487	1.5E-01	4	2.0
P08123	Collagen alpha-2(I) chain	0.26	NA	NA	2	2.4
Q9UBX5	Fibulin-5	0.26	NA	NA	2	5.1
P07305	Histone H1.0	0.26	NA	NA	2	9.3
P17661	Desmin	0.25	NA	NA	2	4.0
P58166	Inhibin beta E chain	0.25	NA	NA	2	5.7
P22352	Glutathione peroxidase 3	0.24	NA	NA	2	8.0
P09497	Clathrin light chain B	0.23	NA	NA	2	8.3
P06899	Histone H2B type 1-J	0.21	NA	NA	2	7.9
P07197	Neurofilament medium polypeptide	0.21	NA	NA	2	3.4
Q16853	Membrane primary amine oxidase	0.21	NA	NA	2	2.6
O94875	Sorbin and SH3 domain-containing protein 2	0.21	NA	NA	2	2.5
P35637	RNA-binding protein FUS	0.20	0.234	1.8E-01	3	6.1
P43320	Beta-crystallin B2	0.17	NA	NA	2	11.7
P10745	Retinol-binding protein 3	0.14	NA	NA	2	2.1
P24844	Myosin regulatory light polypeptide 9	0.13	NA	NA	2	12.2
O14495	Lipid phosphate phosphohydrolase 3	0.13	NA	NA	2	6.8
Q9BXM0	Periaxin	0.10	0.392	6.4E-02	3	1.4
P63211	Guanine nucleotide-binding protein G(T) subunit gamma-T1	0.07	NA	NA	2	17.6
O00159	Unconventional myosin-Ic	0.44	0.091	2.0E-03	10	10.9
Q14112	Nidogen-2	0.43	0.051	8.1E-07	8	7.1
O00264	Membrane-associated progesterone receptor component 1	0.41	0.087	6.9E-04	4	15.9
P02511	Alpha-crystallin B chain	0.41	0.133	9.2E-03	4	21.7
P07355	Annexin A2	0.40	0.036	0.0E+00	22	58.7
P04899	Guanine nucleotide-binding protein G(i) subunit alpha-2	0.40	0.064	3.1E-04	5	15.5
P14543	Nidogen-1	0.39	0.056	8.6E-06	10	9.1
P62987	Ubiquitin-60S ribosomal protein L40	0.39	0.081	6.4E-09	5	41.4
P68366	Tubulin alpha-4A chain	0.39	0.193	5.3E-03	3	8.5
P10643	Complement component C7	0.39	0.209	1.2E-02	5	7.2
P50895	Basal cell adhesion molecule	0.38	0.136	7.6E-04	3	6.8
P04004	Vitronectin	0.38	0.124	3.6E-06	8	16.9
P21333	Filamin-A	0.38	0.043	7.5E-14	54	28.6
P13987	CD59 glycoprotein	0.37	0.072	1.5E-04	3	25.0
Q8IWA5	Choline transporter-like protein 2	0.36	0.206	9.1E-03	3	4.1
Q16695	Histone H3.1t	0.36	0.050	2.2E-07	4	17.6
O43707	Alpha-actinin-4	0.36	0.097	2.0E-05	11	16.0
Q9BXN1	Asporin	0.36	0.097	1.6E-04	3	11.3
P00387	NADH-cytochrome b5 reductase 3	0.35	0.114	1.6E-03	6	23.3
P01031	Complement C5	0.35	0.092	2.5E-06	10	5.7
P62805	Histone H4	0.35	0.043	1.6E-13	7	52.4
P07099	Epoxide hydrolase 1	0.35	0.130	3.2E-03	8	20.4
Q9Y6C2	EMILIN-1	0.34	0.054	1.2E-03	7	8.8
P36269	Gamma-glutamyltransferase 5	0.33	0.202	3.5E-03	4	8.0
Q05707	Collagen alpha-1(XIV) chain	0.32	0.148	1.6E-03	10	6.1
P39059	Collagen alpha-1(XV) chain	0.32	0.137	4.5E-04	6	5.0
Q6NZI2	Polymerase I and transcript release factor	0.31	0.177	3.2E-03	5	18.7
P18206	Vinculin	0.31	0.130	1.8E-05	10	11.6
P05186	Alkaline phosphatase, tissue-nonspecific isozyme	0.28	0.175	3.9E-03	4	8.8
P10909	Clusterin	0.28	0.049	6.0E-11	15	33.0
Q01995	Transgelin	0.28	0.129	1.5E-02	4	23.4
P35555	Fibrillin-1	0.27	0.040	1.1E-12	47	18.8
P02686	Myelin basic protein	0.26	0.252	5.0E-02	3	10.5
Q13885	Tubulin beta-2A chain	0.26	0.092	5.6E-05	3	8.1
P68032	Actin, alpha cardiac muscle 1	0.26	0.203	1.6E-04	7	28.9
P51884	Lumican	0.26	0.059	6.0E-14	12	40.8
Q09666	Neuroblast differentiation-associated protein AHNAK	0.25	0.106	1.2E-05	33	4.5
P01871	Ig mu chain C region	0.25	0.105	6.7E-06	10	27.0
P21926	CD9 antigen	0.25	0.162	2.4E-02	3	9.6
P04275	von Willebrand factor	0.24	0.121	1.9E-06	11	5.3
Q16555	Dihydropyrimidinase-related protein 2	0.22	0.120	4.2E-05	9	20.8
Q01082	Spectrin beta chain, non-erythrocytic 1	0.22	0.055	6.0E-15	41	19.8
P00167	Cytochrome b5	0.22	0.162	8.0E-03	3	35.8
Q13813	Spectrin alpha chain, non-erythrocytic 1	0.22	0.069	2.9E-15	62	28.1
O43491	Band 4.1-like protein 2	0.21	0.142	2.0E-05	7	8.8
P80723	Brain acid soluble protein 1	0.21	0.096	1.8E-04	6	46.7
P51888	Prolargin	0.20	0.063	3.6E-15	10	28.8
P35625	Metalloproteinase inhibitor 3	0.20	0.172	1.5E-05	6	30.3
P23946	Chymase	0.20	0.249	5.4E-03	4	21.1
P21810	Biglycan	0.20	0.072	1.5E-10	15	49.7
P60903	Protein S100-A10	0.19	0.118	5.9E-07	4	35.1
P07585	Decorin	0.19	0.113	5.0E-04	7	21.7
P15088	Mast cell carboxypeptidase A	0.19	0.238	3.6E-02	5	12.0
Q14195	Dihydropyrimidinase-related protein 3	0.19	0.071	4.1E-05	4	10.0
P21980	Protein-glutamine gamma-glutamyltransferase 2	0.18	0.092	1.4E-13	18	28.2
P41219	Peripherin	0.18	0.234	1.6E-05	12	26.8
Q03135	Caveolin-1	0.18	0.150	7.0E-03	3	13.5
P02760	Protein AMBP	0.17	0.120	1.5E-06	4	19.0
P35749	Myosin-11	0.17	0.097	2.4E-10	34	18.9
P02748	Complement component C9	0.17	0.080	2.1E-13	12	21.8
Q15661	Tryptase alpha/beta-1	0.15	0.213	2.2E-04	5	21.8
P02743	Serum amyloid P-component	0.15	0.186	3.6E-04	5	20.6
P20774	Mimecan	0.14	0.174	3.0E-06	6	16.4
P22748	Carbonic anhydrase 4	0.13	0.224	7.5E-05	5	14.7
Q02952	A-kinase anchor protein 12	0.13	0.182	4.8E-03	11	9.3
P25189	Myelin protein P0	0.11	0.225	1.6E-05	7	27.4

Brown denotes change ≥ 2 standard deviations (SD) from the mean, yellow denotes change ≥ 1 SD and green highlights p values ≤ 0.05. NA, not applicable, n<3 unique peptides.
