# Supplementary material for: iTRAQ Quantitative Proteomic Comparison of Metastatic and Non-Metastatic Uveal Melanoma Tumors
Source: PLoS One. 2015 Aug 25;10(8):e0135543. doi: 10.1371/journal.pone.0135543 (PMC4549237; doi:10.1371/journal.pone.0135543)
Supplement: S4 Table — (PDF) [file pone.0135543.s004.pdf]

Supplementary Table S4
Relative Protein Abundance: Sample UM28, Metastatic
Total Proteins Quantified = 948; LogMedian Protein Ratio = .20; LogMean Protein Ratio = 0; Standard Deviation = 0.95

Uni-Prot Accession	Protein	Ratio UM/Control	Standard Deviation	*p* value	Unique Peptides	% Sequence Coverage
P06454	Prothymosin alpha	13.76	0.330	1.8E-02	3	21.6
P62937	Peptidyl-prolyl cis-trans isomerase A	9.40	0.099	0.0E+00	7	38.2
P23381	Tryptophan--tRNA ligase, cytoplasmic	8.28	0.168	2.5E-07	8	14.4
P16401	Histone H1.5	7.60	0.134	1.8E-06	7	15.9
P05413	Fatty acid-binding protein, heart	7.44	0.164	4.0E-03	4	28.6
P78417	Glutathione S-transferase omega-1	5.99	0.144	1.3E-09	9	31.1
P05534	HLA class I histocompatibility antigen, A-24 alpha chain	5.64	0.188	4.1E-05	5	21.4
P00558	Phosphoglycerate kinase 1	5.26	0.115	1.9E-09	14	30.0
P19338	Nucleolin	5.25	0.071	0.0E+00	20	25.5
P06748	Nucleophosmin	5.25	0.094	4.0E-10	7	25.5
P15531	Nucleoside diphosphate kinase A	4.83	0.060	4.6E-11	6	43.4
P61088	Ubiquitin-conjugating enzyme E2 N	4.80	0.208	2.3E-03	5	34.9
P23528	Cofilin-1	4.71	0.103	1.3E-07	11	54.8
P16070	CD44 antigen	4.70	0.059	2.5E-04	8	10.6
P07737	Profilin-1	4.68	0.178	7.1E-07	6	44.3
P60174	Triosephosphate isomerase	4.55	0.089	6.3E-05	14	48.3
P01889	HLA class I histocompatibility antigen, B-7 alpha chain	4.45	0.195	3.1E-02	3	12.7
P06733	Alpha-enolase	4.38	0.067	3.0E-07	15	41.9
P63104	14-3-3 protein zeta/delta	4.26	0.089	9.8E-06	5	26.5
O75368	SH3 domain-binding glutamic acid-rich-like protein	4.15	0.210	7.4E-04	3	20.2
P30043	Flavin reductase (NADPH)	4.14	0.158	4.7E-04	3	18.9
P10412	Histone H1.4	4.03	0.083	1.1E-09	6	15.5
P63241	Eukaryotic translation initiation factor 5A-1	4.00	0.140	2.5E-03	4	13.6
Q9Y2S2	Lambda-crystallin homolog	4.00	0.356	2.1E-02	5	21.6
P02768	Serum albumin	3.95	0.035	0.0E+00	42	61.4
P07108	Acyl-CoA-binding protein	3.93	0.197	8.0E-04	3	50.6
Q99729	Heterogeneous nuclear ribonucleoprotein A/B	3.91	0.083	1.8E-05	4	10.5
P62750	60S ribosomal protein L23a	3.89	0.074	2.0E-05	4	26.9
P51858	Hepatoma-derived growth factor	3.86	0.148	3.6E-03	3	14.6
P40925	Malate dehydrogenase, cytoplasmic	3.81	0.154	1.0E-04	7	24.6
P22087	rRNA 2'-O-methyltransferase fibrillarin	3.78	0.083	2.4E-04	6	17.8
P04406	Glyceraldehyde-3-phosphate dehydrogenase	3.70	0.057	0.0E+00	11	39.1
P07686	Beta-hexosaminidase subunit beta	3.68	0.369	3.8E-02	3	5.9
P30086	Phosphatidylethanolamine-binding protein 1	3.66	0.068	1.0E-06	7	39.0
P06744	Glucose-6-phosphate isomerase	3.64	0.110	9.8E-05	6	11.5
P07195	L-lactate dehydrogenase B chain	3.53	0.069	7.2E-10	7	21.6
P00338	L-lactate dehydrogenase A chain	3.51	0.058	2.0E-10	7	17.8
P04080	Cystatin-B	3.51	0.090	2.8E-05	3	45.9
P02787	Serotransferrin	3.50	0.064	2.3E-12	22	32.5
Q9UL46	Proteasome activator complex subunit 2	3.49	0.160	6.3E-03	4	21.8
P13796	Plastin-2	3.47	0.262	2.4E-02	3	6.2
Q00796	Sorbitol dehydrogenase	3.45	0.084	3.5E-02	3	9.8
P07741	Adenine phosphoribosyltransferase	3.42	0.252	1.9E-02	3	20.0
P23526	Adenosylhomocysteinase	3.37	0.122	1.2E-05	6	16.0
P29401	Transketolase	3.34	0.079	4.2E-06	9	13.6
P31948	Stress-induced-phosphoprotein 1	3.31	0.102	1.3E-07	11	17.9
Q99497	Protein DJ-1	3.26	0.136	3.3E-03	6	28.0
P09429	High mobility group protein B1	3.26	0.125	3.3E-05	6	27.0
Q01105	Protein SET	3.25	0.105	1.2E-04	7	26.2
P99999	Cytochrome c	3.23	0.144	1.9E-04	4	39.0
Q08380	Galectin-3-binding protein	3.17	0.243	4.8E-04	7	15.0
Q06323	Proteasome activator complex subunit 1	3.17	0.060	7.8E-10	10	37.3
P26447	Protein S100-A4	3.15	0.047	3.1E-08	4	35.6
Q13838	Spliceosome RNA helicase DDX39B	3.14	0.074	3.9E-07	8	21.3
Q8NC51	Plasminogen activator inhibitor 1 RNA-binding protein	3.12	0.116	1.2E-04	7	14.5
P54819	Adenylate kinase 2, mitochondrial	3.10	0.150	2.7E-03	4	18.8
P14618	Pyruvate kinase PKM	3.07	0.072	8.3E-13	16	33.9
Q12906	Interleukin enhancer-binding factor 3	3.04	0.099	1.6E-06	15	18.6
Q12931	Heat shock protein 75 kDa, mitochondrial	3.03	0.152	1.0E-03	5	7.8
O95336	6-phosphogluconolactonase	3.00	0.127	1.5E-02	4	22.9
P08758	Annexin A5	2.97	0.043	0.0E+00	13	41.6
Q9Y2X3	Nucleolar protein 58	2.88	0.165	5.8E-03	6	11.9
P49588	Alanine--tRNA ligase, cytoplasmic	2.82	0.159	7.1E-03	4	4.3
P08238	Heat shock protein HSP 90-beta	2.82	0.091	2.8E-06	10	10.8
O75348	V-type proton ATPase subunit G 1	2.71	0.081	2.8E-04	3	31.4
Q9BRA2	Thioredoxin domain-containing protein 17	2.68	0.243	3.2E-02	3	26.8
P13639	Elongation factor 2	2.67	0.074	1.5E-11	18	23.0
Q04760	Lactoylglutathione lyase	2.65	0.169	1.9E-03	3	14.7
P60842	Eukaryotic initiation factor 4A-I	2.63	0.093	3.8E-05	8	19.7
Q96DB5	Regulator of microtubule dynamics protein 1	2.61	0.147	1.1E-02	3	8.9
P17096	High mobility group protein HMG-I/HMG-Y	19.10	NA	NA	2	23.4
P14174	Macrophage migration inhibitory factor	11.59	NA	NA	2	17.4
P31939	Bifunctional purine biosynthesis protein PURH	6.26	NA	NA	2	3.4
P55769	NHP2-like protein 1	5.05	NA	NA	2	18.0
Q99848	Probable rRNA-processing protein EBP2	4.86	NA	NA	2	9.5
Q96G03	Phosphoglucomutase-2	4.13	NA	NA	2	4.1
O00625	Pirin	4.08	NA	NA	2	6.2
O15347	High mobility group protein B3	4.05	NA	NA	2	13.0
Q15819	Ubiquitin-conjugating enzyme E2 variant 2	3.87	0.200	2.1E-01	3	20.0
Q96C86	m7GpppX diphosphatase	3.76	NA	NA	2	8.3
Q16658	Fascin	3.67	NA	NA	2	5.3
P23588	Eukaryotic translation initiation factor 4B	3.42	NA	NA	2	4.7
Q53EL6	Programmed cell death protein 4	3.39	NA	NA	2	4.7
P17540	Creatine kinase S-type, mitochondrial	3.38	NA	NA	2	6.9
O75347	Tubulin-specific chaperone A	3.24	0.628	2.7E-01	3	26.9
Q13428	Treacle protein	3.19	NA	NA	2	2.2
P67809	Nuclease-sensitive element-binding protein 1	3.09	0.369	6.7E-02	3	14.2
P08621	U1 small nuclear ribonucleoprotein 70 kDa	3.02	NA	NA	2	4.3
P16152	Carbonyl reductase [NADPH] 1	3.01	NA	NA	2	8.7
P52566	Rho GDP-dissociation inhibitor 2	3.00	NA	NA	2	15.4
Q07021	Complement component 1 Q subcomponent-binding protein, mitochondrial	2.98	NA	NA	2	5.0
P14550	Alcohol dehydrogenase [NADP(+)]	2.93	NA	NA	2	5.5
Q9H1E3	Nuclear ubiquitous casein and cyclin-dependent kinase substrate 1	2.90	NA	NA	2	9.1
P58546	Myotrophin	2.88	NA	NA	2	16.9
Q13243	Serine/arginine-rich splicing factor 5	2.82	NA	NA	2	5.9
Q3LXA3	Bifunctional ATP-dependent dihydroxyacetone kinase/FAD-AMP lyase (cyclizing)	2.80	NA	NA	2	2.6
P13929	Beta-enolase	2.76	NA	NA	2	6.7
P12955	Xaa-Pro dipeptidase	2.74	0.336	1.2E-01	3	7.1
Q9UQ80	Proliferation-associated protein 2G4	2.73	NA	NA	2	5.1
O15533	Tapasin	2.69	NA	NA	2	4.2
P05141	ADP/ATP translocase 2	2.69	NA	NA	2	7.7
Q13442	28 kDa heat- and acid-stable phosphoprotein	2.66	0.257	2.1E-01	3	24.3
P06865	Beta-hexosaminidase subunit alpha	2.66	0.045	2.9E-01	3	4.9
P00441	Superoxide dismutase [Cu-Zn]	2.63	NA	NA	2	13.0
P53999	Activated RNA polymerase II transcriptional coactivator p15	2.60	NA	NA	2	15.7
P30085	UMP-CMP kinase	2.59	NA	NA	2	10.7
Q9NY12	H/ACA ribonucleoprotein complex subunit 1	2.59	NA	NA	2	7.4
Q9BUP0	EF-hand domain-containing protein D1	2.58	0.138	8.5E-02	3	8.8

P05387	60S acidic ribosomal protein P2	2.57	0.111	2.2E-04	4	53.9
O00567	Nucleolar protein 56	2.57	0.127	4.8E-04	6	9.3
P07910	Heterogeneous nuclear ribonucleoproteins C1/C2	2.55	0.093	1.1E-06	9	26.5
P57729	Ras-related protein Rab-38	2.52	0.226	7.7E-03	4	19.4
Q07955	Serine/arginine-rich splicing factor 1	2.50	0.103	6.1E-05	4	12.9
Q9NR56	Muscleblind-like protein 1	2.50	NA	NA	2	4.4
Q9NX24	H/ACA ribonucleoprotein complex subunit 2	2.49	NA	NA	2	19.0
Q8IVF2	Protein AHNAK2	2.48	0.212	1.9E-02	5	0.8
P10599	Thioredoxin	2.48	0.087	1.6E-06	4	39.0
P52565	Rho GDP-dissociation inhibitor 1	2.46	0.088	1.0E-03	4	22.5
P37837	Transaldolase	2.45	0.065	1.9E-05	8	23.1
P07900	Heat shock protein HSP 90-alpha	2.44	0.065	1.1E-05	12	14.5
P13693	Translationally-controlled tumor protein	2.44	NA	NA	2	15.7
P27695	DNA-(apurinic or apyrimidinic site) lyase	2.44	0.447	2.9E-01	3	8.5
P52209	6-phosphogluconate dehydrogenase, decarboxylating	2.42	0.168	1.1E-01	3	4.8
O76021	Ribosomal L1 domain-containing protein 1	2.41	0.155	4.3E-02	3	5.3
O94906	Pre-mRNA-processing factor 6	2.41	NA	NA	2	2.1
Q12905	Interleukin enhancer-binding factor 2	2.40	0.121	1.1E-04	6	20.3
P62826	GTP-binding nuclear protein Ran	2.39	NA	NA	2	13.9
Q13185	Chromobox protein homolog 3	2.38	0.114	6.8E-05	4	26.8
P22234	Multifunctional protein ADE2	2.38	NA	NA	2	4.5
Q9Y2Q3	Glutathione S-transferase kappa 1	2.35	0.111	2.7E-02	3	15.5
P50395	Rab GDP dissociation inhibitor beta	2.33	0.111	8.1E-03	6	16.9
P48681	Nestin	2.32	0.146	1.1E-03	6	4.7
Q13423	NAD(P) transhydrogenase, mitochondrial	2.32	0.064	9.5E-07	11	11.0
P26641	Elongation factor 1-gamma	2.30	0.143	3.2E-03	4	8.9
Q16698	2,4-dienoyl-CoA reductase, mitochondrial	2.30	0.150	3.7E-03	7	22.1
P30044	Peroxiredoxin-5, mitochondrial	2.30	0.103	7.7E-03	5	28.0
Q9Y3D0	Mitotic spindle-associated MMXD complex subunit MIP18	2.30	NA	NA	2	17.2
P27348	14-3-3 protein theta	2.27	0.136	3.0E-03	5	21.6
P62263	40S ribosomal protein S14	2.27	0.140	8.6E-02	3	29.8
P07602	Prosaposin	2.27	0.095	1.3E-04	7	10.5
O00154	Cytosolic acyl coenzyme A thioester hydrolase	2.27	0.420	1.7E-01	4	15.5
Q16629	Serine/arginine-rich splicing factor 7	2.26	0.141	6.9E-04	3	16.4
P61604	10 kDa heat shock protein, mitochondrial	2.26	0.052	1.4E-05	4	41.2
P46783	40S ribosomal protein S10	2.25	0.448	1.5E-01	3	20.0
P25398	40S ribosomal protein S12	2.24	0.133	1.0E-02	3	22.0
P29692	Elongation factor 1-delta	2.23	0.084	2.4E-02	4	16.0
Q1KMD3	Heterogeneous nuclear ribonucleoprotein U-like protein 2	2.22	0.076	6.9E-04	6	7.9
P25786	Proteasome subunit alpha type-1	2.21	0.091	4.0E-03	5	20.2
Q00688	Peptidyl-prolyl cis-trans isomerase FKBP3	2.20	0.559	3.1E-01	3	12.9
Q96C19	EF-hand domain-containing protein D2	2.20	0.036	1.4E-05	3	9.2
P46776	60S ribosomal protein L27a	2.19	NA	NA	2	14.2
Q99798	Aconitate hydratase, mitochondrial	2.18	0.117	6.6E-04	9	15.0
P62241	40S ribosomal protein S8	2.17	0.139	1.4E-02	4	14.9
P62829	60S ribosomal protein L23	2.17	0.072	1.2E-02	3	23.6
P84077	ADP-ribosylation factor 1	2.17	0.097	3.6E-04	3	16.0
P16188	HLA class I histocompatibility antigen, A-30 alpha chain	2.16	NA	NA	2	6.6
O43852	Calumenin	2.16	0.143	5.5E-03	8	32.7
P08574	Cytochrome c1, heme protein, mitochondrial	2.16	NA	NA	2	7.1
P20042	Eukaryotic translation initiation factor 2 subunit 2	2.15	0.236	6.1E-02	3	13.2
Q9UKV3	Apoptotic chromatin condensation inducer in the nucleus	2.14	NA	NA	2	1.7
Q86V81	THO complex subunit 4	2.13	NA	NA	2	8.2
Q8NFV4	Alpha/beta hydrolase domain-containing protein 11	2.12	NA	NA	2	8.6
P18621	60S ribosomal protein L17	2.12	0.235	2.0E-01	3	18.5
P15121	Aldose reductase	2.11	0.103	2.5E-03	5	17.1
P07093	Glia-derived nexin	2.09	NA	NA	2	6.5
P04075	Fructose-bisphosphate aldolase A	2.08	0.042	3.7E-06	13	42.9
P24821	Tenascin	2.08	0.212	1.6E-01	3	1.6
P40926	Malate dehydrogenase, mitochondrial	2.07	0.058	2.1E-08	12	41.4
P51608	Methyl-CpG-binding protein 2	2.06	0.281	5.0E-02	5	11.5
O00299	Chloride intracellular channel protein 1	2.06	0.167	1.4E-01	3	13.7
P55786	Puromycin-sensitive aminopeptidase	2.05	0.237	1.0E-01	3	3.6
P22314	Ubiquitin-like modifier-activating enzyme 1	2.05	0.160	2.9E-03	6	5.8
P12004	Proliferating cell nuclear antigen	2.04	NA	NA	2	8.4
O00410	Importin-5	2.03	NA	NA	2	1.7
Q9Y383	Putative RNA-binding protein Luc7-like 2	2.03	NA	NA	2	4.3
P37802	Transgelin-2	2.03	0.112	7.4E-04	4	21.6
P31146	Coronin-1A	2.02	0.164	2.9E-01	3	8.0
P63244	Guanine nucleotide-binding protein subunit beta-2-like 1	2.02	0.115	1.5E-03	6	16.7
P07339	Cathepsin D	2.02	0.064	4.5E-07	7	20.1
P09622	Dihydrolipoyl dehydrogenase, mitochondrial	2.01	0.090	1.0E-03	5	10.6
P30533	Alpha-2-macroglobulin receptor-associated protein	2.01	0.153	1.8E-02	5	13.7
P11766	Alcohol dehydrogenase class-3	2.00	NA	NA	2	6.4
O43598	2'-deoxynucleoside 5'-phosphate N-hydrolase 1	2.00	NA	NA	2	18.4
Q00839	Heterogeneous nuclear ribonucleoprotein U	2.00	0.069	2.5E-06	10	12.8
Q14683	Structural maintenance of chromosomes protein 1A	2.00	NA	NA	2	2.3
P42224	Signal transducer and activator of transcription 1-alpha/beta	2.00	0.446	1.8E-01	5	9.3
P62851	40S ribosomal protein S25	2.00	0.108	1.9E-03	4	24.0
O75083	WD repeat-containing protein 1	2.00	0.114	7.5E-04	3	6.8
Q9BVJ6	U3 small nucleolar RNA-associated protein 14 homolog A	1.99	NA	NA	2	4.3
P62424	60S ribosomal protein L7a	1.99	0.198	5.5E-02	3	11.7
Q92598	Heat shock protein 105 kDa	1.99	NA	NA	2	2.4
Q96AG4	Leucine-rich repeat-containing protein 59	1.98	0.077	1.2E-03	5	17.9
O60841	Eukaryotic translation initiation factor 5B	1.98	0.411	3.4E-01	3	3.0
Q9NVI7	ATPase family AAA domain-containing protein 3A	1.98	NA	NA	2	3.9
Q9Y4W6	AFG3-like protein 2	1.98	0.083	1.1E-03	5	5.4
Q92616	Translational activator GCN1	1.98	0.231	9.8E-02	3	1.6
P06753	Tropomyosin alpha-3 chain	1.98	0.118	2.9E-03	4	13.0
P30040	Endoplasmic reticulum resident protein 29	1.97	NA	NA	2	8.4
P16219	Short-chain specific acyl-CoA dehydrogenase, mitochondrial	1.96	NA	NA	2	4.6
P22626	Heterogeneous nuclear ribonucleoproteins A2/B1	1.95	0.069	3.7E-09	13	36.3
Q14103	Heterogeneous nuclear ribonucleoprotein D0	1.95	0.150	9.6E-02	4	15.5
P49207	60S ribosomal protein L34	1.94	0.222	9.0E-02	3	20.5
Q86UX7	Fermitin family homolog 3	1.94	0.290	8.7E-02	3	6.7
Q5JRX3	Presequence protease, mitochondrial	1.93	NA	NA	2	2.4
P37108	Signal recognition particle 14 kDa protein	1.92	0.092	5.5E-02	3	22.1
P18669	Phosphoglycerate mutase 1	1.92	0.073	1.5E-05	5	32.3
O43390	Heterogeneous nuclear ribonucleoprotein R	1.92	0.121	6.1E-04	8	15.0
P13797	Plastin-3	1.92	NA	NA	2	2.5
P23284	Peptidyl-prolyl cis-trans isomerase B	1.92	0.051	2.9E-10	13	51.4
Q9UHX1	Poly(U)-binding-splicing factor PUF60	1.92	0.089	7.8E-03	3	5.2
Q07020	60S ribosomal protein L18	1.91	0.091	3.7E-03	4	25.5
P60900	Proteasome subunit alpha type-6	1.91	NA	NA	2	8.5
P39019	40S ribosomal protein S19	1.91	0.051	3.6E-06	6	25.5
P07203	Glutathione peroxidase 1	1.91	NA	NA	2	10.3
P38646	Stress-70 protein, mitochondrial	1.90	0.090	3.6E-07	16	28.0
Q02818	Nucleobindin-1	1.90	0.072	8.6E-06	5	14.5
P62158	Calmodulin	1.90	0.079	6.0E-04	3	22.1
O75822	Eukaryotic translation initiation factor 3 subunit J	1.90	NA	NA	2	7.8
P08670	Vimentin	1.90	0.035	0.0E+00	31	58.4
P78527	DNA-dependent protein kinase catalytic subunit	1.89	0.054	1.0E-11	32	7.4
Q86UE4	Protein LYRIC	1.89	0.303	1.7E-01	4	9.5
P08865	40S ribosomal protein SA	1.87	0.059	2.3E-05	7	30.5
O75390	Citrate synthase, mitochondrial	1.87	0.055	1.9E-05	6	13.7

O75569	Interferon-inducible double-stranded RNA-dependent protein kinase activator A	1.85	NA	NA	2	5.8
P30084	Enoyl-CoA hydratase, mitochondrial	1.85	0.117	3.5E-01	3	12.8
P21796	Voltage-dependent anion-selective channel protein 1	1.85	0.131	3.0E-04	9	38.5
Q13510	Acid ceramidase	1.85	0.134	1.6E-02	4	8.1
Q08211	ATP-dependent RNA helicase A	1.85	0.167	2.1E-02	7	6.2
Q13435	Splicing factor 3B subunit 2	1.84	0.147	2.5E-02	5	7.2
P12956	X-ray repair cross-complementing protein 6	1.84	0.077	8.6E-06	16	26.4
P26599	Polypyrimidine tract-binding protein 1	1.84	0.123	3.3E-03	5	7.0
P02790	Hemopexin	1.84	0.125	4.0E-03	4	7.6
P24534	Elongation factor 1-beta	1.84	0.082	4.3E-03	4	16.4
Q96TC7	Regulator of microtubule dynamics protein 3	1.83	NA	NA	2	5.3
P23246	Splicing factor, proline- and glutamine-rich	1.83	0.116	5.4E-04	7	11.0
P11142	Heat shock cognate 71 kDa protein	1.83	0.053	2.4E-06	16	29.1
Q9NTZ6	RNA-binding protein 12	1.83	NA	NA	2	3.3
P13010	X-ray repair cross-complementing protein 5	1.83	0.093	4.8E-05	9	14.5
P19367	Hexokinase-1	1.82	0.061	1.3E-05	10	11.5
P07237	Protein disulfide-isomerase	1.82	0.057	9.6E-07	18	32.1
Q9GZT3	SRA stem-loop-interacting RNA-binding protein, mitochondrial	1.81	NA	NA	2	23.9
P09651	Heterogeneous nuclear ribonucleoprotein A1	1.81	0.059	2.6E-07	8	28.5
Q15424	Scaffold attachment factor B1	1.81	NA	NA	2	1.9
P20618	Proteasome subunit beta type-1	1.80	NA	NA	2	9.5
P49792	E3 SUMO-protein ligase RanBP2	1.80	0.174	6.8E-02	4	1.0
P17980	26S protease regulatory subunit 6A	1.80	0.589	1.8E-01	3	7.7
P35637	RNA-binding protein FUS	1.80	0.091	7.4E-03	4	7.6
P09525	Annexin A4	1.80	0.072	4.3E-06	11	30.7
Q9HD20	Manganese-transporting ATPase 13A1	1.80	NA	NA	2	2.0
Q9P2E9	Ribosome-binding protein 1	1.80	0.083	2.0E-04	8	6.7
P53597	Succinyl-CoA ligase [ADP/GDP-forming] subunit alpha, mitochondrial	1.80	0.049	2.9E-03	3	9.8
P55145	Mesencephalic astrocyte-derived neurotrophic factor	1.79	NA	NA	2	11.0
Q13263	Transcription intermediary factor 1-beta	1.79	0.092	3.1E-03	3	3.4
P38117	Electron transfer flavoprotein subunit beta	1.79	0.133	5.7E-02	5	20.0
P62140	Serine/threonine-protein phosphatase PP1-beta catalytic subunit	1.79	NA	NA	2	5.8
Q9NP81	Serine--tRNA ligase, mitochondrial	1.78	NA	NA	2	6.9
P26368	Splicing factor U2AF 65 kDa subunit	1.78	0.077	3.8E-02	3	7.6
Q99523	Sortilin	1.78	NA	NA	2	2.6
P35659	Protein DEK	1.78	NA	NA	2	6.1
P20810	Calpastatin	1.77	0.131	7.1E-02	6	12.4
Q14974	Importin subunit beta-1	1.77	0.087	1.1E-02	6	8.9
O15400	Syntaxin-7	1.77	0.081	1.3E-02	4	19.9
O14979	Heterogeneous nuclear ribonucleoprotein D-like	1.77	0.059	3.4E-02	3	5.0
O43143	Putative pre-mRNA-splicing factor ATP-dependent RNA helicase DHX15	1.77	0.073	1.7E-02	3	4.0
P48426	Phosphatidylinositol 5-phosphate 4-kinase type-2 alpha	1.77	NA	NA	2	6.4
P38159	RNA-binding motif protein, X chromosome	1.76	0.151	3.7E-02	4	11.0
Q13151	Heterogeneous nuclear ribonucleoprotein A0	1.76	0.054	1.6E-04	3	7.5
P13804	Electron transfer flavoprotein subunit alpha, mitochondrial	1.75	NA	NA	2	6.9
Q9ULV4	Coronin-1C	1.75	0.151	6.7E-02	3	6.5
P61254	60S ribosomal protein L26	1.75	NA	NA	2	15.9
Q15084	Protein disulfide-isomerase A6	1.75	0.052	3.2E-08	10	26.8
P78347	General transcription factor II-I	1.75	0.245	9.2E-02	4	4.0
P46940	Ras GTPase-activating-like protein IQGAP1	1.75	0.073	8.9E-05	13	9.7
P51991	Heterogeneous nuclear ribonucleoprotein A3	1.75	0.057	2.2E-06	9	28.6
P02763	Alpha-1-acid glycoprotein 1	1.75	0.106	1.4E-02	3	19.4
P40763	Signal transducer and activator of transcription 3	1.74	NA	NA	2	2.9
P28838	Cytosol aminopeptidase	1.74	0.028	4.2E-02	5	12.1
P30049	ATP synthase subunit delta, mitochondrial	1.73	NA	NA	2	13.7
P36578	60S ribosomal protein L4	1.73	0.208	1.4E-02	9	21.1
P83731	60S ribosomal protein L24	1.73	0.166	1.3E-01	4	22.3
P02765	Alpha-2-HS-glycoprotein	1.72	0.212	1.3E-01	3	7.6
Q01844	RNA-binding protein EWS	1.71	NA	NA	2	2.3
P39023	60S ribosomal protein L3	1.71	NA	NA	2	6.0
P42704	Leucine-rich PPR motif-containing protein, mitochondrial	1.71	0.395	1.1E-01	15	11.6
Q92688	Acidic leucine-rich nuclear phosphoprotein 32 family member B	1.71	0.237	2.8E-01	5	17.5
P27824	Calnexin	1.70	0.091	1.2E-03	12	24.7
P27635	60S ribosomal protein L10	1.70	0.132	3.4E-02	4	21.0
P30101	Protein disulfide-isomerase A3	1.70	0.042	3.2E-08	16	31.1
Q02878	60S ribosomal protein L6	1.70	0.084	7.7E-03	6	20.1
P54652	Heat shock-related 70 kDa protein 2	1.70	0.029	1.7E-04	6	11.7
P26373	60S ribosomal protein L13	1.70	0.085	5.6E-03	3	15.2
P48739	Phosphatidylinositol transfer protein beta isoform	1.70	NA	NA	2	5.5
P14854	Cytochrome c oxidase subunit 6B1	1.70	NA	NA	2	23.3
P55008	Allograft inflammatory factor 1	1.69	NA	NA	2	15.6
Q9Y5J7	Mitochondrial import inner membrane translocase subunit Tim9	1.68	NA	NA	2	25.8
Q92945	Far upstream element-binding protein 2	1.68	0.140	1.7E-01	8	16.5
P34932	Heat shock 70 kDa protein 4	1.67	0.171	3.7E-02	4	4.5
P09668	Pro-cathepsin H	1.67	NA	NA	2	7.8
Q00059	Transcription factor A, mitochondrial	1.67	0.102	7.8E-03	5	16.7
Q13617	Cullin-2	1.66	NA	NA	2	2.0
P25788	Proteasome subunit alpha type-3	1.66	NA	NA	2	4.7
P31949	Protein S100-A11	1.66	NA	NA	2	23.8
P62136	Serine/threonine-protein phosphatase PP1-alpha catalytic subunit	1.65	NA	NA	2	4.2
P62906	60S ribosomal protein L10a	1.65	0.076	4.4E-04	6	26.3
O95202	LETM1 and EF-hand domain-containing protein 1, mitochondrial	1.65	0.175	1.4E-01	6	9.1
P63279	SUMO-conjugating enzyme UBC9	1.65	NA	NA	2	14.6
Q9BZZ5	Apoptosis inhibitor 5	1.65	0.087	7.3E-03	4	7.6
Q15365	Poly(rC)-binding protein 1	1.65	0.121	3.3E-03	3	12.1
P46782	40S ribosomal protein S5	1.64	NA	NA	2	8.3
O14818	Proteasome subunit alpha type-7	1.64	0.231	1.8E-01	3	11.7
Q9NR28	Diablo homolog, mitochondrial	1.64	NA	NA	2	8.8
Q08AM6	Protein VAC14 homolog	1.62	NA	NA	2	3.1
Q16666	Gamma-interferon-inducible protein 16	1.62	0.204	1.1E-01	3	3.7
P30050	60S ribosomal protein L12	1.61	0.155	3.4E-01	4	35.8
P49755	Transmembrane emp24 domain-containing protein 10	1.60	0.381	1.5E-01	4	24.2
P28161	Glutathione S-transferase Mu 2	1.60	0.071	1.5E-03	4	13.8
P30048	Thioredoxin-dependent peroxide reductase, mitochondrial	1.60	0.053	2.7E-05	5	24.2
O43681	ATPase ASNA1	1.60	0.128	4.7E-01	3	10.1
Q15046	Lysine--tRNA ligase	1.60	0.114	4.0E-02	4	6.5
P60866	40S ribosomal protein S20	1.59	NA	NA	2	19.3
Q9NX63	Coiled-coil-helix-coiled-coil-helix domain-containing protein 3, mitochondrial	1.59	NA	NA	2	9.3
P55809	Succinyl-CoA:3-ketoacid coenzyme A transferase 1, mitochondrial	1.59	0.123	1.1E-01	4	5.0
P62258	14-3-3 protein epsilon	1.59	0.083	1.5E-03	8	27.1
Q9Y3D9	28S ribosomal protein S23, mitochondrial	1.59	NA	NA	2	10.5
P26640	Valine--tRNA ligase	1.58	0.068	1.8E-02	5	4.1
Q9H2G2	STE20-like serine/threonine-protein kinase	1.58	NA	NA	2	1.9
P61978	Heterogeneous nuclear ribonucleoprotein K	1.57	0.085	2.9E-04	13	32.6
O43488	Aflatoxin B1 aldehyde reductase member 2	1.57	NA	NA	2	5.3
P49411	Elongation factor Tu, mitochondrial	1.57	0.083	4.1E-03	12	30.5
Q14978	Nucleolar and coiled-body phosphoprotein 1	1.57	NA	NA	2	3.9
O15212	Prefoldin subunit 6	1.56	NA	NA	2	11.6
Q9UKM9	RNA-binding protein Raly	1.56	0.122	1.9E-02	3	13.7
P08195	4F2 cell-surface antigen heavy chain	1.56	0.089	2.1E-04	7	14.9
Q07666	KH domain-containing, RNA-binding, signal transduction-associated protein 1	1.56	0.084	9.6E-03	5	9.3
P07814	Bifunctional glutamate/proline--tRNA ligase	1.56	0.103	6.5E-02	3	2.7
O75874	Isocitrate dehydrogenase [NADP] cytoplasmic	1.55	NA	NA	2	4.6
Q15029	116 kDa U5 small nuclear ribonucleoprotein component	1.54	0.148	2.2E-01	3	3.4
P17844	Probable ATP-dependent RNA helicase DDX5	1.54	0.120	5.5E-02	4	6.2

Q8IZQ5	Selenoprotein H	1.54	NA	NA	2	17.2
Q86UP2	Kinectin	1.53	0.162	1.8E-02	6	5.9
P49756	RNA-binding protein 25	1.53	NA	NA	2	2.6
P39687	Acidic leucine-rich nuclear phosphoprotein 32 family member A	1.52	0.173	7.5E-02	4	14.5
Q14152	Eukaryotic translation initiation factor 3 subunit A	1.52	0.248	6.8E-02	7	5.9
O00571	ATP-dependent RNA helicase DDX3X	1.52	0.571	2.7E-01	3	5.4
P46777	60S ribosomal protein L5	1.52	0.082	4.3E-04	5	18.9
P05388	60S acidic ribosomal protein P0	1.51	0.137	3.3E-02	5	13.9
Q5SSJ5	Heterochromatin protein 1-binding protein 3	1.51	0.088	2.6E-03	4	7.4
P18859	ATP synthase-coupling factor 6, mitochondrial	1.51	NA	NA	2	13.0
P46779	60S ribosomal protein L28	1.51	0.098	5.8E-02	3	19.0
Q15293	Reticulocalbin-1	1.50	NA	NA	2	4.5
P61247	40S ribosomal protein S3a	1.50	0.087	1.5E-02	8	29.2
P52597	Heterogeneous nuclear ribonucleoprotein F	1.50	0.155	2.7E-01	3	7.0
Q9Y4I1	Unconventional myosin-Va	1.49	NA	NA	2	1.0
P62249	40S ribosomal protein S16	1.49	0.051	1.4E-03	5	35.6
Q8IUX7	Adipocyte enhancer-binding protein 1	1.49	0.135	2.8E-02	4	5.4
P28066	Proteasome subunit alpha type-5	1.48	NA	NA	2	15.8
P82909	28S ribosomal protein S36, mitochondrial	1.48	NA	NA	2	25.2
P10809	60 kDa heat shock protein, mitochondrial	1.48	0.115	1.7E-02	14	27.1
O75165	DnaJ homolog subfamily C member 13	1.48	NA	NA	2	0.9
Q16836	Hydroxyacyl-coenzyme A dehydrogenase, mitochondrial	1.47	NA	NA	2	5.7
Q9H2U2	Inorganic pyrophosphatase 2, mitochondrial	1.47	NA	NA	2	6.6
P62701	40S ribosomal protein S4, X isoform	1.46	0.287	1.9E-01	3	9.9
P14866	Heterogeneous nuclear ribonucleoprotein L	1.46	0.091	7.5E-03	9	22.2
P08237	ATP-dependent 6-phosphofructokinase, muscle type	1.46	NA	NA	2	2.8
P09661	U2 small nuclear ribonucleoprotein A'	1.46	NA	NA	2	8.6
P36955	Pigment epithelium-derived factor	1.45	0.291	2.9E-01	6	15.6
P52943	Cysteine-rich protein 2	1.45	NA	NA	2	18.8
O15145	Actin-related protein 2/3 complex subunit 3	1.45	NA	NA	2	12.4
P12270	Nucleoprotein TPR	1.45	0.183	1.8E-01	3	2.0
Q92841	Probable ATP-dependent RNA helicase DDX17	1.44	0.228	1.5E-01	3	3.8
P09012	U1 small nuclear ribonucleoprotein A	1.44	0.080	1.7E-01	3	9.9
Q9Y277	Voltage-dependent anion-selective channel protein 3	1.44	1.830	1.6E-01	4	14.8
P27797	Calreticulin	1.44	0.134	4.6E-02	9	26.6
Q96AE4	Far upstream element-binding protein 1	1.44	0.015	2.1E-01	5	7.8
Q9Y4L1	Hypoxia up-regulated protein 1	1.44	0.075	2.4E-02	7	8.4
Q15942	Zyxin	1.44	NA	NA	2	5.4
P84103	Serine/arginine-rich splicing factor 3	1.44	0.108	1.5E-02	3	20.7
O60506	Heterogeneous nuclear ribonucleoprotein Q	1.43	0.252	9.0E-02	4	5.6
P15880	40S ribosomal protein S2	1.43	0.117	4.6E-03	5	21.2
Q8NBJ5	Procollagen galactosyltransferase 1	1.43	NA	NA	2	3.4
P62280	40S ribosomal protein S11	1.42	0.033	1.3E-02	3	16.5
P08236	Beta-glucuronidase	1.42	NA	NA	2	4.0
Q86U42	Polyadenylate-binding protein 2	1.42	0.561	3.5E-01	3	8.8
P02774	Vitamin D-binding protein	1.42	0.107	5.0E-02	5	8.2
P35232	Prohibitin	1.42	0.048	7.5E-06	9	34.6
Q86VP6	Cullin-associated NEDD8-dissociated protein 1	1.41	0.252	1.6E-01	4	2.9
P52272	Heterogeneous nuclear ribonucleoprotein M	1.41	0.084	3.1E-03	13	19.0
P14314	Glucosidase 2 subunit beta	1.41	0.182	1.1E-02	8	12.1
Q99623	Prohibitin-2	1.41	0.055	2.7E-02	7	24.7
P11137	Microtubule-associated protein 2	1.41	NA	NA	2	1.1
Q6UVK1	Chondroitin sulfate proteoglycan 4	1.40	0.148	2.3E-01	3	2.2
O75367	Core histone macro-H2A.1	1.40	0.119	7.7E-03	9	24.7
P09211	Glutathione S-transferase P	1.40	0.347	4.4E-01	7	39.0
Q02978	Mitochondrial 2-oxoglutarate/malate carrier protein	1.40	NA	NA	2	5.4
P01859	Ig gamma-2 chain C region	1.40	0.251	3.3E-01	3	12.0
Q7KZF4	Staphylococcal nuclease domain-containing protein 1	1.40	NA	NA	2	2.7
Q9BWM7	Sideroflexin-3	1.40	NA	NA	2	8.3
P17931	Galectin-3	1.39	0.050	5.8E-06	6	28.4
P61769	Beta-2-microglobulin	1.39	NA	NA	2	16.8
O00231	26S proteasome non-ATPase regulatory subunit 11	1.39	NA	NA	2	4.5
O75352	Mannose-P-dolichol utilization defect 1 protein	1.38	NA	NA	2	6.1
P62820	Ras-related protein Rab-1A	1.38	NA	NA	2	11.7
O14773	Tripeptidyl-peptidase 1	1.38	0.029	9.8E-07	4	8.7
P06576	ATP synthase subunit beta, mitochondrial	1.38	0.049	1.6E-06	17	35.9
P55084	Trifunctional enzyme subunit beta, mitochondrial	1.38	0.040	5.7E-06	11	19.4
P50914	60S ribosomal protein L14	1.37	NA	NA	2	10.7
Q8N5K1	CDGSH iron-sulfur domain-containing protein 2	1.37	0.144	1.9E-01	4	31.9
Q15907	Ras-related protein Rab-11B	1.37	NA	NA	2	8.7
P09874	Poly [ADP-ribose] polymerase 1	1.37	0.283	1.6E-01	5	5.2
P50225	Sulfotransferase 1A1	1.37	NA	NA	2	7.1
Q9BUJ2	Heterogeneous nuclear ribonucleoprotein U-like protein 1	1.36	NA	NA	2	3.4
Q8NBS9	Thioredoxin domain-containing protein 5	1.36	0.087	7.2E-02	6	14.8
P19971	Thymidine phosphorylase	1.36	0.243	2.6E-01	6	16.2
P62277	40S ribosomal protein S13	1.36	0.246	8.4E-02	5	24.5
Q15717	ELAV-like protein 1	1.36	NA	NA	2	7.7
Q12904	Aminoacyl tRNA synthase complex-interacting multifunctional protein 1	1.36	NA	NA	2	9.6
P50454	Serpin H1	1.35	0.100	5.5E-02	5	13.2
P41091	Eukaryotic translation initiation factor 2 subunit 3	1.35	NA	NA	2	6.1
P48735	Isocitrate dehydrogenase [NADP], mitochondrial	1.35	0.217	9.4E-02	8	18.6
P49189	4-trimethylaminobutyraldehyde dehydrogenase	1.34	NA	NA	2	3.6
Q96KP4	Cytosolic non-specific dipeptidase	1.34	0.112	1.0E-01	5	13.1
P30740	Leukocyte elastase inhibitor	1.34	0.092	7.3E-02	4	12.1
Q14677	Clathrin interactor 1	1.34	0.839	5.7E-01	3	6.6
Q96PK6	RNA-binding protein 14	1.34	NA	NA	2	3.4
P54136	Arginine--tRNA ligase, cytoplasmic	1.34	0.207	7.5E-02	4	6.7
P62318	Small nuclear ribonucleoprotein Sm D3	1.33	NA	NA	2	15.1
Q99714	3-hydroxyacyl-CoA dehydrogenase type-2	1.33	0.147	1.4E-01	3	11.5
P0CW22	40S ribosomal protein S17-like	1.33	0.116	2.0E-01	3	16.3
Q15459	Splicing factor 3A subunit 1	1.33	NA	NA	2	2.4
P51810	G-protein coupled receptor 143	1.33	NA	NA	2	5.7
Q9Y6C9	Mitochondrial carrier homolog 2	1.33	0.106	1.1E-01	4	19.5
P62917	60S ribosomal protein L8	1.33	NA	NA	2	10.5
Q14247	Src substrate cortactin	1.32	0.095	4.1E-01	5	11.8
O75947	ATP synthase subunit d, mitochondrial	1.32	0.101	2.2E-02	8	44.7
P36543	V-type proton ATPase subunit E 1	1.32	NA	NA	2	7.1
P01009	Alpha-1-antitrypsin	1.31	0.109	3.5E-02	14	32.5
P49748	Very long-chain specific acyl-CoA dehydrogenase, mitochondrial	1.31	0.118	3.7E-02	8	13.4
P23368	NAD-dependent malic enzyme, mitochondrial	1.31	NA	NA	2	2.4
P48047	ATP synthase subunit O, mitochondrial	1.31	0.061	7.6E-03	4	25.4
O95831	Apoptosis-inducing factor 1, mitochondrial	1.31	NA	NA	2	5.2
P06730	Eukaryotic translation initiation factor 4E	1.30	0.763	6.1E-01	3	14.7
P18124	60S ribosomal protein L7	1.30	0.123	3.3E-02	7	24.6
P52907	F-actin-capping protein subunit alpha-1	1.30	NA	NA	2	13.3
P62987	Ubiquitin-60S ribosomal protein L40	1.30	0.089	6.4E-02	9	50.8
P62913	60S ribosomal protein L11	1.29	NA	NA	2	11.8
Q9NSE4	Isoleucine--tRNA ligase, mitochondrial	1.29	0.161	1.5E-01	4	4.9
Q8NB37	Parkinson disease 7 domain-containing protein 1	1.29	NA	NA	2	13.6
Q9UJU6	Drebrin-like protein	1.29	NA	NA	2	6.0
Q9Y3Z3	Deoxynucleoside triphosphate triphosphohydrolase SAMHD1	1.29	0.171	1.2E-01	3	5.3
P49591	Serine--tRNA ligase, cytoplasmic	1.28	0.086	4.9E-02	4	8.6
O43809	Cleavage and polyadenylation specificity factor subunit 5	1.28	NA	NA	2	10.6
Q16891	Mitochondrial inner membrane protein	1.28	0.108	2.7E-02	12	20.3
O75396	Vesicle-trafficking protein SEC22b	1.28	0.103	3.7E-02	3	18.1

P46778	60S ribosomal protein L21	1.27	NA	NA	2	13.8
P67936	Tropomyosin alpha-4 chain	1.27	0.226	8.8E-02	10	33.9
Q07960	Rho GTPase-activating protein 1	1.27	NA	NA	2	5.5
Q13596	Sorting nexin-1	1.27	NA	NA	2	5.2
Q02543	60S ribosomal protein L18a	1.26	1.319	5.6E-01	3	17.6
P62269	40S ribosomal protein S18	1.26	0.024	1.3E-02	5	28.3
Q9Y3U8	60S ribosomal protein L36	1.26	0.128	9.7E-02	4	30.5
Q07000	HLA class I histocompatibility antigen, Cw-15 alpha chain	1.25	NA	NA	2	9.6
P45880	Voltage-dependent anion-selective channel protein 2	1.25	0.075	2.1E-02	7	24.5
P80303	Nucleobindin-2	1.25	NA	NA	2	6.0
O00151	PDZ and LIM domain protein 1	1.25	0.159	2.2E-01	3	10.0
P40939	Trifunctional enzyme subunit alpha, mitochondrial	1.24	0.263	2.1E-01	16	24.1
A1L0T0	Acetolactate synthase-like protein	1.24	0.073	1.9E-01	3	5.5
P59998	Actin-related protein 2/3 complex subunit 4	1.24	0.116	1.2E-01	3	16.1
O75643	U5 small nuclear ribonucleoprotein 200 kDa helicase	1.24	NA	NA	2	0.9
O15144	Actin-related protein 2/3 complex subunit 2	1.24	0.099	8.8E-02	5	14.3
Q15233	Non-POU domain-containing octamer-binding protein	1.24	0.155	3.6E-01	6	13.6
Q15582	Transforming growth factor-beta-induced protein ig-h3	1.24	0.061	7.8E-03	9	15.5
P25705	ATP synthase subunit alpha, mitochondrial	1.23	0.106	1.8E-02	20	39.1
P78344	Eukaryotic translation initiation factor 4 gamma 2	1.23	NA	NA	2	1.5
P11940	Polyadenylate-binding protein 1	1.23	0.076	9.0E-03	7	13.8
P46459	Vesicle-fusing ATPase	1.21	0.192	2.8E-01	5	7.3
P07858	Cathepsin B	1.21	0.075	4.0E-01	4	13.6
P14868	Aspartate--tRNA ligase, cytoplasmic	1.21	0.038	1.9E-02	3	8.4
Q9Y2W1	Thyroid hormone receptor-associated protein 3	1.21	NA	NA	2	2.7
Q9Y512	Sorting and assembly machinery component 50 homolog	1.21	NA	NA	2	4.7
Q15005	Signal peptidase complex subunit 2	1.21	NA	NA	2	12.8
Q99584	Protein S100-A13	1.20	0.195	2.2E-01	3	22.4
Q13011	Delta(3,5)-Delta(2,4)-dienoyl-CoA isomerase, mitochondrial	1.20	0.180	1.5E-01	3	7.6
Q9BR76	Coronin-1B	1.20	NA	NA	2	3.5
Q14157	Ubiquitin-associated protein 2-like	1.20	NA	NA	2	2.5
P14927	Cytochrome b-c1 complex subunit 7	1.20	0.407	3.6E-01	3	27.0
P61353	60S ribosomal protein L27	1.19	0.990	2.7E-01	4	31.6
P22695	Cytochrome b-c1 complex subunit 2, mitochondrial	1.18	0.294	1.4E-01	5	17.4
P26885	Peptidyl-prolyl cis-trans isomerase FKBP2	1.18	NA	NA	2	9.2
P36542	ATP synthase subunit gamma, mitochondrial	1.18	0.195	3.5E-01	3	11.1
P27816	Microtubule-associated protein 4	1.18	0.844	3.3E-01	7	8.3
P49458	Signal recognition particle 9 kDa protein	1.18	0.169	5.1E-01	3	34.9
Q09028	Histone-binding protein RBBP4	1.18	0.218	3.9E-01	3	5.4
Q96AY3	Peptidyl-prolyl cis-trans isomerase FKBP10	1.18	NA	NA	2	3.6
P22307	Non-specific lipid-transfer protein	1.17	0.140	2.6E-01	4	6.2
Q15063	Periostin	1.17	NA	NA	2	4.1
O75533	Splicing factor 3B subunit 1	1.17	0.368	7.1E-01	4	3.8
P10644	cAMP-dependent protein kinase type I-alpha regulatory subunit	1.17	0.199	2.4E-01	5	13.4
Q9UPN3	Microtubule-actin cross-linking factor 1, isoforms 1/2/3/5	1.16	NA	NA	2	0.4
P32969	60S ribosomal protein L9	1.16	NA	NA	2	5.7
P51970	NADH dehydrogenase [ubiquinone] 1 alpha subcomplex subunit 8	1.15	NA	NA	2	7.6
Q00325	Phosphate carrier protein, mitochondrial	1.15	0.099	2.3E-01	8	21.3
P11387	DNA topoisomerase 1	1.15	NA	NA	2	2.1
Q00765	Receptor expression-enhancing protein 5	1.14	NA	NA	2	10.1
Q12913	Receptor-type tyrosine-protein phosphatase eta	1.14	NA	NA	2	1.9
O00487	26S proteasome non-ATPase regulatory subunit 14	1.14	NA	NA	2	6.5
P46926	Glucosamine-6-phosphate isomerase 1	1.14	NA	NA	2	5.9
O75380	NADH dehydrogenase [ubiquinone] iron-sulfur protein 6, mitochondrial	1.14	NA	NA	2	20.2
Q04637	Eukaryotic translation initiation factor 4 gamma 1	1.14	NA	NA	2	1.0
P50213	Isocitrate dehydrogenase [NAD] subunit alpha, mitochondrial	1.13	0.132	1.9E-01	3	10.1
Q5VTE0	Putative elongation factor 1-alpha-like 3	1.13	NA	NA	2	4.1
Q14165	Malectin	1.13	NA	NA	2	9.2
Q06830	Peroxiredoxin-1	1.13	0.147	2.1E-01	8	47.7
P11021	78 kDa glucose-regulated protein	1.13	0.064	5.7E-02	25	38.5
P30519	Heme oxygenase 2	1.13	NA	NA	2	7.0
P30041	Peroxiredoxin-6	1.12	0.098	6.6E-02	9	37.9
Q05682	Caldesmon	1.12	0.243	3.6E-01	7	11.5
Q6P2Q9	Pre-mRNA-processing-splicing factor 8	1.12	0.548	4.8E-01	5	2.2
P38606	V-type proton ATPase catalytic subunit A	1.12	1.724	3.5E-01	5	8.8
P01023	Alpha-2-macroglobulin	1.12	41.257	3.6E-01	9	8.5
P43307	Translocon-associated protein subunit alpha	1.12	NA	NA	2	6.6
P09382	Galectin-1	1.12	0.075	1.3E-01	5	43.7
O95881	Thioredoxin domain-containing protein 12	1.11	NA	NA	2	14.0
P00734	Prothrombin	1.11	NA	NA	2	4.0
P53618	Coatomer subunit beta	1.11	1.352	5.1E-01	4	5.5
Q9NVS9	Pyridoxine-5'-phosphate oxidase	1.11	NA	NA	2	13.0
Q9Y262	Eukaryotic translation initiation factor 3 subunit L	1.11	0.220	6.3E-01	5	8.3
Q13547	Histone deacetylase 1	1.11	0.454	8.5E-01	3	6.0
P23396	40S ribosomal protein S3	1.11	0.119	3.0E-01	10	43.2
P61758	Prefoldin subunit 3	1.10	NA	NA	2	8.6
P02452	Collagen alpha-1(I) chain	1.10	4.760	9.0E-01	4	3.9
Q16531	DNA damage-binding protein 1	1.10	1.060	6.6E-01	4	3.4
Q14980	Nuclear mitotic apparatus protein 1	1.09	0.689	6.3E-01	8	5.0
P11586	C-1-tetrahydrofolate synthase, cytoplasmic	1.09	0.260	4.9E-01	5	4.9
P10515	Dihydrolipoyllysine-residue acetyltransferase component of pyruvate dehydrogenase complex, mitochondrial	1.09	0.041	1.2E-01	6	9.0
P78371	T-complex protein 1 subunit beta	1.09	NA	NA	2	4.3
P11216	Glycogen phosphorylase, brain form	1.09	0.390	2.8E-01	7	10.4
P13674	Prolyl 4-hydroxylase subunit alpha-1	1.09	0.319	7.7E-01	3	7.3
O00560	Syntenin-1	1.08	0.331	7.8E-01	3	13.4
Q8N1G4	Leucine-rich repeat-containing protein 47	1.07	NA	NA	2	4.6
O43837	Isocitrate dehydrogenase [NAD] subunit beta, mitochondrial	1.07	NA	NA	2	5.5
P36957	Dihydrolipoyllysine-residue succinyltransferase component of 2-oxoglutarate dehydrogenase complex, mitochondrial	1.07	1.425	7.0E-01	6	14.8
P02461	Collagen alpha-1(III) chain	1.06	0.858	5.9E-01	3	2.9
P46781	40S ribosomal protein S9	1.06	0.172	6.6E-01	7	23.2
P50402	Emerin	1.06	NA	NA	2	9.8
Q16851	UTP--glucose-1-phosphate uridylyltransferase	1.06	NA	NA	2	4.5
P30153	Serine/threonine-protein phosphatase 2A 65 kDa regulatory subunit A alpha isoform	1.06	NA	NA	2	3.6
Q9NZ08	Endoplasmic reticulum aminopeptidase 1	1.06	0.848	8.2E-01	3	3.2
P16435	NADPH--cytochrome P450 reductase	1.05	0.186	5.8E-01	3	5.5
P61158	Actin-related protein 3	1.05	0.269	5.1E-01	7	22.7
P62753	40S ribosomal protein S6	1.05	0.143	7.5E-01	3	14.1
P09496	Clathrin light chain A	1.05	0.474	8.4E-01	3	8.9
Q9UNH7	Sorting nexin-6	1.05	NA	NA	2	3.2
P11177	Pyruvate dehydrogenase E1 component subunit beta, mitochondrial	1.05	0.282	7.5E-01	3	8.6
Q16270	Insulin-like growth factor-binding protein 7	1.05	NA	NA	2	10.6
Q13283	Ras GTPase-activating protein-binding protein 1	1.05	NA	NA	2	7.3
B5ME19	Eukaryotic translation initiation factor 3 subunit C-like protein	1.04	0.200	8.0E-01	4	4.7
P21266	Glutathione S-transferase Mu 3	1.04	0.400	6.6E-01	3	13.8
Q9HC38	Glyoxalase domain-containing protein 4	1.04	0.863	9.5E-01	3	9.6
Q13200	26S proteasome non-ATPase regulatory subunit 2	1.03	0.258	7.0E-01	3	4.4
O95292	Vesicle-associated membrane protein-associated protein B/C	1.03	1.383	8.7E-01	4	21.0
P31930	Cytochrome b-c1 complex subunit 1, mitochondrial	1.03	0.084	6.4E-01	5	14.8
Q02218	2-oxoglutarate dehydrogenase, mitochondrial	1.02	149.065	8.2E-01	10	10.6
Q6NUM9	All-trans-retinol 13,14-reductase	1.02	NA	NA	2	4.3
P49721	Proteasome subunit beta type-2	1.02	NA	NA	2	13.4
P62244	40S ribosomal protein S15a	1.02	0.050	6.1E-01	3	18.5
P09497	Clathrin light chain B	1.02	NA	NA	2	7.4
P61160	Actin-related protein 2	1.02	0.393	8.6E-01	4	14.2
Q14697	Neutral alpha-glucosidase AB	1.02	3.619	8.5E-01	14	15.1

Q9Y6M9	NADH dehydrogenase [ubiquinone] 1 beta subcomplex subunit 9	1.01	NA	NA	2	15.6
P13667	Protein disulfide-isomerase A4	1.01	0.578	9.0E-01	9	13.3
Q07065	Cytoskeleton-associated protein 4	1.01	0.314	8.6E-01	8	17.1
Q9NV70	Exocyst complex component 1	1.01	NA	NA	2	2.1
O15143	Actin-related protein 2/3 complex subunit 1B	1.01	0.716	9.2E-01	4	13.4
P30626	Sorcin	1.01	NA	NA	2	9.1
Q16610	Extracellular matrix protein 1	1.00	0.899	9.8E-01	4	8.9
P40429	60S ribosomal protein L13a	1.00	0.744	9.9E-01	4	14.8
P31040	Succinate dehydrogenase [ubiquinone] flavoprotein subunit, mitochondrial	1.00	0.170	9.6E-01	6	11.9
P61421	V-type proton ATPase subunit d 1	0.99	1.077	8.8E-01	3	7.7
P20700	Lamin-B1	0.98	11.582	9.0E-01	8	14.5
P02647	Apolipoprotein A-I	0.98	0.594	8.4E-01	11	46.1
P02545	Prelamin-A/C	0.98	1.551	6.0E-01	37	51.4
P14625	Endoplasmin	0.98	1.268	7.3E-01	18	22.4
P35613	Basigin	0.97	0.445	9.0E-01	3	12.7
P07305	Histone H1.0	0.97	NA	NA	2	10.8
O60271	C-Jun-amino-terminal kinase-interacting protein 4	0.97	NA	NA	2	2.3
P67870	Casein kinase II subunit beta	0.96	NA	NA	2	10.2
O00303	Eukaryotic translation initiation factor 3 subunit F	0.96	NA	NA	2	8.1
P08134	Rho-related GTP-binding protein RhoC	0.96	0.208	4.8E-01	3	10.4
P50502	Hsc70-interacting protein	0.96	1.733	6.3E-01	3	6.5
P51149	Ras-related protein Rab-7a	0.96	1.937	7.7E-01	4	18.8
P21281	V-type proton ATPase subunit B, brain isoform	0.95	1.117	7.9E-01	5	12.3
P63000	Ras-related C3 botulinum toxin substrate 1	0.95	0.038	3.4E-01	4	24.5
P53621	Coatomer subunit alpha	0.95	0.208	7.8E-01	7	6.9
Q9P2R7	Succinyl-CoA ligase [ADP-forming] subunit beta, mitochondrial	0.95	0.094	5.2E-01	3	5.8
P21912	Succinate dehydrogenase [ubiquinone] iron-sulfur subunit, mitochondrial	0.95	0.057	3.8E-01	5	18.9
P60981	Destrin	0.95	NA	NA	2	10.3
Q27J81	Inverted formin-2	0.94	NA	NA	2	1.8
P26196	Probable ATP-dependent RNA helicase DDX6	0.94	NA	NA	2	4.1
P24752	Acetyl-CoA acetyltransferase, mitochondrial	0.94	0.130	5.7E-01	3	4.9
P26038	Moesin	0.94	0.088	2.4E-01	9	15.3
P0C0S8	Histone H2A type 1	0.93	NA	NA	2	25.4
P01857	Ig gamma-1 chain C region	0.93	0.124	1.9E-01	6	31.5
P54727	UV excision repair protein RAD23 homolog B	0.93	0.410	6.2E-01	3	7.3
P51572	B-cell receptor-associated protein 31	0.92	0.366	3.3E-01	6	26.0
P27708	CAD protein	0.92	0.282	8.6E-01	5	2.4
P29590	Protein PML	0.92	NA	NA	2	2.7
O75964	ATP synthase subunit g, mitochondrial	0.92	0.115	2.2E-01	3	35.9
P21964	Catechol O-methyltransferase	0.92	NA	NA	2	8.9
Q9UHQ9	NADH-cytochrome b5 reductase 1	0.92	0.155	3.7E-01	3	10.5
Q15691	Microtubule-associated protein RP/EB family member 1	0.92	0.013	3.4E-02	3	12.7
O75914	Serine/threonine-protein kinase PAK 3	0.91	NA	NA	2	3.6
P17655	Calpain-2 catalytic subunit	0.91	0.134	5.9E-01	6	6.1
Q9P0J0	NADH dehydrogenase [ubiquinone] 1 alpha subcomplex subunit 13	0.91	NA	NA	2	18.1
P40227	T-complex protein 1 subunit zeta	0.90	0.292	4.7E-01	5	11.1
P61026	Ras-related protein Rab-10	0.90	NA	NA	2	9.5
Q9UHD8	Septin-9	0.90	0.118	3.5E-01	4	7.2
Q99733	Nucleosome assembly protein 1-like 4	0.90	NA	NA	2	6.1
P49419	Alpha-aminoadipic semialdehyde dehydrogenase	0.89	NA	NA	2	4.3
Q9H223	EH domain-containing protein 4	0.89	0.177	6.9E-01	3	5.7
P35998	26S protease regulatory subunit 7	0.89	0.480	6.1E-01	3	8.5
Q8NBJ7	Sulfatase-modifying factor 2	0.89	NA	NA	2	6.3
Q01518	Adenylyl cyclase-associated protein 1	0.89	NA	NA	2	5.1
Q8WXH0	Nesprin-2	0.89	5.420	6.6E-01	4	0.4
O60313	Dynamin-like 120 kDa protein, mitochondrial	0.89	0.222	6.4E-01	3	4.9
P29966	Myristoylated alanine-rich C-kinase substrate	0.88	0.899	7.4E-01	3	13.0
Q99829	Copine-1	0.88	NA	NA	2	4.7
P04179	Superoxide dismutase [Mn], mitochondrial	0.88	0.484	4.0E-01	7	40.5
Q3ZCQ8	Mitochondrial import inner membrane translocase subunit TIM50	0.87	NA	NA	2	5.1
P42765	3-ketoacyl-CoA thiolase, mitochondrial	0.87	0.036	3.1E-02	3	6.5
Q16718	NADH dehydrogenase [ubiquinone] 1 alpha subcomplex subunit 5	0.85	NA	NA	2	22.4
Q15149	Plectin	0.85	0.064	6.3E-04	55	12.7
Q13561	Dynactin subunit 2	0.84	0.190	3.9E-01	5	12.0
Q14108	Lysosome membrane protein 2	0.83	0.145	2.1E-01	4	9.8
Q93050	V-type proton ATPase 116 kDa subunit a isoform 1	0.83	NA	NA	2	2.0
O94979	Protein transport protein Sec31A	0.83	0.366	5.7E-01	3	3.3
P10606	Cytochrome c oxidase subunit 5B, mitochondrial	0.83	0.082	9.1E-02	3	24.0
P11279	Lysosome-associated membrane glycoprotein 1	0.83	NA	NA	2	4.1
P55209	Nucleosome assembly protein 1-like 1	0.83	NA	NA	2	9.0
P48643	T-complex protein 1 subunit epsilon	0.83	0.152	4.9E-02	6	11.8
P49368	T-complex protein 1 subunit gamma	0.82	0.157	1.2E-01	6	11.7
O75323	Protein NipSnap homolog 2	0.82	NA	NA	2	4.9
Q9Y230	RuvB-like 2	0.82	0.144	3.4E-01	3	8.6
Q13228	Selenium-binding protein 1	0.82	NA	NA	2	3.6
P00367	Glutamate dehydrogenase 1, mitochondrial	0.82	0.074	4.0E-02	6	11.5
Q99536	Synaptic vesicle membrane protein VAT-1 homolog	0.81	0.080	1.4E-02	12	41.5
P47756	F-actin-capping protein subunit beta	0.81	0.198	1.7E-01	3	11.6
P11310	Medium-chain specific acyl-CoA dehydrogenase, mitochondrial	0.81	NA	NA	2	5.5
P37235	Hippocalcin-like protein 1	0.80	NA	NA	2	9.3
P13073	Cytochrome c oxidase subunit 4 isoform 1, mitochondrial	0.80	0.178	2.9E-01	4	26.0
Q53GQ0	Estradiol 17-beta-dehydrogenase 12	0.80	NA	NA	2	8.7
Q92597	Protein NDRG1	0.79	NA	NA	2	7.6
P07437	Tubulin beta chain	0.79	NA	NA	2	6.1
P00505	Aspartate aminotransferase, mitochondrial	0.79	0.057	2.3E-02	4	11.4
P20674	Cytochrome c oxidase subunit 5A, mitochondrial	0.79	0.122	4.4E-01	3	20.0
O00483	NADH dehydrogenase [ubiquinone] 1 alpha subcomplex subunit 4	0.78	NA	NA	2	22.2
P31689	DnaJ homolog subfamily A member 1	0.78	0.080	1.5E-01	3	6.8
Q9Y3I0	tRNA-splicing ligase RtcB homolog	0.78	0.077	8.5E-02	3	6.7
P02042	Hemoglobin subunit delta	0.77	0.024	1.2E-02	3	30.6
P39656	Dolichyl-diphosphooligosaccharide--protein glycosyltransferase 48 kDa subunit	0.77	0.277	3.7E-01	5	11.0
Q12797	Aspartyl/asparaginyl beta-hydroxylase	0.77	0.104	4.2E-02	8	10.0
Q15075	Early endosome antigen 1	0.77	NA	NA	2	2.3
Q14011	Cold-inducible RNA-binding protein	0.77	NA	NA	2	14.5
P50990	T-complex protein 1 subunit theta	0.76	0.035	4.6E-04	6	8.9
P42167	Lamina-associated polypeptide 2, isoforms beta/gamma	0.76	1.950	5.5E-01	3	7.3
O95297	Myelin protein zero-like protein 1	0.75	NA	NA	2	8.2
P0CG05	Ig lambda-2 chain C regions	0.75	0.145	1.9E-01	3	46.2
P0C0L5	Complement C4-B	0.75	0.049	8.2E-03	7	3.5
P68871	Hemoglobin subunit beta	0.74	0.079	3.7E-03	5	44.9
O43707	Alpha-actinin-4	0.74	0.065	5.5E-04	18	22.2
P57053	Histone H2B type F-S	0.74	NA	NA	2	7.9
P53992	Protein transport protein Sec24C	0.73	0.391	3.9E-01	3	2.7
P17858	ATP-dependent 6-phosphofructokinase, liver type	0.73	0.085	5.6E-02	3	4.7
O75340	Programmed cell death protein 6	0.73	0.021	7.1E-04	3	15.2
Q7L5N1	COP9 signalosome complex subunit 6	0.73	NA	NA	2	6.7
Q02880	DNA topoisomerase 2-beta	0.72	NA	NA	2	0.9
P04632	Calpain small subunit 1	0.72	0.738	4.8E-01	3	8.6
P01834	Ig kappa chain C region	0.71	0.232	1.6E-01	3	51.9
P15311	Ezrin	0.71	NA	NA	2	2.6
Q9GZR7	ATP-dependent RNA helicase DDX24	0.71	7.142	6.8E-01	5	5.6
P55060	Exportin-2	0.71	0.111	5.3E-02	3	3.1
Q9Y265	RuvB-like 1	0.70	NA	NA	2	4.8
P07384	Calpain-1 catalytic subunit	0.70	0.112	6.0E-02	4	7.0
Q9UQE7	Structural maintenance of chromosomes protein 3	0.70	0.229	2.5E-01	3	2.1

Q9NZM1	Myoferlin	0.69	0.067	9.1E-05	13	7.3
P43304	Glycerol-3-phosphate dehydrogenase, mitochondrial	0.69	0.418	3.9E-01	3	4.3
P12235	ADP/ATP translocase 1	0.69	0.030	6.7E-03	3	10.4
O75489	NADH dehydrogenase [ubiquinone] iron-sulfur protein 3, mitochondrial	0.69	0.217	9.3E-02	3	14.0
E9PAV3	Nascent polypeptide-associated complex subunit alpha, muscle-specific form	0.69	NA	NA	2	0.7
Q7Z6Z7	E3 ubiquitin-protein ligase HUWE1	0.69	NA	NA	2	0.5
P55072	Transitional endoplasmic reticulum ATPase	0.69	0.049	1.3E-07	11	14.8
Q9BS26	Endoplasmic reticulum resident protein 44	0.69	0.036	1.4E-03	4	8.4
Q969X5	Endoplasmic reticulum-Golgi intermediate compartment protein 1	0.68	NA	NA	2	7.9
Q99832	T-complex protein 1 subunit eta	0.68	0.054	2.2E-04	5	10.1
P69905	Hemoglobin subunit alpha	0.68	0.096	2.4E-03	5	41.5
P54709	Sodium/potassium-transporting ATPase subunit beta-3	0.67	0.108	3.5E-03	8	32.6
P06727	Apolipoprotein A-IV	0.67	0.128	1.1E-02	11	26.3
P13861	cAMP-dependent protein kinase type II-alpha regulatory subunit	0.66	NA	NA	2	5.9
Q9HDC9	Adipocyte plasma membrane-associated protein	0.66	NA	NA	2	5.5
Q9Y6N5	Sulfide:quinone oxidoreductase, mitochondrial	0.66	0.161	1.1E-01	4	9.6
P01903	HLA class II histocompatibility antigen, DR alpha chain	0.66	0.126	3.3E-02	4	21.3
P09936	Ubiquitin carboxyl-terminal hydrolase isozyme L1	0.66	0.879	3.4E-01	4	26.0
P01876	Ig alpha-1 chain C region	0.65	NA	NA	2	4.5
Q9C0E8	Protein lunapark	0.65	NA	NA	2	3.7
P12110	Collagen alpha-2(VI) chain	0.65	0.085	8.2E-04	9	10.4
P04843	Dolichyl-diphosphooligosaccharide--protein glycosyltransferase subunit 1	0.64	0.045	7.7E-05	8	14.3
P04216	Thy-1 membrane glycoprotein	0.64	0.086	1.8E-03	4	24.8
P12111	Collagen alpha-3(VI) chain	0.64	0.030	2.2E-16	41	15.2
Q14847	LIM and SH3 domain protein 1	0.63	2.407	7.2E-01	3	12.3
Q9NQC3	Reticulon-4	0.63	0.035	1.0E-04	3	3.6
O75306	NADH dehydrogenase [ubiquinone] iron-sulfur protein 2, mitochondrial	0.63	NA	NA	2	5.6
P51812	Ribosomal protein S6 kinase alpha-3	0.62	NA	NA	2	2.8
Q00577	Transcriptional activator protein Pur-alpha	0.62	NA	NA	2	9.9
Q14204	Cytoplasmic dynein 1 heavy chain 1	0.62	0.057	5.7E-05	27	6.3
Q14203	Dynactin subunit 1	0.62	NA	NA	2	2.0
Q01082	Spectrin beta chain, non-erythrocytic 1	0.61	0.026	0.0E+00	66	33.1
P28288	ATP-binding cassette sub-family D member 3	0.61	NA	NA	2	3.8
P12109	Collagen alpha-1(VI) chain	0.61	0.062	2.4E-05	11	11.1
P31153	S-adenosylmethionine synthase isoform type-2	0.60	NA	NA	2	7.6
P63261	Actin, cytoplasmic 2	0.60	0.047	1.6E-04	5	16.8
Q14764	Major vault protein	0.60	0.084	2.5E-03	5	6.8
P12814	Alpha-actinin-1	0.60	0.086	5.9E-05	12	18.5
P49257	Protein ERGIC-53	0.59	0.057	8.0E-03	5	12.5
P02751	Fibronectin	0.59	0.141	7.4E-03	13	7.1
O75955	Flotillin-1	0.59	NA	NA	2	5.9
O00264	Membrane-associated progesterone receptor component 1	0.59	0.080	1.7E-02	4	16.4
Q6NUK1	Calcium-binding mitochondrial carrier protein SCaMC-1	0.59	0.104	3.2E-01	4	6.9
P26583	High mobility group protein B2	0.59	NA	NA	2	12.4
O75915	PRA1 family protein 3	0.59	0.086	1.3E-03	4	23.4
P04792	Heat shock protein beta-1	0.59	0.052	8.2E-06	6	31.2
P50995	Annexin A11	0.59	0.043	4.0E-05	4	7.7
P02652	Apolipoprotein A-II	0.58	NA	NA	2	17.0
Q9BSJ8	Extended synaptotagmin-1	0.58	0.073	1.4E-02	4	4.1
Q09666	Neuroblast differentiation-associated protein AHNAK	0.58	0.039	1.0E-05	58	8.8
P08123	Collagen alpha-2(I) chain	0.57	NA	NA	2	2.4
P54920	Alpha-soluble NSF attachment protein	0.57	NA	NA	2	8.5
P35611	Alpha-adducin	0.57	NA	NA	2	6.5
Q99442	Translocation protein SEC62	0.57	0.190	2.7E-01	3	7.3
P17987	T-complex protein 1 subunit alpha	0.57	NA	NA	2	3.8
P00403	Cytochrome c oxidase subunit 2	0.57	NA	NA	2	7.5
P62805	Histone H4	0.56	0.046	1.0E-09	7	52.4
Q12965	Unconventional myosin-Ie	0.56	NA	NA	2	1.5
P05107	Integrin beta-2	0.56	0.116	3.1E-02	3	4.2
O43242	26S proteasome non-ATPase regulatory subunit 3	0.56	NA	NA	2	4.1
P50570	Dynamin-2	0.56	0.069	4.0E-03	3	3.0
Q71DI3	Histone H3.2	0.55	0.133	1.7E-03	3	19.1
Q00610	Clathrin heavy chain 1	0.55	0.051	4.9E-10	26	18.8
Q9BQE3	Tubulin alpha-1C chain	0.55	0.110	1.1E-03	4	12.0
Q9BRX8	Redox-regulatory protein FAM213A	0.55	NA	NA	2	11.8
Q15404	Ras suppressor protein 1	0.54	NA	NA	2	9.4
O60716	Catenin delta-1	0.54	NA	NA	2	2.8
Q14344	Guanine nucleotide-binding protein subunit alpha-13	0.54	0.389	5.5E-01	3	9.3
P02462	Collagen alpha-1(IV) chain	0.54	0.257	1.5E-01	4	3.1
P01042	Kininogen-1	0.54	NA	NA	2	4.2
P0C0S5	Histone H2A.Z	0.54	NA	NA	2	18.8
P08107	Heat shock 70 kDa protein 1A/1B	0.54	0.049	1.2E-05	10	15.8
O43175	D-3-phosphoglycerate dehydrogenase	0.53	NA	NA	2	5.1
Q15019	Septin-2	0.53	0.089	3.4E-02	5	22.4
P07942	Laminin subunit beta-1	0.53	0.247	7.1E-02	3	1.7
P32119	Peroxiredoxin-2	0.53	0.102	6.0E-04	5	16.7
Q13813	Spectrin alpha chain, non-erythrocytic 1	0.52	0.022	0.0E+00	93	40.2
O14950	Myosin regulatory light chain 12B	0.52	NA	NA	2	12.2
P00390	Glutathione reductase, mitochondrial	0.51	NA	NA	2	6.3
P35580	Myosin-10	0.51	0.088	2.9E-05	13	8.6
P16615	Sarcoplasmic/endoplasmic reticulum calcium ATPase 2	0.51	0.172	3.1E-03	6	5.5
P09543	2',3'-cyclic-nucleotide 3'-phosphodiesterase	0.50	0.302	5.4E-02	4	6.9
Q01995	Transgelin	0.50	0.074	5.5E-05	5	28.9
Q16181	Septin-7	0.50	NA	NA	2	4.8
Q03252	Lamin-B2	0.49	0.030	8.3E-12	11	17.0
Q96HC4	PDZ and LIM domain protein 5	0.49	NA	NA	2	3.5
Q6DD88	Atlastin-3	0.49	NA	NA	2	3.5
P06737	Glycogen phosphorylase, liver form	0.48	NA	NA	2	3.1
Q9NZN4	EH domain-containing protein 2	0.48	0.163	1.7E-02	3	5.2
P47755	F-actin-capping protein subunit alpha-2	0.48	0.218	3.1E-01	3	19.6
Q16795	NADH dehydrogenase [ubiquinone] 1 alpha subcomplex subunit 9, mitochondrial	0.48	NA	NA	2	6.4
Q8WUM4	Programmed cell death 6-interacting protein	0.47	NA	NA	2	1.8
P60660	Myosin light polypeptide 6	0.47	0.045	1.6E-11	8	58.3
Q9Y490	Talin-1	0.47	0.061	7.0E-13	29	15.5
P06396	Gelsolin	0.46	0.085	2.5E-05	15	23.3
P68371	Tubulin beta-4B chain	0.46	NA	NA	2	7.2
P00387	NADH-cytochrome b5 reductase 3	0.45	0.073	2.4E-06	9	35.9
P05091	Aldehyde dehydrogenase, mitochondrial	0.45	NA	NA	2	3.7
Q14254	Flotillin-2	0.45	NA	NA	2	5.4
P04217	Alpha-1B-glycoprotein	0.44	NA	NA	2	4.8
O75131	Copine-3	0.44	0.066	1.0E-03	3	6.3
P08133	Annexin A6	0.44	0.028	0.0E+00	30	47.8
P06756	Integrin alpha-V	0.44	NA	NA	2	1.4
Q10567	AP-1 complex subunit beta-1	0.44	0.679	2.0E-01	5	4.8
P35579	Myosin-9	0.43	0.031	0.0E+00	61	30.0
Q15836	Vesicle-associated membrane protein 3	0.43	NA	NA	2	24.0
P08603	Complement factor H	0.43	0.246	2.0E-01	3	2.4
O94905	Erlin-2	0.43	0.243	5.2E-02	5	15.9
P43121	Cell surface glycoprotein MUC18	0.42	0.260	1.3E-01	4	8.5
Q16563	Synaptophysin-like protein 1	0.42	NA	NA	2	10.0
Q9BZQ8	Protein Niban	0.42	NA	NA	2	2.2
P00918	Carbonic anhydrase 2	0.42	NA	NA	2	10.8
Q96HN2	Putative adenosylhomocysteinase 3	0.42	NA	NA	2	3.6
P01600	Ig kappa chain V-I region Hau	0.41	NA	NA	2	22.2
P18206	Vinculin	0.41	0.055	7.3E-11	18	18.5

Q01813	ATP-dependent 6-phosphofructokinase, platelet type	0.41	NA	NA	2	3.7
P21333	Filamin-A	0.41	0.037	0.0E+00	54	26.7
P08572	Collagen alpha-2(IV) chain	0.41	0.132	2.9E-03	7	5.4
P01024	Complement C3	0.41	0.151	3.0E-05	16	10.2
O95782	AP-2 complex subunit alpha-1	0.41	NA	NA	2	2.0
P05023	Sodium/potassium-transporting ATPase subunit alpha-1	0.41	0.069	8.7E-09	15	17.3
P05556	Integrin beta-1	0.40	0.053	2.1E-07	7	10.0
P02686	Myelin basic protein	0.40	NA	NA	2	7.2
O75369	Filamin-B	0.39	0.010	5.0E-03	5	3.4
P05362	Intercellular adhesion molecule 1	0.38	NA	NA	2	5.3
Q5JWF2	Guanine nucleotide-binding protein G(s) subunit alpha isoforms XLas	0.37	0.403	2.1E-01	4	5.3
P08571	Monocyte differentiation antigen CD14	0.37	NA	NA	2	7.5
P0DJI8	Serum amyloid A-1 protein	0.34	NA	NA	2	29.5
Q13425	Beta-2-syntrophin	0.33	0.203	1.1E-01	3	3.9
Q14699	Raftlin	0.32	0.271	2.0E-01	3	5.7
Q13361	Microfibrillar-associated protein 5	0.31	NA	NA	2	13.3
Q6UXB8	Peptidase inhibitor 16	0.31	NA	NA	2	4.3
P14207	Folate receptor beta	0.31	NA	NA	2	7.1
P16157	Ankyrin-1	0.30	0.606	1.9E-01	3	2.4
Q9BXN1	Asporin	0.29	NA	NA	2	2.9
P60201	Myelin proteolipid protein	0.29	NA	NA	2	5.4
Q8TEX9	Importin-4	0.29	NA	NA	2	1.7
P13671	Complement component C6	0.29	NA	NA	2	2.6
P00167	Cytochrome b5	0.28	0.252	1.5E-01	3	35.8
Q13509	Tubulin beta-3 chain	0.27	NA	NA	2	5.8
O95865	N(G),N(G)-dimethylarginine dimethylaminohydrolase 2	0.27	NA	NA	2	8.4
P06899	Histone H2B type 1-J	0.27	NA	NA	2	7.9
P04040	Catalase	0.26	NA	NA	2	5.3
P68366	Tubulin alpha-4A chain	0.24	NA	NA	2	4.2
Q14624	Inter-alpha-trypsin inhibitor heavy chain H4	0.23	NA	NA	2	1.8
O14786	Neuropilin-1	0.23	NA	NA	2	3.5
P24844	Myosin regulatory light polypeptide 9	0.23	NA	NA	2	12.2
Q9HBL0	Tensin-1	0.23	0.170	1.4E-01	3	2.5
Q13642	Four and a half LIM domains protein 1	0.23	NA	NA	2	5.6
P02656	Apolipoprotein C-III	0.22	NA	NA	2	27.3
P04196	Histidine-rich glycoprotein	0.21	NA	NA	2	4.0
Q08431	Lactadherin	0.21	NA	NA	2	4.9
P43320	Beta-crystallin B2	0.20	0.060	1.8E-01	3	15.6
P35222	Catenin beta-1	0.20	NA	NA	2	4.0
P05787	Keratin, type II cytoskeletal 8	0.19	NA	NA	2	3.3
O94875	Sorbin and SH3 domain-containing protein 2	0.18	NA	NA	2	3.7
P07360	Complement component C8 gamma chain	0.18	NA	NA	2	18.3
Q969G5	Protein kinase C delta-binding protein	0.17	NA	NA	2	7.7
O14495	Lipid phosphate phosphohydrolase 3	0.17	NA	NA	2	7.4
Q8N5C1	Protein FAM26E	0.16	NA	NA	2	6.8
P21246	Pleiotrophin	0.16	NA	NA	2	9.5
Q03135	Caveolin-1	0.16	NA	NA	2	13.5
Q6UWY5	Olfactomedin-like protein 1	0.16	NA	NA	2	6.5
Q9NRN5	Olfactomedin-like protein 3	0.16	NA	NA	2	4.9
P22105	Tenascin-X	0.15	NA	NA	2	0.4
P58166	Inhibin beta E chain	0.14	0.364	8.5E-02	3	10.0
Q2UY09	Collagen alpha-1(XXVIII) chain	0.13	NA	NA	2	1.7
Q9BS40	Latexin	0.13	NA	NA	2	12.2
Q63ZY3	KN motif and ankyrin repeat domain-containing protein 2	0.13	NA	NA	2	2.7
Q14767	Latent-transforming growth factor beta-binding protein 2	0.13	NA	NA	2	0.9
P03973	Antileukoproteinase	0.12	NA	NA	2	15.9
P61626	Lysozyme C	0.09	NA	NA	2	12.8
Q9HCJ6	Synaptic vesicle membrane protein VAT-1 homolog-like	0.09	NA	NA	2	3.3
Q92777	Synapsin-2	0.09	NA	NA	2	4.1
Q14956	Transmembrane glycoprotein NMB	0.08	NA	NA	2	2.6
P17643	5,6-dihydroxyindole-2-carboxylic acid oxidase	0.08	NA	NA	2	5.6
P35556	Fibrillin-2	0.06	NA	NA	2	0.7
P10745	Retinol-binding protein 3	0.05	NA	NA	2	1.5
P07099	Epoxide hydrolase 1	0.38	0.048	4.1E-09	6	12.3
Q9Y6C2	EMILIN-1	0.38	0.119	4.9E-02	5	6.0
P68032	Actin, alpha cardiac muscle 1	0.38	0.192	7.5E-04	6	22.3
P09493	Tropomyosin alpha-1 chain	0.38	0.102	6.3E-07	5	12.0
P04899	Guanine nucleotide-binding protein G(i) subunit alpha-2	0.35	0.069	3.0E-07	6	19.4
P00352	Retinal dehydrogenase 1	0.35	0.155	1.5E-03	5	8.6
P13987	CD59 glycoprotein	0.33	0.105	1.2E-02	3	23.4
Q9UHG3	Prenylcysteine oxidase 1	0.33	0.167	4.1E-02	3	5.7
P00738	Haptoglobin	0.31	0.073	1.2E-06	7	18.0
O00159	Unconventional myosin-Ic	0.31	0.067	2.8E-05	11	11.4
Q16363	Laminin subunit alpha-4	0.31	0.140	1.9E-03	5	3.7
P98160	Basement membrane-specific heparan sulfate proteoglycan core protein	0.31	0.047	0.0E+00	31	9.0
Q07954	Prolow-density lipoprotein receptor-related protein 1	0.30	0.097	9.0E-06	10	3.1
O15230	Laminin subunit alpha-5	0.29	0.081	9.9E-07	12	4.3
P02749	Beta-2-glycoprotein 1	0.29	0.114	1.1E-07	5	20.6
O00468	Agrin	0.27	0.168	1.8E-02	6	4.9
P39060	Collagen alpha-1(XVIII) chain	0.26	0.125	5.2E-07	8	5.5
P55268	Laminin subunit beta-2	0.25	0.092	1.2E-07	13	8.6
P01011	Alpha-1-antichymotrypsin	0.25	0.089	2.6E-06	7	17.3
P08294	Extracellular superoxide dismutase [Cu-Zn]	0.25	0.084	4.0E-06	4	20.0
P11047	Laminin subunit gamma-1	0.25	0.079	2.5E-07	15	9.2
P07197	Neurofilament medium polypeptide	0.25	0.255	2.1E-02	3	4.6
O43301	Heat shock 70 kDa protein 12A	0.25	0.128	2.7E-03	4	6.7
Q9BTV4	Transmembrane protein 43	0.24	0.097	7.0E-03	4	15.3
P02679	Fibrinogen gamma chain	0.24	0.220	3.2E-04	11	26.7
P01008	Antithrombin-III	0.24	0.135	2.3E-04	6	14.7
P00747	Plasminogen	0.24	0.080	4.7E-07	8	8.9
P27105	Erythrocyte band 7 integral membrane protein	0.23	0.120	1.7E-03	6	20.1
P14543	Nidogen-1	0.23	0.070	2.4E-07	9	8.3
P02654	Apolipoprotein C-I	0.22	0.079	1.5E-04	3	24.1
Q6NZI2	Polymerase I and transcript release factor	0.22	0.132	5.8E-04	6	19.2
Q14112	Nidogen-2	0.22	0.071	3.4E-10	12	10.0
P36269	Gamma-glutamyltransferase 5	0.22	0.175	1.7E-03	4	8.7
P07355	Annexin A2	0.21	0.036	0.0E+00	23	54.0
P02511	Alpha-crystallin B chain	0.20	0.128	4.5E-06	5	29.1
P00450	Ceruloplasmin	0.20	0.165	1.5E-03	6	7.6
Q16555	Dihydropyrimidinase-related protein 2	0.20	0.155	1.2E-06	9	19.2
P02675	Fibrinogen beta chain	0.19	0.110	3.5E-05	8	22.2
Q05707	Collagen alpha-1(XIV) chain	0.19	0.228	7.5E-05	10	6.0
Q96CX2	BTB/POZ domain-containing protein KCTD12	0.19	0.189	1.7E-02	3	10.8
P60903	Protein S100-A10	0.18	0.098	6.1E-08	4	35.1
Q16853	Membrane primary amine oxidase	0.17	0.262	3.0E-02	3	3.7
P02549	Spectrin alpha chain, erythrocytic 1	0.17	0.234	7.1E-05	9	4.9
O43491	Band 4.1-like protein 2	0.17	0.216	7.1E-05	6	7.2
P80723	Brain acid soluble protein 1	0.17	0.176	1.1E-03	4	30.8
P04083	Annexin A1	0.16	0.042	5.5E-12	12	35.0
P02671	Fibrinogen alpha chain	0.16	0.105	5.5E-07	9	11.5
P02649	Apolipoprotein E	0.16	0.071	1.1E-11	13	44.8
P11166	Solute carrier family 2, facilitated glucose transporter member 1	0.16	0.197	1.9E-03	5	7.3
Q9NY15	Stabilin-1	0.15	0.133	7.0E-05	3	1.2
P05164	Myeloperoxidase	0.15	0.267	4.8E-02	4	7.1
P04275	von Willebrand factor	0.15	0.103	8.2E-09	12	4.7

P11277	Spectrin beta chain, erythrocytic	0.13	0.136	2.7E-03	7	4.9
P05186	Alkaline phosphatase, tissue-nonspecific isozyme	0.13	0.272	1.3E-03	3	6.9
P51888	Prolargin	0.13	0.128	1.2E-11	12	35.6
Q02952	A-kinase anchor protein 12	0.13	0.191	2.2E-06	13	10.2
P35749	Myosin-11	0.13	0.071	0.0E+00	30	16.4
P02730	Band 3 anion transport protein	0.12	0.153	2.5E-06	7	10.3
P39059	Collagen alpha-1(XV) chain	0.12	0.205	1.1E-04	5	3.9
Q14195	Dihydropyrimidinase-related protein 3	0.12	0.180	2.8E-04	8	22.8
P15088	Mast cell carboxypeptidase A	0.12	0.302	1.1E-02	5	10.1
Q13885	Tubulin beta-2A chain	0.11	0.177	1.5E-04	3	8.1
P35243	Recoverin	0.11	0.437	1.8E-02	3	14.5
P63211	Guanine nucleotide-binding protein G(T) subunit gamma-T1	0.11	0.215	1.6E-05	3	20.3
P02760	Protein AMBP	0.11	0.198	4.5E-03	4	19.0
P20774	Mimecan	0.11	0.177	8.8E-06	8	24.8
P51884	Lumican	0.10	0.057	0.0E+00	11	35.5
P21980	Protein-glutamine gamma-glutamyltransferase 2	0.10	0.105	4.0E-13	15	23.0
P01031	Complement C5	0.10	0.186	1.2E-04	7	4.4
P41219	Peripherin	0.09	0.236	7.5E-05	10	20.6
P35555	Fibrillin-1	0.09	0.067	0.0E+00	42	18.2
P01871	Ig mu chain C region	0.09	0.231	9.9E-07	9	24.6
P23946	Chymase	0.09	0.326	1.5E-02	3	16.6
P07585	Decorin	0.09	0.159	7.4E-03	8	23.1
P22352	Glutathione peroxidase 3	0.09	0.088	1.4E-05	5	16.8
P21810	Biglycan	0.09	0.076	8.9E-16	12	39.9
P21926	CD9 antigen	0.09	0.152	1.0E-05	3	9.6
Q9BXM0	Periaxin	0.08	0.434	1.1E-02	5	3.5
Q15661	Tryptase alpha/beta-1	0.08	0.192	1.6E-08	6	21.8
P10643	Complement component C7	0.08	0.278	4.5E-03	4	5.6
P22748	Carbonic anhydrase 4	0.07	0.337	1.1E-03	7	20.8
P02748	Complement component C9	0.06	0.198	3.3E-07	9	17.7
P10909	Clusterin	0.06	0.091	0.0E+00	17	37.0
P04004	Vitronectin	0.05	0.143	3.8E-12	9	19.5
P35625	Metalloproteinase inhibitor 3	0.04	0.176	4.4E-11	5	21.3
P02743	Serum amyloid P-component	0.04	0.232	2.0E-06	5	22.0
P25189	Myelin protein P0	0.02	0.226	2.1E-06	7	28.2

Brown denotes change ≥ 2 standard deviations (SD) from the mean, yellow denotes change ≥ 1 SD and green highlights p values ≤ 0.05. NA, not applicable, n<3 unique peptides.
