# Supplementary material for: iTRAQ Quantitative Proteomic Comparison of Metastatic and Non-Metastatic Uveal Melanoma Tumors
Source: PLoS One. 2015 Aug 25;10(8):e0135543. doi: 10.1371/journal.pone.0135543 (PMC4549237; doi:10.1371/journal.pone.0135543)
Supplement: S5 Table — (PDF) [file pone.0135543.s005.pdf]

Supplementary Table S5
Relative Protein Abundance: Sample UM30, Metastatic
Total Proteins Quantified = 912; LogMedian Protein Ratio = .15; LogMean Protein Ratio = 0; Standard Deviation = 0.90

Uni-Prot Accession	Protein	Ratio UM/Control	Standard Deviation	*p* value	Unique Peptides	% Sequence Coverage
P06454	Prothymosin alpha	15.69	0.464	3.6E-02	3	21.6
P62937	Peptidyl-prolyl cis-trans isomerase A	10.01	0.098	1.4E-12	7	38.2
P27695	DNA-(apurinic or apyrimidinic site) lyase	6.95	0.231	9.1E-03	3	11.9
P30086	Phosphatidylethanolamine-binding protein 1	6.04	0.102	3.1E-07	9	52.9
Q13510	Acid ceramidase	6.03	0.103	2.6E-10	8	16.2
P02768	Serum albumin	5.69	0.029	0.0E+00	39	62.6
P78417	Glutathione S-transferase omega-1	5.57	0.141	1.3E-08	10	35.3
P10599	Thioredoxin	5.52	0.144	7.1E-05	3	32.4
P57729	Ras-related protein Rab-38	5.52	0.171	1.0E-05	4	17.1
P63241	Eukaryotic translation initiation factor 5A-1	5.45	0.165	1.1E-03	7	33.1
P06865	Beta-hexosaminidase subunit alpha	5.44	0.088	1.5E-09	9	16.4
Q9BV36	Melanophilin	5.19	0.284	4.3E-02	3	5.8
P60174	Triosephosphate isomerase	5.06	0.115	4.1E-06	14	55.6
P06748	Nucleophosmin	4.99	0.083	7.7E-12	8	26.2
P07686	Beta-hexosaminidase subunit beta	4.93	0.167	1.3E-05	12	20.3
O15400	Syntaxin-7	4.90	0.112	4.3E-06	4	17.2
P23381	Tryptophan--tRNA ligase, cytoplasmic	4.81	0.166	3.9E-03	5	8.3
P16070	CD44 antigen	4.78	0.107	1.8E-07	11	13.6
O75368	SH3 domain-binding glutamic acid-rich-like protein	4.73	0.212	1.5E-04	3	21.1
P0DME0	Protein SETSIP	4.51	0.137	2.8E-06	4	15.6
P52565	Rho GDP-dissociation inhibitor 1	4.48	0.087	2.8E-09	4	22.5
P15531	Nucleoside diphosphate kinase A	4.45	0.105	5.3E-07	7	52.6
Q8IV08	Phospholipase D3	4.35	0.133	2.5E-06	3	6.3
P19338	Nucleolin	4.34	0.075	2.2E-16	18	21.3
P23528	Cofilin-1	4.29	0.147	3.4E-04	6	40.4
P00558	Phosphoglycerate kinase 1	4.27	0.099	3.6E-10	14	29.5
P09429	High mobility group protein B1	4.15	0.130	3.8E-05	6	21.9
P40926	Malate dehydrogenase, mitochondrial	4.10	0.076	1.9E-09	12	41.4
Q9Y2X3	Nucleolar protein 58	4.03	0.104	3.1E-04	4	10.8
Q00796	Sorbitol dehydrogenase	3.97	0.284	1.5E-02	3	4.8
Q08380	Galectin-3-binding protein	3.97	0.099	4.0E-06	5	10.4
P07195	L-lactate dehydrogenase B chain	3.81	0.057	1.5E-08	8	24.0
P12955	Xaa-Pro dipeptidase	3.78	0.230	3.9E-02	4	7.5
P04080	Cystatin-B	3.71	0.128	8.6E-07	4	52.0
P30837	Aldehyde dehydrogenase X, mitochondrial	3.70	0.101	1.0E-09	7	18.8
Q13185	Chromobox protein homolog 3	3.62	0.101	1.3E-05	4	20.8
P29401	Transketolase	3.57	0.143	9.1E-05	9	16.2
P02787	Serotransferrin	3.55	0.056	0.0E+00	19	30.1
P63104	14-3-3 protein zeta/delta	3.29	0.076	1.7E-03	9	42.4
P08758	Annexin A5	3.23	0.052	0.0E+00	16	49.1
P07602	Prosaposin	3.21	0.047	5.2E-10	5	7.1
O14818	Proteasome subunit alpha type-7	3.20	0.065	1.1E-04	4	14.9
Q07955	Serine/arginine-rich splicing factor 1	3.20	0.090	1.8E-07	5	17.3
P33121	Long-chain-fatty-acid--CoA ligase 1	3.15	0.141	8.3E-03	4	7.9
P40925	Malate dehydrogenase, cytoplasmic	3.13	0.127	2.2E-03	6	21.9
P25786	Proteasome subunit alpha type-1	3.04	0.088	1.3E-04	7	25.1
P25788	Proteasome subunit alpha type-3	3.00	0.086	4.0E-05	4	13.7
P31948	Stress-induced-phosphoprotein 1	2.98	0.088	1.8E-05	10	15.5
P20618	Proteasome subunit beta type-1	2.98	0.153	8.9E-05	3	13.3
Q9H3G5	Probable serine carboxypeptidase CPVL	2.96	0.162	6.4E-03	3	6.3
P23526	Adenosylhomocysteinase	2.93	0.127	1.8E-04	6	15.5
Q92820	Gamma-glutamyl hydrolase	2.92	0.147	1.1E-03	6	19.8
P39687	Acidic leucine-rich nuclear phosphoprotein 32 family member A	2.92	0.136	1.1E-03	5	14.5
P51858	Hepatoma-derived growth factor	2.92	0.135	8.7E-04	7	34.6
P04406	Glyceraldehyde-3-phosphate dehydrogenase	2.90	0.061	3.0E-08	9	30.7
P61916	Epididymal secretory protein E1	2.89	0.310	2.6E-02	3	17.2
P10253	Lysosomal alpha-glucosidase	2.79	0.218	2.1E-02	3	3.5
Q04760	Lactoylglutathione lyase	2.78	0.095	5.2E-05	3	14.1
P99999	Cytochrome c	2.77	0.136	9.4E-06	5	43.8
P06737	Glycogen phosphorylase, liver form	2.77	0.064	3.8E-14	16	18.2
P61604	10 kDa heat shock protein, mitochondrial	2.72	0.099	2.8E-05	6	51.0
O75380	NADH dehydrogenase [ubiquinone] iron-sulfur protein 6, mitochondrial	2.72	0.231	1.4E-02	4	31.5
P06733	Alpha-enolase	2.72	0.064	1.0E-09	12	33.9
P60842	Eukaryotic initiation factor 4A-I	2.69	0.090	5.6E-05	3	8.9
P07910	Heterogeneous nuclear ribonucleoproteins C1/C2	2.67	0.104	6.5E-06	9	27.1
O95336	6-phosphogluconolactonase	2.60	0.146	6.9E-03	5	25.2
P62158	Calmodulin	2.58	0.097	3.3E-05	5	34.2
P15121	Aldose reductase	2.57	0.137	6.1E-03	3	10.4
P00338	L-lactate dehydrogenase A chain	2.56	0.101	1.9E-06	8	20.8
P22087	rRNA 2'-O-methyltransferase fibrillarin	2.55	0.044	5.0E-03	4	13.1
P07858	Cathepsin B	2.52	0.066	1.2E-07	5	15.9
Q14247	Src substrate cortactin	2.50	0.261	1.4E-02	5	11.5
P40121	Macrophage-capping protein	2.48	0.255	1.7E-02	3	9.2
P51810	G-protein coupled receptor 143	2.47	0.123	2.1E-04	3	9.9
P06744	Glucose-6-phosphate isomerase	2.47	0.123	1.2E-04	7	13.1
Q9H1E3	Nuclear ubiquitous casein and cyclin-dependent kinase substrate 1	11.86	NA	NA	2	11.9
Q9UL41	Paraneoplastic antigen Ma3	7.45	NA	NA	2	5.4
P55769	NHP2-like protein 1	6.69	NA	NA	2	14.8
O00625	Pirin	6.02	NA	NA	2	6.2
P08236	Beta-glucuronidase	5.94	NA	NA	2	3.4
O14556	Glyceraldehyde-3-phosphate dehydrogenase, testis-specific	5.38	0.387	4.0E-01	3	8.8
P05413	Fatty acid-binding protein, heart	4.75	0.085	5.4E-02	3	21.8
Q8WWM9	Cytoglobin	4.71	NA	NA	2	8.4
P17096	High mobility group protein HMG-I/HMG-Y	4.56	NA	NA	2	23.4
P07108	Acyl-CoA-binding protein	4.56	NA	NA	2	41.4
P58546	Myotrophin	4.08	NA	NA	2	25.4
Q15843	NEDD8	3.88	NA	NA	2	17.3
Q03154	Aminoacylase-1	3.45	0.301	4.0E-01	3	7.4
Q9UBR2	Cathepsin Z	3.45	0.142	9.7E-02	3	9.6
P62310	U6 snRNA-associated Sm-like protein LSm3	3.26	NA	NA	2	19.6
P02766	Transthyretin	3.25	0.434	6.0E-02	3	28.6
Q08170	Serine/arginine-rich splicing factor 4	3.19	NA	NA	2	4.3
P13693	Translationally-controlled tumor protein	3.11	NA	NA	2	15.7
Q8IZQ5	Selenoprotein H	3.11	NA	NA	2	18.0
P11766	Alcohol dehydrogenase class-3	3.07	NA	NA	2	4.5
Q9Y3C8	Ubiquitin-fold modifier-conjugating enzyme 1	3.01	NA	NA	2	11.4
Q9NRV9	Heme-binding protein 1	2.99	NA	NA	2	13.8
P63173	60S ribosomal protein L38	2.91	NA	NA	2	31.4
P55809	Succinyl-CoA:3-ketoacid coenzyme A transferase 1, mitochondrial	2.91	NA	NA	2	3.5
P52815	39S ribosomal protein L12, mitochondrial	2.90	0.295	8.0E-02	3	11.6
Q9BXK5	Bcl-2-like protein 13	2.86	NA	NA	2	5.2
P60900	Proteasome subunit alpha type-6	2.82	0.511	2.3E-01	3	15.4
P07919	Cytochrome b-c1 complex subunit 6, mitochondrial	2.71	NA	NA	2	15.4
P40967	Melanocyte protein PMEL	2.71	NA	NA	2	3.3
P17900	Ganglioside GM2 activator	2.69	NA	NA	2	7.8
Q53EL6	Programmed cell death protein 4	2.69	NA	NA	2	4.7
Q13573	SNW domain-containing protein 1	2.68	NA	NA	2	4.7
Q6GMV3	Putative peptidyl-tRNA hydrolase PTRHD1	2.54	NA	NA	2	19.3

Q8N183	Mimitin, mitochondrial	2.54	NA	NA	2	13.0
O00560	Syntenin-1	2.54	0.273	1.2E-01	4	10.7
Q9Y2S2	Lambda-crystallin homolog	2.53	0.388	9.1E-02	3	10.7
P39748	Flap endonuclease 1	2.53	NA	NA	2	4.5
Q99572	P2X purinoceptor 7	2.53	NA	NA	2	4.5
P30044	Peroxiredoxin-5, mitochondrial	2.50	NA	NA	2	11.7
P07900	Heat shock protein HSP 90-alpha	2.46	0.063	8.5E-06	15	17.6
P16401	Histone H1.5	2.46	NA	NA	2	9.7
P20042	Eukaryotic translation initiation factor 2 subunit 2	2.44	NA	NA	2	9.9
Q16658	Fascin	2.44	NA	NA	2	6.1
P15586	N-acetylglucosamine-6-sulfatase	2.44	0.074	8.1E-04	3	4.7
Q9UHL4	Dipeptidyl peptidase 2	2.44	0.657	2.6E-01	3	6.9
Q99497	Protein DJ-1	2.43	0.182	5.8E-02	6	28.0
P13797	Plastin-3	2.43	0.148	2.6E-03	8	15.2
Q01130	Serine/arginine-rich splicing factor 2	2.40	0.058	2.4E-04	3	14.5
P62857	40S ribosomal protein S28	2.40	NA	NA	2	30.4
P17643	5,6-dihydroxyindole-2-carboxylic acid oxidase	2.39	0.127	3.0E-02	8	15.3
Q14103	Heterogeneous nuclear ribonucleoprotein D0	2.38	0.094	7.5E-05	3	9.0
P62826	GTP-binding nuclear protein Ran	2.38	NA	NA	2	9.7
P07339	Cathepsin D	2.37	0.083	1.1E-07	8	19.7
Q13838	Spliceosome RNA helicase DDX39B	2.37	0.089	5.4E-05	3	7.2
Q9GZY8	Mitochondrial fission factor	2.36	NA	NA	2	6.4
Q15631	Translin	2.35	NA	NA	2	8.8
P49773	Histidine triad nucleotide-binding protein 1	2.35	0.380	6.4E-02	3	31.7
P53999	Activated RNA polymerase II transcriptional coactivator p15	2.34	NA	NA	2	15.7
P08670	Vimentin	2.34	0.046	0.0E+00	27	56.4
P07741	Adenine phosphoribosyltransferase	2.33	NA	NA	2	10.0
O14979	Heterogeneous nuclear ribonucleoprotein D-like	2.32	0.087	2.8E-05	3	4.5
P10721	Mast/stem cell growth factor receptor Kit	2.32	0.097	2.3E-02	3	2.4
P05387	60S acidic ribosomal protein P2	2.32	0.086	2.8E-06	6	60.9
Q12906	Interleukin enhancer-binding factor 3	2.30	0.123	9.6E-05	12	16.0
Q00059	Transcription factor A, mitochondrial	2.29	0.069	1.2E-03	5	20.3
Q86UE4	Protein LYRIC	2.28	NA	NA	2	2.7
P08195	4F2 cell-surface antigen heavy chain	2.28	0.078	2.4E-06	8	16.3
P37837	Transaldolase	2.27	0.115	2.4E-03	7	19.3
Q08AM6	Protein VAC14 homolog	2.25	NA	NA	2	2.8
Q13442	28 kDa heat- and acid-stable phosphoprotein	2.24	NA	NA	2	13.8
P00441	Superoxide dismutase [Cu-Zn]	2.23	0.158	1.7E-03	3	16.9
P09622	Dihydrolipoyl dehydrogenase, mitochondrial	2.23	0.070	1.0E-06	6	12.2
P30533	Alpha-2-macroglobulin receptor-associated protein	2.17	0.074	2.0E-02	4	11.2
Q96KP4	Cytosolic non-specific dipeptidase	2.16	0.064	1.9E-04	5	13.9
Q12905	Interleukin enhancer-binding factor 2	2.14	0.061	2.3E-05	3	9.5
P48637	Glutathione synthetase	2.14	NA	NA	2	3.8
Q00688	Peptidyl-prolyl cis-trans isomerase FKBP3	2.14	NA	NA	2	9.8
P09417	Dihydropteridine reductase	2.13	NA	NA	2	12.3
P06753	Tropomyosin alpha-3 chain	2.13	0.113	8.5E-05	4	13.0
P30042	ES1 protein homolog, mitochondrial	2.12	0.217	1.8E-02	6	28.4
P51608	Methyl-CpG-binding protein 2	2.12	NA	NA	2	5.1
P13489	Ribonuclease inhibitor	2.09	0.085	1.3E-02	3	6.1
Q9Y4W6	AFG3-like protein 2	2.08	0.153	2.0E-02	8	10.8
P43243	Matrin-3	2.07	0.151	6.0E-02	3	4.1
Q9BRA2	Thioredoxin domain-containing protein 17	2.06	NA	NA	2	18.7
P62851	40S ribosomal protein S25	2.06	0.070	6.5E-05	4	24.0
P23246	Splicing factor, proline- and glutamine-rich	2.05	0.103	1.7E-04	7	10.6
P24534	Elongation factor 1-beta	2.05	0.129	2.2E-02	3	13.3
P25398	40S ribosomal protein S12	2.04	NA	NA	2	13.6
P51688	N-sulphoglucosamine sulphohydrolase	2.03	NA	NA	2	4.8
P18669	Phosphoglycerate mutase 1	2.02	0.083	7.8E-05	5	29.9
O75083	WD repeat-containing protein 1	2.02	0.098	1.1E-03	5	8.3
Q99536	Synaptic vesicle membrane protein VAT-1 homolog	2.01	0.073	2.5E-09	12	38.7
Q16698	2,4-dienoyl-CoA reductase, mitochondrial	2.00	0.088	1.6E-02	5	16.4
O75351	Vacuolar protein sorting-associated protein 4B	2.00	NA	NA	2	5.4
P13639	Elongation factor 2	1.99	0.097	9.4E-08	15	19.2
P45973	Chromobox protein homolog 5	1.98	NA	NA	2	11.0
P62258	14-3-3 protein epsilon	1.97	0.111	3.3E-03	9	33.7
Q99798	Aconitate hydratase, mitochondrial	1.96	0.069	2.9E-05	8	12.4
P02765	Alpha-2-HS-glycoprotein	1.96	0.301	2.8E-01	3	7.4
P31939	Bifunctional purine biosynthesis protein PURH	1.95	0.442	2.0E-01	5	11.0
Q14956	Transmembrane glycoprotein NMB	1.94	0.281	1.7E-02	5	10.0
P50395	Rab GDP dissociation inhibitor beta	1.94	0.098	7.8E-05	7	18.4
P61978	Heterogeneous nuclear ribonucleoprotein K	1.94	0.066	2.8E-05	13	32.4
Q06787	Fragile X mental retardation protein 1	1.94	NA	NA	2	2.7
P22626	Heterogeneous nuclear ribonucleoproteins A2/B1	1.94	0.061	9.9E-09	13	33.1
P09651	Heterogeneous nuclear ribonucleoprotein A1	1.93	0.044	1.6E-09	6	16.7
Q15717	ELAV-like protein 1	1.93	NA	NA	2	7.7
O14561	Acyl carrier protein, mitochondrial	1.91	NA	NA	2	9.6
P35268	60S ribosomal protein L22	1.90	NA	NA	2	18.8
P0CW22	40S ribosomal protein S17-like	1.89	0.126	6.3E-03	3	16.3
Q96C86	m7GpppX diphosphatase	1.86	0.488	4.3E-01	3	11.6
P21796	Voltage-dependent anion-selective channel protein 1	1.86	0.059	8.9E-05	8	35.0
P14854	Cytochrome c oxidase subunit 6B1	1.86	0.085	1.3E-01	3	24.4
Q96AG4	Leucine-rich repeat-containing protein 59	1.85	0.076	5.7E-02	4	12.7
P07954	Fumarate hydratase, mitochondrial	1.85	NA	NA	2	4.3
P08574	Cytochrome c1, heme protein, mitochondrial	1.85	0.064	7.1E-04	3	8.3
Q92945	Far upstream element-binding protein 2	1.84	0.186	1.3E-02	7	12.9
P36543	V-type proton ATPase subunit E 1	1.84	NA	NA	2	7.1
P08238	Heat shock protein HSP 90-beta	1.83	0.094	1.5E-04	8	10.9
Q02818	Nucleobindin-1	1.83	0.142	6.1E-03	6	16.1
Q08211	ATP-dependent RNA helicase A	1.83	0.158	1.8E-03	8	7.0
Q14677	Clathrin interactor 1	1.83	0.353	2.2E-01	3	5.6
Q9UPN3	Microtubule-actin cross-linking factor 1, isoforms 1/2/3/5	1.82	NA	NA	2	0.3
O00483	NADH dehydrogenase [ubiquinone] 1 alpha subcomplex subunit 4	1.80	NA	NA	2	22.2
P28066	Proteasome subunit alpha type-5	1.80	NA	NA	2	10.8
Q9Y6M9	NADH dehydrogenase [ubiquinone] 1 beta subcomplex subunit 9	1.80	0.258	6.0E-02	3	19.6
P16152	Carbonyl reductase [NADPH] 1	1.80	NA	NA	2	8.7
Q16629	Serine/arginine-rich splicing factor 7	1.80	NA	NA	2	7.6
P26440	Isovaleryl-CoA dehydrogenase, mitochondrial	1.80	NA	NA	2	4.5
P07093	Glia-derived nexin	1.79	NA	NA	2	6.0
O95182	NADH dehydrogenase [ubiquinone] 1 alpha subcomplex subunit 7	1.79	NA	NA	2	8.8
P30049	ATP synthase subunit delta, mitochondrial	1.78	NA	NA	2	13.7
P61247	40S ribosomal protein S3a	1.78	0.139	1.0E-01	5	18.6
Q15904	V-type proton ATPase subunit S1	1.77	NA	NA	2	4.3
P12270	Nucleoprotein TPR	1.77	0.064	6.6E-05	6	3.7
Q9H2U2	Inorganic pyrophosphatase 2, mitochondrial	1.77	0.183	1.6E-02	5	19.8
Q9NTZ6	RNA-binding protein 12	1.77	NA	NA	2	3.3
P16930	Fumarylacetoacetase	1.76	NA	NA	2	4.3
P14618	Pyruvate kinase PKM	1.75	0.043	1.6E-12	10	23.0
P11142	Heat shock cognate 71 kDa protein	1.75	0.095	1.6E-04	14	23.2
E9PAV3	Nascent polypeptide-associated complex subunit alpha, muscle-specific form	1.74	0.175	1.1E-02	3	2.0
Q9BZZ5	Apoptosis inhibitor 5	1.74	NA	NA	2	3.4
P39019	40S ribosomal protein S19	1.74	0.064	1.4E-03	4	24.1
P22314	Ubiquitin-like modifier-activating enzyme 1	1.74	0.143	3.1E-03	8	8.5
Q15365	Poly(rC)-binding protein 1	1.73	0.204	4.5E-02	3	10.7
P21283	V-type proton ATPase subunit C 1	1.72	NA	NA	2	4.5
O43852	Calumenin	1.71	0.088	2.7E-04	5	18.4

O95299	NADH dehydrogenase [ubiquinone] 1 alpha subcomplex subunit 10, mitochondrial	1.71	NA	NA	2	6.2
P61421	V-type proton ATPase subunit d 1	1.70	0.053	1.3E-04	4	11.4
O95292	Vesicle-associated membrane protein-associated protein B/C	1.70	0.754	3.5E-01	3	14.4
P51991	Heterogeneous nuclear ribonucleoprotein A3	1.70	0.050	1.1E-05	6	17.7
P13010	X-ray repair cross-complementing protein 5	1.70	0.133	7.0E-03	8	13.3
P54819	Adenylate kinase 2, mitochondrial	1.69	0.163	1.9E-02	3	14.2
P62263	40S ribosomal protein S14	1.69	0.236	5.2E-02	4	36.4
P07737	Profilin-1	1.69	0.133	6.1E-02	5	40.7
O75489	NADH dehydrogenase [ubiquinone] iron-sulfur protein 3, mitochondrial	1.68	0.060	8.0E-06	5	19.3
Q07960	Rho GTPase-activating protein 1	1.68	0.081	2.5E-02	4	7.3
P49411	Elongation factor Tu, mitochondrial	1.68	0.097	2.4E-05	10	27.7
Q9UH65	Switch-associated protein 70	1.68	NA	NA	2	3.6
A4D1P6	WD repeat-containing protein 91	1.68	NA	NA	2	1.9
P46783	40S ribosomal protein S10	1.67	0.270	3.5E-01	3	20.0
P29692	Elongation factor 1-delta	1.66	0.134	5.1E-02	3	10.7
P52597	Heterogeneous nuclear ribonucleoprotein F	1.66	0.100	1.3E-02	4	8.2
P46777	60S ribosomal protein L5	1.64	0.097	1.2E-03	4	15.5
P37802	Transgelin-2	1.64	NA	NA	2	10.1
Q9Y2Q3	Glutathione S-transferase kappa 1	1.64	NA	NA	2	11.9
P26599	Polypyrimidine tract-binding protein 1	1.64	0.119	7.3E-03	4	6.2
P42704	Leucine-rich PPR motif-containing protein, mitochondrial	1.63	0.075	5.0E-04	10	7.1
P52272	Heterogeneous nuclear ribonucleoprotein M	1.63	0.124	4.0E-03	11	15.9
Q9Y383	Putative RNA-binding protein Luc7-like 2	1.63	NA	NA	2	4.6
P62829	60S ribosomal protein L23	1.63	NA	NA	2	16.4
O95298	NADH dehydrogenase [ubiquinone] 1 subunit C2	1.63	NA	NA	2	16.8
Q16655	Melanoma antigen recognized by T-cells 1	1.62	NA	NA	2	17.8
Q15907	Ras-related protein Rab-11B	1.62	0.150	1.1E-02	4	17.4
Q9NP81	Serine--tRNA ligase, mitochondrial	1.62	NA	NA	2	6.9
Q9Y2B0	Protein canopy homolog 2	1.61	0.150	6.2E-02	4	25.8
O75390	Citrate synthase, mitochondrial	1.61	0.095	2.1E-02	5	11.8
P54652	Heat shock-related 70 kDa protein 2	1.61	0.126	6.8E-02	5	10.2
P26885	Peptidyl-prolyl cis-trans isomerase FKBP2	1.61	NA	NA	2	9.2
Q15233	Non-POU domain-containing octamer-binding protein	1.61	0.263	5.8E-02	5	13.4
Q13263	Transcription intermediary factor 1-beta	1.60	0.133	8.8E-03	7	7.9
P36955	Pigment epithelium-derived factor	1.60	0.109	2.4E-02	4	10.3
P26641	Elongation factor 1-gamma	1.59	0.167	4.1E-02	4	8.9
P12830	Cadherin-1	1.58	0.186	1.1E-01	7	8.4
P53597	Succinyl-CoA ligase [ADP/GDP-forming] subunit alpha, mitochondrial	1.58	NA	NA	2	6.9
P08865	40S ribosomal protein SA	1.58	0.123	2.0E-03	6	25.4
Q00839	Heterogeneous nuclear ribonucleoprotein U	1.57	0.048	2.9E-06	10	12.6
Q13123	Protein Red	1.57	NA	NA	2	3.8
P78527	DNA-dependent protein kinase catalytic subunit	1.57	0.056	1.7E-06	17	3.8
O43399	Tumor protein D54	1.57	0.165	7.8E-02	3	15.0
P26373	60S ribosomal protein L13	1.57	0.082	1.1E-03	4	19.0
P12956	X-ray repair cross-complementing protein 6	1.57	0.083	5.3E-04	7	10.8
P34932	Heat shock 70 kDa protein 4	1.56	0.079	9.2E-03	7	8.1
P36578	60S ribosomal protein L4	1.56	0.190	2.0E-02	5	11.2
P61019	Ras-related protein Rab-2A	1.56	0.125	7.3E-02	3	20.3
Q92688	Acidic leucine-rich nuclear phosphoprotein 32 family member B	1.56	0.170	5.8E-02	6	17.5
P37108	Signal recognition particle 14 kDa protein	1.55	NA	NA	2	12.5
P19404	NADH dehydrogenase [ubiquinone] flavoprotein 2, mitochondrial	1.55	NA	NA	2	9.6
Q8NC51	Plasminogen activator inhibitor 1 RNA-binding protein	1.55	0.186	1.7E-01	4	9.1
P51149	Ras-related protein Rab-7a	1.55	0.091	1.3E-03	4	18.8
P38646	Stress-70 protein, mitochondrial	1.54	0.159	9.8E-03	11	18.4
O75533	Splicing factor 3B subunit 1	1.54	NA	NA	2	1.7
Q9BUJ2	Heterogeneous nuclear ribonucleoprotein U-like protein 1	1.54	0.141	1.8E-02	4	6.3
P49207	60S ribosomal protein L34	1.54	NA	NA	2	13.7
P02790	Hemopexin	1.54	0.088	1.5E-03	4	7.6
P04075	Fructose-bisphosphate aldolase A	1.53	0.052	5.7E-05	13	44.2
P55786	Puromycin-sensitive aminopeptidase	1.53	0.297	1.2E-01	4	4.9
Q5VTE0	Putative elongation factor 1-alpha-like 3	1.53	0.066	1.1E-04	10	21.4
P62081	40S ribosomal protein S7	1.53	NA	NA	2	8.8
Q16836	Hydroxyacyl-coenzyme A dehydrogenase, mitochondrial	1.53	NA	NA	2	7.3
P46926	Glucosamine-6-phosphate isomerase 1	1.52	0.128	1.6E-02	5	14.2
O94906	Pre-mRNA-processing factor 6	1.52	NA	NA	2	2.1
P14314	Glucosidase 2 subunit beta	1.52	0.091	1.1E-02	8	11.4
Q14683	Structural maintenance of chromosomes protein 1A	1.51	0.326	8.6E-02	3	2.4
Q8TAQ2	SWI/SNF complex subunit SMARCC2	1.50	0.138	2.3E-01	3	3.0
P05141	ADP/ATP translocase 2	1.50	0.102	2.8E-02	4	13.1
O76021	Ribosomal L1 domain-containing protein 1	1.49	NA	NA	2	4.5
Q658Y4	Protein FAM91A1	1.48	NA	NA	2	1.9
P84077	ADP-ribosylation factor 1	1.48	0.040	9.3E-04	3	18.2
Q8IVF2	Protein AHNAK2	1.48	0.268	6.2E-01	11	0.7
Q13428	Treacle protein	1.48	NA	NA	2	1.6
O75494	Serine/arginine-rich splicing factor 10	1.48	NA	NA	2	8.4
P10606	Cytochrome c oxidase subunit 5B, mitochondrial	1.48	0.156	4.3E-02	6	31.0
P16402	Histone H1.3	1.47	0.108	1.0E-03	6	15.4
Q14974	Importin subunit beta-1	1.47	0.757	2.3E-01	4	6.7
P21291	Cysteine and glycine-rich protein 1	1.47	NA	NA	2	14.0
P48426	Phosphatidylinositol 5-phosphate 4-kinase type-2 alpha	1.47	NA	NA	2	4.9
P09525	Annexin A4	1.47	0.123	1.8E-03	10	28.5
P00505	Aspartate aminotransferase, mitochondrial	1.46	0.061	4.6E-04	7	18.6
O14737	Programmed cell death protein 5	1.46	NA	NA	2	17.6
Q9GZT3	SRA stem-loop-interacting RNA-binding protein, mitochondrial	1.45	NA	NA	2	24.8
Q9UHX1	Poly(U)-binding-splicing factor PUF60	1.45	0.120	9.7E-02	4	7.7
Q07666	KH domain-containing, RNA-binding, signal transduction-associated protein 1	1.45	0.096	2.4E-02	4	7.9
P16219	Short-chain specific acyl-CoA dehydrogenase, mitochondrial	1.45	NA	NA	2	4.6
O14773	Tripeptidyl-peptidase 1	1.45	0.057	3.1E-04	4	8.7
P54727	UV excision repair protein RAD23 homolog B	1.45	0.236	8.2E-02	4	9.3
Q06323	Proteasome activator complex subunit 1	1.44	0.159	6.4E-02	3	14.1
P62269	40S ribosomal protein S18	1.44	0.050	1.7E-04	6	31.6
P78347	General transcription factor II-I	1.44	0.084	5.7E-03	5	3.7
P30084	Enoyl-CoA hydratase, mitochondrial	1.44	NA	NA	2	8.3
P14868	Aspartate--tRNA ligase, cytoplasmic	1.44	0.085	6.0E-03	4	11.0
Q14152	Eukaryotic translation initiation factor 3 subunit A	1.44	0.102	4.9E-02	5	4.5
P20674	Cytochrome c oxidase subunit 5A, mitochondrial	1.44	0.113	6.7E-02	4	20.7
O95202	LETM1 and EF-hand domain-containing protein 1, mitochondrial	1.43	0.103	1.9E-02	4	6.1
Q9HD20	Manganese-transporting ATPase 13A1	1.43	0.223	1.9E-01	3	3.2
Q96I24	Far upstream element-binding protein 3	1.43	0.109	2.8E-02	4	7.7
P27348	14-3-3 protein theta	1.42	0.085	7.8E-02	3	6.9
P09211	Glutathione S-transferase P	1.41	0.230	2.2E-01	5	33.8
Q96I99	Succinyl-CoA ligase [GDP-forming] subunit beta, mitochondrial	1.41	NA	NA	2	5.8
P28331	NADH-ubiquinone oxidoreductase 75 kDa subunit, mitochondrial	1.41	0.081	2.2E-02	4	5.9
Q86VP6	Cullin-associated NEDD8-dissociated protein 1	1.41	NA	NA	2	1.5
Q9UKM9	RNA-binding protein Raly	1.40	NA	NA	2	10.8
P50213	Isocitrate dehydrogenase [NAD] subunit alpha, mitochondrial	1.40	0.068	6.1E-03	5	14.5
P49189	4-trimethylaminobutyraldehyde dehydrogenase	1.40	NA	NA	2	3.6
P48047	ATP synthase subunit O, mitochondrial	1.40	0.126	3.0E-03	4	26.3
P51159	Ras-related protein Rab-27A	1.40	0.034	7.4E-04	3	15.4
P62906	60S ribosomal protein L10a	1.39	0.136	4.2E-02	6	27.6
P49591	Serine--tRNA ligase, cytoplasmic	1.39	NA	NA	2	5.3
Q13011	Delta(3,5)-Delta(2,4)-dienoyl-CoA isomerase, mitochondrial	1.39	0.073	4.1E-04	5	15.2
P35232	Prohibitin	1.39	0.047	1.6E-06	7	26.8
Q8NDH3	Probable aminopeptidase NPEPL1	1.39	NA	NA	2	4.2
O95168	NADH dehydrogenase [ubiquinone] 1 beta subcomplex subunit 4	1.38	NA	NA	2	17.8

P62750	60S ribosomal protein L23a	1.38	NA	NA	2	13.5
P14866	Heterogeneous nuclear ribonucleoprotein L	1.38	0.054	3.0E-04	5	10.2
Q08945	FACT complex subunit SSRP1	1.38	NA	NA	2	2.0
P31153	S-adenosylmethionine synthase isoform type-2	1.38	0.062	5.4E-02	3	9.4
P26368	Splicing factor U2AF 65 kDa subunit	1.37	NA	NA	2	5.9
Q8N5K1	CDGSH iron-sulfur domain-containing protein 2	1.37	0.186	8.0E-02	3	26.7
Q9BVK6	Transmembrane emp24 domain-containing protein 9	1.37	0.060	6.8E-03	3	12.8
O75306	NADH dehydrogenase [ubiquinone] iron-sulfur protein 2, mitochondrial	1.37	0.382	1.3E-01	5	12.3
P09669	Cytochrome c oxidase subunit 6C	1.37	0.218	8.5E-02	5	49.3
Q71UM5	40S ribosomal protein S27-like	1.36	NA	NA	2	23.8
Q9NX63	Coiled-coil-helix-coiled-coil-helix domain-containing protein 3, mitochondrial	1.36	0.025	2.3E-03	5	18.1
P30048	Thioredoxin-dependent peroxide reductase, mitochondrial	1.36	0.165	8.6E-02	7	24.6
O75367	Core histone macro-H2A.1	1.36	0.148	1.6E-02	8	24.2
P49755	Transmembrane emp24 domain-containing protein 10	1.36	0.437	4.2E-01	3	19.2
P18859	ATP synthase-coupling factor 6, mitochondrial	1.36	0.336	1.7E-01	5	41.7
P62277	40S ribosomal protein S13	1.36	0.160	4.1E-02	6	29.1
P09012	U1 small nuclear ribonucleoprotein A	1.36	NA	NA	2	7.4
P10809	60 kDa heat shock protein, mitochondrial	1.35	0.185	1.8E-01	12	22.5
Q9P2E9	Ribosome-binding protein 1	1.35	0.144	3.6E-02	5	4.4
O75947	ATP synthase subunit d, mitochondrial	1.35	0.153	1.2E-01	6	28.6
Q9Y224	UPF0568 protein C14orf166	1.35	NA	NA	2	11.1
P50914	60S ribosomal protein L14	1.35	0.226	3.6E-01	3	16.3
P61353	60S ribosomal protein L27	1.34	0.098	2.4E-02	3	27.9
P29966	Myristoylated alanine-rich C-kinase substrate	1.34	0.405	1.5E-01	4	22.0
P46940	Ras GTPase-activating-like protein IQGAP1	1.34	0.133	1.7E-02	12	9.1
O00567	Nucleolar protein 56	1.34	0.067	2.5E-02	4	7.2
P50502	Hsc70-interacting protein	1.34	0.132	9.2E-02	4	10.8
O15212	Prefoldin subunit 6	1.34	NA	NA	2	14.0
P11940	Polyadenylate-binding protein 1	1.33	0.143	5.5E-02	6	12.3
Q9P016	Thymocyte nuclear protein 1	1.33	0.075	8.9E-02	3	11.6
P60866	40S ribosomal protein S20	1.33	NA	NA	2	19.3
P31949	Protein S100-A11	1.33	0.186	1.3E-01	4	42.9
P54136	Arginine--tRNA ligase, cytoplasmic	1.33	0.225	1.5E-01	3	5.5
Q8N2K0	Monoacylglycerol lipase ABHD12	1.32	NA	NA	2	5.3
P04179	Superoxide dismutase [Mn], mitochondrial	1.32	0.124	1.7E-01	7	24.3
Q9NR28	Diablo homolog, mitochondrial	1.32	NA	NA	2	8.8
O15145	Actin-related protein 2/3 complex subunit 3	1.32	NA	NA	2	9.6
P27816	Microtubule-associated protein 4	1.32	0.184	2.2E-01	4	4.1
P84103	Serine/arginine-rich splicing factor 3	1.31	0.106	2.6E-02	3	20.7
P05198	Eukaryotic translation initiation factor 2 subunit 1	1.31	NA	NA	2	7.0
P51532	Transcription activator BRG1	1.31	NA	NA	2	1.7
Q12874	Splicing factor 3A subunit 3	1.31	NA	NA	2	4.0
P42766	60S ribosomal protein L35	1.31	NA	NA	2	15.4
P62979	Ubiquitin-40S ribosomal protein S27a	1.31	0.068	8.8E-04	8	41.0
P38117	Electron transfer flavoprotein subunit beta	1.30	0.197	8.0E-02	7	22.4
Q15691	Microtubule-associated protein RP/EB family member 1	1.30	0.158	1.5E-01	6	19.4
P17844	Probable ATP-dependent RNA helicase DDX5	1.30	0.033	7.4E-03	4	6.0
Q9BWM7	Sideroflexin-3	1.30	NA	NA	2	8.9
P62191	26S protease regulatory subunit 4	1.30	NA	NA	2	5.0
P02545	Prelamin-A/C	1.29	0.048	5.9E-10	37	48.5
Q9NSD9	Phenylalanine--tRNA ligase beta subunit	1.29	0.072	1.4E-02	3	4.9
P10644	cAMP-dependent protein kinase type I-alpha regulatory subunit	1.29	0.084	5.2E-02	4	12.1
O00231	26S proteasome non-ATPase regulatory subunit 11	1.28	0.246	2.2E-01	5	11.8
Q92841	Probable ATP-dependent RNA helicase DDX17	1.28	0.062	5.4E-03	8	12.8
P56556	NADH dehydrogenase [ubiquinone] 1 alpha subcomplex subunit 6	1.27	NA	NA	2	12.3
P82909	28S ribosomal protein S36, mitochondrial	1.27	0.210	4.9E-01	3	36.9
P30041	Peroxiredoxin-6	1.27	0.148	4.4E-02	7	25.0
Q96GK7	Fumarylacetoacetate hydrolase domain-containing protein 2A	1.27	0.491	4.6E-01	3	13.1
Q9Y394	Dehydrogenase/reductase SDR family member 7	1.26	NA	NA	2	4.4
Q6UVK1	Chondroitin sulfate proteoglycan 4	1.26	0.161	2.1E-01	3	2.7
Q16531	DNA damage-binding protein 1	1.26	NA	NA	2	1.4
P61981	14-3-3 protein gamma	1.26	NA	NA	2	9.7
Q00765	Receptor expression-enhancing protein 5	1.26	NA	NA	2	10.6
Q92597	Protein NDRG1	1.26	1.084	3.5E-01	4	12.9
B5ME19	Eukaryotic translation initiation factor 3 subunit C-like protein	1.25	0.066	2.7E-02	4	3.5
Q9Y4I1	Unconventional myosin-Va	1.25	NA	NA	2	1.0
P36957	Dihydrolipoyllysine-residue succinyltransferase component of 2-oxoglutarate dehydrogenase complex, mitochondrial	1.25	0.042	6.2E-04	5	12.8
Q9UL46	Proteasome activator complex subunit 2	1.25	NA	NA	2	11.3
P30405	Peptidyl-prolyl cis-trans isomerase F, mitochondrial	1.25	0.452	7.5E-01	3	12.6
Q01844	RNA-binding protein EWS	1.25	NA	NA	2	2.3
P27635	60S ribosomal protein L10	1.25	NA	NA	2	9.8
Q9Y5X3	Sorting nexin-5	1.25	NA	NA	2	3.7
P13073	Cytochrome c oxidase subunit 4 isoform 1, mitochondrial	1.25	0.156	2.3E-01	4	25.4
Q13423	NAD(P) transhydrogenase, mitochondrial	1.24	0.155	2.1E-01	11	11.1
P62424	60S ribosomal protein L7a	1.24	0.253	4.1E-01	3	11.7
P18124	60S ribosomal protein L7	1.24	0.060	1.1E-03	4	12.1
P60981	Destrin	1.24	NA	NA	2	11.5
Q86U42	Polyadenylate-binding protein 2	1.23	0.043	4.1E-02	3	9.2
Q13151	Heterogeneous nuclear ribonucleoprotein A0	1.23	0.153	2.2E-01	3	7.5
Q92598	Heat shock protein 105 kDa	1.22	NA	NA	2	2.2
P08559	Pyruvate dehydrogenase E1 component subunit alpha, somatic form, mitochondrial	1.22	NA	NA	2	6.2
P50402	Emerin	1.22	0.061	4.8E-02	5	21.3
Q99584	Protein S100-A13	1.22	0.108	4.2E-02	5	41.8
P23284	Peptidyl-prolyl cis-trans isomerase B	1.22	0.050	4.1E-02	10	41.2
P58876	Histone H2B type 1-D	1.22	NA	NA	2	7.9
P62753	40S ribosomal protein S6	1.22	0.198	3.4E-01	6	16.1
Q9UNZ2	NSFL1 cofactor p47	1.22	NA	NA	2	7.8
P61225	Ras-related protein Rap-2b	1.22	NA	NA	2	9.3
Q9Y230	RuvB-like 2	1.22	NA	NA	2	5.0
P62913	60S ribosomal protein L11	1.21	0.159	2.5E-01	3	16.9
P05388	60S acidic ribosomal protein P0	1.21	NA	NA	2	6.6
P46779	60S ribosomal protein L28	1.21	0.034	8.5E-02	3	19.0
Q9Y3U8	60S ribosomal protein L36	1.20	0.039	1.4E-03	4	30.5
P51572	B-cell receptor-associated protein 31	1.20	0.193	3.3E-01	9	31.7
P62318	Small nuclear ribonucleoprotein Sm D3	1.20	NA	NA	2	24.6
O75340	Programmed cell death protein 6	1.20	NA	NA	2	9.9
Q9Y3E1	Hepatoma-derived growth factor-related protein 3	1.20	0.405	5.9E-01	3	15.8
O43169	Cytochrome b5 type B	1.20	NA	NA	2	21.2
P11216	Glycogen phosphorylase, brain form	1.20	0.226	1.7E-01	8	11.6
Q15293	Reticulocalbin-1	1.19	0.180	2.2E-01	4	10.3
P11586	C-1-tetrahydrofolate synthase, cytoplasmic	1.19	0.100	4.1E-02	5	5.6
P27144	Adenylate kinase 4, mitochondrial	1.19	NA	NA	2	9.9
Q02543	60S ribosomal protein L18a	1.19	0.215	5.3E-01	3	17.0
Q9BR76	Coronin-1B	1.18	NA	NA	2	3.9
P45880	Voltage-dependent anion-selective channel protein 2	1.18	0.131	6.5E-02	8	27.2
P63244	Guanine nucleotide-binding protein subunit beta-2-like 1	1.18	0.223	5.1E-01	4	13.2
Q99623	Prohibitin-2	1.18	0.068	2.2E-02	8	28.4
P62910	60S ribosomal protein L32	1.18	NA	NA	2	21.5
Q9Y3Z3	Deoxynucleoside triphosphate triphosphohydrolase SAMHD1	1.18	0.093	1.0E-01	4	6.5
P13674	Prolyl 4-hydroxylase subunit alpha-1	1.17	0.491	6.0E-01	3	7.3
P62280	40S ribosomal protein S11	1.17	0.135	3.0E-01	4	20.9
P15153	Ras-related C3 botulinum toxin substrate 2	1.17	NA	NA	2	9.4
Q92499	ATP-dependent RNA helicase DDX1	1.17	0.145	1.9E-01	4	5.7
P14927	Cytochrome b-c1 complex subunit 7	1.17	0.136	2.5E-01	5	40.5
P22695	Cytochrome b-c1 complex subunit 2, mitochondrial	1.17	0.242	1.7E-01	6	18.8

Q86UP2	Kinectin	1.17	0.113	9.2E-02	10	9.1
P46778	60S ribosomal protein L21	1.17	NA	NA	2	16.9
Q9Y262	Eukaryotic translation initiation factor 3 subunit L	1.17	0.141	2.4E-01	4	6.6
P62249	40S ribosomal protein S16	1.17	0.046	1.2E-02	3	21.2
Q9UIJ7	GTP:AMP phosphotransferase AK3, mitochondrial	1.16	NA	NA	2	11.0
Q3ZCQ8	Mitochondrial import inner membrane translocase subunit TIM50	1.16	NA	NA	2	5.1
P53618	Coatomer subunit beta	1.16	0.120	4.3E-01	3	4.0
P30519	Heme oxygenase 2	1.16	NA	NA	2	10.1
P30101	Protein disulfide-isomerase A3	1.16	0.098	6.9E-02	14	29.1
Q13242	Serine/arginine-rich splicing factor 9	1.16	NA	NA	2	9.5
O15173	Membrane-associated progesterone receptor component 2	1.15	0.481	5.6E-01	4	22.0
P49327	Fatty acid synthase	1.15	0.193	3.2E-01	4	1.8
Q8IX12	Cell division cycle and apoptosis regulator protein 1	1.15	0.403	4.8E-01	3	3.1
P23396	40S ribosomal protein S3	1.15	0.065	1.4E-02	8	30.0
P07237	Protein disulfide-isomerase	1.14	0.305	4.5E-01	16	27.4
P55145	Mesencephalic astrocyte-derived neurotrophic factor	1.14	NA	NA	2	15.4
Q15029	116 kDa U5 small nuclear ribonucleoprotein component	1.14	1.850	4.9E-01	3	3.7
P31040	Succinate dehydrogenase [ubiquinone] flavoprotein subunit, mitochondrial	1.13	0.313	2.7E-01	4	8.0
P06576	ATP synthase subunit beta, mitochondrial	1.12	0.050	6.4E-02	14	33.5
Q1KMD3	Heterogeneous nuclear ribonucleoprotein U-like protein 2	1.12	0.400	5.4E-01	3	3.1
P50991	T-complex protein 1 subunit delta	1.12	0.264	3.3E-01	4	8.5
Q06830	Peroxiredoxin-1	1.12	0.139	8.4E-02	8	43.2
Q8N1G4	Leucine-rich repeat-containing protein 47	1.12	0.290	4.6E-01	4	8.6
Q15942	Zyxin	1.11	1.037	8.3E-01	3	7.2
P11177	Pyruvate dehydrogenase E1 component subunit beta, mitochondrial	1.11	NA	NA	2	4.2
P62701	40S ribosomal protein S4, X isoform	1.11	0.214	4.5E-01	7	24.0
Q9ULV4	Coronin-1C	1.11	NA	NA	2	4.9
P02774	Vitamin D-binding protein	1.11	0.212	5.6E-01	6	10.1
Q93050	V-type proton ATPase 116 kDa subunit a isoform 1	1.11	NA	NA	2	2.5
O15118	Niemann-Pick C1 protein	1.10	NA	NA	2	1.2
P27824	Calnexin	1.10	1.316	5.6E-01	10	17.4
P18621	60S ribosomal protein L17	1.10	NA	NA	2	10.9
Q86TX2	Acyl-coenzyme A thioesterase 1	1.10	NA	NA	2	5.2
P48643	T-complex protein 1 subunit epsilon	1.10	0.218	3.8E-01	10	15.9
Q13310	Polyadenylate-binding protein 4	1.09	NA	NA	2	4.2
Q562E7	WD repeat-containing protein 81	1.09	NA	NA	2	2.0
P08134	Rho-related GTP-binding protein RhoC	1.09	0.102	2.5E-01	3	10.4
P00403	Cytochrome c oxidase subunit 2	1.09	0.686	5.6E-01	3	14.5
P09874	Poly [ADP-ribose] polymerase 1	1.09	0.084	2.3E-01	8	8.9
Q9NSE4	Isoleucine--tRNA ligase, mitochondrial	1.09	0.434	6.9E-01	5	6.8
P15880	40S ribosomal protein S2	1.09	0.185	5.8E-01	4	13.7
P47985	Cytochrome b-c1 complex subunit Rieske, mitochondrial	1.09	NA	NA	2	8.0
P21281	V-type proton ATPase subunit B, brain isoform	1.09	7.616	5.8E-01	5	11.7
P10515	Dihydrolipoyllysine-residue acetyltransferase component of pyruvate dehydrogenase complex, mitochondrial	1.09	0.107	4.6E-01	4	5.1
Q9UBQ0	Vacuolar protein sorting-associated protein 29	1.09	NA	NA	2	9.3
P42765	3-ketoacyl-CoA thiolase, mitochondrial	1.09	0.605	6.2E-01	4	11.6
Q13561	Dynactin subunit 2	1.08	0.203	5.7E-01	5	14.0
O00203	AP-3 complex subunit beta-1	1.08	NA	NA	2	2.6
Q13488	V-type proton ATPase 116 kDa subunit a isoform 3	1.08	NA	NA	2	3.5
O60262	Guanine nucleotide-binding protein G(I)/G(S)/G(O) subunit gamma-7	1.08	NA	NA	2	30.9
O75165	DnaJ homolog subfamily C member 13	1.08	3.569	8.9E-01	3	1.3
P31689	DnaJ homolog subfamily A member 1	1.07	NA	NA	2	4.8
Q14980	Nuclear mitotic apparatus protein 1	1.07	1.424	5.9E-01	8	4.9
P31930	Cytochrome b-c1 complex subunit 1, mitochondrial	1.07	0.111	2.2E-01	3	7.7
P60228	Eukaryotic translation initiation factor 3 subunit E	1.06	NA	NA	2	3.8
O94826	Mitochondrial import receptor subunit TOM70	1.06	NA	NA	2	4.4
P35998	26S protease regulatory subunit 7	1.06	NA	NA	2	5.8
P28838	Cytosol aminopeptidase	1.06	1.687	8.9E-01	4	8.7
P39023	60S ribosomal protein L3	1.05	0.089	7.0E-01	3	6.7
O60763	General vesicular transport factor p115	1.05	0.551	8.2E-01	3	2.3
P21912	Succinate dehydrogenase [ubiquinone] iron-sulfur subunit, mitochondrial	1.05	0.243	5.0E-01	4	15.0
O75643	U5 small nuclear ribonucleoprotein 200 kDa helicase	1.05	0.613	7.5E-01	4	1.9
O75396	Vesicle-trafficking protein SEC22b	1.05	NA	NA	2	12.6
P78371	T-complex protein 1 subunit beta	1.05	0.232	6.3E-01	3	7.5
Q14165	Malectin	1.04	1.937	5.7E-01	3	11.6
P31943	Heterogeneous nuclear ribonucleoprotein H	1.04	NA	NA	2	3.3
P38606	V-type proton ATPase catalytic subunit A	1.04	0.294	7.0E-01	4	6.6
Q13162	Peroxiredoxin-4	1.04	0.049	3.5E-01	3	12.9
P15311	Ezrin	1.03	0.112	6.1E-01	5	6.5
P24752	Acetyl-CoA acetyltransferase, mitochondrial	1.03	0.103	6.8E-01	5	12.6
P61026	Ras-related protein Rab-10	1.02	NA	NA	2	9.0
Q9UJZ1	Stomatin-like protein 2, mitochondrial	1.02	NA	NA	2	7.3
P35637	RNA-binding protein FUS	1.02	1.923	9.3E-01	5	8.6
P40227	T-complex protein 1 subunit zeta	1.02	0.460	9.0E-01	3	6.6
P62917	60S ribosomal protein L8	1.02	NA	NA	2	7.0
P48735	Isocitrate dehydrogenase [NADP], mitochondrial	1.02	0.178	8.6E-01	6	14.6
Q9P0L0	Vesicle-associated membrane protein-associated protein A	1.01	NA	NA	2	10.0
P26038	Moesin	1.01	0.612	8.7E-01	12	16.8
P24539	ATP synthase F(0) complex subunit B1, mitochondrial	1.01	NA	NA	2	9.0
Q13596	Sorting nexin-1	1.01	NA	NA	2	5.2
P51148	Ras-related protein Rab-5C	1.01	NA	NA	2	10.6
Q99733	Nucleosome assembly protein 1-like 4	1.00	NA	NA	2	5.9
P25705	ATP synthase subunit alpha, mitochondrial	1.00	0.147	9.3E-01	16	30.2
P32969	60S ribosomal protein L9	1.00	0.214	9.7E-01	4	15.1
Q9UQE7	Structural maintenance of chromosomes protein 3	1.00	0.282	1.0E+00	3	3.1
Q99653	Calcineurin B homologous protein 1	1.00	0.101	9.9E-01	3	19.0
P43686	26S protease regulatory subunit 6B	1.00	NA	NA	2	6.0
P55084	Trifunctional enzyme subunit beta, mitochondrial	1.00	1.040	9.6E-01	9	14.6
P01009	Alpha-1-antitrypsin	0.99	0.288	9.4E-01	11	30.9
P62244	40S ribosomal protein S15a	0.99	0.795	9.7E-01	3	18.5
P46109	Crk-like protein	0.99	1.032	9.6E-01	3	16.2
P59998	Actin-related protein 2/3 complex subunit 4	0.98	0.263	8.5E-01	3	16.1
P23919	Thymidylate kinase	0.98	NA	NA	2	13.2
P09496	Clathrin light chain A	0.98	NA	NA	2	5.6
O00264	Membrane-associated progesterone receptor component 1	0.98	NA	NA	2	7.7
O43390	Heterogeneous nuclear ribonucleoprotein R	0.98	0.438	8.0E-01	6	9.3
Q16891	Mitochondrial inner membrane protein	0.98	1.019	7.9E-01	9	14.9
Q9Y512	Sorting and assembly machinery component 50 homolog	0.97	NA	NA	2	5.5
P37235	Hippocalcin-like protein 1	0.97	0.562	9.3E-01	3	16.6
P18077	60S ribosomal protein L35a	0.97	1.562	8.4E-01	3	20.0
P54709	Sodium/potassium-transporting ATPase subunit beta-3	0.97	0.105	7.5E-01	6	20.8
P55884	Eukaryotic translation initiation factor 3 subunit B	0.97	NA	NA	2	2.2
P26196	Probable ATP-dependent RNA helicase DDX6	0.97	NA	NA	2	6.4
P30153	Serine/threonine-protein phosphatase 2A 65 kDa regulatory subunit A alpha isoform	0.96	NA	NA	2	4.8
O95479	GDH/6PGL endoplasmic bifunctional protein	0.96	NA	NA	2	2.7
P01860	Ig gamma-3 chain C region	0.96	0.252	8.1E-01	3	6.4
Q9UHQ9	NADH-cytochrome b5 reductase 1	0.96	0.513	7.0E-01	4	15.1
P36542	ATP synthase subunit gamma, mitochondrial	0.96	0.250	6.4E-01	3	9.4
P40429	60S ribosomal protein L13a	0.96	1.787	8.4E-01	5	23.6
Q02218	2-oxoglutarate dehydrogenase, mitochondrial	0.96	0.140	3.9E-01	8	9.5
P49368	T-complex protein 1 subunit gamma	0.95	0.201	4.8E-01	6	11.6
O94874	E3 UFM1-protein ligase 1	0.95	NA	NA	2	2.8
Q92905	COP9 signalosome complex subunit 5	0.95	NA	NA	2	6.9
P01859	Ig gamma-2 chain C region	0.95	0.077	5.9E-01	3	12.0
O95881	Thioredoxin domain-containing protein 12	0.95	NA	NA	2	14.0

Q92896	Golgi apparatus protein 1	0.94	NA	NA	2	1.7
Q27J81	Inverted formin-2	0.94	0.106	4.9E-01	3	2.5
Q9UNH7	Sorting nexin-6	0.94	0.224	7.7E-01	3	5.2
P51812	Ribosomal protein S6 kinase alpha-3	0.93	NA	NA	2	2.8
P23786	Carnitine O-palmitoyltransferase 2, mitochondrial	0.93	NA	NA	2	3.2
P51798	H(+)/Cl(-) exchange transporter 7	0.93	NA	NA	2	4.0
P61160	Actin-related protein 2	0.93	1.396	6.4E-01	4	14.2
Q02252	Methylmalonate-semialdehyde dehydrogenase [acylating], mitochondrial	0.93	NA	NA	2	3.9
Q02878	60S ribosomal protein L6	0.93	0.195	2.9E-01	7	23.3
P01023	Alpha-2-macroglobulin	0.92	0.123	4.0E-01	7	5.7
P84098	60S ribosomal protein L19	0.92	NA	NA	2	8.7
P62805	Histone H4	0.92	0.107	1.3E-01	7	52.4
P11021	78 kDa glucose-regulated protein	0.92	0.098	1.5E-01	17	26.8
Q93009	Ubiquitin carboxyl-terminal hydrolase 7	0.92	NA	NA	2	1.5
P22061	Protein-L-isoaspartate(D-aspartate) O-methyltransferase	0.92	NA	NA	2	11.5
Q15084	Protein disulfide-isomerase A6	0.91	0.168	3.4E-01	6	15.7
P35222	Catenin beta-1	0.91	0.036	1.6E-01	4	6.1
Q12797	Aspartyl/asparaginyl beta-hydroxylase	0.91	0.212	4.5E-01	6	5.8
Q00325	Phosphate carrier protein, mitochondrial	0.91	0.118	2.7E-01	6	16.0
Q00577	Transcriptional activator protein Pur-alpha	0.91	0.355	6.3E-01	3	6.8
Q7KZF4	Staphylococcal nuclease domain-containing protein 1	0.91	0.745	5.4E-01	4	6.8
P35813	Protein phosphatase 1A	0.91	NA	NA	2	6.8
Q9UNF0	Protein kinase C and casein kinase substrate in neurons protein 2	0.90	0.135	4.2E-01	4	9.1
P46781	40S ribosomal protein S9	0.90	0.546	3.3E-01	6	22.7
Q8NBS9	Thioredoxin domain-containing protein 5	0.90	0.237	6.7E-01	5	13.0
Q99832	T-complex protein 1 subunit eta	0.90	0.131	1.2E-01	8	14.5
P01903	HLA class II histocompatibility antigen, DR alpha chain	0.90	0.223	3.7E-01	4	21.3
Q9UHD8	Septin-9	0.90	0.259	4.9E-01	6	10.8
Q16795	NADH dehydrogenase [ubiquinone] 1 alpha subcomplex subunit 9, mitochondrial	0.89	NA	NA	2	6.4
Q9BS26	Endoplasmic reticulum resident protein 44	0.89	0.101	1.9E-01	4	10.1
O75964	ATP synthase subunit g, mitochondrial	0.89	NA	NA	2	27.2
P50990	T-complex protein 1 subunit theta	0.89	0.075	1.6E-02	11	19.0
Q14697	Neutral alpha-glucosidase AB	0.89	0.134	1.0E-01	11	10.3
P01911	HLA class II histocompatibility antigen, DRB1-15 beta chain	0.88	0.298	7.2E-01	3	9.8
P11310	Medium-chain specific acyl-CoA dehydrogenase, mitochondrial	0.88	0.355	5.0E-01	3	8.3
Q7L5N1	COP9 signalosome complex subunit 6	0.88	NA	NA	2	8.0
P02647	Apolipoprotein A-I	0.88	0.090	8.9E-02	7	25.1
P43304	Glycerol-3-phosphate dehydrogenase, mitochondrial	0.88	0.103	1.5E-01	5	8.0
P61158	Actin-related protein 3	0.88	0.232	3.6E-01	4	9.8
Q9H444	Charged multivesicular body protein 4b	0.88	NA	NA	2	8.9
P02652	Apolipoprotein A-II	0.88	0.780	6.2E-01	3	19.0
P38919	Eukaryotic initiation factor 4A-III	0.87	NA	NA	2	6.3
P35221	Catenin alpha-1	0.87	0.122	1.9E-01	7	8.2
Q14258	E3 ubiquitin/ISG15 ligase TRIM25	0.87	NA	NA	2	3.8
P04632	Calpain small subunit 1	0.87	NA	NA	2	6.3
Q96QK1	Vacuolar protein sorting-associated protein 35	0.86	NA	NA	2	3.1
Q9P2R3	Ankyrin repeat and FYVE domain-containing protein 1	0.86	NA	NA	2	2.7
Q9Y3I0	tRNA-splicing ligase RtcB homolog	0.86	0.178	7.3E-01	3	7.5
P49748	Very long-chain specific acyl-CoA dehydrogenase, mitochondrial	0.86	0.095	3.8E-02	5	7.0
P55072	Transitional endoplasmic reticulum ATPase	0.85	0.046	5.3E-04	13	15.4
P25685	DnaJ homolog subfamily B member 1	0.85	NA	NA	2	5.6
Q6P2Q9	Pre-mRNA-processing-splicing factor 8	0.84	NA	NA	2	0.7
O15511	Actin-related protein 2/3 complex subunit 5	0.84	NA	NA	2	11.9
P41222	Prostaglandin-H2 D-isomerase	0.84	NA	NA	2	12.1
O15144	Actin-related protein 2/3 complex subunit 2	0.84	0.178	2.0E-01	4	12.3
Q9NZ08	Endoplasmic reticulum aminopeptidase 1	0.84	0.470	4.2E-01	3	3.0
Q9HBL7	Plasminogen receptor (KT)	0.83	NA	NA	2	13.6
P46459	Vesicle-fusing ATPase	0.83	0.051	1.9E-02	4	6.0
P17987	T-complex protein 1 subunit alpha	0.83	0.128	6.0E-02	7	14.2
O60716	Catenin delta-1	0.82	0.218	3.3E-01	4	4.9
P29590	Protein PML	0.82	NA	NA	2	2.3
Q13228	Selenium-binding protein 1	0.82	NA	NA	2	4.4
P39656	Dolichyl-diphosphooligosaccharide--protein glycosyltransferase 48 kDa subunit	0.82	0.182	3.3E-01	5	10.1
P46977	Dolichyl-diphosphooligosaccharide--protein glycosyltransferase subunit STT3A	0.82	NA	NA	2	2.3
P49419	Alpha-aminoadipic semialdehyde dehydrogenase	0.82	0.503	5.1E-01	3	7.1
P60953	Cell division control protein 42 homolog	0.81	NA	NA	2	11.0
Q8WVC6	Dephospho-CoA kinase domain-containing protein	0.81	NA	NA	2	10.8
P69905	Hemoglobin subunit alpha	0.81	0.135	1.8E-02	5	34.5
Q9HBL0	Tensin-1	0.80	2.451	4.0E-01	4	3.3
Q16270	Insulin-like growth factor-binding protein 7	0.80	NA	NA	2	10.3
Q13200	26S proteasome non-ATPase regulatory subunit 2	0.80	0.123	1.8E-01	3	3.4
P20700	Lamin-B1	0.80	3.183	3.0E-01	7	13.5
P19367	Hexokinase-1	0.80	NA	NA	2	2.1
P54920	Alpha-soluble NSF attachment protein	0.80	0.122	1.1E-01	5	19.7
P04843	Dolichyl-diphosphooligosaccharide--protein glycosyltransferase subunit 1	0.80	0.120	7.6E-03	12	21.1
P43490	Nicotinamide phosphoribosyltransferase	0.79	NA	NA	2	4.1
O94911	ATP-binding cassette sub-family A member 8	0.79	0.300	5.4E-01	5	3.0
P0C0S8	Histone H2A type 1	0.79	NA	NA	2	23.1
P05107	Integrin beta-2	0.78	NA	NA	2	2.3
P40939	Trifunctional enzyme subunit alpha, mitochondrial	0.78	0.106	4.1E-02	14	19.7
O94832	Unconventional myosin-Id	0.78	0.160	2.0E-01	6	6.6
Q99442	Translocation protein SEC62	0.77	0.036	2.4E-02	3	7.3
P47756	F-actin-capping protein subunit beta	0.77	0.109	4.8E-02	6	23.1
P01857	Ig gamma-1 chain C region	0.77	0.051	8.7E-04	3	13.0
Q9BSD7	Cancer-related nucleoside-triphosphatase	0.77	NA	NA	2	8.9
P50454	Serpin H1	0.76	0.074	8.7E-02	5	14.4
Q09666	Neuroblast differentiation-associated protein AHNAK	0.75	0.038	3.7E-07	91	14.2
O43707	Alpha-actinin-4	0.75	0.045	1.7E-06	21	26.3
P12235	ADP/ATP translocase 1	0.74	NA	NA	2	6.7
P27797	Calreticulin	0.74	0.430	4.2E-01	6	13.9
P48681	Nestin	0.74	0.433	3.8E-01	3	2.4
P53621	Coatomer subunit alpha	0.74	0.100	6.1E-02	5	4.4
P68871	Hemoglobin subunit beta	0.73	0.062	1.1E-04	5	44.9
O94979	Protein transport protein Sec31A	0.73	0.165	1.3E-01	5	5.2
Q16695	Histone H3.1t	0.72	0.066	1.0E-02	5	25.7
P35580	Myosin-10	0.72	0.092	2.6E-03	16	9.2
P00367	Glutamate dehydrogenase 1, mitochondrial	0.72	0.081	1.6E-03	6	12.0
Q14204	Cytoplasmic dynein 1 heavy chain 1	0.72	0.040	1.5E-07	27	6.2
Q14203	Dynactin subunit 1	0.72	0.108	1.6E-01	3	3.1
Q969X5	Endoplasmic reticulum-Golgi intermediate compartment protein 1	0.72	0.228	2.3E-01	3	11.0
Q13283	Ras GTPase-activating protein-binding protein 1	0.71	NA	NA	2	7.3
P10321	HLA class I histocompatibility antigen, Cw-7 alpha chain	0.71	0.206	4.7E-01	4	13.1
P09382	Galectin-1	0.70	0.051	2.2E-04	5	43.7
Q07065	Cytoskeleton-associated protein 4	0.70	0.118	4.4E-02	5	11.0
P16435	NADPH--cytochrome P450 reductase	0.70	NA	NA	2	2.5
P16615	Sarcoplasmic/endoplasmic reticulum calcium ATPase 2	0.69	0.077	5.1E-03	8	8.7
P0C0S5	Histone H2A.Z	0.69	NA	NA	2	18.8
Q9Y639	Neuroplastin	0.69	NA	NA	2	4.8
Q9Y4L1	Hypoxia up-regulated protein 1	0.69	0.077	1.5E-01	4	4.1
P50995	Annexin A11	0.69	0.073	2.8E-03	6	11.1
P06727	Apolipoprotein A-IV	0.69	0.092	6.2E-04	11	25.8
Q86WV6	Stimulator of interferon genes protein	0.68	NA	NA	2	5.8
P17931	Galectin-3	0.68	0.094	2.4E-03	7	31.2
P13667	Protein disulfide-isomerase A4	0.68	0.026	4.4E-02	3	5.0
P08237	ATP-dependent 6-phosphofructokinase, muscle type	0.68	0.018	2.8E-03	3	5.1

P17858	ATP-dependent 6-phosphofructokinase, liver type	0.67	0.078	7.2E-02	4	7.9
Q5SSJ5	Heterochromatin protein 1-binding protein 3	0.67	0.173	5.4E-02	3	7.2
Q8IUX7	Adipocyte enhancer-binding protein 1	0.67	NA	NA	2	2.4
P08107	Heat shock 70 kDa protein 1A/1B	0.66	0.079	1.7E-02	7	12.6
O60313	Dynamin-like 120 kDa protein, mitochondrial	0.66	0.063	3.3E-02	3	4.2
P46939	Utrophin	0.65	0.352	2.5E-01	3	1.0
Q9NTJ5	Phosphatidylinositide phosphatase SAC1	0.65	0.079	2.5E-02	4	6.3
Q03252	Lamin-B2	0.65	0.040	4.7E-09	18	28.0
P08603	Complement factor H	0.65	NA	NA	2	1.5
P01834	Ig kappa chain C region	0.64	0.061	2.0E-02	3	48.1
Q16851	UTP--glucose-1-phosphate uridylyltransferase	0.64	NA	NA	2	3.5
Q8NFW8	N-acylneuraminate cytidylyltransferase	0.63	NA	NA	2	5.1
P00390	Glutathione reductase, mitochondrial	0.63	NA	NA	2	6.3
Q9H4M9	EH domain-containing protein 1	0.63	NA	NA	2	5.1
Q02224	Centromere-associated protein E	0.62	NA	NA	2	0.4
P06899	Histone H2B type 1-J	0.62	NA	NA	2	7.9
Q9BRX8	Redox-regulatory protein FAM213A	0.61	NA	NA	2	8.7
P14625	Endoplasmin	0.61	0.044	2.4E-11	16	20.0
P31942	Heterogeneous nuclear ribonucleoprotein H3	0.61	0.629	5.5E-01	3	12.1
Q9BSJ8	Extended synaptotagmin-1	0.61	0.122	5.0E-02	4	4.8
O00571	ATP-dependent RNA helicase DDX3X	0.61	NA	NA	2	3.9
P04844	Dolichyl-diphosphooligosaccharide--protein glycosyltransferase subunit 2	0.60	0.129	5.4E-02	3	5.4
P26583	High mobility group protein B2	0.59	NA	NA	2	13.9
P13861	cAMP-dependent protein kinase type II-alpha regulatory subunit	0.59	0.052	2.2E-02	3	10.1
P61163	Alpha-centractin	0.58	NA	NA	2	4.5
P49257	Protein ERGIC-53	0.58	NA	NA	2	3.7
P09543	2',3'-cyclic-nucleotide 3'-phosphodiesterase	0.58	0.126	2.7E-02	7	14.0
Q9NZ01	Very-long-chain enoyl-CoA reductase	0.57	0.116	4.5E-01	3	10.4
P67870	Casein kinase II subunit beta	0.57	0.128	1.6E-02	3	12.6
Q96S52	GPI transamidase component PIG-S	0.57	NA	NA	2	5.9
Q05682	Caldesmon	0.56	0.294	6.4E-02	4	6.3
Q53GQ0	Estradiol 17-beta-dehydrogenase 12	0.56	NA	NA	2	8.7
Q6NUK1	Calcium-binding mitochondrial carrier protein SCaMC-1	0.56	NA	NA	2	4.4
O14950	Myosin regulatory light chain 12B	0.56	NA	NA	2	12.2
Q15149	Plectin	0.55	0.028	0.0E+00	53	12.7
Q9BZQ8	Protein Niban	0.55	NA	NA	2	1.9
P67936	Tropomyosin alpha-4 chain	0.55	0.170	7.2E-03	6	22.2
P04217	Alpha-1B-glycoprotein	0.55	0.201	5.2E-02	3	6.7
P04792	Heat shock protein beta-1	0.55	0.058	5.3E-05	7	36.1
P05023	Sodium/potassium-transporting ATPase subunit alpha-1	0.55	0.056	3.6E-06	20	22.5
P60709	Actin, cytoplasmic 1	0.55	0.047	1.1E-07	6	22.9
P60033	CD81 antigen	0.55	NA	NA	2	11.9
O95782	AP-2 complex subunit alpha-1	0.55	0.213	6.0E-02	4	3.8
O75746	Calcium-binding mitochondrial carrier protein Aralar1	0.55	NA	NA	2	4.6
Q14108	Lysosome membrane protein 2	0.54	NA	NA	2	3.8
P40763	Signal transducer and activator of transcription 3	0.54	0.246	1.3E-01	3	4.4
P21589	5'-nucleotidase	0.54	0.145	9.0E-02	3	5.6
P13671	Complement component C6	0.54	0.207	1.4E-01	3	3.7
P07358	Complement component C8 beta chain	0.53	NA	NA	2	3.4
O75131	Copine-3	0.53	0.084	1.4E-03	4	6.7
Q16181	Septin-7	0.53	0.073	1.8E-05	8	17.8
Q15836	Vesicle-associated membrane protein 3	0.52	NA	NA	2	24.0
O43865	Putative adenosylhomocysteinase 2	0.52	NA	NA	2	3.8
P68371	Tubulin beta-4B chain	0.51	0.061	1.3E-06	4	13.3
Q9UEY8	Gamma-adducin	0.50	NA	NA	2	3.8
Q15019	Septin-2	0.50	0.145	1.3E-02	6	22.7
P68363	Tubulin alpha-1B chain	0.50	NA	NA	2	6.7
P04196	Histidine-rich glycoprotein	0.50	0.267	2.3E-02	4	8.4
Q9NQC3	Reticulon-4	0.49	0.049	7.4E-07	3	2.7
Q8WUM4	Programmed cell death 6-interacting protein	0.49	0.071	1.9E-04	6	7.9
P07437	Tubulin beta chain	0.49	0.043	5.8E-12	3	9.9
P11498	Pyruvate carboxylase, mitochondrial	0.49	NA	NA	2	2.5
Q8NEV1	Casein kinase II subunit alpha 3	0.49	NA	NA	2	5.4
P05362	Intercellular adhesion molecule 1	0.49	0.219	7.2E-02	3	5.3
P0CG05	Ig lambda-2 chain C regions	0.49	0.085	4.5E-03	3	46.2
P01042	Kininogen-1	0.48	NA	NA	2	3.0
Q9NVA2	Septin-11	0.48	0.071	1.8E-04	3	7.0
P20073	Annexin A7	0.47	NA	NA	2	5.1
P04899	Guanine nucleotide-binding protein G(i) subunit alpha-2	0.47	0.094	1.6E-02	3	7.6
P05091	Aldehyde dehydrogenase, mitochondrial	0.47	NA	NA	2	4.3
P08133	Annexin A6	0.47	0.050	1.4E-13	29	47.3
Q00610	Clathrin heavy chain 1	0.47	0.040	1.1E-13	24	16.4
P01024	Complement C3	0.46	0.152	3.0E-04	14	8.4
P00387	NADH-cytochrome b5 reductase 3	0.46	0.082	3.2E-05	7	27.2
Q9HDC9	Adipocyte plasma membrane-associated protein	0.46	NA	NA	2	7.5
P04040	Catalase	0.46	NA	NA	2	4.7
O94919	Endonuclease domain-containing 1 protein	0.45	NA	NA	2	4.8
P00352	Retinal dehydrogenase 1	0.45	0.023	6.9E-03	5	10.2
P05556	Integrin beta-1	0.45	0.092	6.5E-06	6	8.8
Q14764	Major vault protein	0.45	0.113	8.5E-03	3	3.8
Q9UHG3	Prenylcysteine oxidase 1	0.45	0.167	4.3E-02	4	7.9
P60660	Myosin light polypeptide 6	0.45	0.043	7.8E-15	8	58.3
P17612	cAMP-dependent protein kinase catalytic subunit alpha	0.44	NA	NA	2	4.3
O95865	N(G),N(G)-dimethylarginine dimethylaminohydrolase 2	0.44	NA	NA	2	8.4
Q9Y490	Talin-1	0.44	0.037	0.0E+00	26	13.9
P04114	Apolipoprotein B-100	0.43	NA	NA	2	0.3
P43121	Cell surface glycoprotein MUC18	0.43	0.146	7.4E-02	3	5.1
Q9Y4G6	Talin-2	0.43	NA	NA	2	1.3
Q07954	Prolow-density lipoprotein receptor-related protein 1	0.42	0.101	1.2E-02	4	1.3
Q14344	Guanine nucleotide-binding protein subunit alpha-13	0.42	0.302	5.0E-02	5	14.3
P23634	Plasma membrane calcium-transporting ATPase 4	0.42	0.139	6.1E-03	3	3.9
Q01995	Transgelin	0.42	0.113	4.7E-03	5	24.9
P12814	Alpha-actinin-1	0.41	0.077	2.2E-04	11	15.9
Q14847	LIM and SH3 domain protein 1	0.40	3.677	7.9E-01	3	8.8
P02452	Collagen alpha-1(I) chain	0.39	0.346	3.4E-01	3	2.6
Q9NYL9	Tropomodulin-3	0.39	NA	NA	2	7.4
P62873	Guanine nucleotide-binding protein G(I)/G(S)/G(T) subunit beta-1	0.38	NA	NA	2	9.4
Q13642	Four and a half LIM domains protein 1	0.36	NA	NA	2	8.0
P08571	Monocyte differentiation antigen CD14	0.36	NA	NA	2	5.3
P11413	Glucose-6-phosphate 1-dehydrogenase	0.35	NA	NA	2	3.9
A8MTJ3	Guanine nucleotide-binding protein G(t) subunit alpha-3	0.31	NA	NA	2	5.4
Q6DD88	Atlastin-3	0.29	0.194	1.9E-01	3	5.7
P14136	Glial fibrillary acidic protein	0.29	NA	NA	2	4.6
Q9BS40	Latexin	0.29	NA	NA	2	12.2
P05026	Sodium/potassium-transporting ATPase subunit beta-1	0.29	0.453	1.2E-01	3	12.2
P63010	AP-2 complex subunit beta	0.29	NA	NA	2	2.3
Q13425	Beta-2-syntrophin	0.28	NA	NA	2	2.6
P09936	Ubiquitin carboxyl-terminal hydrolase isozyme L1	0.24	0.662	2.0E-01	3	13.9
Q03135	Caveolin-1	0.24	NA	NA	2	13.5
Q14699	Raftlin	0.23	NA	NA	2	2.1
P05787	Keratin, type II cytoskeletal 8	0.23	NA	NA	2	3.3
P60201	Myelin proteolipid protein	0.23	NA	NA	2	7.6
P61626	Lysozyme C	0.23	NA	NA	2	12.8
P29992	Guanine nucleotide-binding protein subunit alpha-11	0.22	NA	NA	2	6.1
P17661	Desmin	0.22	NA	NA	2	3.4

P16157	Ankyrin-1	0.17	NA	NA	2	1.5
P05186	Alkaline phosphatase, tissue-nonspecific isozyme	0.17	NA	NA	2	3.4
P02462	Collagen alpha-1(IV) chain	0.17	NA	NA	2	1.4
Q9UBX5	Fibulin-5	0.17	NA	NA	2	3.6
P02654	Apolipoprotein C-I	0.16	NA	NA	2	24.1
P11277	Spectrin beta chain, erythrocytic	0.15	0.327	1.2E-01	5	3.4
Q14624	Inter-alpha-trypsin inhibitor heavy chain H4	0.15	NA	NA	2	1.9
O00468	Agrin	0.15	NA	NA	2	1.2
P24844	Myosin regulatory light polypeptide 9	0.14	NA	NA	2	12.2
P58166	Inhibin beta E chain	0.14	0.304	1.2E-01	3	10.0
P05164	Myeloperoxidase	0.12	NA	NA	2	3.0
P43320	Beta-crystallin B2	0.12	NA	NA	2	11.7
P23946	Chymase	0.10	0.326	6.6E-02	3	15.0
Q9BXN1	Asporin	0.09	NA	NA	2	5.5
P32119	Peroxiredoxin-2	0.40	0.084	1.1E-05	4	18.2
Q969G5	Protein kinase C delta-binding protein	0.38	0.144	3.1E-02	3	11.5
O00159	Unconventional myosin-Ic	0.37	0.075	1.1E-05	10	9.4
P18206	Vinculin	0.37	0.094	6.5E-07	12	14.0
O94905	Erlin-2	0.36	0.118	4.1E-03	6	15.6
P00738	Haptoglobin	0.36	0.093	4.5E-05	5	13.1
Q6NZI2	Polymerase I and transcript release factor	0.36	0.108	5.8E-05	7	18.7
O43301	Heat shock 70 kDa protein 12A	0.34	0.183	1.2E-02	4	7.7
P02511	Alpha-crystallin B chain	0.33	0.161	5.5E-03	6	35.4
P00450	Ceruloplasmin	0.33	0.110	1.7E-07	10	11.4
Q15582	Transforming growth factor-beta-induced protein ig-h3	0.33	0.097	8.0E-03	3	5.1
P02749	Beta-2-glycoprotein 1	0.32	0.094	7.2E-03	4	12.5
Q9BUF5	Tubulin beta-6 chain	0.32	0.160	1.5E-04	3	7.0
Q13813	Spectrin alpha chain, non-erythrocytic 1	0.32	0.030	0.0E+00	72	31.5
P0C0L5	Complement C4-B	0.32	0.159	2.4E-04	7	3.2
P35579	Myosin-9	0.32	0.033	0.0E+00	54	26.6
Q9Y6N5	Sulfide:quinone oxidoreductase, mitochondrial	0.32	0.028	1.1E-04	3	7.3
Q01082	Spectrin beta chain, non-erythrocytic 1	0.32	0.043	0.0E+00	50	24.6
P09493	Tropomyosin alpha-1 chain	0.31	0.086	2.6E-07	6	15.1
P46821	Microtubule-associated protein 1B	0.30	0.213	3.2E-02	4	2.0
P12111	Collagen alpha-3(VI) chain	0.29	0.054	0.0E+00	37	12.2
P07355	Annexin A2	0.29	0.033	0.0E+00	25	56.9
P00747	Plasminogen	0.29	0.099	2.5E-05	6	9.1
P12110	Collagen alpha-2(VI) chain	0.29	0.065	5.2E-10	7	7.7
P07197	Neurofilament medium polypeptide	0.28	0.246	1.4E-02	6	5.2
Q9NZM1	Myoferlin	0.28	0.227	3.2E-03	8	4.3
P80723	Brain acid soluble protein 1	0.27	0.222	2.7E-02	5	33.0
P50895	Basal cell adhesion molecule	0.27	0.215	3.8E-03	3	6.7
Q02952	A-kinase anchor protein 12	0.26	0.138	6.0E-05	17	13.4
P55268	Laminin subunit beta-2	0.26	0.127	7.0E-07	13	8.2
P08294	Extracellular superoxide dismutase [Cu-Zn]	0.26	0.102	1.5E-03	3	15.4
P00167	Cytochrome b5	0.26	0.096	7.4E-03	3	35.8
P12109	Collagen alpha-1(VI) chain	0.25	0.068	1.1E-07	10	10.4
O75369	Filamin-B	0.24	0.107	1.5E-04	6	2.9
P60903	Protein S100-A10	0.24	0.187	1.7E-03	3	35.1
O43491	Band 4.1-like protein 2	0.24	0.073	7.7E-03	10	14.4
P27105	Erythrocyte band 7 integral membrane protein	0.24	0.146	2.1E-05	6	20.1
O94875	Sorbin and SH3 domain-containing protein 2	0.24	0.183	2.7E-03	3	3.3
P02751	Fibronectin	0.24	0.096	1.2E-07	12	7.2
P07099	Epoxide hydrolase 1	0.23	0.066	2.8E-08	6	13.0
P06396	Gelsolin	0.23	0.125	4.2E-07	9	11.5
P68032	Actin, alpha cardiac muscle 1	0.23	0.127	2.0E-08	6	21.0
O15230	Laminin subunit alpha-5	0.23	0.121	1.2E-05	14	4.6
P01011	Alpha-1-antichymotrypsin	0.22	0.132	7.9E-06	6	12.8
P21333	Filamin-A	0.22	0.045	0.0E+00	51	26.0
Q9Y6C2	EMILIN-1	0.22	0.289	5.4E-03	5	6.5
P02549	Spectrin alpha chain, erythrocytic 1	0.22	0.319	1.7E-03	6	3.8
P39060	Collagen alpha-1(XVIII) chain	0.22	0.118	2.8E-08	9	5.0
P08572	Collagen alpha-2(IV) chain	0.21	0.125	1.5E-07	6	4.6
P02686	Myelin basic protein	0.21	0.088	4.4E-06	3	11.2
Q14112	Nidogen-2	0.20	0.132	9.9E-07	8	6.7
P98160	Basement membrane-specific heparan sulfate proteoglycan core protein	0.19	0.074	0.0E+00	24	6.7
Q9BXM0	Periaxin	0.19	0.240	2.0E-04	6	1.6
P22105	Tenascin-X	0.19	0.190	2.5E-02	4	1.0
P07942	Laminin subunit beta-1	0.19	0.109	5.2E-04	3	1.6
P14555	Phospholipase A2, membrane associated	0.19	0.238	1.5E-02	4	23.6
P02679	Fibrinogen gamma chain	0.19	0.135	3.2E-08	10	22.5
P11166	Solute carrier family 2, facilitated glucose transporter member 1	0.19	0.190	1.1E-03	3	5.5
Q16555	Dihydropyrimidinase-related protein 2	0.18	0.125	3.4E-09	10	22.0
P11047	Laminin subunit gamma-1	0.18	0.131	5.4E-06	9	5.2
Q05707	Collagen alpha-1(XIV) chain	0.18	0.319	6.2E-04	8	4.7
Q16363	Laminin subunit alpha-4	0.18	0.160	1.5E-02	4	1.9
Q12805	EGF-containing fibulin-like extracellular matrix protein 1	0.17	0.065	1.7E-03	3	5.7
P02649	Apolipoprotein E	0.17	0.111	9.1E-05	13	44.5
P14543	Nidogen-1	0.16	0.132	1.5E-08	9	8.5
P36269	Gamma-glutamyltransferase 5	0.16	0.185	2.4E-04	4	7.3
P35749	Myosin-11	0.15	0.076	1.3E-15	32	17.8
P01871	Ig mu chain C region	0.15	0.122	6.2E-05	7	18.8
P02675	Fibrinogen beta chain	0.15	0.090	6.9E-14	11	29.7
P39059	Collagen alpha-1(XV) chain	0.14	0.105	5.0E-08	5	3.5
P41219	Peripherin	0.13	0.144	6.8E-08	12	24.9
P04083	Annexin A1	0.13	0.074	4.4E-16	11	29.8
P10745	Retinol-binding protein 3	0.13	0.197	3.8E-03	4	3.0
P02760	Protein AMBP	0.12	0.151	9.7E-06	4	19.0
P15088	Mast cell carboxypeptidase A	0.11	0.384	3.6E-03	5	12.0
Q15661	Tryptase alpha/beta-1	0.11	0.165	6.9E-09	6	21.8
P04275	von Willebrand factor	0.11	0.127	1.4E-09	14	5.3
P01008	Antithrombin-III	0.11	0.226	2.1E-04	6	12.9
P51888	Prolargin	0.11	0.095	0.0E+00	12	34.3
P21926	CD9 antigen	0.10	0.131	6.2E-04	3	9.6
P22352	Glutathione peroxidase 3	0.10	0.130	2.0E-03	3	11.5
P07585	Decorin	0.10	0.142	1.6E-04	7	18.7
P21980	Protein-glutamine gamma-glutamyltransferase 2	0.10	0.134	1.9E-10	16	21.3
P26447	Protein S100-A4	0.10	0.120	7.3E-06	3	27.7
P02671	Fibrinogen alpha chain	0.10	0.140	7.5E-09	9	12.4
P08123	Collagen alpha-2(I) chain	0.10	0.081	1.2E-07	3	3.7
P35625	Metalloproteinase inhibitor 3	0.09	0.168	4.1E-08	5	24.6
Q14195	Dihydropyrimidinase-related protein 3	0.09	0.148	3.4E-07	8	18.1
P20774	Mimecan	0.09	0.116	1.4E-11	8	24.8
P02730	Band 3 anion transport protein	0.09	0.180	7.5E-06	8	11.3
P10909	Clusterin	0.09	0.088	0.0E+00	13	26.9
P35555	Fibrillin-1	0.09	0.057	0.0E+00	45	16.7
P51884	Lumican	0.08	0.071	0.0E+00	9	26.0
P02748	Complement component C9	0.08	0.161	1.4E-06	8	13.2
P21810	Biglycan	0.07	0.113	4.4E-16	11	31.5
P01031	Complement C5	0.07	0.242	8.9E-05	5	3.0
P04004	Vitronectin	0.06	0.101	0.0E+00	10	15.9
P02743	Serum amyloid P-component	0.06	0.158	5.1E-08	7	27.8
P25189	Myelin protein P0	0.05	0.085	1.6E-09	7	27.4
P22748	Carbonic anhydrase 4	0.05	0.203	1.7E-07	7	22.8

Brown denotes change ≥ 2 standard deviations (SD) from the mean, yellow denotes change ≥ 1 SD and green highlights p values ≤ 0.05. NA, not applicable, n<3 unique peptides.
