# Supplementary material for: iTRAQ Quantitative Proteomic Comparison of Metastatic and Non-Metastatic Uveal Melanoma Tumors
Source: PLoS One. 2015 Aug 25;10(8):e0135543. doi: 10.1371/journal.pone.0135543 (PMC4549237; doi:10.1371/journal.pone.0135543)
Supplement: S6 Table — (PDF) [file pone.0135543.s006.pdf]

Supplementary Table S6
Relative Protein Abundance: Sample UM13, Non-Metastatic
Total Proteins Quantified = 883; LogMedian Protein Ratio = 0.20; LogMean Protein Ratio = 0; Standard Deviation = 0.98

Uni-Prot Accession	Protein	Ratio UM/Control	Standard Deviation	*p* value	Unique Peptides	% Sequence Coverage
P10599	Thioredoxin	10.93	0.100	2.2E-11	5	51.4
P62937	Peptidyl-prolyl cis-trans isomerase A	9.65	0.088	4.4E-16	11	70.9
P30086	Phosphatidylethanolamine-binding protein 1	9.47	0.114	2.8E-13	11	65.8
P23297	Protein S100-A1	7.79	0.315	2.2E-05	3	41.5
P00338	L-lactate dehydrogenase A chain	7.39	0.116	2.9E-13	12	30.7
P07108	Acyl-CoA-binding protein	6.09	0.179	1.4E-03	4	62.1
P22087	rRNA 2'-O-methyltransferase fibrillarin	6.03	0.123	1.4E-04	5	19.0
P60174	Triosephosphate isomerase	6.01	0.101	5.3E-06	15	59.1
P61604	10 kDa heat shock protein, mitochondrial	5.77	0.105	4.2E-06	7	62.7
P07900	Heat shock protein HSP 90-alpha	5.76	0.068	0.0E+00	20	23.6
P04406	Glyceraldehyde-3-phosphate dehydrogenase	5.28	0.057	2.5E-12	16	47.2
P08238	Heat shock protein HSP 90-beta	5.09	0.094	5.3E-12	18	20.7
P16402	Histone H1.3	5.06	0.084	6.3E-08	6	15.4
P04080	Cystatin-B	4.99	0.107	3.1E-08	4	55.1
P17096	High mobility group protein HMG-I/HMG-Y	4.92	0.245	1.7E-02	3	23.4
Q8NCW5	NAD(P)H-hydrate epimerase	4.90	0.284	1.2E-02	3	10.8
P16070	CD44 antigen	4.89	0.108	3.2E-09	10	13.2
P00558	Phosphoglycerate kinase 1	4.77	0.089	5.1E-14	17	44.6
Q04760	Lactoylglutathione lyase	4.59	0.134	5.9E-06	6	22.8
P15531	Nucleoside diphosphate kinase A	4.58	0.111	7.4E-08	7	44.1
P07195	L-lactate dehydrogenase B chain	4.52	0.109	5.3E-06	7	18.3
P31948	Stress-induced-phosphoprotein 1	4.45	0.071	1.5E-12	18	29.7
P63241	Eukaryotic translation initiation factor 5A-1	4.29	0.135	4.4E-04	7	35.7
P0DME0	Protein SETSIP	4.26	0.172	4.8E-04	5	20.2
P55769	NHP2-like protein 1	4.09	0.128	2.3E-04	3	24.2
P99999	Cytochrome c	3.93	0.135	8.1E-08	6	60.0
P20042	Eukaryotic translation initiation factor 2 subunit 2	3.82	0.197	1.4E-02	4	13.5
P23528	Cofilin-1	3.78	0.173	2.7E-04	10	44.6
P22234	Multifunctional protein ADE2	3.76	0.261	1.5E-02	3	6.4
P58546	Myotrophin	3.65	0.074	4.5E-03	3	31.4
P08758	Annexin A5	3.63	0.046	0.0E+00	21	70.0
P06733	Alpha-enolase	3.62	0.075	3.1E-12	16	48.8
P78417	Glutathione S-transferase omega-1	3.61	0.096	3.8E-06	9	36.1
P23526	Adenosylhomocysteinase	3.58	0.158	6.7E-04	7	17.6
P30044	Peroxiredoxin-5, mitochondrial	3.48	0.114	2.9E-04	5	23.4
P40926	Malate dehydrogenase, mitochondrial	3.46	0.057	3.1E-06	16	58.6
Q13185	Chromobox protein homolog 3	3.41	0.185	1.3E-02	3	18.0
P24941	Cyclin-dependent kinase 2	3.41	0.145	1.6E-04	7	27.9
P62750	60S ribosomal protein L23a	3.40	0.058	1.3E-05	5	27.6
P63104	14-3-3 protein zeta/delta	3.40	0.123	1.1E-03	8	42.4
P11766	Alcohol dehydrogenase class-3	3.38	0.229	3.8E-02	3	7.0
O75368	SH3 domain-binding glutamic acid-rich-like protein	3.32	0.198	6.3E-04	4	31.6
P19338	Nucleolin	3.28	0.065	2.0E-12	18	25.6
P40925	Malate dehydrogenase, cytoplasmic	3.21	0.156	9.9E-04	6	21.9
P38646	Stress-70 protein, mitochondrial	3.20	0.058	5.5E-11	24	39.5
Q99497	Protein DJ-1	3.20	0.320	2.2E-02	5	24.3
P12955	Xaa-Pro dipeptidase	3.19	0.192	1.6E-02	6	12.2
O15400	Syntaxin-7	3.16	0.131	7.7E-05	5	25.3
P07737	Profilin-1	3.16	0.160	2.7E-04	6	47.9
P31937	3-hydroxyisobutyrate dehydrogenase, mitochondrial	3.13	0.356	1.7E-02	5	18.2
Q99798	Aconitate hydratase, mitochondrial	3.08	0.095	7.0E-04	12	22.4
P16949	Stathmin	3.07	0.165	9.4E-04	3	24.2
P51858	Hepatoma-derived growth factor	3.00	0.179	1.4E-03	5	30.4
P49189	4-trimethylaminobutyraldehyde dehydrogenase	3.00	0.202	1.6E-03	4	8.1
P29401	Transketolase	2.96	0.113	2.8E-03	13	22.8
P57729	Ras-related protein Rab-38	2.95	0.173	5.5E-04	4	22.3
Q9Y2S2	Lambda-crystallin homolog	2.95	0.278	3.2E-02	6	19.7
P30043	Flavin reductase (NADPH)	2.94	0.207	9.1E-03	3	18.9
P13797	Plastin-3	2.93	0.105	7.3E-06	10	17.6
Q9BYT8	Neurolysin, mitochondrial	2.92	0.110	4.3E-03	4	7.8
P62826	GTP-binding nuclear protein Ran	2.88	0.113	2.6E-02	5	24.5
Q96I99	Succinyl-CoA ligase [GDP-forming] subunit beta, mitochondrial	2.82	0.070	2.1E-07	6	14.6
P21266	Glutathione S-transferase Mu 3	2.78	0.085	2.7E-07	6	29.3
Q16836	Hydroxyacyl-coenzyme A dehydrogenase, mitochondrial	2.73	0.138	2.2E-03	4	18.2
P30837	Aldehyde dehydrogenase X, mitochondrial	2.72	0.131	9.6E-07	10	26.5
P52565	Rho GDP-dissociation inhibitor 1	2.70	0.227	4.3E-03	4	22.5
P14174	Macrophage migration inhibitory factor	18.58	NA	NA	2	17.4
P06454	Prothymosin alpha	13.55	0.313	1.9E-01	3	21.6
P35270	Sepiapterin reductase	6.67	NA	NA	2	10.0
O75531	Barrier-to-autointegration factor	5.07	0.616	2.6E-01	3	42.7
O75347	Tubulin-specific chaperone A	4.74	0.638	7.5E-02	3	26.9
O60888	Protein CutA	3.96	NA	NA	2	15.6
Q15819	Ubiquitin-conjugating enzyme E2 variant 2	3.60	NA	NA	2	13.1
P52815	39S ribosomal protein L12, mitochondrial	3.10	NA	NA	2	11.6
Q6PI48	Aspartate--tRNA ligase, mitochondrial	3.07	0.260	5.7E-02	4	6.5
P10768	S-formylglutathione hydrolase	3.00	NA	NA	2	7.8
P00915	Carbonic anhydrase 1	2.99	NA	NA	2	8.0
P09429	High mobility group protein B1	2.86	0.116	1.0E-01	6	27.0
O43598	2'-deoxynucleoside 5'-phosphate N-hydrolase 1	2.82	NA	NA	2	18.4
Q9UQ80	Proliferation-associated protein 2G4	2.82	NA	NA	2	6.3
P35613	Basigin	2.82	NA	NA	2	8.3
Q5JNZ5	Putative 40S ribosomal protein S26-like 1	2.80	NA	NA	2	20.9
Q9Y285	Phenylalanine--tRNA ligase alpha subunit	2.77	NA	NA	2	8.7
Q9H0R4	Haloacid dehalogenase-like hydrolase domain-containing protein 2	2.72	NA	NA	2	8.1
P06748	Nucleophosmin	2.67	0.113	2.8E-05	7	27.9
P50453	Serpin B9	2.67	0.190	1.4E-02	7	22.9
O43399	Tumor protein D54	2.66	0.084	9.8E-04	5	39.3
O95834	Echinoderm microtubule-associated protein-like 2	2.66	NA	NA	2	5.5
Q9BUP0	EF-hand domain-containing protein D1	2.65	0.351	9.8E-02	3	10.9
P60842	Eukaryotic initiation factor 4A-I	2.64	0.258	7.2E-03	3	8.1
P06744	Glucose-6-phosphate isomerase	2.63	0.119	2.4E-05	13	29.4
P15121	Aldose reductase	2.59	0.151	3.1E-02	3	11.4
P12830	Cadherin-1	2.58	NA	NA	2	2.4
Q99729	Heterogeneous nuclear ribonucleoprotein A/B	2.52	0.149	9.7E-04	3	6.9
P00441	Superoxide dismutase [Cu-Zn]	2.51	0.358	4.1E-02	3	22.1
Q07955	Serine/arginine-rich splicing factor 1	2.49	0.273	5.7E-02	3	8.5
Q92597	Protein NDRG1	2.48	0.200	4.9E-03	8	30.5
P62861	40S ribosomal protein S30	2.48	NA	NA	2	18.6
Q9Y2X3	Nucleolar protein 58	2.46	0.339	9.5E-02	6	16.1
P30084	Enoyl-CoA hydratase, mitochondrial	2.43	0.765	1.8E-01	5	22.1
Q9NSE4	Isoleucine--tRNA ligase, mitochondrial	2.43	0.085	2.8E-05	9	12.0
Q00796	Sorbitol dehydrogenase	2.43	0.129	3.7E-02	3	4.8
P07954	Fumarate hydratase, mitochondrial	2.43	NA	NA	2	4.3
P61088	Ubiquitin-conjugating enzyme E2 N	2.42	0.395	2.0E-01	4	28.3
P16219	Short-chain specific acyl-CoA dehydrogenase, mitochondrial	2.42	0.102	6.5E-03	3	9.7
Q92820	Gamma-glutamyl hydrolase	2.41	0.269	5.0E-02	3	9.1
Q04837	Single-stranded DNA-binding protein, mitochondrial	2.41	0.292	2.1E-01	3	23.6
E9PAV3	Nascent polypeptide-associated complex subunit alpha, muscle-specific form	2.41	0.219	4.4E-02	3	2.0

P62258	14-3-3 protein epsilon	2.40	0.143	4.8E-04	10	36.9
O00567	Nucleolar protein 56	2.39	0.665	1.8E-01	4	9.3
P30042	ES1 protein homolog, mitochondrial	2.38	0.133	3.3E-02	4	18.7
P50395	Rab GDP dissociation inhibitor beta	2.37	0.276	3.7E-02	8	24.0
O14556	Glyceraldehyde-3-phosphate dehydrogenase, testis-specific	2.37	0.163	9.3E-03	4	12.5
P49773	Histidine triad nucleotide-binding protein 1	2.37	0.536	7.7E-02	4	53.2
P05413	Fatty acid-binding protein, heart	2.37	NA	NA	2	15.8
Q9UL12	Sarcosine dehydrogenase, mitochondrial	2.37	NA	NA	2	2.6
Q15293	Reticulocalbin-1	2.36	0.095	5.5E-03	7	19.0
Q00059	Transcription factor A, mitochondrial	2.35	0.112	5.9E-04	7	25.2
P0CW22	40S ribosomal protein S17-like	2.35	0.225	6.0E-02	3	16.3
P16401	Histone H1.5	2.35	0.125	1.1E-03	4	15.0
P63244	Guanine nucleotide-binding protein subunit beta-2-like 1	2.34	0.183	6.9E-03	6	20.5
P30405	Peptidyl-prolyl cis-trans isomerase F, mitochondrial	2.34	NA	NA	2	8.7
P53985	Monocarboxylate transporter 1	2.34	0.260	1.2E-01	3	6.6
Q13838	Spliceosome RNA helicase DDX39B	2.33	0.147	1.3E-03	8	21.3
Q8NC51	Plasminogen activator inhibitor 1 RNA-binding protein	2.33	0.597	3.7E-01	4	12.3
P10809	60 kDa heat shock protein, mitochondrial	2.32	0.081	2.6E-04	20	40.0
Q8NFV4	Alpha/beta hydrolase domain-containing protein 11	2.32	NA	NA	2	8.6
P09211	Glutathione S-transferase P	2.31	0.184	9.4E-02	8	57.1
P21291	Cysteine and glycine-rich protein 1	2.30	0.159	2.5E-03	3	21.8
P30040	Endoplasmic reticulum resident protein 29	2.30	NA	NA	2	8.4
P27348	14-3-3 protein theta	2.28	NA	NA	2	13.5
P30041	Peroxiredoxin-6	2.27	0.065	1.1E-06	9	37.9
Q9Y4W6	AFG3-like protein 2	2.26	0.103	2.7E-03	9	12.2
P30049	ATP synthase subunit delta, mitochondrial	2.26	NA	NA	2	13.7
P04792	Heat shock protein beta-1	2.25	0.089	5.5E-06	8	55.1
Q13177	Serine/threonine-protein kinase PAK 2	2.25	NA	NA	2	5.9
P13693	Translationally-controlled tumor protein	2.20	NA	NA	2	15.7
P27695	DNA-(apurinic or apyrimidinic site) lyase	2.19	NA	NA	2	7.2
Q99536	Synaptic vesicle membrane protein VAT-1 homolog	2.19	0.049	5.9E-07	13	47.8
P07910	Heterogeneous nuclear ribonucleoproteins C1/C2	2.18	0.135	1.4E-04	8	26.1
P05387	60S acidic ribosomal protein P2	2.17	0.116	1.5E-05	6	70.4
P42766	60S ribosomal protein L35	2.17	0.080	1.8E-02	3	18.7
P06753	Tropomyosin alpha-3 chain	2.17	0.111	3.6E-05	4	13.0
P13929	Beta-enolase	2.17	NA	NA	2	6.7
Q9NPJ3	Acyl-coenzyme A thioesterase 13	2.17	NA	NA	2	15.7
O75390	Citrate synthase, mitochondrial	2.16	0.071	4.8E-06	8	17.6
Q15185	Prostaglandin E synthase 3	2.16	NA	NA	2	9.4
Q86UE4	Protein LYRIC	2.16	0.222	9.7E-03	3	5.8
P08195	4F2 cell-surface antigen heavy chain	2.15	0.104	3.4E-05	8	16.5
O95336	6-phosphogluconolactonase	2.14	0.238	5.4E-02	4	20.9
Q92688	Acidic leucine-rich nuclear phosphoprotein 32 family member B	2.14	0.135	2.3E-03	4	17.1
P08107	Heat shock 70 kDa protein 1A/1B	2.13	0.046	1.0E-07	16	28.7
P30740	Leukocyte elastase inhibitor	2.13	0.121	2.8E-02	5	14.0
Q9HC38	Glyoxalase domain-containing protein 4	2.12	0.119	2.3E-02	3	8.0
P13639	Elongation factor 2	2.12	0.086	5.0E-07	17	22.7
Q96KP4	Cytosolic non-specific dipeptidase	2.11	0.065	1.7E-03	4	11.8
Q5JRX3	Presequence protease, mitochondrial	2.10	0.184	9.5E-03	5	6.9
P21283	V-type proton ATPase subunit C 1	2.08	0.144	1.7E-02	4	9.9
Q13126	S-methyl-5'-thioadenosine phosphorylase	2.05	NA	NA	2	7.8
P02042	Hemoglobin subunit delta	2.05	NA	NA	2	21.8
Q9UH65	Switch-associated protein 70	2.05	0.492	1.9E-01	3	3.2
O76021	Ribosomal L1 domain-containing protein 1	2.04	NA	NA	2	4.3
Q13442	28 kDa heat- and acid-stable phosphoprotein	2.04	0.124	1.4E-01	3	23.2
P02768	Serum albumin	2.04	0.039	5.8E-11	42	63.7
Q07020	60S ribosomal protein L18	2.03	0.075	6.6E-05	3	19.7
Q53EL6	Programmed cell death protein 4	2.03	0.360	4.8E-01	5	13.6
P42704	Leucine-rich PPR motif-containing protein, mitochondrial	2.03	0.114	3.4E-04	14	10.8
O00217	NADH dehydrogenase [ubiquinone] iron-sulfur protein 8, mitochondrial	2.03	NA	NA	2	9.5
Q5VWZ2	Lysophospholipase-like protein 1	2.02	NA	NA	2	8.0
Q86VP6	Cullin-associated NEDD8-dissociated protein 1	2.02	0.089	7.0E-05	7	6.2
Q07021	Complement component 1 Q subcomponent-binding protein, mitochondrial	2.01	0.107	1.8E-02	4	21.6
Q12906	Interleukin enhancer-binding factor 3	2.01	0.081	1.2E-05	9	11.1
Q53H82	Beta-lactamase-like protein 2	2.01	NA	NA	2	9.4
Q9BRA2	Thioredoxin domain-containing protein 17	2.00	0.397	2.2E-01	3	26.8
P12236	ADP/ATP translocase 3	2.00	NA	NA	2	7.0
P55010	Eukaryotic translation initiation factor 5	2.00	0.336	2.9E-01	3	6.7
Q14103	Heterogeneous nuclear ribonucleoprotein D0	1.99	0.085	2.0E-03	4	15.5
P53597	Succinyl-CoA ligase [ADP/GDP-forming] subunit alpha, mitochondrial	1.99	0.237	5.2E-02	6	21.4
O43678	NADH dehydrogenase [ubiquinone] 1 alpha subcomplex subunit 2	1.99	NA	NA	2	11.1
Q5VTE0	Putative elongation factor 1-alpha-like 3	1.99	0.101	4.0E-04	10	24.0
P48147	Prolyl endopeptidase	1.98	NA	NA	2	3.7
Q92616	Translational activator GCN1	1.98	0.201	5.0E-02	4	2.2
P09622	Dihydrolipoyl dehydrogenase, mitochondrial	1.97	0.103	1.8E-02	6	12.6
Q14974	Importin subunit beta-1	1.96	0.098	1.3E-02	8	12.1
P26641	Elongation factor 1-gamma	1.95	0.076	1.1E-04	6	14.4
P20618	Proteasome subunit beta type-1	1.94	NA	NA	2	9.5
Q13423	NAD(P) transhydrogenase, mitochondrial	1.92	0.112	1.4E-03	15	15.0
P07919	Cytochrome b-c1 complex subunit 6, mitochondrial	1.91	NA	NA	2	26.4
P02787	Serotransferrin	1.91	0.093	3.3E-05	11	18.5
P38159	RNA-binding motif protein, X chromosome	1.91	0.073	2.9E-03	4	11.0
P62847	40S ribosomal protein S24	1.90	0.107	5.5E-02	3	29.3
P09651	Heterogeneous nuclear ribonucleoprotein A1	1.90	0.069	4.6E-06	11	37.9
Q9NSD9	Phenylalanine--tRNA ligase beta subunit	1.89	0.136	2.1E-03	6	11.0
P46776	60S ribosomal protein L27a	1.89	NA	NA	2	14.2
P22314	Ubiquitin-like modifier-activating enzyme 1	1.89	0.105	2.6E-03	12	14.5
P35237	Serpin B6	1.88	NA	NA	2	6.9
Q15424	Scaffold attachment factor B1	1.87	0.114	1.9E-01	3	3.7
Q9BV36	Melanophilin	1.87	NA	NA	2	4.8
P18669	Phosphoglycerate mutase 1	1.87	0.097	8.4E-05	4	25.2
P62851	40S ribosomal protein S25	1.87	0.080	4.8E-06	4	24.0
P21796	Voltage-dependent anion-selective channel protein 1	1.87	0.070	2.2E-06	8	34.6
Q9H4A4	Aminopeptidase B	1.87	NA	NA	2	4.2
P31939	Bifunctional purine biosynthesis protein PURH	1.86	0.267	1.8E-01	6	13.5
P35659	Protein DEK	1.86	0.068	2.9E-05	4	10.4
Q9UBQ5	Eukaryotic translation initiation factor 3 subunit K	1.86	NA	NA	2	11.5
Q9BZF1	Oxysterol-binding protein-related protein 8	1.86	NA	NA	2	2.8
P31949	Protein S100-A11	1.85	0.470	4.9E-02	3	34.3
P08574	Cytochrome c1, heme protein, mitochondrial	1.85	NA	NA	2	7.1
P54652	Heat shock-related 70 kDa protein 2	1.84	0.140	1.4E-02	11	20.7
O94826	Mitochondrial import receptor subunit TOM70	1.84	0.103	4.3E-05	9	17.3
Q14157	Ubiquitin-associated protein 2-like	1.84	NA	NA	2	2.5
P24534	Elongation factor 1-beta	1.84	NA	NA	2	10.7
P09525	Annexin A4	1.83	0.736	4.6E-02	11	37.3
Q9UNF0	Protein kinase C and casein kinase substrate in neurons protein 2	1.83	0.260	7.9E-02	3	7.4
Q13428	Treacle protein	1.82	NA	NA	2	1.6
Q13435	Splicing factor 3B subunit 2	1.81	NA	NA	2	3.0
Q08380	Galectin-3-binding protein	1.81	0.216	7.5E-02	3	7.0
P18621	60S ribosomal protein L17	1.81	0.055	4.9E-03	4	27.2
P51149	Ras-related protein Rab-7a	1.81	0.138	5.4E-03	8	44.9
P46777	60S ribosomal protein L5	1.80	0.180	7.1E-03	3	11.1
O60506	Heterogeneous nuclear ribonucleoprotein Q	1.80	0.144	1.1E-02	5	9.6
Q14240	Eukaryotic initiation factor 4A-II	1.79	0.103	9.1E-03	3	8.8
P00390	Glutathione reductase, mitochondrial	1.79	0.141	2.4E-02	4	12.6

Q99572	P2X purinoceptor 7	1.78	0.166	3.2E-01	4	9.6
P49207	60S ribosomal protein L34	1.78	NA	NA	2	12.8
O14818	Proteasome subunit alpha type-7	1.78	NA	NA	2	6.9
Q13011	Delta(3,5)-Delta(2,4)-dienoyl-CoA isomerase, mitochondrial	1.77	0.090	7.9E-04	5	13.7
P35232	Prohibitin	1.77	0.057	3.2E-06	9	39.3
O15371	Eukaryotic translation initiation factor 3 subunit D	1.76	NA	NA	2	3.5
P68871	Hemoglobin subunit beta	1.76	0.065	7.2E-06	6	55.8
P61247	40S ribosomal protein S3a	1.76	0.152	9.5E-04	10	43.2
P62241	40S ribosomal protein S8	1.75	0.073	7.9E-04	3	15.4
P60866	40S ribosomal protein S20	1.75	0.111	2.3E-03	4	28.6
Q16698	2,4-dienoyl-CoA reductase, mitochondrial	1.75	0.110	2.5E-02	7	24.2
P62857	40S ribosomal protein S28	1.74	NA	NA	2	30.4
P46783	40S ribosomal protein S10	1.74	0.212	2.2E-02	3	23.6
Q12905	Interleukin enhancer-binding factor 2	1.74	0.148	2.6E-03	5	15.9
Q1KMD3	Heterogeneous nuclear ribonucleoprotein U-like protein 2	1.73	0.143	3.8E-02	4	5.8
O14980	Exportin-1	1.73	NA	NA	2	2.6
P25685	DnaJ homolog subfamily B member 1	1.73	0.188	4.7E-02	3	7.9
P54136	Arginine--tRNA ligase, cytoplasmic	1.73	0.127	8.2E-02	3	5.5
P05141	ADP/ATP translocase 2	1.73	NA	NA	2	7.7
O60343	TBC1 domain family member 4	1.73	0.085	2.3E-02	3	3.5
O15498	Synaptobrevin homolog YKT6	1.72	NA	NA	2	7.6
P22626	Heterogeneous nuclear ribonucleoproteins A2/B1	1.71	0.075	2.5E-06	13	36.3
P02545	Prelamin-A/C	1.71	0.031	0.0E+00	39	50.6
P25398	40S ribosomal protein S12	1.71	NA	NA	2	13.6
Q9Y2Q3	Glutathione S-transferase kappa 1	1.70	0.054	8.1E-05	4	22.6
P62424	60S ribosomal protein L7a	1.69	0.136	2.2E-02	4	12.8
P62263	40S ribosomal protein S14	1.69	NA	NA	2	15.2
P62249	40S ribosomal protein S16	1.69	0.063	7.7E-04	5	35.6
P14868	Aspartate--tRNA ligase, cytoplasmic	1.69	0.117	4.0E-02	5	12.0
P16152	Carbonyl reductase [NADPH] 1	1.69	0.424	3.2E-01	5	25.6
Q00839	Heterogeneous nuclear ribonucleoprotein U	1.69	0.123	5.2E-02	12	13.9
P15880	40S ribosomal protein S2	1.69	0.125	4.2E-03	4	17.4
Q14247	Src substrate cortactin	1.68	0.253	1.5E-01	4	8.2
O95202	LETM1 and EF-hand domain-containing protein 1, mitochondrial	1.68	0.154	1.7E-02	4	6.2
P39687	Acidic leucine-rich nuclear phosphoprotein 32 family member A	1.67	0.316	1.9E-01	3	14.1
P25786	Proteasome subunit alpha type-1	1.67	0.193	1.1E-01	5	20.2
P84077	ADP-ribosylation factor 1	1.67	NA	NA	2	9.9
P09874	Poly [ADP-ribose] polymerase 1	1.66	0.114	6.2E-04	10	11.5
Q08211	ATP-dependent RNA helicase A	1.66	0.210	2.3E-02	10	10.9
P26373	60S ribosomal protein L13	1.66	0.166	4.8E-02	4	19.9
Q8IZQ5	Selenoprotein H	1.66	NA	NA	2	20.5
Q9UBQ7	Glyoxylate reductase/hydroxypyruvate reductase	1.66	0.290	1.8E-01	4	21.6
Q15181	Inorganic pyrophosphatase	1.66	0.234	1.0E-01	3	10.4
P83731	60S ribosomal protein L24	1.65	0.099	2.2E-02	3	16.6
P06576	ATP synthase subunit beta, mitochondrial	1.65	0.060	5.0E-05	19	42.2
Q13510	Acid ceramidase	1.65	0.116	1.4E-03	6	17.0
Q5JTV8	Torsin-1A-interacting protein 1	1.64	0.317	2.2E-01	3	7.9
P54819	Adenylate kinase 2, mitochondrial	1.62	NA	NA	2	10.9
P62280	40S ribosomal protein S11	1.62	0.628	3.5E-01	3	17.7
P60900	Proteasome subunit alpha type-6	1.62	NA	NA	2	8.5
P11216	Glycogen phosphorylase, brain form	1.60	0.073	1.9E-05	8	11.9
Q86WA6	Valacyclovir hydrolase	1.60	NA	NA	2	7.2
P12270	Nucleoprotein TPR	1.60	0.087	1.1E-03	9	5.5
Q96AG4	Leucine-rich repeat-containing protein 59	1.59	0.124	1.1E-02	4	14.7
P08670	Vimentin	1.59	0.043	1.6E-09	31	58.2
O75083	WD repeat-containing protein 1	1.59	0.112	3.6E-03	8	15.7
Q96GK7	Fumarylacetoacetate hydrolase domain-containing protein 2A	1.59	0.353	4.0E-01	3	10.8
P69905	Hemoglobin subunit alpha	1.58	0.075	1.0E-03	8	68.3
P08865	40S ribosomal protein SA	1.58	0.134	3.5E-03	6	26.8
P46779	60S ribosomal protein L28	1.58	NA	NA	2	13.1
P12956	X-ray repair cross-complementing protein 6	1.58	0.086	1.1E-04	13	24.8
P40967	Melanocyte protein PMEL	1.58	NA	NA	2	3.3
Q9NY12	H/ACA ribonucleoprotein complex subunit 1	1.58	NA	NA	2	7.4
Q9Y376	Calcium-binding protein 39	1.58	NA	NA	2	4.7
P50502	Hsc70-interacting protein	1.58	0.145	2.4E-02	6	14.1
Q6DKI1	60S ribosomal protein L7-like 1	1.57	NA	NA	2	11.0
Q9H2U2	Inorganic pyrophosphatase 2, mitochondrial	1.57	0.159	4.8E-02	4	13.2
Q9H0U4	Ras-related protein Rab-1B	1.57	NA	NA	2	15.4
Q01130	Serine/arginine-rich splicing factor 2	1.55	0.118	9.4E-02	3	13.6
Q86UY8	5'-nucleotidase domain-containing protein 3	1.55	NA	NA	2	3.6
P67870	Casein kinase II subunit beta	1.55	NA	NA	2	10.2
Q9UJS0	Calcium-binding mitochondrial carrier protein Aralar2	1.54	0.077	7.3E-03	4	8.3
P04075	Fructose-bisphosphate aldolase A	1.54	0.089	3.9E-03	14	44.5
P49792	E3 SUMO-protein ligase RanBP2	1.54	NA	NA	2	0.2
P05388	60S acidic ribosomal protein P0	1.54	0.101	1.4E-02	6	19.6
Q9UKV3	Apoptotic chromatin condensation inducer in the nucleus	1.54	0.131	1.7E-01	3	2.9
P61313	60S ribosomal protein L15	1.53	NA	NA	2	10.3
P61978	Heterogeneous nuclear ribonucleoprotein K	1.53	0.123	5.6E-03	15	38.9
P51810	G-protein coupled receptor 143	1.53	0.461	5.0E-01	3	9.9
P14618	Pyruvate kinase PKM	1.53	0.082	4.1E-05	12	24.9
P30048	Thioredoxin-dependent peroxide reductase, mitochondrial	1.53	0.064	1.3E-04	5	36.3
P62906	60S ribosomal protein L10a	1.52	0.097	1.4E-03	7	33.6
P49755	Transmembrane emp24 domain-containing protein 10	1.52	NA	NA	2	9.1
O00560	Syntenin-1	1.52	NA	NA	2	7.0
Q09028	Histone-binding protein RBBP4	1.52	NA	NA	2	8.2
P06737	Glycogen phosphorylase, liver form	1.52	0.094	3.2E-02	5	7.9
P42765	3-ketoacyl-CoA thiolase, mitochondrial	1.52	0.139	1.8E-02	8	23.7
O96000	NADH dehydrogenase [ubiquinone] 1 beta subcomplex subunit 10	1.52	0.171	8.9E-02	3	25.0
Q99623	Prohibitin-2	1.52	0.071	3.6E-03	11	44.1
P50914	60S ribosomal protein L14	1.52	0.062	8.9E-04	4	20.9
Q9UHX1	Poly(U)-binding-splicing factor PUF60	1.52	0.080	1.2E-02	4	8.9
P48681	Nestin	1.52	0.008	6.6E-03	3	2.5
Q8N5K1	CDGSH iron-sulfur domain-containing protein 2	1.52	0.165	4.2E-02	4	34.1
P43304	Glycerol-3-phosphate dehydrogenase, mitochondrial	1.51	NA	NA	2	2.5
Q06323	Proteasome activator complex subunit 1	1.51	0.052	1.4E-03	6	22.9
Q9P2E9	Ribosome-binding protein 1	1.50	0.101	1.7E-02	12	9.7
Q8NBS9	Thioredoxin domain-containing protein 5	1.50	0.077	2.9E-02	8	16.7
P30153	Serine/threonine-protein phosphatase 2A 65 kDa regulatory subunit A alpha isoform	1.50	0.038	8.7E-02	6	11.4
P36543	V-type proton ATPase subunit E 1	1.49	0.130	1.1E-01	3	10.6
P55786	Puromycin-sensitive aminopeptidase	1.49	0.115	2.7E-02	4	4.6
P19404	NADH dehydrogenase [ubiquinone] flavoprotein 2, mitochondrial	1.49	NA	NA	2	9.2
P36578	60S ribosomal protein L4	1.48	0.142	2.1E-02	8	21.1
P49721	Proteasome subunit beta type-2	1.48	0.522	4.9E-01	3	13.9
Q15907	Ras-related protein Rab-11B	1.48	0.121	8.6E-02	3	14.7
P55265	Double-stranded RNA-specific adenosine deaminase	1.48	0.172	2.1E-01	4	3.6
O14880	Microsomal glutathione S-transferase 3	1.48	0.054	3.8E-02	3	25.7
Q8NC56	LEM domain-containing protein 2	1.48	0.139	2.5E-02	5	11.1
P26640	Valine--tRNA ligase	1.48	0.408	2.1E-01	5	5.3
Q9UL46	Proteasome activator complex subunit 2	1.47	NA	NA	2	11.3
P31153	S-adenosylmethionine synthase isoform type-2	1.47	0.226	1.2E-01	3	9.4
P04350	Tubulin beta-4A chain	1.47	0.586	2.5E-01	4	14.2
O75306	NADH dehydrogenase [ubiquinone] iron-sulfur protein 2, mitochondrial	1.46	0.084	2.2E-02	4	8.9
P51991	Heterogeneous nuclear ribonucleoprotein A3	1.46	0.066	3.4E-04	8	26.2
P46926	Glucosamine-6-phosphate isomerase 1	1.46	0.281	2.5E-01	3	8.0
Q14152	Eukaryotic translation initiation factor 3 subunit A	1.46	0.112	2.3E-02	7	6.4

P28066	Proteasome subunit alpha type-5	1.46	0.054	1.2E-02	3	20.7
P19367	Hexokinase-1	1.45	0.136	2.7E-01	13	16.5
Q02878	60S ribosomal protein L6	1.45	0.131	3.7E-02	6	21.5
Q12904	Aminoacyl tRNA synthase complex-interacting multifunctional protein 1	1.44	NA	NA	2	9.6
P17858	ATP-dependent 6-phosphofructokinase, liver type	1.44	0.102	5.8E-02	5	8.3
Q9HAV7	GrpE protein homolog 1, mitochondrial	1.44	NA	NA	2	12.0
B5ME19	Eukaryotic translation initiation factor 3 subunit C-like protein	1.44	0.105	1.3E-02	3	3.4
Q14165	Malectin	1.43	NA	NA	2	5.5
P62158	Calmodulin	1.43	0.175	3.8E-02	3	22.1
P39019	40S ribosomal protein S19	1.43	0.142	1.4E-02	5	29.7
Q9Y2B0	Protein canopy homolog 2	1.43	NA	NA	2	13.7
P78371	T-complex protein 1 subunit beta	1.42	0.683	5.3E-01	3	7.7
Q02818	Nucleobindin-1	1.42	0.171	1.3E-01	5	15.0
P45880	Voltage-dependent anion-selective channel protein 2	1.42	0.055	4.1E-05	8	27.2
P27816	Microtubule-associated protein 4	1.42	0.078	1.6E-02	4	5.1
P23396	40S ribosomal protein S3	1.42	0.112	3.9E-03	8	30.5
Q3ZCQ8	Mitochondrial import inner membrane translocase subunit TIM50	1.41	0.049	3.8E-02	3	13.0
P31040	Succinate dehydrogenase [ubiquinone] flavoprotein subunit, mitochondrial	1.41	0.433	2.6E-01	6	12.3
O75533	Splicing factor 3B subunit 1	1.40	0.219	2.7E-01	3	3.1
Q9Y277	Voltage-dependent anion-selective channel protein 3	1.40	0.180	4.2E-02	7	26.1
Q02252	Methylmalonate-semialdehyde dehydrogenase [acylating], mitochondrial	1.40	0.099	1.7E-01	3	6.4
P62753	40S ribosomal protein S6	1.40	0.117	1.1E-02	4	16.9
Q13151	Heterogeneous nuclear ribonucleoprotein A0	1.40	0.287	2.5E-01	3	7.5
Q9Y262	Eukaryotic translation initiation factor 3 subunit L	1.40	0.193	2.1E-01	3	4.4
O95292	Vesicle-associated membrane protein-associated protein B/C	1.40	NA	NA	2	13.6
O75947	ATP synthase subunit d, mitochondrial	1.39	0.122	3.8E-01	7	57.8
Q99471	Prefoldin subunit 5	1.39	NA	NA	2	9.7
P54727	UV excision repair protein RAD23 homolog B	1.38	2.868	5.6E-01	3	8.8
Q9H7C9	Mth938 domain-containing protein	1.38	NA	NA	2	20.5
P27635	60S ribosomal protein L10	1.38	0.163	6.4E-01	3	15.9
P49821	NADH dehydrogenase [ubiquinone] flavoprotein 1, mitochondrial	1.38	0.060	6.6E-02	3	6.0
Q16718	NADH dehydrogenase [ubiquinone] 1 alpha subcomplex subunit 5	1.37	NA	NA	2	22.4
O75367	Core histone macro-H2A.1	1.37	0.120	1.0E-02	10	30.1
P13010	X-ray repair cross-complementing protein 5	1.37	0.229	1.3E-01	7	14.1
O95831	Apoptosis-inducing factor 1, mitochondrial	1.37	NA	NA	2	5.2
Q9NX63	Coiled-coil-helix-coiled-coil-helix domain-containing protein 3, mitochondrial	1.37	0.121	3.4E-02	3	12.3
P25705	ATP synthase subunit alpha, mitochondrial	1.37	0.067	4.6E-04	19	42.1
P38117	Electron transfer flavoprotein subunit beta	1.37	0.139	1.4E-01	3	14.1
P11142	Heat shock cognate 71 kDa protein	1.37	0.061	1.0E-03	12	22.8
P54709	Sodium/potassium-transporting ATPase subunit beta-3	1.36	0.135	9.4E-02	10	38.7
P42167	Lamina-associated polypeptide 2, isoforms beta/gamma	1.36	0.098	2.4E-02	6	16.3
P50402	Emerin	1.36	NA	NA	2	9.8
P08237	ATP-dependent 6-phosphofructokinase, muscle type	1.35	0.817	2.1E-01	5	9.0
P62081	40S ribosomal protein S7	1.35	0.153	4.3E-01	3	14.9
Q12913	Receptor-type tyrosine-protein phosphatase eta	1.35	NA	NA	2	1.9
Q9UKM9	RNA-binding protein Raly	1.35	NA	NA	2	10.1
P36957	Dihydrolipoyllysine-residue succinyltransferase component of 2-oxoglutarate dehydrogenase complex, mitochondrial	1.35	0.112	1.3E-01	7	16.3
P30533	Alpha-2-macroglobulin receptor-associated protein	1.34	0.130	1.7E-01	3	7.8
O14561	Acyl carrier protein, mitochondrial	1.34	0.269	3.2E-01	4	19.2
P37802	Transgelin-2	1.34	0.151	7.4E-02	4	22.6
P39023	60S ribosomal protein L3	1.34	NA	NA	2	6.9
P01023	Alpha-2-macroglobulin	1.32	0.102	7.4E-03	13	11.7
P09493	Tropomyosin alpha-1 chain	1.32	1.053	4.9E-01	4	12.0
Q96A33	Coiled-coil domain-containing protein 47	1.32	NA	NA	2	4.3
Q05519	Serine/arginine-rich splicing factor 11	1.31	NA	NA	2	6.0
P60228	Eukaryotic translation initiation factor 3 subunit E	1.31	NA	NA	2	4.9
O75964	ATP synthase subunit g, mitochondrial	1.31	NA	NA	2	21.4
O75489	NADH dehydrogenase [ubiquinone] iron-sulfur protein 3, mitochondrial	1.31	0.246	2.8E-01	4	14.4
Q92945	Far upstream element-binding protein 2	1.31	0.263	1.6E-01	7	10.0
P10644	cAMP-dependent protein kinase type I-alpha regulatory subunit	1.31	NA	NA	2	7.1
Q02543	60S ribosomal protein L18a	1.31	0.195	2.0E-01	3	18.2
Q13405	39S ribosomal protein L49, mitochondrial	1.31	NA	NA	2	12.0
P20700	Lamin-B1	1.30	0.155	2.0E-01	8	14.5
O75521	Enoyl-CoA delta isomerase 2, mitochondrial	1.30	NA	NA	2	5.6
Q13228	Selenium-binding protein 1	1.30	0.216	1.3E-01	7	15.5
P21281	V-type proton ATPase subunit B, brain isoform	1.29	0.077	1.0E-01	5	14.1
Q9UHQ9	NADH-cytochrome b5 reductase 1	1.29	0.209	1.3E-01	8	32.1
P14866	Heterogeneous nuclear ribonucleoprotein L	1.29	0.183	8.9E-02	7	19.2
P56192	Methionine--tRNA ligase, cytoplasmic	1.29	NA	NA	2	3.9
Q00341	Vigilin	1.29	NA	NA	2	1.7
P40429	60S ribosomal protein L13a	1.28	0.291	4.2E-01	3	15.3
P28331	NADH-ubiquinone oxidoreductase 75 kDa subunit, mitochondrial	1.28	NA	NA	2	4.1
P61225	Ras-related protein Rap-2b	1.28	NA	NA	2	11.5
Q96AE4	Far upstream element-binding protein 1	1.27	NA	NA	2	2.6
P51608	Methyl-CpG-binding protein 2	1.27	0.771	6.4E-01	5	10.3
P53618	Coatomer subunit beta	1.27	0.008	1.5E-03	4	5.4
P02790	Hemopexin	1.27	NA	NA	2	4.3
Q9BZQ8	Protein Niban	1.27	0.203	1.4E-01	5	5.4
P23368	NAD-dependent malic enzyme, mitochondrial	1.27	NA	NA	2	4.1
P52597	Heterogeneous nuclear ribonucleoprotein F	1.27	0.092	2.5E-01	3	6.0
O75131	Copine-3	1.26	1.222	1.3E-01	6	11.9
P38606	V-type proton ATPase catalytic subunit A	1.26	NA	NA	2	3.1
P46778	60S ribosomal protein L21	1.26	NA	NA	2	14.4
P23246	Splicing factor, proline- and glutamine-rich	1.26	0.213	8.4E-02	7	11.7
O60486	Plexin-C1	1.26	0.398	1.8E-01	4	2.6
P62829	60S ribosomal protein L23	1.26	0.238	4.1E-01	3	30.7
Q9UMX5	Neudesin	1.26	NA	NA	2	16.9
O14949	Cytochrome b-c1 complex subunit 8	1.25	0.005	1.7E-02	3	37.8
Q86UP2	Kinectin	1.25	0.206	2.1E-01	10	10.5
P78527	DNA-dependent protein kinase catalytic subunit	1.25	0.118	5.0E-02	17	4.4
Q9BRX8	Redox-regulatory protein FAM213A	1.25	0.215	1.4E-01	6	29.7
P48047	ATP synthase subunit O, mitochondrial	1.25	0.434	1.0E-01	5	36.6
P51398	28S ribosomal protein S29, mitochondrial	1.25	NA	NA	2	6.5
P26368	Splicing factor U2AF 65 kDa subunit	1.24	0.205	1.7E-01	3	7.6
P34932	Heat shock 70 kDa protein 4	1.24	0.770	4.3E-01	4	6.0
P04040	Catalase	1.24	0.725	4.9E-01	4	11.0
O15144	Actin-related protein 2/3 complex subunit 2	1.24	0.289	2.8E-01	4	13.0
Q14697	Neutral alpha-glucosidase AB	1.24	0.234	6.3E-02	13	14.1
P43243	Matrin-3	1.23	0.140	2.1E-01	5	9.4
P62913	60S ribosomal protein L11	1.23	0.162	1.6E-01	3	16.9
P61353	60S ribosomal protein L27	1.23	NA	NA	2	12.5
P00505	Aspartate aminotransferase, mitochondrial	1.23	0.092	1.9E-02	6	16.7
P61019	Ras-related protein Rab-2A	1.22	NA	NA	2	12.3
P62701	40S ribosomal protein S4, X isoform	1.22	0.110	7.7E-02	8	30.0
Q8NBJ5	Procollagen galactosyltransferase 1	1.22	NA	NA	2	3.4
P51572	B-cell receptor-associated protein 31	1.21	0.193	1.9E-01	8	29.7
P18124	60S ribosomal protein L7	1.21	0.057	3.2E-03	5	15.7
P40227	T-complex protein 1 subunit zeta	1.21	0.733	6.4E-01	3	7.0
Q99714	3-hydroxyacyl-CoA dehydrogenase type-2	1.21	NA	NA	2	8.4
P46782	40S ribosomal protein S5	1.21	NA	NA	2	8.3
P11586	C-1-tetrahydrofolate synthase, cytoplasmic	1.21	0.169	4.7E-01	4	4.8
P01860	Ig gamma-3 chain C region	1.21	NA	NA	2	6.4
P51970	NADH dehydrogenase [ubiquinone] 1 alpha subcomplex subunit 8	1.21	0.300	3.2E-01	4	19.8
P78347	General transcription factor II-I	1.20	0.464	4.8E-01	3	3.4
Q9BXP5	Serrate RNA effector molecule homolog	1.19	NA	NA	2	2.1

P35998	26S protease regulatory subunit 7	1.19	0.264	2.1E-01	4	10.6
P24752	Acetyl-CoA acetyltransferase, mitochondrial	1.19	1.877	2.5E-01	7	12.4
Q16666	Gamma-interferon-inducible protein 16	1.19	0.054	1.5E-01	3	3.7
P47985	Cytochrome b-c1 complex subunit Rieske, mitochondrial	1.19	NA	NA	2	10.9
P14314	Glucosidase 2 subunit beta	1.19	0.188	3.1E-01	9	14.0
P0C0S8	Histone H2A type 1	1.19	0.167	5.3E-02	3	28.5
P49411	Elongation factor Tu, mitochondrial	1.19	0.344	4.6E-02	9	25.9
P46781	40S ribosomal protein S9	1.19	0.098	2.0E-01	6	23.7
O60313	Dynamin-like 120 kDa protein, mitochondrial	1.18	0.257	1.8E-01	7	9.4
Q5SSJ5	Heterochromatin protein 1-binding protein 3	1.18	NA	NA	2	5.4
Q5JWF2	Guanine nucleotide-binding protein G(s) subunit alpha isoforms XLas	1.18	0.143	3.3E-01	8	8.5
Q9BZZ5	Apoptosis inhibitor 5	1.18	NA	NA	2	4.8
P61160	Actin-related protein 2	1.18	0.825	3.3E-01	4	12.9
O00299	Chloride intracellular channel protein 1	1.18	NA	NA	2	10.0
P32969	60S ribosomal protein L9	1.18	0.209	2.4E-01	3	18.2
P05198	Eukaryotic translation initiation factor 2 subunit 1	1.17	0.753	6.7E-01	4	14.9
P09669	Cytochrome c oxidase subunit 6C	1.17	0.519	5.3E-01	3	38.7
Q9BQI0	Allograft inflammatory factor 1-like	1.16	0.198	4.3E-01	3	16.0
Q9P0J0	NADH dehydrogenase [ubiquinone] 1 alpha subcomplex subunit 13	1.16	NA	NA	2	6.9
Q7KZF4	Staphylococcal nuclease domain-containing protein 1	1.16	0.198	5.5E-01	4	6.4
P14854	Cytochrome c oxidase subunit 6B1	1.16	1.526	8.7E-01	4	37.2
Q00325	Phosphate carrier protein, mitochondrial	1.15	0.067	4.9E-02	8	21.3
P36542	ATP synthase subunit gamma, mitochondrial	1.14	0.556	4.9E-01	3	11.1
Q8TAQ2	SWI/SNF complex subunit SMARCC2	1.14	0.093	3.0E-01	3	2.6
P53621	Coatomer subunit alpha	1.14	0.951	5.5E-01	6	6.5
P62333	26S protease regulatory subunit 10B	1.14	NA	NA	2	6.7
Q13162	Peroxiredoxin-4	1.14	NA	NA	2	8.9
P49588	Alanine--tRNA ligase, cytoplasmic	1.13	NA	NA	2	2.4
Q13825	Methylglutaconyl-CoA hydratase, mitochondrial	1.13	NA	NA	2	8.3
O94832	Unconventional myosin-Id	1.13	0.168	2.2E-01	10	11.3
Q9BUJ2	Heterogeneous nuclear ribonucleoprotein U-like protein 1	1.12	NA	NA	2	3.9
P26038	Moesin	1.12	0.294	2.5E-01	10	16.6
P62888	60S ribosomal protein L30	1.12	NA	NA	2	20.9
Q13263	Transcription intermediary factor 1-beta	1.12	0.368	4.4E-01	6	9.5
P35637	RNA-binding protein FUS	1.12	41.860	4.9E-01	4	6.1
Q06210	Glutamine--fructose-6-phosphate aminotransferase [isomerizing] 1	1.11	NA	NA	2	3.7
P21912	Succinate dehydrogenase [ubiquinone] iron-sulfur subunit, mitochondrial	1.11	34.278	6.6E-01	3	13.2
P11310	Medium-chain specific acyl-CoA dehydrogenase, mitochondrial	1.11	1.046	5.4E-01	3	7.4
Q8N163	Cell cycle and apoptosis regulator protein 2	1.11	NA	NA	2	3.7
Q9UQE7	Structural maintenance of chromosomes protein 3	1.10	2.636	8.3E-01	3	3.9
P27708	CAD protein	1.10	1.805	8.5E-01	3	1.7
P43686	26S protease regulatory subunit 6B	1.10	NA	NA	2	6.0
O15173	Membrane-associated progesterone receptor component 2	1.10	0.778	7.2E-01	3	18.4
Q02790	Peptidyl-prolyl cis-trans isomerase FKBP4	1.10	0.381	7.8E-01	3	6.1
Q9P0K7	Ankycorbin	1.10	NA	NA	2	2.6
O43852	Calumenin	1.09	0.032	1.8E-01	4	13.7
Q9P2R7	Succinyl-CoA ligase [ADP-forming] subunit beta, mitochondrial	1.09	0.301	5.6E-01	3	7.6
P10606	Cytochrome c oxidase subunit 5B, mitochondrial	1.09	0.708	6.4E-01	5	30.2
Q15029	116 kDa U5 small nuclear ribonucleoprotein component	1.08	NA	NA	2	2.6
O95716	Ras-related protein Rab-3D	1.08	NA	NA	2	10.5
O60493	Sorting nexin-3	1.08	NA	NA	2	11.7
Q15942	Zyxin	1.08	1.643	8.3E-01	3	8.7
Q27J81	Inverted formin-2	1.07	NA	NA	2	1.8
Q16795	NADH dehydrogenase [ubiquinone] 1 alpha subcomplex subunit 9, mitochondrial	1.07	0.929	7.9E-01	4	12.7
P52272	Heterogeneous nuclear ribonucleoprotein M	1.07	0.645	5.6E-01	7	11.6
Q01844	RNA-binding protein EWS	1.07	NA	NA	2	2.3
Q9Y3U8	60S ribosomal protein L36	1.07	0.488	7.3E-01	5	36.2
P55084	Trifunctional enzyme subunit beta, mitochondrial	1.07	0.342	5.1E-01	11	26.8
Q9Y394	Dehydrogenase/reductase SDR family member 7	1.07	0.192	5.8E-01	3	11.5
Q13200	26S proteasome non-ATPase regulatory subunit 2	1.07	0.663	6.4E-01	4	5.2
P62269	40S ribosomal protein S18	1.06	0.251	7.3E-01	7	39.5
Q96QK1	Vacuolar protein sorting-associated protein 35	1.06	NA	NA	2	3.0
Q14980	Nuclear mitotic apparatus protein 1	1.06	0.741	4.4E-01	9	6.1
P15311	Ezrin	1.06	0.100	5.7E-01	4	7.2
P55884	Eukaryotic translation initiation factor 3 subunit B	1.05	0.193	6.3E-01	6	9.8
P26599	Polypyrimidine tract-binding protein 1	1.05	0.232	8.1E-01	4	8.1
P17844	Probable ATP-dependent RNA helicase DDX5	1.05	NA	NA	2	3.3
P61026	Ras-related protein Rab-10	1.05	NA	NA	2	9.5
P62277	40S ribosomal protein S13	1.04	0.613	7.8E-01	5	29.1
Q9Y265	RuvB-like 1	1.04	Inf	7.0E-01	3	9.4
Q15233	Non-POU domain-containing octamer-binding protein	1.04	0.081	4.5E-01	3	8.1
Q9NTK5	Obg-like ATPase 1	1.04	NA	NA	2	8.3
O43390	Heterogeneous nuclear ribonucleoprotein R	1.04	0.155	7.0E-01	9	15.3
Q86V81	THO complex subunit 4	1.04	NA	NA	2	8.2
Q9Y230	RuvB-like 2	1.04	NA	NA	2	6.3
Q9P0L0	Vesicle-associated membrane protein-associated protein A	1.04	0.448	8.4E-01	3	15.7
P61457	Pterin-4-alpha-carbinolamine dehydratase	1.04	9.656	9.2E-01	3	29.8
Q13561	Dynactin subunit 2	1.03	0.175	7.9E-01	4	11.5
P49327	Fatty acid synthase	1.03	0.507	9.1E-01	6	3.2
P31930	Cytochrome b-c1 complex subunit 1, mitochondrial	1.02	17.063	8.3E-01	6	17.9
Q14344	Guanine nucleotide-binding protein subunit alpha-13	1.02	2.807	9.7E-01	3	8.8
Q92841	Probable ATP-dependent RNA helicase DDX17	1.02	0.654	9.6E-01	4	7.4
O14745	Na(+)/H(+) exchange regulatory cofactor NHE-RF1	1.02	NA	NA	2	8.7
P29966	Myristoylated alanine-rich C-kinase substrate	1.01	0.764	9.7E-01	5	26.5
Q07666	KH domain-containing, RNA-binding, signal transduction-associated protein 1	1.01	0.346	9.0E-01	4	7.9
P27824	Calnexin	1.01	2.235	9.5E-01	7	16.0
P20645	Cation-dependent mannose-6-phosphate receptor	1.01	0.357	9.9E-01	3	15.5
Q9BS26	Endoplasmic reticulum resident protein 44	1.01	2.149	9.6E-01	5	10.6
Q99805	Transmembrane 9 superfamily member 2	1.00	NA	NA	2	3.5
Q9UHD8	Septin-9	1.00	0.433	9.8E-01	4	8.9
P62318	Small nuclear ribonucleoprotein Sm D3	1.00	3.560	9.9E-01	3	31.7
P22695	Cytochrome b-c1 complex subunit 2, mitochondrial	1.00	0.278	9.9E-01	4	11.5
P27144	Adenylate kinase 4, mitochondrial	1.00	NA	NA	2	9.9
P46937	Yorkie homolog	1.00	NA	NA	2	5.8
Q96JB5	CDK5 regulatory subunit-associated protein 3	1.00	NA	NA	2	3.0
Q7Z7H5	Transmembrane emp24 domain-containing protein 4	0.99	NA	NA	2	10.1
P18859	ATP synthase-coupling factor 6, mitochondrial	0.99	1.066	9.8E-01	3	32.4
O60664	Perilipin-3	0.99	NA	NA	2	7.4
P50213	Isocitrate dehydrogenase [NAD] subunit alpha, mitochondrial	0.99	NA	NA	2	6.8
Q9UNH7	Sorting nexin-6	0.99	NA	NA	2	3.2
P55209	Nucleosome assembly protein 1-like 1	0.99	0.377	9.4E-01	3	12.0
Q99829	Copine-1	0.99	NA	NA	2	4.7
Q06830	Peroxiredoxin-1	0.99	Inf	8.5E-01	7	43.2
Q99584	Protein S100-A13	0.98	0.846	9.1E-01	4	41.8
Q6P587	Acylpyruvase FAHD1, mitochondrial	0.98	NA	NA	2	9.8
P11940	Polyadenylate-binding protein 1	0.98	0.256	8.6E-01	8	13.8
P59998	Actin-related protein 2/3 complex subunit 4	0.98	0.299	8.8E-01	3	16.1
P09496	Clathrin light chain A	0.98	NA	NA	2	5.6
P49257	Protein ERGIC-53	0.97	2.201	7.7E-01	6	16.3
Q15008	26S proteasome non-ATPase regulatory subunit 6	0.96	NA	NA	2	5.9
P30050	60S ribosomal protein L12	0.95	3.224	8.2E-01	6	54.5
P55072	Transitional endoplasmic reticulum ATPase	0.95	0.259	5.5E-01	15	20.5
P14927	Cytochrome b-c1 complex subunit 7	0.95	0.217	6.1E-01	3	25.2
Q7L5N1	COP9 signalosome complex subunit 6	0.94	NA	NA	2	8.0
P40939	Trifunctional enzyme subunit alpha, mitochondrial	0.94	0.651	5.8E-01	15	25.6

P62979	Ubiquitin-40S ribosomal protein S27a	0.94	0.365	5.6E-01	9	50.0
P05023	Sodium/potassium-transporting ATPase subunit alpha-1	0.94	0.149	4.2E-01	14	16.4
P37837	Transaldolase	0.93	0.356	8.0E-01	3	7.4
O75643	U5 small nuclear ribonucleoprotein 200 kDa helicase	0.93	0.523	8.0E-01	5	2.3
Q15365	Poly(rC)-binding protein 1	0.93	0.329	6.2E-01	5	19.4
Q15631	Translin	0.92	NA	NA	2	7.5
P45954	Short/branched chain specific acyl-CoA dehydrogenase, mitochondrial	0.92	1.673	8.6E-01	3	7.6
P11021	78 kDa glucose-regulated protein	0.92	0.135	2.8E-01	23	35.2
P61158	Actin-related protein 3	0.92	0.262	6.2E-01	3	11.7
O60841	Eukaryotic translation initiation factor 5B	0.92	NA	NA	2	1.6
Q9UGP8	Translocation protein SEC63 homolog	0.91	1.470	7.4E-01	3	5.4
P04843	Dolichyl-diphosphooligosaccharide--protein glycosyltransferase subunit 1	0.91	0.119	2.6E-01	13	26.2
Q9UIJ7	GTP:AMP phosphotransferase AK3, mitochondrial	0.91	NA	NA	2	11.0
P50991	T-complex protein 1 subunit delta	0.90	0.288	5.6E-01	4	9.3
P00918	Carbonic anhydrase 2	0.90	NA	NA	2	10.8
P26196	Probable ATP-dependent RNA helicase DDX6	0.90	NA	NA	2	4.1
Q99832	T-complex protein 1 subunit eta	0.90	0.965	6.7E-01	4	9.2
P01857	Ig gamma-1 chain C region	0.90	0.195	1.3E-01	6	31.5
Q92542	Nicastrin	0.90	NA	NA	2	2.7
O60645	Exocyst complex component 3	0.90	NA	NA	2	3.6
P48643	T-complex protein 1 subunit epsilon	0.90	0.122	7.0E-02	6	9.6
P62316	Small nuclear ribonucleoprotein Sm D2	0.90	NA	NA	2	23.7
Q16563	Synaptophysin-like protein 1	0.89	NA	NA	2	10.0
P53396	ATP-citrate synthase	0.89	1.608	5.6E-01	3	2.9
P0CG05	Ig lambda-2 chain C regions	0.89	0.123	2.5E-01	4	55.7
Q8N1G4	Leucine-rich repeat-containing protein 47	0.88	NA	NA	2	5.5
P50990	T-complex protein 1 subunit theta	0.88	0.094	1.9E-01	9	16.8
Q9H4M9	EH domain-containing protein 1	0.88	0.165	3.0E-01	3	7.5
P39656	Dolichyl-diphosphooligosaccharide--protein glycosyltransferase 48 kDa subunit	0.88	0.069	1.1E-01	4	8.8
Q01518	Adenylyl cyclase-associated protein 1	0.87	NA	NA	2	5.1
Q9UBI6	Guanine nucleotide-binding protein G(I)/G(S)/G(O) subunit gamma-12	0.87	0.831	5.3E-01	4	45.8
O00303	Eukaryotic translation initiation factor 3 subunit F	0.87	NA	NA	2	8.1
O00231	26S proteasome non-ATPase regulatory subunit 11	0.87	0.341	6.1E-01	4	10.7
P46940	Ras GTPase-activating-like protein IQGAP1	0.87	0.307	2.6E-01	7	5.0
P49368	T-complex protein 1 subunit gamma	0.87	0.120	1.3E-01	9	18.7
P24539	ATP synthase F(0) complex subunit B1, mitochondrial	0.86	NA	NA	2	9.0
O60716	Catenin delta-1	0.86	0.423	6.6E-01	3	3.3
P07237	Protein disulfide-isomerase	0.85	0.885	2.8E-01	13	24.6
Q8NBQ5	Estradiol 17-beta-dehydrogenase 11	0.85	NA	NA	2	8.7
P46459	Vesicle-fusing ATPase	0.85	NA	NA	2	3.1
P63000	Ras-related C3 botulinum toxin substrate 1	0.85	0.314	3.8E-01	4	24.5
P17655	Calpain-2 catalytic subunit	0.85	NA	NA	2	4.3
P23634	Plasma membrane calcium-transporting ATPase 4	0.84	0.311	1.9E-01	3	4.2
P30519	Heme oxygenase 2	0.84	NA	NA	2	9.8
P20674	Cytochrome c oxidase subunit 5A, mitochondrial	0.84	0.107	3.2E-01	4	24.7
P17568	NADH dehydrogenase [ubiquinone] 1 beta subcomplex subunit 7	0.84	NA	NA	2	18.2
O75955	Flotillin-1	0.84	NA	NA	2	5.9
P84103	Serine/arginine-rich splicing factor 3	0.83	NA	NA	2	15.9
Q14203	Dynactin subunit 1	0.83	0.115	2.5E-01	3	4.2
O43707	Alpha-actinin-4	0.82	0.079	1.7E-03	16	21.5
P04179	Superoxide dismutase [Mn], mitochondrial	0.82	0.295	2.7E-01	9	41.9
P57053	Histone H2B type F-S	0.81	NA	NA	2	7.9
Q13283	Ras GTPase-activating protein-binding protein 1	0.81	NA	NA	2	7.3
Q00577	Transcriptional activator protein Pur-alpha	0.81	1.217	5.5E-01	3	13.0
P27797	Calreticulin	0.81	0.326	2.8E-01	9	26.6
P01859	Ig gamma-2 chain C region	0.80	0.103	3.0E-01	3	12.0
P49748	Very long-chain specific acyl-CoA dehydrogenase, mitochondrial	0.80	0.206	1.2E-01	6	8.5
P61764	Syntaxin-binding protein 1	0.80	0.068	8.1E-02	3	6.4
P00367	Glutamate dehydrogenase 1, mitochondrial	0.80	0.408	2.3E-01	7	15.2
Q6UVK1	Chondroitin sulfate proteoglycan 4	0.79	NA	NA	2	1.5
P49591	Serine--tRNA ligase, cytoplasmic	0.79	1.683	6.2E-01	3	6.8
P07602	Prosaposin	0.79	52.784	5.6E-01	5	7.1
P62805	Histone H4	0.79	0.184	4.6E-03	8	52.4
Q15084	Protein disulfide-isomerase A6	0.78	0.208	1.2E-01	7	19.8
Q14258	E3 ubiquitin/ISG15 ligase TRIM25	0.78	NA	NA	2	3.8
Q02218	2-oxoglutarate dehydrogenase, mitochondrial	0.78	0.168	1.0E-01	8	10.1
Q15717	ELAV-like protein 1	0.78	NA	NA	2	8.3
P01876	Ig alpha-1 chain C region	0.78	0.234	2.2E-01	3	10.5
P01834	Ig kappa chain C region	0.78	0.119	7.5E-02	4	65.1
P09012	U1 small nuclear ribonucleoprotein A	0.77	NA	NA	2	9.2
Q00765	Receptor expression-enhancing protein 5	0.77	0.249	3.9E-01	3	14.3
Q14204	Cytoplasmic dynein 1 heavy chain 1	0.77	0.056	4.6E-06	39	9.7
Q14254	Flotillin-2	0.77	0.131	8.8E-02	5	12.9
Q71DI3	Histone H3.2	0.77	0.121	5.2E-02	5	25.7
Q9Y6N5	Sulfide:quinone oxidoreductase, mitochondrial	0.77	NA	NA	2	7.6
P51116	Fragile X mental retardation syndrome-related protein 2	0.75	NA	NA	2	4.0
Q05682	Caldesmon	0.75	0.394	2.9E-01	7	13.2
Q14108	Lysosome membrane protein 2	0.75	0.084	5.6E-02	3	8.4
P62244	40S ribosomal protein S15a	0.75	2.696	4.7E-01	4	30.8
Q8IX12	Cell division cycle and apoptosis regulator protein 1	0.75	NA	NA	2	2.3
P29692	Elongation factor 1-delta	0.75	0.812	3.7E-01	3	13.5
P31942	Heterogeneous nuclear ribonucleoprotein H3	0.74	NA	NA	2	11.0
P46063	ATP-dependent DNA helicase Q1	0.74	NA	NA	2	3.9
P13987	CD59 glycoprotein	0.74	0.116	1.8E-01	3	23.4
Q12797	Aspartyl/asparaginyl beta-hydroxylase	0.74	0.119	5.2E-02	4	6.2
P43307	Translocon-associated protein subunit alpha	0.74	0.136	3.0E-01	3	11.9
P35241	Radixin	0.74	NA	NA	2	3.9
P43490	Nicotinamide phosphoribosyltransferase	0.74	7.356	7.8E-01	3	7.9
P04632	Calpain small subunit 1	0.74	0.182	1.5E-01	5	16.0
P08134	Rho-related GTP-binding protein RhoC	0.74	0.091	7.0E-02	4	21.8
O75746	Calcium-binding mitochondrial carrier protein Aralar1	0.73	0.152	1.4E-01	4	8.1
P50995	Annexin A11	0.73	0.288	2.4E-01	4	9.3
P49458	Signal recognition particle 9 kDa protein	0.73	NA	NA	2	25.6
Q53GQ0	Estradiol 17-beta-dehydrogenase 12	0.73	0.287	1.6E-01	5	20.8
P60953	Cell division control protein 42 homolog	0.73	0.110	1.1E-01	4	22.0
P0C0S5	Histone H2A.Z	0.73	NA	NA	2	18.8
O75915	PRA1 family protein 3	0.72	0.301	5.9E-01	3	19.7
Q16891	Mitochondrial inner membrane protein	0.72	0.355	1.8E-01	8	13.6
Q12965	Unconventional myosin-Ie	0.71	0.174	2.3E-01	3	2.5
P13667	Protein disulfide-isomerase A4	0.71	0.111	3.7E-02	5	9.3
P30101	Protein disulfide-isomerase A3	0.71	0.131	1.1E-03	16	31.3
P14625	Endoplasmin	0.70	0.057	1.9E-06	14	21.2
Q07065	Cytoskeleton-associated protein 4	0.70	0.115	2.3E-02	6	13.5
P01024	Complement C3	0.70	0.197	1.6E-01	15	11.6
P50454	Serpin H1	0.70	1.634	5.3E-01	5	15.8
P13473	Lysosome-associated membrane glycoprotein 2	0.69	NA	NA	2	4.9
P01009	Alpha-1-antitrypsin	0.69	0.121	8.7E-03	10	26.3
P17987	T-complex protein 1 subunit alpha	0.69	0.128	6.5E-02	5	9.7
P04844	Dolichyl-diphosphooligosaccharide--protein glycosyltransferase subunit 2	0.69	0.120	1.2E-01	3	5.4
O43681	ATPase ASNA1	0.68	NA	NA	2	8.0
P35222	Catenin beta-1	0.68	NA	NA	2	4.0
P47756	F-actin-capping protein subunit beta	0.68	NA	NA	2	8.7
P61163	Alpha-centractin	0.67	NA	NA	2	9.8
Q15019	Septin-2	0.67	0.097	3.6E-02	7	29.9
P02774	Vitamin D-binding protein	0.67	0.032	1.9E-03	3	6.5

Q03252	Lamin-B2	0.67	0.058	6.4E-06	15	26.0
P12235	ADP/ATP translocase 1	0.67	0.085	1.9E-02	4	14.1
Q6NUK1	Calcium-binding mitochondrial carrier protein SCaMC-1	0.66	NA	NA	2	3.4
P07305	Histone H1.0	0.66	0.262	3.0E-01	3	16.0
Q08722	Leukocyte surface antigen CD47	0.66	NA	NA	2	5.9
P13073	Cytochrome c oxidase subunit 4 isoform 1, mitochondrial	0.66	NA	NA	2	12.4
Q14683	Structural maintenance of chromosomes protein 1A	0.66	1.794	5.4E-01	3	2.8
P55060	Exportin-2	0.66	NA	NA	2	2.3
Q71U36	Tubulin alpha-1A chain	0.65	0.066	2.1E-04	5	16.0
O00264	Membrane-associated progesterone receptor component 1	0.65	0.226	7.5E-02	3	15.9
P16435	NADPH--cytochrome P450 reductase	0.64	NA	NA	2	2.5
Q92499	ATP-dependent RNA helicase DDX1	0.64	0.144	8.2E-02	3	4.3
P02647	Apolipoprotein A-I	0.63	0.085	1.4E-05	11	44.6
O14773	Tripeptidyl-peptidase 1	0.63	0.070	2.8E-02	4	8.7
P42025	Beta-centractin	0.61	NA	NA	2	10.1
Q7L576	Cytoplasmic FMR1-interacting protein 1	0.61	NA	NA	2	2.0
P07437	Tubulin beta chain	0.61	0.097	9.8E-03	3	9.9
Q15149	Plectin	0.60	0.053	6.6E-07	43	11.0
P51812	Ribosomal protein S6 kinase alpha-3	0.59	0.130	1.2E-01	3	4.3
O75369	Filamin-B	0.59	0.135	1.7E-02	4	2.9
P07384	Calpain-1 catalytic subunit	0.59	0.157	7.4E-02	4	7.0
Q04637	Eukaryotic translation initiation factor 4 gamma 1	0.58	NA	NA	2	1.0
P23284	Peptidyl-prolyl cis-trans isomerase B	0.58	0.092	1.3E-02	9	34.3
O00159	Unconventional myosin-Ic	0.58	0.096	1.9E-02	16	17.9
Q9UHG3	Prenylcysteine oxidase 1	0.58	0.180	1.2E-01	5	10.5
P21964	Catechol O-methyltransferase	0.58	0.088	2.3E-01	3	14.8
P02652	Apolipoprotein A-II	0.58	NA	NA	2	17.0
P29590	Protein PML	0.57	NA	NA	2	2.7
Q9BSJ8	Extended synaptotagmin-1	0.57	0.289	1.4E-01	4	4.4
P13861	cAMP-dependent protein kinase type II-alpha regulatory subunit	0.57	0.020	1.8E-02	3	10.9
P09543	2',3'-cyclic-nucleotide 3'-phosphodiesterase	0.57	0.500	2.7E-01	5	11.4
P60709	Actin, cytoplasmic 1	0.56	0.043	3.4E-08	7	30.9
Q92522	Histone H1x	0.56	0.245	6.4E-02	3	16.9
P07339	Cathepsin D	0.56	0.053	1.4E-02	4	11.4
Q9HDC9	Adipocyte plasma membrane-associated protein	0.56	NA	NA	2	5.0
P35580	Myosin-10	0.56	0.111	1.6E-04	13	9.9
Q16181	Septin-7	0.54	0.092	5.0E-03	3	8.7
Q00610	Clathrin heavy chain 1	0.54	0.058	1.8E-08	24	18.6
Q9NQC3	Reticulon-4	0.53	0.091	9.5E-04	3	5.1
P47755	F-actin-capping protein subunit alpha-2	0.52	NA	NA	2	13.3
P67936	Tropomyosin alpha-4 chain	0.51	0.161	5.1E-03	7	22.2
P00738	Haptoglobin	0.51	0.094	3.2E-03	7	19.0
P06727	Apolipoprotein A-IV	0.51	0.142	1.2E-02	11	29.0
P12814	Alpha-actinin-1	0.50	0.052	4.8E-07	14	21.3
Q9Y490	Talin-1	0.50	0.056	6.3E-09	23	12.0
O94905	Erlin-2	0.50	0.167	5.4E-02	4	10.0
P06899	Histone H2B type 1-J	0.48	NA	NA	2	7.9
Q10567	AP-1 complex subunit beta-1	0.48	0.248	9.4E-02	3	3.8
P41219	Peripherin	0.47	0.341	6.9E-02	7	16.2
O43865	Putative adenosylhomocysteinase 2	0.47	NA	NA	2	3.6
P16615	Sarcoplasmic/endoplasmic reticulum calcium ATPase 2	0.46	0.056	2.6E-04	5	5.9
P46821	Microtubule-associated protein 1B	0.46	NA	NA	2	1.0
P06396	Gelsolin	0.46	0.067	6.1E-07	12	19.7
O14950	Myosin regulatory light chain 12B	0.45	NA	NA	2	12.2
O43301	Heat shock 70 kDa protein 12A	0.44	0.037	4.9E-05	4	6.7
Q92973	Transportin-1	0.44	0.516	1.9E-01	4	5.8
Q93050	V-type proton ATPase 116 kDa subunit a isoform 1	0.44	NA	NA	2	2.7
Q09666	Neuroblast differentiation-associated protein AHNAK	0.44	0.035	1.5E-12	67	9.6
P04217	Alpha-1B-glycoprotein	0.43	0.173	1.8E-01	3	9.3
P01042	Kininogen-1	0.43	NA	NA	2	4.2
P18206	Vinculin	0.43	0.077	1.2E-04	11	11.4
P32119	Peroxiredoxin-2	0.43	0.110	4.0E-04	4	18.2
P00387	NADH-cytochrome b5 reductase 3	0.42	0.106	1.3E-02	5	23.3
Q6DD88	Atlastin-3	0.41	NA	NA	2	3.5
P08294	Extracellular superoxide dismutase [Cu-Zn]	0.40	NA	NA	2	12.1
Q6NZI2	Polymerase I and transcript release factor	0.40	0.107	1.5E-03	6	22.6
Q7Z406	Myosin-14	0.39	0.095	1.5E-05	4	2.4
Q9NZN4	EH domain-containing protein 2	0.38	NA	NA	2	5.2
P05556	Integrin beta-1	0.38	0.085	1.1E-05	6	8.9
Q14764	Major vault protein	0.38	NA	NA	2	2.8
Q03135	Caveolin-1	0.38	NA	NA	2	13.5
P17612	cAMP-dependent protein kinase catalytic subunit alpha	0.38	NA	NA	2	4.3
P43121	Cell surface glycoprotein MUC18	0.35	NA	NA	2	2.6
P11413	Glucose-6-phosphate 1-dehydrogenase	0.33	NA	NA	2	4.9
P05362	Intercellular adhesion molecule 1	0.32	NA	NA	2	4.5
O43175	D-3-phosphoglycerate dehydrogenase	0.31	NA	NA	2	5.1
P17661	Desmin	0.31	NA	NA	2	4.3
P14543	Nidogen-1	0.31	0.356	1.5E-01	6	5.8
P68366	Tubulin alpha-4A chain	0.30	0.498	1.4E-01	3	8.0
Q96HC4	PDZ and LIM domain protein 5	0.28	NA	NA	2	3.5
Q9HBL0	Tensin-1	0.28	0.416	8.2E-02	4	3.4
Q63ZY3	KN motif and ankyrin repeat domain-containing protein 2	0.28	NA	NA	2	2.8
Q08431	Lactadherin	0.27	NA	NA	2	7.2
Q14141	Septin-6	0.27	NA	NA	2	6.9
Q9UKS6	Protein kinase C and casein kinase substrate in neurons protein 3	0.26	NA	NA	2	5.0
Q9Y6C2	EMILIN-1	0.25	NA	NA	2	2.9
Q16270	Insulin-like growth factor-binding protein 7	0.24	NA	NA	2	10.3
P24844	Myosin regulatory light polypeptide 9	0.24	NA	NA	2	12.2
P61769	Beta-2-microglobulin	0.23	NA	NA	2	16.8
Q13885	Tubulin beta-2A chain	0.22	NA	NA	2	5.4
P05026	Sodium/potassium-transporting ATPase subunit beta-1	0.21	NA	NA	2	6.9
P06756	Integrin alpha-V	0.21	0.346	1.3E-01	3	3.8
Q14699	Raftlin	0.20	NA	NA	2	4.2
Q15041	ADP-ribosylation factor-like protein 6-interacting protein 1	0.19	NA	NA	2	7.9
P05186	Alkaline phosphatase, tissue-nonspecific isozyme	0.19	NA	NA	2	4.8
P07360	Complement component C8 gamma chain	0.19	NA	NA	2	18.3
P07196	Neurofilament light polypeptide	0.19	NA	NA	2	4.2
P59768	Guanine nucleotide-binding protein G(I)/G(S)/G(O) subunit gamma-2	0.18	NA	NA	2	32.4
Q13509	Tubulin beta-3 chain	0.18	NA	NA	2	5.8
Q05707	Collagen alpha-1(XIV) chain	0.18	0.833	2.0E-01	3	2.3
Q01995	Transgelin	0.17	0.280	1.2E-01	3	16.9
P11277	Spectrin beta chain, erythrocytic	0.16	0.239	2.9E-01	3	2.0
P08571	Monocyte differentiation antigen CD14	0.16	NA	NA	2	7.5
P36269	Gamma-glutamyltransferase 5	0.15	NA	NA	2	3.9
P02462	Collagen alpha-1(IV) chain	0.13	0.560	7.7E-02	3	2.3
Q14767	Latent-transforming growth factor beta-binding protein 2	0.13	NA	NA	2	1.6
Q63HR2	Tensin-like C1 domain-containing phosphatase	0.12	NA	NA	2	1.4
Q96CX2	BTB/POZ domain-containing protein KCTD12	0.12	NA	NA	2	5.5
O43491	Band 4.1-like protein 2	0.10	NA	NA	2	2.7
P17643	5,6-dihydroxyindole-2-carboxylic acid oxidase	0.10	NA	NA	2	5.6
P21926	CD9 antigen	0.10	NA	NA	2	7.5
Q9BXN1	Asporin	0.09	NA	NA	2	7.4
O94875	Sorbin and SH3 domain-containing protein 2	0.08	NA	NA	2	3.7
P80723	Brain acid soluble protein 1	0.08	0.099	8.8E-02	5	33.5
P43320	Beta-crystallin B2	0.08	NA	NA	2	11.7

Q9BXM0	Periaxin	0.08	NA	NA	2	1.9
P26447	Protein S100-A4	0.07	NA	NA	2	18.8
P07357	Complement component C8 alpha chain	0.03	NA	NA	2	7.0
P22352	Glutathione peroxidase 3	0.03	NA	NA	2	8.0
P08123	Collagen alpha-2(I) chain	0.02	NA	NA	2	2.4
P09936	Ubiquitin carboxyl-terminal hydrolase isozyme L1	0.01	NA	NA	2	10.3
P08133	Annexin A6	0.37	0.042	6.7E-16	24	42.9
P35579	Myosin-9	0.35	0.036	0.0E+00	56	32.1
Q16363	Laminin subunit alpha-4	0.35	0.172	5.0E-02	3	2.4
P17931	Galectin-3	0.35	0.091	5.0E-04	6	28.4
P00167	Cytochrome b5	0.34	0.172	2.9E-02	3	35.8
P48735	Isocitrate dehydrogenase [NADP], mitochondrial	0.34	0.206	1.4E-02	4	9.7
Q9BTV4	Transmembrane protein 43	0.34	0.217	6.6E-03	4	14.0
Q01082	Spectrin beta chain, non-erythrocytic 1	0.34	0.044	0.0E+00	51	26.4
Q9NZM1	Myoferlin	0.33	0.073	9.6E-05	4	2.4
P60660	Myosin light polypeptide 6	0.32	0.062	5.8E-11	7	58.3
P50895	Basal cell adhesion molecule	0.31	0.377	2.9E-02	4	9.4
Q13813	Spectrin alpha chain, non-erythrocytic 1	0.31	0.033	0.0E+00	74	32.5
P27105	Erythrocyte band 7 integral membrane protein	0.31	0.072	1.3E-03	5	18.4
Q16555	Dihydropyrimidinase-related protein 2	0.30	0.067	2.4E-07	11	27.3
P09382	Galectin-1	0.30	0.078	2.5E-07	5	43.7
Q02952	A-kinase anchor protein 12	0.30	0.106	4.3E-02	10	9.5
P55268	Laminin subunit beta-2	0.27	0.173	3.7E-03	11	8.0
P02679	Fibrinogen gamma chain	0.27	0.354	1.8E-02	8	23.6
P02511	Alpha-crystallin B chain	0.27	0.089	3.7E-03	5	29.1
O00468	Agrin	0.25	0.187	3.0E-02	4	2.8
P02675	Fibrinogen beta chain	0.24	0.084	1.4E-06	8	22.2
P07099	Epoxide hydrolase 1	0.22	0.112	1.3E-05	5	13.0
Q14956	Transmembrane glycoprotein NMB	0.22	0.320	2.7E-02	4	7.5
P68032	Actin, alpha cardiac muscle 1	0.22	0.324	5.5E-04	6	28.6
P04196	Histidine-rich glycoprotein	0.21	0.183	3.9E-02	3	8.0
Q14112	Nidogen-2	0.20	0.194	2.4E-03	4	3.4
P02749	Beta-2-glycoprotein 1	0.20	0.079	2.5E-06	7	31.0
P00450	Ceruloplasmin	0.20	0.116	1.1E-03	5	7.2
P12111	Collagen alpha-3(VI) chain	0.19	0.092	3.6E-08	23	8.4
P02549	Spectrin alpha chain, erythrocytic 1	0.19	0.074	4.6E-04	5	2.8
P01903	HLA class II histocompatibility antigen, DR alpha chain	0.19	0.192	2.1E-03	3	15.7
P00747	Plasminogen	0.19	0.313	1.9E-02	3	3.6
P02649	Apolipoprotein E	0.18	0.086	1.2E-08	14	47.9
P98160	Basement membrane-specific heparan sulfate proteoglycan core protein	0.18	0.127	1.5E-11	25	7.8
Q07954	Prolow-density lipoprotein receptor-related protein 1	0.18	0.172	7.7E-03	4	1.1
P11166	Solute carrier family 2, facilitated glucose transporter member 1	0.17	0.166	2.3E-05	4	7.3
P21333	Filamin-A	0.17	0.071	0.0E+00	37	19.8
P12110	Collagen alpha-2(VI) chain	0.17	0.128	7.2E-08	7	6.5
P02686	Myelin basic protein	0.17	0.287	4.6E-02	3	10.5
O15230	Laminin subunit alpha-5	0.17	0.146	4.9E-06	11	4.5
P02671	Fibrinogen alpha chain	0.16	0.102	6.3E-04	9	12.5
P60903	Protein S100-A10	0.16	0.100	2.3E-07	4	35.1
P11047	Laminin subunit gamma-1	0.16	0.218	1.8E-04	9	7.0
P01008	Antithrombin-III	0.16	0.143	2.8E-02	3	6.7
P12109	Collagen alpha-1(VI) chain	0.15	0.143	1.1E-05	8	10.1
P02751	Fibronectin	0.14	0.073	2.4E-02	4	2.4
P07355	Annexin A2	0.14	0.061	0.0E+00	22	58.7
P02654	Apolipoprotein C-I	0.13	0.058	1.1E-05	3	24.1
P39060	Collagen alpha-1(XVIII) chain	0.12	0.280	3.3E-04	8	5.2
P01011	Alpha-1-antichymotrypsin	0.12	0.078	6.1E-08	5	12.5
P63211	Guanine nucleotide-binding protein G(T) subunit gamma-T1	0.12	0.357	3.1E-02	3	20.3
P04275	von Willebrand factor	0.12	0.250	2.2E-04	10	4.5
P02760	Protein AMBP	0.11	0.124	4.9E-06	3	12.5
P23946	Chymase	0.11	0.422	5.0E-02	3	16.2
P01871	Ig mu chain C region	0.10	0.217	3.8E-05	8	23.5
P35625	Metalloproteinase inhibitor 3	0.10	0.189	2.9E-08	6	27.0
Q16853	Membrane primary amine oxidase	0.09	0.230	4.0E-02	3	3.7
P35749	Myosin-11	0.09	0.199	7.0E-07	23	14.7
P02730	Band 3 anion transport protein	0.09	0.189	1.7E-03	7	11.6
P08572	Collagen alpha-2(IV) chain	0.09	0.289	2.0E-03	4	3.0
P10643	Complement component C7	0.09	0.160	9.4E-05	4	6.3
P01031	Complement C5	0.08	0.342	1.6E-03	5	2.9
P39059	Collagen alpha-1(XV) chain	0.08	0.104	9.6E-07	3	2.7
P51888	Prolargin	0.08	0.107	0.0E+00	14	41.9
P04083	Annexin A1	0.07	0.188	3.0E-05	12	40.8
P20774	Mimecan	0.07	0.175	6.8E-05	7	22.8
Q14195	Dihydropyrimidinase-related protein 3	0.07	0.162	1.3E-08	3	8.2
P07585	Decorin	0.06	0.221	1.9E-02	7	21.2
P35555	Fibrillin-1	0.06	0.108	2.2E-16	31	13.2
P10909	Clusterin	0.06	0.093	0.0E+00	16	33.4
P22748	Carbonic anhydrase 4	0.06	0.251	5.5E-04	3	10.3
P02748	Complement component C9	0.06	0.194	1.4E-07	11	21.5
Q15661	Tryptase alpha/beta-1	0.06	0.171	8.8E-06	4	17.1
P04004	Vitronectin	0.05	0.158	2.4E-08	11	23.8
P21980	Protein-glutamine gamma-glutamyltransferase 2	0.05	0.150	3.0E-08	14	22.3
P02743	Serum amyloid P-component	0.05	0.356	7.5E-04	5	22.4
P51884	Lumican	0.04	0.138	4.5E-12	11	40.5
P21810	Biglycan	0.04	0.136	2.7E-09	14	41.6
P25189	Myelin protein P0	0.02	0.254	1.9E-04	5	21.8

Brown denotes change ≥ 2 standard deviations (SD) from the mean, yellow denotes change ≥ 1 SD and green highlights p values ≤ 0.05. NA, not applicable, n<3 unique peptides.
