# Supplementary material for: iTRAQ Quantitative Proteomic Comparison of Metastatic and Non-Metastatic Uveal Melanoma Tumors
Source: PLoS One. 2015 Aug 25;10(8):e0135543. doi: 10.1371/journal.pone.0135543 (PMC4549237; doi:10.1371/journal.pone.0135543)
Supplement: S7 Table — (PDF) [file pone.0135543.s007.pdf]

Supplementary Table S7
Relative Protein Abundance: Sample UM20, Non-Metastatic
Total Proteins Quantified = 803; LogMedian Protein Ratio = 0.18; LogMean ProteinRatio = 0; Standard Deviation = 0.78

Uni-Prot Accession	Protein	Ratio UM/Control	Standard Deviation	*p* value	Unique Peptides	% Sequence Coverage
P16402	Histone H1.3	6.53	0.124	2.8E-09	4	10.4
P16401	Histone H1.5	6.41	0.137	1.3E-05	7	15.9
P62937	Peptidyl-prolyl cis-trans isomerase A	5.64	0.117	2.9E-11	7	38.2
Q9BV36	Melanophilin	5.32	0.217	4.3E-03	5	11.7
P08670	Vimentin	5.28	0.031	0.0E+00	38	68.7
O15400	Syntaxin-7	5.18	0.074	2.0E-04	3	11.5
P29401	Transketolase	4.55	0.162	2.1E-06	13	19.9
P30086	Phosphatidylethanolamine-binding protein 1	4.18	0.122	3.7E-05	7	42.8
P04406	Glyceraldehyde-3-phosphate dehydrogenase	4.08	0.085	2.4E-12	12	40.3
P00558	Phosphoglycerate kinase 1	4.07	0.089	3.8E-07	8	16.3
P22087	rRNA 2'-O-methyltransferase fibrillarin	3.93	0.222	4.1E-03	4	13.1
P78417	Glutathione S-transferase omega-1	3.66	0.155	3.0E-05	7	21.2
P04080	Cystatin-B	3.63	0.121	2.5E-04	3	39.8
P30837	Aldehyde dehydrogenase X, mitochondrial	3.53	0.121	4.6E-04	5	14.1
P17931	Galectin-3	3.52	0.096	3.4E-09	7	31.2
P06733	Alpha-enolase	3.48	0.080	1.1E-08	11	34.3
Q13510	Acid ceramidase	3.44	0.125	8.3E-05	4	10.6
P00338	L-lactate dehydrogenase A chain	3.39	0.109	4.6E-04	5	13.3
Q6UVK1	Chondroitin sulfate proteoglycan 4	3.39	0.142	3.2E-05	10	6.6
P51608	Methyl-CpG-binding protein 2	3.34	0.213	2.0E-03	4	11.1
P60174	Triosephosphate isomerase	3.13	0.168	5.0E-02	7	34.3
P07195	L-lactate dehydrogenase B chain	2.98	0.095	1.3E-05	8	21.6
Q99536	Synaptic vesicle membrane protein VAT-1 homolog	2.88	0.102	7.2E-08	11	37.9
P06748	Nucleophosmin	2.88	0.068	1.1E-05	6	17.3
P07339	Cathepsin D	2.86	0.069	1.4E-07	6	13.6
Q13435	Splicing factor 3B subunit 2	2.85	0.238	2.9E-02	4	6.8
Q15907	Ras-related protein Rab-11B	2.84	0.053	6.9E-03	3	14.7
P51149	Ras-related protein Rab-7a	2.83	0.094	9.4E-08	8	44.0
P07910	Heterogeneous nuclear ribonucleoproteins C1/C2	2.78	0.112	4.2E-08	8	21.2
P07737	Profilin-1	2.71	0.211	2.6E-03	3	17.9
Q12906	Interleukin enhancer-binding factor 3	2.68	0.105	2.5E-05	9	11.6
P51159	Ras-related protein Rab-27A	2.67	0.240	9.3E-03	4	20.4
Q3SY69	Mitochondrial 10-formyltetrahydrofolate dehydrogenase	2.66	0.051	5.7E-05	4	4.7
P14618	Pyruvate kinase PKM	2.61	0.067	2.2E-11	10	19.6
Q00059	Transcription factor A, mitochondrial	2.60	0.098	2.7E-04	3	13.8
Q9Y4W6	AFG3-like protein 2	2.55	0.198	6.7E-03	6	8.5
Q99497	Protein DJ-1	2.53	0.291	1.7E-02	3	20.1
P40926	Malate dehydrogenase, mitochondrial	2.48	0.065	3.2E-07	9	29.3
Q13263	Transcription intermediary factor 1-beta	2.46	0.148	1.8E-02	3	2.9
P48681	Nestin	2.39	0.112	5.3E-05	4	3.3
P16070	CD44 antigen	2.39	0.097	2.0E-04	9	10.1
P54652	Heat shock-related 70 kDa protein 2	2.39	0.069	1.0E-04	9	14.2
P31948	Stress-induced-phosphoprotein 1	2.37	0.144	1.8E-03	6	9.8
Q13185	Chromobox protein homolog 3	2.33	0.111	1.5E-05	3	18.0
Q14247	Src substrate cortactin	2.33	0.249	2.3E-02	3	5.5
Q5SSJ5	Heterochromatin protein 1-binding protein 3	2.31	0.069	3.9E-07	5	10.3
P19338	Nucleolin	2.31	0.106	4.8E-07	13	17.5
P23246	Splicing factor, proline- and glutamine-rich	2.26	0.190	4.6E-02	5	7.4
P61604	10 kDa heat shock protein, mitochondrial	2.25	0.174	4.6E-02	4	29.4
Q00839	Heterogeneous nuclear ribonucleoprotein U	2.25	0.103	1.7E-04	8	8.8
P07602	Prosaposin	2.23	0.222	3.5E-02	4	6.9
Q9UKM9	RNA-binding protein Raly	2.22	0.039	1.7E-06	3	13.1
P99999	Cytochrome c	2.21	0.147	3.7E-03	3	24.8
P05204	Non-histone chromosomal protein HMG-17	7.65	NA	NA	2	35.6
Q9NYF8	Bcl-2-associated transcription factor 1	4.37	NA	NA	2	4.2
P17096	High mobility group protein HMG-I/HMG-Y	4.17	NA	NA	2	23.4
O75348	V-type proton ATPase subunit G 1	3.95	NA	NA	2	19.5
P07108	Acyl-CoA-binding protein	3.91	NA	NA	2	41.4
P52565	Rho GDP-dissociation inhibitor 1	3.54	NA	NA	2	14.7
P23381	Tryptophan--tRNA ligase, cytoplasmic	3.53	NA	NA	2	4.9
P10599	Thioredoxin	3.32	NA	NA	2	20.0
P40925	Malate dehydrogenase, cytoplasmic	3.32	NA	NA	2	6.6
P63241	Eukaryotic translation initiation factor 5A-1	3.32	0.346	1.1E-01	4	27.9
P07686	Beta-hexosaminidase subunit beta	3.22	0.297	5.1E-02	3	6.5
P08621	U1 small nuclear ribonucleoprotein 70 kDa	3.21	NA	NA	2	4.3
Q07955	Serine/arginine-rich splicing factor 1	3.19	0.314	1.1E-01	3	8.5
P55809	Succinyl-CoA:3-ketoacid coenzyme A transferase 1, mitochondrial	3.09	NA	NA	2	3.5
P16152	Carbonyl reductase [NADPH] 1	3.05	NA	NA	2	7.6
O95292	Vesicle-associated membrane protein-associated protein B/C	3.02	NA	NA	2	11.1
O14556	Glyceraldehyde-3-phosphate dehydrogenase, testis-specific	2.97	NA	NA	2	6.1
P05141	ADP/ATP translocase 2	2.79	NA	NA	2	7.7
P07954	Fumarate hydratase, mitochondrial	2.77	NA	NA	2	4.3
P06865	Beta-hexosaminidase subunit alpha	2.73	NA	NA	2	3.6
Q01105	Protein SET	2.70	0.533	7.0E-02	3	11.7
Q9Y653	G-protein coupled receptor 56	2.69	0.481	2.3E-01	3	8.1
Q9Y2S2	Lambda-crystallin homolog	2.62	0.295	2.8E-01	3	9.1
Q06323	Proteasome activator complex subunit 1	2.61	0.432	2.2E-01	3	14.5
Q13243	Serine/arginine-rich splicing factor 5	2.54	NA	NA	2	5.9
Q9Y2X3	Nucleolar protein 58	2.54	0.168	8.1E-02	3	8.1
P04181	Ornithine aminotransferase, mitochondrial	2.47	NA	NA	2	5.0
O95336	6-phosphogluconolactonase	2.45	NA	NA	2	8.9
Q13838	Spliceosome RNA helicase DDX39B	2.38	NA	NA	2	5.1
Q16666	Gamma-interferon-inducible protein 16	2.33	NA	NA	2	2.5
P63104	14-3-3 protein zeta/delta	2.32	0.136	7.1E-02	3	18.0
Q5JNZ5	Putative 40S ribosomal protein S26-like 1	2.32	NA	NA	2	20.9
Q9P0L0	Vesicle-associated membrane protein-associated protein A	2.24	NA	NA	2	8.8
Q9NY12	H/ACA ribonucleoprotein complex subunit 1	2.23	NA	NA	2	7.4
Q16576	Histone-binding protein RBBP7	2.22	NA	NA	2	3.5
P13674	Prolyl 4-hydroxylase subunit alpha-1	2.20	NA	NA	2	4.7
P33121	Long-chain-fatty-acid--CoA ligase 1	2.18	0.526	3.7E-01	3	5.3
O60506	Heterogeneous nuclear ribonucleoprotein Q	2.18	0.183	2.9E-03	3	5.8
P22392	Nucleoside diphosphate kinase B	2.17	0.130	1.9E-02	4	30.3
P50395	Rab GDP dissociation inhibitor beta	2.16	0.176	1.0E-02	5	14.2
Q15063	Periostin	2.15	0.192	9.5E-03	6	9.2
O14818	Proteasome subunit alpha type-7	2.15	0.351	1.5E-01	3	13.7
P61247	40S ribosomal protein S3a	2.15	0.334	1.2E-01	6	25.0
P07858	Cathepsin B	2.14	0.175	1.9E-02	4	13.6
P38646	Stress-70 protein, mitochondrial	2.13	0.099	1.3E-07	10	19.4
O43390	Heterogeneous nuclear ribonucleoprotein R	2.12	0.109	1.6E-04	7	11.5
P07900	Heat shock protein HSP 90-alpha	2.11	0.079	3.1E-04	11	14.1

P62750	60S ribosomal protein L23a	2.11	0.110	2.2E-02	3	19.9
P08195	4F2 cell-surface antigen heavy chain	2.10	0.099	1.1E-04	8	16.5
P14866	Heterogeneous nuclear ribonucleoprotein L	2.08	0.099	1.1E-04	5	10.0
P10644	cAMP-dependent protein kinase type I-alpha regulatory subunit	2.06	0.234	9.7E-02	3	5.5
P50402	Emerin	2.06	NA	NA	2	9.8
Q16836	Hydroxyacyl-coenzyme A dehydrogenase, mitochondrial	2.06	NA	NA	2	7.3
P06454	Prothymosin alpha	2.04	NA	NA	2	21.6
P52597	Heterogeneous nuclear ribonucleoprotein F	2.03	0.261	1.0E-01	3	3.1
P23284	Peptidyl-prolyl cis-trans isomerase B	2.02	0.104	5.3E-07	11	41.7
Q16629	Serine/arginine-rich splicing factor 7	2.01	NA	NA	2	7.6
O43809	Cleavage and polyadenylation specificity factor subunit 5	2.00	NA	NA	2	13.2
Q9UI12	V-type proton ATPase subunit H	1.99	NA	NA	2	4.1
P19367	Hexokinase-1	1.99	0.075	4.0E-05	10	11.0
P08758	Annexin A5	1.98	0.072	6.2E-09	13	40.3
P26006	Integrin alpha-3	1.96	0.131	1.5E-02	4	4.5
Q1KMD3	Heterogeneous nuclear ribonucleoprotein U-like protein 2	1.96	0.289	1.7E-01	3	4.3
Q9UKV3	Apoptotic chromatin condensation inducer in the nucleus	1.96	0.255	7.0E-02	4	3.7
P46776	60S ribosomal protein L27a	1.96	0.309	1.1E-01	3	17.6
O75396	Vesicle-trafficking protein SEC22b	1.95	0.086	5.4E-03	4	22.3
Q13151	Heterogeneous nuclear ribonucleoprotein A0	1.94	0.220	6.6E-02	4	9.8
P22626	Heterogeneous nuclear ribonucleoproteins A2/B1	1.94	0.079	4.3E-08	13	30.0
Q9Y696	Chloride intracellular channel protein 4	1.94	NA	NA	2	7.1
Q9GZY8	Mitochondrial fission factor	1.93	NA	NA	2	6.4
O14745	Na(+)/H(+) exchange regulatory cofactor NHE-RF1	1.92	0.746	3.2E-01	3	14.2
Q15631	Translin	1.92	0.260	8.2E-02	3	14.0
P09651	Heterogeneous nuclear ribonucleoprotein A1	1.92	0.144	9.9E-04	7	21.0
O75915	PRA1 family protein 3	1.92	NA	NA	2	9.6
Q86V81	THO complex subunit 4	1.91	NA	NA	2	8.2
P31040	Succinate dehydrogenase [ubiquinone] flavoprotein subunit, mitochondrial	1.90	0.176	3.6E-02	3	3.2
Q9UJZ1	Stomatin-like protein 2, mitochondrial	1.89	NA	NA	2	7.3
Q5JRX3	Presequence protease, mitochondrial	1.89	0.300	5.9E-02	3	4.4
P14314	Glucosidase 2 subunit beta	1.89	0.073	2.9E-06	8	11.6
P08238	Heat shock protein HSP 90-beta	1.89	0.127	1.5E-03	8	11.3
P51991	Heterogeneous nuclear ribonucleoprotein A3	1.89	0.044	4.4E-06	6	17.7
P61421	V-type proton ATPase subunit d 1	1.89	0.092	1.7E-04	3	7.7
Q99879	Histone H2B type 1-M	1.88	NA	NA	2	7.9
Q14108	Lysosome membrane protein 2	1.87	0.127	9.0E-04	5	11.1
P50454	Serpin H1	1.86	0.239	3.0E-02	3	10.0
Q86UX7	Fermitin family homolog 3	1.85	NA	NA	2	3.9
Q12905	Interleukin enhancer-binding factor 2	1.85	NA	NA	2	6.7
Q04760	Lactoylglutathione lyase	1.85	NA	NA	2	10.3
P21912	Succinate dehydrogenase [ubiquinone] iron-sulfur subunit, mitochondrial	1.85	0.042	1.2E-06	3	11.4
O75083	WD repeat-containing protein 1	1.84	0.135	1.1E-02	5	8.6
P40967	Melanocyte protein PMEL	1.84	NA	NA	2	3.3
Q14103	Heterogeneous nuclear ribonucleoprotein D0	1.83	NA	NA	2	6.8
P01857	Ig gamma-1 chain C region	1.83	0.100	1.1E-04	6	30.3
Q96KP4	Cytosolic non-specific dipeptidase	1.82	NA	NA	2	6.5
Q9UH65	Switch-associated protein 70	1.82	NA	NA	2	2.1
P09429	High mobility group protein B1	1.82	0.195	2.2E-02	5	27.4
Q86U42	Polyadenylate-binding protein 2	1.82	NA	NA	2	5.6
Q99798	Aconitate hydratase, mitochondrial	1.82	0.324	4.4E-02	5	7.3
O75436	Vacuolar protein sorting-associated protein 26A	1.81	NA	NA	2	7.0
P30044	Peroxiredoxin-5, mitochondrial	1.80	NA	NA	2	11.7
P02545	Prelamin-A/C	1.80	0.046	4.4E-16	37	51.4
P30101	Protein disulfide-isomerase A3	1.80	0.093	1.8E-05	13	23.8
Q9UHX1	Poly(U)-binding-splicing factor PUF60	1.80	0.312	1.1E-01	3	5.0
Q08211	ATP-dependent RNA helicase A	1.79	0.140	4.4E-03	6	5.7
O14979	Heterogeneous nuclear ribonucleoprotein D-like	1.79	0.061	3.5E-03	3	4.5
P27797	Calreticulin	1.79	0.134	4.5E-02	7	21.1
Q9UMY4	Sorting nexin-12	1.78	NA	NA	2	9.9
P21796	Voltage-dependent anion-selective channel protein 1	1.78	0.083	4.0E-03	6	19.8
Q07020	60S ribosomal protein L18	1.78	0.037	5.2E-06	3	19.7
Q15181	Inorganic pyrophosphatase	1.77	NA	NA	2	6.2
P55786	Puromycin-sensitive aminopeptidase	1.77	0.219	8.5E-02	4	5.1
Q99523	Sortilin	1.76	0.069	3.8E-02	3	6.1
P83731	60S ribosomal protein L24	1.76	0.100	5.2E-04	5	26.8
O94826	Mitochondrial import receptor subunit TOM70	1.76	0.110	8.8E-02	4	6.3
O75390	Citrate synthase, mitochondrial	1.76	0.121	4.2E-02	4	8.4
Q08380	Galectin-3-binding protein	1.76	NA	NA	2	5.0
P53597	Succinyl-CoA ligase [ADP/GDP-forming] subunit alpha, mitochondrial	1.75	NA	NA	2	6.9
Q02318	Sterol 26-hydroxylase, mitochondrial	1.74	NA	NA	2	5.6
Q14139	Ubiquitin conjugation factor E4 A	1.73	NA	NA	2	2.6
P09211	Glutathione S-transferase P	1.73	0.185	4.4E-01	4	25.2
P84103	Serine/arginine-rich splicing factor 3	1.73	NA	NA	2	10.4
P27816	Microtubule-associated protein 4	1.73	NA	NA	2	2.9
Q9UHQ9	NADH-cytochrome b5 reductase 1	1.71	0.112	3.6E-03	4	15.1
P06744	Glucose-6-phosphate isomerase	1.71	0.128	2.4E-02	4	6.8
P49257	Protein ERGIC-53	1.70	0.226	9.9E-02	3	11.4
P18859	ATP synthase-coupling factor 6, mitochondrial	1.70	10.859	5.3E-01	3	30.6
O00567	Nucleolar protein 56	1.70	0.145	4.2E-02	5	7.6
P13639	Elongation factor 2	1.69	0.145	8.9E-03	6	7.6
P10619	Lysosomal protective protein	1.69	NA	NA	2	5.4
Q00341	Vigilin	1.68	NA	NA	2	1.9
O75494	Serine/arginine-rich splicing factor 10	1.68	NA	NA	2	8.8
P17480	Nucleolar transcription factor 1	1.67	NA	NA	2	3.8
Q9BRA2	Thioredoxin domain-containing protein 17	1.66	NA	NA	2	18.7
P25786	Proteasome subunit alpha type-1	1.66	NA	NA	2	6.5
Q13242	Serine/arginine-rich splicing factor 9	1.66	NA	NA	2	9.5
P17844	Probable ATP-dependent RNA helicase DDX5	1.66	0.112	1.2E-02	5	8.6
P21266	Glutathione S-transferase Mu 3	1.65	0.131	4.8E-03	7	28.4
P23528	Cofilin-1	1.65	0.112	2.6E-02	4	25.3
O75533	Splicing factor 3B subunit 1	1.64	NA	NA	2	2.7
Q8TAQ2	SWI/SNF complex subunit SMARCC2	1.64	NA	NA	2	1.7
P11142	Heat shock cognate 71 kDa protein	1.64	0.099	1.6E-02	10	15.9
P38606	V-type proton ATPase catalytic subunit A	1.64	0.119	1.5E-03	5	9.4
P24752	Acetyl-CoA acetyltransferase, mitochondrial	1.64	0.066	1.6E-02	6	16.2
P14678	Small nuclear ribonucleoprotein-associated proteins B and B'	1.64	NA	NA	2	6.3
P51572	B-cell receptor-associated protein 31	1.64	0.265	1.6E-01	7	25.6
P09669	Cytochrome c oxidase subunit 6C	1.64	0.080	1.1E-03	4	48.0
P54727	UV excision repair protein RAD23 homolog B	1.63	NA	NA	2	5.4
Q9NYL4	Peptidyl-prolyl cis-trans isomerase FKBP11	1.63	NA	NA	2	10.4
P13797	Plastin-3	1.63	0.259	4.6E-02	7	12.1
P30049	ATP synthase subunit delta, mitochondrial	1.63	NA	NA	2	13.7
Q15717	ELAV-like protein 1	1.63	0.390	3.8E-01	3	11.7
Q14165	Malectin	1.62	NA	NA	2	4.8
P18669	Phosphoglycerate mutase 1	1.62	0.148	3.7E-02	4	26.8
O95571	Persulfide dioxygenase ETHE1, mitochondrial	1.62	NA	NA	2	12.2

P21281	V-type proton ATPase subunit B, brain isoform	1.62	0.137	7.6E-02	4	9.8
P10809	60 kDa heat shock protein, mitochondrial	1.61	0.186	6.8E-02	8	10.5
P62158	Calmodulin	1.61	0.165	1.6E-02	6	32.9
P61353	60S ribosomal protein L27	1.61	NA	NA	2	22.1
P54819	Adenylate kinase 2, mitochondrial	1.61	0.146	3.4E-01	4	18.8
Q14697	Neutral alpha-glucosidase AB	1.61	0.129	3.4E-04	14	15.6
O00154	Cytosolic acyl coenzyme A thioester hydrolase	1.60	0.159	5.7E-02	4	15.5
Q9BWM7	Sideroflexin-3	1.60	NA	NA	2	8.3
P13473	Lysosome-associated membrane glycoprotein 2	1.60	NA	NA	2	4.9
Q96AG4	Leucine-rich repeat-containing protein 59	1.60	0.264	1.8E-01	3	10.4
Q12874	Splicing factor 3A subunit 3	1.60	NA	NA	2	4.0
P08865	40S ribosomal protein SA	1.60	0.093	3.8E-02	4	11.9
P52272	Heterogeneous nuclear ribonucleoprotein M	1.59	0.069	4.7E-03	6	10.3
P46777	60S ribosomal protein L5	1.59	0.195	2.8E-02	6	22.2
Q13596	Sorting nexin-1	1.59	NA	NA	2	4.6
P39687	Acidic leucine-rich nuclear phosphoprotein 32 family member A	1.59	NA	NA	2	9.6
O43175	D-3-phosphoglycerate dehydrogenase	1.58	0.142	2.9E-02	6	14.8
Q8N2K0	Monoacylglycerol lipase ABHD12	1.58	NA	NA	2	5.3
P42704	Leucine-rich PPR motif-containing protein, mitochondrial	1.58	0.215	4.2E-02	5	3.6
O00483	NADH dehydrogenase [ubiquinone] 1 alpha subcomplex subunit 4	1.58	NA	NA	2	22.2
Q15084	Protein disulfide-isomerase A6	1.57	0.347	2.4E-02	5	13.6
P25398	40S ribosomal protein S12	1.57	NA	NA	2	13.6
O43488	Aflatoxin B1 aldehyde reductase member 2	1.57	NA	NA	2	5.3
P01023	Alpha-2-macroglobulin	1.57	0.071	1.8E-04	13	10.9
P23526	Adenosylhomocysteinase	1.57	NA	NA	2	5.1
P13073	Cytochrome c oxidase subunit 4 isoform 1, mitochondrial	1.57	0.063	1.0E-03	4	25.4
Q9Y6C9	Mitochondrial carrier homolog 2	1.57	NA	NA	2	7.3
Q15233	Non-POU domain-containing octamer-binding protein	1.57	0.412	2.0E-01	6	13.0
P30041	Peroxiredoxin-6	1.57	0.058	1.9E-04	6	20.1
Q86UP2	Kinectin	1.55	0.072	2.8E-03	9	8.0
P47985	Cytochrome b-c1 complex subunit Rieske, mitochondrial	1.55	0.148	1.8E-02	3	11.3
P25788	Proteasome subunit alpha type-3	1.53	0.313	1.8E-01	3	9.4
P51858	Hepatoma-derived growth factor	1.52	NA	NA	2	11.7
Q53EL6	Programmed cell death protein 4	1.52	NA	NA	2	4.7
Q5JTV8	Torsin-1A-interacting protein 1	1.52	0.150	1.7E-01	3	5.7
Q9Y4I1	Unconventional myosin-Va	1.52	0.223	2.2E-02	8	4.6
P49591	Serine--tRNA ligase, cytoplasmic	1.52	0.150	2.4E-02	4	11.7
P16219	Short-chain specific acyl-CoA dehydrogenase, mitochondrial	1.51	NA	NA	2	4.6
O00231	26S proteasome non-ATPase regulatory subunit 11	1.51	0.288	3.3E-01	3	6.6
P60981	Destrin	1.51	NA	NA	2	13.3
P51970	NADH dehydrogenase [ubiquinone] 1 alpha subcomplex subunit 8	1.51	NA	NA	2	8.7
Q9Y6M9	NADH dehydrogenase [ubiquinone] 1 beta subcomplex subunit 9	1.50	NA	NA	2	15.6
P10606	Cytochrome c oxidase subunit 5B, mitochondrial	1.49	0.059	1.6E-02	4	25.6
P62906	60S ribosomal protein L10a	1.49	0.167	2.9E-02	7	29.0
P50914	60S ribosomal protein L14	1.49	NA	NA	2	10.7
P61978	Heterogeneous nuclear ribonucleoprotein K	1.49	0.117	3.3E-03	14	29.8
P07237	Protein disulfide-isomerase	1.48	0.116	3.3E-02	10	15.6
P12270	Nucleoprotein TPR	1.48	0.252	1.7E-01	6	3.7
O75643	U5 small nuclear ribonucleoprotein 200 kDa helicase	1.48	NA	NA	2	0.9
P51810	G-protein coupled receptor 143	1.48	NA	NA	2	5.7
P05387	60S acidic ribosomal protein P2	1.48	0.155	9.1E-02	3	25.2
Q9NX63	Coiled-coil-helix-coiled-coil-helix domain-containing protein 3, mitochondrial	1.48	NA	NA	2	6.6
P61254	60S ribosomal protein L26	1.47	0.125	1.4E-01	3	17.9
Q9BUJ2	Heterogeneous nuclear ribonucleoprotein U-like protein 1	1.47	NA	NA	2	3.4
Q96I99	Succinyl-CoA ligase [GDP-forming] subunit beta, mitochondrial	1.47	NA	NA	2	4.9
P46940	Ras GTPase-activating-like protein IQGAP1	1.47	0.194	1.3E-02	6	5.1
Q15102	Platelet-activating factor acetylhydrolase IB subunit gamma	1.47	0.346	4.0E-01	3	13.0
Q14683	Structural maintenance of chromosomes protein 1A	1.47	NA	NA	2	1.7
P00441	Superoxide dismutase [Cu-Zn]	1.47	NA	NA	2	13.0
P50213	Isocitrate dehydrogenase [NAD] subunit alpha, mitochondrial	1.46	0.091	5.0E-03	4	12.8
Q99653	Calcineurin B homologous protein 1	1.46	0.120	7.2E-02	3	19.0
P07305	Histone H1.0	1.46	0.182	2.0E-01	3	16.0
P62136	Serine/threonine-protein phosphatase PP1-alpha catalytic subunit	1.45	NA	NA	2	8.2
Q9BZQ8	Protein Niban	1.45	NA	NA	2	2.2
Q07666	KH domain-containing, RNA-binding, signal transduction-associated protein 1	1.45	0.055	2.4E-03	4	7.9
P43304	Glycerol-3-phosphate dehydrogenase, mitochondrial	1.45	0.097	1.1E-01	3	5.0
P26640	Valine--tRNA ligase	1.45	NA	NA	2	2.4
Q8NBJ7	Sulfatase-modifying factor 2	1.43	NA	NA	2	4.3
P38919	Eukaryotic initiation factor 4A-III	1.43	NA	NA	2	6.3
Q92896	Golgi apparatus protein 1	1.43	0.276	8.8E-02	4	3.5
P22695	Cytochrome b-c1 complex subunit 2, mitochondrial	1.42	0.125	1.3E-02	5	13.9
Q9Y2B0	Protein canopy homolog 2	1.42	1.038	5.9E-01	3	22.0
P62277	40S ribosomal protein S13	1.42	0.167	5.8E-02	5	34.4
P49411	Elongation factor Tu, mitochondrial	1.42	0.069	4.0E-04	9	20.4
P35232	Prohibitin	1.42	0.044	1.4E-05	8	29.4
P62280	40S ribosomal protein S11	1.42	0.557	5.8E-01	4	13.3
P04075	Fructose-bisphosphate aldolase A	1.41	0.169	4.2E-02	11	36.3
Q8N5K1	CDGSH iron-sulfur domain-containing protein 2	1.41	0.214	1.6E-01	3	23.7
Q9ULV4	Coronin-1C	1.41	NA	NA	2	5.3
P61158	Actin-related protein 3	1.40	0.040	2.6E-04	4	10.8
P37108	Signal recognition particle 14 kDa protein	1.40	NA	NA	2	16.9
Q9P2E9	Ribosome-binding protein 1	1.40	0.249	1.5E-01	4	3.9
O60262	Guanine nucleotide-binding protein G(I)/G(S)/G(O) subunit gamma-7	1.39	NA	NA	2	30.9
Q9BQI0	Allograft inflammatory factor 1-like	1.39	NA	NA	2	10.0
P26599	Polypyrimidine tract-binding protein 1	1.39	0.229	1.0E-01	3	4.5
Q14980	Nuclear mitotic apparatus protein 1	1.39	0.343	6.5E-02	9	6.1
P48047	ATP synthase subunit O, mitochondrial	1.39	0.153	2.6E-01	8	44.1
P62249	40S ribosomal protein S16	1.38	NA	NA	2	15.8
P01834	Ig kappa chain C region	1.38	NA	NA	2	34.9
Q13217	DnaJ homolog subfamily C member 3	1.38	NA	NA	2	3.6
Q16891	Mitochondrial inner membrane protein	1.38	0.139	2.6E-02	9	15.2
P11498	Pyruvate carboxylase, mitochondrial	1.37	NA	NA	2	2.7
Q13162	Peroxiredoxin-4	1.37	0.113	3.9E-01	3	12.9
P38117	Electron transfer flavoprotein subunit beta	1.37	0.124	1.7E-01	5	19.2
P09012	U1 small nuclear ribonucleoprotein A	1.37	0.136	1.0E-01	3	11.0
P0CG05	Ig lambda-2 chain C regions	1.37	NA	NA	2	23.6
Q9UIJ7	GTP:AMP phosphotransferase AK3, mitochondrial	1.37	0.239	3.9E-01	3	15.0
P62263	40S ribosomal protein S14	1.37	0.058	2.9E-02	3	29.1
Q8NBS9	Thioredoxin domain-containing protein 5	1.37	0.134	1.4E-01	6	14.6
P22314	Ubiquitin-like modifier-activating enzyme 1	1.36	0.368	1.7E-01	6	5.7
P00352	Retinal dehydrogenase 1	1.36	0.164	5.0E-02	8	16.4
P00505	Aspartate aminotransferase, mitochondrial	1.35	0.144	1.4E-01	7	17.7
P62851	40S ribosomal protein S25	1.35	0.102	2.2E-02	4	24.0
Q00765	Receptor expression-enhancing protein 5	1.35	3.790	3.0E-01	3	10.6
Q9NZ08	Endoplasmic reticulum aminopeptidase 1	1.35	NA	NA	2	2.2
P39019	40S ribosomal protein S19	1.35	0.088	3.8E-02	3	22.1
Q15691	Microtubule-associated protein RP/EB family member 1	1.34	1.356	3.0E-01	3	9.7

Q99442	Translocation protein SEC62	1.33	0.052	1.2E-02	3	7.3
P04843	Dolichyl-diphosphooligosaccharide--protein glycosyltransferase subunit 1	1.33	0.067	7.7E-04	10	17.8
P12956	X-ray repair cross-complementing protein 6	1.33	0.123	1.2E-02	7	12.0
Q9BS26	Endoplasmic reticulum resident protein 44	1.33	0.092	3.0E-02	4	10.1
P26373	60S ribosomal protein L13	1.33	0.119	2.0E-02	3	14.2
O75964	ATP synthase subunit g, mitochondrial	1.33	NA	NA	2	27.2
P27824	Calnexin	1.32	0.203	6.1E-02	8	15.2
P78527	DNA-dependent protein kinase catalytic subunit	1.32	0.152	1.2E-02	13	2.9
Q9NTJ5	Phosphatidylinositide phosphatase SAC1	1.32	0.105	5.3E-02	6	8.9
P61160	Actin-related protein 2	1.32	0.168	2.6E-02	4	9.9
O43852	Calumenin	1.31	0.256	1.8E-01	4	13.3
Q99584	Protein S100-A13	1.31	0.084	8.1E-03	6	49.0
Q14974	Importin subunit beta-1	1.31	0.256	4.9E-01	4	6.7
P49773	Histidine triad nucleotide-binding protein 1	1.31	NA	NA	2	26.2
O14773	Tripeptidyl-peptidase 1	1.30	NA	NA	2	5.0
P0CW22	40S ribosomal protein S17-like	1.30	0.158	5.7E-02	3	16.3
Q86WV6	Stimulator of interferon genes protein	1.30	NA	NA	2	5.8
P26641	Elongation factor 1-gamma	1.30	0.210	1.6E-01	3	8.9
P37235	Hippocalcin-like protein 1	1.30	NA	NA	2	9.3
Q13011	Delta(3,5)-Delta(2,4)-dienoyl-CoA isomerase, mitochondrial	1.30	0.086	1.2E-02	6	17.7
Q53GQ0	Estradiol 17-beta-dehydrogenase 12	1.30	0.167	3.7E-01	3	11.9
Q9NPA0	ER membrane protein complex subunit 7	1.30	NA	NA	2	12.0
P13010	X-ray repair cross-complementing protein 5	1.30	0.070	8.0E-03	4	6.1
P62899	60S ribosomal protein L31	1.30	NA	NA	2	13.6
P01625	Ig kappa chain V-IV region Len	1.29	NA	NA	2	13.2
O15511	Actin-related protein 2/3 complex subunit 5	1.29	0.265	4.4E-01	3	21.9
Q9NP81	Serine--tRNA ligase, mitochondrial	1.29	NA	NA	2	6.9
P11310	Medium-chain specific acyl-CoA dehydrogenase, mitochondrial	1.29	0.149	2.7E-01	3	6.2
Q16531	DNA damage-binding protein 1	1.29	0.373	3.4E-01	3	2.4
A0FGR8	Extended synaptotagmin-2	1.29	NA	NA	2	2.8
P45954	Short/branched chain specific acyl-CoA dehydrogenase, mitochondrial	1.29	NA	NA	2	5.8
Q96JB5	CDK5 regulatory subunit-associated protein 3	1.29	NA	NA	2	2.6
Q86VP6	Cullin-associated NEDD8-dissociated protein 1	1.29	0.305	3.5E-01	3	2.8
P30711	Glutathione S-transferase theta-1	1.29	NA	NA	2	7.9
P13987	CD59 glycoprotein	1.28	NA	NA	2	15.6
P20700	Lamin-B1	1.28	0.145	2.4E-01	6	10.6
P05362	Intercellular adhesion molecule 1	1.28	0.094	5.8E-02	3	7.3
P46977	Dolichyl-diphosphooligosaccharide--protein glycosyltransferase subunit STT3A	1.28	NA	NA	2	2.3
Q15029	116 kDa U5 small nuclear ribonucleoprotein component	1.28	NA	NA	2	2.6
P06396	Gelsolin	1.28	0.116	7.1E-02	13	17.5
Q8NC56	LEM domain-containing protein 2	1.27	0.129	1.3E-01	3	6.0
Q92820	Gamma-glutamyl hydrolase	1.27	NA	NA	2	7.2
Q02878	60S ribosomal protein L6	1.27	0.075	3.7E-02	6	21.5
P30519	Heme oxygenase 2	1.26	NA	NA	2	10.1
O75367	Core histone macro-H2A.1	1.26	1.104	3.7E-01	6	19.4
Q5VTE0	Putative elongation factor 1-alpha-like 3	1.26	0.213	8.5E-02	7	14.1
P55084	Trifunctional enzyme subunit beta, mitochondrial	1.26	0.070	3.9E-03	11	19.6
Q16695	Histone H3.1t	1.25	0.079	3.6E-02	6	28.7
O95881	Thioredoxin domain-containing protein 12	1.25	NA	NA	2	14.0
O60486	Plexin-C1	1.25	NA	NA	2	1.3
P61204	ADP-ribosylation factor 3	1.24	NA	NA	2	9.9
P42356	Phosphatidylinositol 4-kinase alpha	1.24	NA	NA	2	1.0
P62241	40S ribosomal protein S8	1.24	NA	NA	2	12.0
Q02818	Nucleobindin-1	1.24	NA	NA	2	6.5
O14561	Acyl carrier protein, mitochondrial	1.24	NA	NA	2	9.6
P09543	2',3'-cyclic-nucleotide 3'-phosphodiesterase	1.24	0.107	4.5E-02	6	14.0
P60866	40S ribosomal protein S20	1.24	NA	NA	2	19.3
O95202	LETM1 and EF-hand domain-containing protein 1, mitochondrial	1.24	0.319	1.5E-01	4	5.3
P28331	NADH-ubiquinone oxidoreductase 75 kDa subunit, mitochondrial	1.23	0.172	1.7E-01	3	4.5
Q96I24	Far upstream element-binding protein 3	1.23	NA	NA	2	5.2
P11216	Glycogen phosphorylase, brain form	1.23	0.354	1.1E-01	7	11.2
P55060	Exportin-2	1.23	NA	NA	2	2.3
Q96C19	EF-hand domain-containing protein D2	1.22	NA	NA	2	6.3
P32969	60S ribosomal protein L9	1.22	NA	NA	2	5.7
P35637	RNA-binding protein FUS	1.22	0.564	4.2E-01	3	7.6
P23786	Carnitine O-palmitoyltransferase 2, mitochondrial	1.22	NA	NA	2	3.3
P20674	Cytochrome c oxidase subunit 5A, mitochondrial	1.22	0.930	2.7E-01	4	15.3
P63244	Guanine nucleotide-binding protein subunit beta-2-like 1	1.22	0.113	4.1E-01	3	10.1
Q8IV08	Phospholipase D3	1.22	0.038	1.5E-01	3	6.3
P14854	Cytochrome c oxidase subunit 6B1	1.22	NA	NA	2	23.3
Q99623	Prohibitin-2	1.22	0.106	1.3E-01	5	18.4
P31146	Coronin-1A	1.21	NA	NA	2	4.6
P06576	ATP synthase subunit beta, mitochondrial	1.21	0.057	1.5E-03	15	38.6
P36957	Dihydrolipoyllysine-residue succinyltransferase component of 2-oxoglutarate dehydrogenase complex, mitochondrial	1.21	0.048	4.1E-03	5	11.5
P06899	Histone H2B type 1-J	1.21	NA	NA	2	7.9
P42167	Lamina-associated polypeptide 2, isoforms beta/gamma	1.21	0.364	5.7E-01	3	6.2
O15144	Actin-related protein 2/3 complex subunit 2	1.21	0.241	1.3E-01	6	21.0
P01860	Ig gamma-3 chain C region	1.21	NA	NA	2	6.4
P59998	Actin-related protein 2/3 complex subunit 4	1.21	0.040	5.6E-03	3	16.1
P40939	Trifunctional enzyme subunit alpha, mitochondrial	1.21	0.180	9.3E-02	9	10.9
O00264	Membrane-associated progesterone receptor component 1	1.20	NA	NA	2	11.8
P51665	26S proteasome non-ATPase regulatory subunit 7	1.20	NA	NA	2	6.2
P39023	60S ribosomal protein L3	1.20	NA	NA	2	4.7
P09382	Galectin-1	1.20	0.142	1.9E-02	6	46.7
P04844	Dolichyl-diphosphooligosaccharide--protein glycosyltransferase subunit 2	1.20	NA	NA	2	4.1
Q9P0M6	Core histone macro-H2A.2	1.19	NA	NA	2	7.3
P28838	Cytosol aminopeptidase	1.19	0.145	4.2E-01	3	7.3
Q9NYU2	UDP-glucose:glycoprotein glucosyltransferase 1	1.19	NA	NA	2	1.7
P11021	78 kDa glucose-regulated protein	1.19	0.179	5.4E-02	18	27.5
Q15459	Splicing factor 3A subunit 1	1.19	NA	NA	2	2.9
P25705	ATP synthase subunit alpha, mitochondrial	1.19	0.057	5.6E-03	15	34.4
O75306	NADH dehydrogenase [ubiquinone] iron-sulfur protein 2, mitochondrial	1.19	0.075	5.3E-02	4	11.2
Q9Y3U8	60S ribosomal protein L36	1.18	0.735	4.8E-01	4	30.5
Q9Y5M8	Signal recognition particle receptor subunit beta	1.18	NA	NA	2	7.7
Q9NQC3	Reticulon-4	1.18	NA	NA	2	2.3
P36578	60S ribosomal protein L4	1.18	0.160	2.2E-01	4	9.8
Q6P2Q9	Pre-mRNA-processing-splicing factor 8	1.17	0.177	3.7E-01	3	1.3
P49748	Very long-chain specific acyl-CoA dehydrogenase, mitochondrial	1.17	0.125	1.5E-01	6	9.2
P26038	Moesin	1.17	1.216	2.6E-01	6	9.5
Q92841	Probable ATP-dependent RNA helicase DDX17	1.17	0.156	3.4E-01	4	6.4
P62913	60S ribosomal protein L11	1.17	NA	NA	2	9.0
P62424	60S ribosomal protein L7a	1.17	NA	NA	2	6.4
Q02252	Methylmalonate-semialdehyde dehydrogenase [acylating], mitochondrial	1.16	0.205	4.9E-01	3	5.8
P42765	3-ketoacyl-CoA thiolase, mitochondrial	1.16	1.442	3.7E-01	5	16.6
O43678	NADH dehydrogenase [ubiquinone] 1 alpha subcomplex subunit 2	1.16	0.504	7.6E-01	3	20.2
O75489	NADH dehydrogenase [ubiquinone] iron-sulfur protein 3, mitochondrial	1.16	0.136	1.7E-01	4	14.4
P02768	Serum albumin	1.16	0.133	1.4E-02	33	52.9

Q12797	Aspartyl/asparaginyl beta-hydroxylase	1.15	0.917	3.1E-01	6	7.4
P30050	60S ribosomal protein L12	1.15	NA	NA	2	17.0
P30048	Thioredoxin-dependent peroxide reductase, mitochondrial	1.15	0.420	5.2E-01	5	18.0
P24539	ATP synthase F(0) complex subunit B1, mitochondrial	1.15	NA	NA	2	9.0
Q9C0E8	Protein lunapark	1.15	NA	NA	2	3.7
P11586	C-1-tetrahydrofolate synthase, cytoplasmic	1.15	0.066	2.6E-02	6	7.3
P31153	S-adenosylmethionine synthase isoform type-2	1.14	NA	NA	2	5.6
P24534	Elongation factor 1-beta	1.13	NA	NA	2	7.1
Q9BR76	Coronin-1B	1.13	NA	NA	2	4.1
Q7Z6Z7	E3 ubiquitin-protein ligase HUWE1	1.13	NA	NA	2	0.5
P62805	Histone H4	1.13	0.359	5.6E-02	7	52.4
P31949	Protein S100-A11	1.12	0.529	8.0E-01	3	34.3
P45880	Voltage-dependent anion-selective channel protein 2	1.12	0.167	3.7E-01	6	21.8
P51798	H(+)/Cl(-) exchange transporter 7	1.12	0.485	8.7E-01	3	5.7
P01859	Ig gamma-2 chain C region	1.12	NA	NA	2	6.7
P08237	ATP-dependent 6-phosphofructokinase, muscle type	1.12	0.194	5.7E-01	3	4.2
P11177	Pyruvate dehydrogenase E1 component subunit beta, mitochondrial	1.12	0.923	4.1E-01	3	8.6
P39656	Dolichyl-diphosphooligosaccharide--protein glycosyltransferase 48 kDa subunit	1.12	0.167	1.6E-01	6	12.9
Q92945	Far upstream element-binding protein 2	1.11	0.116	5.8E-01	5	8.0
P30042	ES1 protein homolog, mitochondrial	1.11	NA	NA	2	8.6
P09622	Dihydrolipoyl dehydrogenase, mitochondrial	1.10	1.871	4.5E-01	5	10.6
P22307	Non-specific lipid-transfer protein	1.10	NA	NA	2	3.1
Q9Y230	RuvB-like 2	1.10	NA	NA	2	5.0
Q13423	NAD(P) transhydrogenase, mitochondrial	1.10	0.288	5.8E-01	7	6.8
O15523	ATP-dependent RNA helicase DDX3Y	1.10	NA	NA	2	2.4
P49755	Transmembrane emp24 domain-containing protein 10	1.10	NA	NA	2	9.6
P62258	14-3-3 protein epsilon	1.10	0.252	7.8E-01	7	26.7
P34932	Heat shock 70 kDa protein 4	1.09	0.253	5.1E-01	4	4.2
P02787	Serotransferrin	1.09	0.304	4.8E-01	6	8.5
Q14258	E3 ubiquitin/ISG15 ligase TRIM25	1.09	NA	NA	2	3.8
P29966	Myristoylated alanine-rich C-kinase substrate	1.09	1.159	6.2E-01	3	12.3
Q99714	3-hydroxyacyl-CoA dehydrogenase type-2	1.09	0.081	3.6E-01	3	16.5
P62269	40S ribosomal protein S18	1.09	0.301	3.8E-01	5	23.7
P09525	Annexin A4	1.09	0.249	5.0E-01	5	12.9
Q9Y4L1	Hypoxia up-regulated protein 1	1.08	0.484	8.8E-01	3	3.7
P61026	Ras-related protein Rab-10	1.08	NA	NA	2	9.5
P31930	Cytochrome b-c1 complex subunit 1, mitochondrial	1.08	0.278	7.1E-01	4	9.8
P10515	Dihydrolipoyllysine-residue acetyltransferase component of pyruvate dehydrogenase complex, mitochondrial	1.07	0.044	2.5E-01	5	6.5
Q8WUD1	Ras-related protein Rab-2B	1.07	NA	NA	2	11.1
P30153	Serine/threonine-protein phosphatase 2A 65 kDa regulatory subunit A alpha isoform	1.07	0.357	8.0E-01	3	7.0
P62987	Ubiquitin-60S ribosomal protein L40	1.06	0.422	4.9E-01	7	46.1
P14625	Endoplasmin	1.06	0.409	3.8E-01	14	17.7
P18077	60S ribosomal protein L35a	1.06	NA	NA	2	11.8
P30740	Leukocyte elastase inhibitor	1.06	NA	NA	2	7.7
P15880	40S ribosomal protein S2	1.06	0.541	7.1E-01	4	16.0
O60313	Dynamin-like 120 kDa protein, mitochondrial	1.06	0.942	7.8E-01	4	4.7
Q03252	Lamin-B2	1.06	1.821	2.4E-01	16	23.5
O75947	ATP synthase subunit d, mitochondrial	1.05	0.280	8.4E-01	5	28.6
P30084	Enoyl-CoA hydratase, mitochondrial	1.05	NA	NA	2	4.5
Q9BRX8	Redox-regulatory protein FAM213A	1.05	0.288	8.4E-01	3	13.1
Q9HCC0	Methylcrotonoyl-CoA carboxylase beta chain, mitochondrial	1.05	NA	NA	2	6.0
O95298	NADH dehydrogenase [ubiquinone] 1 subunit C2	1.04	NA	NA	2	16.8
P11940	Polyadenylate-binding protein 1	1.04	0.438	5.1E-01	5	9.9
P54136	Arginine--tRNA ligase, cytoplasmic	1.03	1.195	9.1E-01	3	5.5
P00367	Glutamate dehydrogenase 1, mitochondrial	1.03	0.588	6.8E-01	6	12.5
Q969X5	Endoplasmic reticulum-Golgi intermediate compartment protein 1	1.03	0.736	7.4E-01	4	14.1
P54920	Alpha-soluble NSF attachment protein	1.03	0.271	8.3E-01	6	22.7
Q14789	Golgin subfamily B member 1	1.03	0.711	7.9E-01	3	1.4
O94919	Endonuclease domain-containing 1 protein	1.03	0.290	7.4E-01	4	8.6
P61586	Transforming protein RhoA	1.03	0.155	7.9E-01	3	10.4
P06703	Protein S100-A6	1.03	NA	NA	2	16.7
Q9P2R7	Succinyl-CoA ligase [ADP-forming] subunit beta, mitochondrial	1.03	NA	NA	2	3.9
P01889	HLA class I histocompatibility antigen, B-7 alpha chain	1.01	NA	NA	2	9.4
P78371	T-complex protein 1 subunit beta	1.01	1.712	9.3E-01	5	12.0
P29692	Elongation factor 1-delta	1.01	0.308	9.7E-01	3	8.5
P14927	Cytochrome b-c1 complex subunit 7	1.01	NA	NA	2	22.5
Q96QK1	Vacuolar protein sorting-associated protein 35	1.01	NA	NA	2	3.0
P0C0S5	Histone H2A.Z	1.00	NA	NA	2	18.8
P67936	Tropomyosin alpha-4 chain	1.00	0.549	9.9E-01	8	25.0
P60953	Cell division control protein 42 homolog	1.00	NA	NA	2	11.0
Q05682	Caldesmon	0.99	1.895	9.9E-01	3	6.4
O60763	General vesicular transport factor p115	0.99	NA	NA	2	1.6
P01876	Ig alpha-1 chain C region	0.99	NA	NA	2	4.5
Q9BSJ8	Extended synaptotagmin-1	0.99	0.310	9.4E-01	4	4.4
Q96JJ7	Protein disulfide-isomerase TMX3	0.99	NA	NA	2	6.8
P18124	60S ribosomal protein L7	0.98	1.707	6.5E-01	5	15.7
Q6DD88	Atlastin-3	0.98	0.214	8.7E-01	4	7.4
P13667	Protein disulfide-isomerase A4	0.98	0.451	7.2E-01	5	7.9
P06737	Glycogen phosphorylase, liver form	0.97	0.176	7.2E-01	5	6.4
P05388	60S acidic ribosomal protein P0	0.97	NA	NA	2	6.6
O95302	Peptidyl-prolyl cis-trans isomerase FKBP9	0.97	NA	NA	2	4.0
P63000	Ras-related C3 botulinum toxin substrate 1	0.96	1.729	6.8E-01	4	24.5
P61006	Ras-related protein Rab-8A	0.96	NA	NA	2	7.2
O75340	Programmed cell death protein 6	0.96	1.551	9.4E-01	3	11.5
P09874	Poly [ADP-ribose] polymerase 1	0.95	0.137	5.3E-01	4	4.4
Q00325	Phosphate carrier protein, mitochondrial	0.95	0.355	7.8E-01	5	13.5
P51812	Ribosomal protein S6 kinase alpha-3	0.95	NA	NA	2	2.3
P62701	40S ribosomal protein S4, X isoform	0.94	0.380	7.6E-01	4	13.3
P23396	40S ribosomal protein S3	0.94	0.196	6.0E-01	5	19.3
Q8IUX7	Adipocyte enhancer-binding protein 1	0.94	0.064	2.6E-01	3	4.2
P62917	60S ribosomal protein L8	0.94	NA	NA	2	10.5
P46781	40S ribosomal protein S9	0.94	34.152	7.6E-01	4	14.9
Q9UHG3	Prenylcysteine oxidase 1	0.93	0.115	5.9E-01	3	6.3
Q9Y5X3	Sorting nexin-5	0.93	NA	NA	2	3.7
P47756	F-actin-capping protein subunit beta	0.93	0.875	5.7E-01	3	12.6
O75521	Enoyl-CoA delta isomerase 2, mitochondrial	0.92	NA	NA	2	5.6
P26196	Probable ATP-dependent RNA helicase DDX6	0.92	NA	NA	2	4.1
Q9H444	Charged multivesicular body protein 4b	0.92	NA	NA	2	8.0
P62753	40S ribosomal protein S6	0.92	0.549	5.5E-01	4	15.7
Q00577	Transcriptional activator protein Pur-alpha	0.91	NA	NA	2	4.7
P40429	60S ribosomal protein L13a	0.91	NA	NA	2	9.9
Q02218	2-oxoglutarate dehydrogenase, mitochondrial	0.91	0.122	2.9E-01	5	5.3
P29590	Protein PML	0.91	3.227	5.2E-01	3	4.3
P49368	T-complex protein 1 subunit gamma	0.91	0.136	2.0E-01	7	12.1
P04040	Catalase	0.91	0.386	7.6E-01	4	9.5
P62244	40S ribosomal protein S15a	0.91	0.096	3.3E-01	4	29.2
Q9UNH7	Sorting nexin-6	0.90	0.240	4.8E-01	3	4.7

Q7KZF4	Staphylococcal nuclease domain-containing protein 1	0.90	0.811	7.2E-01	3	5.4
P02790	Hemopexin	0.90	1.570	8.3E-01	3	8.9
Q14764	Major vault protein	0.90	0.151	2.4E-01	4	4.7
P35573	Glycogen debranching enzyme	0.90	NA	NA	2	2.1
P47897	Glutamine--tRNA ligase	0.89	NA	NA	2	2.6
P46939	Utrophin	0.88	0.105	1.8E-01	4	1.8
O94906	Pre-mRNA-processing factor 6	0.88	NA	NA	2	2.1
Q16795	NADH dehydrogenase [ubiquinone] 1 alpha subcomplex subunit 9, mitochondrial	0.88	0.223	5.4E-01	3	9.8
Q04637	Eukaryotic translation initiation factor 4 gamma 1	0.87	NA	NA	2	1.1
Q15365	Poly(rC)-binding protein 1	0.87	NA	NA	2	5.3
P12235	ADP/ATP translocase 1	0.87	NA	NA	2	6.7
P15586	N-acetylglucosamine-6-sulfatase	0.87	NA	NA	2	3.3
P14868	Aspartate--tRNA ligase, cytoplasmic	0.86	1.111	4.3E-01	4	9.8
P48643	T-complex protein 1 subunit epsilon	0.86	0.104	1.1E-01	6	9.8
P27361	Mitogen-activated protein kinase 3	0.86	0.553	6.7E-01	3	9.8
P26368	Splicing factor U2AF 65 kDa subunit	0.86	NA	NA	2	3.8
Q6NZI2	Polymerase I and transcript release factor	0.86	0.181	4.5E-01	7	19.5
P37837	Transaldolase	0.86	NA	NA	2	4.2
O75746	Calcium-binding mitochondrial carrier protein Aralar1	0.86	NA	NA	2	4.6
P55072	Transitional endoplasmic reticulum ATPase	0.84	0.054	6.9E-04	11	15.9
Q92499	ATP-dependent RNA helicase DDX1	0.84	0.102	7.1E-02	3	3.9
Q93009	Ubiquitin carboxyl-terminal hydrolase 7	0.84	NA	NA	2	1.8
Q27J81	Inverted formin-2	0.84	NA	NA	2	1.6
Q9UHD8	Septin-9	0.84	0.097	6.0E-02	4	7.8
Q6NUK1	Calcium-binding mitochondrial carrier protein SCaMC-1	0.84	NA	NA	2	4.4
P43490	Nicotinamide phosphoribosyltransferase	0.83	NA	NA	2	3.1
P06753	Tropomyosin alpha-3 chain	0.83	2.099	4.4E-01	4	13.0
Q9Y4G6	Talin-2	0.83	NA	NA	2	1.3
Q99733	Nucleosome assembly protein 1-like 4	0.83	NA	NA	2	3.5
P48735	Isocitrate dehydrogenase [NADP], mitochondrial	0.83	0.098	2.2E-01	4	10.6
Q7L5N1	COP9 signalosome complex subunit 6	0.82	0.168	2.4E-01	3	10.1
P50990	T-complex protein 1 subunit theta	0.82	0.040	4.2E-03	6	10.4
P08559	Pyruvate dehydrogenase E1 component subunit alpha, somatic form, mitochondrial	0.82	26.290	5.5E-01	3	8.2
O15212	Prefoldin subunit 6	0.82	NA	NA	2	14.0
P50991	T-complex protein 1 subunit delta	0.82	0.068	3.1E-02	4	9.3
Q14152	Eukaryotic translation initiation factor 3 subunit A	0.82	NA	NA	2	2.2
Q9Y305	Acyl-coenzyme A thioesterase 9, mitochondrial	0.81	NA	NA	2	5.5
B5ME19	Eukaryotic translation initiation factor 3 subunit C-like protein	0.81	0.126	1.8E-01	3	3.1
Q9Y6N5	Sulfide:quinone oxidoreductase, mitochondrial	0.81	0.176	2.5E-01	5	10.7
P54709	Sodium/potassium-transporting ATPase subunit beta-3	0.81	6.700	5.6E-01	3	11.8
Q9NTZ6	RNA-binding protein 12	0.81	NA	NA	2	3.3
P52907	F-actin-capping protein subunit alpha-1	0.81	NA	NA	2	10.5
Q8NFI4	Putative protein FAM10A5	0.80	NA	NA	2	5.1
P05556	Integrin beta-1	0.80	0.092	1.3E-02	6	8.0
Q13200	26S proteasome non-ATPase regulatory subunit 2	0.80	0.154	2.1E-01	4	4.2
Q9Y2J2	Band 4.1-like protein 3	0.80	NA	NA	2	3.1
P49593	Protein phosphatase 1F	0.79	NA	NA	2	7.5
P08107	Heat shock 70 kDa protein 1A/1B	0.79	0.044	7.7E-03	11	17.9
P02652	Apolipoprotein A-II	0.78	NA	NA	2	11.0
Q9Y5S2	Serine/threonine-protein kinase MRCK beta	0.78	0.206	2.7E-01	3	1.7
Q99832	T-complex protein 1 subunit eta	0.77	0.050	5.8E-03	5	10.5
P09496	Clathrin light chain A	0.77	NA	NA	2	5.6
Q07065	Cytoskeleton-associated protein 4	0.76	0.079	3.5E-03	6	13.1
P16615	Sarcoplasmic/endoplasmic reticulum calcium ATPase 2	0.76	0.103	4.0E-02	7	7.4
Q15149	Plectin	0.75	0.031	8.0E-12	52	11.8
P05091	Aldehyde dehydrogenase, mitochondrial	0.75	0.030	2.7E-04	3	6.2
P40227	T-complex protein 1 subunit zeta	0.75	NA	NA	2	3.8
P42025	Beta-centractin	0.74	NA	NA	2	5.1
P50995	Annexin A11	0.74	0.114	5.6E-02	5	9.5
P35221	Catenin alpha-1	0.74	0.131	9.0E-02	4	5.5
P17987	T-complex protein 1 subunit alpha	0.74	0.034	1.3E-03	5	9.7
Q9HBL0	Tensin-1	0.74	7.343	6.1E-01	5	5.0
Q9NZ45	CDGSH iron-sulfur domain-containing protein 1	0.73	0.086	5.5E-02	3	29.6
Q16851	UTP--glucose-1-phosphate uridylyltransferase	0.73	NA	NA	2	4.5
O43707	Alpha-actinin-4	0.73	0.085	2.7E-03	13	16.4
P05023	Sodium/potassium-transporting ATPase subunit alpha-1	0.73	0.038	7.1E-05	17	18.3
O94979	Protein transport protein Sec31A	0.72	0.136	1.2E-01	3	3.4
P60709	Actin, cytoplasmic 1	0.72	0.075	1.5E-02	6	24.8
Q63ZY3	KN motif and ankyrin repeat domain-containing protein 2	0.71	1.079	4.7E-01	3	5.2
O94905	Erlin-2	0.71	0.082	1.4E-03	5	11.8
O95865	N(G),N(G)-dimethylarginine dimethylaminohydrolase 2	0.71	0.081	4.0E-02	3	11.9
P17655	Calpain-2 catalytic subunit	0.71	NA	NA	2	3.6
P00387	NADH-cytochrome b5 reductase 3	0.71	0.118	1.1E-02	6	20.6
Q9Y6G9	Cytoplasmic dynein 1 light intermediate chain 1	0.71	NA	NA	2	4.2
Q06830	Peroxiredoxin-1	0.71	0.104	5.5E-03	7	33.2
P43307	Translocon-associated protein subunit alpha	0.70	NA	NA	2	6.6
P46937	Yorkie homolog	0.70	NA	NA	2	6.7
P69905	Hemoglobin subunit alpha	0.70	0.122	6.6E-03	5	34.5
Q14558	Phosphoribosyl pyrophosphate synthase-associated protein 1	0.70	NA	NA	2	8.4
Q9Y6C2	EMILIN-1	0.69	0.096	3.7E-03	8	9.2
O95782	AP-2 complex subunit alpha-1	0.68	NA	NA	2	1.8
P09497	Clathrin light chain B	0.68	NA	NA	2	7.4
P35222	Catenin beta-1	0.68	0.046	1.6E-02	5	7.4
O14735	CDP-diacylglycerol--inositol 3-phosphatidyltransferase	0.68	NA	NA	2	9.9
O60716	Catenin delta-1	0.68	NA	NA	2	2.0
Q9NZN4	EH domain-containing protein 2	0.67	0.080	8.9E-03	3	5.7
P13861	cAMP-dependent protein kinase type II-alpha regulatory subunit	0.67	0.078	5.6E-02	4	13.6
Q14203	Dynactin subunit 1	0.67	0.027	5.2E-03	4	4.2
O43301	Heat shock 70 kDa protein 12A	0.66	0.181	4.8E-02	3	5.5
Q15293	Reticulocalbin-1	0.66	NA	NA	2	4.5
Q9BTV4	Transmembrane protein 43	0.65	0.250	7.7E-02	4	11.5
P49458	Signal recognition particle 9 kDa protein	0.65	NA	NA	2	22.1
Q9Y3I0	tRNA-splicing ligase RtcB homolog	0.64	NA	NA	2	4.6
O43242	26S proteasome non-ATPase regulatory subunit 3	0.64	NA	NA	2	2.8
Q8IVF2	Protein AHNAK2	0.64	2.199	8.0E-01	3	0.3
Q02952	A-kinase anchor protein 12	0.64	0.190	1.7E-02	19	13.9
P53621	Coatomer subunit alpha	0.63	0.260	2.5E-01	3	3.5
P18206	Vinculin	0.62	0.045	5.8E-07	14	15.3
P31942	Heterogeneous nuclear ribonucleoprotein H3	0.61	NA	NA	2	8.4
P05026	Sodium/potassium-transporting ATPase subunit beta-1	0.61	NA	NA	2	5.9
P02042	Hemoglobin subunit delta	0.61	NA	NA	2	17.7
P11532	Dystrophin	0.61	NA	NA	2	0.6
P04179	Superoxide dismutase [Mn], mitochondrial	0.60	0.083	2.7E-03	4	14.0
Q01484	Ankyrin-2	0.60	NA	NA	2	0.5
P43121	Cell surface glycoprotein MUC18	0.60	NA	NA	2	3.4
P17612	cAMP-dependent protein kinase catalytic subunit alpha	0.60	0.131	2.6E-02	3	6.8
P12814	Alpha-actinin-1	0.58	0.082	4.9E-05	7	10.1

Q14204	Cytoplasmic dynein 1 heavy chain 1	0.58	0.052	1.8E-07	19	4.4
P32119	Peroxiredoxin-2	0.58	0.083	1.4E-04	4	14.1
O94832	Unconventional myosin-Id	0.58	NA	NA	2	1.9
Q13561	Dynactin subunit 2	0.58	0.080	8.5E-02	4	11.7
P63208	S-phase kinase-associated protein 1	0.58	NA	NA	2	13.5
Q00610	Clathrin heavy chain 1	0.57	0.052	7.7E-08	18	11.8
P01009	Alpha-1-antitrypsin	0.57	0.238	1.5E-02	8	17.5
Q9BQE3	Tubulin alpha-1C chain	0.56	NA	NA	2	6.7
Q14254	Flotillin-2	0.56	NA	NA	2	4.2
O75131	Copine-3	0.55	0.024	1.6E-06	4	6.7
Q15942	Zyxin	0.55	NA	NA	2	5.2
Q14956	Transmembrane glycoprotein NMB	0.54	0.363	8.2E-02	3	5.8
P51659	Peroxisomal multifunctional enzyme type 2	0.54	NA	NA	2	3.0
P55884	Eukaryotic translation initiation factor 3 subunit B	0.54	0.161	3.8E-02	3	3.3
P04083	Annexin A1	0.54	0.076	9.0E-09	13	40.8
Q16181	Septin-7	0.53	0.034	2.0E-06	4	9.4
P23634	Plasma membrane calcium-transporting ATPase 4	0.53	0.060	3.6E-03	3	4.0
P35580	Myosin-10	0.53	0.097	1.0E-04	11	6.7
P04899	Guanine nucleotide-binding protein G(i) subunit alpha-2	0.52	0.200	2.6E-02	4	13.8
Q9Y490	Talin-1	0.52	0.034	1.6E-11	20	9.7
P04632	Calpain small subunit 1	0.52	0.509	3.5E-01	3	8.6
P04792	Heat shock protein beta-1	0.52	0.056	1.8E-04	5	31.2
P07360	Complement component C8 gamma chain	0.51	NA	NA	2	15.8
P68371	Tubulin beta-4B chain	0.51	NA	NA	2	7.2
Q15019	Septin-2	0.51	0.098	6.4E-04	4	13.9
Q13813	Spectrin alpha chain, non-erythrocytic 1	0.51	0.027	0.0E+00	65	27.5
Q969G5	Protein kinase C delta-binding protein	0.50	0.223	2.8E-02	3	11.5
Q13418	Integrin-linked protein kinase	0.50	NA	NA	2	3.8
Q14344	Guanine nucleotide-binding protein subunit alpha-13	0.50	0.133	5.9E-03	4	11.1
Q13884	Beta-1-syntrophin	0.50	NA	NA	2	2.6
P07358	Complement component C8 beta chain	0.49	NA	NA	2	3.4
P02511	Alpha-crystallin B chain	0.49	0.069	4.5E-05	5	30.9
P06756	Integrin alpha-V	0.49	NA	NA	2	1.4
P68871	Hemoglobin subunit beta	0.49	0.082	3.5E-06	5	44.9
P08133	Annexin A6	0.49	0.061	2.0E-13	25	42.8
P04196	Histidine-rich glycoprotein	0.48	NA	NA	2	3.6
P00390	Glutathione reductase, mitochondrial	0.48	NA	NA	2	6.3
P04217	Alpha-1B-glycoprotein	0.48	NA	NA	2	4.8
Q09666	Neuroblast differentiation-associated protein AHNAK	0.48	0.039	7.2E-12	45	6.1
P36405	ADP-ribosylation factor-like protein 3	0.47	NA	NA	2	16.5
Q01082	Spectrin beta chain, non-erythrocytic 1	0.47	0.031	0.0E+00	42	20.1
P21333	Filamin-A	0.47	0.036	5.9E-11	44	20.9
Q9BUF5	Tubulin beta-6 chain	0.46	NA	NA	2	4.3
Q16363	Laminin subunit alpha-4	0.41	0.270	9.8E-02	5	3.0
O14950	Myosin regulatory light chain 12B	0.40	NA	NA	2	12.2
O94911	ATP-binding cassette sub-family A member 8	0.40	NA	NA	2	1.2
Q03591	Complement factor H-related protein 1	0.40	NA	NA	2	9.4
P00738	Haptoglobin	0.39	0.108	8.4E-02	5	12.6
P01911	HLA class II histocompatibility antigen, DRB1-15 beta chain	0.38	NA	NA	2	6.4
P60201	Myelin proteolipid protein	0.37	NA	NA	2	5.4
P63010	AP-2 complex subunit beta	0.35	NA	NA	2	1.8
Q15582	Transforming growth factor-beta-induced protein ig-h3	0.33	NA	NA	2	3.4
P0DJI8	Serum amyloid A-1 protein	0.33	NA	NA	2	20.5
P36269	Gamma-glutamyltransferase 5	0.32	NA	NA	2	3.9
Q9NZN3	EH domain-containing protein 3	0.32	NA	NA	2	3.9
Q9BS40	Latexin	0.32	NA	NA	2	12.2
Q5JRA6	Melanoma inhibitory activity protein 3	0.28	NA	NA	2	1.2
Q13642	Four and a half LIM domains protein 1	0.28	NA	NA	2	5.6
P04216	Thy-1 membrane glycoprotein	0.28	NA	NA	2	15.5
P58166	Inhibin beta E chain	0.28	NA	NA	2	6.6
P23229	Integrin alpha-6	0.28	NA	NA	2	1.5
Q14624	Inter-alpha-trypsin inhibitor heavy chain H4	0.26	NA	NA	2	1.9
P13671	Complement component C6	0.26	NA	NA	2	2.6
P61764	Syntaxin-binding protein 1	0.25	NA	NA	2	2.9
O14495	Lipid phosphate phosphohydrolase 3	0.25	NA	NA	2	7.4
P61626	Lysozyme C	0.24	NA	NA	2	12.8
P22413	Ectonucleotide pyrophosphatase/phosphodiesterase family member 1	0.24	0.402	1.2E-01	4	5.2
P05164	Myeloperoxidase	0.22	NA	NA	2	2.7
P43320	Beta-crystallin B2	0.21	NA	NA	2	11.7
P16452	Erythrocyte membrane protein band 4.2	0.21	NA	NA	2	2.7
Q6UXB8	Peptidase inhibitor 16	0.21	NA	NA	2	5.2
P23946	Chymase	0.19	NA	NA	2	11.7
P02760	Protein AMBP	0.18	NA	NA	2	7.7
P10643	Complement component C7	0.17	NA	NA	2	3.6
P05186	Alkaline phosphatase, tissue-nonspecific isozyme	0.16	0.201	5.2E-02	4	7.8
Q9NY15	Stabilin-1	0.16	NA	NA	2	0.6
P26447	Protein S100-A4	0.16	NA	NA	2	18.8
P24844	Myosin regulatory light polypeptide 9	0.15	NA	NA	2	12.2
Q92777	Synapsin-2	0.14	NA	NA	2	4.1
P02686	Myelin basic protein	0.13	NA	NA	2	7.2
Q9BXM0	Periaxin	0.12	0.303	1.6E-01	5	3.5
P80723	Brain acid soluble protein 1	0.12	NA	NA	2	11.9
P08123	Collagen alpha-2(I) chain	0.09	NA	NA	2	2.4
O00159	Unconventional myosin-Ic	0.44	0.065	1.7E-06	7	7.2
Q07954	Prolow-density lipoprotein receptor-related protein 1	0.43	0.076	9.7E-04	8	2.5
P00167	Cytochrome b5	0.41	0.043	5.4E-05	3	35.8
P06727	Apolipoprotein A-IV	0.40	0.079	2.3E-04	5	13.4
P02647	Apolipoprotein A-I	0.39	0.082	1.0E-03	5	18.7
P09493	Tropomyosin alpha-1 chain	0.39	0.212	1.6E-03	5	12.0
P41219	Peripherin	0.39	0.229	4.8E-04	11	22.6
O43491	Band 4.1-like protein 2	0.38	0.094	1.8E-02	6	8.8
O00468	Agrin	0.36	0.158	1.5E-02	3	1.8
P60660	Myosin light polypeptide 6	0.36	0.030	0.0E+00	8	58.3
O15230	Laminin subunit alpha-5	0.35	0.078	4.7E-05	12	4.2
P55268	Laminin subunit beta-2	0.35	0.071	2.5E-09	13	8.6
O75369	Filamin-B	0.35	0.157	9.2E-03	8	4.5
P62736	Actin, aortic smooth muscle	0.35	0.146	3.1E-04	6	21.0
P07099	Epoxide hydrolase 1	0.34	0.182	3.7E-02	5	11.0
P35579	Myosin-9	0.34	0.033	0.0E+00	48	24.7
Q9NZM1	Myoferlin	0.34	0.066	3.7E-07	7	3.5
P16157	Ankyrin-1	0.33	0.145	4.0E-03	3	1.9
P12110	Collagen alpha-2(VI) chain	0.33	0.190	2.0E-04	6	5.7
P01903	HLA class II histocompatibility antigen, DR alpha chain	0.33	0.139	1.5E-02	3	14.6
P02679	Fibrinogen gamma chain	0.33	0.127	1.1E-04	10	17.0
P27105	Erythrocyte band 7 integral membrane protein	0.32	0.043	9.9E-07	5	15.6
P46821	Microtubule-associated protein 1B	0.32	0.118	4.2E-03	4	1.7
P09936	Ubiquitin carboxyl-terminal hydrolase isozyme L1	0.31	0.185	1.4E-02	4	21.5

P00747	Plasminogen	0.30	0.129	2.1E-04	7	11.1
O94875	Sorbin and SH3 domain-containing protein 2	0.30	0.233	2.5E-02	3	4.7
P00450	Ceruloplasmin	0.30	0.082	9.7E-08	7	9.6
P07197	Neurofilament medium polypeptide	0.30	0.175	5.9E-03	4	6.6
P07355	Annexin A2	0.30	0.049	0.0E+00	22	56.6
P01011	Alpha-1-antichymotrypsin	0.29	0.074	7.4E-08	8	16.5
P02649	Apolipoprotein E	0.29	0.073	8.7E-07	13	40.1
P07942	Laminin subunit beta-1	0.29	0.163	3.9E-04	5	3.2
P12111	Collagen alpha-3(VI) chain	0.29	0.053	5.3E-13	27	9.7
Q16555	Dihydropyrimidinase-related protein 2	0.29	0.065	1.4E-07	8	17.0
P01024	Complement C3	0.29	0.143	5.4E-05	13	8.7
Q05707	Collagen alpha-1(XIV) chain	0.27	0.155	8.0E-04	7	4.1
P11047	Laminin subunit gamma-1	0.27	0.092	1.2E-06	10	6.6
Q01995	Transgelin	0.27	0.131	1.2E-02	4	17.9
P11166	Solute carrier family 2, facilitated glucose transporter member 1	0.27	0.073	6.8E-07	3	5.5
P02675	Fibrinogen beta chain	0.27	0.051	1.4E-10	8	23.2
P12109	Collagen alpha-1(VI) chain	0.27	0.064	1.2E-09	9	9.5
P98160	Basement membrane-specific heparan sulfate proteoglycan core protein	0.26	0.060	0.0E+00	27	8.0
Q14112	Nidogen-2	0.26	0.103	1.2E-08	10	7.7
P08572	Collagen alpha-2(IV) chain	0.26	0.196	8.0E-04	5	3.7
P02549	Spectrin alpha chain, erythrocytic 1	0.25	0.113	3.0E-06	6	3.3
P08294	Extracellular superoxide dismutase [Cu-Zn]	0.25	0.151	5.9E-03	3	15.4
P50895	Basal cell adhesion molecule	0.25	0.067	3.6E-03	3	7.0
P02751	Fibronectin	0.23	0.090	2.6E-07	12	6.2
P02671	Fibrinogen alpha chain	0.22	0.088	1.2E-06	7	9.8
Q2UY09	Collagen alpha-1(XXVIII) chain	0.22	0.199	3.6E-03	3	2.8
P14543	Nidogen-1	0.21	0.117	2.5E-05	8	7.4
P60903	Protein S100-A10	0.21	0.135	3.7E-03	4	35.1
P02749	Beta-2-glycoprotein 1	0.20	0.100	2.2E-05	3	9.6
Q14699	Raftlin	0.20	0.277	1.9E-02	3	5.5
P02654	Apolipoprotein C-I	0.20	0.105	5.0E-05	3	24.1
P04275	von Willebrand factor	0.19	0.089	9.6E-11	11	4.4
Q12805	EGF-containing fibulin-like extracellular matrix protein 1	0.19	0.124	7.9E-04	3	5.7
P39059	Collagen alpha-1(XV) chain	0.19	0.108	4.5E-06	4	2.9
P01871	Ig mu chain C region	0.19	0.177	5.8E-04	8	19.0
P02730	Band 3 anion transport protein	0.19	0.109	5.9E-05	6	8.6
Q14195	Dihydropyrimidinase-related protein 3	0.18	0.135	1.3E-03	5	12.8
P39060	Collagen alpha-1(XVIII) chain	0.18	0.103	5.0E-08	7	4.2
P35555	Fibrillin-1	0.18	0.032	0.0E+00	38	14.1
P35749	Myosin-11	0.18	0.060	6.2E-15	27	14.5
P11277	Spectrin beta chain, erythrocytic	0.18	0.114	9.3E-03	5	3.3
P07585	Decorin	0.17	0.095	1.5E-04	5	11.4
P51888	Prolargin	0.17	0.066	0.0E+00	12	34.0
P02743	Serum amyloid P-component	0.17	0.106	8.7E-08	6	23.3
P01008	Antithrombin-III	0.16	0.116	1.7E-05	5	11.4
Q15661	Tryptase alpha/beta-1	0.16	0.083	5.5E-05	5	21.8
P20774	Mimecan	0.15	0.102	8.8E-13	8	28.2
P02748	Complement component C9	0.15	0.102	5.4E-06	8	13.8
P22352	Glutathione peroxidase 3	0.15	0.074	1.1E-09	4	14.6
P21926	CD9 antigen	0.15	0.089	6.8E-07	3	9.6
P35625	Metalloproteinase inhibitor 3	0.15	0.061	4.7E-13	4	15.6
P10909	Clusterin	0.15	0.050	0.0E+00	12	24.1
P04004	Vitronectin	0.14	0.096	5.7E-11	8	16.1
P10745	Retinol-binding protein 3	0.14	0.233	2.4E-02	3	2.4
P21980	Protein-glutamine gamma-glutamyltransferase 2	0.14	0.071	1.8E-12	12	16.7
P01031	Complement C5	0.14	0.180	1.5E-05	3	1.6
P15088	Mast cell carboxypeptidase A	0.13	0.139	2.2E-03	4	7.9
P22748	Carbonic anhydrase 4	0.13	0.106	6.2E-07	6	16.0
P21810	Biglycan	0.12	0.097	3.9E-08	8	23.9
P51884	Lumican	0.12	0.042	0.0E+00	7	20.4
P25189	Myelin protein P0	0.11	0.084	1.8E-10	7	27.4

Brown denotes change ≥ 2 standard deviations (SD) from the mean, yellow denotes change ≥ 1 SD and green highlights p values ≤ 0.05. NA, not applicable, n<3 unique peptides.
