# Supplementary material for: iTRAQ Quantitative Proteomic Comparison of Metastatic and Non-Metastatic Uveal Melanoma Tumors
Source: PLoS One. 2015 Aug 25;10(8):e0135543. doi: 10.1371/journal.pone.0135543 (PMC4549237; doi:10.1371/journal.pone.0135543)
Supplement: S8 Table — (PDF) [file pone.0135543.s008.pdf]

Supplementary Table S8
Relative Protein Abundance: Sample UM23, Non-Metastatic
Total Proteins Quantified = 910; LogMedian Protein Ratio = 0.15; LogMean Protein Ratio = 0; Standard Deviation = 0.92

Uni-Prot Accession	Protein	Ratio UM/Control	Standard Deviation	*p* value	Unique Peptides	% Sequence Coverage
P62328	Thymosin beta-4	9.78	0.297	1.0E-03	3	45.5
P17096	High mobility group protein HMG-I/HMG-Y	9.49	0.151	9.7E-05	4	41.1
P62937	Peptidyl-prolyl cis-trans isomerase A	9.02	0.103	0.0E+00	7	38.2
P30086	Phosphatidylethanolamine-binding protein 1	8.57	0.161	4.1E-09	9	50.3
O14556	Glyceraldehyde-3-phosphate dehydrogenase, testis-specific	8.45	0.192	3.5E-06	5	14.5
P10599	Thioredoxin	6.73	0.252	2.6E-04	3	32.4
P60174	Triosephosphate isomerase	6.48	0.130	1.1E-06	11	50.0
O75368	SH3 domain-binding glutamic acid-rich-like protein	6.11	0.143	5.2E-08	3	21.1
P55769	NHP2-like protein 1	6.10	0.161	5.9E-03	3	24.2
P09211	Glutathione S-transferase P	5.97	0.127	4.0E-09	7	38.1
Q04760	Lactoylglutathione lyase	5.76	0.146	3.7E-10	6	22.8
P06733	Alpha-enolase	5.49	0.061	2.7E-15	13	36.4
P00558	Phosphoglycerate kinase 1	5.10	0.075	0.0E+00	15	28.3
Q9HC38	Glyoxalase domain-containing protein 4	5.04	0.123	8.4E-05	3	10.5
P52565	Rho GDP-dissociation inhibitor 1	4.96	0.177	9.1E-05	3	15.2
P00338	L-lactate dehydrogenase A chain	4.87	0.095	3.7E-08	9	22.3
P78417	Glutathione S-transferase omega-1	4.78	0.145	9.4E-05	7	27.0
P07195	L-lactate dehydrogenase B chain	4.77	0.071	1.7E-14	9	24.3
P61604	10 kDa heat shock protein, mitochondrial	4.44	0.141	4.0E-04	6	51.0
P24941	Cyclin-dependent kinase 2	4.39	0.305	7.7E-03	6	23.8
P40926	Malate dehydrogenase, mitochondrial	4.28	0.069	2.4E-15	11	39.3
P04080	Cystatin-B	4.00	0.108	1.9E-09	4	49.0
P19338	Nucleolin	3.95	0.079	1.1E-13	16	17.3
P0DME0	Protein SETSIP	3.78	0.151	3.1E-05	4	15.6
Q9Y6U3	Adseverin	3.72	0.162	4.3E-04	9	11.7
P12955	Xaa-Pro dipeptidase	3.70	0.086	3.6E-05	5	9.1
P51858	Hepatoma-derived growth factor	3.70	0.167	1.4E-03	3	15.8
P16401	Histone H1.5	3.67	0.169	1.4E-03	6	15.9
P99999	Cytochrome c	3.63	0.149	2.7E-04	3	24.8
P13797	Plastin-3	3.61	0.152	9.2E-06	9	15.4
P09429	High mobility group protein B1	3.53	0.170	3.8E-04	6	25.6
Q16658	Fascin	3.52	0.050	2.1E-05	3	8.1
P52815	39S ribosomal protein L12, mitochondrial	3.42	0.139	2.3E-02	3	11.6
P16152	Carbonyl reductase [NADPH] 1	3.35	0.130	6.7E-05	4	16.6
P16219	Short-chain specific acyl-CoA dehydrogenase, mitochondrial	3.32	0.164	2.9E-02	6	16.0
P63241	Eukaryotic translation initiation factor 5A-1	3.23	0.209	8.8E-04	6	27.9
P25786	Proteasome subunit alpha type-1	3.14	0.177	4.0E-03	5	16.0
P23526	Adenosylhomocysteinase	3.14	0.194	2.2E-04	6	16.7
P04406	Glyceraldehyde-3-phosphate dehydrogenase	3.11	0.066	1.1E-10	11	35.8
O14818	Proteasome subunit alpha type-7	3.10	0.089	1.5E-02	5	20.6
P22087	rRNA 2'-O-methyltransferase fibrillarin	3.09	0.115	2.2E-03	6	19.6
P06748	Nucleophosmin	3.09	0.105	1.1E-05	7	20.1
P21291	Cysteine and glycine-rich protein 1	3.08	0.153	5.4E-03	3	21.8
Q06323	Proteasome activator complex subunit 1	3.05	0.163	1.1E-03	7	25.7
P07737	Profilin-1	2.97	0.150	2.3E-03	5	37.9
Q00796	Sorbitol dehydrogenase	2.95	0.060	6.2E-04	4	9.0
P63104	14-3-3 protein zeta/delta	2.91	0.090	1.1E-02	7	34.7
P29401	Transketolase	2.90	0.123	8.6E-04	13	19.4
O75531	Barrier-to-autointegration factor	2.83	0.115	1.9E-05	3	29.2
P08758	Annexin A5	2.81	0.050	0.0E+00	16	44.4
O95336	6-phosphogluconolactonase	2.80	0.109	2.0E-03	3	14.7
P31948	Stress-induced-phosphoprotein 1	2.79	0.095	2.6E-08	9	13.6
P23528	Cofilin-1	2.79	0.068	2.8E-04	7	49.4
P50453	Serpin B9	2.75	0.131	7.5E-04	6	21.0
O14618	Copper chaperone for superoxide dismutase	2.74	0.334	1.5E-02	3	10.9
Q9H4A4	Aminopeptidase B	2.72	0.031	2.3E-03	3	4.5
P52566	Rho GDP-dissociation inhibitor 2	2.68	0.076	3.3E-03	3	21.9
Q08380	Galectin-3-binding protein	2.67	0.219	3.1E-03	4	8.0
Q00059	Transcription factor A, mitochondrial	2.66	0.193	2.1E-03	4	17.9
Q07955	Serine/arginine-rich splicing factor 1	2.65	0.140	1.8E-05	5	17.3
Q96KP4	Cytosolic non-specific dipeptidase	2.65	0.101	1.1E-06	7	20.0
Q14847	LIM and SH3 domain protein 1	2.63	0.344	2.1E-03	4	14.6
P62750	60S ribosomal protein L23a	2.63	0.054	1.7E-03	4	26.9
P02768	Serum albumin	2.63	0.023	0.0E+00	39	60.6
O00299	Chloride intracellular channel protein 1	2.62	0.188	4.0E-02	3	10.0
Q03154	Aminoacylase-1	2.62	0.153	4.0E-03	3	8.1
P40925	Malate dehydrogenase, cytoplasmic	2.60	0.080	1.4E-05	3	10.5
P51149	Ras-related protein Rab-7a	2.58	0.066	3.5E-09	6	30.0
Q9NR28	Diablo homolog, mitochondrial	2.54	0.055	4.6E-07	4	18.4
P18669	Phosphoglycerate mutase 1	2.53	0.079	1.3E-08	4	23.6
P16402	Histone H1.3	2.53	0.099	1.6E-06	6	15.4
P14174	Macrophage migration inhibitory factor	17.68	NA	NA	2	17.4
P06454	Prothymosin alpha	14.05	0.248	9.4E-02	3	21.6
Q6EEV6	Small ubiquitin-related modifier 4	7.63	NA	NA	2	23.2
O00625	Pirin	6.41	NA	NA	2	6.2
P07108	Acyl-CoA-binding protein	5.68	NA	NA	2	41.4
Q15102	Platelet-activating factor acetylhydrolase IB subunit gamma	4.72	NA	NA	2	9.1
Q12931	Heat shock protein 75 kDa, mitochondrial	3.84	NA	NA	2	4.4
O60220	Mitochondrial import inner membrane translocase subunit Tim8 A	3.75	NA	NA	2	22.7
P16083	Ribosyldihydronicotinamide dehydrogenase [quinone]	3.72	NA	NA	2	9.1
P07919	Cytochrome b-c1 complex subunit 6, mitochondrial	3.48	NA	NA	2	15.4
P36959	GMP reductase 1	3.13	NA	NA	2	7.0
P62310	U6 snRNA-associated Sm-like protein LSm3	3.11	NA	NA	2	19.6
P62826	GTP-binding nuclear protein Ran	3.09	0.494	3.1E-01	3	10.2
P25788	Proteasome subunit alpha type-3	3.06	0.299	5.5E-02	4	13.7
P63279	SUMO-conjugating enzyme UBC9	3.02	NA	NA	2	16.5
P00441	Superoxide dismutase [Cu-Zn]	3.01	NA	NA	2	13.0
P40121	Macrophage-capping protein	2.96	0.197	1.1E-01	3	8.3
Q9BUP0	EF-hand domain-containing protein D1	2.95	0.324	6.5E-02	4	26.4
P16949	Stathmin	2.95	NA	NA	2	14.8
P60900	Proteasome subunit alpha type-6	2.88	0.411	1.1E-01	3	16.3
Q15819	Ubiquitin-conjugating enzyme E2 variant 2	2.87	NA	NA	2	13.1
Q13243	Serine/arginine-rich splicing factor 5	2.80	NA	NA	2	5.9
O15400	Syntaxin-7	2.79	0.241	4.4E-01	3	11.5
P82979	SAP domain-containing ribonucleoprotein	2.77	NA	NA	2	11.4
P07741	Adenine phosphoribosyltransferase	2.74	NA	NA	2	10.0
P02042	Hemoglobin subunit delta	2.73	NA	NA	2	17.7
P15531	Nucleoside diphosphate kinase A	2.69	NA	NA	2	13.2
P27695	DNA-(apurinic or apyrimidinic site) lyase	2.69	NA	NA	2	5.3
O95834	Echinoderm microtubule-associated protein-like 2	2.68	NA	NA	2	4.0
O75347	Tubulin-specific chaperone A	2.60	NA	NA	2	20.4
P25789	Proteasome subunit alpha type-4	2.59	NA	NA	2	6.5
Q86UE4	Protein LYRIC	2.58	NA	NA	2	4.0
P20042	Eukaryotic translation initiation factor 2 subunit 2	2.57	0.357	1.4E-01	3	13.2
P31939	Bifunctional purine biosynthesis protein PURH	2.55	NA	NA	2	3.4
P49720	Proteasome subunit beta type-3	2.54	NA	NA	2	7.8
P16930	Fumarylacetoacetase	2.54	NA	NA	2	5.0
Q9BQI0	Allograft inflammatory factor 1-like	2.53	NA	NA	2	15.3

Q8IV08	Phospholipase D3	2.53	NA	NA	2	4.3
P40967	Melanocyte protein PMEL	2.51	NA	NA	2	3.3
P60842	Eukaryotic initiation factor 4A-I	2.51	0.153	1.5E-04	5	12.8
P54819	Adenylate kinase 2, mitochondrial	2.50	0.118	6.7E-04	3	14.2
P07900	Heat shock protein HSP 90-alpha	2.49	0.053	3.3E-12	14	16.8
P39748	Flap endonuclease 1	2.47	NA	NA	2	4.5
P69905	Hemoglobin subunit alpha	2.45	0.054	1.5E-13	9	71.1
P07910	Heterogeneous nuclear ribonucleoproteins C1/C2	2.44	0.089	2.4E-06	8	21.2
P02787	Serotransferrin	2.43	0.083	2.6E-08	12	18.8
Q53EL6	Programmed cell death protein 4	2.43	0.127	5.4E-03	7	14.5
P30085	UMP-CMP kinase	2.43	NA	NA	2	10.7
P07954	Fumarate hydratase, mitochondrial	2.40	0.131	3.2E-03	6	13.1
Q16576	Histone-binding protein RBBP7	2.40	NA	NA	2	3.5
Q9UL46	Proteasome activator complex subunit 2	2.40	0.083	1.8E-03	3	21.3
P30044	Peroxiredoxin-5, mitochondrial	2.39	NA	NA	2	11.7
Q92597	Protein NDRG1	2.38	0.230	1.7E-02	5	16.8
Q13185	Chromobox protein homolog 3	2.37	0.097	1.8E-03	3	18.0
P31937	3-hydroxyisobutyrate dehydrogenase, mitochondrial	2.36	NA	NA	2	9.2
Q13126	S-methyl-5'-thioadenosine phosphorylase	2.35	0.439	1.3E-01	3	9.5
P78324	Tyrosine-protein phosphatase non-receptor type substrate 1	2.33	NA	NA	2	5.6
P38646	Stress-70 protein, mitochondrial	2.33	0.088	1.0E-10	14	20.5
P08238	Heat shock protein HSP 90-beta	2.32	0.077	2.5E-09	11	11.7
P15121	Aldose reductase	2.32	0.197	9.6E-03	6	19.0
P0C0S8	Histone H2A type 1	2.32	NA	NA	2	23.1
P14618	Pyruvate kinase PKM	2.31	0.036	0.0E+00	8	15.4
P01857	Ig gamma-1 chain C region	2.31	0.108	1.0E-06	6	31.5
P28066	Proteasome subunit alpha type-5	2.29	0.171	2.6E-02	3	17.4
P05387	60S acidic ribosomal protein P2	2.29	0.112	6.5E-05	5	32.2
P16070	CD44 antigen	2.28	0.101	1.2E-03	8	11.1
P23381	Tryptophan--tRNA ligase, cytoplasmic	2.27	0.188	1.6E-01	3	8.5
P13489	Ribonuclease inhibitor	2.27	0.105	4.8E-04	3	7.8
P62857	40S ribosomal protein S28	2.25	NA	NA	2	30.4
Q9Y4W6	AFG3-like protein 2	2.24	0.100	3.5E-05	9	12.7
P68871	Hemoglobin subunit beta	2.23	0.056	2.0E-11	6	51.0
P24534	Elongation factor 1-beta	2.22	0.089	2.9E-03	3	9.8
Q9Y2X3	Nucleolar protein 58	2.21	0.139	3.8E-01	3	5.9
Q13510	Acid ceramidase	2.20	0.093	2.2E-04	5	12.4
P29590	Protein PML	2.20	0.073	1.8E-06	11	12.1
Q13228	Selenium-binding protein 1	2.20	0.089	1.3E-04	5	10.0
Q9Y2B0	Protein canopy homolog 2	2.20	0.070	6.7E-02	4	23.6
Q9UNF0	Protein kinase C and casein kinase substrate in neurons protein 2	2.20	0.170	1.1E-02	4	9.3
P01620	Ig kappa chain V-III region SIE	2.19	NA	NA	2	22.9
P06744	Glucose-6-phosphate isomerase	2.19	0.100	1.1E-05	6	10.9
P20591	Interferon-induced GTP-binding protein Mx1	2.18	0.217	8.6E-02	5	10.3
Q9Y2S2	Lambda-crystallin homolog	2.17	0.215	3.8E-02	3	9.1
Q14103	Heterogeneous nuclear ribonucleoprotein D0	2.16	0.087	1.1E-05	3	9.0
O14979	Heterogeneous nuclear ribonucleoprotein D-like	2.16	0.096	6.1E-06	3	4.5
O94826	Mitochondrial import receptor subunit TOM70	2.14	0.124	9.5E-03	6	10.0
Q99798	Aconitate hydratase, mitochondrial	2.13	0.077	1.9E-05	9	14.7
Q15942	Zyxin	2.13	0.495	5.8E-02	3	8.7
P13693	Translationally-controlled tumor protein	2.11	NA	NA	2	15.7
P04792	Heat shock protein beta-1	2.10	0.056	2.1E-10	8	29.8
P57729	Ras-related protein Rab-38	2.10	0.149	3.5E-03	4	18.0
P46926	Glucosamine-6-phosphate isomerase 1	2.10	0.112	4.8E-03	7	18.7
O00567	Nucleolar protein 56	2.09	0.235	7.9E-02	4	6.6
P25398	40S ribosomal protein S12	2.08	0.079	8.8E-04	4	33.3
P08195	4F2 cell-surface antigen heavy chain	2.08	0.087	8.9E-06	7	13.3
O00148	ATP-dependent RNA helicase DDX39A	2.08	0.064	1.1E-05	3	6.8
Q96GK7	Fumarylacetoacetate hydrolase domain-containing protein 2A	2.07	0.478	9.3E-02	3	14.3
P20618	Proteasome subunit beta type-1	2.07	0.112	1.1E-03	5	23.7
P30042	ES1 protein homolog, mitochondrial	2.06	0.240	1.2E-01	4	20.5
P12270	Nucleoprotein TPR	2.06	0.051	7.1E-05	5	3.2
Q9Y5K6	CD2-associated protein	2.06	0.854	3.8E-01	3	7.0
P29966	Myristoylated alanine-rich C-kinase substrate	2.05	0.263	7.5E-03	6	29.8
P62158	Calmodulin	2.04	0.160	8.8E-03	4	29.5
Q14247	Src substrate cortactin	2.04	0.265	7.2E-02	5	6.9
P35613	Basigin	2.04	0.179	8.6E-04	3	10.9
P27816	Microtubule-associated protein 4	2.03	0.256	2.2E-02	5	5.6
Q92945	Far upstream element-binding protein 2	2.00	0.112	4.6E-05	6	8.7
O75390	Citrate synthase, mitochondrial	1.99	0.124	2.3E-02	4	8.4
Q9NX63	Coiled-coil-helix-coiled-coil-helix domain-containing protein 3, mitochondrial	1.98	0.129	1.3E-02	3	12.3
P13798	Acylamino-acid-releasing enzyme	1.98	0.010	4.5E-03	3	4.6
Q14677	Clathrin interactor 1	1.97	NA	NA	2	3.4
P21796	Voltage-dependent anion-selective channel protein 1	1.97	0.062	1.5E-03	6	19.8
P31949	Protein S100-A11	1.96	0.087	1.4E-03	3	32.4
P06753	Tropomyosin alpha-3 chain	1.96	0.085	3.6E-07	4	13.0
P61106	Ras-related protein Rab-14	1.95	NA	NA	2	7.9
Q9NY12	H/ACA ribonucleoprotein complex subunit 1	1.95	NA	NA	2	7.4
Q9UQ80	Proliferation-associated protein 2G4	1.94	NA	NA	2	5.1
P55786	Puromycin-sensitive aminopeptidase	1.94	0.183	3.6E-02	4	4.8
P62851	40S ribosomal protein S25	1.93	0.054	5.4E-07	5	29.6
P30485	HLA class I histocompatibility antigen, B-47 alpha chain	1.93	0.091	5.1E-04	3	11.0
Q16543	Hsp90 co-chaperone Cdc37	1.93	NA	NA	2	5.3
P51608	Methyl-CpG-binding protein 2	1.93	NA	NA	2	5.1
O75348	V-type proton ATPase subunit G 1	1.92	0.174	2.0E-02	3	28.8
O75874	Isocitrate dehydrogenase [NADP] cytoplasmic	1.92	NA	NA	2	5.3
P14550	Alcohol dehydrogenase [NADP(+)]	1.91	NA	NA	2	7.1
Q13428	Treacle protein	1.91	NA	NA	2	1.6
Q9NRX4	14 kDa phosphohistidine phosphatase	1.91	NA	NA	2	25.6
P28072	Proteasome subunit beta type-6	1.90	NA	NA	2	9.2
P50395	Rab GDP dissociation inhibitor beta	1.90	0.135	4.0E-02	7	18.2
Q9UKY7	Protein CDV3 homolog	1.90	NA	NA	2	6.2
P10809	60 kDa heat shock protein, mitochondrial	1.89	0.110	9.9E-05	14	22.0
Q02790	Peptidyl-prolyl cis-trans isomerase FKBP4	1.89	NA	NA	2	4.8
O43852	Calumenin	1.88	0.075	2.0E-04	6	17.5
Q14240	Eukaryotic initiation factor 4A-II	1.87	0.174	2.6E-02	3	8.8
Q9UKV3	Apoptotic chromatin condensation inducer in the nucleus	1.87	NA	NA	2	1.6
Q12906	Interleukin enhancer-binding factor 3	1.87	0.103	3.5E-04	8	10.5
Q13011	Delta(3,5)-Delta(2,4)-dienoyl-CoA isomerase, mitochondrial	1.86	0.069	9.6E-07	5	15.2
P13639	Elongation factor 2	1.86	0.047	1.3E-09	11	14.5
P39687	Acidic leucine-rich nuclear phosphoprotein 32 family member A	1.85	0.146	6.5E-03	4	17.3
P30512	HLA class I histocompatibility antigen, A-29 alpha chain	1.85	0.315	3.3E-01	3	10.7
Q9UJZ1	Stomatin-like protein 2, mitochondrial	1.85	0.185	1.1E-01	3	12.1
O75083	WD repeat-containing protein 1	1.85	0.124	4.0E-03	4	6.8
P18621	60S ribosomal protein L17	1.85	0.280	3.6E-02	5	19.6
Q9BXK5	Bcl-2-like protein 13	1.84	NA	NA	2	4.3
Q14165	Malectin	1.84	0.122	3.1E-03	4	11.6
Q13277	Syntaxin-3	1.84	NA	NA	2	4.8
Q01130	Serine/arginine-rich splicing factor 2	1.83	NA	NA	2	6.8
Q99497	Protein DJ-1	1.82	0.242	6.2E-02	4	20.1
P49773	Histidine triad nucleotide-binding protein 1	1.81	0.166	3.4E-01	4	42.9
P04075	Fructose-bisphosphate aldolase A	1.80	0.067	3.0E-04	14	45.1
P29692	Elongation factor 1-delta	1.79	0.101	1.5E-02	3	11.4
P08107	Heat shock 70 kDa protein 1A/1B	1.79	0.053	6.9E-05	15	22.0
Q13435	Splicing factor 3B subunit 2	1.79	NA	NA	2	4.4

Q9UH65	Switch-associated protein 70	1.78	0.150	5.8E-02	3	4.8
P02458	Collagen alpha-1(II) chain	1.78	NA	NA	2	1.5
P50502	Hsc70-interacting protein	1.78	0.115	2.9E-02	5	10.8
P42224	Signal transducer and activator of transcription 1-alpha/beta	1.77	NA	NA	2	3.6
Q8NBS9	Thioredoxin domain-containing protein 5	1.77	0.051	9.4E-04	5	12.7
O15533	Tapasin	1.76	NA	NA	2	4.2
P50213	Isocitrate dehydrogenase [NAD] subunit alpha, mitochondrial	1.75	0.080	1.1E-04	4	12.8
Q96I99	Succinyl-CoA ligase [GDP-forming] subunit beta, mitochondrial	1.75	NA	NA	2	4.6
P35232	Prohibitin	1.74	0.046	8.7E-10	9	32.7
P08574	Cytochrome c1, heme protein, mitochondrial	1.73	NA	NA	2	7.1
P11216	Glycogen phosphorylase, brain form	1.73	0.105	1.8E-05	7	10.4
Q9Y371	Endophilin-B1	1.73	NA	NA	2	5.8
P08865	40S ribosomal protein SA	1.73	0.061	2.4E-05	6	25.4
Q96AG4	Leucine-rich repeat-containing protein 59	1.73	0.101	6.9E-04	3	9.4
Q3ZCQ8	Mitochondrial import inner membrane translocase subunit TIM50	1.72	NA	NA	2	8.5
P30405	Peptidyl-prolyl cis-trans isomerase F, mitochondrial	1.72	NA	NA	2	9.2
Q02818	Nucleobindin-1	1.71	0.081	7.6E-05	7	17.1
P23246	Splicing factor, proline- and glutamine-rich	1.71	0.164	2.4E-01	6	8.6
P46776	60S ribosomal protein L27a	1.70	NA	NA	2	14.2
P37802	Transgelin-2	1.70	NA	NA	2	10.1
Q06830	Peroxiredoxin-1	1.69	0.061	3.5E-08	8	44.2
O15173	Membrane-associated progesterone receptor component 2	1.67	0.239	4.6E-02	3	18.4
P09914	Interferon-induced protein with tetratricopeptide repeats 1	1.66	NA	NA	2	6.3
Q53H82	Beta-lactamase-like protein 2	1.66	NA	NA	2	6.3
Q8NFV4	Alpha/beta hydrolase domain-containing protein 11	1.66	NA	NA	2	8.6
P27797	Calreticulin	1.65	0.094	6.7E-03	8	16.1
P26641	Elongation factor 1-gamma	1.65	0.221	3.3E-02	4	8.9
Q99536	Synaptic vesicle membrane protein VAT-1 homolog	1.65	0.083	1.3E-04	11	39.2
B5ME19	Eukaryotic translation initiation factor 3 subunit C-like protein	1.64	0.052	2.2E-03	4	4.3
P01834	Ig kappa chain C region	1.64	NA	NA	2	34.9
Q13263	Transcription intermediary factor 1-beta	1.64	0.159	2.6E-02	4	5.5
O43399	Tumor protein D54	1.63	0.089	3.7E-03	3	16.0
P42704	Leucine-rich PPR motif-containing protein, mitochondrial	1.63	0.110	3.1E-04	9	6.2
P31040	Succinate dehydrogenase [ubiquinone] flavoprotein subunit, mitochondrial	1.63	0.339	8.8E-02	4	7.4
P09651	Heterogeneous nuclear ribonucleoprotein A1	1.62	0.051	9.0E-08	6	16.7
P23284	Peptidyl-prolyl cis-trans isomerase B	1.62	0.069	5.4E-08	9	35.6
P0CG05	Ig lambda-2 chain C regions	1.62	NA	NA	2	23.6
Q5VTE0	Putative elongation factor 1-alpha-like 3	1.62	0.124	6.6E-03	10	20.3
Q9UMY4	Sorting nexin-12	1.61	NA	NA	2	11.0
Q92688	Acidic leucine-rich nuclear phosphoprotein 32 family member B	1.61	0.284	2.3E-01	4	17.1
P62263	40S ribosomal protein S14	1.61	0.089	3.4E-03	4	36.4
P83731	60S ribosomal protein L24	1.60	0.099	1.4E-03	4	25.5
P31153	S-adenosylmethionine synthase isoform type-2	1.59	0.279	2.9E-01	3	9.4
P09874	Poly [ADP-ribose] polymerase 1	1.59	0.098	1.5E-03	9	10.4
P25685	DnaJ homolog subfamily B member 1	1.59	0.124	1.9E-02	4	10.0
Q14974	Importin subunit beta-1	1.59	2.466	2.6E-01	4	6.7
P51991	Heterogeneous nuclear ribonucleoprotein A3	1.58	0.064	3.4E-07	6	17.7
P51532	Transcription activator BRG1	1.58	0.535	4.3E-01	3	2.1
Q15907	Ras-related protein Rab-11B	1.58	0.099	3.0E-03	6	26.6
Q04446	1,4-alpha-glucan-branching enzyme	1.57	NA	NA	2	4.4
P61204	ADP-ribosylation factor 3	1.57	NA	NA	2	10.5
Q1KMD3	Heterogeneous nuclear ribonucleoprotein U-like protein 2	1.56	0.114	7.8E-02	5	6.8
P28838	Cytosol aminopeptidase	1.56	0.241	5.1E-02	8	19.3
P42167	Lamina-associated polypeptide 2, isoforms beta/gamma	1.56	0.335	4.9E-01	4	11.0
O43809	Cleavage and polyadenylation specificity factor subunit 5	1.56	0.512	2.2E-01	3	16.7
P30041	Peroxiredoxin-6	1.55	0.059	2.1E-05	6	19.6
O15145	Actin-related protein 2/3 complex subunit 3	1.55	NA	NA	2	9.6
P61978	Heterogeneous nuclear ribonucleoprotein K	1.55	0.051	5.6E-08	12	28.3
P28062	Proteasome subunit beta type-8	1.55	NA	NA	2	10.1
Q14152	Eukaryotic translation initiation factor 3 subunit A	1.55	0.111	4.1E-02	5	4.7
Q9NSE4	Isoleucine--tRNA ligase, mitochondrial	1.55	0.094	4.9E-03	8	11.7
Q92616	Translational activator GCN1	1.54	0.100	7.0E-02	5	1.6
P49411	Elongation factor Tu, mitochondrial	1.54	0.036	7.9E-09	9	21.5
O75400	Pre-mRNA-processing factor 40 homolog A	1.54	NA	NA	2	1.7
Q9UNZ2	NSFL1 cofactor p47	1.54	0.378	3.2E-01	3	11.1
Q9UHX1	Poly(U)-binding-splicing factor PUF60	1.54	0.071	9.5E-03	4	6.8
P62906	60S ribosomal protein L10a	1.54	0.089	5.6E-04	6	27.6
P34932	Heat shock 70 kDa protein 4	1.54	0.136	5.1E-02	5	6.4
O60814	Histone H2B type 1-K	1.53	NA	NA	2	7.9
O60506	Heterogeneous nuclear ribonucleoprotein Q	1.53	0.114	7.5E-02	3	5.6
P53597	Succinyl-CoA ligase [ADP/GDP-forming] subunit alpha, mitochondrial	1.52	0.211	1.9E-01	4	12.4
P43243	Matrin-3	1.52	NA	NA	2	3.8
P62241	40S ribosomal protein S8	1.52	NA	NA	2	9.1
Q13423	NAD(P) transhydrogenase, mitochondrial	1.52	0.162	1.6E-02	7	6.3
P46783	40S ribosomal protein S10	1.51	0.189	1.0E-01	3	20.0
P35268	60S ribosomal protein L22	1.51	NA	NA	2	18.8
Q86TX2	Acyl-coenzyme A thioesterase 1	1.51	NA	NA	2	5.2
P26885	Peptidyl-prolyl cis-trans isomerase FKBP2	1.51	0.276	2.3E-01	3	14.1
P78347	General transcription factor II-I	1.50	0.963	4.0E-01	3	3.0
P35659	Protein DEK	1.50	0.192	1.7E-01	4	13.6
P22314	Ubiquitin-like modifier-activating enzyme 1	1.50	0.147	1.1E-01	4	4.3
Q9BZZ5	Apoptosis inhibitor 5	1.50	0.299	2.2E-01	3	5.9
Q00839	Heterogeneous nuclear ribonucleoprotein U	1.50	0.074	2.4E-04	8	9.0
P63244	Guanine nucleotide-binding protein subunit beta-2-like 1	1.50	NA	NA	2	8.8
O15511	Actin-related protein 2/3 complex subunit 5	1.49	0.188	7.0E-02	3	21.9
P30049	ATP synthase subunit delta, mitochondrial	1.49	NA	NA	2	13.7
P04233	HLA class II histocompatibility antigen gamma chain	1.49	NA	NA	2	9.8
P10606	Cytochrome c oxidase subunit 5B, mitochondrial	1.49	0.064	2.5E-03	4	30.2
Q15233	Non-POU domain-containing octamer-binding protein	1.49	0.275	1.7E-01	3	7.4
P61981	14-3-3 protein gamma	1.49	0.074	6.0E-02	3	13.8
O14561	Acyl carrier protein, mitochondrial	1.49	0.190	7.7E-02	3	15.4
P60866	40S ribosomal protein S20	1.49	0.136	4.8E-02	3	25.2
P61353	60S ribosomal protein L27	1.49	0.341	1.1E-01	3	27.9
Q15063	Periostin	1.48	0.100	3.6E-02	6	9.8
P12956	X-ray repair cross-complementing protein 6	1.48	0.055	1.1E-06	10	15.9
P07339	Cathepsin D	1.48	0.040	2.6E-06	4	9.7
O75380	NADH dehydrogenase [ubiquinone] iron-sulfur protein 6, mitochondrial	1.48	NA	NA	2	20.2
P51665	26S proteasome non-ATPase regulatory subunit 7	1.48	NA	NA	2	6.2
P26599	Polypyrimidine tract-binding protein 1	1.46	NA	NA	2	2.8
Q12905	Interleukin enhancer-binding factor 2	1.46	0.068	1.9E-04	3	9.5
P62258	14-3-3 protein epsilon	1.46	0.099	3.5E-02	8	27.5
O96000	NADH dehydrogenase [ubiquinone] 1 beta subcomplex subunit 10	1.46	NA	NA	2	12.2
P62424	60S ribosomal protein L7a	1.45	0.121	8.4E-02	4	12.0
Q9UHQ9	NADH-cytochrome b5 reductase 1	1.44	0.150	2.7E-02	7	24.3
P61247	40S ribosomal protein S3a	1.44	0.095	7.8E-03	5	18.9
P01023	Alpha-2-macroglobulin	1.44	0.509	2.5E-01	12	10.0
O43390	Heterogeneous nuclear ribonucleoprotein R	1.44	0.311	2.1E-01	4	7.0
P09622	Dihydrolipoyl dehydrogenase, mitochondrial	1.44	0.145	7.6E-02	4	8.6
P52597	Heterogeneous nuclear ribonucleoprotein F	1.44	0.277	3.6E-01	4	8.2
Q8NC56	LEM domain-containing protein 2	1.43	0.051	2.5E-04	3	5.6
Q05397	Focal adhesion kinase 1	1.43	NA	NA	2	2.3
P49792	E3 SUMO-protein ligase RanBP2	1.43	0.093	6.4E-02	4	0.7
Q13177	Serine/threonine-protein kinase PAK 2	1.43	NA	NA	2	5.9
P22626	Heterogeneous nuclear ribonucleoproteins A2/B1	1.43	0.061	2.3E-06	15	37.4
P40227	T-complex protein 1 subunit zeta	1.42	38.993	5.6E-01	3	7.5

Q9BUJ2	Heterogeneous nuclear ribonucleoprotein U-like protein 1	1.42	0.373	1.8E-01	3	5.5
P27635	60S ribosomal protein L10	1.42	NA	NA	2	9.8
O95202	LETM1 and EF-hand domain-containing protein 1, mitochondrial	1.42	0.796	1.5E-01	4	6.4
P13796	Plastin-2	1.42	NA	NA	2	3.5
P0CW22	40S ribosomal protein S17-like	1.42	0.137	1.1E-02	3	16.3
P51810	G-protein coupled receptor 143	1.42	0.152	3.5E-02	3	9.9
P51148	Ras-related protein Rab-5C	1.41	NA	NA	2	10.6
Q08211	ATP-dependent RNA helicase A	1.41	0.239	1.2E-01	8	6.9
P31689	DnaJ homolog subfamily A member 1	1.41	NA	NA	2	4.8
Q9NP81	Serine--tRNA ligase, mitochondrial	1.41	NA	NA	2	6.9
O75947	ATP synthase subunit d, mitochondrial	1.41	0.243	1.9E-01	5	24.8
Q16629	Serine/arginine-rich splicing factor 7	1.41	NA	NA	2	8.8
O15144	Actin-related protein 2/3 complex subunit 2	1.40	0.113	1.7E-02	5	13.0
P30740	Leukocyte elastase inhibitor	1.40	0.083	1.6E-01	4	17.7
P08621	U1 small nuclear ribonucleoprotein 70 kDa	1.39	NA	NA	2	4.6
Q99623	Prohibitin-2	1.39	0.049	1.8E-05	9	31.8
Q9NX40	OCIA domain-containing protein 1	1.39	NA	NA	2	8.2
P52272	Heterogeneous nuclear ribonucleoprotein M	1.39	0.037	2.4E-06	8	13.7
P29728	2'-5'-oligoadenylate synthase 2	1.39	NA	NA	2	3.8
P01860	Ig gamma-3 chain C region	1.39	0.091	1.5E-03	3	6.4
O15371	Eukaryotic translation initiation factor 3 subunit D	1.39	NA	NA	2	3.5
P61769	Beta-2-microglobulin	1.38	NA	NA	2	16.8
O60664	Perilipin-3	1.38	NA	NA	2	7.4
P30101	Protein disulfide-isomerase A3	1.38	0.050	1.7E-07	17	29.1
P62277	40S ribosomal protein S13	1.38	0.315	2.0E-02	7	42.4
O00231	26S proteasome non-ATPase regulatory subunit 11	1.38	0.092	4.2E-02	4	10.4
P49755	Transmembrane emp24 domain-containing protein 10	1.38	NA	NA	2	9.1
P49756	RNA-binding protein 25	1.37	NA	NA	2	3.9
P46777	60S ribosomal protein L5	1.37	0.244	7.4E-02	6	21.2
Q07065	Cytoskeleton-associated protein 4	1.37	0.176	2.7E-03	10	19.8
P49721	Proteasome subunit beta type-2	1.37	NA	NA	2	10.9
Q16836	Hydroxyacyl-coenzyme A dehydrogenase, mitochondrial	1.37	NA	NA	2	6.1
Q92841	Probable ATP-dependent RNA helicase DDX17	1.37	0.127	8.8E-02	5	7.5
P39019	40S ribosomal protein S19	1.36	0.160	9.7E-02	5	31.0
P36404	ADP-ribosylation factor-like protein 2	1.36	NA	NA	2	9.2
P48739	Phosphatidylinositol transfer protein beta isoform	1.36	NA	NA	2	9.2
Q02252	Methylmalonate-semialdehyde dehydrogenase [acylating], mitochondrial	1.36	0.096	1.7E-02	3	5.8
P08670	Vimentin	1.35	0.056	1.5E-08	31	61.8
P38117	Electron transfer flavoprotein subunit beta	1.35	0.095	9.8E-03	4	12.5
P62136	Serine/threonine-protein phosphatase PP1-alpha catalytic subunit	1.35	NA	NA	2	6.1
Q9UHV9	Prefoldin subunit 2	1.35	NA	NA	2	14.9
P61254	60S ribosomal protein L26	1.35	0.106	3.9E-02	3	16.6
Q03519	Antigen peptide transporter 2	1.35	NA	NA	2	2.8
O43681	ATPase ASNA1	1.34	NA	NA	2	5.5
Q15366	Poly(rC)-binding protein 2	1.34	NA	NA	2	9.9
P14854	Cytochrome c oxidase subunit 6B1	1.33	NA	NA	2	23.3
P62829	60S ribosomal protein L23	1.33	NA	NA	2	12.9
P00505	Aspartate aminotransferase, mitochondrial	1.32	0.076	3.3E-03	7	18.8
P17844	Probable ATP-dependent RNA helicase DDX5	1.32	NA	NA	2	3.4
O75489	NADH dehydrogenase [ubiquinone] iron-sulfur protein 3, mitochondrial	1.31	0.077	4.3E-03	5	19.3
P05141	ADP/ATP translocase 2	1.31	NA	NA	2	7.7
P35637	RNA-binding protein FUS	1.31	0.163	8.2E-02	4	8.9
P46778	60S ribosomal protein L21	1.31	NA	NA	2	16.9
P30048	Thioredoxin-dependent peroxide reductase, mitochondrial	1.31	0.167	6.3E-02	5	18.0
P20700	Lamin-B1	1.31	0.146	7.5E-02	9	16.2
Q07666	KH domain-containing, RNA-binding, signal transduction-associated protein 1	1.30	NA	NA	2	3.4
P07602	Prosaposin	1.30	0.179	1.9E-01	5	7.1
Q27J81	Inverted formin-2	1.30	NA	NA	2	1.8
P45880	Voltage-dependent anion-selective channel protein 2	1.30	0.094	8.6E-03	7	24.5
Q9UIJ7	GTP:AMP phosphotransferase AK3, mitochondrial	1.30	0.223	1.6E-01	5	27.3
P61158	Actin-related protein 3	1.30	0.053	2.4E-03	7	20.6
Q13283	Ras GTPase-activating protein-binding protein 1	1.30	NA	NA	2	5.8
P17858	ATP-dependent 6-phosphofructokinase, liver type	1.29	0.063	4.3E-03	5	8.7
O43678	NADH dehydrogenase [ubiquinone] 1 alpha subcomplex subunit 2	1.29	NA	NA	2	20.2
O75367	Core histone macro-H2A.1	1.29	0.165	9.2E-03	8	23.7
O00483	NADH dehydrogenase [ubiquinone] 1 alpha subcomplex subunit 4	1.29	NA	NA	2	22.2
P62753	40S ribosomal protein S6	1.28	0.138	2.2E-01	3	12.9
P62280	40S ribosomal protein S11	1.28	0.233	1.8E-01	8	34.2
P09382	Galectin-1	1.28	0.065	2.0E-03	5	43.7
P11142	Heat shock cognate 71 kDa protein	1.28	0.046	7.8E-04	9	13.8
P20810	Calpastatin	1.28	NA	NA	2	4.7
P62249	40S ribosomal protein S16	1.27	0.046	1.9E-04	3	23.3
Q15459	Splicing factor 3A subunit 1	1.27	0.557	6.9E-01	3	5.4
P13010	X-ray repair cross-complementing protein 5	1.27	0.404	5.0E-01	5	9.6
O15143	Actin-related protein 2/3 complex subunit 1B	1.27	NA	NA	2	9.4
Q07020	60S ribosomal protein L18	1.27	0.071	7.4E-03	4	25.0
P08134	Rho-related GTP-binding protein RhoC	1.26	0.039	4.5E-04	3	10.4
P56192	Methionine--tRNA ligase, cytoplasmic	1.26	NA	NA	2	3.8
Q86VP6	Cullin-associated NEDD8-dissociated protein 1	1.26	0.183	2.9E-01	4	3.7
P26373	60S ribosomal protein L13	1.26	0.169	6.0E-02	4	19.4
P30533	Alpha-2-macroglobulin receptor-associated protein	1.26	0.191	4.5E-01	3	8.1
Q14683	Structural maintenance of chromosomes protein 1A	1.25	0.269	1.6E-01	4	3.1
Q9Y3U8	60S ribosomal protein L36	1.25	0.182	4.1E-02	4	30.5
P09525	Annexin A4	1.25	0.271	2.0E-01	6	16.0
Q9P2E9	Ribosome-binding protein 1	1.25	0.405	1.3E-01	10	7.8
O75323	Protein NipSnap homolog 2	1.24	NA	NA	2	6.6
O00303	Eukaryotic translation initiation factor 3 subunit F	1.24	NA	NA	2	9.8
P02790	Hemopexin	1.24	0.111	1.3E-02	3	6.3
P49207	60S ribosomal protein L34	1.24	0.237	4.2E-01	3	20.5
P50402	Emerin	1.23	0.309	2.1E-01	5	17.7
P62269	40S ribosomal protein S18	1.23	0.172	5.5E-02	6	28.9
P27348	14-3-3 protein theta	1.22	0.117	2.2E-01	3	12.2
Q8IWB7	WD repeat and FYVE domain-containing protein 1	1.22	NA	NA	2	5.1
Q92598	Heat shock protein 105 kDa	1.21	0.186	2.5E-01	3	3.3
Q15293	Reticulocalbin-1	1.21	0.181	2.2E-01	4	10.3
Q07960	Rho GTPase-activating protein 1	1.21	NA	NA	2	4.6
P08237	ATP-dependent 6-phosphofructokinase, muscle type	1.21	0.096	2.4E-01	5	8.2
Q14980	Nuclear mitotic apparatus protein 1	1.20	0.092	2.6E-02	7	4.6
Q9P2R7	Succinyl-CoA ligase [ADP-forming] subunit beta, mitochondrial	1.20	NA	NA	2	3.7
P26368	Splicing factor U2AF 65 kDa subunit	1.20	NA	NA	2	3.8
P15586	N-acetylglucosamine-6-sulfatase	1.20	NA	NA	2	3.3
Q16762	Thiosulfate sulfurtransferase	1.20	NA	NA	2	10.8
P62917	60S ribosomal protein L8	1.19	NA	NA	2	10.5
P36873	Serine/threonine-protein phosphatase PP1-gamma catalytic subunit	1.19	NA	NA	2	5.9
P17980	26S protease regulatory subunit 6A	1.19	0.052	6.9E-02	3	7.5
P62318	Small nuclear ribonucleoprotein Sm D3	1.19	0.063	4.2E-02	3	31.7
P54727	UV excision repair protein RAD23 homolog B	1.19	0.235	3.6E-01	5	10.8
P61421	V-type proton ATPase subunit d 1	1.18	0.142	1.3E-01	3	7.7
P54136	Arginine--tRNA ligase, cytoplasmic	1.18	0.236	2.1E-01	5	8.2
Q86U42	Polyadenylate-binding protein 2	1.18	NA	NA	2	5.9
P14314	Glucosidase 2 subunit beta	1.18	0.278	2.1E-01	10	12.3
P51572	B-cell receptor-associated protein 31	1.18	0.160	3.0E-01	7	25.6
P50914	60S ribosomal protein L14	1.17	NA	NA	2	10.7
P0CG29	Glutathione S-transferase theta-2	1.17	NA	NA	2	7.8
P48047	ATP synthase subunit O, mitochondrial	1.17	0.160	1.7E-01	6	34.3

P30519	Heme oxygenase 2	1.17	NA	NA	2	7.0
P43686	26S protease regulatory subunit 6B	1.17	NA	NA	2	5.0
Q53H12	Acylglycerol kinase, mitochondrial	1.17	NA	NA	2	5.0
P62987	Ubiquitin-60S ribosomal protein L40	1.16	0.179	2.4E-02	7	46.1
P15880	40S ribosomal protein S2	1.16	0.613	3.2E-01	6	21.8
P55265	Double-stranded RNA-specific adenosine deaminase	1.16	NA	NA	2	1.9
Q9Y2W1	Thyroid hormone receptor-associated protein 3	1.16	0.394	7.0E-01	4	4.4
P02766	Transthyretin	1.16	NA	NA	2	12.9
Q12874	Splicing factor 3A subunit 3	1.16	0.485	5.7E-01	3	5.8
P50454	Serpin H1	1.15	0.082	4.5E-01	3	8.1
Q5SSJ5	Heterochromatin protein 1-binding protein 3	1.15	0.157	3.8E-01	3	6.7
P13073	Cytochrome c oxidase subunit 4 isoform 1, mitochondrial	1.15	0.119	2.1E-01	3	19.5
Q5JRX3	Presequence protease, mitochondrial	1.15	NA	NA	2	3.2
P05198	Eukaryotic translation initiation factor 2 subunit 1	1.15	0.587	8.2E-01	3	9.5
P30153	Serine/threonine-protein phosphatase 2A 65 kDa regulatory subunit A alpha isoform	1.15	NA	NA	2	4.8
P01859	Ig gamma-2 chain C region	1.14	NA	NA	2	6.7
Q15393	Splicing factor 3B subunit 3	1.14	NA	NA	2	2.1
Q9NTZ6	RNA-binding protein 12	1.14	NA	NA	2	2.8
Q13310	Polyadenylate-binding protein 4	1.14	NA	NA	2	4.7
Q99442	Translocation protein SEC62	1.14	0.172	3.2E-01	3	7.3
Q9Y4I1	Unconventional myosin-Va	1.13	0.502	5.2E-01	3	1.6
O95302	Peptidyl-prolyl cis-trans isomerase FKBP9	1.13	0.620	4.0E-01	3	5.3
P59998	Actin-related protein 2/3 complex subunit 4	1.13	0.205	6.8E-01	3	16.1
P01876	Ig alpha-1 chain C region	1.13	0.081	1.1E-01	3	10.8
P19971	Thymidine phosphorylase	1.13	0.301	6.0E-01	3	7.9
Q13347	Eukaryotic translation initiation factor 3 subunit I	1.13	NA	NA	2	6.8
P54652	Heat shock-related 70 kDa protein 2	1.13	0.126	4.3E-01	3	5.6
P13667	Protein disulfide-isomerase A4	1.13	0.190	3.0E-01	4	6.4
P14866	Heterogeneous nuclear ribonucleoprotein L	1.13	0.046	8.4E-03	5	10.0
P36578	60S ribosomal protein L4	1.13	0.145	4.2E-01	4	10.1
O43598	2'-deoxynucleoside 5'-phosphate N-hydrolase 1	1.13	NA	NA	2	19.0
P11940	Polyadenylate-binding protein 1	1.12	0.112	6.9E-02	6	12.3
Q9Y262	Eukaryotic translation initiation factor 3 subunit L	1.12	NA	NA	2	3.4
O60568	Procollagen-lysine,2-oxoglutarate 5-dioxygenase 3	1.11	NA	NA	2	4.2
P46779	60S ribosomal protein L28	1.11	NA	NA	2	13.1
Q02878	60S ribosomal protein L6	1.11	0.098	3.1E-01	6	20.8
Q9BZQ8	Protein Niban	1.11	0.199	5.3E-01	5	5.8
P56556	NADH dehydrogenase [ubiquinone] 1 alpha subcomplex subunit 6	1.11	NA	NA	2	12.3
Q5JTV8	Torsin-1A-interacting protein 1	1.11	NA	NA	2	4.5
P23396	40S ribosomal protein S3	1.10	0.141	9.2E-02	8	30.0
Q02318	Sterol 26-hydroxylase, mitochondrial	1.10	NA	NA	2	5.6
P32969	60S ribosomal protein L9	1.10	NA	NA	2	5.7
P49593	Protein phosphatase 1F	1.10	NA	NA	2	7.5
P61160	Actin-related protein 2	1.09	0.080	2.5E-01	3	9.9
P13674	Prolyl 4-hydroxylase subunit alpha-1	1.09	NA	NA	2	5.8
P48681	Nestin	1.08	0.957	7.7E-01	3	2.2
P82909	28S ribosomal protein S36, mitochondrial	1.08	0.080	4.0E-01	3	36.9
P05388	60S acidic ribosomal protein P0	1.07	NA	NA	2	6.6
P42765	3-ketoacyl-CoA thiolase, mitochondrial	1.07	0.334	6.8E-01	5	14.4
Q9Y230	RuvB-like 2	1.07	NA	NA	2	5.0
P51159	Ras-related protein Rab-27A	1.06	NA	NA	2	10.4
Q02543	60S ribosomal protein L18a	1.06	NA	NA	2	10.2
P11021	78 kDa glucose-regulated protein	1.06	0.155	2.6E-01	18	24.0
P46782	40S ribosomal protein S5	1.06	0.236	6.6E-01	3	8.8
Q12913	Receptor-type tyrosine-protein phosphatase eta	1.06	NA	NA	2	2.2
P20674	Cytochrome c oxidase subunit 5A, mitochondrial	1.05	NA	NA	2	10.7
P54709	Sodium/potassium-transporting ATPase subunit beta-3	1.05	1.663	8.9E-01	3	13.3
Q13596	Sorting nexin-1	1.05	0.535	7.2E-01	3	7.1
P78371	T-complex protein 1 subunit beta	1.05	4.358	7.8E-01	6	13.6
P06737	Glycogen phosphorylase, liver form	1.05	0.475	7.4E-01	4	5.1
P11586	C-1-tetrahydrofolate synthase, cytoplasmic	1.05	0.242	4.5E-01	10	11.4
Q00325	Phosphate carrier protein, mitochondrial	1.04	0.266	5.1E-01	6	16.0
P10644	cAMP-dependent protein kinase type I-alpha regulatory subunit	1.04	0.605	6.7E-01	3	7.6
P22307	Non-specific lipid-transfer protein	1.04	NA	NA	2	3.1
O00410	Importin-5	1.04	NA	NA	2	1.5
P02545	Prelamin-A/C	1.04	0.349	3.4E-01	40	52.7
P43490	Nicotinamide phosphoribosyltransferase	1.04	0.612	8.3E-01	3	5.1
P11177	Pyruvate dehydrogenase E1 component subunit beta, mitochondrial	1.03	NA	NA	2	4.2
P21912	Succinate dehydrogenase [ubiquinone] iron-sulfur subunit, mitochondrial	1.03	0.555	7.5E-01	5	17.5
O95292	Vesicle-associated membrane protein-associated protein B/C	1.03	NA	NA	2	11.1
Q16891	Mitochondrial inner membrane protein	1.02	0.225	6.3E-01	10	16.5
P60763	Ras-related C3 botulinum toxin substrate 3	1.02	1.037	8.0E-01	3	14.1
P14927	Cytochrome b-c1 complex subunit 7	1.02	0.591	8.5E-01	3	28.8
P07237	Protein disulfide-isomerase	1.02	0.311	8.9E-01	18	30.7
O75964	ATP synthase subunit g, mitochondrial	1.02	NA	NA	2	27.2
P62899	60S ribosomal protein L31	1.02	NA	NA	2	13.6
P09496	Clathrin light chain A	1.01	0.020	5.8E-01	4	12.9
P09543	2',3'-cyclic-nucleotide 3'-phosphodiesterase	1.01	3.544	8.2E-01	8	18.3
P42766	60S ribosomal protein L35	1.01	NA	NA	2	15.4
Q96TC7	Regulator of microtubule dynamics protein 3	1.01	NA	NA	2	4.0
P84103	Serine/arginine-rich splicing factor 3	1.01	1.220	9.5E-01	3	20.7
Q15075	Early endosome antigen 1	1.01	NA	NA	2	1.3
O14980	Exportin-1	1.01	NA	NA	2	2.2
Q9NSD9	Phenylalanine--tRNA ligase beta subunit	1.01	0.678	9.6E-01	3	4.9
Q15717	ELAV-like protein 1	1.00	0.227	9.8E-01	3	15.3
P18124	60S ribosomal protein L7	1.00	0.451	9.7E-01	5	16.9
Q8N5K1	CDGSH iron-sulfur domain-containing protein 2	1.00	0.004	4.1E-01	3	15.6
P20645	Cation-dependent mannose-6-phosphate receptor	0.99	NA	NA	2	10.5
O75832	26S proteasome non-ATPase regulatory subunit 10	0.99	NA	NA	2	12.8
O75306	NADH dehydrogenase [ubiquinone] iron-sulfur protein 2, mitochondrial	0.99	0.413	9.3E-01	5	12.5
Q9NTJ5	Phosphatidylinositide phosphatase SAC1	0.99	0.639	9.0E-01	3	5.8
P39023	60S ribosomal protein L3	0.99	NA	NA	2	6.9
P62714	Serine/threonine-protein phosphatase 2A catalytic subunit beta isoform	0.99	NA	NA	2	6.1
P26640	Valine--tRNA ligase	0.99	0.269	9.6E-01	3	2.8
P14868	Aspartate--tRNA ligase, cytoplasmic	0.99	2.023	9.3E-01	6	13.8
Q96AE4	Far upstream element-binding protein 1	0.98	0.207	8.5E-01	4	6.1
P06576	ATP synthase subunit beta, mitochondrial	0.98	0.458	5.9E-01	13	33.6
P62081	40S ribosomal protein S7	0.98	NA	NA	2	8.8
Q9BS26	Endoplasmic reticulum resident protein 44	0.98	0.233	8.3E-01	4	10.1
P04440	HLA class II histocompatibility antigen, DP beta 1 chain	0.97	NA	NA	2	7.8
Q9BRX8	Redox-regulatory protein FAM213A	0.97	NA	NA	2	9.2
P62701	40S ribosomal protein S4, X isoform	0.97	0.098	7.6E-01	6	20.2
Q96C86	m7GpppX diphosphatase	0.97	NA	NA	2	8.3
P48643	T-complex protein 1 subunit epsilon	0.96	1.214	8.1E-01	9	17.6
Q9UKM9	RNA-binding protein Raly	0.96	NA	NA	2	6.2
P00403	Cytochrome c oxidase subunit 2	0.96	NA	NA	2	7.5
P46940	Ras GTPase-activating-like protein IQGAP1	0.96	0.148	7.9E-01	4	3.1
Q99873	Protein arginine N-methyltransferase 1	0.96	NA	NA	2	6.4
P49748	Very long-chain specific acyl-CoA dehydrogenase, mitochondrial	0.95	0.458	5.2E-01	7	11.5
Q9GZY8	Mitochondrial fission factor	0.95	0.106	4.4E-01	3	8.5
P24539	ATP synthase F(0) complex subunit B1, mitochondrial	0.95	NA	NA	2	9.0
Q13151	Heterogeneous nuclear ribonucleoprotein A0	0.95	54.417	8.2E-01	3	7.5
O14773	Tripeptidyl-peptidase 1	0.94	0.162	5.4E-01	3	6.2
P36955	Pigment epithelium-derived factor	0.94	2.698	7.6E-01	3	8.1
Q9BR76	Coronin-1B	0.94	NA	NA	2	3.9

Q6P2Q9	Pre-mRNA-processing-splicing factor 8	0.94	NA	NA	2	0.8
O60313	Dynamin-like 120 kDa protein, mitochondrial	0.94	0.175	6.6E-01	6	7.4
Q13200	26S proteasome non-ATPase regulatory subunit 2	0.94	0.197	5.6E-01	4	4.2
Q8IZP0	Abl interactor 1	0.94	NA	NA	2	4.9
P14625	Endoplasmin	0.94	0.101	1.3E-01	16	19.2
P55072	Transitional endoplasmic reticulum ATPase	0.93	0.114	3.1E-01	13	17.5
O75521	Enoyl-CoA delta isomerase 2, mitochondrial	0.93	NA	NA	2	5.6
P55884	Eukaryotic translation initiation factor 3 subunit B	0.93	0.297	5.5E-01	3	5.3
P61163	Alpha-centractin	0.93	NA	NA	2	7.4
P63208	S-phase kinase-associated protein 1	0.92	NA	NA	2	12.3
P09669	Cytochrome c oxidase subunit 6C	0.92	NA	NA	2	20.0
Q15365	Poly(rC)-binding protein 1	0.92	NA	NA	2	7.6
O95168	NADH dehydrogenase [ubiquinone] 1 beta subcomplex subunit 4	0.92	NA	NA	2	17.1
O00203	AP-3 complex subunit beta-1	0.91	NA	NA	2	2.7
P62805	Histone H4	0.91	0.085	7.4E-03	8	52.4
P07858	Cathepsin B	0.91	1.480	5.0E-01	4	13.0
P67936	Tropomyosin alpha-4 chain	0.91	0.272	5.5E-01	9	28.6
P46781	40S ribosomal protein S9	0.91	0.090	1.5E-01	5	18.0
Q9Y3Z3	Deoxynucleoside triphosphate triphosphohydrolase SAMHD1	0.91	0.040	2.1E-01	4	7.2
P07203	Glutathione peroxidase 1	0.90	NA	NA	2	5.9
Q9UHD8	Septin-9	0.90	0.164	5.0E-01	3	5.8
P28065	Proteasome subunit beta type-9	0.90	NA	NA	2	10.0
O43242	26S proteasome non-ATPase regulatory subunit 3	0.89	NA	NA	2	3.0
O75396	Vesicle-trafficking protein SEC22b	0.89	0.502	5.6E-01	5	28.8
Q15691	Microtubule-associated protein RP/EB family member 1	0.89	0.613	4.0E-01	4	9.7
P61225	Ras-related protein Rap-2b	0.89	0.145	1.6E-01	3	16.4
P78527	DNA-dependent protein kinase catalytic subunit	0.89	0.361	3.3E-01	7	1.6
P53992	Protein transport protein Sec24C	0.89	NA	NA	2	2.0
Q13162	Peroxiredoxin-4	0.88	NA	NA	2	8.9
O43707	Alpha-actinin-4	0.88	0.044	7.6E-03	15	19.2
P35606	Coatomer subunit beta'	0.88	NA	NA	2	2.1
Q00765	Receptor expression-enhancing protein 5	0.88	0.478	5.4E-01	3	10.6
P0C0S5	Histone H2A.Z	0.88	NA	NA	2	18.8
P05107	Integrin beta-2	0.88	0.085	3.2E-01	4	6.0
P49368	T-complex protein 1 subunit gamma	0.87	0.059	7.8E-03	6	11.4
P36543	V-type proton ATPase subunit E 1	0.87	0.182	4.3E-01	4	18.6
Q93050	V-type proton ATPase 116 kDa subunit a isoform 1	0.87	NA	NA	2	3.1
Q7KZF4	Staphylococcal nuclease domain-containing protein 1	0.87	NA	NA	2	2.7
Q15149	Plectin	0.87	0.048	3.7E-06	85	18.9
Q9UBI6	Guanine nucleotide-binding protein G(I)/G(S)/G(O) subunit gamma-12	0.86	NA	NA	2	34.7
P25705	ATP synthase subunit alpha, mitochondrial	0.86	0.080	1.6E-03	15	29.7
P19367	Hexokinase-1	0.86	0.595	2.9E-01	4	4.0
P53618	Coatomer subunit beta	0.86	NA	NA	2	2.1
Q15084	Protein disulfide-isomerase A6	0.86	NA	NA	2	6.8
P54920	Alpha-soluble NSF attachment protein	0.85	0.189	2.4E-01	4	14.2
P43307	Translocon-associated protein subunit alpha	0.85	NA	NA	2	6.6
Q8IZ83	Aldehyde dehydrogenase family 16 member A1	0.85	NA	NA	2	3.0
P50991	T-complex protein 1 subunit delta	0.85	0.205	3.4E-01	6	12.2
Q8N1G4	Leucine-rich repeat-containing protein 47	0.84	0.099	4.1E-01	3	6.7
P04843	Dolichyl-diphosphooligosaccharide--protein glycosyltransferase subunit 1	0.84	0.070	2.0E-02	9	16.3
P67870	Casein kinase II subunit beta	0.84	NA	NA	2	10.2
P62244	40S ribosomal protein S15a	0.84	0.359	3.5E-01	3	18.5
P40429	60S ribosomal protein L13a	0.84	NA	NA	2	9.9
P27824	Calnexin	0.84	0.294	1.9E-01	11	20.3
P35221	Catenin alpha-1	0.83	0.178	1.9E-01	7	7.7
Q99816	Tumor susceptibility gene 101 protein	0.83	NA	NA	2	5.4
Q16695	Histone H3.1t	0.83	0.037	1.8E-04	4	21.3
Q99584	Protein S100-A13	0.83	0.142	1.1E-01	4	30.6
P08559	Pyruvate dehydrogenase E1 component subunit alpha, somatic form, mitochondrial	0.82	NA	NA	2	5.4
Q05682	Caldesmon	0.82	0.348	4.3E-01	6	11.0
P08174	Complement decay-accelerating factor	0.82	NA	NA	2	4.5
P53621	Coatomer subunit alpha	0.82	0.316	3.7E-01	4	3.8
Q6NUK1	Calcium-binding mitochondrial carrier protein SCaMC-1	0.82	NA	NA	2	3.4
P50990	T-complex protein 1 subunit theta	0.82	0.038	6.8E-06	11	18.8
P55060	Exportin-2	0.81	NA	NA	2	1.6
Q9BX66	Sorbin and SH3 domain-containing protein 1	0.81	NA	NA	2	1.9
Q13561	Dynactin subunit 2	0.81	0.209	1.3E-01	4	8.2
Q7Z6Z7	E3 ubiquitin-protein ligase HUWE1	0.81	NA	NA	2	0.5
Q93009	Ubiquitin carboxyl-terminal hydrolase 7	0.81	NA	NA	2	2.1
Q99832	T-complex protein 1 subunit eta	0.80	0.030	2.6E-03	4	8.1
P37837	Transaldolase	0.80	1.042	6.1E-01	3	6.8
Q92522	Histone H1x	0.80	NA	NA	2	11.7
P17931	Galectin-3	0.79	0.212	1.3E-01	6	28.4
P30508	HLA class I histocompatibility antigen, Cw-12 alpha chain	0.79	NA	NA	2	7.1
O94925	Glutaminase kidney isoform, mitochondrial	0.79	NA	NA	2	3.7
P02774	Vitamin D-binding protein	0.79	0.242	3.2E-01	3	5.7
P10515	Dihydrolipoyllysine-residue acetyltransferase component of pyruvate dehydrogenase complex, mitochondrial	0.79	0.364	4.1E-01	3	4.0
Q969X5	Endoplasmic reticulum-Golgi intermediate compartment protein 1	0.78	0.024	5.3E-02	3	11.0
Q53GG5	PDZ and LIM domain protein 3	0.78	NA	NA	2	9.1
Q9UMX5	Neudesin	0.78	NA	NA	2	16.9
P61026	Ras-related protein Rab-10	0.77	NA	NA	2	9.0
Q8WUM4	Programmed cell death 6-interacting protein	0.77	0.060	8.4E-03	6	5.4
P06899	Histone H2B type 1-J	0.76	NA	NA	2	7.9
Q86UP2	Kinectin	0.76	0.164	2.6E-02	7	6.4
Q9BTV4	Transmembrane protein 43	0.76	NA	NA	2	6.3
Q14697	Neutral alpha-glucosidase AB	0.76	0.084	6.7E-03	11	11.5
P24752	Acetyl-CoA acetyltransferase, mitochondrial	0.76	0.828	2.4E-01	4	12.2
P33176	Kinesin-1 heavy chain	0.75	NA	NA	2	2.4
P26038	Moesin	0.74	0.072	9.2E-04	6	9.0
Q03252	Lamin-B2	0.74	0.033	4.7E-07	19	32.0
P31943	Heterogeneous nuclear ribonucleoprotein H	0.73	0.135	9.3E-02	3	8.0
Q12797	Aspartyl/asparaginyl beta-hydroxylase	0.73	0.184	1.4E-01	4	4.7
P07305	Histone H1.0	0.73	0.194	4.9E-01	3	16.0
P35222	Catenin beta-1	0.73	0.097	4.1E-02	6	9.1
P37108	Signal recognition particle 14 kDa protein	0.73	0.279	3.0E-01	3	22.8
O60716	Catenin delta-1	0.72	NA	NA	2	2.0
P22695	Cytochrome b-c1 complex subunit 2, mitochondrial	0.72	0.045	6.4E-04	4	12.1
P16615	Sarcoplasmic/endoplasmic reticulum calcium ATPase 2	0.71	0.073	2.1E-04	8	8.5
Q9Y240	C-type lectin domain family 11 member A	0.71	0.205	5.1E-02	4	14.2
Q14108	Lysosome membrane protein 2	0.71	0.058	8.2E-03	3	6.3
P13987	CD59 glycoprotein	0.71	NA	NA	2	15.6
O94979	Protein transport protein Sec31A	0.71	0.169	5.4E-02	6	5.7
Q7L5N1	COP9 signalosome complex subunit 6	0.70	NA	NA	2	6.7
P17987	T-complex protein 1 subunit alpha	0.70	0.075	1.1E-02	5	9.4
P48735	Isocitrate dehydrogenase [NADP], mitochondrial	0.70	0.057	3.9E-03	5	12.4
P51812	Ribosomal protein S6 kinase alpha-3	0.69	NA	NA	2	2.3
O14950	Myosin regulatory light chain 12B	0.69	NA	NA	2	12.2
P47756	F-actin-capping protein subunit beta	0.69	0.141	7.0E-02	4	14.4
P63261	Actin, cytoplasmic 2	0.69	0.041	8.2E-08	6	24.8
P23634	Plasma membrane calcium-transporting ATPase 4	0.69	0.073	1.0E-02	4	4.9
P06396	Gelsolin	0.68	0.106	3.3E-03	10	12.5
P26196	Probable ATP-dependent RNA helicase DDX6	0.68	NA	NA	2	4.1
P36957	Dihydrolipoyllysine-residue succinyltransferase component of 2-oxoglutarate dehydrogenase complex, mitochondrial	0.68	0.067	9.6E-04	4	9.9
O00264	Membrane-associated progesterone receptor component 1	0.68	0.098	2.6E-03	4	15.9
P21281	V-type proton ATPase subunit B, brain isoform	0.68	0.076	1.1E-02	3	7.4

O00571	ATP-dependent RNA helicase DDX3X	0.68	0.452	2.8E-01	3	5.7
P04844	Dolichyl-diphosphooligosaccharide--protein glycosyltransferase subunit 2	0.68	0.050	7.4E-03	3	5.4
P00387	NADH-cytochrome b5 reductase 3	0.68	0.124	1.1E-02	4	14.6
P00390	Glutathione reductase, mitochondrial	0.67	NA	NA	2	6.3
Q8IX12	Cell division cycle and apoptosis regulator protein 1	0.67	NA	NA	2	2.3
Q16698	2,4-dienoyl-CoA reductase, mitochondrial	0.67	NA	NA	2	8.7
P43121	Cell surface glycoprotein MUC18	0.67	0.096	1.2E-01	4	6.8
P38606	V-type proton ATPase catalytic subunit A	0.67	NA	NA	2	3.2
P30837	Aldehyde dehydrogenase X, mitochondrial	0.67	0.157	3.6E-02	5	14.1
P13861	cAMP-dependent protein kinase type II-alpha regulatory subunit	0.66	0.224	2.2E-01	4	13.9
Q9H223	EH domain-containing protein 4	0.66	NA	NA	2	3.0
O75746	Calcium-binding mitochondrial carrier protein Aralar1	0.66	0.084	1.3E-02	4	5.8
P39656	Dolichyl-diphosphooligosaccharide--protein glycosyltransferase 48 kDa subunit	0.65	0.108	1.3E-02	4	8.3
P02511	Alpha-crystallin B chain	0.65	0.070	3.2E-03	6	35.4
P00367	Glutamate dehydrogenase 1, mitochondrial	0.64	0.065	3.4E-04	6	12.0
P05023	Sodium/potassium-transporting ATPase subunit alpha-1	0.64	0.107	3.6E-03	5	5.9
O75131	Copine-3	0.64	0.106	2.7E-03	4	7.6
P09497	Clathrin light chain B	0.64	0.116	1.2E-02	4	15.3
P01903	HLA class II histocompatibility antigen, DR alpha chain	0.64	0.063	7.5E-02	3	14.6
P07437	Tubulin beta chain	0.64	NA	NA	2	6.5
P04179	Superoxide dismutase [Mn], mitochondrial	0.63	0.250	2.8E-01	6	23.4
Q9Y6N5	Sulfide:quinone oxidoreductase, mitochondrial	0.63	0.564	4.7E-01	3	6.7
P47985	Cytochrome b-c1 complex subunit Rieske, mitochondrial	0.62	NA	NA	2	8.0
P40763	Signal transducer and activator of transcription 3	0.62	NA	NA	2	3.4
Q14558	Phosphoribosyl pyrophosphate synthase-associated protein 1	0.62	NA	NA	2	8.4
P43304	Glycerol-3-phosphate dehydrogenase, mitochondrial	0.61	NA	NA	2	3.0
P07099	Epoxide hydrolase 1	0.61	0.060	1.4E-05	8	14.5
Q6DD88	Atlastin-3	0.61	0.163	7.2E-02	4	7.2
P35580	Myosin-10	0.60	0.094	9.8E-04	13	7.5
P31930	Cytochrome b-c1 complex subunit 1, mitochondrial	0.60	NA	NA	2	4.6
P08571	Monocyte differentiation antigen CD14	0.60	NA	NA	2	5.3
P02647	Apolipoprotein A-I	0.59	0.076	1.6E-05	5	18.0
Q08722	Leukocyte surface antigen CD47	0.59	NA	NA	2	5.9
O43175	D-3-phosphoglycerate dehydrogenase	0.59	NA	NA	2	5.6
P11413	Glucose-6-phosphate 1-dehydrogenase	0.59	0.092	4.9E-02	3	5.4
O75369	Filamin-B	0.59	0.034	3.3E-08	11	5.6
Q16775	Hydroxyacylglutathione hydrolase, mitochondrial	0.58	NA	NA	2	6.5
Q9Y3I0	tRNA-splicing ligase RtcB homolog	0.58	0.061	1.5E-02	3	6.7
P50570	Dynamin-2	0.58	NA	NA	2	2.3
Q92973	Transportin-1	0.57	0.111	6.9E-02	3	4.5
P01871	Ig mu chain C region	0.57	0.046	1.9E-05	10	24.6
P09417	Dihydropteridine reductase	0.57	NA	NA	2	12.3
P01009	Alpha-1-antitrypsin	0.57	0.094	3.2E-04	7	17.2
Q92599	Septin-8	0.56	NA	NA	2	6.0
Q9HDC9	Adipocyte plasma membrane-associated protein	0.56	0.123	9.1E-03	4	12.3
Q14956	Transmembrane glycoprotein NMB	0.55	0.449	1.3E-01	4	8.9
Q00610	Clathrin heavy chain 1	0.55	0.053	3.0E-08	18	12.4
Q09666	Neuroblast differentiation-associated protein AHNAK	0.55	0.036	3.1E-11	67	9.8
Q14204	Cytoplasmic dynein 1 heavy chain 1	0.55	0.038	1.9E-13	21	4.4
Q53GQ0	Estradiol 17-beta-dehydrogenase 12	0.54	NA	NA	2	8.7
Q9UHG3	Prenylcysteine oxidase 1	0.54	NA	NA	2	4.6
P05091	Aldehyde dehydrogenase, mitochondrial	0.53	0.287	1.6E-01	3	5.0
Q02218	2-oxoglutarate dehydrogenase, mitochondrial	0.52	0.099	6.4E-04	5	5.5
Q14203	Dynactin subunit 1	0.52	0.466	2.3E-01	4	3.9
Q9ULA0	Aspartyl aminopeptidase	0.52	NA	NA	2	6.1
P04217	Alpha-1B-glycoprotein	0.51	NA	NA	2	4.8
P32004	Neural cell adhesion molecule L1	0.51	0.118	9.3E-03	5	4.5
P0C0L5	Complement C4-B	0.51	NA	NA	2	1.9
P02652	Apolipoprotein A-II	0.51	NA	NA	2	17.0
Q9BSD7	Cancer-related nucleoside-triphosphatase	0.51	NA	NA	2	8.9
P68371	Tubulin beta-4B chain	0.50	0.039	2.1E-09	3	9.9
Q15019	Septin-2	0.49	0.083	2.1E-03	4	13.9
Q16181	Septin-7	0.49	0.051	1.3E-06	5	11.7
P32119	Peroxiredoxin-2	0.48	0.068	7.7E-06	6	31.3
P04040	Catalase	0.48	0.047	1.2E-02	3	6.3
P17612	cAMP-dependent protein kinase catalytic subunit alpha	0.48	NA	NA	2	4.3
Q9Y4L1	Hypoxia up-regulated protein 1	0.48	NA	NA	2	2.5
O75955	Flotillin-1	0.48	0.140	7.5E-02	3	8.4
P17643	5,6-dihydroxyindole-2-carboxylic acid oxidase	0.48	NA	NA	2	3.7
Q9HBL0	Tensin-1	0.48	0.437	2.8E-01	3	3.2
Q14192	Four and a half LIM domains protein 2	0.47	NA	NA	2	7.9
P08133	Annexin A6	0.47	0.028	0.0E+00	32	48.3
O00159	Unconventional myosin-Ic	0.47	0.075	1.7E-06	11	10.1
P05362	Intercellular adhesion molecule 1	0.46	0.120	3.7E-02	3	7.9
P50995	Annexin A11	0.46	0.085	1.1E-04	6	12.1
P21589	5'-nucleotidase	0.46	0.486	4.6E-01	3	7.8
Q7Z406	Myosin-14	0.45	0.384	1.5E-01	4	2.6
P09493	Tropomyosin alpha-1 chain	0.45	0.216	1.6E-03	5	14.8
Q29974	HLA class II histocompatibility antigen, DRB1-16 beta chain	0.44	NA	NA	2	6.4
O95865	N(G),N(G)-dimethylarginine dimethylaminohydrolase 2	0.44	NA	NA	2	8.4
P60660	Myosin light polypeptide 6	0.43	0.041	0.0E+00	9	58.3
P12814	Alpha-actinin-1	0.43	0.044	7.3E-07	11	13.8
Q969P0	Immunoglobulin superfamily member 8	0.43	NA	NA	2	4.6
P18206	Vinculin	0.42	0.054	6.0E-10	14	14.2
P40939	Trifunctional enzyme subunit alpha, mitochondrial	0.42	0.131	2.8E-02	7	10.2
Q13557	Calcium/calmodulin-dependent protein kinase type II subunit delta	0.42	NA	NA	2	4.2
P06727	Apolipoprotein A-IV	0.42	0.106	8.1E-06	9	19.7
O60645	Exocyst complex component 3	0.41	NA	NA	2	3.4
P29992	Guanine nucleotide-binding protein subunit alpha-11	0.41	NA	NA	2	4.7
Q14764	Major vault protein	0.41	0.087	1.6E-04	5	5.9
O94832	Unconventional myosin-Id	0.40	0.172	7.5E-03	4	4.2
Q10588	ADP-ribosyl cyclase/cyclic ADP-ribose hydrolase 2	0.40	NA	NA	2	5.7
O94905	Erlin-2	0.40	0.048	1.1E-07	5	12.1
Q93052	Lipoma-preferred partner	0.40	NA	NA	2	3.4
P62873	Guanine nucleotide-binding protein G(I)/G(S)/G(T) subunit beta-1	0.39	0.108	2.0E-01	3	10.0
Q9NVD7	Alpha-parvin	0.39	NA	NA	2	4.3
Q16363	Laminin subunit alpha-4	0.38	0.381	7.4E-02	4	2.5
Q9NQC3	Reticulon-4	0.37	NA	NA	2	2.3
Q9NZN4	EH domain-containing protein 2	0.37	NA	NA	2	3.5
P00352	Retinal dehydrogenase 1	0.35	NA	NA	2	4.6
Q15582	Transforming growth factor-beta-induced protein ig-h3	0.33	NA	NA	2	2.8
Q86VB7	Scavenger receptor cysteine-rich type 1 protein M130	0.32	NA	NA	2	1.6
P02452	Collagen alpha-1(I) chain	0.31	NA	NA	2	1.6
P13671	Complement component C6	0.31	NA	NA	2	2.7
P05787	Keratin, type II cytoskeletal 8	0.30	NA	NA	2	3.9
P04216	Thy-1 membrane glycoprotein	0.30	NA	NA	2	15.5
Q9BSJ8	Extended synaptotagmin-1	0.30	NA	NA	2	1.8
P22105	Tenascin-X	0.30	0.102	9.5E-02	4	1.1
P46939	Utrophin	0.29	NA	NA	2	0.8
P35611	Alpha-adducin	0.28	0.108	5.1E-02	3	5.8
P22413	Ectonucleotide pyrophosphatase/phosphodiesterase family member 1	0.27	NA	NA	2	2.3
P61626	Lysozyme C	0.27	NA	NA	2	12.8
P16157	Ankyrin-1	0.27	0.342	5.9E-02	3	2.2
P07358	Complement component C8 beta chain	0.25	NA	NA	2	2.0
O94911	ATP-binding cassette sub-family A member 8	0.25	NA	NA	2	1.2

Q9BS40	Latexin	0.25	0.224	1.2E-01	3	16.7
O94919	Endonuclease domain-containing 1 protein	0.25	NA	NA	2	6.2
Q08431	Lactadherin	0.24	NA	NA	2	5.4
P07197	Neurofilament medium polypeptide	0.24	NA	NA	2	2.4
Q01995	Transgelin	0.22	0.208	8.7E-02	4	19.4
P07360	Complement component C8 gamma chain	0.20	NA	NA	2	15.8
P04196	Histidine-rich glycoprotein	0.19	NA	NA	2	3.6
Q9BUF5	Tubulin beta-6 chain	0.18	NA	NA	2	4.0
P12277	Creatine kinase B-type	0.16	NA	NA	2	4.7
Q9BXN1	Asporin	0.16	NA	NA	2	2.9
P24844	Myosin regulatory light polypeptide 9	0.15	NA	NA	2	12.2
O14495	Lipid phosphate phosphohydrolase 3	0.15	NA	NA	2	7.1
P10745	Retinol-binding protein 3	0.14	0.517	6.4E-02	3	2.4
Q12805	EGF-containing fibulin-like extracellular matrix protein 1	0.13	NA	NA	2	3.9
P58166	Inhibin beta E chain	0.12	NA	NA	2	6.6
P35243	Recoverin	0.12	NA	NA	2	10.5
Q6NUI6	Chondroadherin-like protein	0.11	NA	NA	2	3.0
P05186	Alkaline phosphatase, tissue-nonspecific isozyme	0.09	0.373	2.7E-01	3	5.5
Q2UY09	Collagen alpha-1(XXVIII) chain	0.08	NA	NA	2	1.4
P08123	Collagen alpha-2(I) chain	0.03	NA	NA	2	2.4
Q9Y490	Talin-1	0.38	0.038	0.0E+00	22	10.5
P00739	Haptoglobin-related protein	0.38	0.082	3.2E-02	5	14.7
Q9BQE3	Tubulin alpha-1C chain	0.38	0.062	6.1E-13	11	25.4
P00747	Plasminogen	0.37	0.229	2.0E-02	4	6.8
Q9NZM1	Myoferlin	0.36	0.151	4.7E-05	9	5.4
P35579	Myosin-9	0.36	0.046	1.8E-15	54	27.7
Q14254	Flotillin-2	0.36	0.071	2.6E-02	3	6.8
P26447	Protein S100-A4	0.36	0.109	1.1E-03	3	27.7
P04899	Guanine nucleotide-binding protein G(i) subunit alpha-2	0.35	0.174	1.9E-04	4	13.8
Q6NZI2	Polymerase I and transcript release factor	0.34	0.130	1.9E-03	6	17.7
P05556	Integrin beta-1	0.34	0.036	5.5E-11	6	8.0
Q07954	Prolow-density lipoprotein receptor-related protein 1	0.34	0.161	4.4E-03	7	2.1
P55084	Trifunctional enzyme subunit beta, mitochondrial	0.33	0.089	5.4E-05	4	6.8
P02549	Spectrin alpha chain, erythrocytic 1	0.33	0.223	7.7E-03	5	3.0
P00450	Ceruloplasmin	0.28	0.065	3.2E-13	11	12.1
Q9BXM0	Periaxin	0.28	0.199	9.9E-04	6	2.9
Q63ZY3	KN motif and ankyrin repeat domain-containing protein 2	0.28	0.221	3.4E-02	3	5.3
Q02952	A-kinase anchor protein 12	0.28	0.077	2.2E-07	19	12.8
P02751	Fibronectin	0.25	0.108	1.7E-03	12	5.9
P07942	Laminin subunit beta-1	0.23	0.167	4.0E-04	4	2.0
P01024	Complement C3	0.23	0.179	6.1E-06	14	8.2
P12111	Collagen alpha-3(VI) chain	0.22	0.051	0.0E+00	31	10.7
P02679	Fibrinogen gamma chain	0.22	0.141	6.9E-05	9	19.0
P12110	Collagen alpha-2(VI) chain	0.22	0.076	2.2E-10	8	8.5
P12109	Collagen alpha-1(VI) chain	0.21	0.087	3.5E-09	9	9.8
P07355	Annexin A2	0.21	0.039	0.0E+00	22	55.8
P00167	Cytochrome b5	0.21	0.133	3.6E-05	3	35.8
P46821	Microtubule-associated protein 1B	0.21	0.204	2.2E-02	3	1.3
Q13425	Beta-2-syntrophin	0.21	0.210	3.0E-02	4	6.7
P27105	Erythrocyte band 7 integral membrane protein	0.21	0.094	6.9E-10	5	15.6
P05164	Myeloperoxidase	0.21	0.318	2.4E-02	4	6.3
Q01082	Spectrin beta chain, non-erythrocytic 1	0.21	0.044	0.0E+00	41	20.1
Q13813	Spectrin alpha chain, non-erythrocytic 1	0.20	0.034	0.0E+00	60	26.5
P39059	Collagen alpha-1(XV) chain	0.20	0.127	9.3E-06	6	4.1
Q16555	Dihydropyrimidinase-related protein 2	0.20	0.056	2.4E-12	5	9.4
P21333	Filamin-A	0.20	0.045	0.0E+00	42	21.0
O43491	Band 4.1-like protein 2	0.20	0.172	2.5E-05	4	4.9
P09936	Ubiquitin carboxyl-terminal hydrolase isozyme L1	0.19	0.271	6.1E-03	3	19.3
P02649	Apolipoprotein E	0.19	0.076	1.8E-12	12	37.5
P36269	Gamma-glutamyltransferase 5	0.18	0.124	1.4E-02	3	6.1
P11166	Solute carrier family 2, facilitated glucose transporter member 1	0.18	0.092	1.6E-08	3	5.5
P02749	Beta-2-glycoprotein 1	0.18	0.123	2.3E-05	3	7.0
P15088	Mast cell carboxypeptidase A	0.18	0.153	4.0E-04	4	7.9
P02675	Fibrinogen beta chain	0.18	0.093	1.7E-11	11	27.1
P98160	Basement membrane-specific heparan sulfate proteoglycan core protein	0.18	0.069	0.0E+00	33	8.8
P02671	Fibrinogen alpha chain	0.18	0.115	3.9E-07	7	9.6
P39060	Collagen alpha-1(XVIII) chain	0.18	0.090	2.0E-11	6	3.6
P55268	Laminin subunit beta-2	0.18	0.084	4.9E-13	12	7.7
P60903	Protein S100-A10	0.16	0.130	1.1E-03	3	35.1
P43320	Beta-crystallin B2	0.16	0.151	1.8E-02	3	15.6
P11277	Spectrin beta chain, erythrocytic	0.16	0.158	1.1E-04	8	4.5
P01011	Alpha-1-antichymotrypsin	0.16	0.101	3.0E-10	4	8.3
O15230	Laminin subunit alpha-5	0.16	0.160	7.9E-05	12	4.3
P50895	Basal cell adhesion molecule	0.15	0.183	4.3E-04	6	13.1
O00468	Agrin	0.15	0.040	8.8E-05	3	1.8
O94875	Sorbin and SH3 domain-containing protein 2	0.15	0.438	3.3E-02	3	4.7
P08294	Extracellular superoxide dismutase [Cu-Zn]	0.15	0.177	1.7E-04	3	15.4
P62736	Actin, aortic smooth muscle	0.14	0.198	4.4E-07	6	21.0
P11047	Laminin subunit gamma-1	0.14	0.196	6.0E-05	11	6.5
Q14112	Nidogen-2	0.14	0.170	4.7E-07	9	7.4
P80723	Brain acid soluble protein 1	0.14	0.217	1.6E-02	3	21.1
P08572	Collagen alpha-2(IV) chain	0.13	0.124	8.8E-10	5	3.5
P51888	Prolargin	0.13	0.058	0.0E+00	13	35.6
P35749	Myosin-11	0.13	0.133	5.4E-14	31	17.0
P02730	Band 3 anion transport protein	0.13	0.151	2.3E-05	7	10.3
Q05707	Collagen alpha-1(XIV) chain	0.13	0.198	6.1E-05	8	4.5
P14543	Nidogen-1	0.13	0.125	7.1E-05	9	8.3
P23946	Chymase	0.13	0.261	8.6E-03	3	16.6
Q14195	Dihydropyrimidinase-related protein 3	0.12	0.147	1.1E-08	5	11.9
P01031	Complement C5	0.11	0.346	2.4E-03	5	2.9
P04083	Annexin A1	0.11	0.083	1.5E-12	10	28.3
P02686	Myelin basic protein	0.11	0.180	1.3E-06	3	10.5
Q9Y6C2	EMILIN-1	0.11	0.141	9.2E-07	6	7.3
P01008	Antithrombin-III	0.11	0.345	9.5E-03	6	14.0
P35625	Metalloproteinase inhibitor 3	0.11	0.115	6.1E-12	4	15.6
P04275	von Willebrand factor	0.10	0.136	6.5E-10	7	2.9
Q15661	Tryptase alpha/beta-1	0.10	0.177	1.1E-07	5	20.7
P41219	Peripherin	0.09	0.150	1.4E-07	11	24.9
P21810	Biglycan	0.09	0.097	2.4E-15	11	31.0
P22352	Glutathione peroxidase 3	0.09	0.142	1.9E-05	3	11.5
P02743	Serum amyloid P-component	0.09	0.147	8.6E-06	6	23.3
P07585	Decorin	0.08	0.144	2.8E-06	4	9.5
P35555	Fibrillin-1	0.08	0.064	0.0E+00	42	14.9
P02748	Complement component C9	0.08	0.149	6.4E-10	9	15.9
P21980	Protein-glutamine gamma-glutamyltransferase 2	0.08	0.099	0.0E+00	14	19.1
P21926	CD9 antigen	0.07	0.228	5.9E-05	3	9.6
P10909	Clusterin	0.07	0.083	4.3E-13	14	28.7
P22748	Carbonic anhydrase 4	0.07	0.146	1.8E-06	6	17.0
P20774	Mimecan	0.06	0.198	2.6E-08	9	31.2
P51884	Lumican	0.06	0.071	0.0E+00	8	20.7
P04004	Vitronectin	0.05	0.132	7.7E-14	10	19.5
P25189	Myelin protein P0	0.03	0.205	1.3E-08	7	27.4

Brown denotes change ≥ 2 standard deviations (SD) from the mean, yellow denotes change ≥ 1 SD and green highlights p values ≤ 0.05. NA, not applicable, n<3 unique peptides.
