# Supplementary material for: iTRAQ Quantitative Proteomic Comparison of Metastatic and Non-Metastatic Uveal Melanoma Tumors
Source: PLoS One. 2015 Aug 25;10(8):e0135543. doi: 10.1371/journal.pone.0135543 (PMC4549237; doi:10.1371/journal.pone.0135543)
Supplement: S9 Table — (PDF) [file pone.0135543.s009.pdf]

Supplementary Table S9

Relative Protein Abundance: Sample UM25, Non-Metastatic

Total Proteins Quantified = 868;LogMedian Protein Ratio = 0.11; LogMean Protein Ratio = 0; Standard Deviation = 0.79

Uni-Prot Accession	Protein	Ratio UM/Control	Standard Deviation	*p* value	Unique Peptides	% Sequence Coverage
P09211	Glutathione S-transferase P	8.14	0.081	1.8E-15	9	45.7
P30086	Phosphatidylethanolamine-binding protein 1	8.05	0.083	1.2E-11	9	52.9
P06454	Prothymosin alpha	6.88	0.291	2.2E-03	3	21.6
P62937	Peptidyl-prolyl cis-trans isomerase A	6.71	0.060	0.0E+00	7	38.2
Q9UBR2	Cathepsin Z	6.39	0.152	4.7E-06	3	9.6
P15121	Aldose reductase	5.00	0.140	2.2E-05	5	15.8
Q9Y2S2	Lambda-crystallin homolog	4.73	0.190	4.1E-04	4	13.5
O14556	Glyceraldehyde-3-phosphate dehydrogenase, testis-specific	4.72	0.189	2.9E-04	5	14.5
P00558	Phosphoglycerate kinase 1	4.70	0.091	5.0E-09	9	21.6
P63241	Eukaryotic translation initiation factor 5A-1	4.56	0.190	1.4E-03	4	27.9
P06733	Alpha-enolase	4.53	0.051	8.7E-15	13	39.9
P00338	L-lactate dehydrogenase A chain	4.42	0.067	5.0E-09	8	22.0
P31939	Bifunctional purine biosynthesis protein PURH	4.39	0.140	1.2E-04	4	8.1
P78417	Glutathione S-transferase omega-1	4.24	0.094	1.1E-09	9	31.5
P40925	Malate dehydrogenase, cytoplasmic	4.22	0.145	9.1E-05	6	22.2
P02768	Serum albumin	4.13	0.029	0.0E+00	38	57.0
Q53EL6	Programmed cell death protein 4	4.10	0.125	5.8E-05	3	6.6
P07195	L-lactate dehydrogenase B chain	4.06	0.080	6.1E-09	8	21.3
Q6XQN6	Nicotinate phosphoribosyltransferase	3.83	0.135	1.2E-04	3	6.1
Q16658	Fascin	3.83	0.092	2.0E-04	3	8.1
Q04760	Lactoylglutathione lyase	3.67	0.134	1.3E-04	4	18.5
P60174	Triosephosphate isomerase	3.63	0.114	1.5E-05	9	35.7
P19338	Nucleolin	3.58	0.070	6.4E-14	14	18.3
P52565	Rho GDP-dissociation inhibitor 1	3.48	0.155	6.4E-05	3	22.1
Q9HC38	Glyoxalase domain-containing protein 4	3.44	0.102	1.5E-02	3	10.2
P06737	Glycogen phosphorylase, liver form	3.39	0.080	9.1E-11	19	21.5
P08758	Annexin A5	3.39	0.051	0.0E+00	15	48.4
P10599	Thioredoxin	3.36	0.150	6.8E-04	3	32.4
Q13510	Acid ceramidase	3.35	0.135	4.4E-04	3	7.3
P29401	Transketolase	3.33	0.126	1.4E-04	6	9.5
P15531	Nucleoside diphosphate kinase A	3.28	0.095	2.7E-06	7	52.6
P13797	Plastin-3	3.17	0.106	1.2E-06	6	11.9
P30043	Flavin reductase (NADPH)	3.17	0.244	1.1E-02	3	18.9
P23526	Adenosylhomocysteinase	3.13	0.196	2.8E-03	5	14.1
P16070	CD44 antigen	3.10	0.095	1.5E-05	9	12.1
P02787	Serotransferrin	3.08	0.059	1.6E-15	18	27.9
Q13838	Spliceosome RNA helicase DDX39B	3.06	0.081	4.2E-06	5	12.4
P13489	Ribonuclease inhibitor	3.04	0.155	2.2E-03	4	9.3
Q99497	Protein DJ-1	2.97	0.139	4.1E-04	6	28.0
P22087	rRNA 2'-O-methyltransferase fibrillarin	2.87	0.108	3.3E-03	7	27.4
P01859	Ig gamma-2 chain C region	2.85	0.067	6.2E-04	3	12.0
P06748	Nucleophosmin	2.85	0.115	1.5E-04	6	17.0
P57729	Ras-related protein Rab-38	2.84	0.144	1.1E-02	3	12.3
O95336	6-phosphogluconolactonase	2.82	0.139	1.5E-02	4	20.9
P31948	Stress-induced-phosphoprotein 1	2.76	0.100	2.1E-08	15	27.1
P04080	Cystatin-B	2.75	0.159	6.2E-03	3	39.8
P30837	Aldehyde dehydrogenase X, mitochondrial	2.75	0.112	3.9E-06	7	17.6
P07741	Adenine phosphoribosyltransferase	2.69	0.360	2.3E-02	3	16.7
P40967	Melanocyte protein PMEL	2.62	0.118	1.3E-02	3	5.9
P08195	4F2 cell-surface antigen heavy chain	2.59	0.114	5.8E-05	8	15.4
Q8IZQ5	Selenoprotein H	2.59	0.187	4.7E-02	3	25.4
P07900	Heat shock protein HSP 90-alpha	2.57	0.066	4.3E-07	13	14.8
P09429	High mobility group protein B1	2.51	0.197	2.6E-03	6	18.6
P06865	Beta-hexosaminidase subunit alpha	2.49	0.132	3.7E-02	3	5.5
P61604	10 kDa heat shock protein, mitochondrial	2.47	0.089	2.2E-05	6	51.0
P07737	Profilin-1	2.46	0.147	6.1E-04	5	34.3
P62750	60S ribosomal protein L23a	2.44	0.055	4.6E-05	5	27.6
Q01105	Protein SET	2.39	0.212	9.4E-03	4	16.2
P08238	Heat shock protein HSP 90-beta	2.39	0.067	1.5E-07	9	12.6
P50395	Rab GDP dissociation inhibitor beta	2.37	0.130	3.3E-04	6	16.6
P26641	Elongation factor 1-gamma	2.35	0.157	1.5E-02	4	8.9
Q14240	Eukaryotic initiation factor 4A-II	2.34	0.149	1.1E-02	4	12.0
P25398	40S ribosomal protein S12	2.34	0.075	8.3E-04	3	22.0
P07910	Heterogeneous nuclear ribonucleoproteins C1/C2	2.33	0.080	8.6E-12	9	26.5
Q07955	Serine/arginine-rich splicing factor 1	2.33	0.104	3.1E-03	5	17.3
P16152	Carbonyl reductase [NADPH] 1	2.31	0.153	6.6E-04	5	23.1
Q9Y4W6	AFG3-like protein 2	2.29	0.157	1.2E-02	4	6.4
P31153	S-adenosylmethionine synthase isoform type-2	2.28	0.037	4.6E-03	3	9.4
P05387	60S acidic ribosomal protein P2	2.27	0.089	5.0E-05	6	60.9
P61353	60S ribosomal protein L27	2.27	0.154	4.4E-03	3	30.1
P30041	Peroxiredoxin-6	2.26	0.041	2.7E-13	7	25.0
P21283	V-type proton ATPase subunit C 1	2.25	0.120	3.5E-03	4	8.1
P23528	Cofilin-1	2.25	0.074	7.7E-03	4	27.7
P21266	Glutathione S-transferase Mu 3	2.23	0.077	6.6E-06	4	18.7
P51149	Ras-related protein Rab-7a	2.22	0.065	5.6E-09	7	37.2
P07858	Cathepsin B	2.22	0.108	1.7E-03	4	13.0
O00567	Nucleolar protein 56	2.20	0.191	2.9E-02	4	6.7
O00625	Pirin	9.16	NA	NA	2	6.2
P62328	Thymosin beta-4	5.12	NA	NA	2	43.2
P24941	Cyclin-dependent kinase 2	3.94	NA	NA	2	8.7
P63173	60S ribosomal protein L38	3.90	NA	NA	2	34.3
Q15631	Translin	3.78	NA	NA	2	11.8
Q8IV08	Phospholipase D3	3.78	NA	NA	2	4.3
P58546	Myotrophin	3.69	NA	NA	2	25.4
Q96C86	m7GpppX diphosphatase	3.43	NA	NA	2	7.1
Q04446	1,4-alpha-glucan-branching enzyme	3.39	NA	NA	2	4.4
O75348	V-type proton ATPase subunit G 1	2.98	NA	NA	2	19.5
P07108	Acyl-CoA-binding protein	2.89	NA	NA	2	41.4
P17096	High mobility group protein HMG-I/HMG-Y	2.78	NA	NA	2	23.4
Q15843	NEDD8	2.75	NA	NA	2	17.3
O15400	Syntaxin-7	2.72	NA	NA	2	8.8
Q658Y4	Protein FAM91A1	2.63	NA	NA	2	1.9
O75347	Tubulin-specific chaperone A	2.59	0.420	1.6E-01	4	33.3
Q9UH65	Switch-associated protein 70	2.59	0.269	6.7E-02	3	3.6
Q13442	28 kDa heat- and acid-stable phosphoprotein	2.56	NA	NA	2	13.8
P30044	Peroxiredoxin-5, mitochondrial	2.56	NA	NA	2	11.7
Q9Y2W1	Thyroid hormone receptor-associated protein 3	2.56	NA	NA	2	3.4
P13798	Acylamino-acid-releasing enzyme	2.46	NA	NA	2	2.3
Q9BRA2	Thioredoxin domain-containing protein 17	2.43	NA	NA	2	18.7
Q8WWM7	Ataxin-2-like protein	2.42	NA	NA	2	1.8
Q00796	Sorbitol dehydrogenase	2.42	NA	NA	2	4.8
P00568	Adenylate kinase isoenzyme 1	2.32	NA	NA	2	10.3
P12955	Xaa-Pro dipeptidase	2.28	NA	NA	2	3.7
Q9Y2X3	Nucleolar protein 58	2.28	0.326	6.9E-02	3	8.1
Q03154	Aminoacylase-1	2.27	0.222	1.6E-01	3	8.6
P55786	Puromycin-sensitive aminopeptidase	2.26	0.170	7.8E-02	3	3.6
P13693	Translationally-controlled tumor protein	2.25	NA	NA	2	15.7
Q14677	Clathrin interactor 1	2.24	NA	NA	2	3.4

Q99436	Proteasome subunit beta type-7	2.22	NA	NA	2	7.2
Q9BUJ2	Heterogeneous nuclear ribonucleoprotein U-like protein 1	2.22	NA	NA	2	2.5
Q9Y3C8	Ubiquitin-fold modifier-conjugating enzyme 1	2.21	NA	NA	2	11.4
P24534	Elongation factor 1-beta	2.19	0.085	3.7E-03	3	9.8
P83731	60S ribosomal protein L24	2.19	0.065	4.1E-04	3	21.0
P39019	40S ribosomal protein S19	2.18	0.156	6.6E-03	4	24.1
P37837	Transaldolase	2.18	0.128	1.4E-02	5	13.4
Q08211	ATP-dependent RNA helicase A	2.17	0.082	2.4E-07	9	8.9
P29966	Myristoylated alanine-rich C-kinase substrate	2.17	0.293	6.1E-02	6	31.6
Q96KP4	Cytosolic non-specific dipeptidase	2.16	0.079	1.9E-04	4	11.2
P09661	U2 small nuclear ribonucleoprotein A'	2.16	0.174	3.8E-02	3	14.1
Q14247	Src substrate cortactin	2.15	0.176	5.5E-03	7	16.0
Q9UKV3	Apoptotic chromatin condensation inducer in the nucleus	2.12	NA	NA	2	2.1
O14841	5-oxoprolinase	2.12	0.254	9.8E-02	4	5.1
P13639	Elongation factor 2	2.12	0.183	5.8E-04	9	13.4
Q12905	Interleukin enhancer-binding factor 2	2.12	0.094	3.1E-04	3	9.5
P61254	60S ribosomal protein L26	2.10	NA	NA	2	11.7
Q9UQ80	Proliferation-associated protein 2G4	2.09	0.738	3.7E-01	3	7.6
P61313	60S ribosomal protein L15	2.09	NA	NA	2	12.3
P62263	40S ribosomal protein S14	2.09	0.084	5.9E-04	4	37.7
O75083	WD repeat-containing protein 1	2.09	0.139	3.6E-03	5	9.4
P60842	Eukaryotic initiation factor 4A-I	2.09	0.138	1.8E-03	6	16.0
P08865	40S ribosomal protein SA	2.09	0.068	1.4E-04	7	30.2
P62851	40S ribosomal protein S25	2.08	0.053	2.7E-05	4	24.0
P51858	Hepatoma-derived growth factor	2.08	0.218	9.6E-02	3	14.6
P07339	Cathepsin D	2.07	0.074	2.2E-05	5	11.7
P02790	Hemopexin	2.06	0.063	2.8E-05	3	6.3
P99999	Cytochrome c	2.06	0.125	5.7E-03	3	24.8
P46777	60S ribosomal protein L5	2.06	0.142	9.4E-04	4	14.5
P51608	Methyl-CpG-binding protein 2	2.06	0.170	1.1E-01	3	8.0
Q9Y6C9	Mitochondrial carrier homolog 2	2.06	NA	NA	2	7.3
Q15907	Ras-related protein Rab-11B	2.05	0.123	5.1E-02	3	14.7
Q07020	60S ribosomal protein L18	2.05	0.127	2.0E-03	3	18.6
P35613	Basigin	2.05	0.321	1.4E-01	3	10.9
Q9UHX1	Poly(U)-binding-splicing factor PUF60	2.05	NA	NA	2	3.4
Q13185	Chromobox protein homolog 3	2.05	0.080	1.9E-05	4	26.8
P49773	Histidine triad nucleotide-binding protein 1	2.05	0.192	1.5E-01	3	31.7
P51991	Heterogeneous nuclear ribonucleoprotein A3	2.03	0.047	7.2E-08	7	20.6
P26640	Valine--tRNA ligase	2.03	NA	NA	2	2.4
P27635	60S ribosomal protein L10	2.03	NA	NA	2	9.8
P10768	S-formylglutathione hydrolase	1.98	0.507	1.9E-01	4	13.1
P11216	Glycogen phosphorylase, brain form	1.98	0.077	6.6E-05	8	12.6
P63104	14-3-3 protein zeta/delta	1.98	0.120	1.8E-02	4	22.9
P29692	Elongation factor 1-delta	1.98	0.085	6.5E-04	4	15.7
P62906	60S ribosomal protein L10a	1.97	0.082	4.6E-07	6	27.6
P17643	5,6-dihydroxyindole-2-carboxylic acid oxidase	1.95	0.167	3.6E-02	7	15.3
P12081	Histidine--tRNA ligase, cytoplasmic	1.94	NA	NA	2	3.5
P26373	60S ribosomal protein L13	1.94	0.056	1.8E-06	3	14.2
P0CW22	40S ribosomal protein S17-like	1.94	0.078	2.4E-03	3	16.3
P62826	GTP-binding nuclear protein Ran	1.93	NA	NA	2	13.9
Q9BR76	Coronin-1B	1.93	NA	NA	2	3.7
P06744	Glucose-6-phosphate isomerase	1.93	0.195	3.8E-02	4	7.0
P60866	40S ribosomal protein S20	1.93	NA	NA	2	19.3
Q8N1G4	Leucine-rich repeat-containing protein 47	1.92	NA	NA	2	4.3
Q9BZQ8	Protein Niban	1.92	0.082	3.4E-03	4	4.3
P46926	Glucosamine-6-phosphate isomerase 1	1.92	0.081	8.5E-03	5	12.5
P51159	Ras-related protein Rab-27A	1.92	0.116	8.6E-03	3	15.4
P22314	Ubiquitin-like modifier-activating enzyme 1	1.91	0.171	2.9E-03	5	5.3
Q99536	Synaptic vesicle membrane protein VAT-1 homolog	1.91	0.082	1.7E-05	12	38.7
Q12906	Interleukin enhancer-binding factor 3	1.89	0.123	3.4E-03	10	13.4
P36578	60S ribosomal protein L4	1.89	0.083	2.9E-05	7	15.7
Q8WUY3	Protein prune homolog 2	1.88	NA	NA	2	0.8
P62424	60S ribosomal protein L7a	1.88	0.138	8.6E-03	5	12.4
P12270	Nucleoprotein TPR	1.87	0.077	2.8E-03	8	4.9
P16401	Histone H1.5	1.87	0.150	7.3E-02	4	15.0
Q86UE4	Protein LYRIC	1.87	NA	NA	2	3.8
P18621	60S ribosomal protein L17	1.86	NA	NA	2	10.9
O75380	NADH dehydrogenase [ubiquinone] iron-sulfur protein 6, mitochondrial	1.85	NA	NA	2	20.2
P01857	Ig gamma-1 chain C region	1.85	0.059	1.3E-06	4	18.5
P54819	Adenylate kinase 2, mitochondrial	1.84	0.113	2.3E-02	3	15.5
P40926	Malate dehydrogenase, mitochondrial	1.83	0.055	1.3E-07	11	34.9
P62158	Calmodulin	1.83	0.151	2.4E-02	5	32.2
P62829	60S ribosomal protein L23	1.83	0.035	4.5E-03	3	23.6
P04406	Glyceraldehyde-3-phosphate dehydrogenase	1.83	0.062	1.4E-06	8	23.6
Q02878	60S ribosomal protein L6	1.82	0.121	2.6E-03	10	30.2
P25786	Proteasome subunit alpha type-1	1.82	0.072	2.7E-04	5	16.0
P14868	Aspartate--tRNA ligase, cytoplasmic	1.82	0.110	1.9E-04	5	13.4
Q92597	Protein NDRG1	1.80	NA	NA	2	5.6
Q16836	Hydroxyacyl-coenzyme A dehydrogenase, mitochondrial	1.80	0.066	1.6E-03	3	10.2
P62280	40S ribosomal protein S11	1.79	0.077	2.6E-03	6	27.8
Q1KMD3	Heterogeneous nuclear ribonucleoprotein U-like protein 2	1.78	0.048	3.4E-04	4	4.3
P04792	Heat shock protein beta-1	1.77	0.043	5.2E-08	6	31.2
P46783	40S ribosomal protein S10	1.77	NA	NA	2	14.5
P62258	14-3-3 protein epsilon	1.77	0.088	2.9E-03	8	32.2
Q00839	Heterogeneous nuclear ribonucleoprotein U	1.77	0.060	1.6E-05	11	13.0
P02765	Alpha-2-HS-glycoprotein	1.77	0.236	7.2E-02	4	9.3
Q9H307	Pinin	1.75	NA	NA	2	3.9
P20618	Proteasome subunit beta type-1	1.75	NA	NA	2	9.5
O14979	Heterogeneous nuclear ribonucleoprotein D-like	1.75	0.078	2.6E-03	3	4.5
P61247	40S ribosomal protein S3a	1.74	0.203	8.5E-02	5	20.5
P27348	14-3-3 protein theta	1.74	NA	NA	2	10.2
P54652	Heat shock-related 70 kDa protein 2	1.73	0.110	6.4E-02	7	13.1
P28161	Glutathione S-transferase Mu 2	1.73	NA	NA	2	6.9
Q9NY12	H/ACA ribonucleoprotein complex subunit 1	1.73	NA	NA	2	7.4
Q14103	Heterogeneous nuclear ribonucleoprotein D0	1.72	NA	NA	2	6.8
P18669	Phosphoglycerate mutase 1	1.72	NA	NA	2	15.4
Q12913	Receptor-type tyrosine-protein phosphatase eta	1.71	0.082	6.7E-03	4	4.0
P62913	60S ribosomal protein L11	1.71	0.056	4.7E-04	3	16.9
P07954	Fumarate hydratase, mitochondrial	1.71	NA	NA	2	3.1
Q9NSD9	Phenylalanine--tRNA ligase beta subunit	1.71	0.069	1.3E-03	3	4.9
P23246	Splicing factor, proline- and glutamine-rich	1.70	0.251	5.0E-02	5	7.4
Q15181	Inorganic pyrophosphatase	1.70	0.200	4.1E-02	3	10.4
Q96BM9	ADP-ribosylation factor-like protein 8A	1.69	NA	NA	2	10.8
Q16629	Serine/arginine-rich splicing factor 7	1.68	NA	NA	2	7.6
P39687	Acidic leucine-rich nuclear phosphoprotein 32 family member A	1.67	0.102	4.7E-03	3	12.9
O43809	Cleavage and polyadenylation specificity factor subunit 5	1.67	NA	NA	2	9.7
P62899	60S ribosomal protein L31	1.67	NA	NA	2	13.6
Q8IWB7	WD repeat and FYVE domain-containing protein 1	1.66	0.159	8.5E-02	3	10.0
P20042	Eukaryotic translation initiation factor 2 subunit 2	1.65	NA	NA	2	9.9
P09417	Dihydropteridine reductase	1.65	NA	NA	2	12.3
P38606	V-type proton ATPase catalytic subunit A	1.64	0.166	2.9E-02	4	8.4
P01834	Ig kappa chain C region	1.64	0.084	9.7E-03	3	48.1
P50914	60S ribosomal protein L14	1.64	NA	NA	2	10.7
Q15233	Non-POU domain-containing octamer-binding protein	1.64	0.217	6.5E-02	5	15.5
B5ME19	Eukaryotic translation initiation factor 3 subunit C-like protein	1.64	0.118	6.6E-03	7	6.6

P62241	40S ribosomal protein S8	1.63	0.155	1.8E-02	4	15.4
P46778	60S ribosomal protein L21	1.63	NA	NA	2	16.9
P50502	Hsc70-interacting protein	1.63	0.140	1.4E-01	3	9.2
P02647	Apolipoprotein A-I	1.63	0.054	5.0E-05	8	26.2
P23396	40S ribosomal protein S3	1.62	0.055	7.7E-07	9	33.3
P01625	Ig kappa chain V-IV region Len	1.62	0.422	2.3E-01	3	28.9
P62249	40S ribosomal protein S16	1.62	0.113	1.5E-03	5	33.6
P32969	60S ribosomal protein L9	1.61	0.118	8.7E-03	3	11.5
P14618	Pyruvate kinase PKM	1.61	0.059	3.4E-08	10	23.0
P62277	40S ribosomal protein S13	1.61	0.100	2.9E-03	6	32.5
P78347	General transcription factor II-I	1.59	0.271	6.5E-02	6	5.0
P63279	SUMO-conjugating enzyme UBC9	1.59	NA	NA	2	16.5
Q96GK7	Fumarylacetoacetate hydrolase domain-containing protein 2A	1.58	0.233	1.2E-01	3	14.3
Q86UX7	Fermitin family homolog 3	1.57	NA	NA	2	3.9
P10809	60 kDa heat shock protein, mitochondrial	1.57	0.104	6.0E-03	13	24.8
O95758	Polypyrimidine tract-binding protein 3	1.57	0.101	1.5E-02	3	3.4
P52597	Heterogeneous nuclear ribonucleoprotein F	1.57	0.053	1.3E-01	3	7.0
P62701	40S ribosomal protein S4, X isoform	1.56	0.130	8.3E-03	6	17.5
P11142	Heat shock cognate 71 kDa protein	1.56	0.073	8.5E-05	11	19.3
P15880	40S ribosomal protein S2	1.56	0.095	6.4E-04	5	21.5
Q96AG4	Leucine-rich repeat-containing protein 59	1.55	NA	NA	2	6.2
Q5VTE0	Putative elongation factor 1-alpha-like 3	1.55	0.072	3.7E-04	11	26.6
P62995	Transformer-2 protein homolog beta	1.55	NA	NA	2	5.9
P49753	Acyl-coenzyme A thioesterase 2, mitochondrial	1.55	NA	NA	2	3.9
Q02543	60S ribosomal protein L18a	1.55	NA	NA	2	12.5
P22626	Heterogeneous nuclear ribonucleoproteins A2/B1	1.55	0.069	7.0E-07	12	28.3
P62917	60S ribosomal protein L8	1.54	NA	NA	2	10.5
P34932	Heat shock 70 kDa protein 4	1.54	0.087	1.6E-02	6	7.5
P54727	UV excision repair protein RAD23 homolog B	1.53	0.268	1.4E-01	4	8.6
P22061	Protein-L-isoaspartate(D-aspartate) O-methyltransferase	1.53	NA	NA	2	13.2
P62269	40S ribosomal protein S18	1.53	0.059	3.8E-03	7	36.8
O15145	Actin-related protein 2/3 complex subunit 3	1.53	0.095	2.4E-03	3	15.7
P0C0S8	Histone H2A type 1	1.52	NA	NA	2	23.1
P62753	40S ribosomal protein S6	1.52	0.115	1.6E-02	5	19.3
P18124	60S ribosomal protein L7	1.52	0.040	5.7E-06	6	23.8
P17858	ATP-dependent 6-phosphofructokinase, liver type	1.52	0.296	1.1E-01	4	7.3
Q00059	Transcription factor A, mitochondrial	1.52	0.097	1.3E-01	3	13.8
Q15008	26S proteasome non-ATPase regulatory subunit 6	1.51	0.140	1.4E-01	3	9.0
O94826	Mitochondrial import receptor subunit TOM70	1.51	0.038	7.7E-05	6	12.3
Q9H2U2	Inorganic pyrophosphatase 2, mitochondrial	1.51	0.161	8.8E-02	3	9.9
P35998	26S protease regulatory subunit 7	1.51	0.129	5.6E-02	3	10.2
P57053	Histone H2B type F-S	1.51	NA	NA	2	7.9
O14773	Tripeptidyl-peptidase 1	1.51	NA	NA	2	5.0
P51817	cAMP-dependent protein kinase catalytic subunit PRKX	1.50	NA	NA	2	3.6
E9PAV3	Nascent polypeptide-associated complex subunit alpha, muscle-specific form	1.50	NA	NA	2	1.3
P07602	Prosaposin	1.50	0.074	1.7E-03	5	7.1
Q92841	Probable ATP-dependent RNA helicase DDX17	1.50	0.079	6.7E-03	5	8.5
Q9Y6M9	NADH dehydrogenase [ubiquinone] 1 beta subcomplex subunit 9	1.50	NA	NA	2	15.6
O00483	NADH dehydrogenase [ubiquinone] 1 alpha subcomplex subunit 4	1.50	NA	NA	2	22.2
Q92945	Far upstream element-binding protein 2	1.50	0.119	1.6E-02	6	9.4
P10644	cAMP-dependent protein kinase type I-alpha regulatory subunit	1.49	0.204	1.8E-01	3	10.0
Q9GZT3	SRA stem-loop-interacting RNA-binding protein, mitochondrial	1.49	NA	NA	2	23.9
P40429	60S ribosomal protein L13a	1.49	0.103	6.3E-03	5	23.6
P31943	Heterogeneous nuclear ribonucleoprotein H	1.49	NA	NA	2	4.9
O60506	Heterogeneous nuclear ribonucleoprotein Q	1.48	0.161	8.2E-02	4	7.9
P11586	C-1-tetrahydrofolate synthase, cytoplasmic	1.48	0.100	4.4E-03	4	5.2
P05388	60S acidic ribosomal protein P0	1.48	NA	NA	2	6.6
Q12904	Aminoacyl tRNA synthase complex-interacting multifunctional protein 1	1.47	NA	NA	2	9.6
P46782	40S ribosomal protein S5	1.47	0.073	1.6E-02	3	8.8
P30050	60S ribosomal protein L12	1.47	0.286	3.0E-01	3	26.1
P36543	V-type proton ATPase subunit E 1	1.47	NA	NA	2	7.5
Q07666	KH domain-containing, RNA-binding, signal transduction-associated protein 1	1.47	0.037	7.5E-06	5	9.3
Q12874	Splicing factor 3A subunit 3	1.46	NA	NA	2	4.0
P09651	Heterogeneous nuclear ribonucleoprotein A1	1.46	0.073	8.3E-06	7	21.0
P61978	Heterogeneous nuclear ribonucleoprotein K	1.45	0.064	1.5E-04	14	32.6
O75533	Splicing factor 3B subunit 1	1.45	NA	NA	2	2.4
P69905	Hemoglobin subunit alpha	1.45	0.059	3.9E-06	7	50.7
O00560	Syntenin-1	1.44	0.484	2.9E-01	3	9.1
P42704	Leucine-rich PPR motif-containing protein, mitochondrial	1.44	0.082	1.3E-03	8	6.0
P38646	Stress-70 protein, mitochondrial	1.43	0.187	1.2E-02	11	18.3
Q9NP81	Serine--tRNA ligase, mitochondrial	1.43	NA	NA	2	6.9
P62857	40S ribosomal protein S28	1.43	NA	NA	2	30.4
Q16531	DNA damage-binding protein 1	1.43	NA	NA	2	1.4
P61421	V-type proton ATPase subunit d 1	1.43	0.020	5.0E-05	3	7.7
Q9NTK5	Obg-like ATPase 1	1.42	NA	NA	2	7.1
Q06323	Proteasome activator complex subunit 1	1.42	0.267	1.8E-01	4	18.5
P27816	Microtubule-associated protein 4	1.41	1.032	3.7E-01	6	8.0
P05155	Plasma protease C1 inhibitor	1.41	NA	NA	2	4.6
Q86VP6	Cullin-associated NEDD8-dissociated protein 1	1.41	0.065	6.2E-03	8	6.8
P00441	Superoxide dismutase [Cu-Zn]	1.41	NA	NA	2	13.0
Q14152	Eukaryotic translation initiation factor 3 subunit A	1.40	0.099	3.7E-02	3	2.7
P09012	U1 small nuclear ribonucleoprotein A	1.40	NA	NA	2	9.2
Q8IY95	Transmembrane protein 192	1.39	NA	NA	2	8.5
P14866	Heterogeneous nuclear ribonucleoprotein L	1.39	0.194	1.7E-01	6	11.7
Q6IAA8	Ragulator complex protein LAMTOR1	1.39	0.193	2.0E-01	3	17.4
P21281	V-type proton ATPase subunit B, brain isoform	1.38	0.345	1.6E-01	4	9.6
P00751	Complement factor B	1.38	0.092	7.4E-02	3	3.7
P53597	Succinyl-CoA ligase [ADP/GDP-forming] subunit alpha, mitochondrial	1.37	0.130	8.4E-02	3	9.8
Q9Y4I1	Unconventional myosin-Va	1.37	0.096	2.0E-02	6	3.3
P46940	Ras GTPase-activating-like protein IQGAP1	1.37	0.167	2.4E-02	14	9.7
P48047	ATP synthase subunit O, mitochondrial	1.37	0.172	1.2E-02	4	25.4
P59998	Actin-related protein 2/3 complex subunit 4	1.37	0.040	5.4E-03	3	16.1
P78371	T-complex protein 1 subunit beta	1.37	0.139	2.1E-02	7	14.0
Q08380	Galectin-3-binding protein	1.37	NA	NA	2	4.3
O43390	Heterogeneous nuclear ribonucleoprotein R	1.36	0.123	1.8E-02	6	8.7
Q6UXV4	Apolipoprotein O-like	1.36	NA	NA	2	11.6
Q15149	Plectin	1.35	0.029	0.0E+00	90	20.1
P50453	Serpin B9	1.35	0.148	1.8E-01	4	14.4
P61158	Actin-related protein 3	1.35	0.092	6.5E-03	7	20.6
P84103	Serine/arginine-rich splicing factor 3	1.35	0.233	1.9E-01	3	20.7
P61981	14-3-3 protein gamma	1.34	NA	NA	2	9.7
P31040	Succinate dehydrogenase [ubiquinone] flavoprotein subunit, mitochondrial	1.34	0.138	1.1E-01	6	11.9
P08670	Vimentin	1.33	0.067	1.2E-04	27	56.4
P02774	Vitamin D-binding protein	1.33	0.056	4.0E-02	3	4.9
P62136	Serine/threonine-protein phosphatase PP1-alpha catalytic subunit	1.33	0.101	2.5E-02	4	15.2
Q06830	Peroxiredoxin-1	1.33	0.091	1.2E-03	9	37.7
Q9UKM9	RNA-binding protein Raly	1.33	0.095	1.5E-01	3	13.7
Q08945	FACT complex subunit SSRP1	1.32	NA	NA	2	2.0
Q15365	Poly(rC)-binding protein 1	1.32	0.072	2.8E-02	3	10.7
Q14974	Importin subunit beta-1	1.32	0.352	1.6E-01	5	7.8
P09874	Poly [ADP-ribose] polymerase 1	1.32	0.145	1.0E-01	8	11.5
P38117	Electron transfer flavoprotein subunit beta	1.31	0.044	9.5E-04	5	17.3
P63244	Guanine nucleotide-binding protein subunit beta-2-like 1	1.31	NA	NA	2	8.8
P02652	Apolipoprotein A-II	1.31	0.295	4.0E-01	3	19.0
Q02252	Methylmalonate-semialdehyde dehydrogenase [acylating], mitochondrial	1.31	NA	NA	2	3.9

O75367	Core histone macro-H2A.1	1.31	0.146	7.1E-02	7	24.2
Q14683	Structural maintenance of chromosomes protein 1A	1.31	0.277	1.0E-01	3	2.8
P21912	Succinate dehydrogenase [ubiquinone] iron-sulfur subunit, mitochondrial	1.30	0.102	2.4E-02	4	13.9
Q9NYF8	Bcl-2-associated transcription factor 1	1.30	NA	NA	2	4.2
P02042	Hemoglobin subunit delta	1.30	NA	NA	2	17.7
Q9Y696	Chloride intracellular channel protein 4	1.30	NA	NA	2	7.1
P09525	Annexin A4	1.30	0.293	6.0E-02	6	16.6
Q13435	Splicing factor 3B subunit 2	1.29	0.115	1.3E-01	6	7.3
Q9Y5M8	Signal recognition particle receptor subunit beta	1.29	NA	NA	2	7.7
A4D1P6	WD repeat-containing protein 91	1.29	NA	NA	2	2.8
O75874	Isocitrate dehydrogenase [NADP] cytoplasmic	1.29	NA	NA	2	5.8
P00505	Aspartate aminotransferase, mitochondrial	1.28	0.060	8.6E-03	7	18.8
A1L0T0	Acetolactate synthase-like protein	1.28	NA	NA	2	4.7
P09669	Cytochrome c oxidase subunit 6C	1.28	0.098	3.3E-02	4	48.0
O43399	Tumor protein D54	1.28	0.083	1.6E-01	3	9.7
P12956	X-ray repair cross-complementing protein 6	1.28	0.096	6.2E-03	10	17.6
Q9Y262	Eukaryotic translation initiation factor 3 subunit L	1.28	0.136	3.0E-01	3	4.8
Q14980	Nuclear mitotic apparatus protein 1	1.28	0.221	7.6E-02	12	7.7
P46781	40S ribosomal protein S9	1.27	0.127	4.4E-02	7	26.3
P11177	Pyruvate dehydrogenase E1 component subunit beta, mitochondrial	1.27	0.026	5.5E-04	5	16.2
Q15717	ELAV-like protein 1	1.27	0.860	6.7E-01	3	15.0
O75643	U5 small nuclear ribonucleoprotein 200 kDa helicase	1.27	0.072	5.1E-02	5	2.6
P62244	40S ribosomal protein S15a	1.27	0.195	1.8E-01	4	29.2
P51665	26S proteasome non-ATPase regulatory subunit 7	1.27	NA	NA	2	5.9
Q969P0	Immunoglobulin superfamily member 8	1.27	NA	NA	2	5.7
Q99798	Aconitate hydratase, mitochondrial	1.27	0.175	2.1E-01	6	9.9
P13010	X-ray repair cross-complementing protein 5	1.27	0.100	2.9E-02	5	7.7
P49591	Serine--tRNA ligase, cytoplasmic	1.27	NA	NA	2	5.3
O15144	Actin-related protein 2/3 complex subunit 2	1.27	0.023	7.6E-04	4	12.0
P61160	Actin-related protein 2	1.26	0.102	2.1E-02	5	18.0
Q13151	Heterogeneous nuclear ribonucleoprotein A0	1.26	0.195	1.0E-01	3	7.5
P08559	Pyruvate dehydrogenase E1 component subunit alpha, somatic form, mitochondrial	1.26	NA	NA	2	5.4
Q9NX63	Coiled-coil-helix-coiled-coil-helix domain-containing protein 3, mitochondrial	1.25	NA	NA	2	6.6
P39023	60S ribosomal protein L3	1.25	0.057	2.7E-02	3	8.7
P51810	G-protein coupled receptor 143	1.25	0.293	2.0E-01	3	9.9
P04075	Fructose-bisphosphate aldolase A	1.25	0.063	2.5E-02	14	45.1
P61204	ADP-ribosylation factor 3	1.25	NA	NA	2	11.6
Q9UIJ7	GTP:AMP phosphotransferase AK3, mitochondrial	1.25	0.057	5.4E-02	5	25.1
P20645	Cation-dependent mannose-6-phosphate receptor	1.24	NA	NA	2	10.5
Q9NVI7	ATPase family AAA domain-containing protein 3A	1.24	NA	NA	2	3.8
O14617	AP-3 complex subunit delta-1	1.24	NA	NA	2	2.3
Q9ULA0	Aspartyl aminopeptidase	1.24	NA	NA	2	5.7
P54709	Sodium/potassium-transporting ATPase subunit beta-3	1.24	0.015	3.7E-02	5	22.6
P47985	Cytochrome b-c1 complex subunit Rieske, mitochondrial	1.23	0.739	3.7E-01	3	11.3
Q14738	Serine/threonine-protein phosphatase 2A 56 kDa regulatory subunit delta isoform	1.23	NA	NA	2	4.0
P50402	Emerin	1.23	NA	NA	2	9.8
O95881	Thioredoxin domain-containing protein 12	1.23	NA	NA	2	14.0
P10412	Histone H1.4	1.23	0.203	1.5E-01	6	15.5
O14818	Proteasome subunit alpha type-7	1.22	NA	NA	2	6.9
P26885	Peptidyl-prolyl cis-trans isomerase FKBP2	1.22	NA	NA	2	9.2
P01876	Ig alpha-1 chain C region	1.22	NA	NA	2	5.9
Q9UHQ9	NADH-cytochrome b5 reductase 1	1.21	0.128	6.0E-02	4	15.1
O75390	Citrate synthase, mitochondrial	1.21	0.082	1.4E-01	5	10.3
P12830	Cadherin-1	1.21	0.204	4.0E-01	3	4.4
Q93050	V-type proton ATPase 116 kDa subunit a isoform 1	1.21	0.090	2.0E-01	4	7.0
O15143	Actin-related protein 2/3 complex subunit 1B	1.21	0.113	2.1E-01	4	14.8
Q08257	Quinone oxidoreductase	1.21	NA	NA	2	8.5
P43686	26S protease regulatory subunit 6B	1.20	0.141	2.0E-01	3	8.1
P30153	Serine/threonine-protein phosphatase 2A 65 kDa regulatory subunit A alpha isoform	1.20	0.047	7.5E-02	3	6.5
O75489	NADH dehydrogenase [ubiquinone] iron-sulfur protein 3, mitochondrial	1.20	0.124	8.5E-02	5	19.3
P68871	Hemoglobin subunit beta	1.20	0.199	4.2E-02	5	44.9
P52272	Heterogeneous nuclear ribonucleoprotein M	1.20	0.125	8.6E-02	7	12.1
P21796	Voltage-dependent anion-selective channel protein 1	1.20	0.082	2.9E-02	7	26.9
Q6PI48	Aspartate--tRNA ligase, mitochondrial	1.19	NA	NA	2	2.2
O15511	Actin-related protein 2/3 complex subunit 5	1.19	0.203	5.4E-01	3	21.9
P04632	Calpain small subunit 1	1.19	0.443	2.2E-01	4	13.8
O60716	Catenin delta-1	1.19	0.192	1.4E-01	3	3.3
Q9NSE4	Isoleucine--tRNA ligase, mitochondrial	1.19	0.082	7.7E-02	4	4.6
Q8N0X7	Spartin	1.19	NA	NA	2	3.2
P37108	Signal recognition particle 14 kDa protein	1.18	NA	NA	2	13.2
P48643	T-complex protein 1 subunit epsilon	1.18	0.111	2.0E-02	10	17.0
P50213	Isocitrate dehydrogenase [NAD] subunit alpha, mitochondrial	1.17	0.077	8.2E-02	3	10.1
Q16851	UTP--glucose-1-phosphate uridylyltransferase	1.17	0.226	2.5E-01	3	6.9
P60228	Eukaryotic translation initiation factor 3 subunit E	1.17	NA	NA	2	3.8
Q14165	Malectin	1.17	NA	NA	2	5.5
Q16698	2,4-dienoyl-CoA reductase, mitochondrial	1.17	1.050	6.3E-01	4	15.2
Q9Y3U8	60S ribosomal protein L36	1.17	0.093	1.5E-01	4	30.5
Q14697	Neutral alpha-glucosidase AB	1.17	3.850	2.6E-01	11	12.0
P20700	Lamin-B1	1.16	0.254	3.7E-01	9	18.1
O00231	26S proteasome non-ATPase regulatory subunit 11	1.16	0.115	2.3E-01	3	5.7
P22695	Cytochrome b-c1 complex subunit 2, mitochondrial	1.16	0.243	2.5E-01	4	12.1
P49411	Elongation factor Tu, mitochondrial	1.16	0.026	3.6E-04	8	19.2
P49327	Fatty acid synthase	1.15	NA	NA	2	1.0
O75947	ATP synthase subunit d, mitochondrial	1.15	0.046	2.8E-01	4	19.9
Q9P2E9	Ribosome-binding protein 1	1.15	0.479	2.5E-01	6	5.2
Q5JWF2	Guanine nucleotide-binding protein G(s) subunit alpha isoforms XLas	1.14	NA	NA	2	3.5
P06753	Tropomyosin alpha-3 chain	1.14	0.273	3.4E-01	4	13.0
P51531	Probable global transcription activator SNF2L2	1.14	NA	NA	2	1.7
P62318	Small nuclear ribonucleoprotein Sm D3	1.14	0.081	5.0E-01	3	31.7
P63000	Ras-related C3 botulinum toxin substrate 1	1.13	0.053	2.1E-01	4	24.5
Q13177	Serine/threonine-protein kinase PAK 2	1.13	NA	NA	2	5.9
P13073	Cytochrome c oxidase subunit 4 isoform 1, mitochondrial	1.12	0.022	2.1E-02	4	25.4
Q99832	T-complex protein 1 subunit eta	1.12	0.115	1.2E-01	9	18.2
P11279	Lysosome-associated membrane glycoprotein 1	1.12	NA	NA	2	4.1
Q8NFV4	Alpha/beta hydrolase domain-containing protein 11	1.12	NA	NA	2	8.6
P40227	T-complex protein 1 subunit zeta	1.12	0.476	3.2E-01	7	17.1
P30049	ATP synthase subunit delta, mitochondrial	1.12	NA	NA	2	13.7
Q9Y6G9	Cytoplasmic dynein 1 light intermediate chain 1	1.12	NA	NA	2	5.7
P61225	Ras-related protein Rap-2b	1.12	NA	NA	2	10.4
P50990	T-complex protein 1 subunit theta	1.11	0.089	1.3E-01	10	17.9
P19367	Hexokinase-1	1.11	0.675	7.9E-01	3	3.8
P08107	Heat shock 70 kDa protein 1A/1B	1.11	0.068	1.6E-01	12	22.6
Q13283	Ras GTPase-activating protein-binding protein 1	1.11	0.144	5.9E-01	3	9.9
Q92499	ATP-dependent RNA helicase DDX1	1.11	0.373	3.0E-01	4	5.4
P14927	Cytochrome b-c1 complex subunit 7	1.11	2.382	5.8E-01	4	40.5
P08134	Rho-related GTP-binding protein RhoC	1.11	NA	NA	2	9.8
P04040	Catalase	1.11	1.441	7.9E-01	3	7.8
P55884	Eukaryotic translation initiation factor 3 subunit B	1.11	0.262	6.6E-01	4	6.5
P30740	Leukocyte elastase inhibitor	1.11	NA	NA	2	7.9
P11310	Medium-chain specific acyl-CoA dehydrogenase, mitochondrial	1.11	NA	NA	2	6.2
Q01844	RNA-binding protein EWS	1.10	NA	NA	2	3.4
P07384	Calpain-1 catalytic subunit	1.10	0.044	1.6E-01	3	3.8
P51812	Ribosomal protein S6 kinase alpha-3	1.09	0.322	6.8E-01	3	4.5
P50991	T-complex protein 1 subunit delta	1.09	0.255	4.5E-01	6	13.9
P24752	Acetyl-CoA acetyltransferase, mitochondrial	1.09	0.147	2.5E-01	4	12.2

P31942	Heterogeneous nuclear ribonucleoprotein H3	1.09	NA	NA	2	9.5
P00403	Cytochrome c oxidase subunit 2	1.09	NA	NA	2	7.5
P27708	CAD protein	1.09	0.080	1.7E-01	4	2.1
P54136	Arginine--tRNA ligase, cytoplasmic	1.09	NA	NA	2	3.6
P11387	DNA topoisomerase 1	1.08	0.189	6.3E-01	3	3.8
P55084	Trifunctional enzyme subunit beta, mitochondrial	1.08	0.122	2.3E-01	8	15.8
P43304	Glycerol-3-phosphate dehydrogenase, mitochondrial	1.07	NA	NA	2	3.6
P35232	Prohibitin	1.07	0.122	2.7E-01	8	29.4
P0C0S5	Histone H2A.Z	1.07	NA	NA	2	18.8
P62987	Ubiquitin-60S ribosomal protein L40	1.07	0.458	3.6E-01	9	50.8
P08237	ATP-dependent 6-phosphofructokinase, muscle type	1.07	0.210	6.0E-01	4	6.8
P01009	Alpha-1-antitrypsin	1.07	0.177	5.2E-01	11	30.4
P49368	T-complex protein 1 subunit gamma	1.07	0.079	1.4E-01	6	11.6
O75306	NADH dehydrogenase [ubiquinone] iron-sulfur protein 2, mitochondrial	1.07	0.475	8.6E-01	3	6.7
Q86UP2	Kinectin	1.07	0.592	6.0E-01	5	4.6
P46977	Dolichyl-diphosphooligosaccharide--protein glycosyltransferase subunit STT3A	1.06	NA	NA	2	2.3
Q13200	26S proteasome non-ATPase regulatory subunit 2	1.06	0.206	5.5E-01	4	4.4
P78527	DNA-dependent protein kinase catalytic subunit	1.06	0.448	3.8E-01	11	2.6
P26368	Splicing factor U2AF 65 kDa subunit	1.05	NA	NA	2	3.8
Q96FW1	Ubiquitin thioesterase OTUB1	1.05	NA	NA	2	6.6
P28838	Cytosol aminopeptidase	1.05	NA	NA	2	4.4
P11940	Polyadenylate-binding protein 1	1.05	0.146	4.3E-01	4	8.2
Q9Y3Z3	Deoxynucleoside triphosphate triphosphohydrolase SAMHD1	1.04	0.033	2.5E-01	3	5.3
P43307	Translocon-associated protein subunit alpha	1.04	NA	NA	2	6.6
P45880	Voltage-dependent anion-selective channel protein 2	1.04	0.762	6.5E-01	6	21.8
O15212	Prefoldin subunit 6	1.04	NA	NA	2	14.0
P31930	Cytochrome b-c1 complex subunit 1, mitochondrial	1.04	0.140	6.3E-01	4	9.8
P47756	F-actin-capping protein subunit beta	1.04	6.390	7.4E-01	3	11.9
P17980	26S protease regulatory subunit 6A	1.04	NA	NA	2	5.2
Q92616	Translational activator GCN1	1.04	NA	NA	2	0.6
P29590	Protein PML	1.03	0.310	7.1E-01	4	5.1
O75964	ATP synthase subunit g, mitochondrial	1.03	NA	NA	2	27.2
Q6UVK1	Chondroitin sulfate proteoglycan 4	1.03	0.431	7.1E-01	3	2.5
Q16891	Mitochondrial inner membrane protein	1.03	0.225	7.8E-01	5	8.7
Q9HD20	Manganese-transporting ATPase 13A1	1.02	NA	NA	2	2.6
P13987	CD59 glycoprotein	1.02	NA	NA	2	15.6
Q8NC56	LEM domain-containing protein 2	1.02	0.169	9.0E-01	3	7.0
O60664	Perilipin-3	1.02	NA	NA	2	7.4
Q12965	Unconventional myosin-Ie	1.02	NA	NA	2	1.8
Q9H223	EH domain-containing protein 4	1.01	NA	NA	2	3.3
Q8IYW2	Tetratricopeptide repeat protein 40	1.01	NA	NA	2	0.4
P15311	Ezrin	1.01	0.061	8.5E-01	6	8.7
O14561	Acyl carrier protein, mitochondrial	1.01	NA	NA	2	9.6
P09622	Dihydrolipoyl dehydrogenase, mitochondrial	1.01	0.154	9.2E-01	4	8.1
Q9BW27	Nuclear pore complex protein Nup85	1.01	NA	NA	2	2.6
P46976	Glycogenin-1	1.00	NA	NA	2	4.9
Q15084	Protein disulfide-isomerase A6	1.00	0.464	9.8E-01	6	16.8
Q92598	Heat shock protein 105 kDa	1.00	0.533	9.6E-01	3	4.1
P61026	Ras-related protein Rab-10	1.00	0.148	9.9E-01	3	15.0
P35221	Catenin alpha-1	1.00	0.476	9.9E-01	7	9.4
P53618	Coatomer subunit beta	1.00	NA	NA	2	2.1
P02545	Prelamin-A/C	0.99	0.463	9.0E-01	33	45.8
P10515	Dihydrolipoyllysine-residue acetyltransferase component of pyruvate dehydrogenase complex, mitochondrial	0.99	0.815	9.4E-01	3	3.9
Q8N5K1	CDGSH iron-sulfur domain-containing protein 2	0.99	NA	NA	2	15.6
P33176	Kinesin-1 heavy chain	0.99	0.358	9.0E-01	3	4.7
P17987	T-complex protein 1 subunit alpha	0.99	0.427	8.9E-01	5	9.5
Q92688	Acidic leucine-rich nuclear phosphoprotein 32 family member B	0.99	NA	NA	2	9.6
P25705	ATP synthase subunit alpha, mitochondrial	0.98	6.835	7.1E-01	14	27.1
P27797	Calreticulin	0.98	0.360	9.2E-01	6	18.9
P06576	ATP synthase subunit beta, mitochondrial	0.98	0.367	6.7E-01	10	23.6
O43242	26S proteasome non-ATPase regulatory subunit 3	0.98	NA	NA	2	3.0
O75323	Protein NipSnap homolog 2	0.98	NA	NA	2	8.7
Q9Y512	Sorting and assembly machinery component 50 homolog	0.98	2.995	9.2E-01	4	9.4
P51116	Fragile X mental retardation syndrome-related protein 2	0.98	NA	NA	2	4.0
P38919	Eukaryotic initiation factor 4A-III	0.98	NA	NA	2	5.6
P46459	Vesicle-fusing ATPase	0.97	0.425	8.5E-01	3	3.9
P78344	Eukaryotic translation initiation factor 4 gamma 2	0.97	NA	NA	2	1.5
Q99623	Prohibitin-2	0.96	0.213	5.0E-01	6	22.7
P04844	Dolichyl-diphosphooligosaccharide--protein glycosyltransferase subunit 2	0.96	NA	NA	2	4.1
Q15293	Reticulocalbin-1	0.96	0.225	7.6E-01	4	13.6
P17844	Probable ATP-dependent RNA helicase DDX5	0.96	0.256	7.9E-01	3	4.9
P09543	2',3'-cyclic-nucleotide 3'-phosphodiesterase	0.96	4.664	6.3E-01	8	15.4
P26038	Moesin	0.95	0.500	5.8E-01	5	8.1
P50454	Serpin H1	0.95	0.448	7.6E-01	3	8.9
P26196	Probable ATP-dependent RNA helicase DDX6	0.95	0.096	4.4E-01	3	6.2
Q99442	Translocation protein SEC62	0.95	0.468	6.9E-01	3	7.3
Q9UJZ1	Stomatin-like protein 2, mitochondrial	0.95	NA	NA	2	8.7
P23284	Peptidyl-prolyl cis-trans isomerase B	0.95	0.357	5.6E-01	8	35.2
Q13228	Selenium-binding protein 1	0.94	NA	NA	2	4.4
P30101	Protein disulfide-isomerase A3	0.94	0.577	4.8E-01	12	22.4
O15173	Membrane-associated progesterone receptor component 2	0.94	0.368	7.4E-01	4	22.0
Q96AE4	Far upstream element-binding protein 1	0.94	0.421	7.3E-01	3	3.9
Q8NBS9	Thioredoxin domain-containing protein 5	0.94	0.282	7.2E-01	5	10.6
Q14258	E3 ubiquitin/ISG15 ligase TRIM25	0.93	NA	NA	2	3.8
Q9NYU2	UDP-glucose:glycoprotein glucosyltransferase 1	0.93	0.552	8.8E-01	3	2.3
P40939	Trifunctional enzyme subunit alpha, mitochondrial	0.93	0.076	1.8E-01	12	18.0
P52907	F-actin-capping protein subunit alpha-1	0.93	NA	NA	2	12.2
P39656	Dolichyl-diphosphooligosaccharide--protein glycosyltransferase 48 kDa subunit	0.93	0.736	3.2E-01	4	7.9
P00390	Glutathione reductase, mitochondrial	0.92	NA	NA	2	6.3
P04843	Dolichyl-diphosphooligosaccharide--protein glycosyltransferase subunit 1	0.92	0.103	2.6E-01	12	22.6
Q15691	Microtubule-associated protein RP/EB family member 1	0.92	2.326	7.8E-01	5	15.7
P42765	3-ketoacyl-CoA thiolase, mitochondrial	0.92	0.191	6.2E-01	4	11.6
P14854	Cytochrome c oxidase subunit 6B1	0.91	1.720	8.5E-01	3	24.4
P42167	Lamina-associated polypeptide 2, isoforms beta/gamma	0.91	1.337	7.7E-01	3	7.3
Q53GQ0	Estradiol 17-beta-dehydrogenase 12	0.90	0.140	3.6E-01	4	13.1
Q9BRX8	Redox-regulatory protein FAM213A	0.90	0.088	2.9E-01	3	13.1
Q13561	Dynactin subunit 2	0.90	0.124	2.5E-01	6	16.2
P11498	Pyruvate carboxylase, mitochondrial	0.90	NA	NA	2	2.1
P30519	Heme oxygenase 2	0.90	NA	NA	2	10.1
Q7L5N1	COP9 signalosome complex subunit 6	0.90	0.134	3.6E-01	3	14.1
Q7KZF4	Staphylococcal nuclease domain-containing protein 1	0.90	0.275	6.5E-01	4	6.5
P62805	Histone H4	0.89	0.175	5.8E-02	7	52.4
Q9Y224	UPF0568 protein C14orf166	0.89	NA	NA	2	6.1
P01860	Ig gamma-3 chain C region	0.89	NA	NA	2	6.4
Q9UNH7	Sorting nexin-6	0.89	NA	NA	2	3.2
P22059	Oxysterol-binding protein 1	0.89	NA	NA	2	2.9
P51572	B-cell receptor-associated protein 31	0.88	2.170	4.1E-01	7	25.6
P60953	Cell division control protein 42 homolog	0.88	NA	NA	2	11.0
P36957	Dihydrolipoyllysine-residue succinyltransferase component of 2-oxoglutarate dehydrogenase complex, mitochondrial	0.88	0.071	1.2E-01	4	9.9
P14314	Glucosidase 2 subunit beta	0.88	0.753	3.9E-01	8	12.1
Q96QK1	Vacuolar protein sorting-associated protein 35	0.88	NA	NA	2	3.0
Q99653	Calcineurin B homologous protein 1	0.87	NA	NA	2	11.8
Q9BVK6	Transmembrane emp24 domain-containing protein 9	0.87	NA	NA	2	6.8
P01042	Kininogen-1	0.87	0.236	4.2E-01	5	7.3
Q09666	Neuroblast differentiation-associated protein AHNAK	0.87	0.094	1.2E-03	95	15.7

Q14847	LIM and SH3 domain protein 1	0.87	NA	NA	2	9.6
Q9Y3I0	tRNA-splicing ligase RtcB homolog	0.86	NA	NA	2	5.0
O43707	Alpha-actinin-4	0.86	0.063	1.8E-02	14	19.1
P10606	Cytochrome c oxidase subunit 5B, mitochondrial	0.86	0.042	1.0E-02	5	30.2
P54577	Tyrosine--tRNA ligase, cytoplasmic	0.86	NA	NA	2	4.4
Q9Y230	RuvB-like 2	0.85	NA	NA	2	5.2
P31689	DnaJ homolog subfamily A member 1	0.85	NA	NA	2	4.8
Q14203	Dynactin subunit 1	0.85	0.649	5.2E-01	3	3.3
P30048	Thioredoxin-dependent peroxide reductase, mitochondrial	0.85	0.426	5.0E-01	4	17.2
O75165	DnaJ homolog subfamily C member 13	0.85	0.291	4.0E-01	3	1.3
Q03252	Lamin-B2	0.85	0.092	4.5E-02	20	32.7
Q16695	Histone H3.1t	0.85	0.061	2.9E-02	3	14.7
P01023	Alpha-2-macroglobulin	0.84	0.104	8.8E-02	7	6.0
P24539	ATP synthase F(0) complex subunit B1, mitochondrial	0.84	NA	NA	2	9.0
P02511	Alpha-crystallin B chain	0.84	0.169	1.0E-01	6	35.4
P04179	Superoxide dismutase [Mn], mitochondrial	0.84	0.176	1.7E-01	5	20.3
O95202	LETM1 and EF-hand domain-containing protein 1, mitochondrial	0.84	NA	NA	2	3.2
P11021	78 kDa glucose-regulated protein	0.83	0.102	6.5E-03	23	35.0
P50993	Sodium/potassium-transporting ATPase subunit alpha-2	0.83	NA	NA	2	2.5
P17655	Calpain-2 catalytic subunit	0.83	0.117	1.6E-01	6	7.4
P35222	Catenin beta-1	0.83	0.165	5.6E-01	4	6.1
P55072	Transitional endoplasmic reticulum ATPase	0.83	0.069	3.6E-03	12	15.5
Q9NR46	Endophilin-B2	0.82	NA	NA	2	5.3
Q92973	Transportin-1	0.82	0.195	4.0E-01	4	6.1
Q9P2R7	Succinyl-CoA ligase [ADP-forming] subunit beta, mitochondrial	0.81	NA	NA	2	4.1
Q9UHV9	Prefoldin subunit 2	0.81	NA	NA	2	16.9
P07237	Protein disulfide-isomerase	0.81	0.108	2.1E-02	12	20.7
O75915	PRA1 family protein 3	0.80	NA	NA	2	9.6
P13861	cAMP-dependent protein kinase type II-alpha regulatory subunit	0.80	0.067	4.6E-02	3	7.4
P00352	Retinal dehydrogenase 1	0.80	0.288	1.3E-01	5	13.8
Q9UHD8	Septin-9	0.80	0.081	2.7E-02	4	7.2
P20339	Ras-related protein Rab-5A	0.80	NA	NA	2	10.7
Q13596	Sorting nexin-1	0.80	NA	NA	2	5.2
P05023	Sodium/potassium-transporting ATPase subunit alpha-1	0.79	0.041	1.3E-03	12	13.7
Q07157	Tight junction protein ZO-1	0.79	NA	NA	2	1.5
Q13011	Delta(3,5)-Delta(2,4)-dienoyl-CoA isomerase, mitochondrial	0.79	0.159	2.8E-01	4	11.9
Q15435	Protein phosphatase 1 regulatory subunit 7	0.79	NA	NA	2	6.4
P00367	Glutamate dehydrogenase 1, mitochondrial	0.79	0.073	4.5E-02	5	12.2
O96008	Mitochondrial import receptor subunit TOM40 homolog	0.79	NA	NA	2	4.7
P46939	Utrophin	0.78	0.085	5.3E-02	3	1.0
Q99714	3-hydroxyacyl-CoA dehydrogenase type-2	0.78	0.130	4.4E-01	3	11.5
Q00325	Phosphate carrier protein, mitochondrial	0.78	0.076	3.8E-02	4	11.6
P36969	Phospholipid hydroperoxide glutathione peroxidase, mitochondrial	0.78	NA	NA	2	10.2
Q07065	Cytoskeleton-associated protein 4	0.78	0.120	8.8E-02	5	11.1
O94832	Unconventional myosin-Id	0.78	0.355	2.1E-01	6	6.6
P0C0L5	Complement C4-B	0.78	0.076	6.2E-03	9	5.0
P10619	Lysosomal protective protein	0.78	NA	NA	2	5.4
P20674	Cytochrome c oxidase subunit 5A, mitochondrial	0.78	0.115	1.3E-01	5	25.3
Q9UHG3	Prenylcysteine oxidase 1	0.78	0.074	5.7E-02	3	6.3
P53396	ATP-citrate synthase	0.78	NA	NA	2	1.8
O60313	Dynamin-like 120 kDa protein, mitochondrial	0.77	0.122	9.5E-02	6	7.6
P09496	Clathrin light chain A	0.77	0.091	2.4E-01	3	9.3
P17931	Galectin-3	0.77	0.136	1.4E-02	7	31.2
Q7L576	Cytoplasmic FMR1-interacting protein 1	0.76	NA	NA	2	1.8
O94919	Endonuclease domain-containing 1 protein	0.76	0.125	8.0E-02	4	8.6
Q9UQE7	Structural maintenance of chromosomes protein 3	0.75	0.042	1.9E-02	4	4.4
Q13423	NAD(P) transhydrogenase, mitochondrial	0.75	0.410	2.6E-01	3	3.3
P04217	Alpha-1B-glycoprotein	0.75	NA	NA	2	4.8
Q8WUM4	Programmed cell death 6-interacting protein	0.75	0.050	1.6E-03	7	8.1
P49419	Alpha-aminoadipic semialdehyde dehydrogenase	0.74	NA	NA	2	4.5
Q13162	Peroxiredoxin-4	0.73	NA	NA	2	8.9
P05107	Integrin beta-2	0.73	2.098	5.3E-01	3	4.4
Q93009	Ubiquitin carboxyl-terminal hydrolase 7	0.73	NA	NA	2	1.8
P67870	Casein kinase II subunit beta	0.73	NA	NA	2	8.8
P35637	RNA-binding protein FUS	0.73	NA	NA	2	3.0
Q9Y4L1	Hypoxia up-regulated protein 1	0.72	NA	NA	2	2.4
Q02818	Nucleobindin-1	0.72	NA	NA	2	6.5
Q9NTJ5	Phosphatidylinositide phosphatase SAC1	0.72	0.093	7.8E-02	3	4.8
P06899	Histone H2B type 1-J	0.71	NA	NA	2	7.9
P08603	Complement factor H	0.71	NA	NA	2	1.8
P09382	Galectin-1	0.71	0.061	2.4E-05	5	43.7
P54920	Alpha-soluble NSF attachment protein	0.70	0.064	6.9E-03	4	16.6
P18859	ATP synthase-coupling factor 6, mitochondrial	0.70	0.111	1.6E-01	3	31.5
Q9Y4F1	FERM, RhoGEF and pleckstrin domain-containing protein 1	0.70	NA	NA	2	1.9
Q15942	Zyxin	0.69	NA	NA	2	5.4
Q12797	Aspartyl/asparaginyl beta-hydroxylase	0.69	0.261	1.4E-01	5	7.0
Q63ZY3	KN motif and ankyrin repeat domain-containing protein 2	0.68	NA	NA	2	4.2
Q02218	2-oxoglutarate dehydrogenase, mitochondrial	0.67	0.085	2.5E-03	4	4.1
O75844	CAAX prenyl protease 1 homolog	0.67	NA	NA	2	3.8
O60884	DnaJ homolog subfamily A member 2	0.67	NA	NA	2	4.4
O94979	Protein transport protein Sec31A	0.67	NA	NA	2	1.3
Q6DD88	Atlastin-3	0.67	NA	NA	2	4.1
P53621	Coatomer subunit alpha	0.66	0.074	3.4E-02	4	3.4
Q9BS26	Endoplasmic reticulum resident protein 44	0.66	0.025	6.2E-06	3	7.9
P17612	cAMP-dependent protein kinase catalytic subunit alpha	0.66	NA	NA	2	4.3
Q00765	Receptor expression-enhancing protein 5	0.66	NA	NA	2	10.6
P01024	Complement C3	0.64	0.164	1.0E-02	18	11.2
O95865	N(G),N(G)-dimethylarginine dimethylaminohydrolase 2	0.64	NA	NA	2	8.4
P07358	Complement component C8 beta chain	0.64	0.356	2.0E-01	4	7.8
O75746	Calcium-binding mitochondrial carrier protein Aralar1	0.64	NA	NA	2	4.6
P06727	Apolipoprotein A-IV	0.63	0.067	3.6E-05	11	23.5
O00264	Membrane-associated progesterone receptor component 1	0.63	0.172	4.4E-02	3	12.3
Q6NZI2	Polymerase I and transcript release factor	0.63	0.113	1.4E-02	6	19.2
Q9HDC9	Adipocyte plasma membrane-associated protein	0.63	0.018	1.8E-04	3	7.7
P60709	Actin, cytoplasmic 1	0.63	0.045	2.3E-05	6	24.8
Q14204	Cytoplasmic dynein 1 heavy chain 1	0.62	0.037	1.3E-10	20	4.3
Q14344	Guanine nucleotide-binding protein subunit alpha-13	0.62	0.279	7.1E-02	7	22.3
Q9NVD7	Alpha-parvin	0.62	0.099	1.6E-01	3	9.1
Q14254	Flotillin-2	0.62	NA	NA	2	4.7
P05091	Aldehyde dehydrogenase, mitochondrial	0.62	0.172	1.6E-02	3	6.0
P43121	Cell surface glycoprotein MUC18	0.62	0.244	1.3E-01	4	6.8
P08571	Monocyte differentiation antigen CD14	0.62	NA	NA	2	5.3
P50995	Annexin A11	0.61	0.143	5.1E-02	3	5.7
Q00610	Clathrin heavy chain 1	0.61	0.042	3.4E-10	28	18.0
P27824	Calnexin	0.61	0.166	7.2E-02	7	13.5
Q9NQC3	Reticulon-4	0.61	0.035	3.6E-08	3	2.7
P14625	Endoplasmin	0.61	0.061	7.6E-08	12	15.6
P12235	ADP/ATP translocase 1	0.60	0.031	5.2E-03	3	11.1
Q9Y6N5	Sulfide:quinone oxidoreductase, mitochondrial	0.60	0.127	8.8E-03	5	12.9
O00159	Unconventional myosin-Ic	0.59	0.056	1.1E-04	6	5.6
O43865	Putative adenosylhomocysteinase 2	0.59	0.133	1.7E-01	3	4.2
P08174	Complement decay-accelerating factor	0.59	NA	NA	2	3.9
Q969G5	Protein kinase C delta-binding protein	0.59	0.139	4.5E-02	3	7.7
P49748	Very long-chain specific acyl-CoA dehydrogenase, mitochondrial	0.59	0.074	4.9E-03	4	6.4
A0FGR8	Extended synaptotagmin-2	0.58	NA	NA	2	3.0

P00738	Haptoglobin	0.58	0.090	1.7E-02	7	15.8
Q9P0M6	Core histone macro-H2A.2	0.57	0.230	1.8E-01	3	12.6
Q6NUK1	Calcium-binding mitochondrial carrier protein SCaMC-1	0.57	NA	NA	2	4.4
Q07954	Prolow-density lipoprotein receptor-related protein 1	0.57	0.159	1.1E-02	8	2.4
P48681	Nestin	0.56	NA	NA	2	1.5
P21589	5'-nucleotidase	0.56	0.133	2.3E-02	3	5.9
Q92734	Protein TFG	0.56	NA	NA	2	4.5
P40763	Signal transducer and activator of transcription 3	0.55	NA	NA	2	3.6
Q9BQE3	Tubulin alpha-1C chain	0.55	NA	NA	2	6.7
Q15836	Vesicle-associated membrane protein 3	0.55	NA	NA	2	24.0
P13667	Protein disulfide-isomerase A4	0.54	0.050	6.0E-03	3	5.4
Q9H444	Charged multivesicular body protein 4b	0.54	0.347	3.0E-01	3	15.2
P08133	Annexin A6	0.54	0.047	2.6E-13	26	43.5
Q05682	Caldesmon	0.54	0.166	3.0E-02	3	5.2
P16615	Sarcoplasmic/endoplasmic reticulum calcium ATPase 2	0.53	0.083	4.8E-04	6	6.7
Q99584	Protein S100-A13	0.53	0.103	3.1E-04	3	33.7
P00747	Plasminogen	0.53	0.140	2.6E-03	7	10.6
P01903	HLA class II histocompatibility antigen, DR alpha chain	0.53	0.090	3.5E-03	4	21.3
P01893	Putative HLA class I histocompatibility antigen, alpha chain H	0.52	NA	NA	2	5.8
Q01082	Spectrin beta chain, non-erythrocytic 1	0.52	0.029	0.0E+00	49	25.0
Q9HBL0	Tensin-1	0.51	0.312	2.2E-01	4	4.1
P35580	Myosin-10	0.51	0.058	4.2E-06	15	8.8
Q03591	Complement factor H-related protein 1	0.51	NA	NA	2	9.4
P09497	Clathrin light chain B	0.50	NA	NA	2	8.3
Q9H0D6	5'-3' exoribonuclease 2	0.50	NA	NA	2	2.6
Q14764	Major vault protein	0.49	0.139	1.2E-02	3	3.7
P29992	Guanine nucleotide-binding protein subunit alpha-11	0.49	0.155	9.4E-02	3	10.9
P23634	Plasma membrane calcium-transporting ATPase 4	0.49	NA	NA	2	2.4
P48735	Isocitrate dehydrogenase [NADP], mitochondrial	0.49	0.090	9.1E-04	3	7.7
Q15582	Transforming growth factor-beta-induced protein ig-h3	0.49	NA	NA	2	3.5
O95197	Reticulon-3	0.48	NA	NA	2	1.6
P12814	Alpha-actinin-1	0.48	0.053	1.6E-07	13	20.6
O94905	Erlin-2	0.48	0.068	2.0E-04	6	17.4
Q16181	Septin-7	0.48	0.048	6.9E-05	5	12.4
Q14956	Transmembrane glycoprotein NMB	0.48	0.246	2.0E-02	3	6.5
Q9Y490	Talin-1	0.48	0.042	1.3E-15	25	12.6
Q13813	Spectrin alpha chain, non-erythrocytic 1	0.48	0.021	0.0E+00	65	28.2
P20073	Annexin A7	0.48	0.104	3.8E-02	4	10.9
P61764	Syntaxin-binding protein 1	0.48	NA	NA	2	4.4
P04899	Guanine nucleotide-binding protein G(i) subunit alpha-2	0.48	NA	NA	2	6.2
P05362	Intercellular adhesion molecule 1	0.47	0.174	4.0E-02	4	7.9
P54289	Voltage-dependent calcium channel subunit alpha-2/delta-1	0.47	NA	NA	2	2.1
P63010	AP-2 complex subunit beta	0.47	0.150	9.8E-03	5	4.7
P32119	Peroxiredoxin-2	0.47	0.066	1.8E-06	5	19.7
P61769	Beta-2-microglobulin	0.47	0.550	4.8E-01	3	19.3
O14950	Myosin regulatory light chain 12B	0.47	0.056	8.4E-08	3	12.8
Q86VB7	Scavenger receptor cysteine-rich type 1 protein M130	0.47	NA	NA	2	1.8
P05090	Apolipoprotein D	0.46	NA	NA	2	12.7
O95782	AP-2 complex subunit alpha-1	0.44	0.267	1.1E-01	3	3.3
P60033	CD81 antigen	0.44	NA	NA	2	11.9
P35556	Fibrillin-2	0.44	NA	NA	2	0.6
P07305	Histone H1.0	0.43	0.095	6.7E-02	3	16.0
P06756	Integrin alpha-V	0.42	NA	NA	2	1.4
Q93052	Lipoma-preferred partner	0.41	NA	NA	2	4.2
Q7Z7G0	Target of Nesh-SH3	0.40	NA	NA	2	1.7
P07197	Neurofilament medium polypeptide	0.40	NA	NA	2	2.5
Q13425	Beta-2-syntrophin	0.39	0.326	2.3E-01	4	5.7
Q6UXB8	Peptidase inhibitor 16	0.39	NA	NA	2	5.2
Q03135	Caveolin-1	0.38	NA	NA	2	13.5
P09619	Platelet-derived growth factor receptor beta	0.37	NA	NA	2	2.3
Q14624	Inter-alpha-trypsin inhibitor heavy chain H4	0.36	NA	NA	2	2.2
Q96CX2	BTB/POZ domain-containing protein KCTD12	0.36	NA	NA	2	5.5
P02749	Beta-2-glycoprotein 1	0.33	NA	NA	2	7.0
Q9BS40	Latexin	0.33	NA	NA	2	12.2
P04216	Thy-1 membrane glycoprotein	0.33	NA	NA	2	15.5
P13671	Complement component C6	0.32	NA	NA	2	2.6
P08311	Cathepsin G	0.29	NA	NA	2	6.7
P23946	Chymase	0.28	NA	NA	2	10.1
P05164	Myeloperoxidase	0.27	0.188	9.1E-02	3	4.8
P61626	Lysozyme C	0.27	NA	NA	2	12.8
P02654	Apolipoprotein C-I	0.26	NA	NA	2	24.1
P10643	Complement component C7	0.26	0.140	1.3E-01	3	4.5
P41222	Prostaglandin-H2 D-isomerase	0.25	NA	NA	2	12.1
Q9NY15	Stabilin-1	0.24	NA	NA	2	1.0
O00468	Agrin	0.24	NA	NA	2	1.2
P22105	Tenascin-X	0.23	0.239	1.1E-01	4	1.1
P16157	Ankyrin-1	0.23	NA	NA	2	1.5
P43320	Beta-crystallin B2	0.22	NA	NA	2	11.7
Q01995	Transgelin	0.21	NA	NA	2	9.5
Q9UBX5	Fibulin-5	0.20	NA	NA	2	3.6
P58166	Inhibin beta E chain	0.20	NA	NA	2	6.6
Q12805	EGF-containing fibulin-like extracellular matrix protein 1	0.19	NA	NA	2	3.9
P15144	Aminopeptidase N	0.19	NA	NA	2	2.6
Q9BXN1	Asporin	0.19	NA	NA	2	2.9
P80723	Brain acid soluble protein 1	0.19	0.177	3.2E-01	3	18.1
Q14767	Latent-transforming growth factor beta-binding protein 2	0.19	NA	NA	2	1.6
P03973	Antileukoproteinase	0.17	NA	NA	2	15.9
P26447	Protein S100-A4	0.16	NA	NA	2	18.8
P24844	Myosin regulatory light polypeptide 9	0.16	NA	NA	2	12.2
Q2UY09	Collagen alpha-1(XXVIII) chain	0.14	NA	NA	2	1.8
P63211	Guanine nucleotide-binding protein G(T) subunit gamma-T1	0.13	NA	NA	2	20.3
P22352	Glutathione peroxidase 3	0.12	NA	NA	2	8.0
P08123	Collagen alpha-2(I) chain	0.10	NA	NA	2	2.4
O43301	Heat shock 70 kDa protein 12A	0.45	0.060	4.0E-04	6	10.7
Q15019	Septin-2	0.45	0.072	7.8E-04	6	20.8
P00387	NADH-cytochrome b5 reductase 3	0.45	0.165	1.7E-02	7	24.6
P05556	Integrin beta-1	0.45	0.071	5.8E-06	6	8.0
P06396	Gelsolin	0.44	0.097	5.1E-06	11	17.3
O75369	Filamin-B	0.44	0.112	1.9E-03	8	5.0
Q9BTV4	Transmembrane protein 43	0.43	0.116	5.7E-03	3	8.8
P67936	Tropomyosin alpha-4 chain	0.43	0.150	1.1E-03	8	25.0
P18206	Vinculin	0.42	0.090	8.2E-07	14	15.3
Q9BSJ8	Extended synaptotagmin-1	0.41	0.194	1.3E-02	5	5.8
P46821	Microtubule-associated protein 1B	0.41	0.145	3.6E-02	3	1.3
Q9NZN4	EH domain-containing protein 2	0.41	0.062	2.7E-03	3	5.7
P00450	Ceruloplasmin	0.41	0.066	3.0E-08	9	11.2
P07355	Annexin A2	0.40	0.039	0.0E+00	24	54.6
P35579	Myosin-9	0.40	0.036	0.0E+00	51	26.7
P60660	Myosin light polypeptide 6	0.40	0.031	0.0E+00	6	47.7
P11166	Solute carrier family 2, facilitated glucose transporter member 1	0.39	0.088	2.8E-05	3	5.5
P01011	Alpha-1-antichymotrypsin	0.37	0.097	9.8E-05	7	18.4
P22413	Ectonucleotide pyrophosphatase/phosphodiesterase family member 1	0.36	0.088	3.4E-02	3	4.4
P21333	Filamin-A	0.35	0.037	0.0E+00	45	23.3
P00167	Cytochrome b5	0.34	0.089	1.1E-04	4	42.5
P07099	Epoxide hydrolase 1	0.34	0.063	3.2E-06	4	9.0

P12111	Collagen alpha-3(VI) chain	0.34	0.040	0.0E+00	32	11.1
Q16555	Dihydropyrimidinase-related protein 2	0.34	0.073	1.4E-10	10	21.0
Q16363	Laminin subunit alpha-4	0.33	0.096	1.2E-02	3	2.1
P09493	Tropomyosin alpha-1 chain	0.32	0.194	1.8E-04	5	14.8
Q9NZM1	Myoferlin	0.32	0.091	9.7E-06	5	3.0
Q02952	A-kinase anchor protein 12	0.31	0.110	1.3E-04	14	11.1
O94911	ATP-binding cassette sub-family A member 8	0.31	0.052	3.9E-05	4	2.8
Q9Y6C2	EMILIN-1	0.31	0.134	2.3E-04	7	9.2
P08572	Collagen alpha-2(IV) chain	0.31	0.150	8.3E-04	7	5.4
P02549	Spectrin alpha chain, erythrocytic 1	0.31	0.174	1.3E-04	7	4.1
P04196	Histidine-rich glycoprotein	0.31	0.102	1.3E-03	3	5.3
P12109	Collagen alpha-1(VI) chain	0.30	0.063	3.9E-06	9	9.5
P02679	Fibrinogen gamma chain	0.30	0.189	2.4E-03	7	19.2
P36269	Gamma-glutamyltransferase 5	0.29	0.168	3.2E-03	6	12.8
P60903	Protein S100-A10	0.29	0.096	1.2E-04	4	35.1
P12110	Collagen alpha-2(VI) chain	0.28	0.095	3.5E-08	8	8.5
O43491	Band 4.1-like protein 2	0.28	0.064	2.7E-05	6	8.1
P02751	Fibronectin	0.28	0.098	8.2E-07	8	4.5
P08294	Extracellular superoxide dismutase [Cu-Zn]	0.27	0.111	2.8E-04	3	15.4
P55268	Laminin subunit beta-2	0.27	0.114	5.9E-06	11	6.4
Q05707	Collagen alpha-1(XIV) chain	0.27	0.102	1.2E-03	6	3.8
O94875	Sorbin and SH3 domain-containing protein 2	0.27	0.178	8.0E-03	4	5.5
P50895	Basal cell adhesion molecule	0.27	0.127	4.5E-04	4	8.8
P07942	Laminin subunit beta-1	0.26	0.185	6.7E-03	4	2.6
P02675	Fibrinogen beta chain	0.26	0.086	7.2E-06	9	23.4
P27105	Erythrocyte band 7 integral membrane protein	0.25	0.077	5.5E-06	7	22.9
P02760	Protein AMBP	0.25	0.136	1.5E-04	5	22.2
Q14112	Nidogen-2	0.25	0.137	1.8E-05	7	4.9
P39059	Collagen alpha-1(XV) chain	0.25	0.166	2.9E-04	5	3.9
P68032	Actin, alpha cardiac muscle 1	0.24	0.142	6.0E-06	6	21.0
P02649	Apolipoprotein E	0.24	0.064	3.3E-11	15	45.1
P09936	Ubiquitin carboxyl-terminal hydrolase isozyme L1	0.24	0.296	3.8E-02	4	21.5
P98160	Basement membrane-specific heparan sulfate proteoglycan core protein	0.23	0.068	2.4E-15	27	7.6
P02686	Myelin basic protein	0.23	0.087	9.0E-07	3	11.2
O15230	Laminin subunit alpha-5	0.23	0.126	1.8E-03	10	3.8
P02671	Fibrinogen alpha chain	0.22	0.076	1.6E-08	9	13.0
P01871	Ig mu chain C region	0.21	0.135	1.0E-04	7	19.5
P11277	Spectrin beta chain, erythrocytic	0.21	0.198	1.8E-02	9	5.6
P21926	CD9 antigen	0.21	0.135	9.4E-03	3	9.6
P04083	Annexin A1	0.21	0.079	2.8E-11	11	39.0
P01008	Antithrombin-III	0.21	0.142	1.5E-04	5	12.1
P11047	Laminin subunit gamma-1	0.21	0.123	1.6E-07	13	8.3
Q14195	Dihydropyrimidinase-related protein 3	0.19	0.109	3.2E-08	9	21.6
Q14699	Raftlin	0.19	0.169	3.8E-02	3	4.0
P39060	Collagen alpha-1(XVIII) chain	0.18	0.103	7.8E-09	7	4.1
P05186	Alkaline phosphatase, tissue-nonspecific isozyme	0.18	0.165	1.8E-02	4	8.2
P04275	von Willebrand factor	0.18	0.087	2.4E-10	10	4.0
P10745	Retinol-binding protein 3	0.17	0.108	1.5E-05	5	5.0
P20774	Mimecan	0.17	0.092	1.4E-09	8	22.5
Q9BXM0	Periaxin	0.16	0.173	1.8E-05	7	3.5
P14543	Nidogen-1	0.16	0.097	6.8E-08	8	7.0
P02730	Band 3 anion transport protein	0.16	0.128	4.1E-04	6	8.2
P35749	Myosin-11	0.16	0.068	1.6E-13	27	16.0
P21980	Protein-glutamine gamma-glutamyltransferase 2	0.16	0.083	0.0E+00	15	21.4
P22748	Carbonic anhydrase 4	0.15	0.098	4.3E-07	8	26.9
P35555	Fibrillin-1	0.15	0.042	0.0E+00	38	14.6
P35625	Metalloproteinase inhibitor 3	0.15	0.118	1.0E-07	5	24.6
P41219	Peripherin	0.15	0.105	7.9E-11	13	30.9
P01031	Complement C5	0.14	0.133	1.9E-05	5	3.0
P07585	Decorin	0.14	0.099	7.6E-05	6	16.7
Q15661	Tryptase alpha/beta-1	0.14	0.087	2.2E-11	5	21.8
P10909	Clusterin	0.14	0.062	0.0E+00	14	27.4
P51888	Prolargin	0.14	0.054	0.0E+00	12	34.0
P51884	Lumican	0.14	0.052	0.0E+00	9	26.0
P21810	Biglycan	0.13	0.094	1.2E-11	8	23.9
P15088	Mast cell carboxypeptidase A	0.13	0.198	2.1E-03	3	6.0
P04004	Vitronectin	0.13	0.071	0.0E+00	11	20.3
P02743	Serum amyloid P-component	0.12	0.100	1.3E-05	6	23.3
P02748	Complement component C9	0.12	0.084	6.6E-08	8	15.2
P25189	Myelin protein P0	0.10	0.092	6.5E-09	6	24.2

Brown denotes change ≥ 2 standard deviations (SD) from the mean, yellow denotes change ≥ 1 SD and green highlights p values ≤ 0.05. NA, not applicable, n<3 unique peptides.
