# Supplementary material for: iTRAQ Quantitative Proteomic Comparison of Metastatic and Non-Metastatic Uveal Melanoma Tumors
Source: PLoS One. 2015 Aug 25;10(8):e0135543. doi: 10.1371/journal.pone.0135543 (PMC4549237; doi:10.1371/journal.pone.0135543)
Supplement: S10 Table — (PDF) [file pone.0135543.s010.pdf]

Supplementary Table S10
Relative Protein Abundance: Sample UM26, Non-Metastatic
Total Proteins Quantified = 797; LogMedian Protein Ratio = 0.15; LogMean Protein Ratio = 0; Standard Deviation = 0.84

Uni-Prot Accession	Protein	Ratio UM/Control	Standard Deviation	*p* value	Unique Peptides	% Sequence Coverage
Q9UBR2	Cathepsin Z	7.84	0.169	4.6E-06	4	10.6
Q13510	Acid ceramidase	7.56	0.103	1.1E-13	10	18.2
Q9H3G5	Probable serine carboxypeptidase CPVL	7.17	0.101	1.2E-09	6	11.8
P30086	Phosphatidylethanolamine-binding protein 1	6.95	0.126	1.4E-09	10	58.8
P40121	Macrophage-capping protein	6.78	0.197	9.1E-07	5	15.5
Q8IV08	Phospholipase D3	6.68	0.124	2.2E-09	5	10.0
P09211	Glutathione S-transferase P	5.76	0.095	5.1E-09	6	39.0
P07108	Acyl-CoA-binding protein	5.48	0.265	7.0E-04	3	52.9
P63241	Eukaryotic translation initiation factor 5A-1	5.47	0.194	4.7E-04	5	27.9
P62937	Peptidyl-prolyl cis-trans isomerase A	5.36	0.090	0.0E+00	6	29.7
P07195	L-lactate dehydrogenase B chain	5.10	0.111	1.3E-12	10	29.3
P40925	Malate dehydrogenase, cytoplasmic	5.09	0.208	1.6E-05	5	17.4
P60174	Triosephosphate isomerase	4.78	0.143	1.4E-04	11	42.7
P78417	Glutathione S-transferase omega-1	4.33	0.104	2.8E-06	7	26.6
P04080	Cystatin-B	4.09	0.138	4.1E-06	5	57.1
P07339	Cathepsin D	4.08	0.091	1.2E-10	7	18.0
P06733	Alpha-enolase	3.83	0.067	8.5E-12	11	28.3
P15121	Aldose reductase	3.66	0.121	2.4E-05	5	16.5
P04406	Glyceraldehyde-3-phosphate dehydrogenase	3.65	0.065	3.3E-15	9	28.1
P31939	Bifunctional purine biosynthesis protein PURH	3.59	0.118	7.5E-03	3	5.2
Q9HC38	Glyoxalase domain-containing protein 4	3.55	0.174	3.6E-03	4	13.1
P06865	Beta-hexosaminidase subunit alpha	3.54	0.135	2.0E-04	6	9.6
P07858	Cathepsin B	3.53	0.133	4.2E-08	7	22.1
P08758	Annexin A5	3.51	0.042	0.0E+00	18	59.7
Q9UHL4	Dipeptidyl peptidase 2	3.51	0.336	1.1E-02	6	15.0
P61916	Epididymal secretory protein E1	3.39	0.149	2.3E-02	4	23.2
P07686	Beta-hexosaminidase subunit beta	3.34	0.247	1.1E-03	10	16.5
P23528	Cofilin-1	3.32	0.153	4.8E-04	7	37.3
Q16658	Fascin	3.30	0.214	5.7E-03	4	10.1
P52565	Rho GDP-dissociation inhibitor 1	3.28	0.131	8.9E-06	4	22.5
P06737	Glycogen phosphorylase, liver form	3.28	0.088	1.1E-14	20	24.3
P02768	Serum albumin	3.26	0.026	0.0E+00	36	54.2
P27816	Microtubule-associated protein 4	3.19	0.205	4.2E-04	8	10.2
O75368	SH3 domain-binding glutamic acid-rich-like protein	3.16	0.142	3.5E-03	3	34.2
P08195	4F2 cell-surface antigen heavy chain	3.13	0.084	3.2E-09	10	17.5
P51858	Hepatoma-derived growth factor	3.08	0.182	4.3E-03	4	19.2
Q01105	Protein SET	3.01	0.142	1.0E-04	5	20.0
P10253	Lysosomal alpha-glucosidase	2.96	0.214	9.6E-03	3	3.3
P07900	Heat shock protein HSP 90-alpha	2.95	0.085	8.4E-12	15	17.6
P29401	Transketolase	2.93	0.148	1.7E-04	9	14.1
P00338	L-lactate dehydrogenase A chain	2.92	0.122	7.8E-06	6	15.1
P00558	Phosphoglycerate kinase 1	2.92	0.109	2.4E-07	11	21.1
P07602	Prosaposin	2.90	0.097	1.7E-04	6	9.4
P46926	Glucosamine-6-phosphate isomerase 1	2.87	0.222	2.3E-03	4	12.5
P57729	Ras-related protein Rab-38	2.87	0.141	1.2E-03	3	11.8
P19338	Nucleolin	2.86	0.101	5.4E-09	12	14.6
P06748	Nucleophosmin	2.81	0.109	1.5E-05	7	19.7
P27348	14-3-3 protein theta	2.81	0.044	5.8E-05	4	21.6
P06744	Glucose-6-phosphate isomerase	2.80	0.162	3.0E-05	9	16.1
P63104	14-3-3 protein zeta/delta	2.73	0.089	7.8E-05	6	29.8
Q00796	Sorbitol dehydrogenase	2.70	0.088	3.8E-04	5	12.0
P62158	Calmodulin	2.67	0.118	5.2E-05	4	29.5
P24941	Cyclin-dependent kinase 2	2.64	0.284	1.2E-02	4	13.4
P51149	Ras-related protein Rab-7a	2.62	0.067	1.9E-09	8	42.5
P02787	Serotransferrin	2.60	0.089	1.2E-09	17	24.1
P15531	Nucleoside diphosphate kinase A	2.57	0.234	1.6E-02	3	22.4
Q99536	Synaptic vesicle membrane protein VAT-1 homolog	2.50	0.090	2.0E-05	11	33.3
P10599	Thioredoxin	2.50	0.096	1.2E-02	3	32.4
P23526	Adenosylhomocysteinase	2.48	0.133	1.3E-04	9	24.5
P14618	Pyruvate kinase PKM	2.46	0.052	4.2E-15	11	23.2
P07737	Profilin-1	2.45	0.106	1.6E-06	5	34.3
P13798	Acylamino-acid-releasing enzyme	2.44	0.124	3.9E-03	3	4.1
O14818	Proteasome subunit alpha type-7	2.43	0.175	1.5E-02	3	11.7
P25786	Proteasome subunit alpha type-1	2.39	0.211	1.5E-02	4	15.6
P38606	V-type proton ATPase catalytic subunit A	2.37	0.068	5.8E-04	7	11.8
P16070	CD44 antigen	2.36	0.135	6.0E-03	9	12.1
P62258	14-3-3 protein epsilon	2.36	0.170	5.8E-04	9	26.7
Q9Y2J2	Band 4.1-like protein 3	2.34	0.165	1.0E-02	6	8.8
P10619	Lysosomal protective protein	2.33	0.096	2.4E-04	4	8.3
P06454	Prothymosin alpha	5.53	NA	NA	2	12.6
P61088	Ubiquitin-conjugating enzyme E2 N	5.32	NA	NA	2	13.8
Q8WWM9	Cytoglobin	5.02	NA	NA	2	8.4
P05413	Fatty acid-binding protein, heart	4.91	NA	NA	2	15.8
O14556	Glyceraldehyde-3-phosphate dehydrogenase, testis-specific	4.81	NA	NA	2	5.4
Q99572	P2X purinoceptor 7	3.85	NA	NA	2	4.2
O75347	Tubulin-specific chaperone A	3.73	NA	NA	2	16.7
P43235	Cathepsin K	3.70	NA	NA	2	4.6
P17096	High mobility group protein HMG-I/HMG-Y	3.62	NA	NA	2	23.4
Q04760	Lactoylglutathione lyase	3.61	NA	NA	2	10.3
O00584	Ribonuclease T2	3.40	NA	NA	2	6.6
P07741	Adenine phosphoribosyltransferase	3.24	NA	NA	2	12.8
P30043	Flavin reductase (NADPH)	3.11	NA	NA	2	11.7
P62826	GTP-binding nuclear protein Ran	3.09	NA	NA	2	9.7
P13686	Tartrate-resistant acid phosphatase type 5	2.97	NA	NA	2	5.8
P17900	Ganglioside GM2 activator	2.93	0.369	7.5E-02	3	13.0
P58546	Myotrophin	2.91	NA	NA	2	25.4
Q9UHB6	LIM domain and actin-binding protein 1	2.87	NA	NA	2	4.0
P20039	HLA class II histocompatibility antigen, DRB1-11 beta chain	2.76	NA	NA	2	9.8
Q15819	Ubiquitin-conjugating enzyme E2 variant 2	2.74	NA	NA	2	13.1
P21283	V-type proton ATPase subunit C 1	2.73	NA	NA	2	4.2
P11766	Alcohol dehydrogenase class-3	2.68	NA	NA	2	5.1
P09429	High mobility group protein B1	2.45	0.137	1.1E-01	4	21.9
Q96A05	V-type proton ATPase subunit E 2	2.37	NA	NA	2	7.1
O15400	Syntaxin-7	2.36	NA	NA	2	10.7
Q9Y2S2	Lambda-crystallin homolog	2.32	NA	NA	2	6.3
P13639	Elongation factor 2	2.30	0.121	6.7E-05	8	10.4
P22087	rRNA 2'-O-methyltransferase fibrillarin	2.29	NA	NA	2	7.5
P12955	Xaa-Pro dipeptidase	2.29	0.209	1.8E-02	3	5.7
O75083	WD repeat-containing protein 1	2.26	0.104	3.3E-06	7	11.2
P23246	Splicing factor, proline- and glutamine-rich	2.26	0.250	1.0E-02	5	8.6

Q15631	Translin	2.25	NA	NA	2	11.4
Q12906	Interleukin enhancer-binding factor 3	2.23	0.118	9.8E-04	11	14.5
Q13185	Chromobox protein homolog 3	2.23	0.086	1.8E-06	3	18.0
Q99497	Protein DJ-1	2.21	0.176	1.9E-02	5	28.0
O75874	Isocitrate dehydrogenase [NADP] cytoplasmic	2.21	0.230	1.2E-01	3	8.0
P08670	Vimentin	2.17	0.037	0.0E+00	30	60.1
P08238	Heat shock protein HSP 90-beta	2.14	0.107	4.1E-05	7	9.3
P09382	Galectin-1	2.14	0.058	7.4E-09	6	46.7
O43399	Tumor protein D54	2.11	NA	NA	2	11.7
P16152	Carbonyl reductase [NADPH] 1	2.10	0.070	1.4E-02	3	13.4
O00483	NADH dehydrogenase [ubiquinone] 1 alpha subcomplex subunit 4	2.10	0.185	1.0E-02	3	32.1
P06753	Tropomyosin alpha-3 chain	2.09	0.089	1.4E-05	4	13.0
P09936	Ubiquitin carboxyl-terminal hydrolase isozyme L1	2.08	0.089	3.3E-06	4	25.1
P13489	Ribonuclease inhibitor	2.06	0.182	5.3E-03	5	12.6
P40926	Malate dehydrogenase, mitochondrial	2.06	0.066	1.1E-09	8	26.0
P31948	Stress-induced-phosphoprotein 1	2.06	0.117	3.6E-04	8	13.1
P60842	Eukaryotic initiation factor 4A-I	2.06	0.115	1.5E-03	4	12.3
Q07955	Serine/arginine-rich splicing factor 1	2.06	0.063	4.7E-05	5	17.3
P07910	Heterogeneous nuclear ribonucleoproteins C1/C2	2.05	0.061	6.8E-09	9	27.1
Q12905	Interleukin enhancer-binding factor 2	2.05	0.037	3.1E-05	3	9.5
O14773	Tripeptidyl-peptidase 1	2.04	0.102	3.6E-05	3	6.2
P46940	Ras GTPase-activating-like protein IQGAP1	2.03	0.042	7.3E-12	14	9.4
Q13838	Spliceosome RNA helicase DDX39B	2.03	0.136	3.6E-03	4	9.8
P39687	Acidic leucine-rich nuclear phosphoprotein 32 family member A	2.03	0.208	2.6E-02	4	10.0
P55786	Puromycin-sensitive aminopeptidase	2.03	0.108	7.4E-04	4	4.9
Q13177	Serine/threonine-protein kinase PAK 2	2.02	NA	NA	2	5.9
Q9UH65	Switch-associated protein 70	2.01	0.061	6.1E-05	4	4.8
Q5VTE0	Putative elongation factor 1-alpha-like 3	2.00	0.059	8.9E-08	9	17.1
P20618	Proteasome subunit beta type-1	2.00	0.111	1.0E-03	3	13.3
Q07960	Rho GTPase-activating protein 1	2.00	NA	NA	2	5.0
P15586	N-acetylglucosamine-6-sulfatase	1.99	0.209	6.1E-02	3	4.7
P21796	Voltage-dependent anion-selective channel protein 1	1.97	0.082	8.8E-06	6	19.8
P54652	Heat shock-related 70 kDa protein 2	1.97	0.093	3.1E-06	10	15.6
P99999	Cytochrome c	1.96	0.193	8.6E-03	3	24.8
Q14103	Heterogeneous nuclear ribonucleoprotein D0	1.95	NA	NA	2	6.8
P09651	Heterogeneous nuclear ribonucleoprotein A1	1.95	0.062	3.0E-06	8	23.9
Q92945	Far upstream element-binding protein 2	1.95	0.119	7.9E-04	4	6.2
P30044	Peroxiredoxin-5, mitochondrial	1.94	0.072	3.4E-04	3	15.4
Q14247	Src substrate cortactin	1.92	0.709	1.6E-01	4	8.9
P53999	Activated RNA polymerase II transcriptional coactivator p15	1.90	NA	NA	2	15.7
Q04917	14-3-3 protein eta	1.89	NA	NA	2	9.8
Q13126	S-methyl-5'-thioadenosine phosphorylase	1.89	NA	NA	2	7.4
O00567	Nucleolar protein 56	1.88	NA	NA	2	2.9
P40967	Melanocyte protein PMEL	1.87	NA	NA	2	3.3
Q96FW1	Ubiquitin thioesterase OTUB1	1.86	NA	NA	2	10.3
Q9Y3C8	Ubiquitin-fold modifier-conjugating enzyme 1	1.86	NA	NA	2	11.4
P61604	10 kDa heat shock protein, mitochondrial	1.84	0.081	1.1E-02	4	35.3
Q13619	Cullin-4A	1.84	NA	NA	2	2.2
Q08380	Galectin-3-binding protein	1.84	0.208	3.9E-02	4	8.5
O95834	Echinoderm microtubule-associated protein-like 2	1.81	NA	NA	2	4.0
Q16666	Gamma-interferon-inducible protein 16	1.81	NA	NA	2	2.5
Q01130	Serine/arginine-rich splicing factor 2	1.81	0.106	1.2E-01	3	14.5
Q14683	Structural maintenance of chromosomes protein 1A	1.80	NA	NA	2	1.7
P18669	Phosphoglycerate mutase 1	1.80	0.081	2.2E-05	3	21.7
P51159	Ras-related protein Rab-27A	1.78	NA	NA	2	10.9
P16401	Histone H1.5	1.78	0.170	8.7E-03	3	9.7
P07203	Glutathione peroxidase 1	1.78	0.242	1.5E-01	3	10.3
P25398	40S ribosomal protein S12	1.78	NA	NA	2	13.6
B5ME19	Eukaryotic translation initiation factor 3 subunit C-like protein	1.76	0.315	2.0E-01	3	3.2
Q96KP4	Cytosolic non-specific dipeptidase	1.75	0.132	4.4E-03	5	13.9
Q9UKM9	RNA-binding protein Raly	1.75	0.117	3.3E-03	4	17.0
P20702	Integrin alpha-X	1.74	0.128	2.1E-02	4	3.4
P10155	60 kDa SS-A/Ro ribonucleoprotein	1.73	NA	NA	2	4.5
P17931	Galectin-3	1.72	0.113	2.6E-04	6	28.4
Q14956	Transmembrane glycoprotein NMB	1.72	0.185	5.1E-02	5	10.0
Q92688	Acidic leucine-rich nuclear phosphoprotein 32 family member B	1.72	0.289	9.4E-02	5	14.3
Q15365	Poly(rC)-binding protein 1	1.72	0.331	1.3E-01	3	10.1
P37840	Alpha-synuclein	1.72	NA	NA	2	17.1
Q99523	Sortilin	1.71	NA	NA	2	2.4
P13693	Translationally-controlled tumor protein	1.70	NA	NA	2	15.7
P20042	Eukaryotic translation initiation factor 2 subunit 2	1.70	0.427	6.4E-01	3	11.4
P28066	Proteasome subunit alpha type-5	1.70	NA	NA	2	12.9
P17643	5,6-dihydroxyindole-2-carboxylic acid oxidase	1.70	0.124	3.4E-03	6	13.0
O43681	ATPase ASNA1	1.70	NA	NA	2	5.5
P55809	Succinyl-CoA:3-ketoacid coenzyme A transferase 1, mitochondrial	1.69	NA	NA	2	3.5
Q15691	Microtubule-associated protein RP/EB family member 1	1.69	0.095	1.8E-02	4	8.6
P78347	General transcription factor II-I	1.69	0.423	1.9E-01	3	2.9
P31040	Succinate dehydrogenase [ubiquinone] flavoprotein subunit, mitochondrial	1.67	0.136	4.3E-03	5	10.7
P61353	60S ribosomal protein L27	1.67	0.082	1.2E-02	3	25.7
P62263	40S ribosomal protein S14	1.67	0.198	1.4E-01	4	36.4
P02786	Transferrin receptor protein 1	1.66	NA	NA	2	3.2
P61981	14-3-3 protein gamma	1.66	0.145	1.9E-02	3	13.8
O75380	NADH dehydrogenase [ubiquinone] iron-sulfur protein 6, mitochondrial	1.65	NA	NA	2	20.2
O95292	Vesicle-associated membrane protein-associated protein B/C	1.65	0.156	1.7E-01	3	14.4
P14854	Cytochrome c oxidase subunit 6B1	1.65	0.031	1.5E-03	3	24.4
P08865	40S ribosomal protein SA	1.64	0.051	1.0E-05	5	19.7
P30041	Peroxiredoxin-6	1.64	0.071	8.4E-06	7	25.4
Q15102	Platelet-activating factor acetylhydrolase IB subunit gamma	1.64	NA	NA	2	9.1
P02790	Hemopexin	1.64	0.058	5.3E-04	3	6.3
P22314	Ubiquitin-like modifier-activating enzyme 1	1.64	0.163	1.6E-02	5	5.5
P15311	Ezrin	1.63	NA	NA	2	3.8
P26599	Polypyrimidine tract-binding protein 1	1.63	0.087	3.3E-04	5	7.7
P30084	Enoyl-CoA hydratase, mitochondrial	1.62	0.085	6.5E-02	3	12.8
O14979	Heterogeneous nuclear ribonucleoprotein D-like	1.62	0.170	2.1E-02	3	4.5
P20674	Cytochrome c oxidase subunit 5A, mitochondrial	1.62	0.262	9.0E-02	4	20.0
Q15056	Eukaryotic translation initiation factor 4H	1.62	NA	NA	2	10.9
P50395	Rab GDP dissociation inhibitor beta	1.61	0.182	1.2E-01	4	10.8
P42704	Leucine-rich PPR motif-containing protein, mitochondrial	1.61	0.098	1.1E-02	6	3.9
P54819	Adenylate kinase 2, mitochondrial	1.61	NA	NA	2	10.9
P55084	Trifunctional enzyme subunit beta, mitochondrial	1.60	0.037	7.3E-11	12	21.1
P62906	60S ribosomal protein L10a	1.60	0.086	9.6E-04	5	27.2
P62820	Ras-related protein Rab-1A	1.60	NA	NA	2	8.3
Q99798	Aconitate hydratase, mitochondrial	1.59	0.106	1.3E-04	9	15.0
P02765	Alpha-2-HS-glycoprotein	1.59	NA	NA	2	3.5
Q13576	Ras GTPase-activating-like protein IQGAP2	1.58	0.244	1.4E-01	5	3.3
P46439	Glutathione S-transferase Mu 5	1.58	NA	NA	2	6.9
P31949	Protein S100-A11	1.57	0.267	1.5E-01	3	27.6

P22626	Heterogeneous nuclear ribonucleoproteins A2/B1	1.57	0.048	1.9E-08	14	35.4
Q01469	Fatty acid-binding protein, epidermal	1.57	NA	NA	2	11.9
Q9H2U2	Inorganic pyrophosphatase 2, mitochondrial	1.56	NA	NA	2	6.3
P05198	Eukaryotic translation initiation factor 2 subunit 1	1.56	NA	NA	2	7.0
P05107	Integrin beta-2	1.56	0.078	1.6E-03	5	7.5
P60981	Destrin	1.56	0.129	4.7E-02	3	18.2
P61158	Actin-related protein 3	1.56	0.091	7.4E-03	6	17.2
P12956	X-ray repair cross-complementing protein 6	1.56	0.099	2.6E-04	11	19.9
P37802	Transgelin-2	1.56	NA	NA	2	10.1
Q13011	Delta(3,5)-Delta(2,4)-dienoyl-CoA isomerase, mitochondrial	1.55	0.096	5.9E-02	4	12.5
Q09028	Histone-binding protein RBBP4	1.55	NA	NA	2	3.5
P01903	HLA class II histocompatibility antigen, DR alpha chain	1.55	0.090	1.0E-03	4	21.3
P13010	X-ray repair cross-complementing protein 5	1.54	0.123	4.2E-03	10	13.8
P52815	39S ribosomal protein L12, mitochondrial	1.54	NA	NA	2	11.6
P37837	Transaldolase	1.54	0.116	4.7E-02	5	11.9
P02766	Transthyretin	1.54	NA	NA	2	12.9
P14868	Aspartate--tRNA ligase, cytoplasmic	1.53	0.334	2.0E-01	3	7.6
Q14974	Importin subunit beta-1	1.53	0.960	4.0E-01	3	5.4
P34932	Heat shock 70 kDa protein 4	1.53	0.049	2.8E-05	8	10.7
P46777	60S ribosomal protein L5	1.53	0.087	4.1E-04	4	14.5
P62277	40S ribosomal protein S13	1.52	0.394	3.7E-02	7	37.1
P09012	U1 small nuclear ribonucleoprotein A	1.52	NA	NA	2	6.4
Q15084	Protein disulfide-isomerase A6	1.52	0.064	5.0E-05	5	14.3
P55008	Allograft inflammatory factor 1	1.51	NA	NA	2	11.6
Q13435	Splicing factor 3B subunit 2	1.51	NA	NA	2	3.4
Q16531	DNA damage-binding protein 1	1.51	0.047	6.2E-03	3	2.4
P63244	Guanine nucleotide-binding protein subunit beta-2-like 1	1.50	NA	NA	2	7.3
P26641	Elongation factor 1-gamma	1.49	0.129	1.1E-02	4	8.9
P09669	Cytochrome c oxidase subunit 6C	1.49	0.110	9.4E-02	4	33.3
P30405	Peptidyl-prolyl cis-trans isomerase F, mitochondrial	1.48	NA	NA	2	7.2
O95336	6-phosphogluconolactonase	1.48	0.314	3.8E-01	4	20.9
P61978	Heterogeneous nuclear ribonucleoprotein K	1.48	0.069	7.3E-04	13	29.4
Q06136	3-ketodihydrosphingosine reductase	1.48	NA	NA	2	8.1
P39019	40S ribosomal protein S19	1.48	0.042	1.4E-05	4	24.1
Q86UX7	Fermitin family homolog 3	1.48	NA	NA	2	3.9
P51991	Heterogeneous nuclear ribonucleoprotein A3	1.48	0.058	1.6E-05	6	17.7
Q5JTV8	Torsin-1A-interacting protein 1	1.48	NA	NA	2	4.3
O94832	Unconventional myosin-Id	1.48	0.146	5.8E-03	8	8.4
Q86TX2	Acyl-coenzyme A thioesterase 1	1.48	NA	NA	2	5.2
Q8NHP8	Putative phospholipase B-like 2	1.47	NA	NA	2	2.4
Q15717	ELAV-like protein 1	1.47	NA	NA	2	7.7
Q99436	Proteasome subunit beta type-7	1.47	NA	NA	2	7.2
Q07666	KH domain-containing, RNA-binding, signal transduction-associated protein 1	1.46	0.066	4.5E-03	4	7.9
P78527	DNA-dependent protein kinase catalytic subunit	1.46	0.082	8.4E-05	13	3.1
P02652	Apolipoprotein A-II	1.46	0.328	1.8E-01	3	19.0
Q9NP81	Serine--tRNA ligase, mitochondrial	1.46	NA	NA	2	6.9
P51810	G-protein coupled receptor 143	1.45	NA	NA	2	5.7
O75489	NADH dehydrogenase [ubiquinone] iron-sulfur protein 3, mitochondrial	1.45	0.077	8.7E-03	4	18.9
P49591	Serine--tRNA ligase, cytoplasmic	1.44	NA	NA	2	5.3
P62249	40S ribosomal protein S16	1.44	NA	NA	2	15.8
Q08211	ATP-dependent RNA helicase A	1.44	0.188	1.5E-02	7	6.8
P00441	Superoxide dismutase [Cu-Zn]	1.43	NA	NA	2	13.0
P09622	Dihydrolipoyl dehydrogenase, mitochondrial	1.43	0.263	3.7E-02	6	12.8
P49411	Elongation factor Tu, mitochondrial	1.43	0.064	3.3E-05	11	26.3
Q6UVK1	Chondroitin sulfate proteoglycan 4	1.43	0.244	1.1E-01	5	3.1
P21266	Glutathione S-transferase Mu 3	1.43	NA	NA	2	9.3
Q06323	Proteasome activator complex subunit 1	1.43	0.145	6.3E-02	3	14.5
P19404	NADH dehydrogenase [ubiquinone] flavoprotein 2, mitochondrial	1.43	NA	NA	2	9.6
O95747	Serine/threonine-protein kinase OSR1	1.42	NA	NA	2	3.6
P53634	Dipeptidyl peptidase 1	1.42	NA	NA	2	3.5
P20340	Ras-related protein Rab-6A	1.42	NA	NA	2	11.1
P52272	Heterogeneous nuclear ribonucleoprotein M	1.42	0.114	8.8E-02	5	8.9
Q02318	Sterol 26-hydroxylase, mitochondrial	1.42	0.149	2.9E-01	3	7.5
Q9UJU6	Drebrin-like protein	1.41	NA	NA	2	5.1
P08237	ATP-dependent 6-phosphofructokinase, muscle type	1.41	0.074	1.2E-01	4	6.2
P54727	UV excision repair protein RAD23 homolog B	1.41	0.241	1.8E-01	3	5.4
P26006	Integrin alpha-3	1.41	NA	NA	2	1.8
Q13263	Transcription intermediary factor 1-beta	1.40	NA	NA	2	3.7
P02647	Apolipoprotein A-I	1.40	0.077	1.6E-03	9	30.7
P23284	Peptidyl-prolyl cis-trans isomerase B	1.40	0.053	2.0E-05	10	38.9
P01859	Ig gamma-2 chain C region	1.39	NA	NA	2	6.7
O15145	Actin-related protein 2/3 complex subunit 3	1.39	0.094	1.8E-02	4	19.7
O00560	Syntenin-1	1.39	0.658	4.9E-01	3	9.1
P26373	60S ribosomal protein L13	1.39	0.071	1.5E-02	4	19.4
P04075	Fructose-bisphosphate aldolase A	1.39	0.095	2.6E-03	15	46.4
Q9BXK5	Bcl-2-like protein 13	1.39	NA	NA	2	4.7
P35232	Prohibitin	1.38	0.057	5.3E-05	7	26.8
Q1KMD3	Heterogeneous nuclear ribonucleoprotein U-like protein 2	1.38	NA	NA	2	3.1
Q9ULV4	Coronin-1C	1.38	NA	NA	2	5.3
Q14697	Neutral alpha-glucosidase AB	1.37	0.086	1.1E-03	12	11.9
P50914	60S ribosomal protein L14	1.37	0.035	3.2E-02	3	16.7
P60866	40S ribosomal protein S20	1.37	0.068	1.1E-02	3	26.9
O15144	Actin-related protein 2/3 complex subunit 2	1.37	NA	NA	2	6.0
P05387	60S acidic ribosomal protein P2	1.37	0.126	8.3E-02	4	60.0
Q9BR76	Coronin-1B	1.36	0.156	1.6E-01	3	5.5
Q13409	Cytoplasmic dynein 1 intermediate chain 2	1.36	NA	NA	2	3.9
Q96BM9	ADP-ribosylation factor-like protein 8A	1.36	NA	NA	2	12.4
P62829	60S ribosomal protein L23	1.36	NA	NA	2	16.4
Q15366	Poly(rC)-binding protein 2	1.36	NA	NA	2	10.1
Q9Y6M9	NADH dehydrogenase [ubiquinone] 1 beta subcomplex subunit 9	1.36	NA	NA	2	15.6
P21912	Succinate dehydrogenase [ubiquinone] iron-sulfur subunit, mitochondrial	1.36	0.028	4.1E-06	5	17.5
Q13242	Serine/arginine-rich splicing factor 9	1.36	NA	NA	2	9.5
P62424	60S ribosomal protein L7a	1.36	0.081	2.9E-02	5	16.2
P62913	60S ribosomal protein L11	1.35	NA	NA	2	12.9
Q96AG4	Leucine-rich repeat-containing protein 59	1.35	NA	NA	2	6.2
O60493	Sorting nexin-3	1.35	NA	NA	2	11.7
P10809	60 kDa heat shock protein, mitochondrial	1.35	0.168	1.4E-02	10	21.6
P62917	60S ribosomal protein L8	1.35	0.310	1.1E-01	3	13.2
P29966	Myristoylated alanine-rich C-kinase substrate	1.35	0.480	3.4E-01	5	25.3
P04792	Heat shock protein beta-1	1.34	0.131	9.6E-03	8	40.5
Q00839	Heterogeneous nuclear ribonucleoprotein U	1.34	0.124	2.8E-02	7	8.8
P17844	Probable ATP-dependent RNA helicase DDX5	1.34	0.145	1.1E-01	3	5.0
P13674	Prolyl 4-hydroxylase subunit alpha-1	1.34	NA	NA	2	5.1
P62979	Ubiquitin-40S ribosomal protein S27a	1.33	0.105	4.9E-03	8	41.7
Q00059	Transcription factor A, mitochondrial	1.33	NA	NA	2	11.0
Q8NFV4	Alpha/beta hydrolase domain-containing protein 11	1.33	NA	NA	2	8.6
P24534	Elongation factor 1-beta	1.33	NA	NA	2	7.1

A1L0T0	Acetolactate synthase-like protein	1.33	NA	NA	2	4.1
P62269	40S ribosomal protein S18	1.32	0.060	3.5E-03	4	22.4
P62280	40S ribosomal protein S11	1.32	0.046	4.0E-03	5	21.5
P10644	cAMP-dependent protein kinase type I-alpha regulatory subunit	1.32	0.528	3.8E-01	4	13.6
Q14847	LIM and SH3 domain protein 1	1.32	0.398	3.8E-01	4	18.4
P27797	Calreticulin	1.32	0.278	1.1E-01	9	24.2
P37108	Signal recognition particle 14 kDa protein	1.32	0.182	2.8E-01	3	22.1
Q99873	Protein arginine N-methyltransferase 1	1.32	NA	NA	2	7.5
P09525	Annexin A4	1.31	0.494	2.2E-02	7	16.6
P48047	ATP synthase subunit O, mitochondrial	1.31	0.155	8.6E-03	4	22.5
P47985	Cytochrome b-c1 complex subunit Rieske, mitochondrial	1.31	NA	NA	2	8.0
P30837	Aldehyde dehydrogenase X, mitochondrial	1.31	0.088	1.1E-02	5	14.1
P62851	40S ribosomal protein S25	1.31	0.052	4.5E-04	4	24.0
Q92841	Probable ATP-dependent RNA helicase DDX17	1.31	0.121	8.6E-02	4	7.4
O00231	26S proteasome non-ATPase regulatory subunit 11	1.31	0.046	1.2E-02	3	6.9
P12830	Cadherin-1	1.30	NA	NA	2	3.4
O75390	Citrate synthase, mitochondrial	1.30	0.046	9.3E-03	5	11.8
P21281	V-type proton ATPase subunit B, brain isoform	1.30	0.133	2.2E-02	4	9.8
P09661	U2 small nuclear ribonucleoprotein A'	1.30	NA	NA	2	8.6
P62318	Small nuclear ribonucleoprotein Sm D3	1.29	NA	NA	2	24.6
P0CW22	40S ribosomal protein S17-like	1.29	0.069	4.2E-03	3	16.3
P13804	Electron transfer flavoprotein subunit alpha, mitochondrial	1.29	NA	NA	2	8.1
P23396	40S ribosomal protein S3	1.29	0.058	5.4E-03	6	24.7
O75367	Core histone macro-H2A.1	1.29	0.823	8.3E-02	6	16.4
P50897	Palmitoyl-protein thioesterase 1	1.28	0.102	1.3E-01	3	10.5
Q13459	Unconventional myosin-IXb	1.28	NA	NA	2	1.1
O75165	DnaJ homolog subfamily C member 13	1.28	NA	NA	2	0.8
Q9UNH7	Sorting nexin-6	1.28	NA	NA	2	3.2
Q9BZZ5	Apoptosis inhibitor 5	1.28	0.696	4.9E-01	3	6.5
Q9Y4W6	AFG3-like protein 2	1.28	0.138	9.4E-02	5	7.5
P32969	60S ribosomal protein L9	1.28	NA	NA	2	5.7
O75396	Vesicle-trafficking protein SEC22b	1.28	NA	NA	2	12.6
Q9UJZ1	Stomatin-like protein 2, mitochondrial	1.27	NA	NA	2	8.7
P61026	Ras-related protein Rab-10	1.27	0.146	1.8E-01	4	18.5
O14737	Programmed cell death protein 5	1.27	NA	NA	2	17.6
Q3SY69	Mitochondrial 10-formyltetrahydrofolate dehydrogenase	1.27	NA	NA	2	2.3
O75306	NADH dehydrogenase [ubiquinone] iron-sulfur protein 2, mitochondrial	1.27	0.364	3.8E-01	3	6.7
Q9NTJ5	Phosphatidylinositide phosphatase SAC1	1.25	0.030	3.7E-02	3	5.8
O60486	Plexin-C1	1.25	NA	NA	2	1.4
P63000	Ras-related C3 botulinum toxin substrate 1	1.25	0.031	1.5E-04	5	29.2
P31146	Coronin-1A	1.25	NA	NA	2	4.6
P15880	40S ribosomal protein S2	1.25	0.046	5.5E-03	3	10.2
P62316	Small nuclear ribonucleoprotein Sm D2	1.25	NA	NA	2	16.1
P30049	ATP synthase subunit delta, mitochondrial	1.24	NA	NA	2	13.7
Q99623	Prohibitin-2	1.24	0.058	1.7E-02	8	29.4
P38646	Stress-70 protein, mitochondrial	1.24	0.178	1.4E-01	10	17.5
Q93050	V-type proton ATPase 116 kDa subunit a isoform 1	1.24	NA	NA	2	4.1
P00352	Retinal dehydrogenase 1	1.24	0.067	2.1E-02	8	22.0
P11142	Heat shock cognate 71 kDa protein	1.24	0.106	1.4E-02	10	16.6
Q13596	Sorting nexin-1	1.23	1.886	5.9E-01	3	7.7
Q16851	UTP--glucose-1-phosphate uridylyltransferase	1.23	0.034	3.3E-02	3	6.3
P43121	Cell surface glycoprotein MUC18	1.23	0.158	1.2E-01	5	11.6
P36578	60S ribosomal protein L4	1.23	0.560	3.7E-01	5	11.5
O43852	Calumenin	1.22	0.181	1.4E-01	4	11.1
P55265	Double-stranded RNA-specific adenosine deaminase	1.22	NA	NA	2	1.5
P53990	IST1 homolog	1.22	NA	NA	2	4.4
P62753	40S ribosomal protein S6	1.22	0.035	3.9E-04	3	12.0
P59998	Actin-related protein 2/3 complex subunit 4	1.22	0.093	2.9E-01	3	16.1
P38117	Electron transfer flavoprotein subunit beta	1.22	0.234	4.8E-01	3	13.7
P30101	Protein disulfide-isomerase A3	1.22	0.062	9.1E-03	14	26.1
P35998	26S protease regulatory subunit 7	1.22	NA	NA	2	5.8
Q13310	Polyadenylate-binding protein 4	1.22	NA	NA	2	4.7
P12270	Nucleoprotein TPR	1.22	0.593	4.1E-01	3	1.9
Q14165	Malectin	1.22	0.118	1.8E-01	3	7.9
P69905	Hemoglobin subunit alpha	1.21	0.119	5.0E-03	7	61.3
P61225	Ras-related protein Rap-2b	1.21	NA	NA	2	10.4
O94826	Mitochondrial import receptor subunit TOM70	1.21	0.153	2.2E-01	3	4.3
P18124	60S ribosomal protein L7	1.21	0.058	9.9E-03	6	20.6
P78371	T-complex protein 1 subunit beta	1.20	0.143	1.6E-01	8	14.4
P53597	Succinyl-CoA ligase [ADP/GDP-forming] subunit alpha, mitochondrial	1.20	NA	NA	2	6.9
Q14980	Nuclear mitotic apparatus protein 1	1.20	0.172	9.9E-02	7	4.1
O75947	ATP synthase subunit d, mitochondrial	1.20	0.127	2.1E-01	3	15.5
P43686	26S protease regulatory subunit 6B	1.20	0.041	2.5E-02	4	10.3
P20810	Calpastatin	1.19	0.122	4.9E-01	3	8.2
O15511	Actin-related protein 2/3 complex subunit 5	1.19	0.067	1.4E-01	3	21.9
P06730	Eukaryotic translation initiation factor 4E	1.19	NA	NA	2	9.7
P49821	NADH dehydrogenase [ubiquinone] flavoprotein 1, mitochondrial	1.19	NA	NA	2	3.4
P26038	Moesin	1.18	0.095	1.1E-02	11	13.9
Q16543	Hsp90 co-chaperone Cdc37	1.18	NA	NA	2	5.3
Q14152	Eukaryotic translation initiation factor 3 subunit A	1.18	NA	NA	2	1.8
P14314	Glucosidase 2 subunit beta	1.17	0.188	1.9E-01	11	18.6
P61106	Ras-related protein Rab-14	1.17	0.088	2.2E-01	4	21.4
Q06830	Peroxiredoxin-1	1.17	0.134	6.6E-02	10	48.7
Q86VP6	Cullin-associated NEDD8-dissociated protein 1	1.17	0.410	3.8E-01	5	4.2
Q8N1G4	Leucine-rich repeat-containing protein 47	1.16	0.038	2.5E-02	4	7.0
P31150	Rab GDP dissociation inhibitor alpha	1.15	NA	NA	2	5.4
P07237	Protein disulfide-isomerase	1.15	0.128	1.9E-01	13	21.5
P02774	Vitamin D-binding protein	1.15	NA	NA	2	3.0
Q9Y4I1	Unconventional myosin-Va	1.14	NA	NA	2	1.3
P40939	Trifunctional enzyme subunit alpha, mitochondrial	1.13	0.073	5.9E-02	10	14.3
P20700	Lamin-B1	1.13	0.053	2.7E-02	6	10.4
Q16629	Serine/arginine-rich splicing factor 7	1.12	NA	NA	2	7.6
P13796	Plastin-2	1.12	0.168	3.5E-01	5	8.5
P46781	40S ribosomal protein S9	1.12	0.682	4.9E-01	6	22.7
P27708	CAD protein	1.12	NA	NA	2	0.9
P01023	Alpha-2-macroglobulin	1.12	0.102	9.7E-02	5	3.9
P14866	Heterogeneous nuclear ribonucleoprotein L	1.12	0.220	2.1E-01	5	10.2
Q9H3N1	Thioredoxin-related transmembrane protein 1	1.11	NA	NA	2	8.2
Q53GQ0	Estradiol 17-beta-dehydrogenase 12	1.11	0.093	1.8E-01	4	15.1
Q9Y265	RuvB-like 1	1.11	NA	NA	2	5.0
P35613	Basigin	1.11	NA	NA	2	8.3
P13073	Cytochrome c oxidase subunit 4 isoform 1, mitochondrial	1.10	0.550	6.5E-01	3	19.5
P54709	Sodium/potassium-transporting ATPase subunit beta-3	1.10	0.101	4.0E-01	4	15.8
Q8NBS9	Thioredoxin domain-containing protein 5	1.10	14.905	7.3E-01	3	4.6
P49368	T-complex protein 1 subunit gamma	1.10	0.117	1.2E-01	8	15.4
Q86UP2	Kinectin	1.10	0.342	5.8E-01	5	4.3
P45880	Voltage-dependent anion-selective channel protein 2	1.10	0.163	3.0E-01	7	24.5

P40429	60S ribosomal protein L13a	1.10	0.173	3.6E-01	6	23.6
P29692	Elongation factor 1-delta	1.09	0.199	6.8E-01	3	11.4
Q9BXX0	EMILIN-2	1.09	2.156	7.2E-01	3	3.1
P42858	Huntingtin	1.09	NA	NA	2	0.5
P61160	Actin-related protein 2	1.09	0.334	4.0E-01	4	10.7
P46109	Crk-like protein	1.08	NA	NA	2	7.9
Q92896	Golgi apparatus protein 1	1.08	0.047	1.8E-01	3	2.5
Q02543	60S ribosomal protein L18a	1.08	NA	NA	2	10.2
P01857	Ig gamma-1 chain C region	1.08	0.192	2.1E-01	4	18.5
Q9UNZ2	NSFL1 cofactor p47	1.08	NA	NA	2	7.8
Q9Y2B0	Protein canopy homolog 2	1.08	NA	NA	2	13.7
Q9NSE4	Isoleucine--tRNA ligase, mitochondrial	1.08	3.183	6.0E-01	4	5.9
O75340	Programmed cell death protein 6	1.08	0.438	4.8E-01	3	16.2
P06576	ATP synthase subunit beta, mitochondrial	1.08	0.084	2.0E-01	10	24.6
Q9HD20	Manganese-transporting ATPase 13A1	1.07	0.187	7.9E-01	3	3.6
P46782	40S ribosomal protein S5	1.07	1.281	6.0E-01	3	8.8
O15173	Membrane-associated progesterone receptor component 2	1.07	0.114	2.5E-01	3	17.9
Q01844	RNA-binding protein EWS	1.07	NA	NA	2	2.3
P50454	Serpin H1	1.06	NA	NA	2	6.0
P07919	Cytochrome b-c1 complex subunit 6, mitochondrial	1.06	NA	NA	2	15.4
P58876	Histone H2B type 1-D	1.06	NA	NA	2	7.9
O43707	Alpha-actinin-4	1.06	0.193	1.4E-01	22	27.7
P62701	40S ribosomal protein S4, X isoform	1.06	0.133	2.9E-01	4	9.5
P11586	C-1-tetrahydrofolate synthase, cytoplasmic	1.05	0.119	3.5E-01	5	6.6
P61247	40S ribosomal protein S3a	1.05	0.407	8.9E-01	6	20.5
P04632	Calpain small subunit 1	1.05	0.744	6.4E-01	3	9.0
Q9P2E9	Ribosome-binding protein 1	1.05	0.218	6.4E-01	5	4.1
P50213	Isocitrate dehydrogenase [NAD] subunit alpha, mitochondrial	1.05	0.319	6.4E-01	3	10.1
Q9HDC9	Adipocyte plasma membrane-associated protein	1.04	NA	NA	2	5.5
P02042	Hemoglobin subunit delta	1.04	NA	NA	2	17.7
P09874	Poly [ADP-ribose] polymerase 1	1.04	0.396	8.4E-01	6	7.7
Q15393	Splicing factor 3B subunit 3	1.04	NA	NA	2	2.1
P50502	Hsc70-interacting protein	1.03	0.873	6.9E-01	3	8.9
P55072	Transitional endoplasmic reticulum ATPase	1.03	0.424	4.4E-01	11	14.0
P01009	Alpha-1-antitrypsin	1.03	0.280	6.7E-01	10	24.2
Q9UQE7	Structural maintenance of chromosomes protein 3	1.03	NA	NA	2	1.7
P35580	Myosin-10	1.03	0.601	5.8E-01	23	14.1
Q99442	Translocation protein SEC62	1.03	0.282	8.1E-01	3	7.3
P25705	ATP synthase subunit alpha, mitochondrial	1.03	0.188	6.0E-01	14	26.8
Q9UHD8	Septin-9	1.03	0.439	8.5E-01	4	7.2
Q9Y3U8	60S ribosomal protein L36	1.02	0.598	9.1E-01	3	21.9
P62136	Serine/threonine-protein phosphatase PP1-alpha catalytic subunit	1.02	0.082	5.8E-01	3	9.7
Q02818	Nucleobindin-1	1.02	NA	NA	2	4.8
Q16795	NADH dehydrogenase [ubiquinone] 1 alpha subcomplex subunit 9, mitochondrial	1.02	0.281	8.9E-01	3	9.8
P48681	Nestin	1.01	NA	NA	2	1.5
Q15233	Non-POU domain-containing octamer-binding protein	1.01	0.294	9.6E-01	4	6.6
P35221	Catenin alpha-1	1.01	0.422	9.7E-01	4	5.4
P50990	T-complex protein 1 subunit theta	1.00	1.381	9.6E-01	11	19.7
P10606	Cytochrome c oxidase subunit 5B, mitochondrial	1.00	0.544	9.9E-01	5	25.6
P39023	60S ribosomal protein L3	1.00	NA	NA	2	6.0
P61421	V-type proton ATPase subunit d 1	1.00	0.242	9.4E-01	3	7.7
Q13162	Peroxiredoxin-4	1.00	0.081	9.8E-01	3	12.9
Q8N5K1	CDGSH iron-sulfur domain-containing protein 2	0.99	NA	NA	2	15.6
P02545	Prelamin-A/C	0.99	0.408	8.0E-01	34	49.8
O75964	ATP synthase subunit g, mitochondrial	0.99	0.219	8.8E-01	3	38.8
P22695	Cytochrome b-c1 complex subunit 2, mitochondrial	0.99	0.765	8.9E-01	6	17.0
P17858	ATP-dependent 6-phosphofructokinase, liver type	0.99	0.739	9.5E-01	4	6.4
P36957	Dihydrolipoyllysine-residue succinyltransferase component of 2-oxoglutarate dehydrogenase complex, mitochondrial	0.99	0.080	7.7E-01	5	11.5
P54920	Alpha-soluble NSF attachment protein	0.99	0.532	9.3E-01	5	17.6
O75643	U5 small nuclear ribonucleoprotein 200 kDa helicase	0.99	NA	NA	2	0.9
P11021	78 kDa glucose-regulated protein	0.98	0.203	7.4E-01	21	29.4
Q92499	ATP-dependent RNA helicase DDX1	0.98	1.230	8.9E-01	5	5.9
Q7L5N1	COP9 signalosome complex subunit 6	0.98	0.102	6.5E-01	3	10.1
P08107	Heat shock 70 kDa protein 1A/1B	0.98	0.337	7.9E-01	14	23.1
P04844	Dolichyl-diphosphooligosaccharide--protein glycosyltransferase subunit 2	0.98	NA	NA	2	4.1
Q9UBQ0	Vacuolar protein sorting-associated protein 29	0.98	NA	NA	2	8.2
O60506	Heterogeneous nuclear ribonucleoprotein Q	0.98	NA	NA	2	2.9
P31689	DnaJ homolog subfamily A member 1	0.97	NA	NA	2	4.8
Q00765	Receptor expression-enhancing protein 5	0.96	0.785	7.8E-01	3	10.6
Q9Y3Z3	Deoxynucleoside triphosphate triphosphohydrolase SAMHD1	0.96	NA	NA	2	3.5
P11177	Pyruvate dehydrogenase E1 component subunit beta, mitochondrial	0.96	0.512	5.5E-01	3	7.2
P10515	Dihydrolipoyllysine-residue acetyltransferase component of pyruvate dehydrogenase complex, mitochondrial	0.96	NA	NA	2	2.6
Q02878	60S ribosomal protein L6	0.96	0.090	5.5E-01	5	18.4
P18077	60S ribosomal protein L35a	0.95	NA	NA	2	17.3
Q14108	Lysosome membrane protein 2	0.95	NA	NA	2	4.6
P50402	Emerin	0.95	0.314	7.6E-01	4	17.3
O75915	PRA1 family protein 3	0.95	NA	NA	2	9.6
Q96AE4	Far upstream element-binding protein 1	0.95	1.644	8.5E-01	3	4.5
Q9NYU2	UDP-glucose:glycoprotein glucosyltransferase 1	0.95	NA	NA	2	1.5
P30153	Serine/threonine-protein phosphatase 2A 65 kDa regulatory subunit A alpha isoform	0.94	NA	NA	2	4.8
Q02252	Methylmalonate-semialdehyde dehydrogenase [acylating], mitochondrial	0.94	0.138	4.8E-01	3	6.2
P63092	Guanine nucleotide-binding protein G(s) subunit alpha isoforms short	0.94	0.456	8.4E-01	3	10.4
Q00325	Phosphate carrier protein, mitochondrial	0.94	0.216	5.6E-01	5	13.5
P68871	Hemoglobin subunit beta	0.94	0.127	3.7E-01	5	44.9
O60716	Catenin delta-1	0.93	NA	NA	2	2.0
P11940	Polyadenylate-binding protein 1	0.93	0.113	2.5E-01	4	8.2
Q16891	Mitochondrial inner membrane protein	0.93	0.523	5.4E-01	7	11.3
P53618	Coatomer subunit beta	0.93	NA	NA	2	1.7
P11216	Glycogen phosphorylase, brain form	0.93	0.128	4.3E-01	4	6.3
Q7KZF4	Staphylococcal nuclease domain-containing protein 1	0.92	0.299	6.2E-01	4	4.8
O60313	Dynamin-like 120 kDa protein, mitochondrial	0.92	0.054	2.1E-01	4	6.4
O95299	NADH dehydrogenase [ubiquinone] 1 alpha subcomplex subunit 10, mitochondrial	0.92	NA	NA	2	2.8
P61586	Transforming protein RhoA	0.92	0.220	4.5E-01	5	24.9
Q07020	60S ribosomal protein L18	0.91	NA	NA	2	13.8
Q13200	26S proteasome non-ATPase regulatory subunit 2	0.90	NA	NA	2	2.3
Q9Y262	Eukaryotic translation initiation factor 3 subunit L	0.90	0.954	6.7E-01	3	4.6
Q99832	T-complex protein 1 subunit eta	0.90	0.070	2.5E-01	3	6.6
Q9UHQ9	NADH-cytochrome b5 reductase 1	0.90	0.451	5.1E-01	3	9.8
P00738	Haptoglobin	0.90	0.132	2.2E-01	8	20.0
P17987	T-complex protein 1 subunit alpha	0.90	0.101	2.4E-01	6	11.5
P39656	Dolichyl-diphosphooligosaccharide--protein glycosyltransferase 48 kDa subunit	0.89	0.054	4.3E-02	5	10.3
P30048	Thioredoxin-dependent peroxide reductase, mitochondrial	0.89	NA	NA	2	7.0
P33176	Kinesin-1 heavy chain	0.88	NA	NA	2	3.0
P35222	Catenin beta-1	0.88	0.021	7.1E-03	6	9.3
Q13561	Dynactin subunit 2	0.88	0.292	3.2E-01	5	13.0
Q9BRX8	Redox-regulatory protein FAM213A	0.88	0.448	2.9E-01	4	16.6
P09543	2',3'-cyclic-nucleotide 3'-phosphodiesterase	0.88	0.084	1.8E-01	6	11.9

Q9UIJ7	GTP:AMP phosphotransferase AK3, mitochondrial	0.88	0.094	2.1E-01	3	15.0
P48643	T-complex protein 1 subunit epsilon	0.88	0.256	3.1E-01	7	12.6
P28331	NADH-ubiquinone oxidoreductase 75 kDa subunit, mitochondrial	0.87	NA	NA	2	2.3
O95865	N(G),N(G)-dimethylarginine dimethylaminohydrolase 2	0.87	NA	NA	2	8.4
P51572	B-cell receptor-associated protein 31	0.87	0.727	3.8E-01	7	27.2
P43304	Glycerol-3-phosphate dehydrogenase, mitochondrial	0.86	0.186	3.6E-01	3	5.2
P31930	Cytochrome b-c1 complex subunit 1, mitochondrial	0.86	0.326	3.3E-01	4	9.8
P60709	Actin, cytoplasmic 1	0.86	0.084	4.5E-02	5	21.3
P00505	Aspartate aminotransferase, mitochondrial	0.86	1.264	2.3E-01	6	16.7
P24752	Acetyl-CoA acetyltransferase, mitochondrial	0.86	0.430	4.8E-01	4	10.1
P46977	Dolichyl-diphosphooligosaccharide--protein glycosyltransferase subunit STT3A	0.85	NA	NA	2	2.3
P14927	Cytochrome b-c1 complex subunit 7	0.85	NA	NA	2	20.7
O94979	Protein transport protein Sec31A	0.85	NA	NA	2	2.3
P04843	Dolichyl-diphosphooligosaccharide--protein glycosyltransferase subunit 1	0.85	0.093	4.2E-02	9	15.5
P30533	Alpha-2-macroglobulin receptor-associated protein	0.85	NA	NA	2	4.5
Q13098	COP9 signalosome complex subunit 1	0.85	1.196	7.9E-01	3	5.3
Q12797	Aspartyl/asparaginyl beta-hydroxylase	0.85	0.210	4.0E-01	4	4.1
Q15075	Early endosome antigen 1	0.85	NA	NA	2	1.9
Q10567	AP-1 complex subunit beta-1	0.84	NA	NA	2	2.1
Q02218	2-oxoglutarate dehydrogenase, mitochondrial	0.84	0.114	6.5E-02	7	8.5
Q13228	Selenium-binding protein 1	0.84	NA	NA	2	4.9
Q9Y277	Voltage-dependent anion-selective channel protein 3	0.83	NA	NA	2	8.5
Q14204	Cytoplasmic dynein 1 heavy chain 1	0.83	0.055	4.5E-04	35	8.0
Q9P0L0	Vesicle-associated membrane protein-associated protein A	0.83	NA	NA	2	11.2
P04217	Alpha-1B-glycoprotein	0.82	NA	NA	2	4.8
P38919	Eukaryotic initiation factor 4A-III	0.82	0.026	2.6E-01	3	9.2
P24539	ATP synthase F(0) complex subunit B1, mitochondrial	0.82	NA	NA	2	9.0
P28838	Cytosol aminopeptidase	0.82	0.879	6.5E-01	4	10.8
Q9NQC3	Reticulon-4	0.82	NA	NA	2	2.3
P50991	T-complex protein 1 subunit delta	0.82	0.169	8.4E-02	4	8.5
P27824	Calnexin	0.82	0.236	6.6E-02	12	20.6
O43242	26S proteasome non-ATPase regulatory subunit 3	0.82	NA	NA	2	3.0
P05388	60S acidic ribosomal protein P0	0.81	0.710	4.4E-01	3	10.1
P16615	Sarcoplasmic/endoplasmic reticulum calcium ATPase 2	0.81	0.033	1.4E-03	6	5.8
Q9Y3I0	tRNA-splicing ligase RtcB homolog	0.81	0.216	3.2E-01	5	12.7
P02511	Alpha-crystallin B chain	0.81	0.137	4.4E-02	6	35.4
P53621	Coatomer subunit alpha	0.81	0.266	5.0E-01	3	2.9
P40227	T-complex protein 1 subunit zeta	0.81	0.580	5.2E-01	3	5.6
O14950	Myosin regulatory light chain 12B	0.81	0.078	1.8E-03	3	12.8
Q969X5	Endoplasmic reticulum-Golgi intermediate compartment protein 1	0.81	NA	NA	2	6.2
Q71U36	Tubulin alpha-1A chain	0.81	NA	NA	2	6.7
P12236	ADP/ATP translocase 3	0.80	0.096	1.3E-02	7	21.8
P49748	Very long-chain specific acyl-CoA dehydrogenase, mitochondrial	0.80	0.216	1.5E-01	7	9.3
P50995	Annexin A11	0.80	NA	NA	2	4.4
P42765	3-ketoacyl-CoA thiolase, mitochondrial	0.80	0.148	4.0E-01	3	9.6
P04179	Superoxide dismutase [Mn], mitochondrial	0.80	0.239	1.9E-01	7	24.3
Q9GZM7	Tubulointerstitial nephritis antigen-like	0.80	NA	NA	2	6.2
Q13423	NAD(P) transhydrogenase, mitochondrial	0.79	NA	NA	2	2.3
Q9BS26	Endoplasmic reticulum resident protein 44	0.79	0.064	2.6E-02	3	7.9
P08133	Annexin A6	0.79	0.038	4.2E-07	27	44.3
Q8IVF2	Protein AHNAK2	0.79	NA	NA	2	0.4
Q9NZ08	Endoplasmic reticulum aminopeptidase 1	0.79	NA	NA	2	2.2
Q14203	Dynactin subunit 1	0.78	0.056	8.8E-03	4	4.1
O00203	AP-3 complex subunit beta-1	0.78	NA	NA	2	2.7
P09496	Clathrin light chain A	0.78	NA	NA	2	5.6
P46937	Yorkie homolog	0.78	NA	NA	2	6.2
P47756	F-actin-capping protein subunit beta	0.78	0.118	1.5E-01	3	11.6
P07384	Calpain-1 catalytic subunit	0.77	NA	NA	2	3.9
P00390	Glutathione reductase, mitochondrial	0.77	NA	NA	2	5.7
O43390	Heterogeneous nuclear ribonucleoprotein R	0.77	0.079	1.0E-02	6	8.7
P04040	Catalase	0.77	0.057	1.7E-01	3	6.5
Q6IAA8	Ragulator complex protein LAMTOR1	0.76	NA	NA	2	17.4
P05556	Integrin beta-1	0.75	0.234	7.9E-02	5	6.8
Q16698	2,4-dienoyl-CoA reductase, mitochondrial	0.75	0.275	5.6E-01	3	11.3
Q9UHG3	Prenylcysteine oxidase 1	0.75	0.103	1.1E-01	3	6.3
P43307	Translocon-associated protein subunit alpha	0.75	NA	NA	2	6.6
P61626	Lysozyme C	0.75	NA	NA	2	12.8
P14625	Endoplasmin	0.75	0.050	2.9E-06	10	13.4
P00387	NADH-cytochrome b5 reductase 3	0.75	0.191	8.6E-02	5	18.3
P13667	Protein disulfide-isomerase A4	0.74	0.075	9.7E-04	7	11.3
Q5SSJ5	Heterochromatin protein 1-binding protein 3	0.74	NA	NA	2	5.2
Q93009	Ubiquitin carboxyl-terminal hydrolase 7	0.74	NA	NA	2	1.5
P31153	S-adenosylmethionine synthase isoform type-2	0.74	NA	NA	2	7.6
P62805	Histone H4	0.73	0.034	1.0E-08	8	52.4
P17655	Calpain-2 catalytic subunit	0.73	NA	NA	2	2.9
P0C0S5	Histone H2A.Z	0.73	NA	NA	2	18.8
Q8WUM4	Programmed cell death 6-interacting protein	0.73	0.056	1.4E-02	5	6.2
P62873	Guanine nucleotide-binding protein G(I)/G(S)/G(T) subunit beta-1	0.72	NA	NA	2	9.4
P01860	Ig gamma-3 chain C region	0.72	0.092	3.8E-02	3	6.4
Q07065	Cytoskeleton-associated protein 4	0.72	0.068	3.0E-03	5	11.8
Q15293	Reticulocalbin-1	0.71	0.253	2.0E-01	3	8.2
P48735	Isocitrate dehydrogenase [NADP], mitochondrial	0.71	0.127	4.4E-02	4	10.6
P07305	Histone H1.0	0.71	0.058	1.3E-01	4	20.6
Q9P0M6	Core histone macro-H2A.2	0.71	NA	NA	2	7.3
P00367	Glutamate dehydrogenase 1, mitochondrial	0.70	0.136	6.1E-02	8	16.5
P21589	5'-nucleotidase	0.70	NA	NA	2	4.0
P13987	CD59 glycoprotein	0.69	NA	NA	2	15.6
P13861	cAMP-dependent protein kinase type II-alpha regulatory subunit	0.69	0.096	7.6E-02	4	13.9
P68371	Tubulin beta-4B chain	0.67	0.033	5.9E-10	3	9.9
Q00610	Clathrin heavy chain 1	0.67	0.040	7.1E-10	23	15.4
Q9BSJ8	Extended synaptotagmin-1	0.67	0.130	6.2E-02	3	3.5
Q14344	Guanine nucleotide-binding protein subunit alpha-13	0.67	0.022	3.8E-04	3	8.5
P17980	26S protease regulatory subunit 6A	0.67	0.040	2.0E-02	3	5.5
P01834	Ig kappa chain C region	0.66	NA	NA	2	34.9
P67936	Tropomyosin alpha-4 chain	0.66	0.166	9.6E-03	8	25.8
Q13425	Beta-2-syntrophin	0.66	NA	NA	2	3.1
P05023	Sodium/potassium-transporting ATPase subunit alpha-1	0.65	0.034	4.4E-07	20	22.4
P04899	Guanine nucleotide-binding protein G(i) subunit alpha-2	0.64	NA	NA	2	7.6
P05091	Aldehyde dehydrogenase, mitochondrial	0.64	0.165	7.1E-02	3	6.0
O00264	Membrane-associated progesterone receptor component 1	0.63	0.122	9.1E-03	3	12.3
Q99584	Protein S100-A13	0.63	0.095	6.4E-03	5	41.8
Q16695	Histone H3.1t	0.62	0.062	2.1E-04	3	14.7
P61769	Beta-2-microglobulin	0.62	0.119	2.5E-02	3	19.3
P17612	cAMP-dependent protein kinase catalytic subunit alpha	0.62	NA	NA	2	4.3
Q03252	Lamin-B2	0.62	0.042	6.1E-05	16	27.8
Q6DD88	Atlastin-3	0.62	0.045	1.8E-01	3	5.7
P06899	Histone H2B type 1-J	0.62	NA	NA	2	7.9

Table S10-Sample UM26

P83731	60S ribosomal protein L24	0.61	NA	NA	2	13.4
P09497	Clathrin light chain B	0.61	NA	NA	2	8.3
Q08722	Leukocyte surface antigen CD47	0.61	NA	NA	2	5.9
Q01082	Spectrin beta chain, non-erythrocytic 1	0.60	0.033	2.2E-16	54	27.1
O94905	Erlin-2	0.60	NA	NA	2	5.0
P01042	Kininogen-1	0.60	0.122	1.9E-02	4	6.1
P01024	Complement C3	0.60	0.157	3.1E-03	22	13.2
P11413	Glucose-6-phosphate 1-dehydrogenase	0.59	NA	NA	2	3.7
P06727	Apolipoprotein A-IV	0.59	0.073	3.9E-03	8	17.4
P07099	Epoxide hydrolase 1	0.57	0.072	3.4E-05	8	15.4
O94911	ATP-binding cassette sub-family A member 8	0.57	0.551	4.4E-01	3	2.2
Q13813	Spectrin alpha chain, non-erythrocytic 1	0.57	0.021	0.0E+00	72	32.2
P60660	Myosin light polypeptide 6	0.57	0.047	6.2E-10	9	62.3
Q9Y490	Talin-1	0.56	0.044	5.0E-11	28	14.0
Q92556	Engulfment and cell motility protein 1	0.55	0.177	1.9E-01	3	5.0
P35579	Myosin-9	0.55	0.025	0.0E+00	59	26.9
P29992	Guanine nucleotide-binding protein subunit alpha-11	0.54	NA	NA	2	6.7
P05090	Apolipoprotein D	0.54	NA	NA	2	12.7
Q14764	Major vault protein	0.53	0.127	1.7E-02	3	3.7
O75923	Dysferlin	0.53	NA	NA	2	1.6
Q8TD19	Serine/threonine-protein kinase Nek9	0.53	NA	NA	2	2.2
P0C0L5	Complement C4-B	0.53	0.074	1.8E-05	7	3.4
P06756	Integrin alpha-V	0.53	0.159	2.9E-02	3	2.3
Q15149	Plectin	0.52	0.030	0.0E+00	44	10.1
P10301	Ras-related protein R-Ras	0.52	NA	NA	2	10.6
P80723	Brain acid soluble protein 1	0.51	0.095	7.7E-04	8	40.1
Q9Y6N5	Sulfide:quinone oxidoreductase, mitochondrial	0.51	0.268	7.7E-02	3	7.3
P01876	Ig alpha-1 chain C region	0.50	NA	NA	2	4.5
Q14624	Inter-alpha-trypsin inhibitor heavy chain H4	0.50	0.128	3.3E-02	4	5.4
O00159	Unconventional myosin-Ic	0.49	0.080	2.2E-03	6	5.4
P0CG05	Ig lambda-2 chain C regions	0.49	NA	NA	2	27.4
O43301	Heat shock 70 kDa protein 12A	0.49	0.178	1.0E-01	4	7.3
Q15582	Transforming growth factor-beta-induced protein ig-h3	0.48	NA	NA	2	3.4
Q16181	Septin-7	0.47	0.083	3.8E-05	5	12.4
Q15019	Septin-2	0.47	0.067	5.3E-04	3	12.2
P42167	Lamina-associated polypeptide 2, isoforms beta/gamma	0.47	NA	NA	2	4.4
P12111	Collagen alpha-3(VI) chain	0.46	0.045	5.9E-13	31	10.5
P12109	Collagen alpha-1(VI) chain	0.46	0.080	4.8E-06	9	9.8
P12110	Collagen alpha-2(VI) chain	0.46	0.064	1.5E-07	9	9.2
P07355	Annexin A2	0.46	0.035	0.0E+00	23	56.0
P32119	Peroxiredoxin-2	0.45	0.070	1.4E-06	4	18.2
P20073	Annexin A7	0.45	0.167	5.6E-01	3	8.6
Q2VIR3	Putative eukaryotic translation initiation factor 2 subunit 3-like protein	0.44	NA	NA	2	3.4
Q09666	Neuroblast differentiation-associated protein AHNAK	0.44	0.035	0.0E+00	68	10.4
Q9HBL0	Tensin-1	0.44	0.262	3.6E-01	4	3.9
Q96CW1	AP-2 complex subunit mu	0.44	NA	NA	2	4.1
P06703	Protein S100-A6	0.43	NA	NA	2	16.7
P00747	Plasminogen	0.43	0.118	5.1E-02	5	8.4
O43760	Synaptogyrin-2	0.42	NA	NA	2	8.9
P55083	Microfibril-associated glycoprotein 4	0.40	NA	NA	2	5.1
P23634	Plasma membrane calcium-transporting ATPase 4	0.38	NA	NA	2	2.7
P28289	Tropomodulin-1	0.37	NA	NA	2	4.7
P02462	Collagen alpha-1(IV) chain	0.36	0.106	7.4E-02	3	2.3
P46939	Utrophin	0.36	NA	NA	2	0.8
P22105	Tenascin-X	0.35	NA	NA	2	0.8
Q63HR2	Tensin-like C1 domain-containing phosphatase	0.34	NA	NA	2	1.5
P35611	Alpha-adducin	0.32	NA	NA	2	4.5
P23229	Integrin alpha-6	0.32	NA	NA	2	1.4
Q9BS40	Latexin	0.31	NA	NA	2	12.2
P01892	HLA class I histocompatibility antigen, A-2 alpha chain	0.25	NA	NA	2	6.6
Q92777	Synapsin-2	0.24	NA	NA	2	4.1
Q16363	Laminin subunit alpha-4	0.24	0.278	6.6E-02	5	3.8
O14495	Lipid phosphate phosphohydrolase 3	0.23	NA	NA	2	7.1
Q14699	Raftlin	0.22	NA	NA	2	3.8
P07996	Thrombospondin-1	0.22	NA	NA	2	2.5
P10745	Retinol-binding protein 3	0.22	NA	NA	2	1.8
Q92522	Histone H1x	0.20	NA	NA	2	11.7
Q12805	EGF-containing fibulin-like extracellular matrix protein 1	0.20	NA	NA	2	3.9
Q03135	Caveolin-1	0.19	NA	NA	2	11.8
P16157	Ankyrin-1	0.19	0.306	2.1E-01	3	1.8
P11277	Spectrin beta chain, erythrocytic	0.18	0.455	1.1E-01	5	3.2
P58166	Inhibin beta E chain	0.18	NA	NA	2	5.7
Q9BXN1	Asporin	0.17	NA	NA	2	5.5
P05186	Alkaline phosphatase, tissue-nonspecific isozyme	0.15	0.424	2.6E-01	3	5.0
P23946	Chymase	0.14	0.260	9.5E-02	4	21.1
P07196	Neurofilament light polypeptide	0.14	NA	NA	2	3.7
P24844	Myosin regulatory light polypeptide 9	0.10	NA	NA	2	12.2
P02452	Collagen alpha-1(I) chain	0.07	NA	NA	2	2.2
O75369	Filamin-B	0.41	0.115	5.9E-04	8	4.7
Q05682	Caldesmon	0.41	0.031	2.2E-04	5	7.2
Q07954	Prolow-density lipoprotein receptor-related protein 1	0.40	0.049	1.7E-04	13	3.7
P02751	Fibronectin	0.40	0.094	6.1E-04	13	7.4
P12814	Alpha-actinin-1	0.40	0.074	1.6E-08	8	10.3
P00167	Cytochrome b5	0.37	0.108	5.9E-04	4	42.5
P18206	Vinculin	0.36	0.091	1.2E-08	15	16.7
P00450	Ceruloplasmin	0.36	0.052	7.3E-10	9	10.4
Q13509	Tubulin beta-3 chain	0.36	0.122	1.6E-03	3	6.7
P05362	Intercellular adhesion molecule 1	0.36	0.123	3.2E-02	3	7.9
P22413	Ectonucleotide pyrophosphatase/phosphodiesterase family member 1	0.33	0.057	8.8E-04	4	4.4
P55268	Laminin subunit beta-2	0.33	0.085	4.1E-06	12	7.3
P21333	Filamin-A	0.33	0.038	0.0E+00	53	26.3
P50895	Basal cell adhesion molecule	0.33	0.150	4.4E-04	4	8.9
O15230	Laminin subunit alpha-5	0.31	0.107	2.4E-05	13	4.8
P21926	CD9 antigen	0.31	0.098	9.4E-07	3	9.6
P06396	Gelsolin	0.31	0.104	2.2E-08	11	14.6
P02654	Apolipoprotein C-I	0.30	0.109	2.4E-04	3	24.1
Q969G5	Protein kinase C delta-binding protein	0.30	0.154	2.3E-02	3	11.5
P02675	Fibrinogen beta chain	0.30	0.061	1.8E-06	9	25.3
P01011	Alpha-1-antichymotrypsin	0.29	0.126	2.4E-05	5	8.3
Q6NZI2	Polymerase I and transcript release factor	0.29	0.127	1.1E-05	6	19.2
P68032	Actin, alpha cardiac muscle 1	0.29	0.235	1.0E-03	6	21.0
Q16555	Dihydropyrimidinase-related protein 2	0.28	0.106	1.8E-07	8	15.6
P11166	Solute carrier family 2, facilitated glucose transporter member 1	0.28	0.079	1.1E-04	3	5.5
P07942	Laminin subunit beta-1	0.27	0.201	9.3E-03	3	1.7
Q9NZM1	Myoferlin	0.26	0.243	4.1E-02	3	1.6
P02679	Fibrinogen gamma chain	0.25	0.076	7.9E-10	10	22.1
P02549	Spectrin alpha chain, erythrocytic 1	0.24	0.119	4.4E-06	7	3.7

P11047	Laminin subunit gamma-1	0.24	0.110	4.4E-04	12	7.8
P36269	Gamma-glutamyltransferase 5	0.24	0.178	7.2E-06	7	14.8
P02686	Myelin basic protein	0.24	0.152	1.1E-05	4	14.5
P46821	Microtubule-associated protein 1B	0.24	0.191	2.7E-02	6	3.0
P07197	Neurofilament medium polypeptide	0.22	0.122	2.7E-02	3	3.8
O43491	Band 4.1-like protein 2	0.22	0.150	2.0E-03	5	6.7
P26447	Protein S100-A4	0.22	0.118	6.1E-04	3	27.7
P08572	Collagen alpha-2(IV) chain	0.21	0.170	9.7E-05	7	5.5
P98160	Basement membrane-specific heparan sulfate proteoglycan core protein	0.21	0.070	0.0E+00	25	6.9
P08294	Extracellular superoxide dismutase [Cu-Zn]	0.21	0.176	2.2E-03	3	15.4
P39060	Collagen alpha-1(XVIII) chain	0.21	0.105	1.3E-09	7	4.1
P02649	Apolipoprotein E	0.19	0.073	3.4E-12	15	46.1
Q14112	Nidogen-2	0.19	0.134	2.8E-06	6	4.9
P01008	Antithrombin-III	0.18	0.223	1.6E-02	5	12.1
P02760	Protein AMBP	0.18	0.116	5.5E-06	4	19.0
Q02952	A-kinase anchor protein 12	0.18	0.115	8.1E-07	15	10.6
P04083	Annexin A1	0.17	0.080	8.2E-15	10	28.3
P09493	Tropomyosin alpha-1 chain	0.17	0.170	1.4E-04	5	12.0
P39059	Collagen alpha-1(XV) chain	0.17	0.172	2.2E-04	3	2.2
P01871	Ig mu chain C region	0.17	0.199	1.8E-04	7	19.5
P22748	Carbonic anhydrase 4	0.17	0.073	1.5E-10	9	29.8
P43320	Beta-crystallin B2	0.16	0.157	1.8E-02	3	15.6
O94875	Sorbin and SH3 domain-containing protein 2	0.16	0.338	1.7E-02	3	4.6
P27105	Erythrocyte band 7 integral membrane protein	0.16	0.136	1.1E-04	6	22.9
Q9BXM0	Periaxin	0.15	0.279	5.1E-03	5	1.6
Q05707	Collagen alpha-1(XIV) chain	0.15	0.313	3.2E-03	5	3.0
Q01995	Transgelin	0.15	0.097	1.3E-02	5	25.9
Q14195	Dihydropyrimidinase-related protein 3	0.15	0.149	1.6E-07	9	21.6
P60903	Protein S100-A10	0.15	0.158	1.3E-02	3	35.1
Q9Y6C2	EMILIN-1	0.15	0.163	5.9E-04	6	8.5
P01031	Complement C5	0.15	0.160	9.8E-06	5	3.0
P04275	von Willebrand factor	0.15	0.136	3.7E-07	9	3.6
P02671	Fibrinogen alpha chain	0.15	0.100	2.9E-06	9	12.5
P07585	Decorin	0.14	0.176	1.1E-04	7	19.5
P35749	Myosin-11	0.14	0.109	1.9E-12	28	15.5
P14543	Nidogen-1	0.14	0.122	3.6E-06	7	6.0
P22352	Glutathione peroxidase 3	0.13	0.174	1.4E-05	3	11.5
P35555	Fibrillin-1	0.13	0.045	0.0E+00	38	14.5
P20774	Mimecan	0.13	0.133	2.3E-08	6	16.4
P15088	Mast cell carboxypeptidase A	0.12	0.499	4.0E-02	3	6.5
P41219	Peripherin	0.12	0.215	8.9E-05	9	20.0
P21980	Protein-glutamine gamma-glutamyltransferase 2	0.12	0.098	0.0E+00	14	20.1
Q15661	Tryptase alpha/beta-1	0.12	0.131	6.3E-10	4	17.1
P02748	Complement component C9	0.11	0.160	1.9E-05	7	13.2
Q16518	Retinoid isomerohydrolase	0.11	0.168	1.2E-02	3	6.2
P21810	Biglycan	0.11	0.116	1.3E-12	7	18.2
P10909	Clusterin	0.10	0.078	0.0E+00	14	26.9
P02730	Band 3 anion transport protein	0.10	0.137	2.5E-07	7	9.2
P51888	Prolargin	0.10	0.114	0.0E+00	11	30.6
P02743	Serum amyloid P-component	0.09	0.135	1.1E-04	7	27.8
P35625	Metalloproteinase inhibitor 3	0.09	0.092	7.7E-07	3	10.4
P51884	Lumican	0.08	0.051	0.0E+00	7	20.4
P04004	Vitronectin	0.08	0.134	1.7E-13	8	16.3
P08123	Collagen alpha-2(I) chain	0.06	0.133	4.3E-08	3	3.7
P25189	Myelin protein P0	0.04	0.167	4.4E-09	7	27.4

Brown denotes change ≥ 2 standard deviations (SD) from the mean, yellow denotes change ≥ 1 SD and green highlights p values ≤ 0.05. NA, not applicable, n<3 unique peptides.
