# Supplementary material for: iTRAQ Quantitative Proteomic Comparison of Metastatic and Non-Metastatic Uveal Melanoma Tumors
Source: PLoS One. 2015 Aug 25;10(8):e0135543. doi: 10.1371/journal.pone.0135543 (PMC4549237; doi:10.1371/journal.pone.0135543)
Supplement: S11 Table — (PDF) [file pone.0135543.s011.pdf]

Supplementary Table S11
Relative Protein Abundance: Sample UM02, Non-Metastatic
Total Proteins Quantified = 239; LogMedian Protein Ratio = 0.13; LogMean Protein Ratio = 0; Standard Deviation = 0.85

Uni-Prot Accession	Protein	Ratio UM/Control	Standard Deviation	*p* value	Unique Peptides	% Sequence Coverage
P09211	Glutathione S-transferase P	7.50	0.065	1.6E-12	6	39.5
P62937	Peptidyl-prolyl cis-trans isomerase A	5.90	0.091	0.0E+00	9	43.6
P30086	Phosphatidylethanolamine-binding protein 1	5.69	0.083	0.0E+00	9	51.3
P06748	Nucleophosmin	5.51	0.075	0.0E+00	9	33.3
P52565	Rho GDP-dissociation inhibitor 1	5.21	0.113	1.6E-09	4	22.5
P06733	Alpha-enolase	5.12	0.092	4.0E-15	13	39.6
P23528	Cofilin-1	4.21	0.120	6.2E-07	10	55.4
P13645	Keratin, type I cytoskeletal 10	4.03	0.261	2.6E-02	3	5.0
P02768	Serum albumin	3.71	0.024	0.0E+00	38	55.7
P16949	Stathmin	3.54	0.148	1.2E-04	5	38.9
P40926	Malate dehydrogenase, mitochondrial	3.31	0.066	1.7E-11	13	50.0
P61604	10 kDa heat shock protein, mitochondrial	3.24	0.094	3.9E-07	5	41.2
Q92820	Gamma-glutamyl hydrolase	3.11	0.071	1.2E-09	3	11.0
P23284	Peptidyl-prolyl cis-trans isomerase B	3.02	0.064	2.0E-15	12	54.6
P60174	Triosephosphate isomerase	2.95	0.099	3.0E-04	11	38.8
Q04760	Lactoylglutathione lyase	2.92	0.145	6.3E-06	5	27.7
P05387	60S acidic ribosomal protein P2	2.78	0.120	2.5E-04	5	70.4
P63241	Eukaryotic translation initiation factor 5A-1	2.77	0.144	2.7E-02	4	32.5
P09429	High mobility group protein B1	2.76	0.046	2.0E-15	6	33.0
P00558	Phosphoglycerate kinase 1	2.69	0.133	1.5E-04	4	14.6
P04264	Keratin, type II cytoskeletal 1	2.62	0.143	2.7E-03	4	7.1
P19338	Nucleolin	2.61	0.203	3.3E-02	3	4.1
P07195	L-lactate dehydrogenase B chain	2.46	0.124	3.6E-04	4	12.0
P04792	Heat shock protein beta-1	2.36	0.062	7.9E-10	7	38.0
P14174	Macrophage migration inhibitory factor	6.95	NA	NA	2	17.4
P15531	Nucleoside diphosphate kinase A	5.36	NA	NA	2	13.2
P17096	High mobility group protein HMG-I/HMG-Y	3.77	NA	NA	2	23.4
Q99497	Protein DJ-1	2.97	NA	NA	2	16.4
Q9BV36	Melanophilin	2.84	NA	NA	2	4.7
P35908	Keratin, type II cytoskeletal 2 epidermal	2.76	NA	NA	2	3.0
Q01469	Fatty acid-binding protein, epidermal	2.50	NA	NA	2	11.9
P12955	Xaa-Pro dipeptidase	2.49	NA	NA	2	5.3
P62158	Calmodulin	2.41	NA	NA	2	22.1
P63104	14-3-3 protein zeta/delta	2.39	0.234	7.9E-02	4	21.2
O75368	SH3 domain-binding glutamic acid-rich-like protein	2.31	0.130	3.5E-02	3	32.5
P09651	Heterogeneous nuclear ribonucleoprotein A1	2.25	0.079	9.0E-12	6	18.5
P08238	Heat shock protein HSP 90-beta	2.08	0.099	1.0E-04	5	7.7
P31948	Stress-induced-phosphoprotein 1	2.04	0.252	1.0E-01	4	7.2
P00338	L-lactate dehydrogenase A chain	2.03	0.074	1.5E-04	3	7.2
P29401	Transketolase	2.03	0.155	4.1E-02	11	19.4
P50213	Isocitrate dehydrogenase [NAD] subunit alpha, mitochondrial	2.01	NA	NA	2	5.5
P16070	CD44 antigen	2.00	0.143	5.8E-03	8	10.9
P07108	Acyl-CoA-binding protein	1.99	NA	NA	2	27.6
P99999	Cytochrome c	1.91	0.131	1.2E-03	4	39.0
P07910	Heterogeneous nuclear ribonucleoproteins C1/C2	1.90	0.590	2.4E-01	3	11.8
P08670	Vimentin	1.90	0.021	0.0E+00	33	66.5
P07237	Protein disulfide-isomerase	1.89	0.044	1.1E-08	10	20.3
P14618	Pyruvate kinase PKM	1.83	0.229	3.0E-03	7	14.5
P62263	40S ribosomal protein S14	1.80	NA	NA	2	15.9
Q00796	Sorbitol dehydrogenase	1.78	NA	NA	2	7.3
P06454	Prothymosin alpha	1.77	NA	NA	2	20.7
P18669	Phosphoglycerate mutase 1	1.77	0.052	9.1E-11	3	15.4
P08758	Annexin A5	1.75	0.083	6.6E-06	12	32.8
P30044	Peroxiredoxin-5, mitochondrial	1.73	NA	NA	2	8.9
P30101	Protein disulfide-isomerase A3	1.72	0.072	3.8E-06	14	25.0
Q06830	Peroxiredoxin-1	1.71	0.042	3.7E-13	9	51.8
Q9H0U4	Ras-related protein Rab-1B	1.70	NA	NA	2	11.4
Q08380	Galectin-3-binding protein	1.68	NA	NA	2	5.6
P49189	4-trimethylaminobutyraldehyde dehydrogenase	1.68	NA	NA	2	4.9
P29966	Myristoylated alanine-rich C-kinase substrate	1.67	NA	NA	2	5.4
P36873	Serine/threonine-protein phosphatase PP1-gamma catalytic subunit	1.66	NA	NA	2	6.2
P61313	60S ribosomal protein L15	1.65	NA	NA	2	11.3
P02787	Serotransferrin	1.65	0.090	1.3E-03	5	8.6
P62851	40S ribosomal protein S25	1.63	NA	NA	2	15.2
P30041	Peroxiredoxin-6	1.60	0.326	2.2E-01	3	12.9
P25398	40S ribosomal protein S12	1.60	0.095	8.4E-04	3	25.0
P21796	Voltage-dependent anion-selective channel protein 1	1.58	0.081	3.0E-03	4	17.7
P26373	60S ribosomal protein L13	1.57	NA	NA	2	10.0
P35232	Prohibitin	1.56	0.101	1.9E-03	7	27.6
P04406	Glyceraldehyde-3-phosphate dehydrogenase	1.56	0.103	5.4E-06	8	31.0
P10809	60 kDa heat shock protein, mitochondrial	1.55	0.092	7.3E-04	10	18.3
P20674	Cytochrome c oxidase subunit 5A, mitochondrial	1.54	NA	NA	2	20.7
P62906	60S ribosomal protein L10a	1.54	0.115	7.2E-02	3	18.4
P63244	Guanine nucleotide-binding protein subunit beta-2-like 1	1.50	NA	NA	2	7.6
P0CW22	40S ribosomal protein S17-like	1.50	NA	NA	2	16.3
Q13185	Chromobox protein homolog 3	1.50	NA	NA	2	14.8
P61088	Ubiquitin-conjugating enzyme E2 N	1.50	NA	NA	2	11.2
P78417	Glutathione S-transferase omega-1	1.50	NA	NA	2	7.5
P35637	RNA-binding protein FUS	1.48	NA	NA	2	6.3
P39019	40S ribosomal protein S19	1.48	0.031	7.2E-08	4	23.4
P46783	40S ribosomal protein S10	1.47	NA	NA	2	14.5
P36957	Dihydrolipoyllysine-residue succinyltransferase component of 2-oxoglutarate dehydrogenase complex, mitochondrial	1.46	NA	NA	2	5.5
P50914	60S ribosomal protein L14	1.43	NA	NA	2	11.2
P51149	Ras-related protein Rab-7a	1.42	NA	NA	2	11.6
P62277	40S ribosomal protein S13	1.42	NA	NA	2	11.3
P10599	Thioredoxin	1.41	NA	NA	2	20.0
P40967	Melanocyte protein PMEL	1.40	NA	NA	2	3.3
Q14956	Transmembrane glycoprotein NMB	1.40	0.083	2.9E-04	3	5.8
Q99536	Synaptic vesicle membrane protein VAT-1 homolog	1.39	0.081	5.0E-04	6	20.4
O60814	Histone H2B type 1-K	1.39	NA	NA	2	7.9
P22392	Nucleoside diphosphate kinase B	1.38	NA	NA	2	10.5
P07339	Cathepsin D	1.38	0.056	1.2E-02	3	7.3
P62424	60S ribosomal protein L7a	1.38	NA	NA	2	9.4
O00148	ATP-dependent RNA helicase DDX39A	1.37	NA	NA	2	5.2
P06744	Glucose-6-phosphate isomerase	1.36	NA	NA	2	4.5
P51572	B-cell receptor-associated protein 31	1.35	0.112	4.8E-02	5	23.2
P01859	Ig gamma-2 chain C region	1.34	0.051	2.4E-03	3	11.0
P08107	Heat shock 70 kDa protein 1A/1B	1.34	0.043	4.5E-06	10	18.3
P10412	Histone H1.4	1.32	0.030	1.4E-08	4	15.5
O14773	Tripeptidyl-peptidase 1	1.31	0.028	2.3E-07	3	7.5
P38646	Stress-70 protein, mitochondrial	1.28	0.086	1.3E-02	8	13.7
P09382	Galectin-1	1.27	0.035	4.8E-06	4	31.9
P69905	Hemoglobin subunit alpha	1.27	0.034	1.6E-05	6	58.5
P45880	Voltage-dependent anion-selective channel protein 2	1.27	0.034	5.1E-03	3	8.8

P04075	Fructose-bisphosphate aldolase A	1.26	0.209	6.2E-02	8	26.9
P10606	Cytochrome c oxidase subunit 5B, mitochondrial	1.24	0.056	3.2E-01	3	24.0
P68871	Hemoglobin subunit beta	1.24	0.220	1.7E-02	6	51.0
P22626	Heterogeneous nuclear ribonucleoproteins A2/B1	1.23	0.044	3.2E-03	8	22.9
P37802	Transgelin-2	1.23	NA	NA	2	10.1
Q8NFI4	Putative protein FAM10A5	1.23	NA	NA	2	4.6
P62258	14-3-3 protein epsilon	1.22	0.538	5.0E-01	3	12.2
Q14103	Heterogeneous nuclear ribonucleoprotein D0	1.21	NA	NA	2	6.2
P15880	40S ribosomal protein S2	1.20	NA	NA	2	7.8
P60953	Cell division control protein 42 homolog	1.20	NA	NA	2	9.9
Q9UHQ9	NADH-cytochrome b5 reductase 1	1.18	0.266	4.5E-01	4	16.1
P09497	Clathrin light chain B	1.17	NA	NA	2	8.3
P11216	Glycogen phosphorylase, brain form	1.17	NA	NA	2	3.0
P12236	ADP/ATP translocase 3	1.15	0.095	9.0E-02	8	23.2
P0C0S5	Histone H2A.Z	1.14	NA	NA	2	18.8
Q5VTE0	Putative elongation factor 1-alpha-like 3	1.14	0.155	1.4E-01	4	7.8
Q99623	Prohibitin-2	1.12	0.185	3.1E-01	4	18.1
P00505	Aspartate aminotransferase, mitochondrial	1.12	NA	NA	2	6.0
P0CG47	Polyubiquitin-B	1.11	0.280	1.1E-01	6	27.1
P49411	Elongation factor Tu, mitochondrial	1.09	0.588	7.3E-01	5	14.2
P06703	Protein S100-A6	1.09	NA	NA	2	16.7
P01857	Ig gamma-1 chain C region	1.08	0.196	1.8E-01	7	30.3
P27797	Calreticulin	1.06	NA	NA	2	5.8
P06576	ATP synthase subunit beta, mitochondrial	1.05	0.130	2.6E-01	11	29.5
P0CG05	Ig lambda-2 chain C regions	1.05	NA	NA	2	23.6
P51991	Heterogeneous nuclear ribonucleoprotein A3	1.05	0.196	5.5E-01	4	14.3
P02647	Apolipoprotein A-I	1.04	0.438	6.7E-01	3	11.6
P61978	Heterogeneous nuclear ribonucleoprotein K	1.03	0.608	7.9E-01	3	8.0
P25705	ATP synthase subunit alpha, mitochondrial	1.02	0.238	6.5E-01	7	16.8
Q9UHD8	Septin-9	1.02	0.274	8.8E-01	3	6.7
P62701	40S ribosomal protein S4, X isoform	1.02	0.696	7.5E-01	3	9.5
P06753	Tropomyosin alpha-3 chain	1.01	NA	NA	2	8.4
O75964	ATP synthase subunit g, mitochondrial	1.01	NA	NA	2	27.2
P26641	Elongation factor 1-gamma	1.00	NA	NA	2	6.6
P02511	Alpha-crystallin B chain	1.00	0.133	9.6E-01	6	35.4
P52272	Heterogeneous nuclear ribonucleoprotein M	0.98	NA	NA	2	2.7
P13987	CD59 glycoprotein	0.97	NA	NA	2	15.6
Q00325	Phosphate carrier protein, mitochondrial	0.97	1.418	7.9E-01	4	11.3
P12956	X-ray repair cross-complementing protein 6	0.97	0.924	6.9E-01	3	5.6
P61247	40S ribosomal protein S3a	0.94	NA	NA	2	8.3
P02545	Prelamin-A/C	0.92	0.155	7.8E-02	28	45.2
P11142	Heat shock cognate 71 kDa protein	0.92	0.054	1.3E-01	6	11.0
P04179	Superoxide dismutase [Mn], mitochondrial	0.92	NA	NA	2	9.9
P30048	Thioredoxin-dependent peroxide reductase, mitochondrial	0.90	NA	NA	2	10.2
Q9H361	Polyadenylate-binding protein 3	0.89	1.045	7.6E-01	4	8.6
P67936	Tropomyosin alpha-4 chain	0.89	0.232	3.3E-01	3	13.7
P27824	Calnexin	0.89	0.456	1.9E-01	8	17.4
Q9Y3U8	60S ribosomal protein L36	0.86	NA	NA	2	21.9
O75367	Core histone macro-H2A.1	0.86	0.109	2.3E-01	3	11.8
O43707	Alpha-actinin-4	0.85	NA	NA	2	3.1
P01009	Alpha-1-antitrypsin	0.85	0.055	7.9E-03	6	15.8
P68371	Tubulin beta-4B chain	0.84	0.050	6.2E-03	3	10.6
P18124	60S ribosomal protein L7	0.84	NA	NA	2	8.5
P11021	78 kDa glucose-regulated protein	0.84	0.085	4.9E-02	12	21.1
Q15149	Plectin	0.84	0.143	1.6E-01	6	1.1
P13671	Complement component C6	0.83	NA	NA	2	1.8
P17858	ATP-dependent 6-phosphofructokinase, liver type	0.83	NA	NA	2	4.5
P62805	Histone H4	0.82	0.038	2.3E-05	7	52.4
P07437	Tubulin beta chain	0.79	0.078	1.6E-02	3	10.6
P09525	Annexin A4	0.79	0.068	2.9E-02	3	6.9
P06396	Gelsolin	0.78	0.086	2.8E-02	4	5.5
O00264	Membrane-associated progesterone receptor component 1	0.77	0.089	8.3E-02	3	15.4
P24539	ATP synthase F(0) complex subunit B1, mitochondrial	0.77	NA	NA	2	9.0
Q16181	Septin-7	0.77	NA	NA	2	4.3
P26038	Moesin	0.77	0.112	1.1E-02	7	12.5
Q16695	Histone H3.1t	0.73	0.048	6.2E-04	4	21.3
P22695	Cytochrome b-c1 complex subunit 2, mitochondrial	0.71	0.081	2.2E-02	3	10.8
P55072	Transitional endoplasmic reticulum ATPase	0.70	0.052	3.3E-03	3	4.0
Q00610	Clathrin heavy chain 1	0.69	0.098	2.8E-02	5	3.8
Q03252	Lamin-B2	0.67	NA	NA	2	2.8
Q9NQC3	Reticulon-4	0.66	NA	NA	2	2.3
P06899	Histone H2B type 1-J	0.66	NA	NA	2	7.9
P08133	Annexin A6	0.65	0.052	1.9E-08	18	30.6
P17931	Galectin-3	0.64	0.058	5.3E-06	4	21.2
Q05682	Caldesmon	0.64	NA	NA	2	2.5
P00387	NADH-cytochrome b5 reductase 3	0.63	NA	NA	2	11.6
Q9BQE3	Tubulin alpha-1C chain	0.63	0.037	3.4E-10	11	27.2
Q09666	Neuroblast differentiation-associated protein AHNAK	0.63	0.031	2.2E-10	8	0.8
Q16555	Dihydropyrimidinase-related protein 2	0.62	0.086	9.9E-03	5	12.9
O75131	Copine-3	0.60	NA	NA	2	3.4
P01024	Complement C3	0.58	NA	NA	2	1.2
P09493	Tropomyosin alpha-1 chain	0.57	NA	NA	2	8.8
P02679	Fibrinogen gamma chain	0.57	0.728	3.5E-01	3	8.2
P07099	Epoxide hydrolase 1	0.56	NA	NA	2	5.9
P14625	Endoplasmin	0.55	0.008	4.0E-04	5	7.0
P60660	Myosin light polypeptide 6	0.54	0.038	4.0E-10	7	58.3
P11047	Laminin subunit gamma-1	0.49	NA	NA	2	1.7
P02675	Fibrinogen beta chain	0.47	NA	NA	2	4.5
O14950	Myosin regulatory light chain 12B	0.45	NA	NA	2	12.2
P60709	Actin, cytoplasmic 1	0.45	0.042	1.6E-06	4	21.1
P21333	Filamin-A	0.45	0.112	1.0E-05	15	8.1
P07355	Annexin A2	0.44	0.034	0.0E+00	18	58.1
Q13813	Spectrin alpha chain, non-erythrocytic 1	0.44	0.056	1.6E-11	23	12.8
P35579	Myosin-9	0.44	0.068	3.1E-07	22	12.6
P68032	Actin, alpha cardiac muscle 1	0.43	NA	NA	2	12.2
P22748	Carbonic anhydrase 4	0.43	NA	NA	2	6.4
Q6NZI2	Polymerase I and transcript release factor	0.38	NA	NA	2	7.4
Q14195	Dihydropyrimidinase-related protein 3	0.28	NA	NA	2	4.6
P39060	Collagen alpha-1(XVIII) chain	0.28	NA	NA	2	1.5
O15230	Laminin subunit alpha-5	0.27	NA	NA	2	0.9
P36269	Gamma-glutamyltransferase 5	0.27	NA	NA	2	4.1
P02760	Protein AMBP	0.26	NA	NA	2	7.1
P21926	CD9 antigen	0.25	NA	NA	2	7.5
P04275	von Willebrand factor	0.25	0.195	5.8E-02	3	1.2
P02452	Collagen alpha-1(I) chain	0.08	NA	NA	2	2.2
P12109	Collagen alpha-1(VI) chain	0.43	0.145	8.5E-04	3	4.1
Q01082	Spectrin beta chain, non-erythrocytic 1	0.42	0.080	5.6E-07	11	5.5
P04899	Guanine nucleotide-binding protein G(i) subunit alpha-2	0.41	0.168	1.5E-02	3	9.9
P02649	Apolipoprotein E	0.38	0.075	7.8E-08	10	34.1
Q9Y490	Talin-1	0.38	0.081	1.1E-03	3	1.1

P12111	Collagen alpha-3(VI) chain	0.37	0.135	2.0E-03	4	1.5
P32119	Peroxiredoxin-2	0.37	0.169	7.8E-03	3	17.7
P01011	Alpha-1-antichymotrypsin	0.36	0.079	2.3E-03	3	6.6
P01871	Ig mu chain C region	0.33	0.115	7.4E-06	5	14.8
P04083	Annexin A1	0.32	0.166	6.3E-04	8	23.1
P98160	Basement membrane-specific heparan sulfate proteoglycan core protein	0.29	0.159	1.2E-04	9	2.7
P02749	Beta-2-glycoprotein 1	0.29	0.120	5.3E-03	3	9.3
P02730	Band 3 anion transport protein	0.29	0.123	1.3E-02	3	4.5
P35749	Myosin-11	0.27	0.118	1.2E-04	8	5.1
P22352	Glutathione peroxidase 3	0.25	0.090	1.2E-02	3	16.4
Q15661	Tryptase alpha/beta-1	0.23	0.151	3.6E-05	4	15.6
P21980	Protein-glutamine gamma-glutamyltransferase 2	0.23	0.132	1.2E-05	8	12.2
P51888	Prolargin	0.22	0.052	0.0E+00	10	30.9
P35555	Fibrillin-1	0.18	0.066	0.0E+00	21	7.6
P04004	Vitronectin	0.18	0.110	6.3E-09	7	16.1
P35625	Metalloproteinase inhibitor 3	0.18	0.137	1.9E-04	4	15.6
P02671	Fibrinogen alpha chain	0.18	0.142	3.8E-03	3	4.3
P51884	Lumican	0.16	0.068	0.0E+00	9	29.0
P20774	Mimecan	0.16	0.130	2.1E-08	5	14.1
P25189	Myelin protein P0	0.15	0.095	6.1E-10	5	17.7
P21810	Biglycan	0.15	0.080	0.0E+00	9	30.7
P02748	Complement component C9	0.14	0.069	5.5E-06	4	7.9
P10909	Clusterin	0.14	0.082	4.2E-14	8	17.6
P02743	Serum amyloid P-component	0.11	0.211	3.6E-04	3	13.0
P08123	Collagen alpha-2(I) chain	0.10	0.226	7.1E-05	3	3.2

Brown denotes change ≥ 2 standard deviations (SD) from the mean, yellow denotes change ≥ 1 SD and green highlights p values ≤ 0.05. NA, not applicable, n<3 unique peptides.
