# Supplementary material for: iTRAQ Quantitative Proteomic Comparison of Metastatic and Non-Metastatic Uveal Melanoma Tumors
Source: PLoS One. 2015 Aug 25;10(8):e0135543. doi: 10.1371/journal.pone.0135543 (PMC4549237; doi:10.1371/journal.pone.0135543)
Supplement: S12 Table — (PDF) [file pone.0135543.s012.pdf]

Supplementary Table S12
Relative Protein Abundance: Sample UM09, Metastatic
Total Proteins Quantified = 250; LogMedian Protein Ratio = 0.14; LogMean Protein Ratio = 0; Standard Deviation = 0.81

Uni-Prot Accession	Protein	Ratio UM/Control	Standard Deviation	*p* value	Unique Peptides	% Sequence Coverage
P14174	Macrophage migration inhibitory factor	8.67	0.107	2.6E-10	2	17.4
P06454	Prothymosin alpha	6.11	0.133	1.5E-02	2	12.6
P62937	Peptidyl-prolyl cis-trans isomerase A	4.67	0.095	4.4E-16	8	41.8
O15400	Syntaxin-7	3.33	0.171	2.1E-02	3	13.4
P30086	Phosphatidylethanolamine-binding protein 1	3.31	0.090	1.4E-07	6	34.2
P17096	High mobility group protein HMG-I/HMG-Y	3.28	0.110	1.3E-08	2	23.4
P09651	Heterogeneous nuclear ribonucleoprotein A1	3.28	0.105	4.6E-14	6	18.5
P02768	Serum albumin	3.26	0.023	0.0E+00	34	48.8
P00338	L-lactate dehydrogenase A chain	3.18	0.082	5.9E-05	4	13.0
P00558	Phosphoglycerate kinase 1	3.17	0.130	1.7E-05	7	20.1
P06748	Nucleophosmin	3.09	0.077	3.8E-10	7	23.8
P63104	14-3-3 protein zeta/delta	2.80	0.189	1.6E-02	5	26.1
P06733	Alpha-enolase	2.73	0.100	9.1E-08	8	19.8
P16070	CD44 antigen	2.71	0.195	9.4E-04	8	12.0
P07900	Heat shock protein HSP 90-alpha	2.70	0.170	3.3E-03	4	5.5
P22392	Nucleoside diphosphate kinase B	2.70	0.157	1.5E-04	4	30.3
P60174	Triosephosphate isomerase	2.66	0.206	1.0E-03	10	36.4
P52565	Rho GDP-dissociation inhibitor 1	2.63	0.142	1.4E-03	3	15.2
P08758	Annexin A5	2.62	0.065	1.6E-14	13	45.3
P09429	High mobility group protein B1	2.54	0.053	3.4E-13	4	27.0
P04406	Glyceraldehyde-3-phosphate dehydrogenase	2.45	0.052	7.8E-07	13	43.6
P07195	L-lactate dehydrogenase B chain	2.41	0.121	7.6E-04	3	9.3
Q13510	Acid ceramidase	2.38	0.253	1.8E-02	3	6.8
P16403	Histone H1.2	2.38	0.038	0.0E+00	7	17.8
P63241	Eukaryotic translation initiation factor 5A-1	2.36	0.129	1.1E-02	2	13.0
P16401	Histone H1.5	2.34	0.105	4.8E-05	3	14.6
P08195	4F2 cell-surface antigen heavy chain	2.31	0.182	3.0E-02	4	9.5
Q8IVF2	Protein AHNAK2	2.29	0.225	1.5E-02	2	0.4
P10599	Thioredoxin	2.27	0.084	5.3E-05	3	28.6
P19338	Nucleolin	2.25	0.092	1.5E-04	10	13.9
Q9BV36	Melanophilin	2.77	0.544	NA	2	4.7
P07686	Beta-hexosaminidase subunit beta	2.74	0.537	NA	2	2.9
P37802	Transgelin-2	2.18	0.073	2.1E-04	3	15.1
P62158	Calmodulin	2.17	0.047	4.9E-08	2	22.1
P78417	Glutathione S-transferase omega-1	2.16	0.169	5.3E-03	3	13.3
P40926	Malate dehydrogenase, mitochondrial	2.12	0.052	1.1E-10	10	36.4
P23528	Cofilin-1	2.09	0.102	8.3E-04	3	18.1
P07910	Heterogeneous nuclear ribonucleoproteins C1/C2	2.06	0.080	5.6E-04	4	12.1
Q99798	Aconitate hydratase, mitochondrial	2.05	0.070	1.7E-03	4	7.1
P08670	Vimentin	2.04	0.039	0.0E+00	30	60.3
P14618	Pyruvate kinase PKM	2.02	0.174	3.6E-03	8	14.5
Q08380	Galectin-3-binding protein	2.02	0.174	NA	2	5.6
Q5VTE0	Putative elongation factor 1-alpha-like 3	1.99	0.058	5.0E-09	7	14.1
Q99536	Synaptic vesicle membrane protein VAT-1 homolog	1.98	0.049	2.6E-14	8	23.9
O14556	Glyceraldehyde-3-phosphate dehydrogenase, testis-specific	1.98	0.190	1.3E-02	3	9.8
P29401	Transketolase	1.97	0.170	9.7E-03	4	6.4
P63244	Guanine nucleotide-binding protein subunit beta-2-like 1	1.96	0.063	5.8E-05	3	10.4
P02787	Serotransferrin	1.94	0.178	6.5E-03	6	8.5
P0DME0	Protein SETSIP	1.93	0.075	2.7E-02	2	5.3
P60842	Eukaryotic initiation factor 4A-I	1.90	0.160	5.8E-03	3	10.8
P23526	Adenosylhomocysteinase	1.89	0.064	4.9E-06	2	6.5
P08238	Heat shock protein HSP 90-beta	1.87	0.434	NA	2	3.2
Q12906	Interleukin enhancer-binding factor 3	1.86	0.083	2.1E-02	2	3.0
P04075	Fructose-bisphosphate aldolase A	1.83	0.090	2.7E-04	12	39.0
P61604	10 kDa heat shock protein, mitochondrial	1.81	0.071	2.5E-05	4	29.4
P05387	60S acidic ribosomal protein P2	1.79	0.145	4.2E-03	3	25.2
P08865	40S ribosomal protein SA	1.76	0.107	9.1E-02	3	15.3
Q13185	Chromobox protein homolog 3	1.74	0.072	4.4E-03	2	14.8
P21796	Voltage-dependent anion-selective channel protein 1	1.69	0.110	5.1E-03	6	22.3
P61978	Heterogeneous nuclear ribonucleoprotein K	1.67	0.086	2.2E-02	4	10.8
O60814	Histone H2B type 1-K	1.66	0.025	0.0E+00	2	7.9
P22626	Heterogeneous nuclear ribonucleoproteins A2/B1	1.65	0.053	2.3E-08	9	22.9
P02545	Prelamin-A/C	1.65	0.030	0.0E+00	34	48.5
P07339	Cathepsin D	1.65	0.095	5.7E-03	4	9.2
Q12905	Interleukin enhancer-binding factor 2	1.63	0.138	3.1E-02	2	6.7
Q14956	Transmembrane glycoprotein NMB	1.62	0.076	2.9E-04	3	6.5
P30044	Peroxiredoxin-5, mitochondrial	1.61	0.094	2.8E-03	2	10.3
P06744	Glucose-6-phosphate isomerase	1.60	0.203	1.0E-01	5	9.5
P23284	Peptidyl-prolyl cis-trans isomerase B	1.59	0.100	3.6E-04	9	32.4
P61026	Ras-related protein Rab-10	1.59	0.081	4.6E-02	2	11.5
Q14103	Heterogeneous nuclear ribonucleoprotein D0	1.58	0.073	4.6E-04	2	6.2
P06753	Tropomyosin alpha-3 chain	1.57	15.190	2.3E-01	2	8.4
P27816	Microtubule-associated protein 4	1.57	0.171	1.1E-01	5	5.4
P27635	60S ribosomal protein L10	1.57	0.034	2.2E-04	2	9.8
Q14697	Neutral alpha-glucosidase AB	1.57	0.092	3.4E-03	3	4.9
Q00839	Heterogeneous nuclear ribonucleoprotein U	1.56	0.239	4.0E-01	4	6.3
P40967	Melanocyte protein PMEL	1.53	0.102	8.0E-05	2	3.3
P54709	Sodium/potassium-transporting ATPase subunit beta-3	1.53	0.130	5.1E-02	2	10.4
Q13011	Delta(3,5)-Delta(2,4)-dienoyl-CoA isomerase, mitochondrial	1.50	0.098	2.7E-02	2	5.8
P62249	40S ribosomal protein S16	1.49	0.123	NA	2	15.1
P62851	40S ribosomal protein S25	1.49	0.119	2.2E-02	3	24.0
P11216	Glycogen phosphorylase, brain form	1.48	0.315	3.6E-01	2	3.0
P26373	60S ribosomal protein L13	1.47	0.668	3.2E-01	4	20.4
P39687	Acidic leucine-rich nuclear phosphoprotein 32 family member A	1.46	0.091	8.3E-04	2	9.6

P11142	Heat shock cognate 71 kDa protein	1.45	0.080	2.7E-04	9	16.9
P51991	Heterogeneous nuclear ribonucleoprotein A3	1.45	0.092	1.1E-03	4	14.3
P31040	Succinate dehydrogenase [ubiquinone] flavoprotein subunit, mitochondrial	1.44	0.108	6.0E-02	2	3.2
P26641	Elongation factor 1-gamma	1.44	0.110	1.7E-02	4	12.1
P39019	40S ribosomal protein S19	1.42	0.064	3.2E-03	3	17.2
P46777	60S ribosomal protein L5	1.41	0.097	NA	2	8.8
P18669	Phosphoglycerate mutase 1	1.41	0.046	1.5E-04	2	15.4
P62753	40S ribosomal protein S6	1.40	0.049	NA	2	8.8
P12956	X-ray repair cross-complementing protein 6	1.39	0.109	3.6E-02	6	11.0
P15880	40S ribosomal protein S2	1.39	0.110	1.4E-02	3	11.6
P38646	Stress-70 protein, mitochondrial	1.37	0.146	7.8E-03	9	17.2
P20700	Lamin-B1	1.36	0.119	1.3E-01	2	3.2
P13639	Elongation factor 2	1.36	0.443	3.4E-01	2	3.4
P62857	40S ribosomal protein S28	1.36	0.381	NA	2	30.4
P22087	rRNA 2'-O-methyltransferase fibrillarin	1.34	0.067	8.8E-02	2	10.3
Q13423	NAD(P) transhydrogenase, mitochondrial	1.33	0.214	NA	2	1.6
Q15084	Protein disulfide-isomerase A6	1.33	0.022	1.2E-02	2	7.3
P07237	Protein disulfide-isomerase	1.30	0.076	5.1E-03	6	13.4
P62701	40S ribosomal protein S4, X isoform	1.30	0.109	6.6E-02	2	5.7
P30101	Protein disulfide-isomerase A3	1.29	0.070	1.7E-03	10	22.4
O15173	Membrane-associated progesterone receptor component 2	1.29	0.596	NA	2	14.8
P25398	40S ribosomal protein S12	1.28	0.245	1.1E-01	3	25.0
O60506	Heterogeneous nuclear ribonucleoprotein Q	1.27	0.074	1.2E-01	2	2.9
P49411	Elongation factor Tu, mitochondrial	1.26	0.079	1.3E-02	5	13.7
P51149	Ras-related protein Rab-7a	1.26	0.016	5.1E-03	2	9.7
P0C0S8	Histone H2A type 1	1.26	0.047	2.2E-03	2	23.1
P09525	Annexin A4	1.26	0.097	5.0E-02	3	6.9
P99999	Cytochrome c	1.24	0.356	4.3E-01	2	21.9
P17643	5,6-dihydroxyindole-2-carboxylic acid oxidase	1.23	0.475	4.4E-01	5	9.5
O75367	Core histone macro-H2A.1	1.22	0.599	1.2E-01	5	19.1
P30049	ATP synthase subunit delta, mitochondrial	1.22	0.323	3.6E-01	2	13.7
P10809	60 kDa heat shock protein, mitochondrial	1.22	0.411	3.8E-01	3	7.7
P27824	Calnexin	1.21	0.064	9.4E-04	9	17.1
P09382	Galectin-1	1.20	0.051	1.2E-03	4	31.9
P52272	Heterogeneous nuclear ribonucleoprotein M	1.19	0.082	8.3E-02	2	2.7
P61247	40S ribosomal protein S3a	1.19	7.754	6.9E-01	3	11.7
O43707	Alpha-actinin-4	1.18	0.074	1.3E-03	11	17.2
P04439	HLA class I histocompatibility antigen, A-3 alpha chain	1.17	1.510	NA	2	6.8
P67936	Tropomyosin alpha-4 chain	1.16	0.068	2.9E-01	3	12.1
P45880	Voltage-dependent anion-selective channel protein 2	1.16	0.115	1.1E-01	4	15.0
P18124	60S ribosomal protein L7	1.15	41.991	NA	2	12.5
P0CG47	Polyubiquitin-B	1.15	0.319	1.7E-02	7	29.7
P62906	60S ribosomal protein L10a	1.14	0.164	3.3E-01	3	16.1
P14314	Glucosidase 2 subunit beta	1.13	0.189	5.3E-01	5	9.7
P25705	ATP synthase subunit alpha, mitochondrial	1.13	0.284	3.0E-01	7	17.5
P30041	Peroxiredoxin-6	1.13	0.258	5.3E-01	4	16.1
Q71DI3	Histone H3.2	1.10	0.319	7.1E-02	3	16.2
P0CG05	Ig lambda-2 chain C regions	1.10	0.474	2.4E-01	2	23.6
Q9UHD8	Septin-9	1.10	0.131	3.2E-01	2	4.1
Q8NBS9	Thioredoxin domain-containing protein 5	1.10	0.036	2.2E-01	2	4.4
P11940	Polyadenylate-binding protein 1	1.09	0.038	1.4E-01	2	4.7
Q86UP2	Kinectin	1.09	4.061	NA	2	1.8
P62805	Histone H4	1.08	0.065	6.2E-02	7	58.3
P50502	Hsc70-interacting protein	1.08	0.096	3.2E-01	2	6.5
Q15233	Non-POU domain-containing octamer-binding protein	1.07	1.524	7.6E-01	2	5.1
P35232	Prohibitin	1.07	0.252	4.2E-01	7	28.3
O14773	Tripeptidyl-peptidase 1	1.06	0.297	9.2E-02	3	7.5
P26038	Moesin	1.05	0.088	2.2E-01	6	6.4
Q99584	Protein S100-A13	1.05	1.311	3.5E-01	2	23.5
P51572	B-cell receptor-associated protein 31	1.04	0.555	6.9E-01	5	23.2
P13987	CD59 glycoprotein	1.03	0.234	8.3E-01	2	15.6
P06576	ATP synthase subunit beta, mitochondrial	1.02	0.299	7.5E-01	15	35.2
P17858	ATP-dependent 6-phosphofructokinase, liver type	1.01	0.637	9.1E-01	2	4.5
P0C0S5	Histone H2A.Z	1.01	0.392	8.4E-01	2	18.8
P02652	Apolipoprotein A-II	0.99	1.051	9.7E-01	2	11.0
P01859	Ig gamma-2 chain C region	0.98	0.039	5.5E-01	2	6.1
P31930	Cytochrome b-c1 complex subunit 1, mitochondrial	0.97	1.741	9.6E-01	2	6.5
P23396	40S ribosomal protein S3	0.96	0.181	7.5E-01	3	11.9
P62841	40S ribosomal protein S15	0.96	0.525	8.9E-01	2	9.0
Q03252	Lamin-B2	0.96	0.483	8.7E-01	4	5.8
P05141	ADP/ATP translocase 2	0.95	0.353	6.3E-01	6	18.1
P02511	Alpha-crystallin B chain	0.94	0.223	5.0E-01	5	29.1
P55072	Transitional endoplasmic reticulum ATPase	0.93	0.377	4.2E-01	3	4.6
P09493	Tropomyosin alpha-1 chain	0.93	1.386	7.9E-01	3	12.0
P14625	Endoplasmin	0.92	0.103	3.0E-01	7	9.0
P06899	Histone H2B type 1-J	0.92	0.054	9.8E-02	2	7.9
P01024	Complement C3	0.90	0.236	5.8E-01	3	2.0
Q16181	Septin-7	0.90	0.134	5.3E-01	2	4.3
P04792	Heat shock protein beta-1	0.89	0.150	1.2E-01	6	33.2
P08107	Heat shock 70 kDa protein 1A/1B	0.89	0.064	5.2E-02	9	16.5
Q02952	A-kinase anchor protein 12	0.89	0.220	3.2E-01	8	6.2
P11021	78 kDa glucose-regulated protein	0.88	0.107	2.0E-01	10	18.5
P07437	Tubulin beta chain	0.88	0.060	4.2E-03	4	13.3
P27797	Calreticulin	0.88	0.369	4.6E-01	2	6.7
Q00325	Phosphate carrier protein, mitochondrial	0.87	0.497	5.9E-01	2	7.2
P40939	Trifunctional enzyme subunit alpha, mitochondrial	0.87	0.011	NA	2	4.3
P04843	Dolichyl-diphosphooligosaccharide--protein glycosyltransferase subunit 1	0.87	0.895	6.7E-01	2	4.1
P01009	Alpha-1-antitrypsin	0.86	0.161	3.8E-01	4	11.5
P68371	Tubulin beta-4B chain	0.86	0.055	6.1E-03	3	10.6
P60709	Actin, cytoplasmic 1	0.84	0.041	2.7E-02	6	24.8

P17931	Galectin-3	0.84	0.061	1.1E-02	5	24.0
Q06830	Peroxiredoxin-1	0.83	0.049	4.2E-04	7	36.7
O14950	Myosin regulatory light chain 12B	0.82	0.071	8.8E-02	2	12.2
Q00610	Clathrin heavy chain 1	0.80	0.031	2.6E-05	8	6.1
Q9BQE3	Tubulin alpha-1C chain	0.80	0.167	1.2E-01	5	13.8
P22695	Cytochrome b-c1 complex subunit 2, mitochondrial	0.78	0.153	7.4E-02	2	7.7
P68032	Actin, alpha cardiac muscle 1	0.77	0.113	4.4E-02	2	12.2
P13010	X-ray repair cross-complementing protein 5	0.75	0.549	4.5E-01	2	3.0
P00387	NADH-cytochrome b5 reductase 3	0.75	0.048	9.4E-04	4	12.0
P61769	Beta-2-microglobulin	0.75	0.105	1.2E-01	2	16.8
P35580	Myosin-10	0.74	0.304	4.5E-01	3	2.0
Q15019	Septin-2	0.73	0.135	9.2E-02	2	7.5
P07099	Epoxide hydrolase 1	0.72	0.350	3.3E-01	2	5.9
P60660	Myosin light polypeptide 6	0.72	0.025	2.8E-12	7	58.3
P01857	Ig gamma-1 chain C region	0.72	0.055	1.6E-03	5	21.2
Q9NQC3	Reticulon-4	0.71	0.050	3.0E-03	2	2.3
P08133	Annexin A6	0.70	0.076	1.1E-04	16	26.3
P01834	Ig kappa chain C region	0.69	0.082	1.4E-02	2	33.0
P35579	Myosin-9	0.68	0.045	8.9E-09	36	20.5
P02647	Apolipoprotein A-I	0.68	0.084	6.3E-02	2	7.1
P12814	Alpha-actinin-1	0.64	0.066	1.2E-03	4	5.5
P05023	Sodium/potassium-transporting ATPase subunit alpha-1	0.63	0.112	2.4E-02	6	5.7
P12111	Collagen alpha-3(VI) chain	0.60	0.062	1.7E-02	2	0.7
P69905	Hemoglobin subunit alpha	0.59	0.035	8.7E-06	5	38.0
P05556	Integrin beta-1	0.56	0.117	6.4E-02	2	3.3
Q15149	Plectin	0.56	0.215	2.8E-01	6	1.5
P07355	Annexin A2	0.55	0.026	0.0E+00	23	60.8
P32119	Peroxiredoxin-2	0.54	0.176	1.2E-02	4	22.2
Q9Y490	Talin-1	0.53	0.148	1.0E-01	5	2.4
P21333	Filamin-A	0.53	0.036	1.8E-15	26	12.9
Q01082	Spectrin beta chain, non-erythrocytic 1	0.53	0.038	2.1E-14	17	9.1
P07305	Histone H1.0	0.52	0.196	5.8E-02	2	10.8
Q09666	Neuroblast differentiation-associated protein AHNAK	0.52	0.023	1.3E-10	7	0.8
Q13813	Spectrin alpha chain, non-erythrocytic 1	0.51	0.043	2.2E-16	27	13.2
P12109	Collagen alpha-1(VI) chain	0.51	0.088	5.2E-03	4	5.1
O15230	Laminin subunit alpha-5	0.50	0.072	6.3E-04	2	1.0
P63096	Guanine nucleotide-binding protein G(i) subunit alpha-1	0.50	0.174	6.3E-02	2	5.9
P68871	Hemoglobin subunit beta	0.49	0.023	0.0E+00	11	78.2
P02679	Fibrinogen gamma chain	0.45	0.478	1.8E-01	2	5.7
P11047	Laminin subunit gamma-1	0.38	0.484	2.9E-01	3	2.6
P18206	Vinculin	0.37	0.575	NA	2	2.2
P02462	Collagen alpha-1(IV) chain	0.37	0.296	NA	2	1.7
P39060	Collagen alpha-1(XVIII) chain	0.27	0.190	6.8E-02	3	2.1
P02730	Band 3 anion transport protein	0.24	0.184	2.8E-01	2	2.3
O43491	Band 4.1-like protein 2	0.23	0.550	NA	2	2.2
P01011	Alpha-1-antichymotrypsin	0.42	0.102	5.4E-04	3	7.1
P22748	Carbonic anhydrase 4	0.41	0.068	7.9E-03	2	6.4
P02749	Beta-2-glycoprotein 1	0.40	0.115	1.1E-02	4	13.3
P06396	Gelsolin	0.39	0.051	2.0E-08	6	7.9
Q16555	Dihydropyrimidinase-related protein 2	0.38	0.226	6.9E-03	4	7.3
P01871	Ig mu chain C region	0.36	0.190	4.8E-05	4	13.1
P02675	Fibrinogen beta chain	0.34	0.214	2.7E-03	2	4.3
P14543	Nidogen-1	0.32	0.096	1.1E-02	2	2.2
P36269	Gamma-glutamyltransferase 5	0.31	0.238	3.2E-02	2	4.1
P04083	Annexin A1	0.29	0.099	2.1E-06	6	18.2
P02649	Apolipoprotein E	0.27	0.100	3.3E-06	9	33.8
P63211	Guanine nucleotide-binding protein G(T) subunit gamma-T1	0.26	0.091	3.8E-03	2	17.6
P51888	Prolargin	0.24	0.056	0.0E+00	12	34.0
P98160	Basement membrane-specific heparan sulfate proteoglycan core protein	0.24	0.226	8.2E-04	5	1.5
Q15661	Tryptase alpha/beta-1	0.23	0.215	1.2E-04	4	17.1
P02748	Complement component C9	0.22	0.087	1.6E-04	4	7.9
P35749	Myosin-11	0.21	0.213	2.7E-03	6	4.4
P02760	Protein AMBP	0.21	0.059	4.2E-03	2	7.7
P21980	Protein-glutamine gamma-glutamyltransferase 2	0.18	0.074	4.3E-09	7	12.2
P21926	CD9 antigen	0.18	0.154	2.1E-04	2	7.5
P21810	Biglycan	0.17	0.075	2.2E-16	9	30.7
P51884	Lumican	0.16	0.052	2.2E-16	9	29.0
P20774	Mimecan	0.16	0.141	2.5E-07	5	14.1
P04004	Vitronectin	0.15	0.140	1.3E-07	6	14.6
P35625	Metalloproteinase inhibitor 3	0.15	0.220	2.7E-02	3	12.8
P10909	Clusterin	0.14	0.091	1.5E-11	9	17.6
P02671	Fibrinogen alpha chain	0.14	0.109	1.4E-04	2	2.3
P35555	Fibrillin-1	0.14	0.062	0.0E+00	15	5.4
P25189	Myelin protein P0	0.11	0.179	1.5E-06	5	17.7
P08123	Collagen alpha-2(I) chain	0.06	0.117	6.2E-06	2	2.4
P02743	Serum amyloid P-component	0.04	0.059	3.2E-07	2	8.5

Brown denotes change ≥ 2 standard deviations (SD) from the mean, yellow denotes change ≥ 1 SD and green highlights p values ≤ 0.05. NA, not applicable, n<3 unique peptides.
