# Supplementary material for: iTRAQ Quantitative Proteomic Comparison of Metastatic and Non-Metastatic Uveal Melanoma Tumors
Source: PLoS One. 2015 Aug 25;10(8):e0135543. doi: 10.1371/journal.pone.0135543 (PMC4549237; doi:10.1371/journal.pone.0135543)
Supplement: S13 Table — (PDF) [file pone.0135543.s013.pdf]

Supplementary Table S13
Relative Protein Abundance: Sample UM11, Metastatic
Total Proteins Quantified = 193; LogMedian Protein Ratio = .11; LogMean ProteinRatio = 0; Standard Deviation = 0.70

Uni-Prot Accession	Protein	Ratio UM/Control	Standard Deviation	*p* value	Unique Peptides	% Sequence Coverage
P02768	Serum albumin	4.61	0.035	0.0E+00	33	51.4
P00558	Phosphoglycerate kinase 1	3.91	0.092	6.6E-04	3	7.9
P63104	14-3-3 protein zeta/delta	3.66	0.117	6.1E-04	3	15.5
P62937	Peptidyl-prolyl cis-trans isomerase A	3.21	0.078	1.3E-11	7	41.8
P08670	Vimentin	2.78	0.027	0.0E+00	31	61.6
P06733	Alpha-enolase	2.60	0.070	2.4E-06	7	14.3
P02787	Serotransferrin	2.58	0.103	4.5E-05	3	5.0
P04406	Glyceraldehyde-3-phosphate dehydrogenase	2.57	0.064	6.6E-11	10	36.7
P06748	Nucleophosmin	2.57	0.094	5.0E-05	5	14.6
P08758	Annexin A5	2.53	0.049	0.0E+00	10	35.0
P60174	Triosephosphate isomerase	2.52	0.186	2.4E-02	3	13.6
P07900	Heat shock protein HSP 90-alpha	2.50	0.125	1.4E-03	5	7.7
P10412	Histone H1.4	2.48	0.045	3.8E-15	6	15.5
P07339	Cathepsin D	2.48	0.105	1.2E-05	4	9.2
P14618	Pyruvate kinase PKM	2.48	0.107	1.1E-05	6	11.1
Q13423	NAD(P) transhydrogenase, mitochondrial	2.46	0.037	5.0E-05	4	3.3
P40926	Malate dehydrogenase, mitochondrial	2.29	0.058	2.1E-10	9	32.8
P21796	Voltage-dependent anion-selective channel protein 1	2.25	0.063	4.3E-06	6	22.3
P22392	Nucleoside diphosphate kinase B	2.21	0.137	2.0E-03	3	24.3
P14174	Macrophage migration inhibitory factor	3.96	NA	NA	2	17.4
P00338	L-lactate dehydrogenase A chain	3.33	NA	NA	2	5.4
P21912	Succinate dehydrogenase [ubiquinone] iron-sulfur subunit, mitochondrial	2.15	NA	NA	2	7.1
P07910	Heterogeneous nuclear ribonucleoproteins C1/C2	2.08	NA	NA	2	8.2
P52565	Rho GDP-dissociation inhibitor 1	2.07	NA	NA	2	14.7
P23528	Cofilin-1	2.02	NA	NA	2	10.8
P01859	Ig gamma-2 chain C region	2.02	0.210	1.4E-02	3	11.0
P07195	L-lactate dehydrogenase B chain	2.01	NA	NA	2	6.9
P99999	Cytochrome c	2.00	NA	NA	2	21.9
P55084	Trifunctional enzyme subunit beta, mitochondrial	1.97	NA	NA	2	4.2
P30086	Phosphatidylethanolamine-binding protein 1	1.95	0.094	3.5E-02	5	31.0
P01857	Ig gamma-1 chain C region	1.94	0.054	3.9E-07	5	20.3
Q14103	Heterogeneous nuclear ribonucleoprotein D0	1.93	NA	NA	2	6.2
P30049	ATP synthase subunit delta, mitochondrial	1.93	NA	NA	2	13.7
P06576	ATP synthase subunit beta, mitochondrial	1.93	0.053	1.4E-13	15	34.4
P05141	ADP/ATP translocase 2	1.92	0.068	4.7E-06	6	16.4
Q99798	Aconitate hydratase, mitochondrial	1.91	NA	NA	2	4.0
P17096	High mobility group protein HMG-I/HMG-Y	1.91	NA	NA	2	23.4
P62277	40S ribosomal protein S13	1.90	NA	NA	2	12.6
P61604	10 kDa heat shock protein, mitochondrial	1.88	0.112	1.6E-03	4	29.4
P04075	Fructose-bisphosphate aldolase A	1.83	0.062	1.3E-05	11	36.3
P09651	Heterogeneous nuclear ribonucleoprotein A1	1.81	0.137	2.9E-03	3	9.1
P78417	Glutathione S-transferase omega-1	1.77	0.202	4.1E-02	3	13.3
P09622	Dihydrolipoyl dehydrogenase, mitochondrial	1.75	NA	NA	2	5.1
P45880	Voltage-dependent anion-selective channel protein 2	1.71	0.119	4.1E-03	3	12.9
P01834	Ig kappa chain C region	1.70	NA	NA	2	33.0
P10809	60 kDa heat shock protein, mitochondrial	1.67	0.183	4.1E-02	6	11.5
P14927	Cytochrome b-c1 complex subunit 7	1.66	NA	NA	2	19.8
Q5VTE0	Putative elongation factor 1-alpha-like 3	1.66	0.053	1.1E-06	7	14.1
P11142	Heat shock cognate 71 kDa protein	1.66	0.060	2.4E-06	11	20.6
Q8IVF2	Protein AHNAK2	1.64	NA	NA	2	0.4
Q00325	Phosphate carrier protein, mitochondrial	1.63	0.039	4.2E-04	3	9.7
P62158	Calmodulin	1.59	NA	NA	2	22.1
P25705	ATP synthase subunit alpha, mitochondrial	1.58	0.079	2.3E-04	11	23.9
P04179	Superoxide dismutase [Mn], mitochondrial	1.58	0.089	3.0E-02	4	16.7
P19338	Nucleolin	1.57	NA	NA	2	2.8
P09429	High mobility group protein B1	1.56	0.060	9.1E-05	4	27.0
Q99623	Prohibitin-2	1.56	NA	NA	2	9.4
P30048	Thioredoxin-dependent peroxide reductase, mitochondrial	1.55	NA	NA	2	10.2
P0CG05	Ig lambda-2 chain C regions	1.54	NA	NA	2	23.6
O75964	ATP synthase subunit g, mitochondrial	1.49	0.290	1.7E-01	3	33.0
P38646	Stress-70 protein, mitochondrial	1.49	0.078	5.7E-04	9	17.2
O14773	Tripeptidyl-peptidase 1	1.49	0.080	2.3E-04	4	8.7
P01903	HLA class II histocompatibility antigen, DR alpha chain	1.48	NA	NA	2	9.8
P35232	Prohibitin	1.47	0.171	1.1E-01	6	23.9
Q99536	Synaptic vesicle membrane protein VAT-1 homolog	1.43	0.050	8.9E-06	5	13.2
P62851	40S ribosomal protein S25	1.43	NA	NA	2	12.0
P36957	Dihydrolipoyllysine-residue succinyltransferase component of 2-oxoglutarate dehydrogenase complex, mitochondrial	1.41	0.124	3.5E-02	4	11.0
P30044	Peroxiredoxin-5, mitochondrial	1.40	NA	NA	2	8.9
P49411	Elongation factor Tu, mitochondrial	1.39	0.059	7.1E-03	7	18.4
P22626	Heterogeneous nuclear ribonucleoproteins A2/B1	1.37	0.065	4.8E-04	7	21.5
Q12905	Interleukin enhancer-binding factor 2	1.34	NA	NA	2	6.7
O60814	Histone H2B type 1-K	1.34	NA	NA	2	7.9
P01009	Alpha-1-antitrypsin	1.34	0.046	1.4E-03	4	11.7
P09382	Galectin-1	1.34	0.065	1.5E-04	3	23.0
P40939	Trifunctional enzyme subunit alpha, mitochondrial	1.33	NA	NA	2	2.8
P40967	Melanocyte protein PMEL	1.32	NA	NA	2	3.3
O75367	Core histone macro-H2A.1	1.32	0.107	1.3E-02	5	19.1
P29401	Transketolase	1.31	NA	NA	2	3.4
P24539	ATP synthase F(0) complex subunit B1, mitochondrial	1.31	NA	NA	2	9.0
Q14697	Neutral alpha-glucosidase AB	1.31	0.125	9.4E-02	3	4.8
P30101	Protein disulfide-isomerase A3	1.30	0.046	9.9E-03	8	16.0
P23284	Peptidyl-prolyl cis-trans isomerase B	1.30	0.037	5.1E-05	5	25.5
P61978	Heterogeneous nuclear ribonucleoprotein K	1.30	0.299	3.5E-01	3	6.3
P22695	Cytochrome b-c1 complex subunit 2, mitochondrial	1.25	0.071	4.9E-03	4	13.9
P31930	Cytochrome b-c1 complex subunit 1, mitochondrial	1.23	NA	NA	2	6.5
Q15149	Plectin	1.20	0.159	9.2E-02	10	2.3
P26038	Moesin	1.19	0.152	1.2E-01	7	9.7
P61247	40S ribosomal protein S3a	1.17	0.618	3.8E-01	3	11.4
O15230	Laminin subunit alpha-5	1.16	1.878	2.3E-01	5	2.1
P27797	Calreticulin	1.16	0.053	1.3E-01	3	6.2
P18124	60S ribosomal protein L7	1.15	NA	NA	2	7.7
P12956	X-ray repair cross-complementing protein 6	1.15	0.074	7.3E-02	4	6.6
P07237	Protein disulfide-isomerase	1.15	0.082	8.1E-02	5	9.8
P02545	Prelamin-A/C	1.15	0.059	5.1E-04	25	35.7
P02647	Apolipoprotein A-I	1.14	NA	NA	2	7.1
Q99584	Protein S100-A13	1.13	NA	NA	2	23.5

P10606	Cytochrome c oxidase subunit 5B, mitochondrial	1.11	NA	NA	2	18.6
P11021	78 kDa glucose-regulated protein	1.10	0.022	3.4E-02	5	5.8
P51572	B-cell receptor-associated protein 31	1.09	0.714	6.4E-01	3	13.8
P67936	Tropomyosin alpha-4 chain	1.08	0.338	3.0E-01	5	18.5
Q9NQC3	Reticulon-4	1.06	NA	NA	2	2.3
P09493	Tropomyosin alpha-1 chain	1.04	3.565	7.0E-01	4	12.0
O00264	Membrane-associated progesterone receptor component 1	1.03	NA	NA	2	12.3
Q8NFI4	Putative protein FAM10A5	1.02	NA	NA	2	4.6
Q06830	Peroxiredoxin-1	1.01	20.667	7.6E-01	8	40.7
Q71DI3	Histone H3.2	1.00	1.198	9.8E-01	3	16.2
P63261	Actin, cytoplasmic 2	1.00	0.122	9.6E-01	6	24.8
P08107	Heat shock 70 kDa protein 1A/1B	0.99	0.238	9.5E-01	5	8.9
P68032	Actin, alpha cardiac muscle 1	0.98	0.106	7.3E-01	6	25.5
P35637	RNA-binding protein FUS	0.98	NA	NA	2	6.3
P27824	Calnexin	0.97	0.062	2.1E-01	4	6.8
P55072	Transitional endoplasmic reticulum ATPase	0.96	0.167	6.2E-01	3	5.0
P0C0S5	Histone H2A.Z	0.96	NA	NA	2	18.8
P14625	Endoplasmin	0.95	1.153	5.4E-01	9	11.2
P69905	Hemoglobin subunit alpha	0.94	0.227	3.4E-01	5	38.0
O43707	Alpha-actinin-4	0.93	1.055	6.4E-01	6	9.3
P12814	Alpha-actinin-1	0.92	0.167	3.4E-01	5	6.8
Q03252	Lamin-B2	0.90	0.655	5.7E-01	6	9.5
P17931	Galectin-3	0.90	0.838	3.6E-01	4	21.2
P18206	Vinculin	0.90	0.414	4.6E-01	4	4.7
P02751	Fibronectin	0.89	0.491	5.0E-01	4	2.4
P62805	Histone H4	0.88	0.060	5.4E-03	8	59.2
Q00610	Clathrin heavy chain 1	0.88	0.118	3.5E-01	8	6.4
P06396	Gelsolin	0.82	0.122	8.4E-02	5	6.9
P0CG47	Polyubiquitin-B	0.82	0.046	7.8E-03	5	23.1
P08133	Annexin A6	0.82	0.069	5.3E-04	14	24.1
P06899	Histone H2B type 1-J	0.82	NA	NA	2	7.9
P68871	Hemoglobin subunit beta	0.82	0.087	1.6E-02	12	84.4
P39060	Collagen alpha-1(XVIII) chain	0.81	0.141	1.1E-01	4	3.0
P21333	Filamin-A	0.79	0.083	7.9E-05	25	11.6
P26447	Protein S100-A4	0.78	NA	NA	2	15.8
P35555	Fibrillin-1	0.78	0.038	2.9E-09	27	9.7
P55268	Laminin subunit beta-2	0.77	0.055	2.2E-02	4	2.2
P12111	Collagen alpha-3(VI) chain	0.76	0.047	2.8E-03	5	1.8
P11047	Laminin subunit gamma-1	0.75	0.073	1.7E-02	3	2.3
P04792	Heat shock protein beta-1	0.73	0.075	1.8E-03	6	36.1
P35749	Myosin-11	0.72	0.119	3.5E-02	12	7.3
P50993	Sodium/potassium-transporting ATPase subunit alpha-2	0.71	0.083	2.8E-02	3	3.7
P08572	Collagen alpha-2(IV) chain	0.71	0.213	2.1E-01	3	2.5
P24844	Myosin regulatory light polypeptide 9	0.68	NA	NA	2	12.2
P14543	Nidogen-1	0.67	0.171	9.3E-02	3	3.0
P12110	Collagen alpha-2(VI) chain	0.67	0.102	1.0E-01	3	3.2
P98160	Basement membrane-specific heparan sulfate proteoglycan core protein	0.66	0.097	7.9E-03	9	2.6
P60660	Myosin light polypeptide 6	0.65	0.045	3.2E-07	7	58.3
P05556	Integrin beta-1	0.63	NA	NA	2	3.3
Q09666	Neuroblast differentiation-associated protein AHNAK	0.63	0.047	3.7E-07	7	0.6
P32119	Peroxiredoxin-2	0.61	0.062	2.6E-03	5	27.3
P12109	Collagen alpha-1(VI) chain	0.60	0.107	5.5E-03	4	4.7
Q01082	Spectrin beta chain, non-erythrocytic 1	0.59	0.054	3.9E-07	9	5.2
P07437	Tubulin beta chain	0.58	NA	NA	2	7.2
P02511	Alpha-crystallin B chain	0.58	NA	NA	2	12.6
Q9Y490	Talin-1	0.58	0.091	2.0E-03	6	2.6
Q9Y6C2	EMILIN-1	0.58	NA	NA	2	2.9
O14950	Myosin regulatory light chain 12B	0.56	NA	NA	2	12.2
Q13813	Spectrin alpha chain, non-erythrocytic 1	0.55	0.059	2.0E-10	22	11.7
P68371	Tubulin beta-4B chain	0.54	0.060	3.3E-05	3	10.6
P02749	Beta-2-glycoprotein 1	0.54	0.055	6.3E-04	3	9.3
P35579	Myosin-9	0.52	0.090	7.6E-05	18	9.3
P51884	Lumican	0.51	0.025	0.0E+00	9	29.0
P07099	Epoxide hydrolase 1	0.45	NA	NA	2	4.0
P01011	Alpha-1-antichymotrypsin	0.44	NA	NA	2	4.0
Q02952	A-kinase anchor protein 12	0.42	NA	NA	2	1.5
P80723	Brain acid soluble protein 1	0.42	NA	NA	2	11.9
P22352	Glutathione peroxidase 3	0.41	NA	NA	2	8.0
P07585	Decorin	0.41	NA	NA	2	6.7
Q14195	Dihydropyrimidinase-related protein 3	0.39	0.116	7.1E-02	3	7.2
P04275	von Willebrand factor	0.38	NA	NA	2	0.7
P02730	Band 3 anion transport protein	0.27	NA	NA	2	2.3
P08123	Collagen alpha-2(I) chain	0.20	NA	NA	2	2.4
P02452	Collagen alpha-1(I) chain	0.18	NA	NA	2	2.2
P01024	Complement C3	0.48	0.091	6.9E-03	4	2.4
P51888	Prolargin	0.47	0.030	0.0E+00	13	37.4
P04899	Guanine nucleotide-binding protein G(i) subunit alpha-2	0.47	0.003	5.0E-05	3	9.9
P07355	Annexin A2	0.46	0.050	0.0E+00	20	55.5
P21810	Biglycan	0.46	0.037	0.0E+00	12	37.8
Q9BQE3	Tubulin alpha-1C chain	0.46	0.061	1.1E-07	11	24.7
Q16555	Dihydropyrimidinase-related protein 2	0.45	0.076	9.5E-04	3	5.6
P01871	Ig mu chain C region	0.44	0.111	6.1E-06	4	13.3
P04083	Annexin A1	0.42	0.145	1.8E-03	8	26.9
P02675	Fibrinogen beta chain	0.38	0.196	2.2E-02	4	12.8
Q15661	Tryptase alpha/beta-1	0.35	0.157	2.5E-04	3	11.6
P41219	Peripherin	0.35	0.181	1.4E-02	3	5.3
P21926	CD9 antigen	0.33	0.127	2.8E-03	3	9.6
P20774	Mimecan	0.32	0.098	3.5E-07	5	14.1
P02671	Fibrinogen alpha chain	0.29	0.140	4.6E-03	4	4.8
P21980	Protein-glutamine gamma-glutamyltransferase 2	0.29	0.083	7.9E-09	10	13.5
P10909	Clusterin	0.28	0.056	0.0E+00	11	24.5
P02649	Apolipoprotein E	0.27	0.099	5.0E-08	9	31.2
P60903	Protein S100-A10	0.27	0.107	2.3E-06	3	35.1
P04004	Vitronectin	0.19	0.081	5.7E-09	7	16.9
P02748	Complement component C9	0.16	0.082	3.1E-06	5	9.3
P02743	Serum amyloid P-component	0.15	0.209	4.2E-04	3	13.9
P25189	Myelin protein P0	0.08	0.117	1.2E-08	4	15.3

Brown denotes change ≥ 2 standard deviations (SD) from the mean, yellow denotes change ≥ 1 SD and green highlights p values ≤ 0.05. NA, not applicable, n<3 unique peptides.
