# Supplementary material for: iTRAQ Quantitative Proteomic Comparison of Metastatic and Non-Metastatic Uveal Melanoma Tumors
Source: PLoS One. 2015 Aug 25;10(8):e0135543. doi: 10.1371/journal.pone.0135543 (PMC4549237; doi:10.1371/journal.pone.0135543)
Supplement: S14 Table — (PDF) [file pone.0135543.s014.pdf]

Supplementary Table S14
Relative Protein Abundance: Sample UM12, Metastatic
Total Proteins Quantified = 193; LogMedian Protein Ratio = 0.07; LogMean Protein Ratio = 0; Standard Deviation = 0.69

Uni-Prot Accession	Protein	Ratio UM/Control	Standard Deviation	*p* value	Unique Peptides	% Sequence Coverage
P02768	Serum albumin	6.84	0.035	0.0E+00	42	66.3
P08670	Vimentin	4.04	0.029	0.0E+00	26	55.2
P00338	L-lactate dehydrogenase A chain	3.99	0.149	1.2E-03	3	10.2
P07195	L-lactate dehydrogenase B chain	3.53	0.069	5.4E-05	3	9.6
P62937	Peptidyl-prolyl cis-trans isomerase A	3.45	0.148	2.7E-03	6	35.2
P06733	Alpha-enolase	3.34	0.101	1.4E-03	6	16.8
P23528	Cofilin-1	3.25	0.132	1.1E-04	3	15.7
P00558	Phosphoglycerate kinase 1	3.08	0.080	1.3E-04	6	15.3
P69905	Hemoglobin subunit alpha	2.75	0.050	2.2E-16	6	58.5
P04406	Glyceraldehyde-3-phosphate dehydrogenase	2.68	0.060	0.0E+00	12	42.1
P68871	Hemoglobin subunit beta	2.42	0.023	0.0E+00	12	84.4
P22392	Nucleoside diphosphate kinase B	2.39	0.070	7.1E-04	3	24.3
Q15746	Myosin light chain kinase, smooth muscle	2.39	0.106	9.7E-05	6	4.3
P02787	Serotransferrin	2.29	0.074	8.0E-10	11	19.9
P01024	Complement C3	2.22	0.298	6.9E-03	5	3.8
P60174	Triosephosphate isomerase	2.18	0.198	4.6E-03	5	21.3
P30086	Phosphatidylethanolamine-binding protein 1	2.13	0.059	6.5E-06	6	36.9
Q99798	Aconitate hydratase, mitochondrial	2.07	0.063	7.6E-03	3	5.3
P04075	Fructose-bisphosphate aldolase A	2.07	0.078	4.1E-03	8	28.3
P06748	Nucleophosmin	2.03	0.103	4.3E-03	4	12.2
P30044	Peroxiredoxin-5, mitochondrial	1.99	0.137	4.6E-03	3	15.4
P14174	Macrophage migration inhibitory factor	8.48	NA	NA	2	17.4
P63104	14-3-3 protein zeta/delta	2.98	NA	NA	2	10.6
P06744	Glucose-6-phosphate isomerase	2.69	NA	NA	2	4.7
O75083	WD repeat-containing protein 1	2.63	NA	NA	2	5.9
P09211	Glutathione S-transferase P	2.53	0.152	3.4E-01	4	25.7
P31946	14-3-3 protein beta/alpha	2.02	NA	NA	2	8.9
P52565	Rho GDP-dissociation inhibitor 1	1.95	0.156	5.6E-03	3	15.2
P14618	Pyruvate kinase PKM	1.94	0.117	1.5E-01	10	21.3
P30041	Peroxiredoxin-6	1.93	0.075	1.1E-02	5	16.5
P40926	Malate dehydrogenase, mitochondrial	1.90	0.059	1.2E-06	8	29.0
P07339	Cathepsin D	1.90	0.052	6.1E-03	3	7.3
P02652	Apolipoprotein A-II	1.85	NA	NA	2	11.0
P08758	Annexin A5	1.81	0.074	1.3E-07	10	33.4
P02647	Apolipoprotein A-I	1.79	0.066	6.6E-07	6	24.0
P05141	ADP/ATP translocase 2	1.78	0.079	1.6E-02	6	17.8
P62158	Calmodulin	1.77	NA	NA	2	22.1
P29401	Transketolase	1.76	0.104	2.0E-02	3	5.5
Q01995	Transgelin	1.75	0.026	3.3E-06	3	15.9
P35749	Myosin-11	1.74	0.042	0.0E+00	36	20.0
P09493	Tropomyosin alpha-1 chain	1.73	0.042	4.9E-09	7	19.7
Q9H0U4	Ras-related protein Rab-1B	1.70	NA	NA	2	11.4
P07900	Heat shock protein HSP 90-alpha	1.67	NA	NA	2	2.2
P0CG05	Ig lambda-2 chain C regions	1.67	NA	NA	2	23.6
P45880	Voltage-dependent anion-selective channel protein 2	1.65	0.105	5.6E-02	3	12.9
P21796	Voltage-dependent anion-selective channel protein 1	1.61	0.115	1.1E-02	5	22.3
P11142	Heat shock cognate 71 kDa protein	1.61	0.077	1.6E-02	12	20.9
Q12905	Interleukin enhancer-binding factor 2	1.57	NA	NA	2	6.7
P01009	Alpha-1-antitrypsin	1.57	0.058	9.9E-07	6	17.7
P31040	Succinate dehydrogenase [ubiquinone] flavoprotein subunit, mitochondrial	1.52	NA	NA	2	4.2
P25705	ATP synthase subunit alpha, mitochondrial	1.49	0.072	1.8E-05	5	11.8
Q5VTE0	Putative elongation factor 1-alpha-like 3	1.49	0.073	4.0E-05	7	14.1
P16615	Sarcoplasmic/endoplasmic reticulum calcium ATPase 2	1.49	NA	NA	2	2.6
P10412	Histone H1.4	1.48	0.035	6.2E-09	4	15.1
P06576	ATP synthase subunit beta, mitochondrial	1.48	0.066	4.0E-07	11	23.1
P09651	Heterogeneous nuclear ribonucleoprotein A1	1.47	0.040	1.1E-07	5	13.7
P24844	Myosin regulatory light polypeptide 9	1.43	NA	NA	2	12.2
Q15063	Periostin	1.43	NA	NA	2	4.7
Q02878	60S ribosomal protein L6	1.42	NA	NA	2	8.0
P05787	Keratin, type II cytoskeletal 8	1.41	NA	NA	2	4.6
P30101	Protein disulfide-isomerase A3	1.39	0.027	1.5E-04	6	12.9
P06703	Protein S100-A6	1.39	NA	NA	2	16.7
P38646	Stress-70 protein, mitochondrial	1.38	0.100	1.8E-02	5	9.1
P22626	Heterogeneous nuclear ribonucleoproteins A2/B1	1.38	0.036	3.0E-09	6	17.8
P49411	Elongation factor Tu, mitochondrial	1.36	0.084	9.4E-03	5	14.2
P09429	High mobility group protein B1	1.36	0.069	1.0E-04	3	18.1
P35232	Prohibitin	1.35	0.025	3.2E-03	3	12.9
P01857	Ig gamma-1 chain C region	1.32	0.062	3.1E-05	4	18.8
P50895	Basal cell adhesion molecule	1.31	NA	NA	2	4.3
O15230	Laminin subunit alpha-5	1.30	0.163	2.6E-02	6	2.4
Q99623	Prohibitin-2	1.29	NA	NA	2	11.0
Q14697	Neutral alpha-glucosidase AB	1.29	NA	NA	2	3.2
P61978	Heterogeneous nuclear ribonucleoprotein K	1.28	NA	NA	2	5.2
O75964	ATP synthase subunit g, mitochondrial	1.27	NA	NA	2	27.2
P39019	40S ribosomal protein S19	1.23	0.247	3.2E-01	3	18.6
P24539	ATP synthase F(0) complex subunit B1, mitochondrial	1.21	NA	NA	2	10.5
Q06830	Peroxiredoxin-1	1.21	0.164	8.3E-03	9	51.8
P12814	Alpha-actinin-1	1.20	0.084	1.3E-02	8	11.3
P26641	Elongation factor 1-gamma	1.19	0.050	2.4E-02	3	8.9
P61604	10 kDa heat shock protein, mitochondrial	1.18	NA	NA	2	19.6
P11047	Laminin subunit gamma-1	1.18	0.070	4.7E-02	3	2.4
P62701	40S ribosomal protein S4, X isoform	1.17	NA	NA	2	7.2
P23284	Peptidyl-prolyl cis-trans isomerase B	1.17	0.075	9.5E-02	5	22.7
P55268	Laminin subunit beta-2	1.17	0.098	4.5E-02	9	5.3
P18206	Vinculin	1.15	NA	NA	2	2.0
P26447	Protein S100-A4	1.13	NA	NA	2	18.8
P08107	Heat shock 70 kDa protein 1A/1B	1.12	0.316	2.1E-01	7	10.1
O00264	Membrane-associated progesterone receptor component 1	1.11	0.092	1.4E-01	3	15.4
P17931	Galectin-3	1.11	0.156	8.4E-02	5	24.0
O14773	Tripeptidyl-peptidase 1	1.10	0.069	2.3E-02	3	7.5
P07237	Protein disulfide-isomerase	1.10	0.143	4.8E-01	4	7.9
P12956	X-ray repair cross-complementing protein 6	1.09	NA	NA	2	3.0
P10809	60 kDa heat shock protein, mitochondrial	1.09	NA	NA	2	3.3
P11166	Solute carrier family 2, facilitated glucose transporter member 1	1.09	0.020	5.9E-02	3	7.1
P27797	Calreticulin	1.08	0.119	3.4E-01	3	7.2
P04843	Dolichyl-diphosphooligosaccharide--protein glycosyltransferase subunit 1	1.08	NA	NA	2	4.1

P22695	Cytochrome b-c1 complex subunit 2, mitochondrial	1.07	0.143	3.0E-01	3	10.4
P62906	60S ribosomal protein L10a	1.07	NA	NA	2	12.0
P06396	Gelsolin	1.04	2.839	6.9E-01	6	10.9
P26038	Moesin	1.04	0.321	7.4E-01	6	9.5
P67936	Tropomyosin alpha-4 chain	1.04	0.266	5.8E-01	4	17.3
P0CG47	Polyubiquitin-B	1.03	0.132	3.5E-01	7	32.3
Q15084	Protein disulfide-isomerase A6	1.03	NA	NA	2	6.8
Q15149	Plectin	1.02	0.043	4.4E-01	5	1.2
Q16891	Mitochondrial inner membrane protein	0.97	NA	NA	2	4.4
P14625	Endoplasmin	0.97	0.402	8.9E-01	6	8.8
P60660	Myosin light polypeptide 6	0.97	0.223	5.2E-01	7	58.3
P27824	Calnexin	0.96	0.779	5.5E-01	5	11.0
P62736	Actin, aortic smooth muscle	0.96	0.108	6.2E-01	8	33.4
Q99536	Synaptic vesicle membrane protein VAT-1 homolog	0.95	0.087	2.5E-01	3	7.9
P32119	Peroxiredoxin-2	0.94	0.287	7.7E-01	4	22.2
P11021	78 kDa glucose-regulated protein	0.94	0.350	7.3E-01	6	11.3
P40939	Trifunctional enzyme subunit alpha, mitochondrial	0.93	0.121	4.2E-01	3	5.6
P02751	Fibronectin	0.92	NA	NA	2	1.1
P14543	Nidogen-1	0.90	0.185	3.9E-01	3	3.4
P17858	ATP-dependent 6-phosphofructokinase, liver type	0.88	NA	NA	2	4.5
O43707	Alpha-actinin-4	0.86	0.152	1.5E-01	5	9.2
P55072	Transitional endoplasmic reticulum ATPase	0.84	0.330	5.3E-01	3	5.0
P08133	Annexin A6	0.83	0.096	4.2E-03	13	23.0
P01834	Ig kappa chain C region	0.83	NA	NA	2	33.0
P37802	Transgelin-2	0.83	NA	NA	2	11.1
Q00610	Clathrin heavy chain 1	0.83	0.156	1.1E-01	7	5.1
P63096	Guanine nucleotide-binding protein G(i) subunit alpha-1	0.83	NA	NA	2	5.9
P05023	Sodium/potassium-transporting ATPase subunit alpha-1	0.83	NA	NA	2	3.2
O75367	Core histone macro-H2A.1	0.81	0.090	2.7E-02	4	13.7
P98160	Basement membrane-specific heparan sulfate proteoglycan core protein	0.81	0.050	2.2E-04	12	3.3
Q9NQC3	Reticulon-4	0.81	NA	NA	2	2.3
P07099	Epoxide hydrolase 1	0.81	0.092	1.1E-01	4	13.4
P08294	Extracellular superoxide dismutase [Cu-Zn]	0.81	NA	NA	2	12.1
P01903	HLA class II histocompatibility antigen, DR alpha chain	0.80	NA	NA	2	11.4
P58876	Histone H2B type 1-D	0.78	NA	NA	2	7.9
P60709	Actin, cytoplasmic 1	0.77	0.046	1.2E-04	7	27.7
P09382	Galectin-1	0.75	0.060	2.5E-04	5	43.7
P21333	Filamin-A	0.74	0.057	1.9E-05	22	11.4
P08572	Collagen alpha-2(IV) chain	0.74	0.171	6.0E-02	4	2.9
Q9UHG3	Prenylcysteine oxidase 1	0.73	NA	NA	2	4.4
Q09666	Neuroblast differentiation-associated protein AHNAK	0.73	0.080	1.9E-03	12	1.4
P04792	Heat shock protein beta-1	0.68	0.076	4.0E-04	6	36.1
Q01082	Spectrin beta chain, non-erythrocytic 1	0.68	0.055	4.4E-06	12	7.7
P02545	Prelamin-A/C	0.67	0.023	5.3E-15	19	29.1
P02511	Alpha-crystallin B chain	0.66	NA	NA	2	12.6
Q71DI3	Histone H3.2	0.65	0.026	2.4E-08	4	21.3
P00450	Ceruloplasmin	0.64	0.140	6.2E-02	3	4.4
Q9Y490	Talin-1	0.64	NA	NA	2	0.7
Q29718	HLA class I histocompatibility antigen, B-82 alpha chain	0.64	NA	NA	2	8.3
P00738	Haptoglobin	0.63	0.024	1.3E-02	3	6.2
P35579	Myosin-9	0.62	0.061	9.1E-09	22	13.0
P06899	Histone H2B type 1-J	0.62	NA	NA	2	7.9
P12111	Collagen alpha-3(VI) chain	0.62	0.171	4.6E-02	3	1.3
P39060	Collagen alpha-1(XVIII) chain	0.61	0.200	5.8E-01	3	2.5
P13987	CD59 glycoprotein	0.60	NA	NA	2	15.6
P12109	Collagen alpha-1(VI) chain	0.58	NA	NA	2	2.6
Q9BQE3	Tubulin alpha-1C chain	0.58	0.039	2.9E-08	11	29.4
P62805	Histone H4	0.58	0.029	1.2E-10	6	51.5
P41219	Peripherin	0.57	0.023	2.9E-06	4	10.9
P07437	Tubulin beta chain	0.55	0.049	6.4E-06	3	10.6
Q14195	Dihydropyrimidinase-related protein 3	0.54	0.091	2.7E-03	3	7.7
Q13813	Spectrin alpha chain, non-erythrocytic 1	0.54	0.038	0.0E+00	27	15.1
P0C0S5	Histone H2A.Z	0.54	NA	NA	2	18.8
P68371	Tubulin beta-4B chain	0.54	0.067	2.1E-04	3	10.6
Q16555	Dihydropyrimidinase-related protein 2	0.54	0.086	4.3E-03	3	9.1
P01011	Alpha-1-antichymotrypsin	0.54	0.116	7.1E-03	4	9.0
P80723	Brain acid soluble protein 1	0.52	0.262	1.5E-01	4	31.7
Q03252	Lamin-B2	0.51	NA	NA	2	3.0
O14950	Myosin regulatory light chain 12B	0.50	NA	NA	2	12.2
Q15019	Septin-2	0.50	NA	NA	2	6.4
Q14956	Transmembrane glycoprotein NMB	0.48	NA	NA	2	4.7
P02675	Fibrinogen beta chain	0.46	NA	NA	2	6.7
P07585	Decorin	0.35	0.198	2.5E-01	3	6.4
P21926	CD9 antigen	0.32	NA	NA	2	7.5
P08123	Collagen alpha-2(I) chain	0.32	NA	NA	2	2.4
P60903	Protein S100-A10	0.28	NA	NA	2	35.1
P23946	Chymase	0.21	NA	NA	2	11.7
P02743	Serum amyloid P-component	0.13	NA	NA	2	11.2
P07355	Annexin A2	0.48	0.043	1.2E-14	19	53.7
P01871	Ig mu chain C region	0.46	0.074	2.9E-09	4	13.1
P35555	Fibrillin-1	0.43	0.023	0.00E+00	18	6.9
P51888	Prolargin	0.43	0.040	0.00E+00	13	37.7
P02749	Beta-2-glycoprotein 1	0.38	0.033	3.8E-05	3	9.3
P21810	Biglycan	0.37	0.055	1.5E-10	10	32.9
P21980	Protein-glutamine gamma-glutamyltransferase 2	0.35	0.097	4.4E-06	8	13.1
P51884	Lumican	0.34	0.048	1.0E-10	11	40.5
P04083	Annexin A1	0.34	0.087	1.1E-04	9	32.7
P02671	Fibrinogen alpha chain	0.33	0.220	2.4E-03	5	7.3
P20774	Mimecan	0.31	0.109	4.6E-06	4	11.4
P10909	Clusterin	0.31	0.068	2.2E-12	12	27.8
Q15661	Tryptase alpha/beta-1	0.31	0.121	1.2E-06	4	17.1
P25189	Myelin protein P0	0.30	0.097	4.8E-10	5	20.6
P04004	Vitronectin	0.29	0.097	8.1E-07	8	20.7
P02730	Band 3 anion transport protein	0.24	0.103	1.4E-06	5	8.7
P02649	Apolipoprotein E	0.24	0.132	2.3E-05	7	23.3
P02748	Complement component C9	0.22	0.103	1.7E-06	4	7.9
P35625	Metalloproteinase inhibitor 3	0.21	0.200	8.0E-03	3	15.6

Brown denotes change ≥ 2 standard deviations (SD) from the mean, yellow denotes change ≥ 1 SD and green highlights p values ≤ 0.05. NA, not applicable, n<3 unique peptides.
