# Supplementary material for: iTRAQ Quantitative Proteomic Comparison of Metastatic and Non-Metastatic Uveal Melanoma Tumors
Source: PLoS One. 2015 Aug 25;10(8):e0135543. doi: 10.1371/journal.pone.0135543 (PMC4549237; doi:10.1371/journal.pone.0135543)
Supplement: S15 Table — (PDF) [file pone.0135543.s015.pdf]

Supplementary Table S15
Relative Protein Abundance: Sample UM15, Non-Metastatic
Total Proteins Quantified = 965; LogMedian Protein Ratio = 0.15; LogMean Protein Ratio = 0; Standard Deviation = 0.89

Uni-Prot Accession	Protein	Ratio UM/Control	Standard Deviation	*p* value	Unique Peptides	% Sequence Coverage
P09211	Glutathione S-transferase P	8.91	0.109	4.1E-09	6	30.5
P08473	Neprilysin	8.12	0.306	4.1E-03	4	5.5
P55769	NHP2-like protein 1	8.00	0.025	9.0E-07	3	24.2
P00338	L-lactate dehydrogenase A chain	6.58	0.094	3.2E-10	9	22.9
P63241	Eukaryotic translation initiation factor 5A-1	6.45	0.175	1.0E-03	7	33.1
P29401	Transketolase	6.23	0.161	2.7E-08	14	23.1
P62937	Peptidyl-prolyl cis-trans isomerase A	5.68	0.108	1.1E-15	7	38.2
P31948	Stress-induced-phosphoprotein 1	5.36	0.102	1.8E-13	19	25.8
P06748	Nucleophosmin	5.02	0.120	2.3E-09	7	19.4
P00558	Phosphoglycerate kinase 1	4.90	0.080	1.3E-15	13	27.3
P78417	Glutathione S-transferase omega-1	4.87	0.134	1.5E-07	7	24.5
P07195	L-lactate dehydrogenase B chain	4.78	0.100	5.9E-08	11	31.1
P39748	Flap endonuclease 1	4.75	0.251	9.8E-04	3	7.4
Q04760	Lactoylglutathione lyase	4.73	0.123	1.4E-05	4	18.5
P04080	Cystatin-B	4.66	0.139	5.7E-07	4	55.1
Q9Y2X3	Nucleolar protein 58	4.65	0.431	1.9E-02	6	14.7
P19338	Nucleolin	4.60	0.076	0.0E+00	17	22.1
Q12931	Heat shock protein 75 kDa, mitochondrial	4.50	0.331	4.3E-03	3	6.1
Q9Y2S2	Lambda-crystallin homolog	4.41	0.194	4.8E-04	5	16.0
Q9UQ80	Proliferation-associated protein 2G4	4.41	0.174	2.4E-03	3	9.9
P22087	rRNA 2'-O-methyltransferase fibrillarin	4.37	0.161	1.6E-05	7	27.4
P30086	Phosphatidylethanolamine-binding protein 1	4.33	0.094	7.9E-06	8	48.7
P23528	Cofilin-1	4.22	0.125	9.1E-07	9	42.8
P02768	Serum albumin	4.14	0.030	0.0E+00	39	53.4
P60174	Triosephosphate isomerase	4.13	0.117	1.1E-06	13	49.7
P40925	Malate dehydrogenase, cytoplasmic	4.12	0.121	9.9E-07	6	22.2
P15531	Nucleoside diphosphate kinase A	3.98	0.078	6.8E-09	7	48.0
Q92820	Gamma-glutamyl hydrolase	3.93	0.104	5.3E-06	5	16.4
P15121	Aldose reductase	3.93	0.283	2.5E-03	5	20.3
P06733	Alpha-enolase	3.82	0.073	6.5E-13	13	36.6
Q13838	Spliceosome RNA helicase DDX39B	3.67	0.151	3.0E-02	5	12.4
O75347	Tubulin-specific chaperone A	3.61	0.184	4.6E-03	4	33.3
P63104	14-3-3 protein zeta/delta	3.59	0.107	6.6E-06	5	26.5
P04406	Glyceraldehyde-3-phosphate dehydrogenase	3.57	0.061	2.2E-16	11	38.2
P07108	Acyl-CoA-binding protein	3.55	0.149	5.4E-03	3	52.9
P20591	Interferon-induced GTP-binding protein Mx1	3.50	0.285	3.4E-03	9	15.0
P07900	Heat shock protein HSP 90-alpha	3.45	0.078	8.7E-13	15	16.0
P16152	Carbonyl reductase [NADPH] 1	3.43	0.181	1.8E-03	5	23.1
Q13185	Chromobox protein homolog 3	3.36	0.143	2.1E-05	4	20.8
P57729	Ras-related protein Rab-38	3.29	0.183	2.8E-03	4	17.5
P23526	Adenosylhomocysteinase	3.28	0.157	3.1E-04	7	18.8
P24941	Cyclin-dependent kinase 2	3.23	0.028	1.4E-03	3	14.8
P08670	Vimentin	3.23	0.033	0.0E+00	32	61.6
P50453	Serpin B9	3.21	0.159	2.3E-04	8	25.0
P61088	Ubiquitin-conjugating enzyme E2 N	3.21	0.259	2.0E-02	3	20.4
P13639	Elongation factor 2	3.20	0.107	5.1E-09	16	18.4
P02787	Serotransferrin	3.19	0.069	8.0E-15	20	30.9
O00567	Nucleolar protein 56	3.12	0.102	8.3E-06	7	11.8
P16401	Histone H1.5	3.12	0.092	7.7E-03	4	14.6
O75368	SH3 domain-binding glutamic acid-rich-like protein	3.00	0.093	1.6E-05	3	32.5
P62826	GTP-binding nuclear protein Ran	2.99	0.180	3.4E-03	3	18.5
P13798	Acylamino-acid-releasing enzyme	2.93	0.170	5.6E-03	4	5.7
Q13576	Ras GTPase-activating-like protein IQGAP2	2.90	0.107	1.7E-02	3	1.8
P17931	Galectin-3	2.87	0.052	4.3E-06	6	28.4
P14618	Pyruvate kinase PKM	2.86	0.061	1.5E-12	13	27.3
Q12906	Interleukin enhancer-binding factor 3	2.85	0.095	1.2E-06	12	16.1
P12955	Xaa-Pro dipeptidase	2.77	0.039	4.1E-02	3	5.5
P60842	Eukaryotic initiation factor 4A-I	2.75	0.223	2.8E-03	4	12.1
Q92598	Heat shock protein 105 kDa	2.73	0.117	7.1E-04	8	9.8
P08238	Heat shock protein HSP 90-beta	2.72	0.101	3.8E-08	14	17.4
Q8IV08	Phospholipase D3	2.70	0.195	3.7E-03	3	6.3
Q08380	Galectin-3-binding protein	2.67	0.168	1.2E-03	4	6.5
P52565	Rho GDP-dissociation inhibitor 1	2.64	0.175	1.0E-02	4	22.5
P06744	Glucose-6-phosphate isomerase	2.63	0.119	1.2E-05	8	14.7
Q14247	Src substrate cortactin	2.62	0.168	8.2E-04	7	16.5
P05387	60S acidic ribosomal protein P2	2.60	0.190	9.7E-03	5	25.2
P07910	Heterogeneous nuclear ribonucleoproteins C1/C2	2.58	0.098	1.3E-05	8	28.8
Q07955	Serine/arginine-rich splicing factor 1	2.52	0.134	3.9E-04	5	17.3
P50395	Rab GDP dissociation inhibitor beta	2.51	0.170	1.3E-03	8	20.7
Q96AG4	Leucine-rich repeat-containing protein 59	2.49	0.198	2.6E-03	5	16.9
Q13423	NAD(P) transhydrogenase, mitochondrial	2.48	0.092	1.6E-06	21	19.3
P07737	Profilin-1	2.48	0.118	1.9E-05	5	32.9
P09429	High mobility group protein B1	2.47	0.155	7.1E-03	9	36.7
Q15233	Non-POU domain-containing octamer-binding protein	2.44	0.201	2.2E-03	4	9.3
P23381	Tryptophan--tRNA ligase, cytoplasmic	7.92	NA	NA	2	4.5
P40967	Melanocyte protein PMEL	6.08	NA	NA	2	3.3
Q99541	Perilipin-2	6.02	NA	NA	2	8.0
Q9UBE0	SUMO-activating enzyme subunit 1	5.88	NA	NA	2	5.5
P20042	Eukaryotic translation initiation factor 2 subunit 2	5.67	NA	NA	2	9.9
Q96IU4	Alpha/beta hydrolase domain-containing protein 14B	4.97	NA	NA	2	11.0
Q13247	Serine/arginine-rich splicing factor 6	4.83	NA	NA	2	5.5
P48739	Phosphatidylinositol transfer protein beta isoform	4.13	NA	NA	2	7.0
Q00796	Sorbitol dehydrogenase	3.95	NA	NA	2	4.8
P13693	Translationally-controlled tumor protein	3.70	NA	NA	2	15.7
P10768	S-formylglutathione hydrolase	3.65	NA	NA	2	6.0
P58546	Myotrophin	3.62	NA	NA	2	25.4
P05413	Fatty acid-binding protein, heart	3.59	NA	NA	2	11.3
Q86WA6	Valacyclovir hydrolase	3.54	NA	NA	2	8.6
Q03154	Aminoacylase-1	3.43	0.369	3.6E-01	3	7.4
P05067	Amyloid beta A4 protein	3.40	NA	NA	2	2.3
Q9Y2W1	Thyroid hormone receptor-associated protein 3	3.20	0.294	8.8E-02	3	4.3
Q99497	Protein DJ-1	3.15	NA	NA	2	16.4
Q16658	Fascin	3.08	NA	NA	2	5.3
P17096	High mobility group protein HMG-I/HMG-Y	3.04	NA	NA	2	23.4
P60900	Proteasome subunit alpha type-6	2.96	NA	NA	2	8.5
Q6BCY4	NADH-cytochrome b5 reductase 2	2.92	NA	NA	2	11.2
P30085	UMP-CMP kinase	2.88	NA	NA	2	10.7
Q9BVP2	Guanine nucleotide-binding protein-like 3	2.84	0.729	2.1E-01	3	4.4
Q9BRA2	Thioredoxin domain-containing protein 17	2.83	NA	NA	2	18.7
P12081	Histidine--tRNA ligase, cytoplasmic	2.82	NA	NA	2	3.9
P49773	Histidine triad nucleotide-binding protein 1	2.79	0.572	1.2E-01	3	37.3
P54819	Adenylate kinase 2, mitochondrial	2.77	NA	NA	2	10.9
Q00688	Peptidyl-prolyl cis-trans isomerase FKBP3	2.73	NA	NA	2	12.9
Q9BUP0	EF-hand domain-containing protein D1	2.72	0.227	1.2E-01	3	10.9
P21283	V-type proton ATPase subunit C 1	2.71	NA	NA	2	3.9
P30040	Endoplasmic reticulum resident protein 29	2.62	NA	NA	2	8.4
P00441	Superoxide dismutase [Cu-Zn]	2.61	NA	NA	2	13.0
Q14978	Nucleolar and coiled-body phosphoprotein 1	2.60	NA	NA	2	3.9

P13929	Beta-enolase	2.59	NA	NA	2	6.7
P62495	Eukaryotic peptide chain release factor subunit 1	2.55	0.202	5.8E-02	3	6.6
O00541	Pescadillo homolog	2.55	NA	NA	2	2.7
P10599	Thioredoxin	2.53	NA	NA	2	20.0
P25788	Proteasome subunit alpha type-3	2.50	NA	NA	2	4.7
P36915	Guanine nucleotide-binding protein-like 1	2.50	NA	NA	2	4.3
Q16629	Serine/arginine-rich splicing factor 7	2.49	NA	NA	2	8.8
Q9BV40	Vesicle-associated membrane protein 8	2.49	NA	NA	2	24.0
Q9UBR2	Cathepsin Z	2.47	NA	NA	2	7.3
P49006	MARCKS-related protein	2.47	0.295	5.9E-02	3	17.9
O15371	Eukaryotic translation initiation factor 3 subunit D	2.45	NA	NA	2	3.5
Q01105	Protein SET	2.43	0.098	1.1E-04	6	21.4
Q92688	Acidic leucine-rich nuclear phosphoprotein 32 family member B	2.42	NA	NA	2	9.6
P27816	Microtubule-associated protein 4	2.42	0.217	3.2E-04	7	9.0
Q12905	Interleukin enhancer-binding factor 2	2.42	0.087	2.0E-05	3	9.5
P62258	14-3-3 protein epsilon	2.40	0.074	3.3E-07	9	31.4
P26640	Valine--tRNA ligase	2.40	0.158	3.4E-03	4	4.4
P18754	Regulator of chromosome condensation	2.39	NA	NA	2	8.6
Q9UHX1	Poly(U)-binding-splicing factor PUF60	2.36	0.142	1.6E-02	3	5.0
O76021	Ribosomal L1 domain-containing protein 1	2.33	0.283	9.1E-02	4	7.1
P18669	Phosphoglycerate mutase 1	2.32	0.102	1.5E-04	4	26.8
P31949	Protein S100-A11	2.32	NA	NA	2	23.8
P50454	Serpin H1	2.32	0.087	1.9E-06	9	23.4
O00625	Pirin	2.31	NA	NA	2	6.2
P67809	Nuclease-sensitive element-binding protein 1	2.30	NA	NA	2	11.7
P53999	Activated RNA polymerase II transcriptional coactivator p15	2.28	NA	NA	2	15.7
P25786	Proteasome subunit alpha type-1	2.27	0.135	5.4E-04	4	15.6
P42224	Signal transducer and activator of transcription 1-alpha/beta	2.26	0.138	4.7E-03	6	9.1
P31939	Bifunctional purine biosynthesis protein PURH	2.25	0.271	1.4E-01	4	7.4
P06454	Prothymosin alpha	2.23	NA	NA	2	20.7
P20618	Proteasome subunit beta type-1	2.22	0.090	5.3E-04	3	13.3
P05198	Eukaryotic translation initiation factor 2 subunit 1	2.21	NA	NA	2	7.0
Q08211	ATP-dependent RNA helicase A	2.20	0.142	4.0E-05	8	6.8
P11216	Glycogen phosphorylase, brain form	2.20	0.085	4.3E-08	12	16.4
P30044	Peroxiredoxin-5, mitochondrial	2.20	NA	NA	2	11.7
P30043	Flavin reductase (NADPH)	2.19	0.104	7.8E-03	3	18.9
P42766	60S ribosomal protein L35	2.19	NA	NA	2	13.8
Q13435	Splicing factor 3B subunit 2	2.19	0.155	8.1E-03	5	7.2
P49207	60S ribosomal protein L34	2.18	0.144	5.5E-02	3	20.5
P62906	60S ribosomal protein L10a	2.15	0.081	1.6E-05	8	35.9
P27348	14-3-3 protein theta	2.15	0.076	1.1E-05	5	21.6
P02765	Alpha-2-HS-glycoprotein	2.13	0.225	6.5E-02	4	9.3
P26641	Elongation factor 1-gamma	2.12	0.159	5.8E-03	5	11.9
Q8NC51	Plasminogen activator inhibitor 1 RNA-binding protein	2.12	0.420	2.5E-01	3	10.8
Q92945	Far upstream element-binding protein 2	2.11	0.136	1.9E-04	10	14.2
P63244	Guanine nucleotide-binding protein subunit beta-2-like 1	2.11	0.218	1.6E-02	3	11.0
P08758	Annexin A5	2.10	0.061	3.7E-10	16	38.8
Q9HAU0	Pleckstrin homology domain-containing family A member 5	2.10	NA	NA	2	3.3
P40926	Malate dehydrogenase, mitochondrial	2.08	0.129	2.2E-04	7	22.5
Q13442	28 kDa heat- and acid-stable phosphoprotein	2.07	NA	NA	2	13.8
Q02790	Peptidyl-prolyl cis-trans isomerase FKBP4	2.06	0.235	1.7E-02	6	21.4
P62750	60S ribosomal protein L23a	2.06	0.062	2.5E-05	5	27.6
Q9H0A0	N-acetyltransferase 10	2.05	NA	NA	2	2.2
Q13510	Acid ceramidase	2.05	0.182	9.5E-03	4	7.6
O75533	Splicing factor 3B subunit 1	2.04	0.192	9.6E-02	4	3.6
P21796	Voltage-dependent anion-selective channel protein 1	2.03	0.075	4.8E-07	8	35.0
Q01130	Serine/arginine-rich splicing factor 2	2.03	NA	NA	2	10.9
P99999	Cytochrome c	2.02	NA	NA	2	17.1
P07741	Adenine phosphoribosyltransferase	2.00	0.491	3.0E-01	3	20.0
P61247	40S ribosomal protein S3a	2.00	0.090	1.4E-05	7	28.4
P46777	60S ribosomal protein L5	2.00	0.089	5.5E-04	5	17.8
P48426	Phosphatidylinositol 5-phosphate 4-kinase type-2 alpha	1.99	NA	NA	2	6.2
P62136	Serine/threonine-protein phosphatase PP1-alpha catalytic subunit	1.99	0.177	3.2E-02	3	9.1
Q8WXF1	Paraspeckle component 1	1.99	NA	NA	2	4.0
O14818	Proteasome subunit alpha type-7	1.99	0.110	1.3E-03	5	20.6
P23246	Splicing factor, proline- and glutamine-rich	1.99	0.259	8.8E-03	8	11.9
Q9NY12	H/ACA ribonucleoprotein complex subunit 1	1.99	NA	NA	2	7.4
Q01628	Interferon-induced transmembrane protein 3	1.98	NA	NA	2	12.8
P10619	Lysosomal protective protein	1.98	0.176	1.8E-02	3	6.9
P41091	Eukaryotic translation initiation factor 2 subunit 3	1.97	NA	NA	2	5.5
Q15631	Translin	1.97	0.517	2.5E-01	3	12.7
P05388	60S acidic ribosomal protein P0	1.97	0.171	3.6E-02	4	10.7
P07858	Cathepsin B	1.97	0.191	5.3E-03	5	15.9
P61916	Epididymal secretory protein E1	1.96	NA	NA	2	12.6
P07954	Fumarate hydratase, mitochondrial	1.96	0.231	5.3E-02	3	6.9
O14556	Glyceraldehyde-3-phosphate dehydrogenase, testis-specific	1.96	0.149	8.6E-02	3	8.8
P48681	Nestin	1.95	0.299	4.2E-02	3	3.0
Q16576	Histone-binding protein RBBP7	1.95	NA	NA	2	9.2
P05141	ADP/ATP translocase 2	1.94	0.106	4.7E-03	5	16.8
Q99436	Proteasome subunit beta type-7	1.94	NA	NA	2	7.2
P13010	X-ray repair cross-complementing protein 5	1.94	0.081	8.5E-06	13	16.0
P46783	40S ribosomal protein S10	1.94	0.293	5.8E-02	3	20.0
Q9BZZ5	Apoptosis inhibitor 5	1.93	0.064	2.2E-03	3	6.5
P27695	DNA-(apurinic or apyrimidinic site) lyase	1.93	0.973	5.5E-01	4	12.9
Q15181	Inorganic pyrophosphatase	1.92	1.006	2.4E-01	3	13.1
P10809	60 kDa heat shock protein, mitochondrial	1.92	0.109	3.6E-05	14	25.5
P16070	CD44 antigen	1.92	0.146	1.0E-01	8	10.0
P09651	Heterogeneous nuclear ribonucleoprotein A1	1.92	0.117	5.5E-05	7	25.5
Q9Y5B9	FACT complex subunit SPT16	1.92	0.437	2.7E-01	3	3.4
P62263	40S ribosomal protein S14	1.91	0.087	6.0E-04	4	36.4
E9PAV3	Nascent polypeptide-associated complex subunit alpha, muscle-specific form	1.90	0.134	1.1E-02	3	2.0
P51159	Ras-related protein Rab-27A	1.90	NA	NA	2	10.9
P61204	ADP-ribosylation factor 3	1.90	NA	NA	2	10.5
P61254	60S ribosomal protein L26	1.89	0.082	5.2E-03	4	26.2
O75083	WD repeat-containing protein 1	1.89	0.126	7.1E-04	5	8.3
Q07020	60S ribosomal protein L18	1.89	0.171	4.2E-02	3	18.6
Q14974	Importin subunit beta-1	1.88	0.070	1.9E-02	4	6.7
P25398	40S ribosomal protein S12	1.88	0.081	3.4E-03	3	22.0
P23284	Peptidyl-prolyl cis-trans isomerase B	1.88	0.054	1.3E-04	13	51.4
Q9BUJ2	Heterogeneous nuclear ribonucleoprotein U-like protein 1	1.88	NA	NA	2	3.4
Q92616	Translational activator GCN1	1.88	0.080	8.0E-02	3	1.2
Q9BR76	Coronin-1B	1.88	0.102	3.8E-03	4	6.7
Q9Y608	Leucine-rich repeat flightless-interacting protein 2	1.88	NA	NA	2	4.9
P51858	Hepatoma-derived growth factor	1.87	0.154	2.4E-02	3	14.6
O75436	Vacuolar protein sorting-associated protein 26A	1.87	0.301	1.2E-01	3	11.0
P26373	60S ribosomal protein L13	1.87	0.044	3.7E-08	4	19.4
P43243	Matrin-3	1.87	0.164	1.4E-02	5	7.6
P62829	60S ribosomal protein L23	1.87	NA	NA	2	12.9
P49792	E3 SUMO-protein ligase RanBP2	1.87	0.212	4.9E-02	4	0.8
Q9UJS0	Calcium-binding mitochondrial carrier protein Aralar2	1.86	0.214	4.2E-02	4	6.8
P51991	Heterogeneous nuclear ribonucleoprotein A3	1.86	0.054	5.2E-05	8	24.9
P42704	Leucine-rich PPR motif-containing protein, mitochondrial	1.85	0.089	2.4E-03	5	3.8
P29692	Elongation factor 1-delta	1.85	0.106	2.2E-01	3	15.7
P34932	Heat shock 70 kDa protein 4	1.85	0.123	1.0E-03	8	11.1
P13796	Plastin-2	1.85	NA	NA	2	3.3

P39687	Acidic leucine-rich nuclear phosphoprotein 32 family member A	1.84	0.249	6.6E-02	3	14.1
Q07666	KH domain-containing, RNA-binding, signal transduction-associated protein 1	1.84	0.092	6.6E-04	6	11.5
P17900	Ganglioside GM2 activator	1.84	NA	NA	2	7.8
P61604	10 kDa heat shock protein, mitochondrial	1.84	0.115	2.1E-03	5	49.0
P61353	60S ribosomal protein L27	1.84	0.104	3.2E-04	3	27.9
Q13303	Voltage-gated potassium channel subunit beta-2	1.83	NA	NA	2	4.9
P12956	X-ray repair cross-complementing protein 6	1.82	0.104	7.7E-04	9	16.6
Q02878	60S ribosomal protein L6	1.82	0.069	8.7E-06	9	33.0
P18621	60S ribosomal protein L17	1.82	0.040	4.9E-02	3	19.0
O14879	Interferon-induced protein with tetratricopeptide repeats 3	1.82	NA	NA	2	4.5
P28070	Proteasome subunit beta type-4	1.81	NA	NA	2	7.2
Q8TCS8	Polyribonucleotide nucleotidyltransferase 1, mitochondrial	1.80	NA	NA	2	2.8
P50914	60S ribosomal protein L14	1.80	0.098	1.4E-04	3	16.3
P24534	Elongation factor 1-beta	1.78	0.147	2.0E-02	3	9.8
Q9GZT3	SRA stem-loop-interacting RNA-binding protein, mitochondrial	1.77	NA	NA	2	23.9
P02790	Hemopexin	1.77	0.053	1.6E-05	3	6.3
P09622	Dihydrolipoyl dehydrogenase, mitochondrial	1.77	0.142	4.1E-02	6	12.8
P07093	Glia-derived nexin	1.77	NA	NA	2	6.0
P52272	Heterogeneous nuclear ribonucleoprotein M	1.76	0.076	4.7E-02	10	15.8
P31153	S-adenosylmethionine synthase isoform type-2	1.76	0.239	9.4E-02	3	9.4
P01859	Ig gamma-2 chain C region	1.76	0.167	7.6E-02	3	12.0
P52597	Heterogeneous nuclear ribonucleoprotein F	1.76	0.132	1.1E-02	4	10.1
P46926	Glucosamine-6-phosphate isomerase 1	1.76	NA	NA	2	5.9
P36578	60S ribosomal protein L4	1.75	0.102	2.2E-03	7	17.8
Q1KMD3	Heterogeneous nuclear ribonucleoprotein U-like protein 2	1.75	0.191	7.8E-02	3	4.1
Q9NR30	Nucleolar RNA helicase 2	1.75	NA	NA	2	3.3
P11142	Heat shock cognate 71 kDa protein	1.74	0.058	5.9E-07	10	16.1
P60866	40S ribosomal protein S20	1.74	NA	NA	2	19.3
Q00059	Transcription factor A, mitochondrial	1.73	0.104	9.1E-03	4	16.7
P22626	Heterogeneous nuclear ribonucleoproteins A2/B1	1.73	0.080	6.4E-06	14	37.7
P62316	Small nuclear ribonucleoprotein Sm D2	1.73	NA	NA	2	16.1
Q14165	Malectin	1.72	NA	NA	2	10.6
Q92499	ATP-dependent RNA helicase DDX1	1.72	NA	NA	2	2.8
Q99729	Heterogeneous nuclear ribonucleoprotein A/B	1.71	NA	NA	2	3.9
P55265	Double-stranded RNA-specific adenosine deaminase	1.71	0.482	2.7E-01	3	2.7
P17844	Probable ATP-dependent RNA helicase DDX5	1.71	0.104	6.3E-02	4	6.4
P0CW22	40S ribosomal protein S17-like	1.71	NA	NA	2	15.6
Q15102	Platelet-activating factor acetylhydrolase IB subunit gamma	1.70	NA	NA	2	15.2
O00170	AH receptor-interacting protein	1.70	0.268	7.0E-02	5	18.5
P83731	60S ribosomal protein L24	1.70	0.298	8.7E-02	3	19.1
P51149	Ras-related protein Rab-7a	1.69	0.129	5.0E-04	7	35.7
P78527	DNA-dependent protein kinase catalytic subunit	1.69	0.045	1.7E-10	20	4.5
P39019	40S ribosomal protein S19	1.69	0.136	2.2E-03	4	19.3
P21281	V-type proton ATPase subunit B, brain isoform	1.69	NA	NA	2	5.1
Q5VTE0	Putative elongation factor 1-alpha-like 3	1.69	0.049	3.0E-06	10	21.4
P62857	40S ribosomal protein S28	1.68	NA	NA	2	30.4
P62277	40S ribosomal protein S13	1.68	0.107	2.8E-04	6	29.1
P06737	Glycogen phosphorylase, liver form	1.68	0.077	3.2E-05	15	19.7
Q9UKM9	RNA-binding protein Raly	1.67	0.081	3.0E-02	5	19.0
Q9NTK5	Obg-like ATPase 1	1.67	0.615	2.7E-01	3	10.4
Q99873	Protein arginine N-methyltransferase 1	1.66	NA	NA	2	7.5
Q00839	Heterogeneous nuclear ribonucleoprotein U	1.65	0.090	4.3E-04	8	9.6
P07919	Cytochrome b-c1 complex subunit 6, mitochondrial	1.65	NA	NA	2	15.4
P53985	Monocarboxylate transporter 1	1.65	NA	NA	2	6.4
P38646	Stress-70 protein, mitochondrial	1.65	0.102	6.1E-04	16	28.4
Q06323	Proteasome activator complex subunit 1	1.65	0.133	4.9E-03	5	21.7
P62888	60S ribosomal protein L30	1.64	NA	NA	2	20.9
P62424	60S ribosomal protein L7a	1.64	0.299	9.8E-02	6	20.3
Q8IWB7	WD repeat and FYVE domain-containing protein 1	1.64	NA	NA	2	5.1
P55209	Nucleosome assembly protein 1-like 1	1.63	0.056	5.2E-03	3	12.0
P50402	Emerin	1.63	NA	NA	2	9.8
P60981	Destrin	1.63	0.175	4.6E-02	3	18.2
P06753	Tropomyosin alpha-3 chain	1.63	0.169	3.5E-03	4	13.0
Q12874	Splicing factor 3A subunit 3	1.62	0.502	2.2E-01	3	5.8
Q15717	ELAV-like protein 1	1.62	0.344	1.2E-01	5	19.0
P62899	60S ribosomal protein L31	1.61	NA	NA	2	13.6
P62158	Calmodulin	1.61	0.159	1.0E-02	3	22.1
P20700	Lamin-B1	1.61	0.155	2.2E-02	12	21.3
P46937	Yorkie homolog	1.61	NA	NA	2	6.7
P62318	Small nuclear ribonucleoprotein Sm D3	1.60	NA	NA	2	23.8
Q8N766	ER membrane protein complex subunit 1	1.60	NA	NA	2	2.4
P13489	Ribonuclease inhibitor	1.60	0.244	1.9E-01	3	6.1
Q02543	60S ribosomal protein L18a	1.59	NA	NA	2	10.2
Q96C86	m7GpppX diphosphatase	1.59	NA	NA	2	7.1
Q99623	Prohibitin-2	1.59	0.042	5.8E-06	7	24.7
P61981	14-3-3 protein gamma	1.59	NA	NA	2	9.7
P04075	Fructose-bisphosphate aldolase A	1.59	0.073	2.8E-04	11	37.9
P08107	Heat shock 70 kDa protein 1A/1B	1.57	0.056	1.1E-07	14	23.2
P35232	Prohibitin	1.56	0.049	2.0E-07	7	25.4
O14773	Tripeptidyl-peptidase 1	1.56	0.188	1.4E-02	4	8.7
Q07960	Rho GTPase-activating protein 1	1.55	NA	NA	2	3.6
P14854	Cytochrome c oxidase subunit 6B1	1.55	NA	NA	2	24.4
Q15365	Poly(rC)-binding protein 1	1.54	0.097	2.2E-03	5	16.3
P04792	Heat shock protein beta-1	1.54	0.137	1.5E-02	6	31.2
Q13428	Treacle protein	1.54	0.133	6.4E-02	3	2.3
O00560	Syntenin-1	1.54	0.411	2.8E-01	4	11.4
O60506	Heterogeneous nuclear ribonucleoprotein Q	1.54	0.166	2.0E-02	4	5.8
Q00341	Vigilin	1.54	NA	NA	2	1.0
Q9Y6M9	NADH dehydrogenase [ubiquinone] 1 beta subcomplex subunit 9	1.54	NA	NA	2	15.6
Q13347	Eukaryotic translation initiation factor 3 subunit I	1.53	NA	NA	2	7.1
Q92841	Probable ATP-dependent RNA helicase DDX17	1.53	0.175	2.8E-02	8	12.3
P02766	Transthyretin	1.52	NA	NA	2	24.5
P09661	U2 small nuclear ribonucleoprotein A'	1.52	0.167	5.9E-02	3	12.9
O75643	U5 small nuclear ribonucleoprotein 200 kDa helicase	1.52	0.202	3.7E-02	7	3.1
P62847	40S ribosomal protein S24	1.51	NA	NA	2	9.0
P02652	Apolipoprotein A-II	1.51	1.149	3.0E-01	3	21.0
Q9HD20	Manganese-transporting ATPase 13A1	1.50	NA	NA	2	2.6
P07602	Prosaposin	1.50	0.135	3.7E-02	4	5.3
P14406	Cytochrome c oxidase subunit 7A2, mitochondrial	1.50	NA	NA	2	27.7
P13797	Plastin-3	1.50	NA	NA	2	4.0
P08865	40S ribosomal protein SA	1.50	0.064	1.6E-03	6	24.4
O95336	6-phosphogluconolactonase	1.50	0.356	2.2E-01	4	20.9
P62249	40S ribosomal protein S16	1.50	0.127	1.9E-03	4	26.7
Q15907	Ras-related protein Rab-11B	1.49	0.106	2.6E-02	4	17.0
P69905	Hemoglobin subunit alpha	1.49	0.105	5.5E-04	5	53.5
P09874	Poly [ADP-ribose] polymerase 1	1.49	0.075	1.5E-03	7	7.9
Q9Y4I1	Unconventional myosin-Va	1.48	0.119	1.5E-02	9	5.0
P02042	Hemoglobin subunit delta	1.48	NA	NA	2	17.7
P40429	60S ribosomal protein L13a	1.48	0.136	1.0E-02	7	26.6
Q96I99	Succinyl-CoA ligase [GDP-forming] subunit beta, mitochondrial	1.48	NA	NA	2	4.6
Q13310	Polyadenylate-binding protein 4	1.48	NA	NA	2	3.6
P27635	60S ribosomal protein L10	1.47	0.075	1.3E-02	5	22.0
P39023	60S ribosomal protein L3	1.47	0.271	1.5E-01	5	10.9
P49327	Fatty acid synthase	1.47	NA	NA	2	1.0
P46779	60S ribosomal protein L28	1.46	0.096	1.9E-02	3	19.0

Q9Y3U8	60S ribosomal protein L36	1.45	0.121	6.1E-03	5	36.2
Q9UNF0	Protein kinase C and casein kinase substrate in neurons protein 2	1.45	0.109	1.1E-01	3	6.8
Q9BW27	Nuclear pore complex protein Nup85	1.45	NA	NA	2	2.6
P46109	Crk-like protein	1.44	NA	NA	2	7.9
P35268	60S ribosomal protein L22	1.44	NA	NA	2	18.8
P61978	Heterogeneous nuclear ribonucleoprotein K	1.44	0.078	3.5E-04	13	32.4
P02774	Vitamin D-binding protein	1.44	0.161	3.5E-02	6	8.6
P14866	Heterogeneous nuclear ribonucleoprotein L	1.44	0.160	3.3E-02	8	19.5
P30101	Protein disulfide-isomerase A3	1.44	0.073	2.6E-05	16	28.9
O14979	Heterogeneous nuclear ribonucleoprotein D-like	1.43	NA	NA	2	2.1
P23396	40S ribosomal protein S3	1.43	0.034	4.9E-09	9	37.0
P07339	Cathepsin D	1.42	0.096	3.5E-03	4	9.7
P62851	40S ribosomal protein S25	1.42	0.159	1.9E-02	3	24.0
P30837	Aldehyde dehydrogenase X, mitochondrial	1.42	0.189	9.1E-02	5	14.1
P84103	Serine/arginine-rich splicing factor 3	1.42	0.329	1.9E-01	3	20.7
P15880	40S ribosomal protein S2	1.42	0.124	1.9E-02	4	16.0
Q16666	Gamma-interferon-inducible protein 16	1.41	NA	NA	2	2.5
P61421	V-type proton ATPase subunit d 1	1.41	0.066	4.8E-03	3	7.4
O75390	Citrate synthase, mitochondrial	1.41	0.345	2.9E-01	4	8.4
P49189	4-trimethylaminobutyraldehyde dehydrogenase	1.41	NA	NA	2	3.6
P18124	60S ribosomal protein L7	1.41	0.051	5.2E-05	5	15.7
Q9ULV4	Coronin-1C	1.40	2.010	5.9E-01	3	6.5
P30050	60S ribosomal protein L12	1.40	0.247	1.2E-01	4	35.8
Q9Y6U3	Adseverin	1.40	0.291	7.3E-01	4	4.9
O75494	Serine/arginine-rich splicing factor 10	1.40	NA	NA	2	8.4
O75367	Core histone macro-H2A.1	1.40	0.165	5.8E-03	9	22.0
Q86U42	Polyadenylate-binding protein 2	1.40	0.094	2.2E-02	4	12.4
P62280	40S ribosomal protein S11	1.39	0.372	3.3E-01	4	22.8
P10155	60 kDa SS-A/Ro ribonucleoprotein	1.39	NA	NA	2	4.5
Q9Y262	Eukaryotic translation initiation factor 3 subunit L	1.39	0.226	9.8E-02	6	10.3
P32969	60S ribosomal protein L9	1.39	0.076	3.4E-02	5	22.9
Q9P2E9	Ribosome-binding protein 1	1.39	0.709	1.3E-01	8	7.0
O00231	26S proteasome non-ATPase regulatory subunit 11	1.38	0.244	9.8E-02	5	12.6
P11586	C-1-tetrahydrofolate synthase, cytoplasmic	1.38	0.233	6.4E-02	5	6.3
Q16543	Hsp90 co-chaperone Cdc37	1.38	NA	NA	2	5.3
Q14103	Heterogeneous nuclear ribonucleoprotein D0	1.38	NA	NA	2	6.8
P11940	Polyadenylate-binding protein 1	1.38	0.069	2.1E-03	6	11.3
Q13263	Transcription intermediary factor 1-beta	1.38	0.080	9.1E-03	4	5.3
Q15942	Zyxin	1.37	NA	NA	2	5.4
Q96KP4	Cytosolic non-specific dipeptidase	1.37	0.070	9.0E-03	4	11.8
P37802	Transgelin-2	1.37	NA	NA	2	10.1
O14561	Acyl carrier protein, mitochondrial	1.37	NA	NA	2	9.6
P30533	Alpha-2-macroglobulin receptor-associated protein	1.36	0.509	2.9E-01	4	9.0
Q9Y4W6	AFG3-like protein 2	1.36	0.472	2.2E-01	7	10.0
P52566	Rho GDP-dissociation inhibitor 2	1.36	NA	NA	2	15.4
B5ME19	Eukaryotic translation initiation factor 3 subunit C-like protein	1.36	NA	NA	2	2.2
P36543	V-type proton ATPase subunit E 1	1.35	0.372	3.4E-01	3	12.4
P55786	Puromycin-sensitive aminopeptidase	1.35	0.193	1.0E-01	5	6.3
P50213	Isocitrate dehydrogenase [NAD] subunit alpha, mitochondrial	1.34	0.101	3.1E-02	3	10.1
P12830	Cadherin-1	1.34	NA	NA	2	2.7
P12270	Nucleoprotein TPR	1.34	0.172	7.1E-02	13	7.2
P27144	Adenylate kinase 4, mitochondrial	1.34	NA	NA	2	9.9
P61758	Prefoldin subunit 3	1.34	NA	NA	2	8.1
P62269	40S ribosomal protein S18	1.34	0.184	8.9E-02	6	31.6
P38606	V-type proton ATPase catalytic subunit A	1.33	0.147	7.4E-02	3	5.7
P30041	Peroxiredoxin-6	1.33	0.111	1.4E-02	7	25.4
O14949	Cytochrome b-c1 complex subunit 8	1.33	NA	NA	2	25.6
O43681	ATPase ASNA1	1.33	NA	NA	2	4.6
P62913	60S ribosomal protein L11	1.33	0.038	5.6E-03	3	16.9
P27824	Calnexin	1.33	0.258	5.8E-02	11	20.8
P37837	Transaldolase	1.32	0.212	3.2E-01	5	14.2
Q9GZS3	WD repeat-containing protein 61	1.31	NA	NA	2	7.5
Q8N1F7	Nuclear pore complex protein Nup93	1.31	NA	NA	2	3.2
Q99536	Synaptic vesicle membrane protein VAT-1 homolog	1.31	0.179	3.7E-02	10	28.2
P45880	Voltage-dependent anion-selective channel protein 2	1.31	0.093	1.7E-03	6	21.8
P53396	ATP-citrate synthase	1.31	NA	NA	2	2.6
Q13098	COP9 signalosome complex subunit 1	1.30	NA	NA	2	3.3
Q9NSD9	Phenylalanine--tRNA ligase beta subunit	1.30	0.793	6.9E-01	3	5.4
O75380	NADH dehydrogenase [ubiquinone] iron-sulfur protein 6, mitochondrial	1.30	NA	NA	2	20.2
Q04837	Single-stranded DNA-binding protein, mitochondrial	1.30	NA	NA	2	11.5
P26599	Polypyrimidine tract-binding protein 1	1.30	0.076	1.2E-02	5	8.3
Q7KZF4	Staphylococcal nuclease domain-containing protein 1	1.30	0.104	4.2E-02	4	6.5
P47813	Eukaryotic translation initiation factor 1A, X-chromosomal	1.30	NA	NA	2	15.3
P35613	Basigin	1.29	NA	NA	2	8.3
P42167	Lamina-associated polypeptide 2, isoforms beta/gamma	1.29	NA	NA	2	4.6
P26885	Peptidyl-prolyl cis-trans isomerase FKBP2	1.29	NA	NA	2	14.1
Q9NVI7	ATPase family AAA domain-containing protein 3A	1.29	NA	NA	2	3.9
P35580	Myosin-10	1.29	0.075	1.1E-04	32	17.6
P49755	Transmembrane emp24 domain-containing protein 10	1.29	0.118	8.0E-02	3	14.6
O43852	Calumenin	1.28	0.091	3.8E-02	6	25.7
Q15424	Scaffold attachment factor B1	1.28	NA	NA	2	1.9
P35998	26S protease regulatory subunit 7	1.28	NA	NA	2	5.8
Q92597	Protein NDRG1	1.28	0.038	6.6E-03	3	9.1
P30519	Heme oxygenase 2	1.28	0.683	3.1E-01	3	15.8
Q16891	Mitochondrial inner membrane protein	1.28	0.112	2.3E-02	8	14.6
Q92599	Septin-8	1.28	NA	NA	2	6.0
P40616	ADP-ribosylation factor-like protein 1	1.27	NA	NA	2	12.7
O75694	Nuclear pore complex protein Nup155	1.27	NA	NA	2	1.6
P31689	DnaJ homolog subfamily A member 1	1.27	0.178	2.7E-01	3	6.8
P21266	Glutathione S-transferase Mu 3	1.27	NA	NA	2	8.4
P14868	Aspartate--tRNA ligase, cytoplasmic	1.26	0.207	1.2E-01	5	13.2
P51532	Transcription activator BRG1	1.26	NA	NA	2	1.7
Q13011	Delta(3,5)-Delta(2,4)-dienoyl-CoA isomerase, mitochondrial	1.26	0.221	1.7E-01	3	7.6
Q15063	Periostin	1.26	0.044	7.5E-03	4	6.5
Q99798	Aconitate hydratase, mitochondrial	1.26	0.483	9.8E-02	7	13.3
P49588	Alanine--tRNA ligase, cytoplasmic	1.25	NA	NA	2	3.2
P00747	Plasminogen	1.25	0.084	3.9E-02	9	14.9
P08574	Cytochrome c1, heme protein, mitochondrial	1.25	0.125	3.3E-02	4	17.2
P53597	Succinyl-CoA ligase [ADP/GDP-forming] subunit alpha, mitochondrial	1.25	NA	NA	2	6.9
P37108	Signal recognition particle 14 kDa protein	1.25	0.202	3.5E-01	3	24.3
P46782	40S ribosomal protein S5	1.24	0.048	1.1E-02	4	16.2
O15145	Actin-related protein 2/3 complex subunit 3	1.24	NA	NA	2	9.6
Q86VP6	Cullin-associated NEDD8-dissociated protein 1	1.24	NA	NA	2	1.9
P30153	Serine/threonine-protein phosphatase 2A 65 kDa regulatory subunit A alpha isoform	1.24	0.032	3.5E-02	3	7.0
P08195	4F2 cell-surface antigen heavy chain	1.24	0.128	6.8E-02	5	9.0
P62241	40S ribosomal protein S8	1.23	0.111	1.3E-01	3	11.5
Q07065	Cytoskeleton-associated protein 4	1.22	0.675	1.5E-01	10	21.9
Q5JTV8	Torsin-1A-interacting protein 1	1.22	0.104	1.7E-01	3	5.7
Q9NP81	Serine--tRNA ligase, mitochondrial	1.22	NA	NA	2	6.9
P67936	Tropomyosin alpha-4 chain	1.22	0.218	7.3E-02	6	23.0
P62753	40S ribosomal protein S6	1.22	0.153	8.6E-02	3	12.9
P49411	Elongation factor Tu, mitochondrial	1.22	0.096	3.5E-02	13	32.7
Q13363	C-terminal-binding protein 1	1.21	NA	NA	2	4.3
P61158	Actin-related protein 3	1.21	0.038	4.5E-03	5	13.9
O15144	Actin-related protein 2/3 complex subunit 2	1.21	1.034	4.3E-01	5	17.0

Q13547	Histone deacetylase 1	1.20	NA	NA	2	4.1
P22314	Ubiquitin-like modifier-activating enzyme 1	1.20	0.105	1.4E-01	7	7.7
Q05682	Caldesmon	1.20	0.636	3.4E-01	5	8.8
Q9Y512	Sorting and assembly machinery component 50 homolog	1.20	NA	NA	2	3.8
P27797	Calreticulin	1.20	1.188	5.0E-01	8	22.3
P40227	T-complex protein 1 subunit zeta	1.20	0.067	6.0E-02	6	12.8
Q9BVJ6	U3 small nucleolar RNA-associated protein 14 homolog A	1.20	NA	NA	2	4.0
P62917	60S ribosomal protein L8	1.20	NA	NA	2	10.5
Q5H9R7	Serine/threonine-protein phosphatase 6 regulatory subunit 3	1.19	NA	NA	2	2.6
P09012	U1 small nuclear ribonucleoprotein A	1.19	NA	NA	2	7.4
O94826	Mitochondrial import receptor subunit TOM70	1.19	0.465	2.9E-01	5	9.7
P19367	Hexokinase-1	1.19	0.117	2.0E-01	5	6.0
Q15008	26S proteasome non-ATPase regulatory subunit 6	1.19	NA	NA	2	5.4
Q9NTJ5	Phosphatidylinositide phosphatase SAC1	1.19	0.150	1.3E-01	4	7.2
P20340	Ras-related protein Rab-6A	1.18	NA	NA	2	10.6
Q9BVK6	Transmembrane emp24 domain-containing protein 9	1.18	0.283	3.4E-01	3	10.6
P09669	Cytochrome c oxidase subunit 6C	1.18	0.165	2.7E-01	3	32.0
Q15691	Microtubule-associated protein RP/EB family member 1	1.18	0.030	1.4E-02	3	8.6
P62701	40S ribosomal protein S4, X isoform	1.18	0.173	1.6E-01	7	24.3
Q63HN8	E3 ubiquitin-protein ligase RNF213	1.18	NA	NA	2	0.4
P42765	3-ketoacyl-CoA thiolase, mitochondrial	1.18	0.258	2.0E-01	6	18.9
P28838	Cytosol aminopeptidase	1.18	0.475	6.3E-01	6	15.2
Q9BQG0	Myb-binding protein 1A	1.18	NA	NA	2	2.0
O43399	Tumor protein D54	1.17	NA	NA	2	7.8
Q8N5K1	CDGSH iron-sulfur domain-containing protein 2	1.17	0.097	1.5E-01	3	26.7
P35637	RNA-binding protein FUS	1.17	0.386	2.5E-01	3	6.8
Q9UQE7	Structural maintenance of chromosomes protein 3	1.16	NA	NA	2	1.4
Q96HE7	ERO1-like protein alpha	1.16	NA	NA	2	5.3
P14927	Cytochrome b-c1 complex subunit 7	1.16	0.176	3.9E-01	3	28.8
Q16851	UTP--glucose-1-phosphate uridylyltransferase	1.16	0.883	4.1E-01	7	15.4
Q8NC56	LEM domain-containing protein 2	1.16	0.041	3.3E-02	6	11.3
P48047	ATP synthase subunit O, mitochondrial	1.15	0.856	1.6E-01	5	30.0
P51572	B-cell receptor-associated protein 31	1.15	0.167	2.5E-01	7	25.6
O60568	Procollagen-lysine,2-oxoglutarate 5-dioxygenase 3	1.15	NA	NA	2	4.6
Q15029	116 kDa U5 small nuclear ribonucleoprotein component	1.15	0.061	1.1E-01	3	3.4
P13073	Cytochrome c oxidase subunit 4 isoform 1, mitochondrial	1.15	0.085	6.8E-02	4	25.4
P07237	Protein disulfide-isomerase	1.15	0.176	1.0E-01	13	21.5
Q15084	Protein disulfide-isomerase A6	1.14	0.248	1.5E-01	8	25.9
P25685	DnaJ homolog subfamily B member 1	1.14	NA	NA	2	6.8
O60664	Perilipin-3	1.13	NA	NA	2	7.4
P36776	Lon protease homolog, mitochondrial	1.13	NA	NA	2	2.7
Q13162	Peroxiredoxin-4	1.13	NA	NA	2	8.9
O60716	Catenin delta-1	1.13	1.751	7.6E-01	3	4.1
Q9GZR7	ATP-dependent RNA helicase DDX24	1.13	0.624	5.6E-01	3	5.0
O75915	PRA1 family protein 3	1.12	0.377	7.8E-01	3	19.7
Q00765	Receptor expression-enhancing protein 5	1.12	0.397	6.9E-01	3	10.6
P48735	Isocitrate dehydrogenase [NADP], mitochondrial	1.12	0.218	3.7E-01	8	19.0
Q86UP2	Kinectin	1.12	0.191	5.0E-01	6	5.5
P11234	Ras-related protein Ral-B	1.12	NA	NA	2	12.1
O94906	Pre-mRNA-processing factor 6	1.12	NA	NA	2	3.0
P68871	Hemoglobin subunit beta	1.12	0.459	1.8E-01	4	32.0
Q92542	Nicastrin	1.12	NA	NA	2	2.7
P30740	Leukocyte elastase inhibitor	1.11	0.835	5.9E-01	4	14.5
P46940	Ras GTPase-activating-like protein IQGAP1	1.11	0.392	4.8E-01	6	4.0
P51810	G-protein coupled receptor 143	1.11	0.205	4.1E-01	3	9.9
Q02818	Nucleobindin-1	1.10	0.192	4.3E-01	4	11.7
P35659	Protein DEK	1.10	NA	NA	2	6.9
P05091	Aldehyde dehydrogenase, mitochondrial	1.10	0.254	4.3E-01	5	10.6
Q9NX63	Coiled-coil-helix-coiled-coil-helix domain-containing protein 3, mitochondrial	1.10	0.406	4.9E-01	3	12.3
P51148	Ras-related protein Rab-5C	1.09	NA	NA	2	10.6
Q99832	T-complex protein 1 subunit eta	1.09	0.035	6.5E-02	6	11.4
Q14152	Eukaryotic translation initiation factor 3 subunit A	1.09	0.177	5.7E-01	10	8.7
Q9NSE4	Isoleucine--tRNA ligase, mitochondrial	1.09	0.234	6.4E-01	5	7.1
O15511	Actin-related protein 2/3 complex subunit 5	1.09	NA	NA	2	11.9
O00571	ATP-dependent RNA helicase DDX3X	1.08	3.908	8.5E-01	3	5.7
P22307	Non-specific lipid-transfer protein	1.08	0.344	8.4E-01	3	4.8
P62081	40S ribosomal protein S7	1.08	NA	NA	2	9.3
P47985	Cytochrome b-c1 complex subunit Rieske, mitochondrial	1.08	0.060	2.2E-01	3	11.3
P46977	Dolichyl-diphosphooligosaccharide--protein glycosyltransferase subunit STT3A	1.07	NA	NA	2	2.3
P62244	40S ribosomal protein S15a	1.07	0.097	3.0E-01	4	29.2
P27708	CAD protein	1.07	NA	NA	2	0.9
P49458	Signal recognition particle 9 kDa protein	1.07	NA	NA	2	22.1
P46781	40S ribosomal protein S9	1.07	0.618	7.6E-01	6	27.3
P78347	General transcription factor II-I	1.07	0.847	8.3E-01	4	4.0
P50990	T-complex protein 1 subunit theta	1.07	0.101	2.5E-01	13	24.8
P04844	Dolichyl-diphosphooligosaccharide--protein glycosyltransferase subunit 2	1.06	NA	NA	2	4.1
O60271	C-Jun-amino-terminal kinase-interacting protein 4	1.06	NA	NA	2	2.1
P63279	SUMO-conjugating enzyme UBC9	1.06	0.171	6.6E-01	3	22.8
O75131	Copine-3	1.06	11.287	7.4E-01	4	6.7
Q9Y6C9	Mitochondrial carrier homolog 2	1.06	NA	NA	2	11.2
P09525	Annexin A4	1.06	0.245	5.4E-01	8	23.8
Q14956	Transmembrane glycoprotein NMB	1.06	0.938	8.5E-01	5	10.0
P82909	28S ribosomal protein S36, mitochondrial	1.06	NA	NA	2	24.3
P61160	Actin-related protein 2	1.06	0.210	4.2E-01	6	17.3
P22695	Cytochrome b-c1 complex subunit 2, mitochondrial	1.06	1.318	7.5E-01	6	18.3
P01857	Ig gamma-1 chain C region	1.05	NA	NA	2	7.9
O95292	Vesicle-associated membrane protein-associated protein B/C	1.05	0.490	7.8E-01	4	21.0
Q06830	Peroxiredoxin-1	1.05	0.764	6.0E-01	8	36.7
O60814	Histone H2B type 1-K	1.05	NA	NA	2	7.9
O75323	Protein NipSnap homolog 2	1.04	NA	NA	2	8.7
Q9H2G2	STE20-like serine/threonine-protein kinase	1.04	0.629	8.7E-01	3	3.0
P01892	HLA class I histocompatibility antigen, A-2 alpha chain	1.04	1.390	8.5E-01	4	13.7
P59998	Actin-related protein 2/3 complex subunit 4	1.04	0.452	6.5E-01	3	16.1
P31942	Heterogeneous nuclear ribonucleoprotein H3	1.04	0.238	8.0E-01	3	11.3
P30405	Peptidyl-prolyl cis-trans isomerase F, mitochondrial	1.04	NA	NA	2	8.7
P19823	Inter-alpha-trypsin inhibitor heavy chain H2	1.04	NA	NA	2	2.4
P47897	Glutamine--tRNA ligase	1.04	0.175	7.9E-01	3	3.9
Q99733	Nucleosome assembly protein 1-like 4	1.03	NA	NA	2	7.7
P49368	T-complex protein 1 subunit gamma	1.03	0.145	6.5E-01	9	20.0
Q15370	Transcription elongation factor B polypeptide 2	1.02	0.779	8.5E-01	3	23.7
P31930	Cytochrome b-c1 complex subunit 1, mitochondrial	1.02	5.017	8.8E-01	4	9.8
P53990	IST1 homolog	1.02	NA	NA	2	5.5
P20810	Calpastatin	1.02	0.950	9.8E-01	3	6.2
Q00325	Phosphate carrier protein, mitochondrial	1.02	0.132	7.9E-01	5	12.2
P78371	T-complex protein 1 subunit beta	1.02	0.909	9.1E-01	9	17.9
P30049	ATP synthase subunit delta, mitochondrial	1.01	NA	NA	2	13.7
Q13200	26S proteasome non-ATPase regulatory subunit 2	1.01	0.385	9.6E-01	3	3.4
P30048	Thioredoxin-dependent peroxide reductase, mitochondrial	1.01	0.272	9.3E-01	5	20.3
Q9Y394	Dehydrogenase/reductase SDR family member 7	1.01	NA	NA	2	5.0
O43390	Heterogeneous nuclear ribonucleoprotein R	1.01	0.396	9.6E-01	6	11.5
Q14980	Nuclear mitotic apparatus protein 1	1.01	0.435	9.1E-01	5	3.3
P02786	Transferrin receptor protein 1	1.00	NA	NA	2	3.2
Q8N1G4	Leucine-rich repeat-containing protein 47	1.00	1.001	9.9E-01	3	6.7
P36955	Pigment epithelium-derived factor	1.00	NA	NA	2	5.7
P51812	Ribosomal protein S6 kinase alpha-3	1.00	5.093	1.0E+00	3	4.1

P08237	ATP-dependent 6-phosphofructokinase, muscle type	0.99	1.158	9.7E-01	5	7.8
Q7L014	Probable ATP-dependent RNA helicase DDX46	0.99	0.424	9.8E-01	3	2.8
O75947	ATP synthase subunit d, mitochondrial	0.99	2.037	9.7E-01	4	22.4
P55884	Eukaryotic translation initiation factor 3 subunit B	0.99	NA	NA	2	2.3
P22061	Protein-L-isoaspartate(D-aspartate) O-methyltransferase	0.99	NA	NA	2	13.2
O95202	LETM1 and EF-hand domain-containing protein 1, mitochondrial	0.99	NA	NA	2	3.9
O75832	26S proteasome non-ATPase regulatory subunit 10	0.99	NA	NA	2	11.9
P48643	T-complex protein 1 subunit epsilon	0.99	1.585	8.4E-01	14	21.8
P13674	Prolyl 4-hydroxylase subunit alpha-1	0.99	NA	NA	2	5.8
P21291	Cysteine and glycine-rich protein 1	0.98	NA	NA	2	22.8
Q8TD19	Serine/threonine-protein kinase Nek9	0.98	NA	NA	2	2.2
P54136	Arginine--tRNA ligase, cytoplasmic	0.98	0.496	9.0E-01	3	5.5
O00264	Membrane-associated progesterone receptor component 1	0.98	0.725	9.1E-01	4	16.4
P09914	Interferon-induced protein with tetratricopeptide repeats 1	0.98	3.223	9.5E-01	3	8.8
P30626	Sorcin	0.98	NA	NA	2	10.6
P31040	Succinate dehydrogenase [ubiquinone] flavoprotein subunit, mitochondrial	0.97	0.626	9.3E-01	4	9.0
Q3ZCQ8	Mitochondrial import inner membrane translocase subunit TIM50	0.97	NA	NA	2	5.1
Q9Y230	RuvB-like 2	0.97	0.364	8.3E-01	3	7.3
P53618	Coatomer subunit beta	0.97	0.129	8.5E-01	4	4.9
Q9BSF4	Uncharacterized protein C19orf52	0.97	NA	NA	2	11.5
P0C0S5	Histone H2A.Z	0.97	0.176	7.1E-01	4	31.3
Q9P0M6	Core histone macro-H2A.2	0.96	2.553	9.1E-01	3	12.6
P49591	Serine--tRNA ligase, cytoplasmic	0.96	NA	NA	2	5.3
P02647	Apolipoprotein A-I	0.96	0.902	7.0E-01	4	15.4
Q9BXF6	Rab11 family-interacting protein 5	0.96	NA	NA	2	4.6
P50502	Hsc70-interacting protein	0.96	0.847	9.0E-01	3	7.0
Q16698	2,4-dienoyl-CoA reductase, mitochondrial	0.95	0.411	9.3E-01	3	10.7
Q52LJ0	Protein FAM98B	0.95	NA	NA	2	7.0
P12268	Inosine-5'-monophosphate dehydrogenase 2	0.95	0.045	3.0E-01	3	9.1
P14314	Glucosidase 2 subunit beta	0.95	18.388	6.1E-01	5	9.8
O43678	NADH dehydrogenase [ubiquinone] 1 alpha subcomplex subunit 2	0.95	NA	NA	2	11.1
Q14315	Filamin-C	0.95	NA	NA	2	1.0
Q9P0K7	Ankycorbin	0.94	NA	NA	2	1.8
Q93050	V-type proton ATPase 116 kDa subunit a isoform 1	0.94	0.119	5.9E-01	4	6.6
P01009	Alpha-1-antitrypsin	0.94	0.457	6.4E-01	10	22.7
O75306	NADH dehydrogenase [ubiquinone] iron-sulfur protein 2, mitochondrial	0.94	0.022	1.1E-01	3	6.7
Q14683	Structural maintenance of chromosomes protein 1A	0.94	0.226	5.7E-01	4	4.0
P51608	Methyl-CpG-binding protein 2	0.94	0.228	5.5E-01	3	8.0
Q14697	Neutral alpha-glucosidase AB	0.93	0.164	5.6E-01	10	10.8
Q6P2Q9	Pre-mRNA-processing-splicing factor 8	0.93	0.123	4.3E-01	3	1.3
P04843	Dolichyl-diphosphooligosaccharide--protein glycosyltransferase subunit 1	0.93	0.120	4.0E-01	12	22.6
P38117	Electron transfer flavoprotein subunit beta	0.93	NA	NA	2	7.8
Q8NBS9	Thioredoxin domain-containing protein 5	0.93	3.633	6.6E-01	5	10.0
P05090	Apolipoprotein D	0.93	0.239	6.1E-01	3	16.4
Q8IVF2	Protein AHNAK2	0.93	NA	NA	2	0.4
P40763	Signal transducer and activator of transcription 3	0.93	2.009	8.7E-01	3	3.9
P09496	Clathrin light chain A	0.92	0.216	3.3E-01	4	12.5
P50991	T-complex protein 1 subunit delta	0.92	0.630	4.5E-01	7	16.3
Q96TC7	Regulator of microtubule dynamics protein 3	0.91	NA	NA	2	4.0
O75340	Programmed cell death protein 6	0.91	NA	NA	2	9.9
Q53H12	Acylglycerol kinase, mitochondrial	0.91	NA	NA	2	5.9
Q9UIQ6	Leucyl-cystinyl aminopeptidase	0.91	NA	NA	2	2.7
P62979	Ubiquitin-40S ribosomal protein S27a	0.91	0.262	2.4E-01	9	41.0
P06576	ATP synthase subunit beta, mitochondrial	0.90	0.139	6.1E-02	11	26.5
P11021	78 kDa glucose-regulated protein	0.90	0.139	9.5E-02	17	25.8
P43490	Nicotinamide phosphoribosyltransferase	0.89	0.958	7.6E-01	4	8.4
P16435	NADPH--cytochrome P450 reductase	0.89	0.168	4.0E-01	3	5.8
Q15293	Reticulocalbin-1	0.89	0.317	5.0E-01	4	11.5
Q15413	Ryanodine receptor 3	0.89	NA	NA	2	0.3
P43686	26S protease regulatory subunit 6B	0.88	0.137	2.9E-01	3	8.4
P10606	Cytochrome c oxidase subunit 5B, mitochondrial	0.88	0.099	1.0E-01	6	31.0
P61586	Transforming protein RhoA	0.88	0.114	1.2E-01	3	10.4
P01834	Ig kappa chain C region	0.88	0.203	3.0E-01	3	48.1
P07384	Calpain-1 catalytic subunit	0.87	0.126	2.3E-01	3	4.8
P02545	Prelamin-A/C	0.87	0.102	4.0E-03	33	46.5
P21912	Succinate dehydrogenase [ubiquinone] iron-sulfur subunit, mitochondrial	0.87	0.147	2.1E-01	3	11.4
P63000	Ras-related C3 botulinum toxin substrate 1	0.87	0.097	2.0E-01	4	24.5
P00505	Aspartate aminotransferase, mitochondrial	0.86	0.180	1.3E-01	5	13.5
Q15075	Early endosome antigen 1	0.86	NA	NA	2	1.8
P14625	Endoplasmin	0.86	0.089	8.8E-03	17	21.5
O00429	Dynamin-1-like protein	0.86	NA	NA	2	3.5
P04040	Catalase	0.86	0.063	1.7E-01	4	10.1
P51116	Fragile X mental retardation syndrome-related protein 2	0.86	NA	NA	2	4.0
P36542	ATP synthase subunit gamma, mitochondrial	0.86	NA	NA	2	7.0
P67870	Casein kinase II subunit beta	0.86	0.996	6.1E-01	3	12.6
P17987	T-complex protein 1 subunit alpha	0.86	0.153	8.1E-02	7	13.3
Q9UGP8	Translocation protein SEC63 homolog	0.86	NA	NA	2	3.0
P11177	Pyruvate dehydrogenase E1 component subunit beta, mitochondrial	0.85	NA	NA	2	4.2
Q8N0X7	Spartin	0.85	NA	NA	2	3.2
Q9UHD8	Septin-9	0.85	0.198	3.5E-01	5	11.1
Q14108	Lysosome membrane protein 2	0.85	0.201	2.5E-01	3	6.3
P29966	Myristoylated alanine-rich C-kinase substrate	0.85	0.333	6.6E-01	5	22.0
P26038	Moesin	0.85	0.076	3.0E-02	8	13.0
P40939	Trifunctional enzyme subunit alpha, mitochondrial	0.85	0.190	2.7E-01	14	20.4
Q01844	RNA-binding protein EWS	0.84	0.219	3.1E-01	3	3.4
P13667	Protein disulfide-isomerase A4	0.84	0.342	2.7E-01	8	14.7
O96000	NADH dehydrogenase [ubiquinone] 1 beta subcomplex subunit 10	0.84	NA	NA	2	12.2
P43304	Glycerol-3-phosphate dehydrogenase, mitochondrial	0.84	0.113	1.4E-01	4	6.5
P04632	Calpain small subunit 1	0.84	0.057	1.7E-02	5	16.0
Q8IZP0	Abl interactor 1	0.84	NA	NA	2	4.9
Q13561	Dynactin subunit 2	0.83	0.210	3.1E-01	4	12.2
P17858	ATP-dependent 6-phosphofructokinase, liver type	0.83	0.099	1.4E-01	4	6.9
A1L0T0	Acetolactate synthase-like protein	0.83	NA	NA	2	5.1
P25705	ATP synthase subunit alpha, mitochondrial	0.83	0.092	6.6E-03	16	31.1
P24539	ATP synthase F(0) complex subunit B1, mitochondrial	0.83	0.100	1.5E-01	3	13.7
P01042	Kininogen-1	0.82	NA	NA	2	1.4
P43307	Translocon-associated protein subunit alpha	0.82	NA	NA	2	6.6
P61225	Ras-related protein Rap-2b	0.81	NA	NA	2	10.4
P49257	Protein ERGIC-53	0.81	0.051	4.2E-02	3	8.0
P15311	Ezrin	0.81	0.154	3.2E-01	3	3.8
P60953	Cell division control protein 42 homolog	0.80	NA	NA	2	12.6
P07951	Tropomyosin beta chain	0.80	NA	NA	2	7.0
Q7L5N1	COP9 signalosome complex subunit 6	0.80	0.025	1.7E-02	3	10.4
Q96N67	Dedicator of cytokinesis protein 7	0.80	0.517	6.3E-01	3	2.0
P13861	cAMP-dependent protein kinase type II-alpha regulatory subunit	0.79	0.256	8.9E-02	5	15.1
Q9BRX8	Redox-regulatory protein FAM213A	0.79	NA	NA	2	8.7
Q99805	Transmembrane 9 superfamily member 2	0.79	NA	NA	2	3.5
P10644	cAMP-dependent protein kinase type I-alpha regulatory subunit	0.79	NA	NA	2	5.5
P00390	Glutathione reductase, mitochondrial	0.78	NA	NA	2	5.7
P39656	Dolichyl-diphosphooligosaccharide--protein glycosyltransferase 48 kDa subunit	0.78	0.067	2.0E-02	5	10.7
Q13596	Sorting nexin-1	0.78	0.180	2.9E-01	3	7.1
P26196	Probable ATP-dependent RNA helicase DDX6	0.77	0.090	4.2E-02	3	9.3
P20674	Cytochrome c oxidase subunit 5A, mitochondrial	0.77	0.227	3.8E-01	5	25.3
P42566	Epidermal growth factor receptor substrate 15	0.76	NA	NA	2	3.2
Q99714	3-hydroxyacyl-CoA dehydrogenase type-2	0.76	NA	NA	2	7.7

Q8IYB3	Serine/arginine repetitive matrix protein 1	0.76	NA	NA	2	2.4
O60256	Phosphoribosyl pyrophosphate synthase-associated protein 2	0.76	NA	NA	2	8.1
P36957	Dihydrolipoyllysine-residue succinyltransferase component of 2-oxoglutarate dehydrogenase complex, mitochondrial	0.76	0.059	4.4E-04	6	14.3
Q8IUX7	Adipocyte enhancer-binding protein 1	0.75	NA	NA	2	2.8
O43837	Isocitrate dehydrogenase [NAD] subunit beta, mitochondrial	0.75	NA	NA	2	5.5
P17643	5,6-dihydroxyindole-2-carboxylic acid oxidase	0.74	0.121	9.5E-02	5	11.5
P61769	Beta-2-microglobulin	0.74	NA	NA	2	16.8
Q9BQE3	Tubulin alpha-1C chain	0.73	0.159	2.8E-02	4	12.0
Q9NY61	Protein AATF	0.73	NA	NA	2	5.0
P51665	26S proteasome non-ATPase regulatory subunit 7	0.73	NA	NA	2	6.2
O00487	26S proteasome non-ATPase regulatory subunit 14	0.72	NA	NA	2	6.5
P18859	ATP synthase-coupling factor 6, mitochondrial	0.72	NA	NA	2	13.0
Q00577	Transcriptional activator protein Pur-alpha	0.72	NA	NA	2	5.0
Q5JWF2	Guanine nucleotide-binding protein G(s) subunit alpha isoforms XLas	0.72	0.241	1.3E-01	3	4.0
Q9Y277	Voltage-dependent anion-selective channel protein 3	0.72	NA	NA	2	6.7
Q14789	Golgin subfamily B member 1	0.72	NA	NA	2	0.9
P05107	Integrin beta-2	0.72	NA	NA	2	2.9
Q16795	NADH dehydrogenase [ubiquinone] 1 alpha subcomplex subunit 9, mitochondrial	0.72	NA	NA	2	6.4
Q9P2R7	Succinyl-CoA ligase [ADP-forming] subunit beta, mitochondrial	0.71	NA	NA	2	4.3
Q9UHG3	Prenylcysteine oxidase 1	0.71	0.134	4.9E-02	6	15.4
P11310	Medium-chain specific acyl-CoA dehydrogenase, mitochondrial	0.71	NA	NA	2	5.5
O60313	Dynamin-like 120 kDa protein, mitochondrial	0.71	0.084	3.2E-02	6	7.9
P54727	UV excision repair protein RAD23 homolog B	0.71	0.057	4.0E-02	3	7.3
Q9Y3Z3	Deoxynucleoside triphosphate triphosphohydrolase SAMHD1	0.70	NA	NA	2	5.1
Q9Y3I0	tRNA-splicing ligase RtcB homolog	0.70	0.471	1.8E-01	6	15.8
Q00610	Clathrin heavy chain 1	0.70	0.051	6.1E-07	27	16.2
P62805	Histone H4	0.69	0.048	9.5E-07	7	52.4
P35606	Coatomer subunit beta'	0.69	NA	NA	2	3.5
Q9UHQ9	NADH-cytochrome b5 reductase 1	0.69	0.096	4.9E-03	5	18.7
P04217	Alpha-1B-glycoprotein	0.69	0.520	3.9E-01	3	6.7
Q6DD88	Atlastin-3	0.69	NA	NA	2	4.3
P35573	Glycogen debranching enzyme	0.69	NA	NA	2	2.1
P09382	Galectin-1	0.68	0.062	1.3E-04	6	46.7
P05787	Keratin, type II cytoskeletal 8	0.68	0.054	6.0E-03	3	5.4
P61163	Alpha-centractin	0.68	NA	NA	2	4.5
Q14764	Major vault protein	0.68	0.100	5.4E-03	7	8.7
P55084	Trifunctional enzyme subunit beta, mitochondrial	0.68	0.061	6.9E-05	10	20.5
P04196	Histidine-rich glycoprotein	0.67	0.160	3.7E-02	4	7.4
Q07157	Tight junction protein ZO-1	0.67	NA	NA	2	1.3
P35221	Catenin alpha-1	0.67	0.261	2.5E-01	3	3.4
O14950	Myosin regulatory light chain 12B	0.65	NA	NA	2	12.2
Q16695	Histone H3.1t	0.64	0.072	2.2E-04	3	14.7
P16402	Histone H1.3	0.64	0.155	9.3E-03	6	15.4
P57105	Synaptojanin-2-binding protein	0.64	NA	NA	2	16.6
P61026	Ras-related protein Rab-10	0.64	0.127	3.1E-01	3	15.0
P60709	Actin, cytoplasmic 1	0.64	0.079	7.3E-05	5	16.8
Q9UIJ7	GTP:AMP phosphotransferase AK3, mitochondrial	0.64	NA	NA	2	11.0
Q9Y305	Acyl-coenzyme A thioesterase 9, mitochondrial	0.64	NA	NA	2	5.5
P01860	Ig gamma-3 chain C region	0.64	0.062	1.0E-03	3	6.4
P33176	Kinesin-1 heavy chain	0.63	NA	NA	2	2.8
Q9UBS4	DnaJ homolog subfamily B member 11	0.63	NA	NA	2	6.4
P08133	Annexin A6	0.63	0.048	1.4E-10	25	40.6
O14745	Na(+)/H(+) exchange regulatory cofactor NHE-RF1	0.63	NA	NA	2	5.6
Q969X5	Endoplasmic reticulum-Golgi intermediate compartment protein 1	0.62	0.152	6.2E-02	3	11.0
P00367	Glutamate dehydrogenase 1, mitochondrial	0.62	0.059	1.6E-04	11	22.8
P61626	Lysozyme C	0.62	NA	NA	2	12.8
P43121	Cell surface glycoprotein MUC18	0.62	0.302	1.6E-01	4	7.4
P53992	Protein transport protein Sec24C	0.62	NA	NA	2	2.6
P28289	Tropomodulin-1	0.61	NA	NA	2	5.3
Q9H4M9	EH domain-containing protein 1	0.61	0.090	3.8E-02	5	7.5
O43707	Alpha-actinin-4	0.60	0.050	2.5E-08	16	21.0
P09543	2',3'-cyclic-nucleotide 3'-phosphodiesterase	0.60	0.107	7.2E-03	4	9.5
O15173	Membrane-associated progesterone receptor component 2	0.60	NA	NA	2	9.9
P35222	Catenin beta-1	0.60	0.067	1.1E-02	4	5.8
Q9BS26	Endoplasmic reticulum resident protein 44	0.59	0.032	1.1E-03	4	8.4
Q9NZ08	Endoplasmic reticulum aminopeptidase 1	0.59	NA	NA	2	2.2
P55072	Transitional endoplasmic reticulum ATPase	0.59	0.043	1.1E-10	11	14.6
P06899	Histone H2B type 1-J	0.59	NA	NA	2	7.9
Q15435	Protein phosphatase 1 regulatory subunit 7	0.59	NA	NA	2	5.6
P54709	Sodium/potassium-transporting ATPase subunit beta-3	0.59	0.170	2.2E-01	5	22.6
P07099	Epoxide hydrolase 1	0.58	0.080	1.2E-04	9	14.5
P53621	Coatomer subunit alpha	0.58	NA	NA	2	1.4
P50995	Annexin A11	0.57	0.064	7.6E-04	4	7.7
Q14344	Guanine nucleotide-binding protein subunit alpha-13	0.57	0.349	1.1E-01	5	14.3
P47756	F-actin-capping protein subunit beta	0.57	0.169	1.2E-01	4	16.2
P68371	Tubulin beta-4B chain	0.57	0.109	5.6E-03	3	10.6
Q9Y639	Neuroplastin	0.57	0.178	5.5E-02	3	9.3
Q15436	Protein transport protein Sec23A	0.56	0.342	3.2E-01	3	5.6
Q6NUK1	Calcium-binding mitochondrial carrier protein SCaMC-1	0.56	NA	NA	2	3.4
Q8WUM4	Programmed cell death 6-interacting protein	0.56	0.017	3.8E-04	3	2.9
P05109	Protein S100-A8	0.55	NA	NA	2	19.4
P17655	Calpain-2 catalytic subunit	0.55	0.039	1.2E-02	3	4.3
Q9P0V9	Septin-10	0.55	NA	NA	2	3.7
P20073	Annexin A7	0.55	NA	NA	2	4.1
P11166	Solute carrier family 2, facilitated glucose transporter member 1	0.55	0.023	2.2E-07	3	5.5
Q6UVK1	Chondroitin sulfate proteoglycan 4	0.54	0.382	5.0E-01	3	2.3
Q9NQC3	Reticulon-4	0.54	NA	NA	2	2.3
Q15019	Septin-2	0.54	0.060	7.1E-03	4	13.9
Q16181	Septin-7	0.54	0.144	7.4E-03	8	23.1
P11413	Glucose-6-phosphate 1-dehydrogenase	0.54	NA	NA	2	3.7
P01023	Alpha-2-macroglobulin	0.53	0.135	1.0E-01	6	5.2
P02649	Apolipoprotein E	0.53	0.076	1.2E-06	14	45.4
Q9UKG1	DCC-interacting protein 13-alpha	0.52	NA	NA	2	4.7
O95782	AP-2 complex subunit alpha-1	0.52	0.234	1.3E-01	3	2.9
Q02218	2-oxoglutarate dehydrogenase, mitochondrial	0.52	0.053	5.6E-06	7	9.1
P16615	Sarcoplasmic/endoplasmic reticulum calcium ATPase 2	0.52	0.061	2.3E-05	8	8.3
P10515	Dihydrolipoyllysine-residue acetyltransferase component of pyruvate dehydrogenase complex, mitochondrial	0.52	NA	NA	2	2.6
P01903	HLA class II histocompatibility antigen, DR alpha chain	0.52	NA	NA	2	9.8
Q9Y4G6	Talin-2	0.52	NA	NA	2	1.3
P24752	Acetyl-CoA acetyltransferase, mitochondrial	0.52	0.204	8.6E-02	5	13.1
Q9Y4L1	Hypoxia up-regulated protein 1	0.51	NA	NA	2	2.4
P12814	Alpha-actinin-1	0.51	0.046	1.4E-07	14	18.9
O43175	D-3-phosphoglycerate dehydrogenase	0.50	NA	NA	2	5.6
Q14847	LIM and SH3 domain protein 1	0.50	0.287	2.8E-01	3	12.6
Q01813	ATP-dependent 6-phosphofructokinase, platelet type	0.50	NA	NA	2	3.7
Q14203	Dynactin subunit 1	0.50	0.046	1.6E-02	3	3.3
O75369	Filamin-B	0.49	0.085	9.1E-06	11	6.3
Q9BTV4	Transmembrane protein 43	0.49	NA	NA	2	6.3
P60660	Myosin light polypeptide 6	0.49	0.050	1.5E-13	6	47.7
P09497	Clathrin light chain B	0.49	NA	NA	2	7.9
P54920	Alpha-soluble NSF attachment protein	0.48	0.408	3.2E-01	3	11.2
P04179	Superoxide dismutase [Mn], mitochondrial	0.48	0.109	7.5E-03	5	23.0
Q6PIU2	Neutral cholesterol ester hydrolase 1	0.48	NA	NA	2	5.4
P32119	Peroxiredoxin-2	0.48	0.077	3.6E-04	4	18.2
P18206	Vinculin	0.47	0.074	8.8E-06	15	15.9

Q9NVA2	Septin-11	0.47	0.109	4.9E-02	4	10.3
Q15149	Plectin	0.46	0.033	0.0E+00	52	11.5
P06727	Apolipoprotein A-IV	0.46	0.079	2.4E-04	8	20.7
P01024	Complement C3	0.46	0.199	1.5E-03	21	14.7
P60033	CD81 antigen	0.45	NA	NA	2	11.9
Q9UPT5	Exocyst complex component 7	0.45	NA	NA	2	3.0
Q15836	Vesicle-associated membrane protein 3	0.45	NA	NA	2	24.0
Q12797	Aspartyl/asparaginyl beta-hydroxylase	0.45	0.173	8.8E-03	5	4.7
Q13409	Cytoplasmic dynein 1 intermediate chain 2	0.44	NA	NA	2	3.9
Q96CW1	AP-2 complex subunit mu	0.44	0.144	1.8E-02	3	6.4
Q14204	Cytoplasmic dynein 1 heavy chain 1	0.44	0.032	3.5E-14	20	4.6
Q9Y6N5	Sulfide:quinone oxidoreductase, mitochondrial	0.44	0.252	3.4E-02	5	12.7
Q9BZF9	Uveal autoantigen with coiled-coil domains and ankyrin repeats	0.44	0.206	3.7E-01	3	2.8
P04899	Guanine nucleotide-binding protein G(i) subunit alpha-2	0.43	NA	NA	2	6.8
P35579	Myosin-9	0.43	0.031	0.0E+00	59	29.6
Q9Y240	C-type lectin domain family 11 member A	0.43	NA	NA	2	7.7
A0FGR8	Extended synaptotagmin-2	0.42	NA	NA	2	3.0
P0C0L5	Complement C4-B	0.42	0.250	7.5E-02	3	1.0
P49748	Very long-chain specific acyl-CoA dehydrogenase, mitochondrial	0.42	0.168	1.3E-02	4	6.7
Q14254	Flotillin-2	0.42	0.101	4.6E-03	3	7.5
Q96FJ2	Dynein light chain 2, cytoplasmic	0.42	NA	NA	2	20.2
Q9UM54	Unconventional myosin-VI	0.41	NA	NA	2	1.5
O43865	Putative adenosylhomocysteinase 2	0.41	NA	NA	2	3.6
P26447	Protein S100-A4	0.41	0.164	4.1E-03	3	27.7
Q13425	Beta-2-syntrophin	0.41	NA	NA	2	3.1
Q9BZQ8	Protein Niban	0.41	NA	NA	2	2.4
O94919	Endonuclease domain-containing 1 protein	0.40	NA	NA	2	5.8
P05362	Intercellular adhesion molecule 1	0.40	0.179	2.2E-01	3	7.9
Q13557	Calcium/calmodulin-dependent protein kinase type II subunit delta	0.39	NA	NA	2	4.2
P01876	Ig alpha-1 chain C region	0.39	NA	NA	2	4.5
P59768	Guanine nucleotide-binding protein G(I)/G(S)/G(O) subunit gamma-2	0.38	NA	NA	2	42.3
Q03591	Complement factor H-related protein 1	0.38	NA	NA	2	9.4
P12235	ADP/ATP translocase 1	0.36	NA	NA	2	7.4
P00352	Retinal dehydrogenase 1	0.35	NA	NA	2	4.8
Q63ZY3	KN motif and ankyrin repeat domain-containing protein 2	0.34	NA	NA	2	4.2
P02654	Apolipoprotein C-I	0.33	NA	NA	2	24.1
Q96AC1	Fermitin family homolog 2	0.33	NA	NA	2	3.1
Q6NY19	KN motif and ankyrin repeat domain-containing protein 3	0.31	NA	NA	2	3.3
P07305	Histone H1.0	0.30	NA	NA	2	9.3
P46939	Utrophin	0.30	0.179	1.3E-01	3	0.9
P62873	Guanine nucleotide-binding protein G(I)/G(S)/G(T) subunit beta-1	0.29	0.302	3.8E-01	3	12.9
P13671	Complement component C6	0.29	NA	NA	2	2.7
O94832	Unconventional myosin-Id	0.29	NA	NA	2	2.4
P04839	Cytochrome b-245 heavy chain	0.28	NA	NA	2	3.0
P13987	CD59 glycoprotein	0.28	NA	NA	2	15.6
P10745	Retinol-binding protein 3	0.27	0.696	1.6E-01	3	2.3
P06756	Integrin alpha-V	0.27	NA	NA	2	1.4
P05164	Myeloperoxidase	0.27	0.209	5.6E-02	3	4.7
Q92556	Engulfment and cell motility protein 1	0.24	NA	NA	2	3.2
O43301	Heat shock 70 kDa protein 12A	0.23	0.272	1.1E-01	3	5.2
Q03135	Caveolin-1	0.22	NA	NA	2	13.5
Q12805	EGF-containing fibulin-like extracellular matrix protein 1	0.22	NA	NA	2	3.9
P07942	Laminin subunit beta-1	0.22	0.173	9.8E-02	3	2.1
Q96CX2	BTB/POZ domain-containing protein KCTD12	0.22	0.183	1.7E-01	3	9.8
Q14767	Latent-transforming growth factor beta-binding protein 2	0.21	NA	NA	2	1.7
Q9HBL0	Tensin-1	0.21	0.565	7.1E-01	3	3.1
P23229	Integrin alpha-6	0.20	NA	NA	2	2.3
Q13642	Four and a half LIM domains protein 1	0.18	NA	NA	2	5.6
Q9BS40	Latexin	0.17	NA	NA	2	12.2
P05186	Alkaline phosphatase, tissue-nonspecific isozyme	0.15	NA	NA	2	3.8
O94875	Sorbin and SH3 domain-containing protein 2	0.14	NA	NA	2	3.7
Q14699	Raftlin	0.14	NA	NA	2	3.8
P22105	Tenascin-X	0.13	NA	NA	2	0.4
P35243	Recoverin	0.13	NA	NA	2	13.5
Q969G5	Protein kinase C delta-binding protein	0.11	NA	NA	2	7.7
Q9BXN1	Asporin	0.11	NA	NA	2	2.9
P24844	Myosin regulatory light polypeptide 9	0.10	NA	NA	2	12.2
P02686	Myelin basic protein	0.09	NA	NA	2	7.2
P07196	Neurofilament light polypeptide	0.07	NA	NA	2	3.5
P08123	Collagen alpha-2(I) chain	0.05	NA	NA	2	2.4
P23946	Chymase	0.04	0.371	5.1E-02	5	25.5
P02751	Fibronectin	0.41	0.049	1.7E-10	15	7.7
Q03252	Lamin-B2	0.41	0.051	4.4E-12	17	27.0
P00387	NADH-cytochrome b5 reductase 3	0.40	0.090	5.1E-05	6	26.6
P51648	Fatty aldehyde dehydrogenase	0.39	0.028	3.5E-02	3	6.2
P06396	Gelsolin	0.39	0.064	1.2E-09	13	18.7
Q9BSJ8	Extended synaptotagmin-1	0.39	0.182	1.4E-02	4	4.7
Q09666	Neuroblast differentiation-associated protein AHNAK	0.38	0.032	0.0E+00	63	9.4
Q07954	Prolow-density lipoprotein receptor-related protein 1	0.37	0.063	6.0E-08	11	3.2
O94905	Erlin-2	0.37	0.120	1.4E-04	5	12.4
P07358	Complement component C8 beta chain	0.37	0.231	2.9E-02	4	7.8
P05023	Sodium/potassium-transporting ATPase subunit alpha-1	0.36	0.061	8.5E-10	10	10.9
P02749	Beta-2-glycoprotein 1	0.36	0.202	1.9E-03	3	9.6
O00159	Unconventional myosin-Ic	0.35	0.204	7.7E-03	7	7.0
Q99584	Protein S100-A13	0.34	0.126	5.1E-04	3	33.7
P01011	Alpha-1-antichymotrypsin	0.34	0.091	6.4E-08	8	17.7
P04216	Thy-1 membrane glycoprotein	0.34	0.066	9.1E-05	3	16.1
P04004	Vitronectin	0.33	0.090	4.6E-08	10	20.9
P02675	Fibrinogen beta chain	0.33	0.074	7.8E-11	9	23.6
P05556	Integrin beta-1	0.33	0.080	1.9E-04	6	8.3
P12111	Collagen alpha-3(VI) chain	0.33	0.049	0.0E+00	39	13.3
P07355	Annexin A2	0.33	0.038	0.0E+00	22	54.0
P00167	Cytochrome b5	0.32	0.078	2.9E-03	4	42.5
P02679	Fibrinogen gamma chain	0.31	0.131	1.0E-04	10	22.7
P12110	Collagen alpha-2(VI) chain	0.31	0.168	8.9E-05	7	7.5
Q9NZM1	Myoferlin	0.30	0.210	1.6E-03	6	3.5
P12109	Collagen alpha-1(VI) chain	0.30	0.064	1.1E-07	9	9.5
P43320	Beta-crystallin B2	0.28	0.271	3.8E-02	3	15.6
P00450	Ceruloplasmin	0.28	0.063	6.1E-08	10	11.4
P09493	Tropomyosin alpha-1 chain	0.27	0.125	2.3E-05	4	10.2
P27105	Erythrocyte band 7 integral membrane protein	0.27	0.066	6.4E-05	4	14.2
P00738	Haptoglobin	0.27	0.038	4.4E-03	3	6.4
Q9Y490	Talin-1	0.26	0.076	4.6E-10	20	10.2
P21333	Filamin-A	0.26	0.042	0.0E+00	43	21.8
Q01082	Spectrin beta chain, non-erythrocytic 1	0.26	0.034	0.0E+00	44	22.4
P02671	Fibrinogen alpha chain	0.26	0.080	1.3E-08	11	14.9
Q9Y6C2	EMILIN-1	0.26	0.150	2.7E-04	7	9.2
Q13813	Spectrin alpha chain, non-erythrocytic 1	0.26	0.031	0.0E+00	69	32.1
P08294	Extracellular superoxide dismutase [Cu-Zn]	0.24	0.125	1.1E-03	3	15.4
Q16363	Laminin subunit alpha-4	0.24	0.208	1.7E-03	6	3.6
P68032	Actin, alpha cardiac muscle 1	0.23	0.191	2.1E-06	6	21.0
P02549	Spectrin alpha chain, erythrocytic 1	0.23	0.189	1.3E-04	10	4.9
P98160	Basement membrane-specific heparan sulfate proteoglycan core protein	0.22	0.059	0.0E+00	33	9.3
P08572	Collagen alpha-2(IV) chain	0.22	0.170	3.9E-06	6	3.9
P10909	Clusterin	0.22	0.048	0.0E+00	17	34.5

Q16555	Dihydropyrimidinase-related protein 2	0.21	0.112	9.5E-07	7	15.9
P02511	Alpha-crystallin B chain	0.20	0.220	5.2E-04	3	17.1
P55268	Laminin subunit beta-2	0.20	0.090	5.1E-10	14	8.7
P50895	Basal cell adhesion molecule	0.20	0.250	8.3E-03	4	9.2
P09936	Ubiquitin carboxyl-terminal hydrolase isozyme L1	0.19	0.141	1.0E-04	4	21.5
P04083	Annexin A1	0.17	0.058	1.6E-12	14	44.5
O43491	Band 4.1-like protein 2	0.17	0.046	3.2E-05	7	10.0
P39060	Collagen alpha-1(XVIII) chain	0.17	0.085	3.5E-10	5	3.1
P80723	Brain acid soluble protein 1	0.17	0.093	1.8E-07	6	38.3
P11277	Spectrin beta chain, erythrocytic	0.17	0.135	5.7E-03	9	5.1
P60903	Protein S100-A10	0.17	0.071	4.1E-11	4	35.1
P01008	Antithrombin-III	0.16	0.147	7.7E-04	4	9.9
Q6NZI2	Polymerase I and transcript release factor	0.16	0.131	5.4E-05	6	19.2
Q05707	Collagen alpha-1(XIV) chain	0.16	0.147	4.1E-07	9	5.1
Q01995	Transgelin	0.16	0.160	4.1E-03	4	21.9
P16157	Ankyrin-1	0.15	0.138	3.9E-04	3	2.1
Q9BXM0	Periaxin	0.15	0.254	4.1E-03	4	1.4
P46821	Microtubule-associated protein 1B	0.15	0.126	4.6E-06	6	3.1
O15230	Laminin subunit alpha-5	0.14	0.114	4.2E-10	10	3.3
Q14112	Nidogen-2	0.14	0.149	2.0E-06	9	7.7
P01031	Complement C5	0.14	0.155	1.8E-04	7	4.3
P02462	Collagen alpha-1(IV) chain	0.13	0.161	2.7E-03	4	3.3
P01871	Ig mu chain C region	0.13	0.194	1.5E-04	6	15.9
P14543	Nidogen-1	0.12	0.147	3.6E-06	7	7.2
P11047	Laminin subunit gamma-1	0.12	0.131	5.6E-06	11	6.3
P07197	Neurofilament medium polypeptide	0.12	0.272	1.8E-02	4	6.8
P02743	Serum amyloid P-component	0.12	0.135	1.7E-08	6	23.3
P02760	Protein AMBP	0.12	0.175	1.3E-03	3	12.5
P04275	von Willebrand factor	0.12	0.136	1.1E-09	11	4.3
P22352	Glutathione peroxidase 3	0.12	0.092	1.6E-07	3	11.5
P21980	Protein-glutamine gamma-glutamyltransferase 2	0.11	0.097	2.7E-11	13	18.5
P02730	Band 3 anion transport protein	0.11	0.131	1.5E-07	9	12.1
Q14195	Dihydropyrimidinase-related protein 3	0.11	0.111	5.7E-09	9	21.6
P58166	Inhibin beta E chain	0.11	0.395	3.7E-02	3	10.0
P07585	Decorin	0.11	0.134	2.5E-05	7	21.7
P02748	Complement component C9	0.11	0.096	4.1E-08	9	14.5
P51888	Prolargin	0.11	0.063	0.0E+00	10	29.3
P35749	Myosin-11	0.11	0.111	1.2E-12	34	20.2
P20774	Mimecan	0.10	0.115	6.0E-11	8	24.8
P36269	Gamma-glutamyltransferase 5	0.10	0.203	4.0E-05	6	12.3
P35625	Metalloproteinase inhibitor 3	0.10	0.163	1.3E-06	4	19.4
P35555	Fibrillin-1	0.10	0.057	0.0E+00	42	16.5
Q02952	A-kinase anchor protein 12	0.10	0.161	8.4E-05	9	5.2
P22748	Carbonic anhydrase 4	0.10	0.192	9.7E-07	8	23.1
P15088	Mast cell carboxypeptidase A	0.08	0.156	2.2E-06	6	12.5
P21810	Biglycan	0.08	0.100	1.8E-13	8	24.5
P21926	CD9 antigen	0.08	0.133	1.7E-03	3	9.6
P41219	Peripherin	0.08	0.123	2.5E-08	13	26.6
P39059	Collagen alpha-1(XV) chain	0.07	0.283	3.0E-03	4	2.9
P51884	Lumican	0.07	0.063	0.0E+00	10	29.3
Q15661	Tryptase alpha/beta-1	0.06	0.139	9.1E-09	5	21.8
P25189	Myelin protein P0	0.04	0.131	1.2E-10	7	27.4

Brown denotes change ≥ 2 standard deviations (SD) from the mean, yellow denotes change ≥ 1 SD and green highlights p values ≤ 0.05. NA, not applicable, n<3 unique peptides.
