# Supplementary material for: iTRAQ Quantitative Proteomic Comparison of Metastatic and Non-Metastatic Uveal Melanoma Tumors
Source: PLoS One. 2015 Aug 25;10(8):e0135543. doi: 10.1371/journal.pone.0135543 (PMC4549237; doi:10.1371/journal.pone.0135543)
Supplement: S16 Table — (PDF) [file pone.0135543.s016.pdf]

Supplementary Table S16

Average Relative Protein Abundance: Metastatatic Tumors (Samples UM19, UM21, UM24, UM28, UM30)

Total Proteins Quantified = 1405, LogMedian Protein Ratio = 0.11; LogMean Protein Ratio = 0; Standard Deviation = 0.77

Uni-Prot Accession	Protein	Sample Frequency n = 5 total	Ratio UM/Control	SEM	*p* value
P62937	Peptidyl-prolyl cis-trans isomerase A	5	6.11	0.042	1.8E-06
P05413	Fatty acid-binding protein, heart	3	4.91	0.193	1.4E-02
P17096	High mobility group protein HMG-I/HMG-Y	4	4.70	0.349	2.1E-02
P23381	Tryptophan--tRNA ligase, cytoplasmic	5	4.42	0.151	6.0E-04
P07108	Acyl-CoA-binding protein	3	4.23	0.041	8.2E-04
P78417	Glutathione S-transferase omega-1	5	4.16	0.060	1.9E-05
P06454	Prothymosin alpha	4	4.15	0.060	1.6E-04
P02768	Serum albumin	5	4.04	0.235	4.0E-03
O75368	SH3 domain-binding glutamic acid-rich-like protein	4	4.04	0.144	2.3E-03
O14556	Glyceraldehyde-3-phosphate dehydrogenase, testis-specific	4	3.99	0.240	1.0E-02
P31939	Bifunctional purine biosynthesis protein PURH	4	3.98	0.241	1.1E-02
P30043	Flavin reductase (NADPH)	4	3.78	0.080	4.8E-04
P52566	Rho GDP-dissociation inhibitor 2	4	3.74	0.291	2.0E-02
P06865	Beta-hexosaminidase subunit alpha	4	3.71	0.143	2.7E-03
Q8IV08	Phospholipase D3	4	3.64	0.122	1.8E-03
P63241	Eukaryotic translation initiation factor 5A-1	5	3.58	0.122	4.6E-04
P00558	Phosphoglycerate kinase 1	5	3.46	0.043	8.5E-06
Q00796	Sorbitol dehydrogenase	5	3.33	0.137	9.4E-04
P31146	Coronin-1A	4	3.31	0.252	1.8E-02
Q9UBR2	Cathepsin Z	3	3.28	0.067	3.1E-03
P40925	Malate dehydrogenase, cytoplasmic	4	3.20	0.118	2.2E-03
Q9Y2S2	Lambda-crystallin homolog	4	3.19	0.226	1.4E-02
P23528	Cofilin-1	5	3.11	0.051	2.4E-05
P06733	Alpha-enolase	5	3.09	0.135	1.1E-03
P07737	Profilin-1	5	3.07	0.162	2.3E-03
P12955	Xaa-Pro dipeptidase	4	3.06	0.152	5.2E-03
P30086	Phosphatidylethanolamine-binding protein 1	5	3.06	0.288	1.8E-02
P60174	Triosephosphate isomerase	5	3.05	0.108	5.0E-04
P61088	Ubiquitin-conjugating enzyme E2 N	3	3.02	0.067	3.7E-03
P07686	Beta-hexosaminidase subunit beta	4	3.02	0.147	4.9E-03
Q16658	Fascin	4	3.00	0.080	8.3E-04
O75347	Tubulin-specific chaperone A	3	2.95	0.149	1.9E-02
Q96C86	m7GpppX diphosphatase	4	2.93	0.133	3.9E-03
P02787	Serotransferrin	5	2.91	0.207	6.6E-03
P40121	Macrophage-capping protein	3	2.89	0.110	1.1E-02
P16401	Histone H1.5	5	2.88	0.250	1.3E-02
Q99497	Protein DJ-1	5	2.88	0.190	5.1E-03
O15400	Syntaxin-7	4	2.88	0.215	1.6E-02
Q01105	Protein SET	3	2.79	0.144	1.9E-02
O95336	6-phosphogluconolactonase	4	2.79	0.093	1.6E-03
P10599	Thioredoxin	5	2.79	0.251	1.5E-02
P23526	Adenosylhomocysteinase	5	2.78	0.069	1.2E-04
P29401	Transketolase	5	2.76	0.108	7.1E-04
P52565	Rho GDP-dissociation inhibitor 1	5	2.76	0.124	1.2E-03
P04406	Glyceraldehyde-3-phosphate dehydrogenase	5	2.74	0.052	4.3E-05
P57729	Ras-related protein Rab-38	4	2.73	0.138	5.4E-03
P15531	Nucleoside diphosphate kinase A	5	2.73	0.158	3.2E-03
P08758	Annexin A5	5	2.72	0.043	2.0E-05
P13693	Translationally-controlled tumor protein	3	2.70	0.128	1.6E-02
P19338	Nucleolin	5	2.70	0.151	2.8E-03
Q9Y2X3	Nucleolar protein 58	5	2.68	0.082	2.8E-04
P22087	rRNA 2'-O-methyltransferase fibrillarin	5	2.67	0.100	5.9E-04
P50395	Rab GDP dissociation inhibitor beta	4	2.66	0.154	7.9E-03
Q9BRA2	Thioredoxin domain-containing protein 17	3	2.64	0.134	1.9E-02
P11766	Alcohol dehydrogenase class-3	5	2.64	0.129	1.7E-03
P13796	Plastin-2	4	2.62	0.177	1.2E-02
P06748	Nucleophosmin	5	2.60	0.128	1.7E-03
P16070	CD44 antigen	5	2.58	0.141	2.5E-03
P04080	Cystatin-B	5	2.57	0.107	9.1E-04
P20042	Eukaryotic translation initiation factor 2 subunit 2	4	2.56	0.103	2.8E-03
P07195	L-lactate dehydrogenase B chain	5	2.55	0.223	1.4E-02
P51858	Hepatoma-derived growth factor	3	2.55	0.130	1.9E-02
Q53EL6	Programmed cell death protein 4	3	2.50	0.076	6.8E-03
P07741	Adenine phosphoribosyltransferase	4	2.49	0.100	2.7E-03
P58546	Myotrophin	3	2.46	0.085	8.7E-03
P16152	Carbonyl reductase [NADPH] 1	5	2.45	0.139	2.9E-03
Q04760	Lactoylglutathione lyase	4	2.45	0.091	2.3E-03
P22234	Multifunctional protein ADE2	4	2.44	0.073	1.2E-03
P17900	Ganglioside GM2 activator	3	2.38	0.129	2.1E-02
P63104	14-3-3 protein zeta/delta	5	2.37	0.116	1.8E-03
P06744	Glucose-6-phosphate isomerase	5	2.37	0.187	9.9E-03
P40967	Melanocyte protein PMEL	3	2.36	0.154	3.1E-02
P33121	Long-chain-fatty-acid--CoA ligase 1	4	2.35	0.145	9.7E-03
Q9H3G5	Probable serine carboxypeptidase CPVL	3	2.35	0.138	2.5E-02
P31948	Stress-induced-phosphoprotein 1	5	2.33	0.080	4.5E-04
P07858	Cathepsin B	5	2.31	0.204	1.5E-02
P60842	Eukaryotic initiation factor 4A-I	5	2.30	0.078	4.3E-04
O14818	Proteasome subunit alpha type-7	4	2.30	0.151	1.2E-02
P07900	Heat shock protein HSP 90-alpha	5	2.29	0.058	1.4E-04
Q13838	Spliceosome RNA helicase DDX39B	5	2.27	0.114	1.9E-03
Q13185	Chromobox protein homolog 3	5	2.26	0.179	1.0E-02
Q13510	Acid ceramidase	5	2.25	0.220	2.1E-02
P15121	Aldose reductase	4	2.20	0.098	4.0E-03
P07339	Cathepsin D	5	2.18	0.091	1.0E-03
P14618	Pyruvate kinase PKM	5	2.16	0.110	2.2E-03
P14174	Macrophage migration inhibitory factor	1	8.66	NA	NA
Q9BQE5	Apolipoprotein L2	1	7.90	NA	NA
P27169	Serum paraoxonase/arylesterase 1	1	7.23	NA	NA
Q9UL41	Paraneoplastic antigen Ma3	1	6.85	NA	NA
Q9UI08	Ena/VASP-like protein	1	5.93	NA	NA
P06702	Protein S100-A9	1	5.75	NA	NA
P13686	Tartrate-resistant acid phosphatase type 5	1	5.06	NA	NA
Q5SNT6	WASH complex subunit FAM21B	1	4.95	NA	NA
Q03519	Antigen peptide transporter 2	1	4.86	NA	NA
P32455	Interferon-induced guanylate-binding protein 1	1	4.79	NA	NA
P18463	HLA class I histocompatibility antigen, B-37 alpha chain	1	4.78	NA	NA
P55769	NHP2-like protein 1	2	4.76	NA	NA
P49863	Granzyme K	1	4.36	NA	NA
P27695	DNA-(apurinic or apyrimidinic site) lyase	2	4.29	NA	NA
P30273	High affinity immunoglobulin epsilon receptor subunit gamma	1	4.18	NA	NA
O00625	Pirin	2	4.14	NA	NA
P05109	Protein S100-A8	1	4.05	NA	NA
P59665	Neutrophil defensin 1	1	4.03	NA	NA
P43652	Afamin	1	4.01	NA	NA
Q99848	Probable rRNA-processing protein EBP2	1	3.96	NA	NA
P09601	Heme oxygenase 1	1	3.78	NA	NA
Q96G03	Phosphoglucomutase-2	1	3.66	NA	NA
Q13637	Ras-related protein Rab-32	1	3.64	NA	NA
P17213	Bactericidal permeability-increasing protein	1	3.63	NA	NA
Q9NRV9	Heme-binding protein 1	2	3.62	NA	NA
Q9H1E3	Nuclear ubiquitous casein and cyclin-dependent kinase substrate 1	2	3.58	NA	NA
P24158	Myeloblastin	1	3.40	NA	NA
Q15819	Ubiquitin-conjugating enzyme E2 variant 2	2	3.39	NA	NA
Q08170	Serine/arginine-rich splicing factor 4	1	3.24	NA	NA
P30085	UMP-CMP kinase	2	3.23	NA	NA
P15586	N-acetylglucosamine-6-sulfatase	2	3.23	NA	NA
P00915	Carbonic anhydrase 1	1	3.19	NA	NA
P04439	HLA class I histocompatibility antigen, A-3 alpha chain	1	3.18	NA	NA
P05534	HLA class I histocompatibility antigen, A-24 alpha chain	1	3.17	NA	NA
P62310	U6 snRNA-associated Sm-like protein LSm3	1	3.17	NA	NA
Q03518	Antigen peptide transporter 1	1	3.16	NA	NA
P41218	Myeloid cell nuclear differentiation antigen	1	3.06	NA	NA
Q8WWM9	Cytoglobin	2	3.03	NA	NA
Q9BXK5	Bcl-2-like protein 13	1	2.99	NA	NA
Q9BVJ6	U3 small nucleolar RNA-associated protein 14 homolog A	1	2.94	NA	NA
P16930	Fumarylacetoacetase	2	2.93	NA	NA
O15347	High mobility group protein B3	1	2.90	NA	NA
O15533	Tapasin	2	2.89	NA	NA
P67809	Nuclease-sensitive element-binding protein 1	1	2.85	NA	NA
P09960	Leukotriene A-4 hydrolase	1	2.83	NA	NA
O00115	Deoxyribonuclease-2-alpha	1	2.79	NA	NA
Q13428	Treacle protein	2	2.77	NA	NA
Q9UQ80	Proliferation-associated protein 2G4	1	2.72	NA	NA
P10253	Lysosomal alpha-glucosidase	1	2.72	NA	NA
Q9UHL4	Dipeptidyl peptidase 2	2	2.67	NA	NA
O75348	V-type proton ATPase subunit G 1	1	2.62	NA	NA
Q15843	NEDD8	2	2.55	NA	NA
Q99729	Heterogeneous nuclear ribonucleoprotein A/B	2	2.53	NA	NA
P30837	Aldehyde dehydrogenase X, mitochondrial	3	2.51	0.217	0.051
P04233	HLA class II histocompatibility antigen gamma chain	1	2.50	NA	NA
Q9BV36	Melanophilin	3	2.49	0.438	0.173
P23588	Eukaryotic translation initiation factor 4B	1	2.48	NA	NA
Q14376	UDP-glucose 4-epimerase	1	2.48	NA	NA
P20702	Integrin alpha-X	1	2.48	NA	NA
Q3LXA3	Bifunctional ATP-dependent dihydroxyacetone kinase/FAD-AMP lyase (cyclizing)	1	2.47	NA	NA
P55008	Allograft inflammatory factor 1	2	2.46	NA	NA
P08236	Beta-glucuronidase	2	2.46	NA	NA
Q9BUP0	EF-hand domain-containing protein D1	1	2.44	NA	NA
P50453	Serpin B9	1	2.43	NA	NA
P02786	Transferrin receptor protein 1	1	2.43	NA	NA
P02792	Ferritin light chain	1	2.43	NA	NA
Q9UL46	Proteasome activator complex subunit 2	3	2.43	0.364	0.136
P08246	Neutrophil elastase	1	2.42	NA	NA
Q15181	Inorganic pyrophosphatase	3	2.41	0.362	0.136
Q9Y3C8	Ubiquitin-fold modifier-conjugating enzyme 1	1	2.40	NA	NA
Q9UHB6	LIM domain and actin-binding protein 1	1	2.39	NA	NA
P19971	Thymidine phosphorylase	3	2.38	0.465	0.204
P51688	N-sulphoglucosamine sulphohydrolase	1	2.38	NA	NA
P61916	Epididymal secretory protein E1	1	2.38	NA	NA
Q03154	Aminoacylase-1	1	2.37	NA	NA
P24941	Cyclin-dependent kinase 2	1	2.35	NA	NA
Q63HN8	E3 ubiquitin-protein ligase RNF213	1	2.35	NA	NA
Q9NY33	Dipeptidyl peptidase 3	1	2.34	NA	NA
Q13573	SNW domain-containing protein 1	1	2.34	NA	NA
P02765	Alpha-2-HS-glycoprotein	4	2.34	0.383	0.113
P10619	Lysosomal protective protein	1	2.33	NA	NA
Q13303	Voltage-gated potassium channel subunit beta-2	1	2.31	NA	NA
P28065	Proteasome subunit beta type-9	1	2.31	NA	NA
P07093	Glia-derived nexin	3	2.30	0.211	0.059
P14550	Alcohol dehydrogenase [NADP(+)]	3	2.30	0.203	0.054
P01620	Ig kappa chain V-III region SIE	1	2.30	NA	NA
Q6UW68	Transmembrane protein 205	1	2.29	NA	NA
P05155	Plasma protease C1 inhibitor	2	2.28	NA	NA
Q6PIU2	Neutral cholesterol ester hydrolase 1	1	2.25	NA	NA
P12429	Annexin A3	1	2.25	NA	NA
Q13442	28 kDa heat- and acid-stable phosphoprotein	2	2.23	NA	NA
P10412	Histone H1.4	2	2.22	NA	NA
P10721	Mast/stem cell growth factor receptor Kit	1	2.22	NA	NA
P16188	HLA class I histocompatibility antigen, A-30 alpha chain	1	2.22	NA	NA
P48637	Glutathione synthetase	1	2.21	NA	NA

Table S16-Average Metastatic Tumors

Q9NYL4	Peptidyl-prolyl cis-trans isomerase FKBP11	2	2.21	NA	NA
P63173	60S ribosomal protein L38	1	2.20	NA	NA
Q9NY12	H/ACA ribonucleoprotein complex subunit 1	1	2.20	NA	NA
Q6IAN0	Dehydrogenase/reductase SDR family member 7B	1	2.20	NA	NA
Q92820	Gamma-glutamyl hydrolase	2	2.19	NA	NA
Q13435	Splicing factor 3B subunit 2	2	2.17	NA	NA
O43776	Asparagine--tRNA ligase, cytoplasmic	2	2.17	NA	NA
P04440	HLA class II histocompatibility antigen, DP beta 1 chain	2	2.16	NA	NA
P62826	GTP-binding nuclear protein Ran	5	2.15	0.097	0.001
Q13576	Ras GTPase-activating-like protein IQGAP2	1	2.15	NA	NA
P00751	Complement factor B	1	2.14	NA	NA
P13639	Elongation factor 2	5	2.13	0.129	0.004
P52815	39S ribosomal protein L12, mitochondrial	2	2.13	NA	NA
P30044	Peroxiredoxin-5, mitochondrial	5	2.12	0.076	0.001
P20618	Proteasome subunit beta type-1	4	2.12	0.136	0.012
Q9NR56	Muscleblind-like protein 1	1	2.10	NA	NA
P11215	Integrin alpha-M	1	2.10	NA	NA
P52209	6-phosphogluconate dehydrogenase, decarboxylating	1	2.09	NA	NA
Q92688	Acidic leucine-rich nuclear phosphoprotein 32 family member B	5	2.09	0.081	0.001
Q86UX7	Fermitin family homolog 3	2	2.08	NA	NA
Q96KP4	Cytosolic non-specific dipeptidase	4	2.08	0.157	0.019
A4D1P6	WD repeat-containing protein 91	2	2.08	NA	NA
P13797	Plastin-3	4	2.07	0.126	0.010
Q08380	Galectin-3-binding protein	4	2.07	0.145	0.016
Q13243	Serine/arginine-rich splicing factor 5	2	2.07	NA	NA
P42224	Signal transducer and activator of transcription 1-alpha/beta	3	2.06	0.390	0.206
Q12906	Interleukin enhancer-binding factor 3	5	2.05	0.030	0.000
P53999	Activated RNA polymerase II transcriptional coactivator p15	3	2.05	0.066	0.008
Q96DB5	Regulator of microtubule dynamics protein 1	1	2.05	NA	NA
O15371	Eukaryotic translation initiation factor 3 subunit D	1	2.04	NA	NA
P21399	Cytoplasmic aconitate hydratase	1	2.03	NA	NA
P36871	Phosphoglucomutase-1	2	2.03	NA	NA
Q9NQ39	Putative 40S ribosomal protein S10-like	1	2.02	NA	NA
P25788	Proteasome subunit alpha type-3	4	2.02	0.134	0.013
Q99523	Sortilin	3	2.02	0.237	0.097
Q9GZY8	Mitochondrial fission factor	2	2.02	NA	NA
O75874	Isocitrate dehydrogenase [NADP] cytoplasmic	2	2.02	NA	NA
P49588	Alanine--tRNA ligase, cytoplasmic	1	2.01	NA	NA
P62750	60S ribosomal protein L23a	4	2.01	0.198	0.039
P25786	Proteasome subunit alpha type-1	5	2.01	0.191	0.022
O00154	Cytosolic acyl coenzyme A thioester hydrolase	3	2.01	0.083	0.014
P62857	40S ribosomal protein S28	2	2.00	NA	NA
P00338	L-lactate dehydrogenase A chain	5	2.00	0.195	0.024
Q14978	Nucleolar and coiled-body phosphoprotein 1	1	1.99	NA	NA
P02763	Alpha-1-acid glycoprotein 1	3	1.99	0.480	0.289
P84077	ADP-ribosylation factor 1	3	1.99	0.224	0.092
P45973	Chromobox protein homolog 5	1	1.98	NA	NA
P08238	Heat shock protein HSP 90-beta	5	1.97	0.125	0.006
P13489	Ribonuclease inhibitor	2	1.97	NA	NA
Q07955	Serine/arginine-rich splicing factor 1	5	1.96	0.134	0.007
P00734	Prothrombin	2	1.96	NA	NA
P62158	Calmodulin	5	1.96	0.111	0.004
O14561	Acyl carrier protein, mitochondrial	1	1.95	NA	NA
Q01130	Serine/arginine-rich splicing factor 2	2	1.94	NA	NA
O60841	Eukaryotic translation initiation factor 5B	1	1.94	NA	NA
Q9UKV3	Apoptotic chromatin condensation inducer in the nucleus	2	1.94	NA	NA
P54819	Adenylate kinase 2, mitochondrial	4	1.93	0.106	0.008
Q12931	Heat shock protein 75 kDa, mitochondrial	1	1.93	NA	NA
P13760	HLA class II histocompatibility antigen, DRB1-4 beta chain	1	1.92	NA	NA
Q06323	Proteasome activator complex subunit 1	5	1.92	0.194	0.028
Q9Y2W1	Thyroid hormone receptor-associated protein 3	2	1.92	NA	NA
P46776	60S ribosomal protein L27a	3	1.91	0.059	0.008
P28066	Proteasome subunit alpha type-5	4	1.91	0.231	0.068
P40926	Malate dehydrogenase, mitochondrial	5	1.91	0.192	0.028
O75083	WD repeat-containing protein 1	5	1.91	0.084	0.002
Q9NYU1	UDP-glucose:glycoprotein glucosyltransferase 2	1	1.91	NA	NA
P55809	Succinyl-CoA:3-ketoacid coenzyme A transferase 1, mitochondrial	3	1.90	0.173	0.066
P60900	Proteasome subunit alpha type-6	3	1.90	0.101	0.024
P0DME0	Protein SETSIP	2	1.90	NA	NA
Q9HC38	Glyoxalase domain-containing protein 4	2	1.89	NA	NA
P02766	Transthyretin	2	1.89	NA	NA
P37802	Transgelin-2	3	1.88	0.037	0.003
Q8N183	Mimitin, mitochondrial	1	1.88	NA	NA
Q96JB5	CDK5 regulatory subunit-associated protein 3	1	1.88	NA	NA
P28838	Cytosol aminopeptidase	4	1.87	0.297	0.125
P49773	Histidine triad nucleotide-binding protein 1	3	1.87	0.317	0.187
P08670	Vimentin	5	1.87	0.282	0.091
P63279	SUMO-conjugating enzyme UBC9	2	1.87	NA	NA
Q9NVH1	DnaJ homolog subfamily C member 11	1	1.86	NA	NA
P39687	Acidic leucine-rich nuclear phosphoprotein 32 family member A	4	1.86	0.137	0.020
P35268	60S ribosomal protein L22	1	1.86	NA	NA
P07954	Fumarate hydratase, mitochondrial	2	1.85	NA	NA
P37837	Transaldolase	5	1.85	0.066	0.001
Q8IZQ5	Selenoprotein H	2	1.84	NA	NA
Q3SY69	Mitochondrial 10-formyltetrahydrofolate dehydrogenase	2	1.84	NA	NA
Q9H3N1	Thioredoxin-related transmembrane protein 1	1	1.83	NA	NA
O00299	Chloride intracellular channel protein 1	4	1.83	0.079	0.005
P36955	Pigment epithelium-derived factor	3	1.83	0.313	0.193
P01889	HLA class I histocompatibility antigen, B-7 alpha chain	1	1.83	NA	NA
P30504	HLA class I histocompatibility antigen, Cw-4 alpha chain	1	1.82	NA	NA
P07602	Prosaposin	5	1.81	0.130	0.010
Q7Z7H5	Transmembrane emp24 domain-containing protein 4	1	1.81	NA	NA
Q8NC51	Plasminogen activator inhibitor 1 RNA-binding protein	3	1.81	0.121	0.039
P13929	Beta-enolase	1	1.81	NA	NA
O75351	Vacuolar protein sorting-associated protein 4B	1	1.80	NA	NA
P07910	Heterogeneous nuclear ribonucleoproteins C1/C2	5	1.80	0.071	0.001
Q08AM6	Protein VAC14 homolog	2	1.80	NA	NA
Q99538	Legumain	1	1.79	NA	NA
Q96AG4	Leucine-rich repeat-containing protein 59	4	1.79	0.119	0.016
P24821	Tenascin	1	1.78	NA	NA
P55786	Puromycin-sensitive aminopeptidase	4	1.78	0.064	0.003
P12004	Proliferating cell nuclear antigen	1	1.77	NA	NA
O00567	Nucleolar protein 56	3	1.77	0.185	0.091
P43243	Matrin-3	3	1.76	0.063	0.012
Q15907	Ras-related protein Rab-11B	5	1.75	0.115	0.008
P61204	ADP-ribosylation factor 3	2	1.75	NA	NA
Q15631	Translin	3	1.75	0.047	0.007
P09211	Glutathione S-transferase P	5	1.74	0.156	0.024
P21796	Voltage-dependent anion-selective channel protein 1	5	1.74	0.160	0.025
O43399	Tumor protein D54	3	1.74	0.056	0.010
Q00341	Vigilin	1	1.74	NA	NA
P30040	Endoplasmic reticulum resident protein 29	3	1.73	0.335	0.242
Q9Y4W6	AFG3-like protein 2	5	1.73	0.084	0.003
P36543	V-type proton ATPase subunit E 1	5	1.73	0.143	0.019
P06753	Tropomyosin alpha-3 chain	5	1.72	0.191	0.047
O00160	Unconventional myosin-If	2	1.72	NA	NA
P05090	Apolipoprotein D	1	1.72	NA	NA
P05141	ADP/ATP translocase 2	4	1.71	0.062	0.003
Q00688	Peptidyl-prolyl cis-trans isomerase FKBP3	2	1.71	NA	NA
P22314	Ubiquitin-like modifier-activating enzyme 1	5	1.70	0.085	0.003
Q9BZZ5	Apoptosis inhibitor 5	4	1.70	0.065	0.004
P26440	Isovaleryl-CoA dehydrogenase, mitochondrial	1	1.70	NA	NA
P06737	Glycogen phosphorylase, liver form	5	1.70	0.394	0.249
P48426	Phosphatidylinositol 5-phosphate 4-kinase type-2 alpha	3	1.70	0.178	0.096
P08574	Cytochrome c1, heme protein, mitochondrial	4	1.70	0.237	0.111
Q16629	Serine/arginine-rich splicing factor 7	2	1.70	NA	NA
Q8NFV4	Alpha/beta hydrolase domain-containing protein 11	1	1.70	NA	NA
P00441	Superoxide dismutase [Cu-Zn]	3	1.70	0.171	0.091
P99999	Cytochrome c	5	1.69	0.216	0.071
P10155	60 kDa SS-A/Ro ribonucleoprotein	1	1.69	NA	NA
Q12905	Interleukin enhancer-binding factor 2	5	1.69	0.051	0.001
Q9H2U2	Inorganic pyrophosphatase 2, mitochondrial	4	1.68	0.123	0.024
P49792	E3 SUMO-protein ligase RanBP2	1	1.68	NA	NA
Q9Y2Q3	Glutathione S-transferase kappa 1	4	1.68	0.231	0.111
P62847	40S ribosomal protein S24	1	1.67	NA	NA
P08195	4F2 cell-surface antigen heavy chain	5	1.67	0.130	0.017
Q07960	Rho GTPase-activating protein 1	5	1.67	0.132	0.018
Q5JRX3	Presequence protease, mitochondrial	3	1.66	0.052	0.010
Q14103	Heterogeneous nuclear ribonucleoprotein D0	5	1.66	0.141	0.023
O75822	Eukaryotic translation initiation factor 3 subunit J	1	1.66	NA	NA
O14737	Programmed cell death protein 5	1	1.65	NA	NA
O00560	Syntenin-1	2	1.65	NA	NA
O76021	Ribosomal L1 domain-containing protein 1	3	1.65	0.159	0.088
Q6GMV3	Putative peptidyl-tRNA hydrolase PTRHD1	1	1.65	NA	NA
P46783	40S ribosomal protein S10	2	1.65	NA	NA
Q08945	FACT complex subunit SSRP1	2	1.64	NA	NA
Q13423	NAD(P) transhydrogenase, mitochondrial	5	1.64	0.088	0.005
P23284	Peptidyl-prolyl cis-trans isomerase B	5	1.64	0.086	0.004
P05387	60S acidic ribosomal protein P2	5	1.64	0.163	0.039
P61769	Beta-2-microglobulin	3	1.63	0.225	0.161
Q00839	Heterogeneous nuclear ribonucleoprotein U	5	1.63	0.120	0.015
Q96C19	EF-hand domain-containing protein D2	3	1.63	0.107	0.045
P16402	Histone H1.3	1	1.63	NA	NA
Q8IVF2	Protein AHNAK2	4	1.62	0.253	0.152
Q13547	Histone deacetylase 1	1	1.62	NA	NA
O95182	NADH dehydrogenase [ubiquinone] 1 alpha subcomplex subunit 7	1	1.62	NA	NA
Q02818	Nucleobindin-1	4	1.62	0.025	0.000
P09429	High mobility group protein B1	5	1.62	0.188	0.064
O14979	Heterogeneous nuclear ribonucleoprotein D-like	3	1.61	0.100	0.041
P26641	Elongation factor 1-gamma	5	1.61	0.097	0.008
P08621	U1 small nuclear ribonucleoprotein 70 kDa	2	1.60	NA	NA
Q06787	Fragile X mental retardation protein 1	3	1.60	0.164	0.104
P09651	Heterogeneous nuclear ribonucleoprotein A1	5	1.60	0.055	0.001
P62241	40S ribosomal protein S8	4	1.60	0.096	0.016
Q15424	Scaffold attachment factor B1	1	1.59	NA	NA
P01034	Cystatin-C	1	1.59	NA	NA
P02790	Hemopexin	5	1.58	0.223	0.109
P18669	Phosphoglycerate mutase 1	5	1.58	0.165	0.050
Q15063	Periostin	3	1.58	0.297	0.263
P35659	Protein DEK	1	1.58	NA	NA
Q13263	Transcription intermediary factor 1-beta	4	1.58	0.073	0.008
P30464	HLA class I histocompatibility antigen, B-15 alpha chain	1	1.57	NA	NA
Q1KMD3	Heterogeneous nuclear ribonucleoprotein U-like protein 2	5	1.57	0.120	0.020
Q96D96	Voltage-gated hydrogen channel 1	1	1.56	NA	NA
P01625	Ig kappa chain V-IV region Len	2	1.56	NA	NA
Q9UH65	Switch-associated protein 70	2	1.56	NA	NA
P25398	40S ribosomal protein S12	5	1.56	0.069	0.003
P04075	Fructose-bisphosphate aldolase A	5	1.56	0.039	0.000
Q9Y5X3	Sorting nexin-5	2	1.55	NA	NA
P51810	G-protein coupled receptor 143	5	1.55	0.159	0.050
P51665	26S proteasome non-ATPase regulatory subunit 7	2	1.55	NA	NA
P07814	Bifunctional glutamate/proline--tRNA ligase	2	1.55	NA	NA
Q08211	ATP-dependent RNA helicase A	5	1.55	0.098	0.011
P30484	HLA class I histocompatibility antigen, B-46 alpha chain	1	1.54	NA	NA
P17540	Creatine kinase S-type, mitochondrial	2	1.54	NA	NA
O43852	Calumenin	5	1.54	0.063	0.002
P62258	14-3-3 protein epsilon	4	1.54	0.064	0.007

Q16698	2,4-dienoyl-CoA reductase, mitochondrial	5	1.53	0.081	0.006
Q92616	Translational activator GCN1	1	1.53	NA	NA
O75390	Citrate synthase, mitochondrial	5	1.53	0.050	0.001
P14854	Cytochrome c oxidase subunit 6B1	4	1.52	0.122	0.041
P30533	Alpha-2-macroglobulin receptor-associated protein	5	1.52	0.076	0.005
Q14247	Src substrate cortactin	4	1.52	0.106	0.029
P61604	10 kDa heat shock protein, mitochondrial	5	1.51	0.278	0.211
P27348	14-3-3 protein theta	4	1.51	0.117	0.039
P09668	Pro-cathepsin H	1	1.51	NA	NA
Q07020	60S ribosomal protein L18	2	1.51	NA	NA
P02042	Hemoglobin subunit delta	3	1.51	0.725	0.627
P30101	Protein disulfide-isomerase A3	5	1.51	0.118	0.026
P01859	Ig gamma-2 chain C region	4	1.51	0.337	0.311
Q01518	Adenylyl cyclase-associated protein 1	2	1.51	NA	NA
P39748	Flap endonuclease 1	2	1.51	NA	NA
P46940	Ras GTPase-activating-like protein IQGAP1	5	1.51	0.056	0.002
P10809	60 kDa heat shock protein, mitochondrial	5	1.50	0.064	0.003
Q9Y3D0	Mitotic spindle-associated MMXD complex subunit MIP18	1	1.50	NA	NA
P61019	Ras-related protein Rab-2A	2	1.50	NA	NA
Q9GZT3	SRA stem-loop-interacting RNA-binding protein, mitochondrial	2	1.50	NA	NA
P31150	Rab GDP dissociation inhibitor alpha	1	1.50	NA	NA
Q9Y6C9	Mitochondrial carrier homolog 2	3	1.49	0.137	0.099
P22626	Heterogeneous nuclear ribonucleoproteins A2/B1	5	1.49	0.065	0.004
P42704	Leucine-rich PPR motif-containing protein, mitochondrial	4	1.48	0.097	0.027
P51149	Ras-related protein Rab-7a	5	1.48	0.152	0.061
O95302	Peptidyl-prolyl cis-trans isomerase FKBP9	2	1.48	NA	NA
P31949	Protein S100-A11	5	1.48	0.122	0.032
O43488	Aflatoxin B1 aldehyde reductase member 2	2	1.48	NA	NA
Q71UM5	40S ribosomal protein S27-like	2	1.48	NA	NA
Q5VTE0	Putative elongation factor 1-alpha-like 3	5	1.48	0.110	0.024
P46926	Glucosamine-6-phosphate isomerase 1	4	1.48	0.161	0.094
Q15904	V-type proton ATPase subunit S1	1	1.48	NA	NA
P12830	Cadherin-1	1	1.47	NA	NA
P08697	Alpha-2-antiplasmin	1	1.47	NA	NA
P48739	Phosphatidylinositol transfer protein beta isoform	1	1.47	NA	NA
Q15717	ELAV-like protein 1	2	1.47	NA	NA
P46063	ATP-dependent DNA helicase Q1	1	1.47	NA	NA
P07203	Glutathione peroxidase 1	1	1.46	NA	NA
Q99873	Protein arginine N-methyltransferase 1	2	1.46	NA	NA
P30405	Peptidyl-prolyl cis-trans isomerase F, mitochondrial	2	1.46	NA	NA
P63220	40S ribosomal protein S21	1	1.46	NA	NA
P09661	U2 small nuclear ribonucleoprotein A'	1	1.46	NA	NA
Q86UE4	Protein LYRIC	4	1.45	0.139	0.074
Q8NBJ7	Sulfatase-modifying factor 2	3	1.45	0.272	0.302
P18621	60S ribosomal protein L17	4	1.45	0.128	0.061
P14317	Hematopoietic lineage cell-specific protein	2	1.45	NA	NA
Q9UKM9	RNA-binding protein Raly	5	1.45	0.076	0.008
P62263	40S ribosomal protein S14	5	1.45	0.147	0.066
Q9P2R7	Succinyl-CoA ligase [ADP-forming] subunit beta, mitochondrial	2	1.45	NA	NA
P62851	40S ribosomal protein S25	5	1.44	0.164	0.090
Q16610	Extracellular matrix protein 1	2	1.44	NA	NA
P61421	V-type proton ATPase subunit d 1	5	1.44	0.137	0.056
Q14974	Importin subunit beta-1	5	1.44	0.110	0.029
O75533	Splicing factor 3B subunit 1	3	1.44	0.076	0.040
Q99614	Tetratricopeptide repeat protein 1	1	1.44	NA	NA
Q5T1M5	FK506-binding protein 15	1	1.44	NA	NA
Q8IY95	Transmembrane protein 192	1	1.43	NA	NA
P62136	Serine/threonine-protein phosphatase PP1-alpha catalytic subunit	4	1.43	0.051	0.006
P62829	60S ribosomal protein L23	4	1.43	0.102	0.040
Q92890	Ubiquitin fusion degradation protein 1 homolog	2	1.43	NA	NA
Q92945	Far upstream element-binding protein 2	4	1.43	0.051	0.006
O75380	NADH dehydrogenase [ubiquinone] iron-sulfur protein 6, mitochondrial	2	1.43	NA	NA
P54136	Arginine--tRNA ligase, cytoplasmic	5	1.43	0.117	0.038
P51159	Ras-related protein Rab-27A	3	1.43	0.073	0.040
Q99798	Aconitate hydratase, mitochondrial	5	1.43	0.086	0.014
P13804	Electron transfer flavoprotein subunit alpha, mitochondrial	2	1.43	NA	NA
P52597	Heterogeneous nuclear ribonucleoprotein F	4	1.42	0.028	0.001
Q9UBQ7	Glyoxylate reductase/hydroxypyruvate reductase	1	1.42	NA	NA
P78527	DNA-dependent protein kinase catalytic subunit	5	1.42	0.116	0.038
P11142	Heat shock cognate 71 kDa protein	5	1.42	0.049	0.002
Q96TC7	Regulator of microtubule dynamics protein 3	2	1.42	NA	NA
P02746	Complement C1q subcomponent subunit B	2	1.42	NA	NA
P49207	60S ribosomal protein L34	4	1.42	0.133	0.077
Q9Y5J7	Mitochondrial import inner membrane translocase subunit Tim9	1	1.42	NA	NA
P62081	40S ribosomal protein S7	2	1.42	NA	NA
Q9NYU2	UDP-glucose:glycoprotein glucosyltransferase 1	2	1.42	NA	NA
P07237	Protein disulfide-isomerase	5	1.42	0.099	0.025
O00410	Importin-5	2	1.41	NA	NA
P29728	2'-5'-oligoadenylate synthase 2	1	1.41	NA	NA
P29692	Elongation factor 1-delta	5	1.41	0.133	0.060
O15118	Niemann-Pick C1 protein	2	1.41	NA	NA
Q9Y383	Putative RNA-binding protein Luc7-like 2	3	1.41	0.210	0.242
Q92598	Heat shock protein 105 kDa	2	1.41	NA	NA
Q16836	Hydroxyacyl-coenzyme A dehydrogenase, mitochondrial	3	1.41	0.097	0.071
P83731	60S ribosomal protein L24	4	1.41	0.215	0.207
Q99572	P2X purinoceptor 7	3	1.41	0.385	0.465
Q14677	Clathrin interactor 1	2	1.41	NA	NA
P30084	Enoyl-CoA hydratase, mitochondrial	4	1.41	0.033	0.002
Q96I99	Succinyl-CoA ligase [GDP-forming] subunit beta, mitochondrial	1	1.41	NA	NA
Q9UNZ2	NSFL1 cofactor p47	1	1.41	NA	NA
P37108	Signal recognition particle 14 kDa protein	2	1.41	NA	NA
O95299	NADH dehydrogenase [ubiquinone] 1 alpha subcomplex subunit 10, mitochondrial	2	1.41	NA	NA
Q15366	Poly(rC)-binding protein 2	1	1.41	NA	NA
P07919	Cytochrome b-c1 complex subunit 6, mitochondrial	4	1.40	0.281	0.314
Q9Y2B0	Protein canopy homolog 2	1	1.40	NA	NA
Q9HB90	Ras-related GTP-binding protein C	1	1.40	NA	NA
P19404	NADH dehydrogenase [ubiquinone] flavoprotein 2, mitochondrial	1	1.40	NA	NA
Q13123	Protein Red	1	1.40	NA	NA
P21283	V-type proton ATPase subunit C 1	3	1.40	0.089	0.064
P26599	Polypyrimidine tract-binding protein 1	5	1.40	0.047	0.002
Q99536	Synaptic vesicle membrane protein VAT-1 homolog	5	1.40	0.174	0.127
P19827	Inter-alpha-trypsin inhibitor heavy chain H1	1	1.40	NA	NA
P16219	Short-chain specific acyl-CoA dehydrogenase, mitochondrial	3	1.39	0.040	0.015
P13010	X-ray repair cross-complementing protein 5	5	1.39	0.046	0.002
Q9UHX1	Poly(U)-binding-splicing factor PUF60	4	1.39	0.070	0.019
P27144	Adenylate kinase 4, mitochondrial	1	1.39	NA	NA
P12270	Nucleoprotein TPR	4	1.38	0.068	0.018
P62140	Serine/threonine-protein phosphatase PP1-beta catalytic subunit	1	1.38	NA	NA
P21291	Cysteine and glycine-rich protein 1	1	1.38	NA	NA
Q16655	Melanoma antigen recognized by T-cells 1	1	1.37	NA	NA
P09622	Dihydrolipoyl dehydrogenase, mitochondrial	5	1.37	0.099	0.032
P14406	Cytochrome c oxidase subunit 7A2, mitochondrial	1	1.37	NA	NA
Q04917	14-3-3 protein eta	1	1.37	NA	NA
Q13617	Cullin-2	1	1.37	NA	NA
P11137	Microtubule-associated protein 2	1	1.37	NA	NA
P05388	60S acidic ribosomal protein P0	5	1.36	0.034	0.001
O15145	Actin-related protein 2/3 complex subunit 3	4	1.36	0.123	0.087
P51608	Methyl-CpG-binding protein 2	3	1.36	0.272	0.375
P49756	RNA-binding protein 25	1	1.36	NA	NA
Q9NX63	Coiled-coil-helix-coiled-coil-helix domain-containing protein 3, mitochondrial	4	1.36	0.063	0.016
Q07021	Complement component 1 Q subcomponent-binding protein, mitochondrial	2	1.36	NA	NA
P62906	60S ribosomal protein L10a	5	1.36	0.048	0.003
Q15046	Lysine--tRNA ligase	3	1.36	0.239	0.330
O14773	Tripeptidyl-peptidase 1	5	1.35	0.086	0.025
P61247	40S ribosomal protein S3a	5	1.35	0.106	0.046
O60506	Heterogeneous nuclear ribonucleoprotein Q	4	1.35	0.095	0.050
Q9NP81	Serine--tRNA ligase, mitochondrial	4	1.35	0.179	0.192
P19367	Hexokinase-1	5	1.35	0.185	0.179
P0CW22	40S ribosomal protein S17-like	5	1.35	0.049	0.004
P22059	Oxysterol-binding protein 1	2	1.35	NA	NA
Q08379	Golgin subfamily A member 2	1	1.35	NA	NA
P08865	40S ribosomal protein SA	5	1.35	0.089	0.029
P61978	Heterogeneous nuclear ribonucleoprotein K	5	1.34	0.091	0.031
Q12904	Aminoacyl tRNA synthase complex-interacting multifunctional protein 1	1	1.34	NA	NA
Q9Y3Z3	Deoxynucleoside triphosphate triphosphohydrolase SAMHD1	4	1.34	0.224	0.281
Q9NTZ6	RNA-binding protein 12	3	1.34	0.062	0.042
P62191	26S protease regulatory subunit 4	2	1.34	NA	NA
P15153	Ras-related C3 botulinum toxin substrate 2	2	1.34	NA	NA
O43598	2'-deoxynucleoside 5'-phosphate N-hydrolase 1	1	1.34	NA	NA
P35232	Prohibitin	5	1.34	0.190	0.200
Q9H2G2	STE20-like serine/threonine-protein kinase	1	1.34	NA	NA
P51991	Heterogeneous nuclear ribonucleoprotein A3	5	1.33	0.078	0.021
P43490	Nicotinamide phosphoribosyltransferase	4	1.33	0.201	0.247
Q9BR76	Coronin-1B	4	1.33	0.144	0.138
Q16666	Gamma-interferon-inducible protein 16	2	1.33	NA	NA
Q00059	Transcription factor A, mitochondrial	3	1.33	0.304	0.447
Q86V81	THO complex subunit 4	2	1.33	NA	NA
O00483	NADH dehydrogenase [ubiquinone] 1 alpha subcomplex subunit 4	4	1.33	0.175	0.204
P50213	Isocitrate dehydrogenase [NAD] subunit alpha, mitochondrial	5	1.33	0.085	0.030
Q9NVI7	ATPase family AAA domain-containing protein 3A	2	1.33	NA	NA
P78347	General transcription factor II-I	4	1.32	0.035	0.004
P38606	V-type proton ATPase catalytic subunit A	5	1.32	0.155	0.145
P53597	Succinyl-CoA ligase [ADP/GDP-forming] subunit alpha, mitochondrial	3	1.32	0.100	0.108
Q13488	V-type proton ATPase 116 kDa subunit a isoform 3	2	1.32	NA	NA
P12956	X-ray repair cross-complementing protein 6	5	1.32	0.039	0.002
P62979	Ubiquitin-40S ribosomal protein S27a	2	1.32	NA	NA
Q9Y3D9	28S ribosomal protein S23, mitochondrial	1	1.32	NA	NA
P49721	Proteasome subunit beta type-2	1	1.32	NA	NA
Q9Y4I1	Unconventional myosin-Va	4	1.32	0.092	0.058
P08575	Receptor-type tyrosine-protein phosphatase C	2	1.32	NA	NA
Q562E7	WD repeat-containing protein 81	1	1.32	NA	NA
P63244	Guanine nucleotide-binding protein subunit beta-2-like 1	5	1.31	0.032	0.001
P60981	Destrin	4	1.31	0.159	0.186
O75436	Vacuolar protein sorting-associated protein 26A	1	1.31	NA	NA
P01857	Ig gamma-1 chain C region	5	1.31	0.307	0.428
Q8NBQ5	Estradiol 17-beta-dehydrogenase 11	1	1.31	NA	NA
P53990	IST1 homolog	1	1.31	NA	NA
P41091	Eukaryotic translation initiation factor 2 subunit 3	1	1.31	NA	NA
P62330	ADP-ribosylation factor 6	1	1.31	NA	NA
O43681	ATPase ASNA1	1	1.30	NA	NA
Q9NQP4	Prefoldin subunit 4	1	1.30	NA	NA
Q14152	Eukaryotic translation initiation factor 3 subunit A	5	1.30	0.034	0.001
P02774	Vitamin D-binding protein	5	1.30	0.354	0.496
P38646	Stress-70 protein, mitochondrial	5	1.30	0.067	0.017
P52272	Heterogeneous nuclear ribonucleoprotein M	5	1.30	0.093	0.049
P30050	60S ribosomal protein L12	3	1.30	0.115	0.152
Q99623	Prohibitin-2	5	1.29	0.194	0.256
Q9P2E9	Ribosome-binding protein 1	5	1.29	0.117	0.094
P09669	Cytochrome c oxidase subunit 6C	3	1.29	0.069	0.066
Q5JTV8	Torsin-1A-interacting protein 1	2	1.29	NA	NA
Q9BVK6	Transmembrane emp24 domain-containing protein 9	2	1.29	NA	NA
P26639	Threonine--tRNA ligase, cytoplasmic	1	1.29	NA	NA
P62249	40S ribosomal protein S16	5	1.28	0.031	0.001
Q99805	Transmembrane 9 superfamily member 2	2	1.28	NA	NA
P38159	RNA-binding motif protein, X chromosome	3	1.28	0.189	0.326
P36776	Lon protease homolog, mitochondrial	1	1.28	NA	NA
P54652	Heat shock-related 70 kDa protein 2	5	1.27	0.129	0.133

P26885	Peptidyl-prolyl cis-trans isomerase FKBP2	2	1.27	NA	NA
Q86VP6	Cullin-associated NEDD8-dissociated protein 1	5	1.27	0.076	0.034
O94906	Pre-mRNA-processing factor 6	2	1.27	NA	NA
P59998	Actin-related protein 2/3 complex subunit 4	5	1.27	0.137	0.155
Q15029	116 kDa U5 small nuclear ribonucleoprotein component	5	1.27	0.244	0.384
Q9NYF8	Bcl-2-associated transcription factor 1	1	1.27	NA	NA
Q9ULV4	Coronin-1C	3	1.27	0.087	0.114
Q13098	COP9 signalosome complex subunit 1	1	1.26	NA	NA
Q15233	Non-POU domain-containing octamer-binding protein	5	1.26	0.055	0.013
P51572	B-cell receptor-associated protein 31	5	1.26	0.133	0.155
P24534	Elongation factor 1-beta	5	1.26	0.162	0.226
P14866	Heterogeneous nuclear ribonucleoprotein L	5	1.25	0.060	0.019
Q9Y608	Leucine-rich repeat flightless-interacting protein 2	1	1.25	NA	NA
Q8NBJ5	Procollagen galactosyltransferase 1	3	1.25	0.352	0.589
P34932	Heat shock 70 kDa protein 4	4	1.25	0.073	0.056
P10644	cAMP-dependent protein kinase type I-alpha regulatory subunit	4	1.25	0.126	0.174
Q9GZS3	WD repeat-containing protein 61	1	1.25	NA	NA
P19823	Inter-alpha-trypsin inhibitor heavy chain H2	2	1.25	NA	NA
O75494	Serine/arginine-rich splicing factor 10	1	1.25	NA	NA
P09874	Poly [ADP-ribose] polymerase 1	5	1.24	0.032	0.002
P30042	ES1 protein homolog, mitochondrial	4	1.24	0.219	0.396
Q09028	Histone-binding protein RBBP4	2	1.24	NA	NA
Q9BWM7	Sideroflexin-3	2	1.24	NA	NA
Q15459	Splicing factor 3A subunit 1	1	1.24	NA	NA
Q00765	Receptor expression-enhancing protein 5	4	1.24	0.128	0.195
O95202	LETM1 and EF-hand domain-containing protein 1, mitochondrial	5	1.24	0.057	0.021
Q8N2K0	Monoacylglycerol lipase ABHD12	1	1.23	NA	NA
P13674	Prolyl 4-hydroxylase subunit alpha-1	3	1.23	0.088	0.140
P27635	60S ribosomal protein L10	5	1.23	0.084	0.066
Q96AY3	Peptidyl-prolyl cis-trans isomerase FKBP10	1	1.23	NA	NA
O43809	Cleavage and polyadenylation specificity factor subunit 5	2	1.23	NA	NA
P23246	Splicing factor, proline- and glutamine-rich	5	1.23	0.082	0.063
O75352	Mannose-P-dolichol utilization defect 1 protein	1	1.23	NA	NA
Q8N163	Cell cycle and apoptosis regulator protein 2	1	1.23	NA	NA
Q5JPE7	Nodal modulator 2	1	1.23	NA	NA
P46782	40S ribosomal protein S5	2	1.23	NA	NA
Q07000	HLA class I histocompatibility antigen, Cw-15 alpha chain	1	1.23	NA	NA
P09012	U1 small nuclear ribonucleoprotein A	3	1.23	0.043	0.041
Q9P016	Thymocyte nuclear protein 1	1	1.23	NA	NA
P04179	Superoxide dismutase [Mn], mitochondrial	5	1.23	0.212	0.392
P46779	60S ribosomal protein L28	3	1.22	0.044	0.045
O94874	E3 UFM1-protein ligase 1	2	1.22	NA	NA
P61586	Transforming protein RhoA	1	1.22	NA	NA
Q9BUJ2	Heterogeneous nuclear ribonucleoprotein U-like protein 1	2	1.22	NA	NA
Q92556	Engulfment and cell motility protein 1	1	1.22	NA	NA
O15144	Actin-related protein 2/3 complex subunit 2	5	1.22	0.139	0.221
O95168	NADH dehydrogenase [ubiquinone] 1 beta subcomplex subunit 4	1	1.22	NA	NA
Q12874	Splicing factor 3A subunit 3	1	1.22	NA	NA
P17844	Probable ATP-dependent RNA helicase DDX5	4	1.22	0.028	0.006
Q04637	Eukaryotic translation initiation factor 4 gamma 1	2	1.22	NA	NA
P39023	60S ribosomal protein L3	5	1.22	0.052	0.019
P26368	Splicing factor U2AF 65 kDa subunit	3	1.22	0.095	0.175
P49591	Serine--tRNA ligase, cytoplasmic	5	1.22	0.068	0.046
P26640	Valine--tRNA ligase	3	1.22	0.038	0.035
Q9Y394	Dehydrogenase/reductase SDR family member 7	2	1.21	NA	NA
Q15365	Poly(rC)-binding protein 1	4	1.21	0.123	0.214
P09417	Dihydropteridine reductase	2	1.21	NA	NA
P02647	Apolipoprotein A-I	5	1.21	0.287	0.538
P60866	40S ribosomal protein S20	5	1.21	0.096	0.116
P14868	Aspartate--tRNA ligase, cytoplasmic	5	1.21	0.086	0.090
Q16531	DNA damage-binding protein 1	4	1.21	0.102	0.156
P20810	Calpastatin	2	1.21	NA	NA
P63000	Ras-related C3 botulinum toxin substrate 1	4	1.21	0.118	0.203
Q86TX2	Acyl-coenzyme A thioesterase 1	1	1.21	NA	NA
P53618	Coatomer subunit beta	4	1.21	0.062	0.056
O43143	Putative pre-mRNA-splicing factor ATP-dependent RNA helicase DHX15	1	1.21	NA	NA
Q9Y266	Nuclear migration protein nudC	1	1.21	NA	NA
O00151	PDZ and LIM domain protein 1	1	1.21	NA	NA
P11216	Glycogen phosphorylase, brain form	5	1.20	0.109	0.163
P11717	Cation-independent mannose-6-phosphate receptor	1	1.20	NA	NA
P30049	ATP synthase subunit delta, mitochondrial	5	1.20	0.111	0.170
A1L0T0	Acetolactate synthase-like protein	3	1.20	0.080	0.149
P50454	Serpin H1	5	1.20	0.160	0.318
P46778	60S ribosomal protein L21	3	1.20	0.089	0.178
Q53H82	Beta-lactamase-like protein 2	1	1.20	NA	NA
O75367	Core histone macro-H2A.1	5	1.20	0.036	0.008
O75569	Interferon-inducible double-stranded RNA-dependent protein kinase activator A	1	1.20	NA	NA
Q13217	DnaJ homolog subfamily C member 3	2	1.20	NA	NA
P39019	40S ribosomal protein S19	5	1.19	0.222	0.468
P02652	Apolipoprotein A-II	5	1.19	0.394	0.678
P49189	4-trimethylaminobutyraldehyde dehydrogenase	5	1.19	0.141	0.280
O00231	26S proteasome non-ATPase regulatory subunit 11	5	1.19	0.064	0.052
P62820	Ras-related protein Rab-1A	1	1.19	NA	NA
Q9NSD9	Phenylalanine--tRNA ligase beta subunit	3	1.19	0.069	0.128
Q96GK7	Fumarylacetoacetate hydrolase domain-containing protein 2A	2	1.19	NA	NA
Q13162	Peroxiredoxin-4	4	1.19	0.130	0.280
Q9H0U4	Ras-related protein Rab-1B	1	1.19	NA	NA
O15143	Actin-related protein 2/3 complex subunit 1B	4	1.19	0.149	0.334
B5ME19	Eukaryotic translation initiation factor 3 subunit C-like protein	5	1.19	0.041	0.015
Q9Y224	UPF0568 protein C14orf166	1	1.19	NA	NA
O43169	Cytochrome b5 type B	1	1.19	NA	NA
Q8TAQ2	SWI/SNF complex subunit SMARCC2	2	1.18	NA	NA
P62714	Serine/threonine-protein phosphatase 2A catalytic subunit beta isoform	1	1.18	NA	NA
Q8TCT9	Minor histocompatibility antigen H13	1	1.18	NA	NA
P21266	Glutathione S-transferase Mu 3	4	1.18	0.152	0.354
Q07666	KH domain-containing, RNA-binding, signal transduction-associated protein 1	5	1.18	0.065	0.065
P62913	60S ribosomal protein L11	5	1.18	0.021	0.001
P31153	S-adenosylmethionine synthase isoform type-2	4	1.18	0.205	0.482
Q9UBQ0	Vacuolar protein sorting-associated protein 29	2	1.18	NA	NA
P22695	Cytochrome b-c1 complex subunit 2, mitochondrial	5	1.18	0.158	0.364
P62910	60S ribosomal protein L32	1	1.17	NA	NA
P11586	C-1-tetrahydrofolate synthase, cytoplasmic	4	1.17	0.085	0.158
P61353	60S ribosomal protein L27	4	1.17	0.116	0.262
P52907	F-actin-capping protein subunit alpha-1	4	1.17	0.161	0.396
O95298	NADH dehydrogenase [ubiquinone] 1 subunit C2	2	1.17	NA	NA
P17980	26S protease regulatory subunit 6A	2	1.17	NA	NA
P55145	Mesencephalic astrocyte-derived neurotrophic factor	3	1.17	0.108	0.281
P61160	Actin-related protein 2	5	1.17	0.126	0.282
O75844	CAAX prenyl protease 1 homolog	1	1.17	NA	NA
P28161	Glutathione S-transferase Mu 2	2	1.17	NA	NA
P61106	Ras-related protein Rab-14	1	1.17	NA	NA
Q9NR28	Diablo homolog, mitochondrial	3	1.16	0.106	0.291
P30519	Heme oxygenase 2	4	1.16	0.066	0.106
P09525	Annexin A4	5	1.16	0.103	0.221
Q13151	Heterogeneous nuclear ribonucleoprotein A0	5	1.16	0.113	0.257
P06730	Eukaryotic translation initiation factor 4E	1	1.16	NA	NA
P36578	60S ribosomal protein L4	5	1.16	0.101	0.225
Q8N5K1	CDGSH iron-sulfur domain-containing protein 2	5	1.16	0.057	0.064
P06576	ATP synthase subunit beta, mitochondrial	5	1.15	0.105	0.254
O15212	Prefoldin subunit 6	2	1.15	NA	NA
Q9UJU6	Drebrin-like protein	1	1.15	NA	NA
P62318	Small nuclear ribonucleoprotein Sm D3	4	1.15	0.059	0.100
P49755	Transmembrane emp24 domain-containing protein 10	5	1.15	0.040	0.026
Q9NX24	H/ACA ribonucleoprotein complex subunit 2	2	1.15	NA	NA
P28482	Mitogen-activated protein kinase 1	1	1.15	NA	NA
Q15393	Splicing factor 3B subunit 3	2	1.15	NA	NA
P18124	60S ribosomal protein L7	5	1.14	0.028	0.009
Q96PK6	RNA-binding protein 14	1	1.14	NA	NA
P26373	60S ribosomal protein L13	5	1.14	0.175	0.488
P01009	Alpha-1-antitrypsin	5	1.14	0.248	0.619
P45880	Voltage-dependent anion-selective channel protein 2	5	1.14	0.226	0.590
O60568	Procollagen-lysine,2-oxoglutarate 5-dioxygenase 3	1	1.14	NA	NA
Q15084	Protein disulfide-isomerase A6	5	1.14	0.124	0.361
Q92599	Septin-8	1	1.13	NA	NA
Q86UP2	Kinectin	5	1.13	0.050	0.071
P30740	Leukocyte elastase inhibitor	2	1.13	NA	NA
P21281	V-type proton ATPase subunit B, brain isoform	5	1.13	0.129	0.405
P61158	Actin-related protein 3	5	1.13	0.106	0.322
O95292	Vesicle-associated membrane protein-associated protein B/C	5	1.12	0.083	0.235
P27824	Calnexin	5	1.12	0.034	0.027
O43390	Heterogeneous nuclear ribonucleoprotein R	5	1.12	0.162	0.516
O95881	Thioredoxin domain-containing protein 12	4	1.12	0.129	0.440
Q9NZ08	Endoplasmic reticulum aminopeptidase 1	5	1.12	0.131	0.436
P02788	Lactotransferrin	1	1.12	NA	NA
Q9Y262	Eukaryotic translation initiation factor 3 subunit L	3	1.12	0.027	0.055
P62424	60S ribosomal protein L7a	5	1.12	0.126	0.429
P61254	60S ribosomal protein L26	1	1.12	NA	NA
Q06830	Peroxiredoxin-1	5	1.12	0.209	0.629
P31040	Succinate dehydrogenase [ubiquinone] flavoprotein subunit, mitochondrial	5	1.12	0.088	0.285
P00505	Aspartate aminotransferase, mitochondrial	4	1.11	0.163	0.556
P11310	Medium-chain specific acyl-CoA dehydrogenase, mitochondrial	4	1.11	0.166	0.562
P50225	Sulfotransferase 1A1	1	1.11	NA	NA
P11940	Polyadenylate-binding protein 1	5	1.11	0.029	0.021
P46777	60S ribosomal protein L5	5	1.11	0.057	0.142
Q9UJZ1	Stomatin-like protein 2, mitochondrial	3	1.11	0.100	0.409
Q14165	Malectin	5	1.11	0.099	0.356
P01911	HLA class II histocompatibility antigen, DRB1-15 beta chain	1	1.11	NA	NA
Q9UNH7	Sorting nexin-6	3	1.11	0.084	0.356
P43686	26S protease regulatory subunit 6B	3	1.11	0.107	0.450
P58876	Histone H2B type 1-D	2	1.10	NA	NA
Q86U42	Polyadenylate-binding protein 2	3	1.10	0.041	0.140
Q9UMS4	Pre-mRNA-processing factor 19	1	1.10	NA	NA
P01903	HLA class II histocompatibility antigen, DR alpha chain	5	1.10	0.231	0.697
E9PAV3	Nascent polypeptide-associated complex subunit alpha, muscle-specific form	3	1.10	0.592	0.886
Q9HD20	Manganese-transporting ATPase 13A1	3	1.10	0.226	0.721
O75643	U5 small nuclear ribonucleoprotein 200 kDa helicase	5	1.09	0.044	0.111
Q02878	60S ribosomal protein L6	5	1.09	0.104	0.438
O76003	Glutaredoxin-3	1	1.09	NA	NA
P82909	28S ribosomal protein S36, mitochondrial	4	1.09	0.070	0.295
Q02978	Mitochondrial 2-oxoglutarate/malate carrier protein	2	1.09	NA	NA
P23396	40S ribosomal protein S3	5	1.09	0.034	0.066
O14745	Na(+)/H(+) exchange regulatory cofactor NHE-RF1	1	1.09	NA	NA
P18859	ATP synthase-coupling factor 6, mitochondrial	4	1.09	0.093	0.432
Q15435	Protein phosphatase 1 regulatory subunit 7	1	1.09	NA	NA
O75396	Vesicle-trafficking protein SEC22b	5	1.09	0.165	0.644
Q8IUX7	Adipocyte enhancer-binding protein 1	3	1.09	0.278	0.795
P22307	Non-specific lipid-transfer protein	1	1.08	NA	NA
P62269	40S ribosomal protein S18	5	1.08	0.075	0.342
O00487	26S proteasome non-ATPase regulatory subunit 14	1	1.08	NA	NA
Q96AE4	Far upstream element-binding protein 1	3	1.08	0.084	0.438
P14314	Glucosidase 2 subunit beta	5	1.08	0.118	0.534
P61981	14-3-3 protein gamma	2	1.08	NA	NA
O75947	ATP synthase subunit d, mitochondrial	5	1.08	0.069	0.324
P68871	Hemoglobin subunit beta	5	1.08	0.439	0.869
P08134	Rho-related GTP-binding protein RhoC	4	1.08	0.098	0.493
P11387	DNA topoisomerase 1	2	1.08	NA	NA
P23368	NAD-dependent malic enzyme, mitochondrial	1	1.08	NA	NA

P30048	Thioredoxin-dependent peroxide reductase, mitochondrial	5	1.08	0.194	0.726
P30041	Peroxiredoxin-6	5	1.08	0.059	0.287
P22061	Protein-L-isoaspartate(D-aspartate) O-methyltransferase	1	1.07	NA	NA
Q9BS26	Endoplasmic reticulum resident protein 44	5	1.07	0.172	0.705
P80303	Nucleobindin-2	1	1.07	NA	NA
P54727	UV excision repair protein RAD23 homolog B	4	1.07	0.092	0.509
Q9Y3E1	Hepatoma-derived growth factor-related protein 3	1	1.07	NA	NA
Q8NB37	Parkinson disease 7 domain-containing protein 1	1	1.07	NA	NA
P0CG05	Ig lambda-2 chain C regions	5	1.07	0.326	0.846
P08237	ATP-dependent 6-phosphofructokinase, muscle type	5	1.07	0.166	0.706
Q9UNM6	26S proteasome non-ATPase regulatory subunit 13	2	1.07	NA	NA
P62280	40S ribosomal protein S11	5	1.07	0.134	0.646
P69905	Hemoglobin subunit alpha	5	1.07	0.438	0.888
Q13596	Sorting nexin-1	3	1.07	0.106	0.606
Q92841	Probable ATP-dependent RNA helicase DDX17	5	1.06	0.060	0.354
Q6UVK1	Chondroitin sulfate proteoglycan 4	4	1.06	0.023	0.076
P05198	Eukaryotic translation initiation factor 2 subunit 1	3	1.06	0.093	0.583
Q16401	26S proteasome non-ATPase regulatory subunit 5	1	1.06	NA	NA
P55884	Eukaryotic translation initiation factor 3 subunit B	3	1.06	0.106	0.640
P38117	Electron transfer flavoprotein subunit beta	5	1.06	0.092	0.576
Q9Y3U8	60S ribosomal protein L36	4	1.06	0.049	0.337
P15880	40S ribosomal protein S2	5	1.06	0.053	0.362
Q02543	60S ribosomal protein L18a	4	1.05	0.078	0.548
P50914	60S ribosomal protein L14	5	1.05	0.085	0.579
P26038	Moesin	5	1.05	0.131	0.717
Q16891	Mitochondrial inner membrane protein	5	1.05	0.105	0.661
Q5SSJ5	Heterochromatin protein 1-binding protein 3	5	1.05	0.149	0.755
P35998	26S protease regulatory subunit 7	5	1.05	0.090	0.610
Q8N1G4	Leucine-rich repeat-containing protein 47	4	1.05	0.101	0.661
P63208	S-phase kinase-associated protein 1	1	1.05	NA	NA
Q658Y4	Protein FAM91A1	1	1.05	NA	NA
Q13242	Serine/arginine-rich splicing factor 9	1	1.05	NA	NA
P28331	NADH-ubiquinone oxidoreductase 75 kDa subunit, mitochondrial	3	1.05	0.081	0.632
P31930	Cytochrome b-c1 complex subunit 1, mitochondrial	5	1.05	0.149	0.781
Q15691	Microtubule-associated protein RP/EB family member 1	4	1.04	0.112	0.722
P62753	40S ribosomal protein S6	5	1.04	0.060	0.505
P01023	Alpha-2-macroglobulin	5	1.04	0.232	0.861
Q14157	Ubiquitin-associated protein 2-like	1	1.04	NA	NA
P62917	60S ribosomal protein L8	4	1.04	0.169	0.818
P25705	ATP synthase subunit alpha, mitochondrial	5	1.04	0.076	0.609
P11021	78 kDa glucose-regulated protein	5	1.04	0.074	0.617
P20700	Lamin-B1	5	1.04	0.055	0.509
P11177	Pyruvate dehydrogenase E1 component subunit beta, mitochondrial	5	1.04	0.068	0.595
O43242	26S proteasome non-ATPase regulatory subunit 3	4	1.04	0.093	0.706
P10515	Dihydrolipoyllysine-residue acetyltransferase component of pyruvate dehydrogenase complex, mitochondrial	4	1.04	0.112	0.760
P01876	Ig alpha-1 chain C region	3	1.04	0.628	0.958
P46459	Vesicle-fusing ATPase	3	1.04	0.165	0.849
P27797	Calreticulin	5	1.03	0.155	0.837
O14980	Exportin-1	2	1.03	NA	NA
P78371	T-complex protein 1 subunit beta	5	1.03	0.070	0.659
Q9NSE4	Isoleucine--tRNA ligase, mitochondrial	5	1.03	0.054	0.575
P21912	Succinate dehydrogenase [ubiquinone] iron-sulfur subunit, mitochondrial	5	1.03	0.120	0.803
P36542	ATP synthase subunit gamma, mitochondrial	4	1.03	0.075	0.709
P49327	Fatty acid synthase	2	1.03	NA	NA
P05107	Integrin beta-2	5	1.03	0.345	0.939
P55795	Heterogeneous nuclear ribonucleoprotein H2	1	1.03	NA	NA
Q14697	Neutral alpha-glucosidase AB	5	1.03	0.108	0.818
P15311	Ezrin	5	1.03	0.153	0.879
P84098	60S ribosomal protein L19	1	1.02	NA	NA
O00303	Eukaryotic translation initiation factor 3 subunit F	3	1.02	0.183	0.917
P13073	Cytochrome c oxidase subunit 4 isoform 1, mitochondrial	5	1.02	0.113	0.858
Q13011	Delta(3,5)-Delta(2,4)-dienoyl-CoA isomerase, mitochondrial	5	1.02	0.060	0.742
O60664	Perilipin-3	1	1.02	NA	NA
P61026	Ras-related protein Rab-10	5	1.02	0.115	0.888
Q8IX12	Cell division cycle and apoptosis regulator protein 1	1	1.02	NA	NA
Q8WVC6	Dephospho-CoA kinase domain-containing protein	2	1.01	NA	NA
P32969	60S ribosomal protein L9	5	1.01	0.054	0.823
Q15005	Signal peptidase complex subunit 2	1	1.01	NA	NA
P20674	Cytochrome c oxidase subunit 5A, mitochondrial	4	1.01	0.124	0.932
Q12907	Vesicular integral-membrane protein VIP36	1	1.01	NA	NA
P46781	40S ribosomal protein S9	5	1.01	0.043	0.837
Q08378	Golgin subfamily A member 3	1	1.01	NA	NA
P84103	Serine/arginine-rich splicing factor 3	4	1.01	0.168	0.968
O43837	Isocitrate dehydrogenase [NAD] subunit beta, mitochondrial	2	1.01	NA	NA
Q9UIJ7	GTP:AMP phosphotransferase AK3, mitochondrial	3	1.01	0.045	0.896
Q15008	26S proteasome non-ATPase regulatory subunit 6	2	1.00	NA	NA
Q15121	Astrocytic phosphoprotein PEA-15	2	1.00	NA	NA
Q00325	Phosphate carrier protein, mitochondrial	5	1.00	0.090	0.978
Q7Z6Z7	E3 ubiquitin-protein ligase HUWE1	2	1.00	NA	NA
P36957	Dihydrolipoyllysine-residue succinyltransferase component of 2-oxoglutarate dehydrogenase complex, mitochondrial	5	1.00	0.042	0.981
P52943	Cysteine-rich protein 2	1	1.00	NA	NA
O75489	NADH dehydrogenase [ubiquinone] iron-sulfur protein 3, mitochondrial	5	1.00	0.128	0.971
P17643	5,6-dihydroxyindole-2-carboxylic acid oxidase	5	0.99	0.498	0.991
Q01085	Nucleolysin TIAR	1	0.99	NA	NA
P78344	Eukaryotic translation initiation factor 4 gamma 2	1	0.99	NA	NA
Q9NVS9	Pyridoxine-5'-phosphate oxidase	1	0.99	NA	NA
Q01844	RNA-binding protein EWS	3	0.99	0.235	0.965
Q9Y6M9	NADH dehydrogenase [ubiquinone] 1 beta subcomplex subunit 9	3	0.99	0.270	0.966
Q96QK1	Vacuolar protein sorting-associated protein 35	4	0.99	0.083	0.873
Q13310	Polyadenylate-binding protein 4	1	0.98	NA	NA
P10606	Cytochrome c oxidase subunit 5B, mitochondrial	5	0.98	0.141	0.913
P27708	CAD protein	3	0.98	0.182	0.929
P46977	Dolichyl-diphosphooligosaccharide--protein glycosyltransferase subunit STT3A	2	0.98	NA	NA
O00203	AP-3 complex subunit beta-1	3	0.98	0.108	0.864
P55084	Trifunctional enzyme subunit beta, mitochondrial	5	0.98	0.103	0.843
P08559	Pyruvate dehydrogenase E1 component subunit alpha, somatic form, mitochondrial	2	0.98	NA	NA
P50991	T-complex protein 1 subunit delta	4	0.97	0.017	0.236
P31689	DnaJ homolog subfamily A member 1	3	0.97	0.176	0.895
P24539	ATP synthase F(0) complex subunit B1, mitochondrial	3	0.97	0.016	0.211
O15511	Actin-related protein 2/3 complex subunit 5	3	0.97	0.200	0.889
Q15293	Reticulocalbin-1	5	0.97	0.143	0.829
Q9BPX5	Actin-related protein 2/3 complex subunit 5-like protein	1	0.97	NA	NA
P01860	Ig gamma-3 chain C region	4	0.97	0.174	0.860
P48643	T-complex protein 1 subunit epsilon	5	0.97	0.073	0.667
P62701	40S ribosomal protein S4, X isoform	5	0.97	0.062	0.616
P35606	Coatomer subunit beta'	1	0.97	NA	NA
Q7L5N1	COP9 signalosome complex subunit 6	5	0.96	0.101	0.730
Q14258	E3 ubiquitin/ISG15 ligase TRIM25	3	0.96	0.106	0.754
P62277	40S ribosomal protein S13	5	0.96	0.071	0.608
P42766	60S ribosomal protein L35	1	0.96	NA	NA
Q02218	2-oxoglutarate dehydrogenase, mitochondrial	5	0.96	0.107	0.718
Q13200	26S proteasome non-ATPase regulatory subunit 2	5	0.96	0.130	0.762
P18077	60S ribosomal protein L35a	2	0.96	NA	NA
P60953	Cell division control protein 42 homolog	2	0.96	NA	NA
Q9Y512	Sorting and assembly machinery component 50 homolog	2	0.96	NA	NA
P48735	Isocitrate dehydrogenase [NADP], mitochondrial	5	0.96	0.135	0.758
P11279	Lysosome-associated membrane glycoprotein 1	3	0.96	0.490	0.935
P01834	Ig kappa chain C region	5	0.96	0.326	0.896
Q7L576	Cytoplasmic FMR1-interacting protein 1	1	0.96	NA	NA
O75165	DnaJ homolog subfamily C member 13	2	0.95	NA	NA
P35613	Basigin	3	0.95	0.300	0.890
P40227	T-complex protein 1 subunit zeta	5	0.95	0.069	0.527
P49411	Elongation factor Tu, mitochondrial	5	0.95	0.243	0.853
Q6IAA8	Ragulator complex protein LAMTOR1	1	0.95	NA	NA
Q8NBS9	Thioredoxin domain-containing protein 5	4	0.95	0.129	0.724
P42858	Huntingtin	1	0.95	NA	NA
Q13177	Serine/threonine-protein kinase PAK 2	1	0.95	NA	NA
Q8NDH3	Probable aminopeptidase NPEPL1	1	0.95	NA	NA
Q14847	LIM and SH3 domain protein 1	3	0.95	0.164	0.778
P16403	Histone H1.2	2	0.95	NA	NA
Q92973	Transportin-1	2	0.95	NA	NA
P14927	Cytochrome b-c1 complex subunit 7	4	0.95	0.097	0.604
O00232	26S proteasome non-ATPase regulatory subunit 12	1	0.95	NA	NA
Q99653	Calcineurin B homologous protein 1	2	0.95	NA	NA
P39656	Dolichyl-diphosphooligosaccharide--protein glycosyltransferase 48 kDa subunit	5	0.94	0.093	0.551
O95479	GDH/6PGL endoplasmic bifunctional protein	1	0.94	NA	NA
Q92896	Golgi apparatus protein 1	2	0.94	NA	NA
Q96I24	Far upstream element-binding protein 3	2	0.94	NA	NA
P49748	Very long-chain specific acyl-CoA dehydrogenase, mitochondrial	4	0.94	0.104	0.566
O60262	Guanine nucleotide-binding protein G(I)/G(S)/G(O) subunit gamma-7	1	0.93	NA	NA
O60749	Sorting nexin-2	2	0.93	NA	NA
Q14980	Nuclear mitotic apparatus protein 1	5	0.93	0.023	0.039
Q9Y4L1	Hypoxia up-regulated protein 1	5	0.93	0.148	0.665
P26196	Probable ATP-dependent RNA helicase DDX6	4	0.93	0.053	0.268
P62987	Ubiquitin-60S ribosomal protein L40	3	0.93	0.382	0.869
P35573	Glycogen debranching enzyme	1	0.93	NA	NA
P47756	F-actin-capping protein subunit beta	5	0.93	0.088	0.453
O15173	Membrane-associated progesterone receptor component 2	4	0.93	0.117	0.566
Q92597	Protein NDRG1	3	0.93	0.128	0.610
O75964	ATP synthase subunit g, mitochondrial	5	0.93	0.073	0.345
P61758	Prefoldin subunit 3	1	0.92	NA	NA
P13667	Protein disulfide-isomerase A4	5	0.92	0.135	0.559
Q92499	ATP-dependent RNA helicase DDX1	4	0.92	0.127	0.544
P23919	Thymidylate kinase	1	0.92	NA	NA
Q9UHQ9	NADH-cytochrome b5 reductase 1	5	0.92	0.065	0.249
O43760	Synaptogyrin-2	1	0.92	NA	NA
P56556	NADH dehydrogenase [ubiquinone] 1 alpha subcomplex subunit 6	2	0.91	NA	NA
Q3ZCQ8	Mitochondrial import inner membrane translocase subunit TIM50	2	0.91	NA	NA
P14625	Endoplasmin	5	0.91	0.125	0.490
P06703	Protein S100-A6	2	0.91	NA	NA
P48444	Coatomer subunit delta	2	0.91	NA	NA
P48047	ATP synthase subunit O, mitochondrial	5	0.91	0.115	0.443
P40763	Signal transducer and activator of transcription 3	4	0.91	0.106	0.423
P17931	Galectin-3	5	0.91	0.112	0.428
P46109	Crk-like protein	1	0.90	NA	NA
P40939	Trifunctional enzyme subunit alpha, mitochondrial	5	0.90	0.103	0.378
O15260	Surfeit locus protein 4	1	0.90	NA	NA
P60228	Eukaryotic translation initiation factor 3 subunit E	1	0.90	NA	NA
P30626	Sorcin	1	0.90	NA	NA
Q14683	Structural maintenance of chromosomes protein 1A	4	0.90	0.056	0.149
P47985	Cytochrome b-c1 complex subunit Rieske, mitochondrial	3	0.89	0.066	0.233
P51970	NADH dehydrogenase [ubiquinone] 1 alpha subcomplex subunit 8	1	0.89	NA	NA
P63261	Actin, cytoplasmic 2	2	0.89	NA	NA
Q6P2Q9	Pre-mRNA-processing-splicing factor 8	3	0.89	0.087	0.315
P04839	Cytochrome b-245 heavy chain	1	0.89	NA	NA
P49368	T-complex protein 1 subunit gamma	5	0.89	0.077	0.196
P04632	Calpain small subunit 1	5	0.89	0.067	0.148
P50990	T-complex protein 1 subunit theta	5	0.89	0.081	0.216
Q15942	Zyxin	4	0.89	0.184	0.562
Q93050	V-type proton ATPase 116 kDa subunit a isoform 1	5	0.89	0.142	0.443
Q7KZF4	Staphylococcal nuclease domain-containing protein 1	5	0.89	0.058	0.106
O75306	NADH dehydrogenase [ubiquinone] iron-sulfur protein 2, mitochondrial	4	0.88	0.166	0.506
Q9UGP8	Translocation protein SEC63 homolog	1	0.88	NA	NA
P40429	60S ribosomal protein L13a	5	0.88	0.132	0.384
Q9UBS4	DnaJ homolog subfamily B member 11	3	0.88	0.097	0.308
P48681	Nestin	3	0.88	0.369	0.754
Q9P0L0	Vesicle-associated membrane protein-associated protein A	3	0.88	0.060	0.155

Q9Y265	RuvB-like 1	2	0.87	NA	NA
Q9UPN3	Microtubule-actin cross-linking factor 1, isoforms 1/2/3/5	4	0.87	0.132	0.379
P43304	Glycerol-3-phosphate dehydrogenase, mitochondrial	5	0.87	0.162	0.447
P60891	Ribose-phosphate pyrophosphokinase 1	1	0.87	NA	NA
P47813	Eukaryotic translation initiation factor 1A, X-chromosomal	1	0.87	NA	NA
O60763	General vesicular transport factor p115	2	0.87	NA	NA
Q99460	26S proteasome non-ATPase regulatory subunit 1	2	0.87	NA	NA
P01042	Kininogen-1	3	0.87	0.574	0.828
Q99733	Nucleosome assembly protein 1-like 4	4	0.86	0.102	0.240
Q99584	Protein S100-A13	5	0.86	0.160	0.400
Q9P0J0	NADH dehydrogenase [ubiquinone] 1 alpha subcomplex subunit 13	1	0.86	NA	NA
P24752	Acetyl-CoA acetyltransferase, mitochondrial	5	0.86	0.089	0.158
O15455	Toll-like receptor 3	1	0.86	NA	NA
P25685	DnaJ homolog subfamily B member 1	1	0.86	NA	NA
P33176	Kinesin-1 heavy chain	1	0.86	NA	NA
P30153	Serine/threonine-protein phosphatase 2A 65 kDa regulatory subunit A alpha isoform	4	0.86	0.153	0.386
Q99714	3-hydroxyacyl-CoA dehydrogenase type-2	4	0.85	0.158	0.391
Q99832	T-complex protein 1 subunit eta	5	0.85	0.114	0.236
Q9Y376	Calcium-binding protein 39	1	0.85	NA	NA
P55072	Transitional endoplasmic reticulum ATPase	5	0.85	0.122	0.244
P51798	H(+)/Cl(-) exchange transporter 7	1	0.84	NA	NA
P50502	Hsc70-interacting protein	5	0.84	0.136	0.278
P42765	3-ketoacyl-CoA thiolase, mitochondrial	5	0.84	0.064	0.053
P02461	Collagen alpha-1(III) chain	1	0.84	NA	NA
P17987	T-complex protein 1 subunit alpha	5	0.84	0.179	0.380
Q16352	Alpha-internexin	1	0.84	NA	NA
P57053	Histone H2B type F-S	2	0.83	NA	NA
Q27J81	Inverted formin-2	4	0.83	0.067	0.071
P07384	Calpain-1 catalytic subunit	4	0.83	0.134	0.264
Q9P2R3	Ankyrin repeat and FYVE domain-containing protein 1	2	0.83	NA	NA
P09496	Clathrin light chain A	4	0.83	0.077	0.095
P30622	CAP-Gly domain-containing linker protein 1	1	0.83	NA	NA
Q9Y230	RuvB-like 2	4	0.83	0.122	0.224
P53621	Coatomer subunit alpha	4	0.83	0.090	0.129
Q9H4M9	EH domain-containing protein 1	3	0.83	0.197	0.440
P67936	Tropomyosin alpha-4 chain	5	0.83	0.101	0.132
Q14108	Lysosome membrane protein 2	5	0.83	0.188	0.365
O43678	NADH dehydrogenase [ubiquinone] 1 alpha subcomplex subunit 2	1	0.82	NA	NA
P31943	Heterogeneous nuclear ribonucleoprotein H	2	0.82	NA	NA
P55209	Nucleosome assembly protein 1-like 1	3	0.82	0.141	0.301
P51812	Ribosomal protein S6 kinase alpha-3	3	0.82	0.228	0.478
P17858	ATP-dependent 6-phosphofructokinase, liver type	5	0.82	0.133	0.211
O75340	Programmed cell death protein 6	2	0.82	NA	NA
Q6UXV4	Apolipoprotein O-like	1	0.82	NA	NA
Q9ULA0	Aspartyl aminopeptidase	2	0.82	NA	NA
Q9UNF0	Protein kinase C and casein kinase substrate in neurons protein 2	1	0.82	NA	NA
Q13561	Dynactin subunit 2	5	0.81	0.081	0.064
P00403	Cytochrome c oxidase subunit 2	3	0.81	0.279	0.534
P62244	40S ribosomal protein S15a	5	0.81	0.071	0.043
Q9H3Z4	DnaJ homolog subfamily C member 5	1	0.81	NA	NA
P00488	Coagulation factor XIII A chain	1	0.81	NA	NA
P61225	Ras-related protein Rap-2b	3	0.81	0.173	0.348
P42025	Beta-centractin	1	0.81	NA	NA
P27816	Microtubule-associated protein 4	5	0.81	0.259	0.452
Q9NTJ5	Phosphatidylinositide phosphatase SAC1	3	0.81	0.145	0.274
P51532	Transcription activator BRG1	1	0.81	NA	NA
P16435	NADPH--cytochrome P450 reductase	3	0.80	0.084	0.123
O95831	Apoptosis-inducing factor 1, mitochondrial	2	0.80	NA	NA
P50402	Emerin	5	0.80	0.145	0.204
Q9NV70	Exocyst complex component 1	1	0.80	NA	NA
P54920	Alpha-soluble NSF attachment protein	5	0.80	0.149	0.213
Q9Y4F1	FERM, RhoGEF and pleckstrin domain-containing protein 1	2	0.80	NA	NA
P50151	Guanine nucleotide-binding protein G(I)/G(S)/G(O) subunit gamma-10	1	0.80	NA	NA
Q14558	Phosphoribosyl pyrophosphate synthase-associated protein 1	2	0.80	NA	NA
Q86VB7	Scavenger receptor cysteine-rich type 1 protein M130	1	0.80	NA	NA
P21589	5'-nucleotidase	2	0.80	NA	NA
P21964	Catechol O-methyltransferase	1	0.79	NA	NA
Q10567	AP-1 complex subunit beta-1	3	0.79	0.169	0.306
P02545	Prelamin-A/C	5	0.79	0.138	0.168
Q12913	Receptor-type tyrosine-protein phosphatase eta	1	0.79	NA	NA
O75914	Serine/threonine-protein kinase PAK 3	1	0.79	NA	NA
P49419	Alpha-aminoadipic semialdehyde dehydrogenase	2	0.79	NA	NA
O94826	Mitochondrial import receptor subunit TOM70	2	0.79	NA	NA
P09382	Galectin-1	5	0.79	0.114	0.104
Q14011	Cold-inducible RNA-binding protein	1	0.79	NA	NA
Q8IZP0	Abl interactor 1	1	0.79	NA	NA
P29590	Protein PML	5	0.78	0.143	0.162
Q86WV6	Stimulator of interferon genes protein	2	0.78	NA	NA
P35637	RNA-binding protein FUS	5	0.78	0.281	0.427
P37235	Hippocalcin-like protein 1	2	0.78	NA	NA
Q93009	Ubiquitin carboxyl-terminal hydrolase 7	1	0.78	NA	NA
Q02252	Methylmalonate-semialdehyde dehydrogenase [acylating], mitochondrial	3	0.78	0.075	0.079
Q9H444	Charged multivesicular body protein 4b	1	0.78	NA	NA
Q05193	Dynamin-1	1	0.78	NA	NA
Q9UHD8	Septin-9	5	0.77	0.062	0.015
P31942	Heterogeneous nuclear ribonucleoprotein H3	1	0.77	NA	NA
O94979	Protein transport protein Sec31A	5	0.77	0.084	0.038
Q13228	Selenium-binding protein 1	4	0.77	0.027	0.002
Q16851	UTP--glucose-1-phosphate uridylyltransferase	2	0.77	NA	NA
P51648	Fatty aldehyde dehydrogenase	1	0.77	NA	NA
Q8WXH0	Nesprin-2	2	0.76	NA	NA
P55735	Protein SEC13 homolog	1	0.76	NA	NA
P04843	Dolichyl-diphosphooligosaccharide--protein glycosyltransferase subunit 1	5	0.76	0.112	0.073
O00571	ATP-dependent RNA helicase DDX3X	3	0.76	0.187	0.285
P35813	Protein phosphatase 1A	1	0.76	NA	NA
Q99829	Copine-1	1	0.76	NA	NA
Q9HBL7	Plasminogen receptor (KT)	1	0.76	NA	NA
P51148	Ras-related protein Rab-5C	1	0.76	NA	NA
Q16270	Insulin-like growth factor-binding protein 7	4	0.76	0.210	0.275
P63092	Guanine nucleotide-binding protein G(s) subunit alpha isoforms short	1	0.76	NA	NA
Q9GZR7	ATP-dependent RNA helicase DDX24	1	0.75	NA	NA
Q13283	Ras GTPase-activating protein-binding protein 1	2	0.75	NA	NA
O60271	C-Jun-amino-terminal kinase-interacting protein 4	1	0.75	NA	NA
P04792	Heat shock protein beta-1	5	0.75	0.138	0.107
P06727	Apolipoprotein A-IV	5	0.75	0.293	0.384
Q9Y6N5	Sulfide:quinone oxidoreductase, mitochondrial	5	0.75	0.260	0.331
Q53GQ0	Estradiol 17-beta-dehydrogenase 12	5	0.75	0.117	0.070
Q15075	Early endosome antigen 1	2	0.75	NA	NA
Q9BSD7	Cancer-related nucleoside-triphosphatase	1	0.75	NA	NA
Q12797	Aspartyl/asparaginyl beta-hydroxylase	5	0.74	0.068	0.012
P04844	Dolichyl-diphosphooligosaccharide--protein glycosyltransferase subunit 2	4	0.74	0.135	0.114
P49458	Signal recognition particle 9 kDa protein	3	0.74	0.217	0.302
A0FGR8	Extended synaptotagmin-2	2	0.74	NA	NA
P55060	Exportin-2	4	0.74	0.037	0.004
Q9P0K7	Ankycorbin	1	0.74	NA	NA
Q9UQE7	Structural maintenance of chromosomes protein 3	3	0.74	0.067	0.045
O14735	CDP-diacylglycerol--inositol 3-phosphatidyltransferase	2	0.74	NA	NA
Q07065	Cytoskeleton-associated protein 4	5	0.73	0.086	0.023
Q16718	NADH dehydrogenase [ubiquinone] 1 alpha subcomplex subunit 5	1	0.73	NA	NA
P00367	Glutamate dehydrogenase 1, mitochondrial	5	0.72	0.043	0.002
Q9UJS0	Calcium-binding mitochondrial carrier protein Aralar2	1	0.72	NA	NA
P02452	Collagen alpha-1(I) chain	3	0.72	0.588	0.629
P53992	Protein transport protein Sec24C	1	0.72	NA	NA
P35221	Catenin alpha-1	3	0.72	0.060	0.031
P49257	Protein ERGIC-53	5	0.71	0.132	0.062
P23786	Carnitine O-palmitoyltransferase 2, mitochondrial	1	0.71	NA	NA
Q14203	Dynactin subunit 1	5	0.71	0.087	0.018
Q8TEX9	Importin-4	1	0.71	NA	NA
Q99442	Translocation protein SEC62	3	0.71	0.155	0.155
P10321	HLA class I histocompatibility antigen, Cw-7 alpha chain	2	0.70	NA	NA
Q9NP97	Dynein light chain roadblock-type 1	1	0.70	NA	NA
O75323	Protein NipSnap homolog 2	1	0.70	NA	NA
P00740	Coagulation factor IX	1	0.70	NA	NA
Q6NUM9	All-trans-retinol 13,14-reductase	1	0.70	NA	NA
Q9Y277	Voltage-dependent anion-selective channel protein 3	3	0.70	0.157	0.148
P38919	Eukaryotic initiation factor 4A-III	1	0.70	NA	NA
Q9HDC9	Adipocyte plasma membrane-associated protein	5	0.69	0.180	0.111
P08603	Complement factor H	3	0.69	0.427	0.482
Q14956	Transmembrane glycoprotein NMB	5	0.69	0.498	0.494
P67870	Casein kinase II subunit beta	3	0.69	0.108	0.074
O43707	Alpha-actinin-4	5	0.69	0.123	0.038
Q71DI3	Histone H3.2	1	0.69	NA	NA
P54709	Sodium/potassium-transporting ATPase subunit beta-3	5	0.69	0.201	0.133
P29966	Myristoylated alanine-rich C-kinase substrate	5	0.69	0.160	0.078
Q9Y639	Neuroplastin	2	0.68	NA	NA
P16615	Sarcoplasmic/endoplasmic reticulum calcium ATPase 2	5	0.68	0.106	0.022
P60709	Actin, cytoplasmic 1	3	0.68	0.076	0.037
Q9Y3I0	tRNA-splicing ligase RtcB homolog	5	0.68	0.046	0.001
P50995	Annexin A11	5	0.67	0.151	0.060
Q15165	Serum paraoxonase/arylesterase 2	1	0.67	NA	NA
P35580	Myosin-10	5	0.67	0.147	0.051
P0C0S8	Histone H2A type 1	4	0.66	0.127	0.048
Q14204	Cytoplasmic dynein 1 heavy chain 1	5	0.66	0.090	0.010
O75915	PRA1 family protein 3	4	0.66	0.139	0.060
P42566	Epidermal growth factor receptor substrate 15	1	0.66	NA	NA
P04217	Alpha-1B-glycoprotein	5	0.66	0.267	0.197
Q16795	NADH dehydrogenase [ubiquinone] 1 alpha subcomplex subunit 9, mitochondrial	4	0.66	0.178	0.102
P43307	Translocon-associated protein subunit alpha	2	0.66	NA	NA
P46939	Utrophin	4	0.65	0.139	0.056
Q96D15	Reticulocalbin-3	1	0.65	NA	NA
P12235	ADP/ATP translocase 1	2	0.65	NA	NA
O75477	Erlin-1	1	0.65	NA	NA
Q01813	ATP-dependent 6-phosphofructokinase, platelet type	2	0.65	NA	NA
O95297	Myelin protein zero-like protein 1	1	0.65	NA	NA
P04114	Apolipoprotein B-100	3	0.65	0.534	0.502
P08311	Cathepsin G	2	0.64	NA	NA
Q16695	Histone H3.1t	4	0.64	0.171	0.082
P62805	Histone H4	5	0.64	0.135	0.030
Q96S52	GPI transamidase component PIG-S	1	0.64	NA	NA
Q15149	Plectin	5	0.64	0.062	0.002
Q969X5	Endoplasmic reticulum-Golgi intermediate compartment protein 1	3	0.64	0.174	0.122
P07437	Tubulin beta chain	4	0.64	0.102	0.022
Q15582	Transforming growth factor-beta-induced protein ig-h3	4	0.64	0.330	0.264
P14209	CD99 antigen	1	0.63	NA	NA
O00264	Membrane-associated progesterone receptor component 1	5	0.63	0.133	0.026
P08107	Heat shock 70 kDa protein 1A/1B	5	0.63	0.102	0.011
O60504	Vinexin	1	0.63	NA	NA
Q9BRX8	Redox-regulatory protein FAM213A	3	0.63	0.143	0.083
Q8NFW8	N-acylneuraminate cytidylyltransferase	1	0.63	NA	NA
Q14764	Major vault protein	5	0.62	0.068	0.002
O43865	Putative adenosylhomocysteinase 2	3	0.62	0.135	0.074
P61163	Alpha-centractin	2	0.62	NA	NA
P00390	Glutathione reductase, mitochondrial	3	0.62	0.189	0.128
Q9H223	EH domain-containing protein 4	2	0.62	NA	NA
O60313	Dynamin-like 120 kDa protein, mitochondrial	5	0.62	0.087	0.005
Q9Y2J2	Band 4.1-like protein 3	2	0.62	NA	NA
Q15019	Septin-2	5	0.62	0.084	0.005

Table S16-Average Metastatic Tumors

Q9BQE3	Tubulin alpha-1C chain	3	0.61	0.222	0.158
P17655	Calpain-2 catalytic subunit	2	0.61	NA	NA
Q9BZZ2	Sialoadhesin	1	0.61	NA	NA
Q00610	Clathrin heavy chain 1	5	0.61	0.124	0.016
Q92905	COP9 signalosome complex subunit 5	2	0.61	NA	NA
P05362	Intercellular adhesion molecule 1	4	0.61	0.157	0.050
Q9NVA2	Septin-11	3	0.60	0.178	0.106
Q05682	Caldesmon	5	0.60	0.235	0.099
Q13418	Integrin-linked protein kinase	2	0.60	NA	NA
Q9NQC3	Reticulon-4	5	0.60	0.051	0.001
P09543	2',3'-cyclic-nucleotide 3'-phosphodiesterase	5	0.60	0.106	0.009
P13861	cAMP-dependent protein kinase type II-alpha regulatory subunit	5	0.60	0.037	0.000
Q96CW1	AP-2 complex subunit mu	2	0.60	NA	NA
O95782	AP-2 complex subunit alpha-1	5	0.59	0.125	0.014
Q6NUK1	Calcium-binding mitochondrial carrier protein SCaMC-1	4	0.59	0.104	0.015
Q9C0E8	Protein lunapark	2	0.59	NA	NA
P02776	Platelet factor 4	1	0.58	NA	NA
P47755	F-actin-capping protein subunit alpha-2	2	0.58	NA	NA
P12111	Collagen alpha-3(VI) chain	5	0.58	0.198	0.052
Q71U36	Tubulin alpha-1A chain	1	0.58	NA	NA
Q9UBI6	Guanine nucleotide-binding protein G(I)/G(S)/G(O) subunit gamma-12	1	0.58	NA	NA
P42167	Lamina-associated polypeptide 2, isoforms beta/gamma	4	0.58	0.139	0.029
O75131	Copine-3	5	0.58	0.123	0.011
Q8NC56	LEM domain-containing protein 2	1	0.57	NA	NA
Q14344	Guanine nucleotide-binding protein subunit alpha-13	4	0.57	0.062	0.003
Q9NYL9	Tropomodulin-3	1	0.57	NA	NA
P28288	ATP-binding cassette sub-family D member 3	1	0.57	NA	NA
P00352	Retinal dehydrogenase 1	5	0.57	0.271	0.106
Q9P0M6	Core histone macro-H2A.2	2	0.57	NA	NA
O60716	Catenin delta-1	5	0.57	0.128	0.011
P68363	Tubulin alpha-1B chain	1	0.57	NA	NA
O94919	Endonuclease domain-containing 1 protein	3	0.56	0.147	0.060
P09619	Platelet-derived growth factor receptor beta	1	0.56	NA	NA
P09497	Clathrin light chain B	4	0.56	0.284	0.131
Q9NZ01	Very-long-chain enoyl-CoA reductase	1	0.55	NA	NA
Q8WUM4	Programmed cell death 6-interacting protein	5	0.55	0.110	0.006
P26583	High mobility group protein B2	5	0.55	0.170	0.025
Q02880	DNA topoisomerase 2-beta	1	0.55	NA	NA
Q12965	Unconventional myosin-Ie	1	0.55	NA	NA
P11498	Pyruvate carboxylase, mitochondrial	1	0.55	NA	NA
O14950	Myosin regulatory light chain 12B	5	0.54	0.141	0.012
Q9BSJ8	Extended synaptotagmin-1	5	0.54	0.088	0.002
P05091	Aldehyde dehydrogenase, mitochondrial	5	0.54	0.134	0.010
P04040	Catalase	5	0.54	0.306	0.115
P09936	Ubiquitin carboxyl-terminal hydrolase isozyme L1	5	0.54	0.244	0.064
P11413	Glucose-6-phosphate 1-dehydrogenase	3	0.54	0.253	0.134
Q16181	Septin-7	5	0.54	0.057	0.000
O60814	Histone H2B type 1-K	1	0.53	NA	NA
P17612	cAMP-dependent protein kinase catalytic subunit alpha	4	0.53	0.089	0.006
Q14254	Flotillin-2	3	0.53	0.234	0.114
P12109	Collagen alpha-1(VI) chain	5	0.53	0.216	0.043
Q09666	Neuroblast differentiation-associated protein AHNAK	5	0.53	0.121	0.006
Q9NVD7	Alpha-parvin	1	0.53	NA	NA
P60033	CD81 antigen	3	0.53	0.065	0.010
P07357	Complement component C8 alpha chain	1	0.53	NA	NA
P12110	Collagen alpha-2(VI) chain	5	0.52	0.213	0.039
P23634	Plasma membrane calcium-transporting ATPase 4	4	0.52	0.118	0.012
O94832	Unconventional myosin-Id	4	0.52	0.193	0.041
Q9Y490	Talin-1	5	0.52	0.085	0.001
P15144	Aminopeptidase N	1	0.52	NA	NA
P08133	Annexin A6	5	0.51	0.135	0.008
P01024	Complement C3	5	0.51	0.290	0.082
Q03252	Lamin-B2	5	0.51	0.075	0.001
O43175	D-3-phosphoglycerate dehydrogenase	2	0.51	NA	NA
Q15413	Ryanodine receptor 3	1	0.51	NA	NA
P35222	Catenin beta-1	5	0.51	0.242	0.048
P08571	Monocyte differentiation antigen CD14	5	0.50	0.249	0.052
Q00577	Transcriptional activator protein Pur-alpha	3	0.50	0.202	0.075
P35579	Myosin-9	5	0.50	0.169	0.015
P00387	NADH-cytochrome b5 reductase 3	5	0.49	0.141	0.007
P12814	Alpha-actinin-1	5	0.49	0.068	0.000
Q9UHG3	Prenylcysteine oxidase 1	5	0.49	0.128	0.005
P60660	Myosin light polypeptide 6	5	0.49	0.097	0.002
Q6DD88	Atlastin-3	5	0.49	0.157	0.010
Q15836	Vesicle-associated membrane protein 3	3	0.49	0.102	0.020
Q16563	Synaptophysin-like protein 1	2	0.49	NA	NA
P0C0S5	Histone H2A.Z	5	0.49	0.082	0.001
Q08722	Leukocyte surface antigen CD47	2	0.49	NA	NA
P20073	Annexin A7	3	0.48	0.158	0.043
P04216	Thy-1 membrane glycoprotein	4	0.48	0.100	0.005
P35611	Alpha-adducin	3	0.47	0.055	0.005
P32119	Peroxiredoxin-2	5	0.47	0.096	0.002
O75746	Calcium-binding mitochondrial carrier protein Aralar1	1	0.47	NA	NA
Q8NEV1	Casein kinase II subunit alpha 3	1	0.47	NA	NA
P50570	Dynamin-2	1	0.47	NA	NA
Q9NZN4	EH domain-containing protein 2	4	0.47	0.154	0.016
Q15404	Ras suppressor protein 1	1	0.46	NA	NA
P41222	Prostaglandin-H2 D-isomerase	2	0.46	NA	NA
O94911	ATP-binding cassette sub-family A member 8	2	0.46	NA	NA
O75955	Flotillin-1	2	0.46	NA	NA
Q14192	Four and a half LIM domains protein 2	1	0.46	NA	NA
P02751	Fibronectin	5	0.45	0.452	0.156
P00918	Carbonic anhydrase 2	2	0.45	NA	NA
P04196	Histidine-rich glycoprotein	4	0.44	0.507	0.205
P07358	Complement component C8 beta chain	3	0.43	0.462	0.212
Q9BZQ8	Protein Niban	2	0.43	NA	NA
Q5JWF2	Guanine nucleotide-binding protein G(s) subunit alpha isoforms XLas	1	0.43	NA	NA
O94760	N(G),N(G)-dimethylarginine dimethylaminohydrolase 1	1	0.43	NA	NA
Q9Y4G6	Talin-2	1	0.43	NA	NA
Q03591	Complement factor H-related protein 1	1	0.42	NA	NA
Q5JRA6	Melanoma inhibitory activity protein 3	1	0.42	NA	NA
Q15041	ADP-ribosylation factor-like protein 6-interacting protein 1	1	0.42	NA	NA
Q9UEY8	Gamma-adducin	1	0.41	NA	NA
Q13557	Calcium/calmodulin-dependent protein kinase type II subunit delta	1	0.41	NA	NA
Q9BT22	Chitobiosyldiphosphodolichol beta-mannosyltransferase	1	0.41	NA	NA
Q9BTV4	Transmembrane protein 43	3	0.41	0.331	0.112
P23229	Integrin alpha-6	1	0.40	NA	NA
P23142	Fibulin-1	1	0.40	NA	NA
P26006	Integrin alpha-3	2	0.40	NA	NA
P00747	Plasminogen	5	0.39	0.442	0.103
P29992	Guanine nucleotide-binding protein subunit alpha-11	3	0.39	0.315	0.097
Q14118	Dystroglycan	1	0.37	NA	NA
Q9NR12	PDZ and LIM domain protein 7	1	0.37	NA	NA
P26447	Protein S100-A4	5	0.37	0.524	0.129
Q9BUF5	Tubulin beta-6 chain	2	0.37	NA	NA
P22413	Ectonucleotide pyrophosphatase/phosphodiesterase family member 1	3	0.36	0.324	0.089
Q96HC4	PDZ and LIM domain protein 5	2	0.36	NA	NA
Q96HN2	Putative adenosylhomocysteinase 3	1	0.36	NA	NA
P06756	Integrin alpha-V	1	0.36	NA	NA
P63010	AP-2 complex subunit beta	1	0.36	NA	NA
Q7Z7G0	Target of Nesh-SH3	2	0.35	NA	NA
Q8IWA5	Choline transporter-like protein 2	1	0.35	NA	NA
P54289	Voltage-dependent calcium channel subunit alpha-2/delta-1	1	0.35	NA	NA
Q93052	Lipoma-preferred partner	1	0.35	NA	NA
Q9NY15	Stabilin-1	2	0.33	NA	NA
P10301	Ras-related protein R-Ras	1	0.33	NA	NA
P59768	Guanine nucleotide-binding protein G(I)/G(S)/G(O) subunit gamma-2	1	0.33	NA	NA
P02679	Fibrinogen gamma chain	5	0.33	0.415	0.054
P01600	Ig kappa chain V-I region Hau	1	0.32	NA	NA
P02654	Apolipoprotein C-I	5	0.31	0.458	0.065
O14786	Neuropilin-1	2	0.31	NA	NA
P02749	Beta-2-glycoprotein 1	4	0.30	0.512	0.102
Q92522	Histone H1x	2	0.30	NA	NA
P02675	Fibrinogen beta chain	5	0.29	0.447	0.052
P14207	Folate receptor beta	1	0.29	NA	NA
Q13361	Microfibrillar-associated protein 5	2	0.29	NA	NA
P05026	Sodium/potassium-transporting ATPase subunit beta-1	1	0.28	NA	NA
Q08431	Lactadherin	2	0.28	NA	NA
P61626	Lysozyme C	5	0.27	0.579	0.084
P0DJI8	Serum amyloid A-1 protein	1	0.26	NA	NA
P60201	Myelin proteolipid protein	2	0.26	NA	NA
P05164	Myeloperoxidase	5	0.26	0.628	0.099
A8MTJ3	Guanine nucleotide-binding protein G(t) subunit alpha-3	1	0.26	NA	NA
Q96CX2	BTB/POZ domain-containing protein KCTD12	3	0.24	0.352	0.055
Q9Y240	C-type lectin domain family 11 member A	1	0.24	NA	NA
Q6UXB8	Peptidase inhibitor 16	2	0.23	NA	NA
P14555	Phospholipase A2, membrane associated	2	0.21	NA	NA
P10643	Complement component C7	3	0.21	0.421	0.066
P02656	Apolipoprotein C-III	1	0.19	NA	NA
P05787	Keratin, type II cytoskeletal 8	2	0.19	NA	NA
P14136	Glial fibrillary acidic protein	2	0.18	NA	NA
Q02224	Centromere-associated protein E	1	0.18	NA	NA
P12277	Creatine kinase B-type	1	0.17	NA	NA
P62736	Actin, aortic smooth muscle	1	0.16	NA	NA
Q8N5C1	Protein FAM26E	1	0.15	NA	NA
Q63ZY3	KN motif and ankyrin repeat domain-containing protein 2	1	0.15	NA	NA
Q92777	Synapsin-2	2	0.15	NA	NA
Q6UWY5	Olfactomedin-like protein 1	2	0.14	NA	NA
Q9NRN5	Olfactomedin-like protein 3	1	0.13	NA	NA
Q14767	Latent-transforming growth factor beta-binding protein 2	1	0.11	NA	NA
P35243	Recoverin	1	0.11	NA	NA
P21246	Pleiotrophin	2	0.10	NA	NA
P35556	Fibrillin-2	1	0.10	NA	NA
Q9HCJ6	Synaptic vesicle membrane protein VAT-1 homolog-like	1	0.10	NA	NA
P03973	Antileukoproteinase	2	0.09	NA	NA
Q07954	Prolow-density lipoprotein receptor-related protein 1	5	0.46	0.182	1.3E-02
P68371	Tubulin beta-4B chain	5	0.45	0.060	1.8E-04
O94905	Erlin-2	5	0.44	0.163	7.7E-03
P05023	Sodium/potassium-transporting ATPase subunit alpha-1	5	0.44	0.112	1.9E-03
P05556	Integrin beta-1	5	0.44	0.112	1.8E-03
P04899	Guanine nucleotide-binding protein G(i) subunit alpha-2	5	0.43	0.119	2.0E-03
Q9NZM1	Myoferlin	5	0.42	0.264	3.1E-02
Q9HBL0	Tensin-1	4	0.42	0.267	4.8E-02
P13987	CD59 glycoprotein	4	0.42	0.237	3.5E-02
P06396	Gelsolin	5	0.42	0.184	8.9E-03
P0C0L5	Complement C4-B	4	0.41	0.155	1.1E-02
P07099	Epoxide hydrolase 1	5	0.41	0.151	4.1E-03
P43121	Cell surface glycoprotein MUC18	5	0.40	0.051	5.7E-05
P06899	Histone H2B type 1-J	5	0.40	0.175	6.5E-03
O95865	N(G),N(G)-dimethylarginine dimethylaminohydrolase 2	4	0.40	0.170	1.2E-02
P00738	Haptoglobin	5	0.40	0.112	1.2E-03
P07305	Histone H1.0	4	0.39	0.260	3.6E-02
P13671	Complement component C6	4	0.39	0.217	2.2E-02
P62873	Guanine nucleotide-binding protein G(I)/G(S)/G(T) subunit beta-1	3	0.37	0.049	2.4E-03
O00159	Unconventional myosin-Ic	5	0.36	0.061	7.7E-05
P18206	Vinculin	5	0.36	0.036	8.6E-06
P07942	Laminin subunit beta-1	5	0.35	0.228	1.0E-02

Q01082	Spectrin beta chain, non-erythrocytic 1	5	0.35	0.125	1.1E-03
Q13813	Spectrin alpha chain, non-erythrocytic 1	5	0.35	0.109	6.2E-04
P00450	Ceruloplasmin	5	0.34	0.256	1.4E-02
O00468	Agrin	4	0.34	0.288	3.4E-02
O75369	Filamin-B	5	0.33	0.143	1.5E-03
P21333	Filamin-A	5	0.32	0.106	4.5E-04
P01011	Alpha-1-antichymotrypsin	5	0.31	0.339	2.7E-02
P39060	Collagen alpha-1(XVIII) chain	5	0.31	0.231	7.0E-03
O15230	Laminin subunit alpha-5	5	0.30	0.208	4.6E-03
P07355	Annexin A2	5	0.30	0.137	9.4E-04
Q13509	Tubulin beta-3 chain	3	0.30	0.262	4.4E-02
Q13425	Beta-2-syntrophin	3	0.30	0.055	2.1E-03
P02511	Alpha-crystallin B chain	5	0.30	0.241	7.2E-03
P09493	Tropomyosin alpha-1 chain	5	0.30	0.063	4.2E-05
P02462	Collagen alpha-1(IV) chain	4	0.29	0.328	3.3E-02
P27105	Erythrocyte band 7 integral membrane protein	5	0.29	0.278	1.1E-02
Q01995	Transgelin	5	0.28	0.210	3.8E-03
P08294	Extracellular superoxide dismutase [Cu-Zn]	5	0.28	0.138	7.8E-04
P02671	Fibrinogen alpha chain	5	0.28	0.446	4.6E-02
P08572	Collagen alpha-2(IV) chain	5	0.28	0.242	6.2E-03
Q6NZI2	Polymerase I and transcript release factor	5	0.28	0.159	1.3E-03
P16157	Ankyrin-1	5	0.27	0.297	1.2E-02
P55268	Laminin subunit beta-2	5	0.27	0.154	1.1E-03
Q02952	A-kinase anchor protein 12	5	0.27	0.197	2.7E-03
P50895	Basal cell adhesion molecule	4	0.27	0.069	3.2E-04
Q9BS40	Latexin	4	0.27	0.365	3.7E-02
Q13642	Four and a half LIM domains protein 1	3	0.27	0.180	1.8E-02
P98160	Basement membrane-specific heparan sulfate proteoglycan core protein	5	0.26	0.169	1.4E-03
O43301	Heat shock 70 kDa protein 12A	4	0.26	0.054	1.4E-04
P02549	Spectrin alpha chain, erythrocytic 1	5	0.26	0.246	5.2E-03
O43491	Band 4.1-like protein 2	5	0.25	0.170	1.3E-03
P17661	Desmin	3	0.25	0.164	1.4E-02
Q14624	Inter-alpha-trypsin inhibitor heavy chain H4	3	0.25	0.211	2.3E-02
P80723	Brain acid soluble protein 1	5	0.25	0.181	1.6E-03
Q14699	Raftlin	3	0.25	0.143	1.0E-02
P46821	Microtubule-associated protein 1B	3	0.25	0.141	1.0E-02
P11047	Laminin subunit gamma-1	5	0.25	0.206	2.4E-03
P00167	Cytochrome b5	5	0.24	0.081	6.5E-05
P01008	Antithrombin-III	5	0.24	0.367	1.8E-02
P11277	Spectrin beta chain, erythrocytic	5	0.23	0.297	8.1E-03
Q16363	Laminin subunit alpha-4	5	0.23	0.226	3.0E-03
Q9BXN1	Asporin	4	0.23	0.351	2.5E-02
P04083	Annexin A1	5	0.23	0.246	3.9E-03
Q14112	Nidogen-2	5	0.23	0.173	1.0E-03
Q969G5	Protein kinase C delta-binding protein	4	0.23	0.193	4.5E-03
P36269	Gamma-glutamyltransferase 5	5	0.22	0.169	8.8E-04
Q9Y6C2	EMILIN-1	5	0.22	0.154	6.0E-04
P02649	Apolipoprotein E	5	0.22	0.337	1.1E-02
P07197	Neurofilament medium polypeptide	5	0.22	0.067	2.2E-05
O94875	Sorbin and SH3 domain-containing protein 2	5	0.22	0.107	1.4E-04
Q16555	Dihydropyrimidinase-related protein 2	5	0.21	0.107	1.4E-04
P14543	Nidogen-1	5	0.21	0.188	1.2E-03
P60903	Protein S100-A10	5	0.21	0.096	8.5E-05
P22105	Tenascin-X	4	0.21	0.200	4.2E-03
P68366	Tubulin alpha-4A chain	3	0.21	0.133	7.0E-03
P07360	Complement component C8 gamma chain	4	0.20	0.194	3.7E-03
Q12805	EGF-containing fibulin-like extracellular matrix protein 1	4	0.20	0.335	1.7E-02
P02686	Myelin basic protein	5	0.20	0.110	1.3E-04
P39059	Collagen alpha-1(XV) chain	5	0.19	0.216	1.6E-03
P11166	Solute carrier family 2, facilitated glucose transporter member 1	5	0.18	0.280	3.7E-03
P68032	Actin, alpha cardiac muscle 1	4	0.17	0.188	2.6E-03
P02730	Band 3 anion transport protein	5	0.17	0.391	1.1E-02
P05186	Alkaline phosphatase, tissue-nonspecific isozyme	5	0.17	0.185	6.5E-04
P58166	Inhibin beta E chain	5	0.17	0.196	8.0E-04
Q05707	Collagen alpha-1(XIV) chain	5	0.17	0.180	5.7E-04
P04275	von Willebrand factor	5	0.17	0.138	2.0E-04
P43320	Beta-crystallin B2	4	0.16	0.076	1.6E-04
Q13885	Tubulin beta-2A chain	3	0.16	0.334	3.2E-02
O14495	Lipid phosphate phosphohydrolase 3	4	0.16	0.170	1.7E-03
Q03135	Caveolin-1	5	0.16	0.240	1.6E-03
P02760	Protein AMBP	5	0.16	0.081	2.1E-05
Q15661	Tryptase alpha/beta-1	5	0.15	0.154	2.7E-04
Q2UY09	Collagen alpha-1(XXVIII) chain	3	0.15	0.246	1.7E-02
P01871	Ig mu chain C region	5	0.15	0.272	2.2E-03
Q9BXM0	Periaxin	5	0.15	0.202	7.1E-04
P01031	Complement C5	5	0.15	0.267	2.0E-03
P20774	Mimecan	5	0.15	0.101	4.5E-05
Q9UBX5	Fibulin-5	4	0.15	0.260	5.1E-03
P35749	Myosin-11	5	0.14	0.089	2.6E-05
P24844	Myosin regulatory light polypeptide 9	5	0.14	0.115	7.0E-05
Q14195	Dihydropyrimidinase-related protein 3	5	0.13	0.084	1.9E-05
P21980	Protein-glutamine gamma-glutamyltransferase 2	5	0.13	0.129	9.7E-05
P21810	Biglycan	5	0.13	0.159	2.2E-04
P51884	Lumican	5	0.13	0.178	3.3E-04
P35555	Fibrillin-1	5	0.13	0.226	8.3E-04
P21926	CD9 antigen	5	0.13	0.245	1.1E-03
Q16853	Membrane primary amine oxidase	4	0.12	0.077	1.1E-04
P10909	Clusterin	5	0.12	0.235	8.5E-04
P51888	Prolargin	5	0.12	0.129	7.9E-05
P41219	Peripherin	5	0.12	0.118	5.5E-05
P08123	Collagen alpha-2(I) chain	5	0.12	0.483	1.1E-02
P07585	Decorin	5	0.11	0.193	3.5E-04
P22352	Glutathione peroxidase 3	5	0.11	0.263	1.1E-03
P15088	Mast cell carboxypeptidase A	5	0.11	0.113	3.8E-05
P35625	Metalloproteinase inhibitor 3	5	0.10	0.226	5.6E-04
P02748	Complement component C9	5	0.10	0.202	3.5E-04
P04004	Vitronectin	5	0.10	0.341	2.5E-03
P22748	Carbonic anhydrase 4	5	0.09	0.117	3.5E-05
P23946	Chymase	5	0.09	0.253	6.9E-04
P10745	Retinol-binding protein 3	4	0.08	0.259	2.4E-03
P25189	Myelin protein P0	5	0.07	0.139	4.8E-05
P63211	Guanine nucleotide-binding protein G(T) subunit gamma-T1	3	0.07	0.184	4.8E-03
P02743	Serum amyloid P-component	5	0.07	0.267	5.6E-04

Average LC MS/MS iTRAQ results from metastasized UM 19, 21, 24, 28, 30. Brown denotes change ≥ 2 standard deviations (SD) from the mean, yellow denotes change ≥ 1 SD and green highlights p values ≤ 0.05. NA, not applicable, n<3 samples.
