# Supplementary material for: iTRAQ Quantitative Proteomic Comparison of Metastatic and Non-Metastatic Uveal Melanoma Tumors
Source: PLoS One. 2015 Aug 25;10(8):e0135543. doi: 10.1371/journal.pone.0135543 (PMC4549237; doi:10.1371/journal.pone.0135543)
Supplement: S17 Table — (PDF) [file pone.0135543.s017.pdf]

Supplementary Table S17

Average Relative Protein Abundance: Non-Metastasized Tumors (Samples UM13, UM20, UM23, UM25, UM26)

Total Proteins Quantified = 1389; LogMedian Protein Ratio = 0.13; LogMean Protein Ratio =0; Standard Deviation = 0.75

Uni-Prot Accession	Protein	Sample Frequency n = 5 total	Ratio UM/Control	SEM	*p* value
P62937	Peptidyl-prolyl cis-trans isomerase A	5	5.18	0.071	2.0E-05
P30086	Phosphatidylethanolamine-binding protein 1	5	4.46	0.113	1.9E-04
O75368	SH3 domain-binding glutamic acid-rich-like protein	3	4.38	0.275	3.3E-02
P17096	High mobility group protein HMG-I/HMG-Y	5	4.16	0.198	2.0E-03
P06454	Prothymosin alpha	5	4.08	0.297	9.1E-03
P07108	Acyl-CoA-binding protein	5	3.89	0.131	4.9E-04
P16402	Histone H1.3	3	3.85	0.200	2.1E-02
P78417	Glutathione S-transferase omega-1	5	3.60	0.020	3.8E-07
P07195	L-lactate dehydrogenase B chain	5	3.51	0.066	4.4E-05
O14556	Glyceraldehyde-3-phosphate dehydrogenase, testis-specific	5	3.49	0.214	4.2E-03
P63241	Eukaryotic translation initiation factor 5A-1	5	3.47	0.104	2.8E-04
Q04760	Lactoylglutathione lyase	5	3.46	0.167	1.7E-03
P24941	Cyclin-dependent kinase 2	4	3.46	0.047	1.2E-04
P10599	Thioredoxin	5	3.45	0.219	4.8E-03
P00338	L-lactate dehydrogenase A chain	5	3.40	0.156	1.4E-03
P60174	Triosephosphate isomerase	5	3.38	0.105	3.1E-04
P09211	Glutathione S-transferase P	5	3.29	0.198	3.8E-03
P58546	Myotrophin	3	3.25	0.096	6.5E-03
P00558	Phosphoglycerate kinase 1	5	3.17	0.092	2.4E-04
P06733	Alpha-enolase	5	3.11	0.078	1.3E-04
Q16658	Fascin	3	3.11	0.075	4.4E-03
Q9Y2S2	Lambda-crystallin homolog	5	3.10	0.133	1.1E-03
Q9HC38	Glyoxalase domain-containing protein 4	4	3.10	0.200	1.1E-02
P04080	Cystatin-B	5	3.03	0.064	6.6E-05
P31939	Bifunctional purine biosynthesis protein PURH	4	3.01	0.199	1.2E-02
P22087	rRNA 2'-O-methyltransferase fibrillarin	5	2.99	0.104	4.6E-04
P04406	Glyceraldehyde-3-phosphate dehydrogenase	5	2.97	0.166	2.8E-03
O75348	V-type proton ATPase subunit G 1	3	2.96	0.233	4.3E-02
P15121	Aldose reductase	4	2.95	0.188	1.0E-02
P12955	Xaa-Pro dipeptidase	4	2.95	0.121	2.9E-03
O75347	Tubulin-specific chaperone A	4	2.94	0.074	7.0E-04
P52565	Rho GDP-dissociation inhibitor 1	5	2.88	0.105	5.4E-04
P30043	Flavin reductase (NADPH)	3	2.87	0.053	2.5E-03
P06865	Beta-hexosaminidase subunit alpha	3	2.87	0.128	1.4E-02
P61604	10 kDa heat shock protein, mitochondrial	5	2.81	0.202	6.9E-03
P19338	Nucleolin	5	2.77	0.093	3.9E-04
P16070	CD44 antigen	5	2.74	0.128	1.4E-03
Q15819	Ubiquitin-conjugating enzyme E2 variant 2	3	2.72	0.114	1.3E-02
P16401	Histone H1.5	5	2.70	0.192	6.7E-03
O15400	Syntaxin-7	5	2.69	0.157	3.2E-03
P29401	Transketolase	5	2.69	0.024	2.1E-06
P07900	Heat shock protein HSP 90-alpha	5	2.64	0.148	2.8E-03
P13797	Plastin-3	4	2.61	0.126	4.7E-03
P15531	Nucleoside diphosphate kinase A	4	2.60	0.162	9.7E-03
Q9Y2X3	Nucleolar protein 58	4	2.59	0.065	6.8E-04
P40925	Malate dehydrogenase, cytoplasmic	5	2.58	0.055	6.5E-05
P07741	Adenine phosphoribosyltransferase	3	2.57	0.082	7.5E-03
P13489	Ribonuclease inhibitor	3	2.56	0.129	1.8E-02
Q00796	Sorbitol dehydrogenase	4	2.56	0.041	1.8E-04
P31948	Stress-induced-phosphoprotein 1	5	2.54	0.112	1.1E-03
O95834	Echinoderm microtubule-associated protein-like 2	3	2.53	0.215	5.0E-02
P51858	Hepatoma-derived growth factor	5	2.52	0.158	4.2E-03
P63104	14-3-3 protein zeta/delta	5	2.52	0.145	3.1E-03
P57729	Ras-related protein Rab-38	4	2.51	0.119	4.4E-03
P40926	Malate dehydrogenase, mitochondrial	5	2.49	0.145	3.3E-03
P62750	60S ribosomal protein L23a	4	2.45	0.084	1.7E-03
Q13510	Acid ceramidase	5	2.42	0.153	4.4E-03
O95336	6-phosphogluconolactonase	5	2.41	0.063	1.5E-04
Q99497	Protein DJ-1	5	2.35	0.058	1.2E-04
Q53EL6	Programmed cell death protein 4	4	2.35	0.190	2.0E-02
P07954	Fumarate hydratase, mitochondrial	4	2.34	0.084	2.0E-03
P51149	Ras-related protein Rab-7a	5	2.33	0.069	2.6E-04
P08758	Annexin A5	5	2.30	0.128	2.9E-03
P62826	GTP-binding nuclear protein Ran	4	2.29	0.165	1.5E-02
P23526	Adenosylhomocysteinase	5	2.28	0.092	8.5E-04
Q96GK7	Fumarylacetoacetate hydrolase domain-containing protein 2A	3	2.27	0.115	1.9E-02
P07737	Profilin-1	5	2.26	0.077	4.4E-04
P23528	Cofilin-1	5	2.26	0.064	2.2E-04
P06748	Nucleophosmin	5	2.25	0.038	3.0E-05
P08238	Heat shock protein HSP 90-beta	5	2.24	0.165	8.1E-03
Q13185	Chromobox protein homolog 3	5	2.23	0.085	7.0E-04
P20042	Eukaryotic translation initiation factor 2 subunit 2	4	2.23	0.197	2.7E-02
P16152	Carbonyl reductase [NADPH] 1	5	2.21	0.129	3.5E-03
Q13126	S-methyl-5'-thioadenosine phosphorylase	3	2.20	0.117	2.1E-02
P06744	Glucose-6-phosphate isomerase	5	2.20	0.081	6.1E-04
P21283	V-type proton ATPase subunit C 1	3	2.19	0.103	1.7E-02
Q13838	Spliceosome RNA helicase DDX39B	4	2.19	0.081	2.3E-03
P30044	Peroxiredoxin-5, mitochondrial	5	2.18	0.088	9.1E-04
Q07955	Serine/arginine-rich splicing factor 1	5	2.17	0.070	3.9E-04
P14174	Macrophage migration inhibitory factor	2	15.28	NA	NA
P05204	Non-histone chromosomal protein HMG-17	1	7.97	NA	NA
Q6EEV6	Small ubiquitin-related modifier 4	1	5.69	NA	NA
O00625	Pirin	2	5.29	NA	NA
Q9H3G5	Probable serine carboxypeptidase CPVL	1	4.99	NA	NA
Q9Y6U3	Adseverin	1	4.47	NA	NA
P55769	NHP2-like protein 1	2	4.40	NA	NA
Q9UBR2	Cathepsin Z	2	4.37	NA	NA
P62328	Thymosin beta-4	2	4.33	NA	NA
Q8WWM9	Cytoglobin	1	3.94	NA	NA
O60220	Mitochondrial import inner membrane translocase subunit Tim8 A	1	3.85	NA	NA
P13686	Tartrate-resistant acid phosphatase type 5	1	3.84	NA	NA
Q8NCW5	NAD(P)H-hydrate epimerase	1	3.82	NA	NA
Q6XQN6	Nicotinate phosphoribosyltransferase	1	3.71	NA	NA
P22234	Multifunctional protein ADE2	1	3.71	NA	NA
P62310	U6 snRNA-associated Sm-like protein LSm3	1	3.68	NA	NA
P40121	Macrophage-capping protein	2	3.61	NA	NA
P43235	Cathepsin K	1	3.53	NA	NA
P16083	Ribosyldihydronicotinamide dehydrogenase [quinone]	1	3.47	NA	NA
O60888	Protein CutA	1	3.46	NA	NA
Q9UHB6	LIM domain and actin-binding protein 1	1	3.26	NA	NA
O00584	Ribonuclease T2	1	3.12	NA	NA
P20039	HLA class II histocompatibility antigen, DRB1-11 beta chain	1	3.07	NA	NA
P61916	Epididymal secretory protein E1	1	3.01	NA	NA
P35270	Sepiapterin reductase	1	2.86	NA	NA
P0DME0	Protein SETSIP	2	2.83	NA	NA
P27695	DNA-(apurinic or apyrimidinic site) lyase	2	2.80	NA	NA
Q8IV08	Phospholipase D3	4	2.78	0.359	6.5E-02
P05413	Fatty acid-binding protein, heart	2	2.76	NA	NA
P23297	Protein S100-A1	1	2.73	NA	NA
P82979	SAP domain-containing ribonucleoprotein	1	2.73	NA	NA
P11766	Alcohol dehydrogenase class-3	2	2.71	NA	NA
P17900	Ganglioside GM2 activator	1	2.71	NA	NA
P25789	Proteasome subunit alpha type-4	1	2.70	NA	NA
Q9UHL4	Dipeptidyl peptidase 2	1	2.65	NA	NA
P16949	Stathmin	2	2.63	NA	NA
Q9BUP0	EF-hand domain-containing protein D1	2	2.63	NA	NA
P52566	Rho GDP-dissociation inhibitor 2	1	2.63	NA	NA
Q9H0R4	Haloacid dehalogenase-like hydrolase domain-containing protein 2	1	2.62	NA	NA
Q9BYT8	Neurolysin, mitochondrial	1	2.58	NA	NA
P07686	Beta-hexosaminidase subunit beta	2	2.58	NA	NA
Q99572	P2X purinoceptor 7	2	2.57	NA	NA
P39748	Flap endonuclease 1	1	2.55	NA	NA
P36959	GMP reductase 1	1	2.52	NA	NA
P30085	UMP-CMP kinase	1	2.50	NA	NA
Q9BV36	Melanophilin	2	2.49	NA	NA
P23381	Tryptophan--tRNA ligase, cytoplasmic	2	2.49	NA	NA
P55010	Eukaryotic translation initiation factor 5	1	2.48	NA	NA
Q9NR28	Diablo homolog, mitochondrial	1	2.48	NA	NA
P16930	Fumarylacetoacetase	1	2.48	NA	NA
Q13243	Serine/arginine-rich splicing factor 5	2	2.47	NA	NA
Q5JNZ5	Putative 40S ribosomal protein S26-like 1	2	2.44	NA	NA
Q96A05	V-type proton ATPase subunit E 2	1	2.42	NA	NA
Q9H307	Pinin	1	2.41	NA	NA
P49189	4-trimethylaminobutyraldehyde dehydrogenase	1	2.34	NA	NA
Q8IZQ5	Selenoprotein H	2	2.33	NA	NA
P20591	Interferon-induced GTP-binding protein Mx1	1	2.32	NA	NA
P55809	Succinyl-CoA:3-ketoacid coenzyme A transferase 1, mitochondrial	2	2.32	NA	NA
Q03154	Aminoacylase-1	2	2.29	NA	NA
O75531	Barrier-to-autointegration factor	2	2.26	NA	NA
P21291	Cysteine and glycine-rich protein 1	2	2.26	NA	NA
Q15185	Prostaglandin E synthase 3	1	2.25	NA	NA
O14618	Copper chaperone for superoxide dismutase	1	2.24	NA	NA
Q07021	Complement component 1 Q subcomponent-binding protein, mitochondrial	1	2.22	NA	NA
P00568	Adenylate kinase isoenzyme 1	1	2.22	NA	NA
P62861	40S ribosomal protein S30	1	2.21	NA	NA
P52815	39S ribosomal protein L12, mitochondrial	3	2.20	0.236	7.9E-02
Q9UL12	Sarcosine dehydrogenase, mitochondrial	1	2.18	NA	NA
P10253	Lysosomal alpha-glucosidase	1	2.18	NA	NA
Q9NYF8	Bcl-2-associated transcription factor 1	2	2.16	NA	NA
P10768	S-formylglutathione hydrolase	2	2.15	NA	NA
Q658Y4	Protein FAM91A1	1	2.15	NA	NA
Q15102	Platelet-activating factor acetylhydrolase IB subunit gamma	3	2.15	0.281	1.1E-01
Q9NRX4	14 kDa phosphohistidine phosphatase	1	2.13	NA	NA
P63279	SUMO-conjugating enzyme UBC9	2	2.13	NA	NA
P13798	Acylamino-acid-releasing enzyme	3	2.11	0.062	6.8E-03
Q14677	Clathrin interactor 1	2	2.11	NA	NA
Q9NPJ3	Acyl-coenzyme A thioesterase 13	1	2.11	NA	NA
P30040	Endoplasmic reticulum resident protein 29	1	2.10	NA	NA
P01620	Ig kappa chain V-III region SIE	1	2.08	NA	NA
P08670	Vimentin	5	2.07	0.208	2.5E-02
Q99729	Heterogeneous nuclear ribonucleoprotein A/B	1	2.07	NA	NA
P12081	Histidine--tRNA ligase, cytoplasmic	1	2.06	NA	NA
P61088	Ubiquitin-conjugating enzyme E2 N	2	2.06	NA	NA
P50395	Rab GDP dissociation inhibitor beta	5	2.06	0.063	3.3E-04
P02768	Serum albumin	5	2.05	0.195	2.1E-02
P28072	Proteasome subunit beta type-6	1	2.05	NA	NA
Q04837	Single-stranded DNA-binding protein, mitochondrial	1	2.05	NA	NA
P63173	60S ribosomal protein L38	1	2.03	NA	NA
Q00059	Transcription factor A, mitochondrial	5	2.03	0.180	1.7E-02
P08195	4F2 cell-surface antigen heavy chain	5	2.03	0.066	4.3E-04
Q3SY69	Mitochondrial 10-formyltetrahydrofolate dehydrogenase	2	2.03	NA	NA
Q9UBQ7	Glyoxylate reductase/hydroxypyruvate reductase	1	2.03	NA	NA
Q9BRA2	Thioredoxin domain-containing protein 17	3	2.02	0.101	2.0E-02
Q99536	Synaptic vesicle membrane protein VAT-1 homolog	5	2.02	0.038	5.3E-05
P21266	Glutathione S-transferase Mu 3	4	2.02	0.129	1.2E-02
Q04446	1,4-alpha-glucan-branching enzyme	2	2.00	NA	NA
Q86WA6	Valacyclovir hydrolase	1	2.00	NA	NA
Q9UH65	Switch-associated protein 70	5	2.00	0.090	1.6E-03
Q15843	NEDD8	1	1.99	NA	NA
P50453	Serpin B9	3	1.99	0.172	5.7E-02
Q01105	Protein SET	3	1.98	0.041	3.7E-03

Q13442	28 kDa heat- and acid-stable phosphoprotein	2	1.98	NA	NA
P25786	Proteasome subunit alpha type-1	5	1.98	0.131	6.5E-03
Q6PI48	Aspartate--tRNA ligase, mitochondrial	2	1.98	NA	NA
Q9UI12	V-type proton ATPase subunit H	1	1.97	NA	NA
P53985	Monocarboxylate transporter 1	1	1.97	NA	NA
P09914	Interferon-induced protein with tetratricopeptide repeats 1	1	1.97	NA	NA
Q9H4A4	Aminopeptidase B	2	1.97	NA	NA
P22392	Nucleoside diphosphate kinase B	1	1.96	NA	NA
Q14247	Src substrate cortactin	5	1.96	0.113	4.0E-03
Q9Y2W1	Thyroid hormone receptor-associated protein 3	2	1.96	NA	NA
P00915	Carbonic anhydrase 1	1	1.95	NA	NA
P38159	RNA-binding motif protein, X chromosome	1	1.95	NA	NA
P13693	Translationally-controlled tumor protein	4	1.94	0.079	3.6E-03
P20618	Proteasome subunit beta type-1	4	1.94	0.040	4.8E-04
P16219	Short-chain specific acyl-CoA dehydrogenase, mitochondrial	3	1.93	0.273	1.4E-01
Q14240	Eukaryotic initiation factor 4A-II	3	1.93	0.058	7.8E-03
P62847	40S ribosomal protein S24	1	1.92	NA	NA
P27348	14-3-3 protein theta	4	1.92	0.171	3.2E-02
Q9Y285	Phenylalanine--tRNA ligase alpha subunit	1	1.91	NA	NA
P33121	Long-chain-fatty-acid--CoA ligase 1	1	1.91	NA	NA
P62258	14-3-3 protein epsilon	5	1.91	0.094	2.3E-03
Q96C86	m7GpppX diphosphatase	2	1.91	NA	NA
Q16836	Hydroxyacyl-coenzyme A dehydrogenase, mitochondrial	4	1.90	0.142	2.0E-02
P60900	Proteasome subunit alpha type-6	2	1.90	NA	NA
P07858	Cathepsin B	4	1.89	0.227	6.7E-02
P30485	HLA class I histocompatibility antigen, B-47 alpha chain	1	1.89	NA	NA
Q12906	Interleukin enhancer-binding factor 3	5	1.89	0.079	1.3E-03
P18669	Phosphoglycerate mutase 1	5	1.88	0.089	2.1E-03
Q96I99	Succinyl-CoA ligase [GDP-forming] subunit beta, mitochondrial	3	1.87	0.132	4.1E-02
O00217	NADH dehydrogenase [ubiquinone] iron-sulfur protein 8, mitochondrial	1	1.87	NA	NA
P04181	Ornithine aminotransferase, mitochondrial	1	1.87	NA	NA
Q5VWZ2	Lysophospholipase-like protein 1	1	1.86	NA	NA
Q15907	Ras-related protein Rab-11B	4	1.86	0.140	2.1E-02
P31937	3-hydroxyisobutyrate dehydrogenase, mitochondrial	2	1.85	NA	NA
P99999	Cytochrome c	5	1.85	0.107	4.5E-03
P51608	Methyl-CpG-binding protein 2	4	1.85	0.151	2.6E-02
P48147	Prolyl endopeptidase	1	1.85	NA	NA
Q9H7C9	Mth938 domain-containing protein	1	1.85	NA	NA
P46776	60S ribosomal protein L27a	3	1.85	0.068	1.2E-02
P10809	60 kDa heat shock protein, mitochondrial	5	1.84	0.133	1.0E-02
Q9UQ80	Proliferation-associated protein 2G4	3	1.83	0.083	1.8E-02
Q15631	Translin	4	1.83	0.200	5.6E-02
Q15181	Inorganic pyrophosphatase	3	1.83	0.051	6.9E-03
Q13435	Splicing factor 3B subunit 2	5	1.83	0.127	9.0E-03
Q04917	14-3-3 protein eta	1	1.83	NA	NA
Q9Y3C8	Ubiquitin-fold modifier-conjugating enzyme 1	2	1.83	NA	NA
O76021	Ribosomal L1 domain-containing protein 1	1	1.83	NA	NA
P00441	Superoxide dismutase [Cu-Zn]	5	1.83	0.184	3.1E-02
Q9Y4W6	AFG3-like protein 2	5	1.82	0.100	3.9E-03
O00567	Nucleolar protein 56	5	1.82	0.050	2.8E-04
Q9UBQ5	Eukaryotic translation initiation factor 3 subunit K	1	1.82	NA	NA
P84077	ADP-ribosylation factor 1	1	1.81	NA	NA
Q9Y653	G-protein coupled receptor 56	1	1.81	NA	NA
P60842	Eukaryotic initiation factor 4A-I	4	1.81	0.023	1.3E-04
Q13428	Treacle protein	2	1.81	NA	NA
P08621	U1 small nuclear ribonucleoprotein 70 kDa	2	1.80	NA	NA
O43399	Tumor protein D54	4	1.80	0.137	2.3E-02
P53999	Activated RNA polymerase II transcriptional coactivator p15	1	1.80	NA	NA
P49720	Proteasome subunit beta type-3	1	1.79	NA	NA
Q14103	Heterogeneous nuclear ribonucleoprotein D0	5	1.79	0.018	5.2E-06
Q12905	Interleukin enhancer-binding factor 2	5	1.78	0.059	6.3E-04
P14618	Pyruvate kinase PKM	5	1.77	0.115	7.7E-03
P05141	ADP/ATP translocase 2	3	1.77	0.223	1.2E-01
P13639	Elongation factor 2	5	1.77	0.057	5.4E-04
P20702	Integrin alpha-X	1	1.77	NA	NA
P07910	Heterogeneous nuclear ribonucleoproteins C1/C2	5	1.77	0.067	1.0E-03
Q15424	Scaffold attachment factor B1	1	1.76	NA	NA
Q13619	Cullin-4A	1	1.76	NA	NA
Q8WWM7	Ataxin-2-like protein	1	1.75	NA	NA
P30837	Aldehyde dehydrogenase X, mitochondrial	5	1.75	0.288	1.2E-01
P46926	Glucosamine-6-phosphate isomerase 1	4	1.75	0.105	1.3E-02
Q9NY12	H/ACA ribonucleoprotein complex subunit 1	4	1.75	0.068	3.8E-03
Q92597	Protein NDRG1	3	1.75	0.043	5.7E-03
P48739	Phosphatidylinositol transfer protein beta isoform	1	1.75	NA	NA
P02787	Serotransferrin	5	1.75	0.161	2.6E-02
P25788	Proteasome subunit alpha type-3	2	1.75	NA	NA
Q9UL46	Proteasome activator complex subunit 2	2	1.74	NA	NA
O14818	Proteasome subunit alpha type-7	5	1.74	0.116	8.6E-03
O75380	NADH dehydrogenase [ubiquinone] iron-sulfur protein 6, mitochondrial	3	1.74	0.052	8.6E-03
O95571	Persulfide dioxygenase ETHE1, mitochondrial	1	1.74	NA	NA
P35237	Serpin B6	1	1.73	NA	NA
Q9UNF0	Protein kinase C and casein kinase substrate in neurons protein 2	2	1.73	NA	NA
P46783	40S ribosomal protein S10	3	1.73	0.133	5.4E-02
Q9UHX1	Poly(U)-binding-splicing factor PUF60	4	1.73	0.098	1.2E-02
O14841	5-oxoprolinase	1	1.72	NA	NA
P38646	Stress-70 protein, mitochondrial	5	1.72	0.134	1.5E-02
Q53H82	Beta-lactamase-like protein 2	2	1.72	NA	NA
P55786	Puromycin-sensitive aminopeptidase	5	1.72	0.066	1.2E-03
P62857	40S ribosomal protein S28	3	1.71	0.144	6.5E-02
P09661	U2 small nuclear ribonucleoprotein A'	2	1.71	NA	NA
Q13405	39S ribosomal protein L49, mitochondrial	1	1.71	NA	NA
P39687	Acidic leucine-rich nuclear phosphoprotein 32 family member A	5	1.71	0.079	2.5E-03
Q96KP4	Cytosolic non-specific dipeptidase	5	1.71	0.125	1.3E-02
O00148	ATP-dependent RNA helicase DDX39A	1	1.70	NA	NA
P62158	Calmodulin	5	1.70	0.114	9.4E-03
Q9UKY7	Protein CDV3 homolog	1	1.70	NA	NA
P49773	Histidine triad nucleotide-binding protein 1	4	1.70	0.123	2.3E-02
P54819	Adenylate kinase 2, mitochondrial	5	1.69	0.060	9.4E-04
Q8WUY3	Protein prune homolog 2	1	1.69	NA	NA
P07919	Cytochrome b-c1 complex subunit 6, mitochondrial	3	1.69	0.289	2.1E-01
P09429	High mobility group protein B1	5	1.69	0.099	6.2E-03
O75436	Vacuolar protein sorting-associated protein 26A	1	1.69	NA	NA
Q00839	Heterogeneous nuclear ribonucleoprotein U	5	1.68	0.110	9.2E-03
P30405	Peptidyl-prolyl cis-trans isomerase F, mitochondrial	3	1.68	0.133	6.0E-02
P02765	Alpha-2-HS-glycoprotein	2	1.68	NA	NA
Q5JTV8	Torsin-1A-interacting protein 1	4	1.68	0.058	3.0E-03
P21796	Voltage-dependent anion-selective channel protein 1	5	1.67	0.076	2.4E-03
O00154	Cytosolic acyl coenzyme A thioester hydrolase	1	1.67	NA	NA
P42224	Signal transducer and activator of transcription 1-alpha/beta	1	1.67	NA	NA
Q9GZT3	SRA stem-loop-interacting RNA-binding protein, mitochondrial	1	1.67	NA	NA
Q99798	Aconitate hydratase, mitochondrial	5	1.67	0.106	8.5E-03
P25398	40S ribosomal protein S12	5	1.67	0.062	1.2E-03
P02786	Transferrin receptor protein 1	1	1.67	NA	NA
P28066	Proteasome subunit alpha type-5	3	1.67	0.140	6.8E-02
Q16666	Gamma-interferon-inducible protein 16	3	1.66	0.233	1.6E-01
Q9UNZ2	NSFL1 cofactor p47	2	1.66	NA	NA
P30041	Peroxiredoxin-6	5	1.66	0.097	6.5E-03
O00299	Chloride intracellular channel protein 1	2	1.66	NA	NA
O75494	Serine/arginine-rich splicing factor 10	1	1.66	NA	NA
Q13177	Serine/threonine-protein kinase PAK 2	4	1.66	0.195	8.0E-02
E9PAV3	Nascent polypeptide-associated complex subunit alpha, muscle-specific form	2	1.66	NA	NA
O15533	Tapasin	1	1.66	NA	NA
P62820	Ras-related protein Rab-1A	1	1.66	NA	NA
P05387	60S acidic ribosomal protein P2	5	1.65	0.067	1.7E-03
P28161	Glutathione S-transferase Mu 2	1	1.65	NA	NA
Q99523	Sortilin	2	1.65	NA	NA
Q86UX7	Fermitin family homolog 3	3	1.65	0.057	1.3E-02
Q9NSD9	Phenylalanine--tRNA ligase beta subunit	3	1.65	0.174	1.0E-01
P09651	Heterogeneous nuclear ribonucleoprotein A1	5	1.65	0.054	7.5E-04
Q15063	Periostin	2	1.64	NA	NA
Q9UMY4	Sorting nexin-12	2	1.64	NA	NA
O94826	Mitochondrial import receptor subunit TOM70	5	1.64	0.098	7.1E-03
P54652	Heat shock-related 70 kDa protein 2	5	1.64	0.124	1.6E-02
Q08380	Galectin-3-binding protein	5	1.64	0.062	1.4E-03
Q8NC51	Plasminogen activator inhibitor 1 RNA-binding protein	1	1.64	NA	NA
O75083	WD repeat-containing protein 1	5	1.64	0.070	2.1E-03
Q92820	Gamma-glutamyl hydrolase	2	1.64	NA	NA
P27635	60S ribosomal protein L10	3	1.64	0.122	5.6E-02
O75533	Splicing factor 3B subunit 1	3	1.64	0.082	2.7E-02
P27816	Microtubule-associated protein 4	5	1.63	0.119	1.5E-02
Q92688	Acidic leucine-rich nuclear phosphoprotein 32 family member B	4	1.63	0.136	3.7E-02
P49753	Acyl-coenzyme A thioesterase 2, mitochondrial	1	1.62	NA	NA
P06753	Tropomyosin alpha-3 chain	5	1.62	0.105	1.0E-02
P04350	Tubulin beta-4A chain	1	1.62	NA	NA
P12830	Cadherin-1	3	1.62	0.116	5.3E-02
P39019	40S ribosomal protein S19	5	1.62	0.089	5.7E-03
O15371	Eukaryotic translation initiation factor 3 subunit D	2	1.62	NA	NA
Q16543	Hsp90 co-chaperone Cdc37	2	1.62	NA	NA
P51798	H(+)/Cl(-) exchange transporter 7	1	1.61	NA	NA
P13929	Beta-enolase	1	1.61	NA	NA
Q9BZF1	Oxysterol-binding protein-related protein 8	1	1.61	NA	NA
Q9Y6C9	Mitochondrial carrier homolog 2	2	1.61	NA	NA
P07339	Cathepsin D	5	1.61	0.344	2.4E-01
P22626	Heterogeneous nuclear ribonucleoproteins A2/B1	5	1.61	0.047	5.4E-04
P10155	60 kDa SS-A/Ro ribonucleoprotein	1	1.61	NA	NA
Q8NHP8	Putative phospholipase B-like 2	1	1.61	NA	NA
Q16576	Histone-binding protein RBBP7	2	1.60	NA	NA
Q96AG4	Leucine-rich repeat-containing protein 59	5	1.60	0.062	1.6E-03
P06737	Glycogen phosphorylase, liver form	5	1.60	0.228	1.1E-01
Q9NYL4	Peptidyl-prolyl cis-trans isomerase FKBP11	1	1.60	NA	NA
Q8N2K0	Monoacylglycerol lipase ABHD12	1	1.60	NA	NA
P55008	Allograft inflammatory factor 1	1	1.60	NA	NA
Q9Y2Q3	Glutathione S-transferase kappa 1	1	1.59	NA	NA
Q01130	Serine/arginine-rich splicing factor 2	3	1.59	0.063	1.8E-02
O60343	TBC1 domain family member 4	1	1.59	NA	NA
Q9H2U2	Inorganic pyrophosphatase 2, mitochondrial	3	1.59	0.070	2.2E-02
Q08945	FACT complex subunit SSRP1	1	1.59	NA	NA
O95758	Polypyrimidine tract-binding protein 3	1	1.59	NA	NA
P12270	Nucleoprotein TPR	5	1.59	0.098	9.4E-03
P0CW22	40S ribosomal protein S17-like	5	1.58	0.120	1.9E-02
P30042	ES1 protein homolog, mitochondrial	3	1.58	0.231	1.9E-01
P35659	Protein DEK	2	1.58	NA	NA
Q5VTE0	Putative elongation factor 1-alpha-like 3	5	1.57	0.084	5.6E-03
Q14157	Ubiquitin-associated protein 2-like	1	1.57	NA	NA
P35613	Basigin	4	1.57	0.194	1.0E-01
Q9Y371	Endophilin-B1	1	1.57	NA	NA
P42704	Leucine-rich PPR motif-containing protein, mitochondrial	5	1.57	0.080	4.8E-03
P62851	40S ribosomal protein S25	5	1.57	0.083	5.6E-03
P23246	Splicing factor, proline- and glutamine-rich	5	1.57	0.111	1.5E-02
Q1KMD3	Heterogeneous nuclear ribonucleoprotein U-like protein 2	5	1.56	0.068	2.8E-03
P49207	60S ribosomal protein L34	2	1.56	NA	NA
P61254	60S ribosomal protein L26	3	1.56	0.203	1.6E-01
Q06323	Proteasome activator complex subunit 1	5	1.56	0.086	6.8E-03
O00483	NADH dehydrogenase [ubiquinone] 1 alpha subcomplex subunit 4	4	1.56	0.075	9.9E-03
P62263	40S ribosomal protein S14	5	1.55	0.100	1.2E-02
P61247	40S ribosomal protein S3a	5	1.55	0.107	1.5E-02
P78324	Tyrosine-protein phosphatase non-receptor type substrate 1	1	1.55	NA	NA

O14979	Heterogeneous nuclear ribonucleoprotein D-like	4	1.55	0.062	6.0E-03
P08865	40S ribosomal protein SA	5	1.55	0.056	1.4E-03
Q9UKV3	Apoptotic chromatin condensation inducer in the nucleus	4	1.55	0.075	1.0E-02
O15498	Synaptobrevin homolog YKT6	1	1.54	NA	NA
P42766	60S ribosomal protein L35	2	1.54	NA	NA
O43488	Aflatoxin B1 aldehyde reductase member 2	1	1.54	NA	NA
P51991	Heterogeneous nuclear ribonucleoprotein A3	5	1.54	0.091	9.3E-03
P18621	60S ribosomal protein L17	3	1.54	0.019	2.0E-03
P26006	Integrin alpha-3	2	1.53	NA	NA
P51817	cAMP-dependent protein kinase catalytic subunit PRKX	1	1.53	NA	NA
Q13263	Transcription intermediary factor 1-beta	4	1.53	0.194	1.2E-01
P17480	Nucleolar transcription factor 1	1	1.52	NA	NA
Q8NFV4	Alpha/beta hydrolase domain-containing protein 11	4	1.52	0.159	7.7E-02
O75874	Isocitrate dehydrogenase [NADP] cytoplasmic	3	1.52	0.140	9.5E-02
P10412	Histone H1.4	1	1.52	NA	NA
Q9Y6M9	NADH dehydrogenase [ubiquinone] 1 beta subcomplex subunit 9	3	1.52	0.045	1.1E-02
P24534	Elongation factor 1-beta	5	1.52	0.150	5.0E-02
A4D1P6	WD repeat-containing protein 91	1	1.51	NA	NA
P05155	Plasma protease C1 inhibitor	1	1.51	NA	NA
Q08211	ATP-dependent RNA helicase A	5	1.51	0.090	1.0E-02
P53597	Succinyl-CoA ligase [ADP/GDP-forming] subunit alpha, mitochondrial	5	1.51	0.064	3.0E-03
P10619	Lysosomal protective protein	3	1.50	0.339	3.5E-01
P30512	HLA class I histocompatibility antigen, A-29 alpha chain	1	1.50	NA	NA
Q9UJZ1	Stomatin-like protein 2, mitochondrial	4	1.50	0.179	1.1E-01
Q9Y696	Chloride intracellular channel protein 4	2	1.49	NA	NA
P62906	60S ribosomal protein L10a	5	1.49	0.074	5.6E-03
O75400	Pre-mRNA-processing factor 40 homolog A	1	1.49	NA	NA
Q06210	Glutamine--fructose-6-phosphate aminotransferase [isomerizing] 1	1	1.49	NA	NA
Q15366	Poly(rC)-binding protein 2	2	1.49	NA	NA
Q9Y376	Calcium-binding protein 39	1	1.48	NA	NA
P49792	E3 SUMO-protein ligase RanBP2	2	1.48	NA	NA
O75390	Citrate synthase, mitochondrial	5	1.48	0.112	2.5E-02
Q12931	Heat shock protein 75 kDa, mitochondrial	1	1.48	NA	NA
Q9BWM7	Sideroflexin-3	1	1.48	NA	NA
Q9UKM9	RNA-binding protein Raly	5	1.48	0.127	3.7E-02
Q07960	Rho GTPase-activating protein 1	2	1.48	NA	NA
O15145	Actin-related protein 2/3 complex subunit 3	3	1.47	0.035	7.9E-03
P31153	S-adenosylmethionine synthase isoform type-2	5	1.47	0.138	4.9E-02
Q9UJS0	Calcium-binding mitochondrial carrier protein Aralar2	1	1.47	NA	NA
Q99436	Proteasome subunit beta type-7	2	1.47	NA	NA
Q86UE4	Protein LYRIC	3	1.47	0.147	1.2E-01
P51159	Ras-related protein Rab-27A	4	1.46	0.207	1.6E-01
Q6DKI1	60S ribosomal protein L7-like 1	1	1.46	NA	NA
P14550	Alcohol dehydrogenase [NADP(+)]	1	1.46	NA	NA
Q9HAV7	GrpE protein homolog 1, mitochondrial	1	1.46	NA	NA
P26373	60S ribosomal protein L13	5	1.46	0.111	2.8E-02
Q12904	Aminoacyl tRNA synthase complex-interacting multifunctional protein 1	2	1.46	NA	NA
Q92945	Far upstream element-binding protein 2	5	1.45	0.038	6.1E-04
P37802	Transgelin-2	3	1.45	0.047	1.5E-02
Q3ZCQ8	Mitochondrial import inner membrane translocase subunit TIM50	2	1.45	NA	NA
P61978	Heterogeneous nuclear ribonucleoprotein K	5	1.45	0.037	5.6E-04
P31150	Rab GDP dissociation inhibitor alpha	1	1.45	NA	NA
P62995	Transformer-2 protein homolog beta	1	1.45	NA	NA
O43678	NADH dehydrogenase [ubiquinone] 1 alpha subcomplex subunit 2	3	1.45	0.096	6.2E-02
Q16629	Serine/arginine-rich splicing factor 7	4	1.44	0.124	5.9E-02
Q08257	Quinone oxidoreductase	1	1.44	NA	NA
P62136	Serine/threonine-protein phosphatase PP1-alpha catalytic subunit	4	1.44	0.155	9.9E-02
O96000	NADH dehydrogenase [ubiquinone] 1 beta subcomplex subunit 10	2	1.44	NA	NA
Q13242	Serine/arginine-rich splicing factor 9	2	1.44	NA	NA
Q02318	Sterol 26-hydroxylase, mitochondrial	3	1.44	0.145	1.3E-01
P62241	40S ribosomal protein S8	4	1.44	0.068	1.3E-02
P07602	Prosaposin	5	1.44	0.169	9.8E-02
P04075	Fructose-bisphosphate aldolase A	5	1.44	0.077	9.0E-03
P51970	NADH dehydrogenase [ubiquinone] 1 alpha subcomplex subunit 8	2	1.44	NA	NA
Q9BXK5	Bcl-2-like protein 13	2	1.43	NA	NA
P63244	Guanine nucleotide-binding protein subunit beta-2-like 1	5	1.43	0.061	4.1E-03
Q13217	DnaJ homolog subfamily C member 3	1	1.43	NA	NA
P13674	Prolyl 4-hydroxylase subunit alpha-1	3	1.43	0.189	2.0E-01
O15511	Actin-related protein 2/3 complex subunit 5	4	1.43	0.068	1.4E-02
Q9BUJ2	Heterogeneous nuclear ribonucleoprotein U-like protein 1	4	1.42	0.162	1.2E-01
P09417	Dihydropteridine reductase	2	1.42	NA	NA
O95292	Vesicle-associated membrane protein-associated protein B/C	4	1.42	0.188	1.6E-01
P04792	Heat shock protein beta-1	5	1.42	0.230	2.0E-01
P62280	40S ribosomal protein S11	5	1.42	0.079	1.1E-02
Q15459	Splicing factor 3A subunit 1	2	1.42	NA	NA
P25685	DnaJ homolog subfamily B member 1	2	1.42	NA	NA
P52597	Heterogeneous nuclear ribonucleoprotein F	4	1.42	0.065	1.3E-02
Q12874	Splicing factor 3A subunit 3	3	1.42	0.134	1.2E-01
P22314	Ubiquitin-like modifier-activating enzyme 1	5	1.42	0.068	6.8E-03
O14880	Microsomal glutathione S-transferase 3	1	1.41	NA	NA
P49721	Proteasome subunit beta type-2	2	1.41	NA	NA
Q99471	Prefoldin subunit 5	1	1.41	NA	NA
P40967	Melanocyte protein PMEL	5	1.41	0.057	3.9E-03
Q01469	Fatty acid-binding protein, epidermal	1	1.41	NA	NA
Q15056	Eukaryotic translation initiation factor 4H	1	1.41	NA	NA
P02790	Hemopexin	5	1.41	0.110	3.6E-02
Q02790	Peptidyl-prolyl cis-trans isomerase FKBP4	2	1.41	NA	NA
Q13576	Ras GTPase-activating-like protein IQGAP2	1	1.41	NA	NA
P46777	60S ribosomal protein L5	5	1.40	0.077	1.2E-02
Q14139	Ubiquitin conjugation factor E4 A	1	1.40	NA	NA
P00751	Complement factor B	1	1.40	NA	NA
P84103	Serine/arginine-rich splicing factor 3	4	1.40	0.105	5.0E-02
Q92616	Translational activator GCN1	3	1.39	0.132	1.3E-01
P35232	Prohibitin	5	1.39	0.073	1.0E-02
Q9NX63	Coiled-coil-helix-coiled-coil-helix domain-containing protein 3, mitochondrial	4	1.39	0.071	1.9E-02
P37840	Alpha-synuclein	1	1.39	NA	NA
P51810	G-protein coupled receptor 143	5	1.39	0.094	2.5E-02
Q9NP81	Serine--tRNA ligase, mitochondrial	4	1.39	0.043	4.7E-03
Q86U42	Polyadenylate-binding protein 2	2	1.39	NA	NA
P61106	Ras-related protein Rab-14	2	1.39	NA	NA
P37837	Transaldolase	5	1.39	0.153	9.8E-02
P83731	60S ribosomal protein L24	5	1.39	0.219	2.1E-01
P60981	Destrin	2	1.39	NA	NA
P62829	60S ribosomal protein L23	4	1.39	0.083	2.9E-02
Q09028	Histone-binding protein RBBP4	2	1.39	NA	NA
P54727	UV excision repair protein RAD23 homolog B	5	1.39	0.087	2.0E-02
Q8IWB7	WD repeat and FYVE domain-containing protein 1	2	1.38	NA	NA
P19404	NADH dehydrogenase [ubiquinone] flavoprotein 2, mitochondrial	2	1.38	NA	NA
O60814	Histone H2B type 1-K	1	1.38	NA	NA
Q13277	Syntaxin-3	1	1.38	NA	NA
Q86UY8	5'-nucleotidase domain-containing protein 3	1	1.38	NA	NA
Q86V81	THO complex subunit 4	2	1.38	NA	NA
P50914	60S ribosomal protein L14	5	1.38	0.066	8.2E-03
P30049	ATP synthase subunit delta, mitochondrial	5	1.38	0.081	1.7E-02
Q9BQI0	Allograft inflammatory factor 1-like	3	1.38	0.130	1.3E-01
P30711	Glutathione S-transferase theta-1	1	1.37	NA	NA
Q07020	60S ribosomal protein L18	5	1.37	0.142	8.9E-02
O00560	Syntenin-1	3	1.37	0.029	8.1E-03
P62424	60S ribosomal protein L7a	5	1.37	0.073	1.2E-02
Q13151	Heterogeneous nuclear ribonucleoprotein A0	4	1.37	0.065	1.7E-02
Q9ULV4	Coronin-1C	2	1.37	NA	NA
O95747	Serine/threonine-protein kinase OSR1	1	1.37	NA	NA
Q86TX2	Acyl-coenzyme A thioesterase 1	2	1.36	NA	NA
Q6UXV4	Apolipoprotein O-like	1	1.36	NA	NA
Q06136	3-ketodihydrosphingosine reductase	1	1.36	NA	NA
Q8NBJ7	Sulfatase-modifying factor 2	1	1.36	NA	NA
P61981	14-3-3 protein gamma	3	1.36	0.056	3.2E-02
P08574	Cytochrome c1, heme protein, mitochondrial	2	1.36	NA	NA
P55265	Double-stranded RNA-specific adenosine deaminase	3	1.36	0.145	1.7E-01
Q14738	Serine/threonine-protein phosphatase 2A 56 kDa regulatory subunit delta isoform	1	1.36	NA	NA
P53634	Dipeptidyl peptidase 1	1	1.36	NA	NA
Q99879	Histone H2B type 1-M	1	1.36	NA	NA
Q05519	Serine/arginine-rich splicing factor 11	1	1.35	NA	NA
P62249	40S ribosomal protein S16	5	1.35	0.052	4.2E-03
O60262	Guanine nucleotide-binding protein G(I)/G(S)/G(O) subunit gamma-7	1	1.35	NA	NA
P28062	Proteasome subunit beta type-8	1	1.35	NA	NA
P30084	Enoyl-CoA hydratase, mitochondrial	3	1.35	0.113	1.2E-01
P26599	Polypyrimidine tract-binding protein 1	4	1.34	0.127	1.0E-01
P29692	Elongation factor 1-delta	5	1.34	0.131	8.6E-02
P50502	Hsc70-interacting protein	4	1.34	0.108	7.2E-02
Q9UJU6	Drebrin-like protein	1	1.34	NA	NA
Q14165	Malectin	5	1.34	0.091	3.2E-02
Q9Y2B0	Protein canopy homolog 2	4	1.34	0.187	2.1E-01
P35268	60S ribosomal protein L22	1	1.34	NA	NA
P51532	Transcription activator BRG1	1	1.34	NA	NA
O43809	Cleavage and polyadenylation specificity factor subunit 5	3	1.34	0.163	2.2E-01
P14678	Small nuclear ribonucleoprotein-associated proteins B and B'	1	1.34	NA	NA
P26641	Elongation factor 1-gamma	5	1.34	0.091	3.2E-02
Q9GZY8	Mitochondrial fission factor	2	1.34	NA	NA
P61353	60S ribosomal protein L27	5	1.34	0.101	4.5E-02
P51148	Ras-related protein Rab-5C	1	1.33	NA	NA
Q5JRX3	Presequence protease, mitochondrial	3	1.33	0.213	3.1E-01
P19971	Thymidine phosphorylase	1	1.33	NA	NA
O95831	Apoptosis-inducing factor 1, mitochondrial	1	1.33	NA	NA
Q96FW1	Ubiquitin thioesterase OTUB1	2	1.33	NA	NA
P14866	Heterogeneous nuclear ribonucleoprotein L	5	1.32	0.107	5.8E-02
P13010	X-ray repair cross-complementing protein 5	5	1.32	0.053	6.2E-03
P62269	40S ribosomal protein S18	5	1.32	0.084	2.9E-02
Q8TAQ2	SWI/SNF complex subunit SMARCC2	2	1.32	NA	NA
P46778	60S ribosomal protein L21	3	1.32	0.107	1.2E-01
Q9NSE4	Isoleucine--tRNA ligase, mitochondrial	4	1.32	0.166	2.0E-01
P09874	Poly [ADP-ribose] polymerase 1	5	1.31	0.108	6.5E-02
B5ME19	Eukaryotic translation initiation factor 3 subunit C-like protein	5	1.31	0.122	9.0E-02
Q9BZQ8	Protein Niban	4	1.31	0.123	1.1E-01
O14737	Programmed cell death protein 5	1	1.31	NA	NA
P56192	Methionine--tRNA ligase, cytoplasmic	2	1.31	NA	NA
P62913	60S ribosomal protein L11	4	1.31	0.062	2.3E-02
O14561	Acyl carrier protein, mitochondrial	4	1.31	0.124	1.2E-01
Q86VP6	Cullin-associated NEDD8-dissociated protein 1	5	1.31	0.108	6.8E-02
Q8IY95	Transmembrane protein 192	1	1.31	NA	NA
P31040	Succinate dehydrogenase [ubiquinone] flavoprotein subunit, mitochondrial	5	1.30	0.068	1.8E-02
Q9NPA0	ER membrane protein complex subunit 7	1	1.30	NA	NA
P26640	Valine--tRNA ligase	4	1.30	0.090	6.1E-02
P61313	60S ribosomal protein L15	2	1.30	NA	NA
P14854	Cytochrome c oxidase subunit 6B1	5	1.30	0.081	3.2E-02
Q9HD20	Manganese-transporting ATPase 13A1	2	1.30	NA	NA
P69905	Hemoglobin subunit alpha	5	1.30	0.161	1.8E-01
P60866	40S ribosomal protein S20	5	1.30	0.119	9.6E-02
P11216	Glycogen phosphorylase, brain form	5	1.29	0.117	9.5E-02
Q02878	60S ribosomal protein L6	5	1.29	0.119	1.0E-01
Q16531	DNA damage-binding protein 1	3	1.29	0.041	2.5E-02
P26885	Peptidyl-prolyl cis-trans isomerase FKBP2	2	1.29	NA	NA
P61204	ADP-ribosylation factor 3	3	1.29	0.116	1.6E-01
Q8NBJ5	Procollagen galactosyltransferase 1	1	1.29	NA	NA
Q12913	Receptor-type tyrosine-protein phosphatase eta	3	1.29	0.178	2.9E-01
P10644	cAMP-dependent protein kinase type I-alpha regulatory subunit	5	1.29	0.115	9.4E-02
Q8NC56	LEM domain-containing protein 2	4	1.28	0.029	3.1E-03

P11142	Heat shock cognate 71 kDa protein	5	1.28	0.065	1.8E-02
Q9NX40	OCIA domain-containing protein 1	1	1.28	NA	NA
P20700	Lamin-B1	5	1.28	0.052	8.7E-03
P36404	ADP-ribosylation factor-like protein 2	1	1.28	NA	NA
P30740	Leukocyte elastase inhibitor	4	1.28	0.112	1.1E-01
Q9H0U4	Ras-related protein Rab-1B	1	1.28	NA	NA
P15586	N-acetylglucosamine-6-sulfatase	3	1.28	0.276	4.7E-01
Q92841	Probable ATP-dependent RNA helicase DDX17	5	1.28	0.028	9.5E-04
P07203	Glutathione peroxidase 1	2	1.28	NA	NA
O14745	Na(+)/H(+) exchange regulatory cofactor NHE-RF1	2	1.28	NA	NA
P46439	Glutathione S-transferase Mu 5	1	1.27	NA	NA
P02042	Hemoglobin subunit delta	5	1.27	0.229	3.5E-01
O75396	Vesicle-trafficking protein SEC22b	3	1.27	0.107	1.5E-01
Q9Y4I1	Unconventional myosin-Va	4	1.27	0.085	6.7E-02
Q00341	Vigilin	2	1.27	NA	NA
Q07666	KH domain-containing, RNA-binding, signal transduction-associated protein 1	5	1.27	0.096	6.7E-02
Q6UVK1	Chondroitin sulfate proteoglycan 4	4	1.27	0.234	3.8E-01
O75947	ATP synthase subunit d, mitochondrial	5	1.27	0.065	2.2E-02
P21912	Succinate dehydrogenase [ubiquinone] iron-sulfur subunit, mitochondrial	5	1.27	0.120	1.2E-01
P01859	Ig gamma-2 chain C region	5	1.27	0.192	2.8E-01
P12956	X-ray repair cross-complementing protein 6	5	1.27	0.049	8.2E-03
P52272	Heterogeneous nuclear ribonucleoprotein M	5	1.27	0.092	6.2E-02
P34932	Heat shock 70 kDa protein 4	5	1.27	0.073	3.2E-02
Q13011	Delta(3,5)-Delta(2,4)-dienoyl-CoA isomerase, mitochondrial	5	1.27	0.142	1.7E-01
P00505	Aspartate aminotransferase, mitochondrial	5	1.27	0.089	5.7E-02
P20340	Ras-related protein Rab-6A	1	1.26	NA	NA
Q9NVI7	ATPase family AAA domain-containing protein 3A	1	1.26	NA	NA
P78347	General transcription factor II-I	4	1.26	0.085	7.1E-02
P09669	Cytochrome c oxidase subunit 6C	5	1.26	0.098	7.7E-02
P49821	NADH dehydrogenase [ubiquinone] flavoprotein 1, mitochondrial	2	1.26	NA	NA
P38117	Electron transfer flavoprotein subunit beta	5	1.26	0.029	1.3E-03
Q16762	Thiosulfate sulfurtransferase	1	1.26	NA	NA
P61421	V-type proton ATPase subunit d 1	4	1.26	0.112	1.3E-01
O43598	2'-deoxynucleoside 5'-phosphate N-hydrolase 1	2	1.26	NA	NA
P38606	V-type proton ATPase catalytic subunit A	5	1.25	0.201	3.2E-01
O15144	Actin-related protein 2/3 complex subunit 2	5	1.25	0.048	9.2E-03
P22061	Protein-L-isoaspartate(D-aspartate) O-methyltransferase	1	1.25	NA	NA
Q9Y2J2	Band 4.1-like protein 3	2	1.25	NA	NA
Q8NBS9	Thioredoxin domain-containing protein 5	5	1.25	0.126	1.5E-01
P19367	Hexokinase-1	4	1.25	0.214	3.7E-01
P40429	60S ribosomal protein L13a	5	1.25	0.069	3.3E-02
P50213	Isocitrate dehydrogenase [NAD] subunit alpha, mitochondrial	5	1.25	0.134	1.7E-01
P50897	Palmitoyl-protein thioesterase 1	1	1.25	NA	NA
Q9Y5X3	Sorting nexin-5	1	1.25	NA	NA
P62899	60S ribosomal protein L31	3	1.25	0.139	2.5E-01
O75367	Core histone macro-H2A.1	5	1.24	0.048	1.1E-02
P49411	Elongation factor Tu, mitochondrial	5	1.24	0.063	2.6E-02
Q9BR76	Coronin-1B	4	1.24	0.101	1.2E-01
P04233	HLA class II histocompatibility antigen gamma chain	1	1.24	NA	NA
P01625	Ig kappa chain V-IV region Len	2	1.24	NA	NA
P05198	Eukaryotic translation initiation factor 2 subunit 1	3	1.24	0.110	1.9E-01
O75489	NADH dehydrogenase [ubiquinone] iron-sulfur protein 3, mitochondrial	5	1.24	0.037	4.3E-03
Q9UHQ9	NADH-cytochrome b5 reductase 1	5	1.24	0.072	4.1E-02
P62753	40S ribosomal protein S6	5	1.24	0.063	2.8E-02
P0C0S8	Histone H2A type 1	3	1.23	0.213	4.3E-01
P29728	2'-5'-oligoadenylate synthase 2	1	1.23	NA	NA
P49755	Transmembrane emp24 domain-containing protein 10	3	1.23	0.068	9.1E-02
O60506	Heterogeneous nuclear ribonucleoprotein Q	5	1.23	0.112	1.4E-01
P20810	Calpastatin	2	1.22	NA	NA
Q96JB5	CDK5 regulatory subunit-associated protein 3	2	1.22	NA	NA
P0CG29	Glutathione S-transferase theta-2	1	1.22	NA	NA
Q14974	Importin subunit beta-1	5	1.22	0.143	2.3E-01
Q13459	Unconventional myosin-IXb	1	1.22	NA	NA
P35998	26S protease regulatory subunit 7	3	1.22	0.096	1.8E-01
P08107	Heat shock 70 kDa protein 1A/1B	5	1.22	0.178	3.3E-01
P13796	Plastin-2	2	1.22	NA	NA
P14314	Glucosidase 2 subunit beta	5	1.22	0.133	2.2E-01
O43390	Heterogeneous nuclear ribonucleoprotein R	5	1.21	0.151	2.7E-01
Q14847	LIM and SH3 domain protein 1	3	1.21	0.195	4.2E-01
P49257	Protein ERGIC-53	2	1.21	NA	NA
Q05397	Focal adhesion kinase 1	1	1.21	NA	NA
Q9Y5K6	CD2-associated protein	1	1.21	NA	NA
Q99653	Calcineurin B homologous protein 1	2	1.21	NA	NA
O15143	Actin-related protein 2/3 complex subunit 1B	2	1.21	NA	NA
P23786	Carnitine O-palmitoyltransferase 2, mitochondrial	1	1.21	NA	NA
Q02252	Methylmalonate-semialdehyde dehydrogenase [acylating], mitochondrial	5	1.21	0.076	6.8E-02
P49591	Serine--tRNA ligase, cytoplasmic	4	1.21	0.094	1.4E-01
P43686	26S protease regulatory subunit 6B	4	1.20	0.041	2.0E-02
P09622	Dihydrolipoyl dehydrogenase, mitochondrial	5	1.20	0.087	1.0E-01
P45954	Short/branched chain specific acyl-CoA dehydrogenase, mitochondrial	2	1.20	NA	NA
Q9P2E9	Ribosome-binding protein 1	5	1.20	0.055	2.9E-02
Q9P0K7	Ankycorbin	1	1.20	NA	NA
P14868	Aspartate--tRNA ligase, cytoplasmic	5	1.20	0.102	1.5E-01
Q13409	Cytoplasmic dynein 1 intermediate chain 2	1	1.20	NA	NA
P06730	Eukaryotic translation initiation factor 4E	1	1.20	NA	NA
O43852	Calumenin	4	1.20	0.094	1.5E-01
P31949	Protein S100-A11	4	1.20	0.223	4.8E-01
Q02818	Nucleobindin-1	5	1.20	0.156	3.1E-01
Q14152	Eukaryotic translation initiation factor 3 subunit A	5	1.20	0.096	1.4E-01
Q03519	Antigen peptide transporter 2	1	1.19	NA	NA
P36578	60S ribosomal protein L4	5	1.19	0.099	1.5E-01
P53990	IST1 homolog	1	1.19	NA	NA
O95202	LETM1 and EF-hand domain-containing protein 1, mitochondrial	4	1.19	0.133	2.8E-01
P37235	Hippocalcin-like protein 1	1	1.19	NA	NA
P01857	Ig gamma-1 chain C region	5	1.19	0.162	3.5E-01
P32969	60S ribosomal protein L9	5	1.19	0.071	7.2E-02
Q96BM9	ADP-ribosylation factor-like protein 8A	2	1.19	NA	NA
Q9Y262	Eukaryotic translation initiation factor 3 subunit L	4	1.18	0.083	1.3E-01
P51572	B-cell receptor-associated protein 31	5	1.18	0.108	1.9E-01
P23396	40S ribosomal protein S3	5	1.18	0.084	1.2E-01
P62277	40S ribosomal protein S13	5	1.18	0.095	1.6E-01
Q92896	Golgi apparatus protein 1	2	1.18	NA	NA
P45880	Voltage-dependent anion-selective channel protein 2	5	1.18	0.047	2.5E-02
P50402	Emerin	5	1.18	0.131	2.8E-01
Q9BZZ5	Apoptosis inhibitor 5	3	1.18	0.039	5.3E-02
P15311	Ezrin	3	1.18	0.091	2.1E-01
P02766	Transthyretin	2	1.18	NA	NA
Q5SSJ5	Heterochromatin protein 1-binding protein 3	4	1.17	0.228	5.3E-01
P61158	Actin-related protein 3	5	1.17	0.116	2.4E-01
Q99623	Prohibitin-2	5	1.17	0.068	8.2E-02
Q9Y265	RuvB-like 1	2	1.17	NA	NA
P17844	Probable ATP-dependent RNA helicase DDX5	5	1.17	0.104	2.1E-01
P38919	Eukaryotic initiation factor 4A-III	3	1.17	0.188	5.0E-01
P46779	60S ribosomal protein L28	2	1.17	NA	NA
O14617	AP-3 complex subunit delta-1	1	1.16	NA	NA
Q06830	Peroxiredoxin-1	5	1.16	0.130	3.1E-01
Q86UP2	Kinectin	5	1.16	0.113	2.5E-01
O14949	Cytochrome b-c1 complex subunit 8	1	1.16	NA	NA
P15880	40S ribosomal protein S2	5	1.16	0.102	2.2E-01
P48047	ATP synthase subunit O, mitochondrial	5	1.16	0.055	5.7E-02
P37108	Signal recognition particle 14 kDa protein	4	1.16	0.110	2.8E-01
P30101	Protein disulfide-isomerase A3	5	1.15	0.145	3.8E-01
P30048	Thioredoxin-dependent peroxide reductase, mitochondrial	5	1.15	0.127	3.3E-01
Q5JWF2	Guanine nucleotide-binding protein G(s) subunit alpha isoforms XLas	2	1.15	NA	NA
P17858	ATP-dependent 6-phosphofructokinase, liver type	4	1.15	0.062	1.1E-01
P49327	Fatty acid synthase	2	1.15	NA	NA
P78371	T-complex protein 1 subunit beta	5	1.15	0.057	7.0E-02
P11310	Medium-chain specific acyl-CoA dehydrogenase, mitochondrial	3	1.15	0.078	2.2E-01
P12236	ADP/ATP translocase 3	2	1.14	NA	NA
Q15008	26S proteasome non-ATPase regulatory subunit 6	2	1.14	NA	NA
P46782	40S ribosomal protein S5	4	1.14	0.094	2.5E-01
Q8N0X7	Spartin	1	1.14	NA	NA
P13804	Electron transfer flavoprotein subunit alpha, mitochondrial	1	1.14	NA	NA
A1L0T0	Acetolactate synthase-like protein	2	1.14	NA	NA
P06576	ATP synthase subunit beta, mitochondrial	5	1.14	0.079	1.7E-01
Q15691	Microtubule-associated protein RP/EB family member 1	4	1.14	0.123	3.6E-01
P31146	Coronin-1A	2	1.14	NA	NA
Q9P0L0	Vesicle-associated membrane protein-associated protein A	3	1.14	0.308	7.1E-01
Q99873	Protein arginine N-methyltransferase 1	2	1.14	NA	NA
P23284	Peptidyl-prolyl cis-trans isomerase B	5	1.14	0.218	5.9E-01
P36873	Serine/threonine-protein phosphatase PP1-gamma catalytic subunit	1	1.14	NA	NA
Q13283	Ras GTPase-activating protein-binding protein 1	3	1.14	0.070	2.1E-01
Q9NTK5	Obg-like ATPase 1	2	1.13	NA	NA
P59998	Actin-related protein 2/3 complex subunit 4	5	1.13	0.086	2.2E-01
P51665	26S proteasome non-ATPase regulatory subunit 7	3	1.13	0.078	2.5E-01
Q9Y5M8	Signal recognition particle receptor subunit beta	2	1.13	NA	NA
O14980	Exportin-1	2	1.13	NA	NA
P27797	Calreticulin	5	1.13	0.105	3.0E-01
Q96I24	Far upstream element-binding protein 3	1	1.13	NA	NA
P27708	CAD protein	3	1.13	0.028	4.7E-02
P30153	Serine/threonine-protein phosphatase 2A 65 kDa regulatory subunit A alpha isoform	5	1.13	0.062	1.2E-01
P21281	V-type proton ATPase subunit B, brain isoform	5	1.13	0.152	4.6E-01
P50454	Serpin H1	5	1.13	0.075	1.8E-01
P36543	V-type proton ATPase subunit E 1	3	1.13	0.200	6.0E-01
O95716	Ras-related protein Rab-3D	1	1.13	NA	NA
P11586	C-1-tetrahydrofolate synthase, cytoplasmic	5	1.13	0.063	1.3E-01
O14773	Tripeptidyl-peptidase 1	5	1.13	0.201	5.8E-01
P20645	Cation-dependent mannose-6-phosphate receptor	3	1.13	0.103	3.7E-01
P62081	40S ribosomal protein S7	2	1.13	NA	NA
Q13423	NAD(P) transhydrogenase, mitochondrial	5	1.13	0.177	5.4E-01
P02545	Prelamin-A/C	5	1.13	0.120	3.8E-01
O60493	Sorting nexin-3	2	1.12	NA	NA
O00231	26S proteasome non-ATPase regulatory subunit 11	5	1.12	0.100	3.2E-01
P46109	Crk-like protein	1	1.12	NA	NA
P62318	Small nuclear ribonucleoprotein Sm D3	4	1.12	0.074	2.2E-01
P54136	Arginine--tRNA ligase, cytoplasmic	4	1.12	0.098	3.3E-01
P30533	Alpha-2-macroglobulin receptor-associated protein	3	1.12	0.153	5.4E-01
P39023	60S ribosomal protein L3	5	1.12	0.057	1.2E-01
Q13310	Polyadenylate-binding protein 4	2	1.12	NA	NA
Q16718	NADH dehydrogenase [ubiquinone] 1 alpha subcomplex subunit 5	1	1.12	NA	NA
Q86WV6	Stimulator of interferon genes protein	1	1.12	NA	NA
Q15717	ELAV-like protein 1	5	1.12	0.141	4.8E-01
Q99442	Translocation protein SEC62	4	1.12	0.070	2.1E-01
P23368	NAD-dependent malic enzyme, mitochondrial	1	1.12	NA	NA
P18124	60S ribosomal protein L7	5	1.12	0.079	2.4E-01
P02458	Collagen alpha-1(II) chain	1	1.11	NA	NA
P11387	DNA topoisomerase 1	1	1.11	NA	NA
O60486	Plexin-C1	3	1.11	0.043	1.3E-01
Q53H12	Acylglycerol kinase, mitochondrial	1	1.11	NA	NA
Q9UIJ7	GTP:AMP phosphotransferase AK3, mitochondrial	5	1.11	0.121	4.3E-01
P05388	60S acidic ribosomal protein P0	5	1.11	0.106	3.8E-01
P78527	DNA-dependent protein kinase catalytic subunit	5	1.11	0.073	2.3E-01
O75306	NADH dehydrogenase [ubiquinone] iron-sulfur protein 2, mitochondrial	5	1.11	0.081	2.7E-01
P61006	Ras-related protein Rab-8A	1	1.11	NA	NA
P09012	U1 small nuclear ribonucleoprotein A	4	1.10	0.212	6.8E-01
Q02543	60S ribosomal protein L18a	4	1.10	0.051	1.6E-01
P28838	Cytosol aminopeptidase	4	1.10	0.167	6.1E-01

Table S17-Average Non-Metastatic Tumors

P10606	Cytochrome c oxidase subunit 5B, mitochondrial	5	1.10	0.100	4.1E-01
Q13596	Sorting nexin-1	4	1.10	0.117	4.9E-01
P30050	60S ribosomal protein L12	3	1.10	0.091	4.2E-01
P25705	ATP synthase subunit alpha, mitochondrial	5	1.09	0.076	3.0E-01
Q9BXP5	Serrate RNA effector molecule homolog	1	1.09	NA	NA
P54709	Sodium/potassium-transporting ATPase subunit beta-3	5	1.09	0.059	2.2E-01
P08237	ATP-dependent 6-phosphofructokinase, muscle type	5	1.09	0.077	3.3E-01
P11177	Pyruvate dehydrogenase E1 component subunit beta, mitochondrial	4	1.09	0.067	3.0E-01
Q9Y230	RuvB-like 2	4	1.09	0.122	5.4E-01
P62917	60S ribosomal protein L8	4	1.09	0.127	5.6E-01
Q92598	Heat shock protein 105 kDa	2	1.09	NA	NA
Q15233	Non-POU domain-containing octamer-binding protein	5	1.09	0.110	5.0E-01
P43490	Nicotinamide phosphoribosyltransferase	3	1.08	0.109	5.3E-01
O75340	Programmed cell death protein 6	2	1.08	NA	NA
P13073	Cytochrome c oxidase subunit 4 isoform 1, mitochondrial	5	1.08	0.121	5.7E-01
O15173	Membrane-associated progesterone receptor component 2	4	1.08	0.058	2.9E-01
P62979	Ubiquitin-40S ribosomal protein S27a	2	1.08	NA	NA
P18077	60S ribosomal protein L35a	2	1.08	NA	NA
Q96A33	Coiled-coil domain-containing protein 47	1	1.07	NA	NA
O95881	Thioredoxin domain-containing protein 12	2	1.07	NA	NA
Q13228	Selenium-binding protein 1	4	1.07	0.212	7.6E-01
P28331	NADH-ubiquinone oxidoreductase 75 kDa subunit, mitochondrial	3	1.07	0.111	5.9E-01
Q14697	Neutral alpha-glucosidase AB	5	1.07	0.118	6.1E-01
P46940	Ras GTPase-activating-like protein IQGAP1	5	1.07	0.152	7.0E-01
Q13347	Eukaryotic translation initiation factor 3 subunit I	1	1.06	NA	NA
O60664	Perilipin-3	3	1.06	0.086	5.5E-01
P36542	ATP synthase subunit gamma, mitochondrial	1	1.06	NA	NA
O00303	Eukaryotic translation initiation factor 3 subunit F	2	1.06	NA	NA
O94906	Pre-mRNA-processing factor 6	1	1.06	NA	NA
P42167	Lamina-associated polypeptide 2, isoforms beta/gamma	5	1.06	0.229	8.2E-01
P42858	Huntingtin	1	1.06	NA	NA
O60568	Procollagen-lysine,2-oxoglutarate 5-dioxygenase 3	1	1.05	NA	NA
P62701	40S ribosomal protein S4, X isoform	5	1.05	0.104	6.4E-01
P29590	Protein PML	4	1.05	0.273	8.6E-01
O95298	NADH dehydrogenase [ubiquinone] 1 subunit C2	1	1.05	NA	NA
O75323	Protein NipSnap homolog 2	2	1.05	NA	NA
Q7Z7H5	Transmembrane emp24 domain-containing protein 4	1	1.05	NA	NA
O75964	ATP synthase subunit g, mitochondrial	5	1.05	0.059	4.4E-01
P56556	NADH dehydrogenase [ubiquinone] 1 alpha subcomplex subunit 6	1	1.05	NA	NA
Q14980	Nuclear mitotic apparatus protein 1	5	1.05	0.036	2.3E-01
Q8WUD1	Ras-related protein Rab-2B	1	1.05	NA	NA
P63000	Ras-related C3 botulinum toxin substrate 1	4	1.05	0.064	5.0E-01
O75643	U5 small nuclear ribonucleoprotein 200 kDa helicase	4	1.05	0.127	7.3E-01
P51531	Probable global transcription activator SNF2L2	1	1.05	NA	NA
Q27J81	Inverted formin-2	3	1.05	0.127	7.5E-01
Q15365	Poly(rC)-binding protein 1	5	1.05	0.178	8.1E-01
Q9H3N1	Thioredoxin-related transmembrane protein 1	1	1.05	NA	NA
Q96AE4	Far upstream element-binding protein 1	4	1.04	0.062	5.3E-01
Q8N5K1	CDGSH iron-sulfur domain-containing protein 2	5	1.04	0.083	6.3E-01
P22307	Non-specific lipid-transfer protein	2	1.04	NA	NA
P01860	Ig gamma-3 chain C region	5	1.04	0.121	7.4E-01
Q6P2Q9	Pre-mRNA-processing-splicing factor 8	2	1.04	NA	NA
P22059	Oxysterol-binding protein 1	1	1.04	NA	NA
P57053	Histone H2B type F-S	2	1.04	NA	NA
P24752	Acetyl-CoA acetyltransferase, mitochondrial	5	1.04	0.115	7.6E-01
P01023	Alpha-2-macroglobulin	5	1.04	0.089	7.0E-01
P46977	Dolichyl-diphosphooligosaccharide--protein glycosyltransferase subunit STT3A	3	1.03	0.150	8.4E-01
P43304	Glycerol-3-phosphate dehydrogenase, mitochondrial	5	1.03	0.164	8.5E-01
P46781	40S ribosomal protein S9	5	1.03	0.070	6.6E-01
P61019	Ras-related protein Rab-2A	1	1.03	NA	NA
Q01844	RNA-binding protein EWS	3	1.03	0.073	7.0E-01
P11940	Polyadenylate-binding protein 1	5	1.03	0.030	3.6E-01
Q99829	Copine-1	1	1.03	NA	NA
Q9Y394	Dehydrogenase/reductase SDR family member 7	1	1.03	NA	NA
P22695	Cytochrome b-c1 complex subunit 2, mitochondrial	5	1.02	0.118	8.4E-01
P62987	Ubiquitin-60S ribosomal protein L40	3	1.02	0.042	6.2E-01
P61160	Actin-related protein 2	5	1.02	0.031	4.8E-01
O43681	ATPase ASNA1	3	1.02	0.282	9.4E-01
P04440	HLA class II histocompatibility antigen, DP beta 1 chain	1	1.02	NA	NA
P07237	Protein disulfide-isomerase	5	1.02	0.082	8.2E-01
P49588	Alanine--tRNA ligase, cytoplasmic	1	1.02	NA	NA
P26368	Splicing factor U2AF 65 kDa subunit	4	1.02	0.085	8.4E-01
P01889	HLA class I histocompatibility antigen, B-7 alpha chain	1	1.02	NA	NA
Q15393	Splicing factor 3B subunit 3	2	1.02	NA	NA
Q8N1G4	Leucine-rich repeat-containing protein 47	4	1.02	0.245	9.5E-01
P30519	Heme oxygenase 2	4	1.01	0.101	8.9E-01
P11498	Pyruvate carboxylase, mitochondrial	2	1.01	NA	NA
P36955	Pigment epithelium-derived factor	1	1.01	NA	NA
Q9UHV9	Prefoldin subunit 2	2	1.01	NA	NA
P51398	28S ribosomal protein S29, mitochondrial	1	1.01	NA	NA
Q9BRX8	Redox-regulatory protein FAM213A	5	1.01	0.090	9.2E-01
Q9NZ08	Endoplasmic reticulum aminopeptidase 1	2	1.01	NA	NA
P20674	Cytochrome c oxidase subunit 5A, mitochondrial	5	1.01	0.135	9.5E-01
Q8IYW2	Tetratricopeptide repeat protein 40	1	1.01	NA	NA
Q9NTZ6	RNA-binding protein 12	2	1.00	NA	NA
Q15293	Reticulocalbin-1	5	1.00	0.247	9.9E-01
P62244	40S ribosomal protein S15a	4	1.00	0.058	9.5E-01
Q9HCC0	Methylcrotonoyl-CoA carboxylase beta chain, mitochondrial	1	1.00	NA	NA
Q6IAA8	Ragulator complex protein LAMTOR1	2	1.00	NA	NA
Q7KZF4	Staphylococcal nuclease domain-containing protein 1	5	1.00	0.077	9.8E-01
Q9NTJ5	Phosphatidylinositide phosphatase SAC1	4	1.00	0.134	9.9E-01
Q13162	Peroxiredoxin-4	5	1.00	0.126	9.9E-01
Q15084	Protein disulfide-isomerase A6	5	1.00	0.118	9.9E-01
Q14789	Golgin subfamily B member 1	1	1.00	NA	NA
O00410	Importin-5	1	1.00	NA	NA
Q9P2R7	Succinyl-CoA ligase [ADP-forming] subunit beta, mitochondrial	4	1.00	0.076	9.7E-01
Q99714	3-hydroxyacyl-CoA dehydrogenase type-2	3	0.99	0.150	9.8E-01
Q15029	116 kDa U5 small nuclear ribonucleoprotein component	2	0.99	NA	NA
P61225	Ras-related protein Rap-2b	4	0.99	0.071	9.2E-01
P42356	Phosphatidylinositol 4-kinase alpha	1	0.99	NA	NA
P35637	RNA-binding protein FUS	4	0.99	0.138	9.5E-01
O75521	Enoyl-CoA delta isomerase 2, mitochondrial	3	0.99	0.129	9.5E-01
P68871	Hemoglobin subunit beta	5	0.99	0.240	9.7E-01
Q9Y3U8	60S ribosomal protein L36	5	0.99	0.063	8.8E-01
Q16851	UTP--glucose-1-phosphate uridylyltransferase	3	0.99	0.159	9.3E-01
P29966	Myristoylated alanine-rich C-kinase substrate	5	0.98	0.101	8.8E-01
Q9BW27	Nuclear pore complex protein Nup85	1	0.98	NA	NA
P47897	Glutamine--tRNA ligase	1	0.98	NA	NA
Q9Y512	Sorting and assembly machinery component 50 homolog	1	0.98	NA	NA
P43243	Matrin-3	2	0.98	NA	NA
P62316	Small nuclear ribonucleoprotein Sm D2	2	0.98	NA	NA
P13473	Lysosome-associated membrane glycoprotein 2	2	0.98	NA	NA
P05107	Integrin beta-2	3	0.98	0.245	9.4E-01
P61026	Ras-related protein Rab-10	5	0.98	0.075	7.8E-01
P36957	Dihydrolipoyllysine-residue succinyltransferase component of 2-oxoglutarate dehydrogenase complex, mitochondrial	5	0.98	0.115	8.5E-01
P14927	Cytochrome b-c1 complex subunit 7	5	0.98	0.068	7.4E-01
O95302	Peptidyl-prolyl cis-trans isomerase FKBP9	2	0.97	NA	NA
P42765	3-ketoacyl-CoA thiolase, mitochondrial	5	0.97	0.080	7.4E-01
P08559	Pyruvate dehydrogenase E1 component subunit alpha, somatic form, mitochondrial	3	0.97	0.113	8.2E-01
Q99805	Transmembrane 9 superfamily member 2	1	0.97	NA	NA
P60228	Eukaryotic translation initiation factor 3 subunit E	2	0.97	NA	NA
Q8IVF2	Protein AHNAK2	2	0.97	NA	NA
P62333	26S protease regulatory subunit 10B	1	0.97	NA	NA
P08134	Rho-related GTP-binding protein RhoC	3	0.96	0.181	8.6E-01
P17931	Galectin-3	5	0.96	0.337	9.1E-01
P17980	26S protease regulatory subunit 6A	3	0.96	0.200	8.6E-01
P31689	DnaJ homolog subfamily A member 1	3	0.96	0.077	6.6E-01
Q9C0E8	Protein lunapark	1	0.96	NA	NA
Q96QK1	Vacuolar protein sorting-associated protein 35	3	0.96	0.025	2.4E-01
P47985	Cytochrome b-c1 complex subunit Rieske, mitochondrial	5	0.96	0.119	7.5E-01
P11021	78 kDa glucose-regulated protein	5	0.96	0.068	5.8E-01
P55884	Eukaryotic translation initiation factor 3 subunit B	4	0.96	0.148	7.9E-01
P40227	T-complex protein 1 subunit zeta	5	0.96	0.108	7.1E-01
P53618	Coatomer subunit beta	4	0.95	0.088	6.3E-01
Q9UNH7	Sorting nexin-6	4	0.95	0.072	5.7E-01
Q14108	Lysosome membrane protein 2	4	0.95	0.199	8.3E-01
Q96C19	EF-hand domain-containing protein D2	1	0.95	NA	NA
P60763	Ras-related C3 botulinum toxin substrate 3	1	0.95	NA	NA
P10515	Dihydrolipoyllysine-residue acetyltransferase component of pyruvate dehydrogenase complex, mitochondrial	4	0.95	0.045	3.7E-01
Q9NYU2	UDP-glucose:glycoprotein glucosyltransferase 1	3	0.95	0.122	7.3E-01
Q16891	Mitochondrial inner membrane protein	5	0.95	0.105	6.6E-01
P26038	Moesin	5	0.95	0.080	5.7E-01
P27824	Calnexin	5	0.95	0.122	7.0E-01
P78344	Eukaryotic translation initiation factor 4 gamma 2	1	0.95	NA	NA
Q9UMX5	Neudesin	2	0.95	NA	NA
P27144	Adenylate kinase 4, mitochondrial	1	0.95	NA	NA
Q16698	2,4-dienoyl-CoA reductase, mitochondrial	4	0.95	0.239	8.3E-01
P48681	Nestin	5	0.95	0.328	8.7E-01
Q00577	Transcriptional activator protein Pur-alpha	2	0.95	NA	NA
Q96TC7	Regulator of microtubule dynamics protein 3	1	0.94	NA	NA
Q9BXX0	EMILIN-2	1	0.94	NA	NA
Q14683	Structural maintenance of chromosomes protein 1A	5	0.94	0.038	1.8E-01
Q9BVK6	Transmembrane emp24 domain-containing protein 9	1	0.94	NA	NA
P61586	Transforming protein RhoA	2	0.94	NA	NA
Q96JJ7	Protein disulfide-isomerase TMX3	1	0.94	NA	NA
P35606	Coatomer subunit beta'	1	0.94	NA	NA
Q13825	Methylglutaconyl-CoA hydratase, mitochondrial	1	0.94	NA	NA
Q16795	NADH dehydrogenase [ubiquinone] 1 alpha subcomplex subunit 9, mitochondrial	3	0.93	0.053	3.3E-01
Q93050	V-type proton ATPase 116 kDa subunit a isoform 1	4	0.93	0.277	8.2E-01
O75165	DnaJ homolog subfamily C member 13	2	0.93	NA	NA
P62888	60S ribosomal protein L30	1	0.93	NA	NA
Q9Y6G9	Cytoplasmic dynein 1 light intermediate chain 1	2	0.93	NA	NA
Q9UBQ0	Vacuolar protein sorting-associated protein 29	1	0.93	NA	NA
Q9P0J0	NADH dehydrogenase [ubiquinone] 1 alpha subcomplex subunit 13	1	0.92	NA	NA
P31943	Heterogeneous nuclear ribonucleoprotein H	2	0.92	NA	NA
P46976	Glycogenin-1	1	0.92	NA	NA
P49368	T-complex protein 1 subunit gamma	5	0.92	0.058	2.1E-01
P61457	Pterin-4-alpha-carbinolamine dehydratase	1	0.92	NA	NA
P0CG05	Ig lambda-2 chain C regions	4	0.92	0.271	7.7E-01
Q7Z6Z7	E3 ubiquitin-protein ligase HUWE1	2	0.91	NA	NA
P11279	Lysosome-associated membrane glycoprotein 1	1	0.91	NA	NA
O75915	PRA1 family protein 3	4	0.91	0.265	7.5E-01
Q8IUX7	Adipocyte enhancer-binding protein 1	1	0.91	NA	NA
Q15435	Protein phosphatase 1 regulatory subunit 7	1	0.91	NA	NA
P50990	T-complex protein 1 subunit theta	5	0.91	0.082	2.9E-01
O60763	General vesicular transport factor p115	1	0.91	NA	NA
Q9Y224	UPF0568 protein C14orf166	1	0.90	NA	NA
P02774	Vitamin D-binding protein	4	0.90	0.167	5.9E-01
Q6P587	Acylpyruvase FAHD1, mitochondrial	1	0.90	NA	NA
Q13200	26S proteasome non-ATPase regulatory subunit 2	5	0.90	0.102	3.7E-01
P62805	Histone H4	5	0.90	0.062	1.6E-01
P55084	Trifunctional enzyme subunit beta, mitochondrial	5	0.90	0.255	7.0E-01
Q16695	Histone H3.1t	4	0.90	0.192	6.1E-01
O43242	26S proteasome non-ATPase regulatory subunit 3	4	0.89	0.047	9.6E-02
P35573	Glycogen debranching enzyme	1	0.89	NA	NA
Q9UQE7	Structural maintenance of chromosomes protein 3	3	0.89	0.063	2.1E-01
Q71DI3	Histone H3.2	1	0.89	NA	NA

Table S17-Average Non-Metastatic Tumors

P63092	Guanine nucleotide-binding protein G(s) subunit alpha isoforms short	1	0.89	NA	NA
Q8IZP0	Abl interactor 1	1	0.89	NA	NA
Q9Y277	Voltage-dependent anion-selective channel protein 3	2	0.89	NA	NA
O00203	AP-3 complex subunit beta-1	2	0.89	NA	NA
P00390	Glutathione reductase, mitochondrial	5	0.89	0.193	5.7E-01
Q9Y3Z3	Deoxynucleoside triphosphate triphosphohydrolase SAMHD1	3	0.88	0.091	3.1E-01
P50991	T-complex protein 1 subunit delta	5	0.88	0.042	4.3E-02
P55072	Transitional endoplasmic reticulum ATPase	5	0.88	0.028	1.1E-02
P67870	Casein kinase II subunit beta	3	0.88	0.189	5.7E-01
Q00325	Phosphate carrier protein, mitochondrial	5	0.88	0.045	4.4E-02
P04179	Superoxide dismutase [Mn], mitochondrial	5	0.88	0.025	6.8E-03
P46459	Vesicle-fusing ATPase	2	0.88	NA	NA
Q9BS26	Endoplasmic reticulum resident protein 44	5	0.88	0.111	3.0E-01
P24539	ATP synthase F(0) complex subunit B1, mitochondrial	5	0.88	0.048	5.0E-02
P48643	T-complex protein 1 subunit epsilon	5	0.87	0.062	9.9E-02
P49756	RNA-binding protein 25	1	0.87	NA	NA
Q15075	Early endosome antigen 1	2	0.87	NA	NA
P01876	Ig alpha-1 chain C region	5	0.87	0.147	4.1E-01
P31930	Cytochrome b-c1 complex subunit 1, mitochondrial	5	0.87	0.119	3.1E-01
O60313	Dynamin-like 120 kDa protein, mitochondrial	5	0.87	0.060	7.9E-02
P09382	Galectin-1	5	0.87	0.277	6.4E-01
P09543	2',3'-cyclic-nucleotide 3'-phosphodiesterase	5	0.87	0.136	3.6E-01
P35221	Catenin alpha-1	4	0.87	0.103	2.6E-01
P49593	Protein phosphatase 1F	2	0.87	NA	NA
Q14258	E3 ubiquitin/ISG15 ligase TRIM25	3	0.87	0.113	3.3E-01
P01834	Ig kappa chain C region	5	0.86	0.160	4.2E-01
Q99832	T-complex protein 1 subunit eta	5	0.86	0.078	1.3E-01
P40939	Trifunctional enzyme subunit alpha, mitochondrial	5	0.86	0.182	4.7E-01
P00403	Cytochrome c oxidase subunit 2	2	0.86	NA	NA
O75832	26S proteasome non-ATPase regulatory subunit 10	1	0.86	NA	NA
P18859	ATP synthase-coupling factor 6, mitochondrial	3	0.86	0.090	2.3E-01
A0FGR8	Extended synaptotagmin-2	2	0.86	NA	NA
P00352	Retinal dehydrogenase 1	4	0.86	0.325	6.6E-01
Q9UHD8	Septin-9	5	0.86	0.028	5.1E-03
Q12965	Unconventional myosin-Ie	2	0.86	NA	NA
O15523	ATP-dependent RNA helicase DDX3Y	1	0.85	NA	NA
P49748	Very long-chain specific acyl-CoA dehydrogenase, mitochondrial	5	0.85	0.118	2.5E-01
Q92499	ATP-dependent RNA helicase DDX1	4	0.85	0.136	3.1E-01
P54577	Tyrosine--tRNA ligase, cytoplasmic	1	0.85	NA	NA
P04843	Dolichyl-diphosphooligosaccharide--protein glycosyltransferase subunit 1	5	0.85	0.104	1.9E-01
Q969X5	Endoplasmic reticulum-Golgi intermediate compartment protein 1	3	0.85	0.094	2.2E-01
Q53GQ0	Estradiol 17-beta-dehydrogenase 12	5	0.85	0.186	4.2E-01
P09525	Annexin A4	5	0.84	0.082	1.1E-01
Q13561	Dynactin subunit 2	5	0.84	0.086	1.2E-01
P13987	CD59 glycoprotein	5	0.84	0.144	3.0E-01
P39656	Dolichyl-diphosphooligosaccharide--protein glycosyltransferase 48 kDa subunit	5	0.84	0.073	8.0E-02
Q9UBI6	Guanine nucleotide-binding protein G(I)/G(S)/G(O) subunit gamma-12	2	0.84	NA	NA
Q7L5N1	COP9 signalosome complex subunit 6	5	0.84	0.053	3.1E-02
P60953	Cell division control protein 42 homolog	3	0.84	0.118	2.7E-01
O94925	Glutaminase kidney isoform, mitochondrial	1	0.84	NA	NA
O60841	Eukaryotic translation initiation factor 5B	1	0.84	NA	NA
P58876	Histone H2B type 1-D	1	0.84	NA	NA
P53992	Protein transport protein Sec24C	1	0.84	NA	NA
P51812	Ribosomal protein S6 kinase alpha-3	4	0.83	0.151	3.2E-01
P47756	F-actin-capping protein subunit beta	5	0.83	0.081	8.8E-02
P09496	Clathrin light chain A	5	0.83	0.044	1.4E-02
P04844	Dolichyl-diphosphooligosaccharide--protein glycosyltransferase subunit 2	5	0.83	0.108	1.7E-01
Q92542	Nicastrin	1	0.83	NA	NA
O96008	Mitochondrial import receptor subunit TOM40 homolog	1	0.83	NA	NA
Q99584	Protein S100-A13	5	0.83	0.158	3.0E-01
Q8N163	Cell cycle and apoptosis regulator protein 2	1	0.83	NA	NA
P30508	HLA class I histocompatibility antigen, Cw-12 alpha chain	1	0.83	NA	NA
P52907	F-actin-capping protein subunit alpha-1	2	0.83	NA	NA
O60716	Catenin delta-1	5	0.82	0.115	1.7E-01
P53396	ATP-citrate synthase	2	0.82	NA	NA
Q8IZ83	Aldehyde dehydrogenase family 16 member A1	1	0.82	NA	NA
Q9Y4F1	FERM, RhoGEF and pleckstrin domain-containing protein 1	1	0.82	NA	NA
Q00765	Receptor expression-enhancing protein 5	5	0.82	0.119	1.7E-01
Q9P0M6	Core histone macro-H2A.2	3	0.82	0.156	3.3E-01
P28065	Proteasome subunit beta type-9	1	0.82	NA	NA
P82909	28S ribosomal protein S36, mitochondrial	1	0.82	NA	NA
P33176	Kinesin-1 heavy chain	3	0.81	0.102	1.8E-01
Q9Y5S2	Serine/threonine-protein kinase MRCK beta	1	0.81	NA	NA
P04632	Calpain small subunit 1	4	0.81	0.122	1.9E-01
O43707	Alpha-actinin-4	5	0.81	0.065	3.4E-02
P54920	Alpha-soluble NSF attachment protein	4	0.81	0.138	2.3E-01
Q9NR46	Endophilin-B2	1	0.81	NA	NA
P51116	Fragile X mental retardation syndrome-related protein 2	2	0.81	NA	NA
P55209	Nucleosome assembly protein 1-like 1	1	0.81	NA	NA
Q9H4M9	EH domain-containing protein 1	1	0.81	NA	NA
O15212	Prefoldin subunit 6	2	0.80	NA	NA
Q14344	Guanine nucleotide-binding protein subunit alpha-13	4	0.80	0.076	6.0E-02
P07384	Calpain-1 catalytic subunit	3	0.80	0.141	2.5E-01
Q9H223	EH domain-containing protein 4	2	0.80	NA	NA
P07305	Histone H1.0	5	0.80	0.193	3.0E-01
P14625	Endoplasmin	5	0.79	0.097	7.4E-02
P00918	Carbonic anhydrase 2	1	0.79	NA	NA
Q99816	Tumor susceptibility gene 101 protein	1	0.79	NA	NA
P0C0S5	Histone H2A.Z	5	0.79	0.063	2.1E-02
P13667	Protein disulfide-isomerase A4	5	0.79	0.132	1.5E-01
Q8NBQ5	Estradiol 17-beta-dehydrogenase 11	1	0.79	NA	NA
P26196	Probable ATP-dependent RNA helicase DDX6	4	0.79	0.067	3.8E-02
P62714	Serine/threonine-protein phosphatase 2A catalytic subunit beta isoform	1	0.79	NA	NA
Q07065	Cytoskeleton-associated protein 4	5	0.79	0.110	9.8E-02
O94832	Unconventional myosin-Id	5	0.79	0.224	3.5E-01
O95168	NADH dehydrogenase [ubiquinone] 1 beta subcomplex subunit 4	1	0.79	NA	NA
Q01518	Adenylyl cyclase-associated protein 1	1	0.79	NA	NA
Q15942	Zyxin	4	0.79	0.168	2.5E-01
Q8NFI4	Putative protein FAM10A5	1	0.79	NA	NA
Q9ULA0	Aspartyl aminopeptidase	2	0.79	NA	NA
P27361	Mitogen-activated protein kinase 3	1	0.78	NA	NA
P02647	Apolipoprotein A-I	5	0.78	0.267	4.1E-01
P04040	Catalase	5	0.78	0.159	1.9E-01
P17987	T-complex protein 1 subunit alpha	5	0.78	0.048	6.6E-03
Q93009	Ubiquitin carboxyl-terminal hydrolase 7	4	0.77	0.028	2.6E-03
P53621	Coatomer subunit alpha	5	0.77	0.069	1.9E-02
O00571	ATP-dependent RNA helicase DDX3X	1	0.77	NA	NA
P01009	Alpha-1-antitrypsin	5	0.77	0.142	1.4E-01
P55060	Exportin-2	3	0.77	0.120	1.6E-01
P31942	Heterogeneous nuclear ribonucleoprotein H3	3	0.76	0.209	3.3E-01
Q9UGP8	Translocation protein SEC63 homolog	1	0.76	NA	NA
Q07157	Tight junction protein ZO-1	1	0.76	NA	NA
P00367	Glutamate dehydrogenase 1, mitochondrial	5	0.76	0.061	1.1E-02
P36969	Phospholipid hydroperoxide glutathione peroxidase, mitochondrial	1	0.76	NA	NA
P35241	Radixin	1	0.76	NA	NA
Q9BX66	Sorbin and SH3 domain-containing protein 1	1	0.76	NA	NA
Q03252	Lamin-B2	5	0.76	0.082	2.7E-02
Q15149	Plectin	5	0.76	0.159	1.5E-01
P49419	Alpha-aminoadipic semialdehyde dehydrogenase	1	0.76	NA	NA
P02652	Apolipoprotein A-II	5	0.75	0.216	2.6E-01
Q14558	Phosphoribosyl pyrophosphate synthase-associated protein 1	2	0.75	NA	NA
P46937	Yorkie homolog	3	0.75	0.096	9.9E-02
Q9Y4G6	Talin-2	1	0.75	NA	NA
Q9Y305	Acyl-coenzyme A thioesterase 9, mitochondrial	1	0.75	NA	NA
Q13098	COP9 signalosome complex subunit 1	1	0.75	NA	NA
Q12797	Aspartyl/asparaginyl beta-hydroxylase	5	0.75	0.091	3.2E-02
O14735	CDP-diacylglycerol--inositol 3-phosphatidyltransferase	1	0.73	NA	NA
Q9Y3I0	tRNA-splicing ligase RtcB homolog	4	0.73	0.079	2.8E-02
Q99733	Nucleosome assembly protein 1-like 4	1	0.72	NA	NA
P46063	ATP-dependent DNA helicase Q1	1	0.72	NA	NA
Q04637	Eukaryotic translation initiation factor 4 gamma 1	2	0.72	NA	NA
P61163	Alpha-centractin	2	0.72	NA	NA
P43307	Translocon-associated protein subunit alpha	5	0.72	0.075	1.2E-02
Q71U36	Tubulin alpha-1A chain	2	0.72	NA	NA
Q16563	Synaptophysin-like protein 1	1	0.72	NA	NA
Q6NUK1	Calcium-binding mitochondrial carrier protein SCaMC-1	4	0.72	0.101	4.6E-02
P08603	Complement factor H	1	0.71	NA	NA
Q8TD19	Serine/threonine-protein kinase Nek9	1	0.71	NA	NA
P17655	Calpain-2 catalytic subunit	4	0.71	0.047	5.2E-03
O95299	NADH dehydrogenase [ubiquinone] 1 alpha subcomplex subunit 10, mitochondrial	1	0.71	NA	NA
P63208	S-phase kinase-associated protein 1	2	0.71	NA	NA
Q969P0	Immunoglobulin superfamily member 8	2	0.70	NA	NA
P60709	Actin, cytoplasmic 1	4	0.70	0.089	2.8E-02
Q9NZ45	CDGSH iron-sulfur domain-containing protein 1	1	0.70	NA	NA
O75131	Copine-3	3	0.70	0.178	1.8E-01
O94979	Protein transport protein Sec31A	4	0.70	0.087	2.6E-02
Q53GG5	PDZ and LIM domain protein 3	1	0.70	NA	NA
P05023	Sodium/potassium-transporting ATPase subunit alpha-1	5	0.69	0.068	5.9E-03
Q8WUM4	Programmed cell death 6-interacting protein	3	0.69	0.025	4.5E-03
Q9UHG3	Prenylcysteine oxidase 1	5	0.69	0.084	1.2E-02
P67936	Tropomyosin alpha-4 chain	5	0.69	0.156	7.5E-02
O60884	DnaJ homolog subfamily A member 2	1	0.69	NA	NA
P13861	cAMP-dependent protein kinase type II-alpha regulatory subunit	5	0.69	0.072	6.4E-03
P49458	Signal recognition particle 9 kDa protein	2	0.68	NA	NA
Q9H0D6	5'-3' exoribonuclease 2	1	0.68	NA	NA
Q8IX12	Cell division cycle and apoptosis regulator protein 1	2	0.68	NA	NA
P12235	ADP/ATP translocase 1	3	0.68	0.095	5.6E-02
P61769	Beta-2-microglobulin	4	0.68	0.250	2.2E-01
Q9H444	Charged multivesicular body protein 4b	2	0.68	NA	NA
O95865	N(G),N(G)-dimethylarginine dimethylaminohydrolase 2	4	0.68	0.123	5.0E-02
Q14203	Dynactin subunit 1	5	0.68	0.116	2.8E-02
Q16775	Hydroxyacylglutathione hydrolase, mitochondrial	1	0.68	NA	NA
P06899	Histone H2B type 1-J	5	0.68	0.143	5.3E-02
Q02218	2-oxoglutarate dehydrogenase, mitochondrial	5	0.67	0.097	1.5E-02
O00264	Membrane-associated progesterone receptor component 1	5	0.67	0.157	6.5E-02
Q05682	Caldesmon	5	0.67	0.135	4.2E-02
P63261	Actin, cytoplasmic 2	1	0.67	NA	NA
P17568	NADH dehydrogenase [ubiquinone] 1 beta subcomplex subunit 7	1	0.67	NA	NA
Q9HDC9	Adipocyte plasma membrane-associated protein	4	0.67	0.138	6.2E-02
O75844	CAAX prenyl protease 1 homolog	1	0.67	NA	NA
P08174	Complement decay-accelerating factor	2	0.66	NA	NA
P42025	Beta-centractin	2	0.66	NA	NA
Q92973	Transportin-1	3	0.66	0.047	1.2E-02
Q7L576	Cytoplasmic FMR1-interacting protein 1	2	0.65	NA	NA
P43121	Cell surface glycoprotein MUC18	5	0.65	0.195	9.1E-02
P16435	NADPH--cytochrome P450 reductase	1	0.65	NA	NA
Q9Y4L1	Hypoxia up-regulated protein 1	3	0.64	0.195	1.5E-01
P16615	Sarcoplasmic/endoplasmic reticulum calcium ATPase 2	5	0.64	0.110	1.6E-02
Q14204	Cytoplasmic dynein 1 heavy chain 1	5	0.64	0.067	2.8E-03
Q14956	Transmembrane glycoprotein NMB	5	0.64	0.409	3.4E-01
P35222	Catenin beta-1	5	0.64	0.113	1.7E-02
P07437	Tubulin beta chain	2	0.64	NA	NA
P06703	Protein S100-A6	2	0.63	NA	NA
Q9Y6N5	Sulfide:quinone oxidoreductase, mitochondrial	5	0.62	0.100	9.1E-03
Q9Y240	C-type lectin domain family 11 member A	1	0.62	NA	NA
O75746	Calcium-binding mitochondrial carrier protein Aralar1	4	0.62	0.114	2.4E-02
P05091	Aldehyde dehydrogenase, mitochondrial	4	0.61	0.098	1.5E-02

Table S17-Average Non-Metastatic Tumors

P00387	NADH-cytochrome b5 reductase 3	5	0.61	0.070	2.2E-03
P50995	Annexin A11	5	0.61	0.104	9.3E-03
P62873	Guanine nucleotide-binding protein G(I)/G(S)/G(T) subunit beta-1	2	0.61	NA	NA
Q92599	Septin-8	1	0.60	NA	NA
P02511	Alpha-crystallin B chain	5	0.60	0.230	9.2E-02
P06396	Gelsolin	5	0.60	0.244	1.1E-01
Q2VIR3	Putative eukaryotic translation initiation factor 2 subunit 3-like protein	1	0.60	NA	NA
Q9NQC3	Reticulon-4	5	0.60	0.202	6.3E-02
P17643	5,6-dihydroxyindole-2-carboxylic acid oxidase	4	0.59	0.627	4.7E-01
P09497	Clathrin light chain B	4	0.59	0.082	7.6E-03
Q9BQE3	Tubulin alpha-1C chain	3	0.59	0.103	3.6E-02
Q10567	AP-1 complex subunit beta-1	2	0.59	NA	NA
P50570	Dynamin-2	1	0.59	NA	NA
O14950	Myosin regulatory light chain 12B	5	0.58	0.140	1.8E-02
P68371	Tubulin beta-4B chain	3	0.58	0.083	2.3E-02
Q08722	Leukocyte surface antigen CD47	3	0.58	0.009	2.9E-04
P21589	5'-nucleotidase	3	0.58	0.072	1.7E-02
P35580	Myosin-10	5	0.58	0.139	1.7E-02
P00738	Haptoglobin	4	0.58	0.129	2.4E-02
O43175	D-3-phosphoglycerate dehydrogenase	3	0.57	0.421	3.2E-01
P0C0L5	Complement C4-B	3	0.57	0.167	7.9E-02
O94919	Endonuclease domain-containing 1 protein	3	0.57	0.398	2.9E-01
Q00610	Clathrin heavy chain 1	5	0.57	0.053	4.5E-04
Q6DD88	Atlastin-3	5	0.56	0.161	2.4E-02
Q13884	Beta-1-syntrophin	1	0.56	NA	NA
P40763	Signal transducer and activator of transcription 3	2	0.56	NA	NA
O60645	Exocyst complex component 3	2	0.56	NA	NA
O75955	Flotillin-1	2	0.56	NA	NA
Q92556	Engulfment and cell motility protein 1	1	0.56	NA	NA
Q09666	Neuroblast differentiation-associated protein AHNAK	5	0.56	0.126	9.5E-03
P04217	Alpha-1B-glycoprotein	5	0.55	0.134	1.1E-02
Q63ZY3	KN motif and ankyrin repeat domain-containing protein 2	4	0.55	0.257	1.0E-01
P11532	Dystrophin	1	0.55	NA	NA
P01042	Kininogen-1	3	0.55	0.227	1.2E-01
Q7Z406	Myosin-14	2	0.55	NA	NA
Q15836	Vesicle-associated membrane protein 3	1	0.55	NA	NA
Q92734	Protein TFG	1	0.55	NA	NA
O00159	Unconventional myosin-Ic	5	0.55	0.042	1.4E-04
P20339	Ras-related protein Rab-5A	1	0.55	NA	NA
Q9NVD7	Alpha-parvin	2	0.55	NA	NA
O75923	Dysferlin	1	0.54	NA	NA
P47755	F-actin-capping protein subunit alpha-2	1	0.54	NA	NA
P51659	Peroxisomal multifunctional enzyme type 2	1	0.54	NA	NA
P17612	cAMP-dependent protein kinase catalytic subunit alpha	5	0.54	0.110	5.2E-03
P23634	Plasma membrane calcium-transporting ATPase 4	5	0.54	0.132	9.6E-03
Q9HBL0	Tensin-1	5	0.54	0.184	2.9E-02
Q01484	Ankyrin-2	1	0.54	NA	NA
P48735	Isocitrate dehydrogenase [NADP], mitochondrial	5	0.53	0.121	6.6E-03
Q9BSD7	Cancer-related nucleoside-triphosphatase	1	0.53	NA	NA
Q9GZM7	Tubulointerstitial nephritis antigen-like	1	0.53	NA	NA
Q14254	Flotillin-2	4	0.53	0.164	3.0E-02
Q15019	Septin-2	5	0.53	0.038	7.4E-05
Q9BTV4	Transmembrane protein 43	4	0.53	0.170	3.3E-02
P05362	Intercellular adhesion molecule 1	5	0.52	0.238	5.2E-02
Q03591	Complement factor H-related protein 1	2	0.52	NA	NA
O94905	Erlin-2	5	0.51	0.097	2.4E-03
O43301	Heat shock 70 kDa protein 12A	4	0.51	0.114	1.0E-02
O43865	Putative adenosylhomocysteinase 2	2	0.51	NA	NA
P06727	Apolipoprotein A-IV	5	0.51	0.081	1.1E-03
P05556	Integrin beta-1	5	0.51	0.183	2.1E-02
P01893	Putative HLA class I histocompatibility antigen, alpha chain H	1	0.51	NA	NA
Q9BSJ8	Extended synaptotagmin-1	5	0.51	0.217	3.5E-02
O95197	Reticulon-3	1	0.50	NA	NA
P01903	HLA class II histocompatibility antigen, DR alpha chain	5	0.50	0.318	9.7E-02
Q13418	Integrin-linked protein kinase	1	0.50	NA	NA
P46939	Utrophin	4	0.50	0.288	9.6E-02
Q9Y490	Talin-1	5	0.50	0.076	7.9E-04
P36405	ADP-ribosylation factor-like protein 3	1	0.50	NA	NA
Q92522	Histone H1x	3	0.50	0.336	1.7E-01
P08133	Annexin A6	5	0.50	0.129	5.6E-03
P21964	Catechol O-methyltransferase	1	0.49	NA	NA
Q6NZI2	Polymerase I and transcript release factor	5	0.49	0.206	2.6E-02
O95782	AP-2 complex subunit alpha-1	2	0.49	NA	NA
P12814	Alpha-actinin-1	5	0.49	0.047	1.1E-04
Q14764	Major vault protein	5	0.48	0.145	7.6E-03
P32119	Peroxiredoxin-2	5	0.48	0.037	4.1E-05
P20073	Annexin A7	2	0.48	NA	NA
Q16181	Septin-7	5	0.48	0.048	1.0E-04
P10301	Ras-related protein R-Ras	1	0.48	NA	NA
O75369	Filamin-B	5	0.48	0.108	2.3E-03
P61764	Syntaxin-binding protein 1	3	0.47	0.247	9.2E-02
P50993	Sodium/potassium-transporting ATPase subunit alpha-2	1	0.47	NA	NA
P11413	Glucose-6-phosphate 1-dehydrogenase	3	0.47	0.192	5.9E-02
Q14192	Four and a half LIM domains protein 2	1	0.47	NA	NA
P05090	Apolipoprotein D	2	0.47	NA	NA
P32004	Neural cell adhesion molecule L1	1	0.46	NA	NA
P60201	Myelin proteolipid protein	1	0.45	NA	NA
Q29974	HLA class II histocompatibility antigen, DRB1-16 beta chain	1	0.43	NA	NA
P54289	Voltage-dependent calcium channel subunit alpha-2/delta-1	1	0.43	NA	NA
P0DJI8	Serum amyloid A-1 protein	1	0.42	NA	NA
P60033	CD81 antigen	1	0.42	NA	NA
Q969G5	Protein kinase C delta-binding protein	3	0.42	0.239	6.8E-02
Q5JRA6	Melanoma inhibitory activity protein 3	1	0.42	NA	NA
P35556	Fibrillin-2	1	0.41	NA	NA
Q86VB7	Scavenger receptor cysteine-rich type 1 protein M130	2	0.41	NA	NA
P00739	Haptoglobin-related protein	1	0.41	NA	NA
Q13557	Calcium/calmodulin-dependent protein kinase type II subunit delta	1	0.41	NA	NA
P08571	Monocyte differentiation antigen CD14	3	0.40	0.534	2.3E-01
Q93052	Lipoma-preferred partner	2	0.40	NA	NA
Q7Z7G0	Target of Nesh-SH3	1	0.40	NA	NA
O43760	Synaptogyrin-2	1	0.39	NA	NA
Q96CW1	AP-2 complex subunit mu	1	0.39	NA	NA
P01911	HLA class II histocompatibility antigen, DRB1-15 beta chain	1	0.37	NA	NA
P01892	HLA class I histocompatibility antigen, A-2 alpha chain	1	0.37	NA	NA
P63010	AP-2 complex subunit beta	2	0.37	NA	NA
P55083	Microfibril-associated glycoprotein 4	1	0.36	NA	NA
P05026	Sodium/potassium-transporting ATPase subunit beta-1	2	0.36	NA	NA
P28289	Tropomodulin-1	1	0.35	NA	NA
Q13425	Beta-2-syntrophin	3	0.35	0.357	9.9E-02
Q9NZN3	EH domain-containing protein 3	1	0.35	NA	NA
P09619	Platelet-derived growth factor receptor beta	1	0.33	NA	NA
P08311	Cathepsin G	1	0.30	NA	NA
Q9BUF5	Tubulin beta-6 chain	2	0.30	NA	NA
Q6UXB8	Peptidase inhibitor 16	2	0.29	NA	NA
P23229	Integrin alpha-6	2	0.29	NA	NA
P05787	Keratin, type II cytoskeletal 8	1	0.28	NA	NA
P17661	Desmin	1	0.28	NA	NA
P41222	Prostaglandin-H2 D-isomerase	1	0.28	NA	NA
Q10588	ADP-ribosyl cyclase/cyclic ADP-ribose hydrolase 2	1	0.28	NA	NA
P68366	Tubulin alpha-4A chain	1	0.28	NA	NA
P35611	Alpha-adducin	2	0.28	NA	NA
Q14141	Septin-6	1	0.27	NA	NA
Q96HC4	PDZ and LIM domain protein 5	1	0.26	NA	NA
Q9UKS6	Protein kinase C and casein kinase substrate in neurons protein 3	1	0.26	NA	NA
Q13642	Four and a half LIM domains protein 1	1	0.26	NA	NA
P09936	Ubiquitin carboxyl-terminal hydrolase isozyme L1	5	0.25	0.677	1.1E-01
Q14767	Latent-transforming growth factor beta-binding protein 2	2	0.25	NA	NA
Q13509	Tubulin beta-3 chain	2	0.25	NA	NA
Q08431	Lactadherin	2	0.22	NA	NA
Q15041	ADP-ribosylation factor-like protein 6-interacting protein 1	1	0.22	NA	NA
P16452	Erythrocyte membrane protein band 4.2	1	0.22	NA	NA
Q16270	Insulin-like growth factor-binding protein 7	1	0.22	NA	NA
Q96CX2	BTB/POZ domain-containing protein KCTD12	2	0.21	NA	NA
Q92777	Synapsin-2	2	0.20	NA	NA
P07996	Thrombospondin-1	1	0.20	NA	NA
Q9NY15	Stabilin-1	2	0.20	NA	NA
P15144	Aminopeptidase N	1	0.20	NA	NA
Q6NUI6	Chondroadherin-like protein	1	0.19	NA	NA
Q63HR2	Tensin-like C1 domain-containing phosphatase	2	0.19	NA	NA
Q9UBX5	Fibulin-5	1	0.19	NA	NA
P02462	Collagen alpha-1(IV) chain	2	0.19	NA	NA
P62736	Actin, aortic smooth muscle	2	0.18	NA	NA
P12277	Creatine kinase B-type	1	0.17	NA	NA
P59768	Guanine nucleotide-binding protein G(I)/G(S)/G(O) subunit gamma-2	1	0.17	NA	NA
P03973	Antileukoproteinase	1	0.16	NA	NA
Q16518	Retinoid isomerohydrolase	1	0.14	NA	NA
P63211	Guanine nucleotide-binding protein G(T) subunit gamma-T1	2	0.12	NA	NA
P35243	Recoverin	1	0.11	NA	NA
Q13885	Tubulin beta-2A chain	1	0.11	NA	NA
P02452	Collagen alpha-1(I) chain	2	0.10	NA	NA
P07196	Neurofilament light polypeptide	2	0.09	NA	NA
Q16853	Membrane primary amine oxidase	1	0.07	NA	NA
P07357	Complement component C8 alpha chain	1	0.05	NA	NA
P01024	Complement C3	5	0.47	0.187	1.5E-02
P06756	Integrin alpha-V	4	0.46	0.111	6.0E-03
P29992	Guanine nucleotide-binding protein subunit alpha-11	3	0.46	0.153	3.7E-02
Q9NZN4	EH domain-containing protein 2	4	0.44	0.122	6.7E-03
P04899	Guanine nucleotide-binding protein G(i) subunit alpha-2	4	0.44	0.133	8.6E-03
P07099	Epoxide hydrolase 1	5	0.44	0.181	1.0E-02
P18206	Vinculin	5	0.43	0.082	4.8E-04
Q15582	Transforming growth factor-beta-induced protein ig-h3	4	0.42	0.106	3.7E-03
P35579	Myosin-9	5	0.41	0.091	6.3E-04
Q13813	Spectrin alpha chain, non-erythrocytic 1	5	0.41	0.149	3.8E-03
P60660	Myosin light polypeptide 6	5	0.40	0.072	2.2E-04
Q01082	Spectrin beta chain, non-erythrocytic 1	5	0.40	0.185	7.4E-03
Q14624	Inter-alpha-trypsin inhibitor heavy chain H4	3	0.38	0.197	3.9E-02
Q07954	Prolow-density lipoprotein receptor-related protein 1	5	0.36	0.202	7.3E-03
Q16363	Laminin subunit alpha-4	5	0.35	0.136	1.5E-03
P04216	Thy-1 membrane glycoprotein	3	0.34	0.090	7.0E-03
P00747	Plasminogen	5	0.34	0.211	6.8E-03
Q03135	Caveolin-1	3	0.34	0.233	4.3E-02
Q02952	A-kinase anchor protein 12	5	0.34	0.176	3.4E-03
P22105	Tenascin-X	3	0.33	0.050	2.1E-03
O94911	ATP-binding cassette sub-family A member 8	4	0.33	0.139	4.0E-03
Q9BS40	Latexin	4	0.33	0.078	7.2E-04
P61626	Lysozyme C	4	0.32	0.252	2.0E-02
P07355	Annexin A2	5	0.32	0.204	4.8E-03
Q9NZM1	Myoferlin	5	0.32	0.029	2.5E-06
P04196	Histidine-rich glycoprotein	4	0.31	0.172	6.7E-03
P21333	Filamin-A	5	0.31	0.178	2.7E-03
P00167	Cytochrome b5	5	0.31	0.122	6.4E-04
P07942	Laminin subunit beta-1	4	0.30	0.090	9.4E-04
P09493	Tropomyosin alpha-1 chain	5	0.30	0.114	4.8E-04
P07358	Complement component C8 beta chain	3	0.30	0.270	4.7E-02
Q16555	Dihydropyrimidinase-related protein 2	5	0.30	0.104	3.1E-04
P00450	Ceruloplasmin	5	0.30	0.171	2.1E-03
P12111	Collagen alpha-3(VI) chain	5	0.29	0.163	1.6E-03

P12109	Collagen alpha-1(VI) chain	5	0.29	0.156	1.3E-03
P22413	Ectonucleotide pyrophosphatase/phosphodiesterase family member 1	4	0.29	0.144	3.2E-03
P16157	Ankyrin-1	4	0.28	0.130	2.3E-03
O00468	Agrin	4	0.28	0.230	1.2E-02
P02679	Fibrinogen gamma chain	5	0.28	0.127	5.6E-04
P13671	Complement component C6	3	0.27	0.082	4.0E-03
P02675	Fibrinogen beta chain	5	0.27	0.095	1.6E-04
P55268	Laminin subunit beta-2	5	0.27	0.127	4.9E-04
P02751	Fibronectin	5	0.26	0.174	1.5E-03
P02549	Spectrin alpha chain, erythrocytic 1	5	0.26	0.079	6.9E-05
O15230	Laminin subunit alpha-5	5	0.26	0.168	1.3E-03
P01011	Alpha-1-antichymotrypsin	5	0.25	0.203	2.4E-03
Q9Y6C2	EMILIN-1	5	0.25	0.289	8.8E-03
P02649	Apolipoprotein E	5	0.25	0.095	1.3E-04
P36269	Gamma-glutamyltransferase 5	5	0.25	0.213	2.7E-03
P12110	Collagen alpha-2(VI) chain	5	0.24	0.198	2.0E-03
P08294	Extracellular superoxide dismutase [Cu-Zn]	5	0.24	0.204	2.3E-03
P02749	Beta-2-glycoprotein 1	4	0.24	0.125	1.5E-03
P50895	Basal cell adhesion molecule	5	0.24	0.100	1.4E-04
P46821	Microtubule-associated protein 1B	5	0.23	0.184	1.4E-03
P11166	Solute carrier family 2, facilitated glucose transporter member 1	5	0.23	0.181	1.3E-03
P27105	Erythrocyte band 7 integral membrane protein	5	0.23	0.098	1.2E-04
P07197	Neurofilament medium polypeptide	4	0.23	0.206	5.8E-03
P07360	Complement component C8 gamma chain	3	0.23	0.177	1.4E-02
P05164	Myeloperoxidase	3	0.23	0.103	4.9E-03
P98160	Basement membrane-specific heparan sulfate proteoglycan core protein	5	0.23	0.099	1.2E-04
P02671	Fibrinogen alpha chain	5	0.21	0.077	3.5E-05
P11277	Spectrin beta chain, erythrocytic	5	0.21	0.080	4.1E-05
Q14112	Nidogen-2	5	0.21	0.091	6.4E-05
P02654	Apolipoprotein C-I	4	0.21	0.178	3.0E-03
Q14699	Raftlin	4	0.20	0.113	7.8E-04
P60903	Protein S100-A10	5	0.20	0.133	2.8E-04
O43491	Band 4.1-like protein 2	5	0.20	0.218	1.9E-03
P11047	Laminin subunit gamma-1	5	0.20	0.161	5.8E-04
Q01995	Transgelin	5	0.20	0.130	2.4E-04
Q9BXN1	Asporin	4	0.20	0.039	3.1E-05
P08572	Collagen alpha-2(IV) chain	5	0.19	0.123	1.8E-04
P01008	Antithrombin-III	5	0.19	0.133	2.4E-04
P01871	Ig mu chain C region	5	0.19	0.285	4.4E-03
O94875	Sorbin and SH3 domain-containing protein 2	5	0.19	0.207	1.3E-03
P04083	Annexin A1	5	0.19	0.309	5.7E-03
P58166	Inhibin beta E chain	4	0.19	0.154	1.7E-03
P02686	Myelin basic protein	5	0.19	0.136	2.5E-04
P80723	Brain acid soluble protein 1	5	0.18	0.264	3.0E-03
P39060	Collagen alpha-1(XVIII) chain	5	0.18	0.096	5.8E-05
Q12805	EGF-containing fibulin-like extracellular matrix protein 1	4	0.18	0.081	2.2E-04
P23946	Chymase	5	0.18	0.247	2.2E-03
P02760	Protein AMBP	4	0.17	0.242	5.5E-03
P10745	Retinol-binding protein 3	4	0.17	0.070	1.4E-04
Q2UY09	Collagen alpha-1(XXVIII) chain	3	0.17	0.290	2.6E-02
P05186	Alkaline phosphatase, tissue-nonspecific isozyme	5	0.17	0.094	4.7E-05
P20774	Mimecan	5	0.17	0.131	1.7E-04
P26447	Protein S100-A4	5	0.17	0.224	1.3E-03
O14495	Lipid phosphate phosphohydrolase 3	3	0.17	0.319	3.0E-02
P39059	Collagen alpha-1(XV) chain	5	0.17	0.207	9.7E-04
P43320	Beta-crystallin B2	5	0.16	0.198	8.1E-04
P21926	CD9 antigen	5	0.16	0.241	1.6E-03
P35749	Myosin-11	5	0.16	0.071	1.3E-05
P14543	Nidogen-1	5	0.16	0.104	6.0E-05
P21810	Biglycan	5	0.15	0.239	1.5E-03
Q05707	Collagen alpha-1(XIV) chain	5	0.15	0.190	5.9E-04
Q15661	Tryptase alpha/beta-1	5	0.15	0.157	2.7E-04
P68032	Actin, alpha cardiac muscle 1	3	0.15	0.186	9.4E-03
P10643	Complement component C7	3	0.15	0.270	1.9E-02
Q14195	Dihydropyrimidinase-related protein 3	5	0.15	0.213	8.4E-04
P04275	von Willebrand factor	5	0.15	0.152	2.3E-04
P02730	Band 3 anion transport protein	5	0.14	0.134	1.3E-04
Q9BXM0	Periaxin	5	0.14	0.248	1.4E-03
P01031	Complement C5	5	0.14	0.181	3.9E-04
P41219	Peripherin	5	0.14	0.217	7.8E-04
P51888	Prolargin	5	0.13	0.180	3.4E-04
P02748	Complement component C9	5	0.13	0.236	9.3E-04
P24844	Myosin regulatory light polypeptide 9	5	0.13	0.094	2.5E-05
P07585	Decorin	5	0.12	0.184	3.4E-04
P10909	Clusterin	5	0.12	0.191	3.7E-04
P15088	Mast cell carboxypeptidase A	4	0.12	0.096	1.9E-04
P35555	Fibrillin-1	5	0.12	0.226	6.8E-04
P35625	Metalloproteinase inhibitor 3	5	0.11	0.140	1.0E-04
P04004	Vitronectin	5	0.11	0.181	2.6E-04
P22748	Carbonic anhydrase 4	5	0.11	0.222	5.8E-04
P21980	Protein-glutamine gamma-glutamyltransferase 2	5	0.10	0.207	4.0E-04
P51884	Lumican	5	0.10	0.201	3.4E-04
P22352	Glutathione peroxidase 3	5	0.10	0.271	1.0E-03
P02743	Serum amyloid P-component	5	0.09	0.292	1.3E-03
P25189	Myelin protein P0	5	0.09	0.238	5.2E-04
P08123	Collagen alpha-2(I) chain	5	0.06	0.326	9.2E-04

Average LC MS/MS iTRAQ results from non-metastasized tumor specimens UM 13, 20, 23, 25, 26. Brown denotes change ≥ 2 standard deviations (SD) from the mean, yellow denotes change ≥ 1 SD and green highlights p values ≤ 0.05. NA, not applicable, n<3 samples.
