# Supplementary material for: iTRAQ Quantitative Proteomic Comparison of Metastatic and Non-Metastatic Uveal Melanoma Tumors
Source: PLoS One. 2015 Aug 25;10(8):e0135543. doi: 10.1371/journal.pone.0135543 (PMC4549237; doi:10.1371/journal.pone.0135543)
Supplement: S18 Table — (PDF) [file pone.0135543.s018.pdf]

Supplementary Table S18

Overall Summary of Quantitative Proteomic Results

Sample	[a]Proteins Quantified	[b]Significantly Elevated Proteins	[c]Significantly Decreased Proteins	Supplementary Table Number
Metastatic Tumors in Training Set				
UM19	899	81	85	S1
UM21	835	49	61	S2
UM24	724	65	66	S3
UM28	949	70	87	S4
UM30	912	72	100	S5
[d]Average (n = 5)	1405	85	125	S16
Non-Metastatic Tumors in Training Set				
UM13	884	66	79	S6
UM20	803	53	85	S7
UM23	910	71	92	S8
UM25	869	77	92	S9
UM26	798	69	84	S10
[e]Average (n = 5)	1389	79	120	S17
Independent Tumor Sample Set				
UM02 No Metastasis	239	24	30	S11
UM09 Metastasis	250	30	31	S12
UM11 Metastasis	193	19	23	S13
UM12 Metastasis	193	21	19	S14
UM15 No Metastasis	966	74	96	S15

a. Total number of proteins quantified with ≥ 2 peptides/protein. b. Number of proteins with ratios ≥ 1 standard deviation above the mean and p values ≤ 0.05 (t-test). c. Number of proteins with ratios ≥ 1 standard deviation below the mean and p values ≤ 0.05. d. From samples UM 19, 21, 24, 28, 30. e. From samples UM 13, 20, 23, 25, 26.
