# Supplementary material for: iTRAQ Quantitative Proteomic Comparison of Metastatic and Non-Metastatic Uveal Melanoma Tumors
Source: PLoS One. 2015 Aug 25;10(8):e0135543. doi: 10.1371/journal.pone.0135543 (PMC4549237; doi:10.1371/journal.pone.0135543)
Supplement: S19 Table — (PDF) [file pone.0135543.s019.pdf]

Supplementary Table S19

Relative Abundance: Proteins Detected Only in Metastatic UM Tumors

Uni-Prot Accession	Protein	Sample Frequency n = 5 total	Ratio UM/Control	SEM	*p* value
P62191	26S protease regulatory subunit 4	2	1.340	NA	NA
Q99460	26S proteasome non-ATPase regulatory subunit 1	2	0.869	NA	NA
O00232	26S proteasome non-ATPase regulatory subunit 12	1	0.945	NA	NA
Q9UNM6	26S proteasome non-ATPase regulatory subunit 13	2	1.069	NA	NA
O00487	26S proteasome non-ATPase regulatory subunit 14	1	1.084	NA	NA
Q16401	26S proteasome non-ATPase regulatory subunit 5	1	1.062	NA	NA
Q9Y3D9	28S ribosomal protein S23, mitochondrial	1	1.319	NA	NA
P63220	40S ribosomal protein S21	1	1.462	NA	NA
Q71UM5	40S ribosomal protein S27-like	2	1.479	NA	NA
P52209	6-phosphogluconate dehydrogenase, decarboxylating	1	2.093	NA	NA
P84098	60S ribosomal protein L19	1	1.023	NA	NA
P62910	60S ribosomal protein L32	1	1.174	NA	NA
Q9BPX5	Actin-related protein 2/3 complex subunit 5-like protein	1	0.967	NA	NA
P62330	ADP-ribosylation factor 6	1	1.306	NA	NA
P43652	Afamin	1	4.012	NA	NA
Q6NUM9	All-trans-retinol 13,14-reductase	1	0.702	NA	NA
P02763	Alpha-1-acid glycoprotein 1	3	1.988	0.480	0.289
P08697	Alpha-2-antiplasmin	1	1.473	NA	NA
Q16352	Alpha-internexin	1	0.836	NA	NA
Q9P2R3	Ankyrin repeat and FYVE domain-containing protein 1	2	0.832	NA	NA
P12429	Annexin A3	1	2.252	NA	NA
Q03518	Antigen peptide transporter 1	1	3.164	NA	NA
P04114	Apolipoprotein B-100	3	0.648	0.534	0.502
P02656	Apolipoprotein C-III	1	0.191	NA	NA
Q9BQE5	Apolipoprotein L2	1	7.896	NA	NA
O43776	Asparagine--tRNA ligase, cytoplasmic	2	2.170	NA	NA
Q15121	Astrocytic phosphoprotein PEA-15	2	1.003	NA	NA
P28288	ATP-binding cassette sub-family D member 3	1	0.572	NA	NA
Q01813	ATP-dependent 6-phosphofructokinase, platelet type	2	0.652	NA	NA
Q9GZR7	ATP-dependent RNA helicase DDX24	1	0.754	NA	NA
P17213	Bactericidal permeability-increasing protein	1	3.625	NA	NA
P08236	Beta-glucuronidase	2	2.459	NA	NA
Q3LXA3	Bifunctional ATP-dependent dihydroxyacetone kinase/FAD-AMP lyase (cyclizing)	1	2.466	NA	NA
P07814	Bifunctional glutamate/proline--tRNA ligase	2	1.553	NA	NA
O60271	C-Jun-amino-terminal kinase-interacting protein 4	1	0.751	NA	NA
P30622	CAP-Gly domain-containing linker protein 1	1	0.830	NA	NA
Q8NEV1	Casein kinase II subunit alpha 3	1	0.468	NA	NA
P11717	Cation-independent mannose-6-phosphate receptor	1	1.205	NA	NA
P14209	CD99 antigen	1	0.633	NA	NA
Q02224	Centromere-associated protein E	1	0.182	NA	NA
Q9BT22	Chitobiosyldiphosphodolichol beta-mannosyltransferase	1	0.406	NA	NA
Q8IWA5	Choline transporter-like protein 2	1	0.351	NA	NA
P45973	Chromobox protein homolog 5	1	1.979	NA	NA
P00740	Coagulation factor IX	1	0.703	NA	NA
P00488	Coagulation factor XIII A chain	1	0.811	NA	NA
P48444	Coatomer subunit delta	2	0.908	NA	NA
Q14011	Cold-inducible RNA-binding protein	1	0.787	NA	NA
P02461	Collagen alpha-1(III) chain	1	0.839	NA	NA
P02746	Complement C1q subcomponent subunit B	2	1.420	NA	NA
Q92905	COP9 signalosome complex subunit 5	2	0.606	NA	NA
P17540	Creatine kinase S-type, mitochondrial	2	1.542	NA	NA
Q13617	Cullin-2	1	1.367	NA	NA
P01034	Cystatin-C	1	1.591	NA	NA
P52943	Cysteine-rich protein 2	1	0.997	NA	NA
P04839	Cytochrome b-245 heavy chain	1	0.888	NA	NA
O43169	Cytochrome b5 type B	1	1.185	NA	NA
P14406	Cytochrome c oxidase subunit 7A2, mitochondrial	1	1.374	NA	NA
P21399	Cytoplasmic aconitate hydratase	1	2.031	NA	NA
Q6IAN0	Dehydrogenase/reductase SDR family member 7B	1	2.196	NA	NA
O00115	Deoxyribonuclease-2-alpha	1	2.790	NA	NA
Q8WVC6	Dephospho-CoA kinase domain-containing protein	2	1.015	NA	NA
Q9NY33	Dipeptidyl peptidase 3	1	2.343	NA	NA
Q02880	DNA topoisomerase 2-beta	1	0.549	NA	NA
Q9UBS4	DnaJ homolog subfamily B member 11	3	0.877	0.097	0.308
Q9NVH1	DnaJ homolog subfamily C member 11	1	1.862	NA	NA
Q9H3Z4	DnaJ homolog subfamily C member 5	1	0.811	NA	NA
Q05193	Dynamin-1	1	0.776	NA	NA
Q9NP97	Dynein light chain roadblock-type 1	1	0.704	NA	NA
Q14118	Dystroglycan	1	0.375	NA	NA
Q63HN8	E3 ubiquitin-protein ligase RNF213	1	2.346	NA	NA
O94874	E3 UFM1-protein ligase 1	2	1.223	NA	NA
Q9UI08	Ena/VASP-like protein	1	5.929	NA	NA
P42566	Epidermal growth factor receptor substrate 15	1	0.662	NA	NA
O75477	Erlin-1	1	0.653	NA	NA
P47813	Eukaryotic translation initiation factor 1A, X-chromosomal	1	0.871	NA	NA
P41091	Eukaryotic translation initiation factor 2 subunit 3	1	1.310	NA	NA
O75822	Eukaryotic translation initiation factor 3 subunit J	1	1.658	NA	NA
P23588	Eukaryotic translation initiation factor 4B	1	2.485	NA	NA
Q9NV70	Exocyst complex component 1	1	0.802	NA	NA
Q16610	Extracellular matrix protein 1	2	1.442	NA	NA
P51648	Fatty aldehyde dehydrogenase	1	0.767	NA	NA
P02792	Ferritin light chain	1	2.429	NA	NA
P23142	Fibulin-1	1	0.399	NA	NA
Q5T1M5	FK506-binding protein 15	1	1.438	NA	NA
P14207	Folate receptor beta	1	0.294	NA	NA
Q06787	Fragile X mental retardation protein 1	3	1.599	0.164	0.104
Q9UEY8	Gamma-adducin	1	0.414	NA	NA
O95479	GDH/6PGL endoplasmic bifunctional protein	1	0.941	NA	NA
P07093	Glia-derived nexin	3	2.304	0.211	0.059
P14136	Glial fibrillary acidic protein	2	0.184	NA	NA
O76003	Glutaredoxin-3	1	1.093	NA	NA
P48637	Glutathione synthetase	1	2.213	NA	NA
Q08379	Golgin subfamily A member 2	1	1.347	NA	NA
Q08378	Golgin subfamily A member 3	1	1.008	NA	NA
Q96S52	GPI transamidase component PIG-S	1	0.639	NA	NA
P49863	Granzyme K	1	4.362	NA	NA
P50151	Guanine nucleotide-binding protein G(I)/G(S)/G(O) subunit gamma-10	1	0.799	NA	NA
A8MTJ3	Guanine nucleotide-binding protein G(t) subunit alpha-3	1	0.259	NA	NA
Q9NX24	H/ACA ribonucleoprotein complex subunit 2	2	1.146	NA	NA
P14317	Hematopoietic lineage cell-specific protein	2	1.450	NA	NA
P09601	Heme oxygenase 1	1	3.785	NA	NA
Q9NRV9	Heme-binding protein 1	2	3.617	NA	NA
Q9Y3E1	Hepatoma-derived growth factor-related protein 3	1	1.071	NA	NA
P55795	Heterogeneous nuclear ribonucleoprotein H2	1	1.028	NA	NA
P30273	High affinity immunoglobulin epsilon receptor subunit gamma	1	4.176	NA	NA
P26583	High mobility group protein B2	5	0.552	0.170	0.025
O15347	High mobility group protein B3	1	2.896	NA	NA
Q13547	Histone deacetylase 1	1	1.623	NA	NA
P16403	Histone H1.2	2	0.948	NA	NA
P05534	HLA class I histocompatibility antigen, A-24 alpha chain	1	3.170	NA	NA
P04439	HLA class I histocompatibility antigen, A-3 alpha chain	1	3.182	NA	NA
P16188	HLA class I histocompatibility antigen, A-30 alpha chain	1	2.218	NA	NA
P30464	HLA class I histocompatibility antigen, B-15 alpha chain	1	1.571	NA	NA
P18463	HLA class I histocompatibility antigen, B-37 alpha chain	1	4.777	NA	NA
P30484	HLA class I histocompatibility antigen, B-46 alpha chain	1	1.542	NA	NA
Q07000	HLA class I histocompatibility antigen, Cw-15 alpha chain	1	1.227	NA	NA
P30504	HLA class I histocompatibility antigen, Cw-4 alpha chain	1	1.825	NA	NA
P10321	HLA class I histocompatibility antigen, Cw-7 alpha chain	2	0.705	NA	NA
P13760	HLA class II histocompatibility antigen, DRB1-4 beta chain	1	1.925	NA	NA
P01600	Ig kappa chain V-I region Hau	1	0.316	NA	NA
Q8TEX9	Importin-4	1	0.711	NA	NA
P11215	Integrin alpha-M	1	2.098	NA	NA
P19827	Inter-alpha-trypsin inhibitor heavy chain H1	1	1.396	NA	NA
P19823	Inter-alpha-trypsin inhibitor heavy chain H2	2	1.248	NA	NA
P32455	Interferon-induced guanylate-binding protein 1	1	4.785	NA	NA
O75569	Interferon-inducible double-stranded RNA-dependent protein kinase activator A	1	1.197	NA	NA
O43837	Isocitrate dehydrogenase [NAD] subunit beta, mitochondrial	2	1.007	NA	NA
P26440	Isovaleryl-CoA dehydrogenase, mitochondrial	1	1.702	NA	NA
P02788	Lactotransferrin	1	1.119	NA	NA
Q99538	Legumain	1	1.795	NA	NA

Q9Y608	Leucine-rich repeat flightless-interacting protein 2	1	1.252	NA	NA
P09960	Leukotriene A-4 hydrolase	1	2.834	NA	NA
P36776	Lon protease homolog, mitochondrial	1	1.276	NA	NA
Q15046	Lysine--tRNA ligase	3	1.357	0.239	0.330
O75352	Mannose-P-dolichol utilization defect 1 protein	1	1.230	NA	NA
P10721	Mast/stem cell growth factor receptor Kit	1	2.221	NA	NA
Q16655	Melanoma antigen recognized by T-cells 1	1	1.375	NA	NA
P55145	Mesencephalic astrocyte-derived neurotrophic factor	3	1.171	0.108	0.281
Q13361	Microfibrillar-associated protein 5	2	0.289	NA	NA
Q9UPN3	Microtubule-actin cross-linking factor 1, isoforms 1/2/3/5	4	0.873	0.132	0.379
P11137	Microtubule-associated protein 2	1	1.366	NA	NA
Q8N183	Mimitin, mitochondrial	1	1.883	NA	NA
Q8TCT9	Minor histocompatibility antigen H13	1	1.182	NA	NA
Q02978	Mitochondrial 2-oxoglutarate/malate carrier protein	2	1.091	NA	NA
Q9Y5J7	Mitochondrial import inner membrane translocase subunit Tim9	1	1.420	NA	NA
P28482	Mitogen-activated protein kinase 1	1	1.146	NA	NA
Q9Y3D0	Mitotic spindle-associated MMXD complex subunit MIP18	1	1.502	NA	NA
Q9NR56	Muscleblind-like protein 1	1	2.098	NA	NA
O95297	Myelin protein zero-like protein 1	1	0.650	NA	NA
P24158	Myeloblastin	1	3.403	NA	NA
P41218	Myeloid cell nuclear differentiation antigen	1	3.062	NA	NA
Q8NFW8	N-acylneuraminate cytidylyltransferase	1	0.627	NA	NA
P51688	N-sulphoglucosamine sulphohydrolase	1	2.376	NA	NA
O94760	N(G),N(G)-dimethylarginine dimethylaminohydrolase 1	1	0.427	NA	NA
O95182	NADH dehydrogenase [ubiquinone] 1 alpha subcomplex subunit 7	1	1.619	NA	NA
Q8WXH0	Nesprin-2	2	0.765	NA	NA
O14786	Neuropilin-1	2	0.307	NA	NA
Q9Y639	Neuroplastin	2	0.685	NA	NA
Q6PIU2	Neutral cholesterol ester hydrolase 1	1	2.255	NA	NA
P59665	Neutrophil defensin 1	1	4.027	NA	NA
P08246	Neutrophil elastase	1	2.417	NA	NA
O15118	Niemann-Pick C1 protein	2	1.413	NA	NA
Q5JPE7	Nodal modulator 2	1	1.228	NA	NA
Q9Y266	Nuclear migration protein nudC	1	1.206	NA	NA
Q9H1E3	Nuclear ubiquitous casein and cyclin-dependent kinase substrate 1	2	3.585	NA	NA
P67809	Nuclease-sensitive element-binding protein 1	1	2.848	NA	NA
P80303	Nucleobindin-2	1	1.072	NA	NA
Q14978	Nucleolar and coiled-body phosphoprotein 1	1	1.991	NA	NA
Q01085	Nucleolysin TIAR	1	0.992	NA	NA
Q6UWY5	Olfactomedin-like protein 1	2	0.144	NA	NA
Q9NRN5	Olfactomedin-like protein 3	1	0.129	NA	NA
Q9UL41	Paraneoplastic antigen Ma3	1	6.849	NA	NA
Q8NB37	Parkinson disease 7 domain-containing protein 1	1	1.071	NA	NA
O00151	PDZ and LIM domain protein 1	1	1.206	NA	NA
Q9NR12	PDZ and LIM domain protein 7	1	0.374	NA	NA
Q96AY3	Peptidyl-prolyl cis-trans isomerase FKBP10	1	1.232	NA	NA
Q00688	Peptidyl-prolyl cis-trans isomerase FKBP3	2	1.706	NA	NA
P48426	Phosphatidylinositol 5-phosphate 4-kinase type-2 alpha	3	1.700	0.178	0.096
P36871	Phosphoglucomutase-1	2	2.026	NA	NA
Q96G03	Phosphoglucomutase-2	1	3.656	NA	NA
P14555	Phospholipase A2, membrane associated	2	0.212	NA	NA
Q9HBL7	Plasminogen receptor (KT)	1	0.760	NA	NA
P02776	Platelet factor 4	1	0.584	NA	NA
P21246	Pleiotrophin	2	0.105	NA	NA
Q9UMS4	Pre-mRNA-processing factor 19	1	1.102	NA	NA
P61758	Prefoldin subunit 3	1	0.921	NA	NA
Q9NQP4	Prefoldin subunit 4	1	1.304	NA	NA
P09668	Pro-cathepsin H	1	1.511	NA	NA
Q8NDH3	Probable aminopeptidase NPEPL1	1	0.949	NA	NA
Q99848	Probable rRNA-processing protein EBP2	1	3.956	NA	NA
P12004	Proliferating cell nuclear antigen	1	1.771	NA	NA
Q8N5C1	Protein FAM26E	1	0.149	NA	NA
P35813	Protein phosphatase 1A	1	0.761	NA	NA
Q13123	Protein Red	1	1.399	NA	NA
P05109	Protein S100-A8	1	4.046	NA	NA
P06702	Protein S100-A9	1	5.754	NA	NA
P55735	Protein SEC13 homolog	1	0.765	NA	NA
Q08AM6	Protein VAC14 homolog	2	1.798	NA	NA
P00734	Prothrombin	2	1.963	NA	NA
Q9NQ39	Putative 40S ribosomal protein S10-like	1	2.025	NA	NA
Q96HN2	Putative adenosylhomocysteinase 3	1	0.360	NA	NA
Q6GMV3	Putative peptidyl-tRNA hydrolase PTRHD1	1	1.648	NA	NA
O43143	Putative pre-mRNA-splicing factor ATP-dependent RNA helicase DHX15	1	1.208	NA	NA
Q9Y383	Putative RNA-binding protein Luc7-like 2	3	1.413	0.210	0.242
Q9NVS9	Pyridoxine-5'-phosphate oxidase	1	0.988	NA	NA
Q15404	Ras suppressor protein 1	1	0.464	NA	NA
P15153	Ras-related C3 botulinum toxin substrate 2	2	1.338	NA	NA
Q9HB90	Ras-related GTP-binding protein C	1	1.401	NA	NA
Q13637	Ras-related protein Rab-32	1	3.642	NA	NA
P08575	Receptor-type tyrosine-protein phosphatase C	2	1.316	NA	NA
Q96DB5	Regulator of microtubule dynamics protein 1	1	2.046	NA	NA
Q96D15	Reticulocalbin-3	1	0.654	NA	NA
P60891	Ribose-phosphate pyrophosphokinase 1	1	0.872	NA	NA
Q96PK6	RNA-binding protein 14	1	1.143	NA	NA
Q15413	Ryanodine receptor 3	1	0.506	NA	NA
Q9NVA2	Septin-11	3	0.605	0.178	0.106
Q08170	Serine/arginine-rich splicing factor 4	1	3.243	NA	NA
O75914	Serine/threonine-protein kinase PAK 3	1	0.789	NA	NA
P62140	Serine/threonine-protein phosphatase PP1-beta catalytic subunit	1	1.380	NA	NA
P27169	Serum paraoxonase/arylesterase 1	1	7.234	NA	NA
Q15165	Serum paraoxonase/arylesterase 2	1	0.671	NA	NA
Q9BZZ2	Sialoadhesin	1	0.607	NA	NA
Q15005	Signal peptidase complex subunit 2	1	1.012	NA	NA
Q13573	SNW domain-containing protein 1	1	2.341	NA	NA
P30626	Sorcin	1	0.900	NA	NA
O60749	Sorting nexin-2	2	0.934	NA	NA
Q9H2G2	STE20-like serine/threonine-protein kinase	1	1.337	NA	NA
P50225	Sulfotransferase 1A1	1	1.114	NA	NA
O15260	Surfeit locus protein 4	1	0.902	NA	NA
Q9HCJ6	Synaptic vesicle membrane protein VAT-1 homolog-like	1	0.096	NA	NA
P24821	Tenascin	1	1.785	NA	NA
Q99614	Tetratricopeptide repeat protein 1	1	1.439	NA	NA
P26639	Threonine--tRNA ligase, cytoplasmic	1	1.286	NA	NA
P23919	Thymidylate kinase	1	0.917	NA	NA
Q9P016	Thymocyte nuclear protein 1	1	1.225	NA	NA
O15455	Toll-like receptor 3	1	0.858	NA	NA
Q6UW68	Transmembrane protein 205	1	2.288	NA	NA
Q9NYL9	Tropomodulin-3	1	0.574	NA	NA
P68363	Tubulin alpha-1B chain	1	0.565	NA	NA
Q9BVJ6	U3 small nucleolar RNA-associated protein 14 homolog A	1	2.937	NA	NA
Q92890	Ubiquitin fusion degradation protein 1 homolog	2	1.431	NA	NA
Q14376	UDP-glucose 4-epimerase	1	2.481	NA	NA
Q9NYU1	UDP-glucose:glycoprotein glucosyltransferase 2	1	1.907	NA	NA
O00160	Unconventional myosin-If	2	1.721	NA	NA
Q13488	V-type proton ATPase 116 kDa subunit a isoform 3	2	1.323	NA	NA
Q15904	V-type proton ATPase subunit S1	1	1.475	NA	NA
O75351	Vacuolar protein sorting-associated protein 4B	1	1.805	NA	NA
Q9NZ01	Very-long-chain enoyl-CoA reductase	1	0.554	NA	NA
Q12907	Vesicular integral-membrane protein VIP36	1	1.011	NA	NA
O60504	Vinexin	1	0.631	NA	NA
Q96D96	Voltage-gated hydrogen channel 1	1	1.564	NA	NA
Q13303	Voltage-gated potassium channel subunit beta-2	1	2.315	NA	NA
Q5SNT6	WASH complex subunit FAM21B	1	4.948	NA	NA
Q9GZS3	WD repeat-containing protein 61	1	1.249	NA	NA
Q562E7	WD repeat-containing protein 81	1	1.315	NA	NA

Proteins (n = 255) detected only in metastasized UM tumors among 1646 total proteins quantified with 2 or more peptides in metastatic samples UM 19, 21, 24, 28, 30 and non-metastatastic samples UM 13, 20, 23, 25, 26. NA, not applicable, n<3 samples.
