# Supplementary material for: iTRAQ Quantitative Proteomic Comparison of Metastatic and Non-Metastatic Uveal Melanoma Tumors
Source: PLoS One. 2015 Aug 25;10(8):e0135543. doi: 10.1371/journal.pone.0135543 (PMC4549237; doi:10.1371/journal.pone.0135543)
Supplement: S20 Table — (PDF) [file pone.0135543.s020.pdf]

Supplementary Table S20

Relative Abundance: Proteins Detected Only in Non-Metastatic UM Tumors

Uni-Prot Accession	Protein	Sample Frequency n = 5 total	Ratio UM/Control	SEM	*p* value
Q04446	1,4-alpha-glucan-branching enzyme	2	2.004	NA	NA
Q9NRX4	14 kDa phosphohistidine phosphatase	1	2.133	NA	NA
P62333	26S protease regulatory subunit 10B	1	0.968	NA	NA
O75832	26S proteasome non-ATPase regulatory subunit 10	1	0.860	NA	NA
P51398	28S ribosomal protein S29, mitochondrial	1	1.010	NA	NA
P31937	3-hydroxyisobutyrate dehydrogenase, mitochondrial	2	1.854	NA	NA
Q06136	3-ketodihydrosphingosine reductase	1	1.361	NA	NA
Q13405	39S ribosomal protein L49, mitochondrial	1	1.706	NA	NA
P62861	40S ribosomal protein S30	1	2.207	NA	NA
O14841	5-oxoprolinase	1	1.723	NA	NA
Q9H0D6	5'-3' exoribonuclease 2	1	0.683	NA	NA
Q86UY8	5'-nucleotidase domain-containing protein 3	1	1.379	NA	NA
P61313	60S ribosomal protein L15	2	1.299	NA	NA
P62888	60S ribosomal protein L30	1	0.929	NA	NA
P62899	60S ribosomal protein L31	3	1.246	0.139	0.255
Q6DKI1	60S ribosomal protein L7-like 1	1	1.461	NA	NA
Q9NPJ3	Acyl-coenzyme A thioesterase 13	1	2.105	NA	NA
P49753	Acyl-coenzyme A thioesterase 2, mitochondrial	1	1.625	NA	NA
Q9Y305	Acyl-coenzyme A thioesterase 9, mitochondrial	1	0.749	NA	NA
P13798	Acylamino-acid-releasing enzyme	3	2.110	0.062	0.007
Q53H12	Acylglycerol kinase, mitochondrial	1	1.112	NA	NA
Q6P587	Acylpyruvase FAHD1, mitochondrial	1	0.903	NA	NA
P00568	Adenylate kinase isoenzyme 1	1	2.218	NA	NA
Q10588	ADP-ribosyl cyclase/cyclic ADP-ribose hydrolase 2	1	0.280	NA	NA
P36404	ADP-ribosylation factor-like protein 2	1	1.283	NA	NA
P36405	ADP-ribosylation factor-like protein 3	1	0.498	NA	NA
Q96BM9	ADP-ribosylation factor-like protein 8A	2	1.186	NA	NA
P12236	ADP/ATP translocase 3	2	1.145	NA	NA
Q9Y6U3	Adseverin	1	4.470	NA	NA
Q8IZ83	Aldehyde dehydrogenase family 16 member A1	1	0.823	NA	NA
Q9BQI0	Allograft inflammatory factor 1-like	3	1.376	0.130	0.134
P37840	Alpha-synuclein	1	1.391	NA	NA
Q9H4A4	Aminopeptidase B	2	1.965	NA	NA
Q01484	Ankyrin-2	1	0.539	NA	NA
O14617	AP-3 complex subunit delta-1	1	1.163	NA	NA
Q6PI48	Aspartate--tRNA ligase, mitochondrial	2	1.980	NA	NA
Q8WWM7	Ataxin-2-like protein	1	1.753	NA	NA
P53396	ATP-citrate synthase	2	0.824	NA	NA
O00148	ATP-dependent RNA helicase DDX39A	1	1.703	NA	NA
O15523	ATP-dependent RNA helicase DDX3Y	1	0.853	NA	NA
O75531	Barrier-to-autointegration factor	2	2.264	NA	NA
Q13884	Beta-1-syntrophin	1	0.561	NA	NA
P51817	cAMP-dependent protein kinase catalytic subunit PRKX	1	1.527	NA	NA
P43235	Cathepsin K	1	3.533	NA	NA
P20645	Cation-dependent mannose-6-phosphate receptor	3	1.127	0.103	0.367
Q9Y5K6	CD2-associated protein	1	1.211	NA	NA
Q9NZ45	CDGSH iron-sulfur domain-containing protein 1	1	0.702	NA	NA
Q9Y696	Chloride intracellular channel protein 4	2	1.495	NA	NA
Q6NUI6	Chondroadherin-like protein	1	0.192	NA	NA
Q96A33	Coiled-coil domain-containing protein 47	1	1.074	NA	NA
P02458	Collagen alpha-1(II) chain	1	1.115	NA	NA
P08174	Complement decay-accelerating factor	2	0.663	NA	NA
O14618	Copper chaperone for superoxide dismutase	1	2.239	NA	NA
Q13619	Cullin-4A	1	1.761	NA	NA
O14949	Cytochrome b-c1 complex subunit 8	1	1.160	NA	NA
Q13409	Cytoplasmic dynein 1 intermediate chain 2	1	1.199	NA	NA
Q9Y6G9	Cytoplasmic dynein 1 light intermediate chain 1	2	0.927	NA	NA
P53634	Dipeptidyl peptidase 1	1	1.356	NA	NA
O60884	DnaJ homolog subfamily A member 2	1	0.687	NA	NA
P55265	Double-stranded RNA-specific adenosine deaminase	3	1.359	0.145	0.169
O75923	Dysferlin	1	0.545	NA	NA
P11532	Dystrophin	1	0.551	NA	NA
O95834	Echinoderm microtubule-associated protein-like 2	3	2.528	0.215	0.050
Q9NZN3	EH domain-containing protein 3	1	0.348	NA	NA
Q9BXX0	EMILIN-2	1	0.941	NA	NA
Q9Y371	Endophilin-B1	1	1.572	NA	NA
Q9NR46	Endophilin-B2	1	0.812	NA	NA
O75521	Enoyl-CoA delta isomerase 2, mitochondrial	3	0.990	0.129	0.947
Q9NPA0	ER membrane protein complex subunit 7	1	1.302	NA	NA
P16452	Erythrocyte membrane protein band 4.2	1	0.221	NA	NA
Q14240	Eukaryotic initiation factor 4A-II	3	1.929	0.058	0.008
Q13347	Eukaryotic translation initiation factor 3 subunit I	1	1.064	NA	NA
Q9UBQ5	Eukaryotic translation initiation factor 3 subunit K	1	1.819	NA	NA
Q15056	Eukaryotic translation initiation factor 4H	1	1.408	NA	NA
P55010	Eukaryotic translation initiation factor 5	1	2.484	NA	NA
O60645	Exocyst complex component 3	2	0.559	NA	NA
Q01469	Fatty acid-binding protein, epidermal	1	1.410	NA	NA
Q05397	Focal adhesion kinase 1	1	1.212	NA	NA
P51116	Fragile X mental retardation syndrome-related protein 2	2	0.811	NA	NA
Q9Y653	G-protein coupled receptor 56	1	1.813	NA	NA
O94925	Glutaminase kidney isoform, mitochondrial	1	0.838	NA	NA
Q06210	Glutamine--fructose-6-phosphate aminotransferase [isomerizing] 1	1	1.491	NA	NA
P47897	Glutamine--tRNA ligase	1	0.983	NA	NA
P46439	Glutathione S-transferase Mu 5	1	1.274	NA	NA
P30711	Glutathione S-transferase theta-1	1	1.375	NA	NA
P0CG29	Glutathione S-transferase theta-2	1	1.224	NA	NA
P46976	Glycogenin-1	1	0.920	NA	NA
P36959	GMP reductase 1	1	2.525	NA	NA
Q14789	Golgin subfamily B member 1	1	0.999	NA	NA
Q9HAV7	GrpE protein homolog 1, mitochondrial	1	1.460	NA	NA
Q9H0R4	Haloacid dehalogenase-like hydrolase domain-containing protein 2	1	2.624	NA	NA
P00739	Haptoglobin-related protein	1	0.407	NA	NA
P12081	Histidine--tRNA ligase, cytoplasmic	1	2.063	NA	NA
Q99879	Histone H2B type 1-M	1	1.356	NA	NA
Q16576	Histone-binding protein RBBP7	2	1.605	NA	NA
P01892	HLA class I histocompatibility antigen, A-2 alpha chain	1	0.371	NA	NA
P30512	HLA class I histocompatibility antigen, A-29 alpha chain	1	1.499	NA	NA
P30485	HLA class I histocompatibility antigen, B-47 alpha chain	1	1.889	NA	NA
P30508	HLA class I histocompatibility antigen, Cw-12 alpha chain	1	0.827	NA	NA
P20039	HLA class II histocompatibility antigen, DRB1-11 beta chain	1	3.072	NA	NA
Q29974	HLA class II histocompatibility antigen, DRB1-16 beta chain	1	0.430	NA	NA
Q16543	Hsp90 co-chaperone Cdc37	2	1.616	NA	NA
Q16775	Hydroxyacylglutathione hydrolase, mitochondrial	1	0.676	NA	NA
Q969P0	Immunoglobulin superfamily member 8	2	0.703	NA	NA
P20591	Interferon-induced GTP-binding protein Mx1	1	2.317	NA	NA
P09914	Interferon-induced protein with tetratricopeptide repeats 1	1	1.966	NA	NA
Q5VWZ2	Lysophospholipase-like protein 1	1	1.863	NA	NA
P13473	Lysosome-associated membrane glycoprotein 2	2	0.980	NA	NA
P56192	Methionine--tRNA ligase, cytoplasmic	2	1.309	NA	NA
Q9HCC0	Methylcrotonoyl-CoA carboxylase beta chain, mitochondrial	1	1.004	NA	NA
Q13825	Methylglutaconyl-CoA hydratase, mitochondrial	1	0.936	NA	NA
P55083	Microfibril-associated glycoprotein 4	1	0.365	NA	NA
O14880	Microsomal glutathione S-transferase 3	1	1.414	NA	NA
O60220	Mitochondrial import inner membrane translocase subunit Tim8 A	1	3.847	NA	NA
O96008	Mitochondrial import receptor subunit TOM40 homolog	1	0.828	NA	NA
P27361	Mitogen-activated protein kinase 3	1	0.784	NA	NA
P53985	Monocarboxylate transporter 1	1	1.967	NA	NA
Q9H7C9	Mth938 domain-containing protein	1	1.850	NA	NA
Q7Z406	Myosin-14	2	0.551	NA	NA
Q8NCW5	NAD(P)H-hydrate epimerase	1	3.819	NA	NA
O96000	NADH dehydrogenase [ubiquinone] 1 beta subcomplex subunit 10	2	1.442	NA	NA
P17568	NADH dehydrogenase [ubiquinone] 1 beta subcomplex subunit 7	1	0.671	NA	NA
P49821	NADH dehydrogenase [ubiquinone] flavoprotein 1, mitochondrial	2	1.261	NA	NA

O00217	NADH dehydrogenase [ubiquinone] iron-sulfur protein 8, mitochondrial	1	1.869	NA	NA
Q9UMX5	Neudesin	2	0.950	NA	NA
P32004	Neural cell adhesion molecule L1	1	0.462	NA	NA
P07196	Neurofilament light polypeptide	2	0.094	NA	NA
Q9BYT8	Neurolysin, mitochondrial	1	2.580	NA	NA
Q92542	Nicastrin	1	0.831	NA	NA
Q6XQN6	Nicotinate phosphoribosyltransferase	1	3.714	NA	NA
P05204	Non-histone chromosomal protein HMG-17	1	7.968	NA	NA
Q9BW27	Nuclear pore complex protein Nup85	1	0.984	NA	NA
P17480	Nucleolar transcription factor 1	1	1.523	NA	NA
P22392	Nucleoside diphosphate kinase B	1	1.963	NA	NA
Q9NTK5	Obg-like ATPase 1	2	1.135	NA	NA
Q9NX40	OCIA domain-containing protein 1	1	1.284	NA	NA
P04181	Ornithine aminotransferase, mitochondrial	1	1.865	NA	NA
Q9BZF1	Oxysterol-binding protein-related protein 8	1	1.613	NA	NA
P50897	Palmitoyl-protein thioesterase 1	1	1.248	NA	NA
Q53GG5	PDZ and LIM domain protein 3	1	0.698	NA	NA
Q02790	Peptidyl-prolyl cis-trans isomerase FKBP4	2	1.407	NA	NA
P51659	Peroxisomal multifunctional enzyme type 2	1	0.544	NA	NA
O95571	Persulfide dioxygenase ETHE1, mitochondrial	1	1.737	NA	NA
Q9Y285	Phenylalanine--tRNA ligase alpha subunit	1	1.914	NA	NA
P42356	Phosphatidylinositol 4-kinase alpha	1	0.992	NA	NA
P36969	Phospholipid hydroperoxide glutathione peroxidase, mitochondrial	1	0.759	NA	NA
Q9H307	Pinin	1	2.406	NA	NA
Q15102	Platelet-activating factor acetylhydrolase IB subunit gamma	3	2.147	0.281	0.113
O60486	Plexin-C1	3	1.113	0.043	0.129
O95758	Polypyrimidine tract-binding protein 3	1	1.587	NA	NA
O75400	Pre-mRNA-processing factor 40 homolog A	1	1.493	NA	NA
Q9UHV9	Prefoldin subunit 2	2	1.010	NA	NA
Q99471	Prefoldin subunit 5	1	1.412	NA	NA
P51531	Probable global transcription activator SNF2L2	1	1.049	NA	NA
P48147	Prolyl endopeptidase	1	1.851	NA	NA
Q15185	Prostaglandin E synthase 3	1	2.246	NA	NA
P25789	Proteasome subunit alpha type-4	1	2.697	NA	NA
P49720	Proteasome subunit beta type-3	1	1.790	NA	NA
P28072	Proteasome subunit beta type-6	1	2.054	NA	NA
Q99436	Proteasome subunit beta type-7	2	1.470	NA	NA
P28062	Proteasome subunit beta type-8	1	1.348	NA	NA
Q9UKY7	Protein CDV3 homolog	1	1.701	NA	NA
O60888	Protein CutA	1	3.458	NA	NA
Q96JJ7	Protein disulfide-isomerase TMX3	1	0.938	NA	NA
Q9UKS6	Protein kinase C and casein kinase substrate in neurons protein 3	1	0.263	NA	NA
P49593	Protein phosphatase 1F	2	0.867	NA	NA
Q8WUY3	Protein prune homolog 2	1	1.694	NA	NA
P23297	Protein S100-A1	1	2.730	NA	NA
Q92734	Protein TFG	1	0.549	NA	NA
P61457	Pterin-4-alpha-carbinolamine dehydratase	1	0.919	NA	NA
Q5JNZ5	Putative 40S ribosomal protein S26-like 1	2	2.441	NA	NA
Q2VIR3	Putative eukaryotic translation initiation factor 2 subunit 3-like protein	1	0.598	NA	NA
P01893	Putative HLA class I histocompatibility antigen, alpha chain H	1	0.506	NA	NA
Q8NHP8	Putative phospholipase B-like 2	1	1.607	NA	NA
Q8NFI4	Putative protein FAM10A5	1	0.786	NA	NA
Q08257	Quinone oxidoreductase	1	1.443	NA	NA
P35241	Radixin	1	0.759	NA	NA
P60763	Ras-related C3 botulinum toxin substrate 3	1	0.953	NA	NA
Q8WUD1	Ras-related protein Rab-2B	1	1.052	NA	NA
O95716	Ras-related protein Rab-3D	1	1.131	NA	NA
P20339	Ras-related protein Rab-5A	1	0.546	NA	NA
P20340	Ras-related protein Rab-6A	1	1.265	NA	NA
P61006	Ras-related protein Rab-8A	1	1.107	NA	NA
O95197	Reticulon-3	1	0.504	NA	NA
Q16518	Retinoid isomerohydrolase	1	0.144	NA	NA
O00584	Ribonuclease T2	1	3.119	NA	NA
P16083	Ribosyldihydronicotinamide dehydrogenase [quinone]	1	3.470	NA	NA
P10768	S-formylglutathione hydrolase	2	2.152	NA	NA
Q13126	S-methyl-5'-thioadenosine phosphorylase	3	2.197	0.117	0.021
P82979	SAP domain-containing ribonucleoprotein	1	2.726	NA	NA
Q9UL12	Sarcosine dehydrogenase, mitochondrial	1	2.183	NA	NA
P35270	Sepiapterin reductase	1	2.858	NA	NA
Q14141	Septin-6	1	0.272	NA	NA
Q05519	Serine/arginine-rich splicing factor 11	1	1.354	NA	NA
Q9Y5S2	Serine/threonine-protein kinase MRCK beta	1	0.815	NA	NA
Q8TD19	Serine/threonine-protein kinase Nek9	1	0.711	NA	NA
O95747	Serine/threonine-protein kinase OSR1	1	1.366	NA	NA
Q14738	Serine/threonine-protein phosphatase 2A 56 kDa regulatory subunit delta isoform	1	1.358	NA	NA
P36873	Serine/threonine-protein phosphatase PP1-gamma catalytic subunit	1	1.137	NA	NA
P35237	Serpin B6	1	1.729	NA	NA
Q9BXP5	Serrate RNA effector molecule homolog	1	1.093	NA	NA
P45954	Short/branched chain specific acyl-CoA dehydrogenase, mitochondrial	2	1.202	NA	NA
Q9Y5M8	Signal recognition particle receptor subunit beta	2	1.134	NA	NA
Q04837	Single-stranded DNA-binding protein, mitochondrial	1	2.050	NA	NA
P62316	Small nuclear ribonucleoprotein Sm D2	2	0.980	NA	NA
P14678	Small nuclear ribonucleoprotein-associated proteins B and B'	1	1.338	NA	NA
Q6EEV6	Small ubiquitin-related modifier 4	1	5.688	NA	NA
P50993	Sodium/potassium-transporting ATPase subunit alpha-2	1	0.470	NA	NA
Q9BX66	Sorbin and SH3 domain-containing protein 1	1	0.757	NA	NA
Q9UMY4	Sorting nexin-12	2	1.643	NA	NA
O60493	Sorting nexin-3	2	1.122	NA	NA
Q8N0X7	Spartin	1	1.143	NA	NA
P16949	Stathmin	2	2.627	NA	NA
Q02318	Sterol 26-hydroxylase, mitochondrial	3	1.440	0.145	0.129
O15498	Synaptobrevin homolog YKT6	1	1.542	NA	NA
Q13277	Syntaxin-3	1	1.380	NA	NA
P61764	Syntaxin-binding protein 1	3	0.470	0.247	0.092
O60343	TBC1 domain family member 4	1	1.594	NA	NA
Q63HR2	Tensin-like C1 domain-containing phosphatase	2	0.190	NA	NA
Q8IYW2	Tetratricopeptide repeat protein 40	1	1.008	NA	NA
Q16762	Thiosulfate sulfurtransferase	1	1.259	NA	NA
P07996	Thrombospondin-1	1	0.199	NA	NA
P62328	Thymosin beta-4	2	4.334	NA	NA
Q07157	Tight junction protein ZO-1	1	0.761	NA	NA
P62995	Transformer-2 protein homolog beta	1	1.448	NA	NA
P28289	Tropomodulin-1	1	0.351	NA	NA
P04350	Tubulin beta-4A chain	1	1.622	NA	NA
Q9GZM7	Tubulointerstitial nephritis antigen-like	1	0.532	NA	NA
Q99816	Tumor susceptibility gene 101 protein	1	0.793	NA	NA
P54577	Tyrosine--tRNA ligase, cytoplasmic	1	0.847	NA	NA
P78324	Tyrosine-protein phosphatase non-receptor type substrate 1	1	1.549	NA	NA
Q14139	Ubiquitin conjugation factor E4 A	1	1.403	NA	NA
Q96FW1	Ubiquitin thioesterase OTUB1	2	1.328	NA	NA
Q13459	Unconventional myosin-IXb	1	1.219	NA	NA
Q96A05	V-type proton ATPase subunit E 2	1	2.417	NA	NA
Q9UI12	V-type proton ATPase subunit H	1	1.973	NA	NA
Q86WA6	Valacyclovir hydrolase	1	1.997	NA	NA
Q8IWB7	WD repeat and FYVE domain-containing protein 1	2	1.384	NA	NA
P46937	Yorkie homolog	3	0.753	0.096	0.099

Proteins (n = 239) detected only in non-metastasized UM tumors among 1646 total proteins quantified with 2 or more peptides in metastatic samples UM 19, 21, 24, 28, 30 and/or non-metastatic samples UM 13, 20, 23, 25, 26. NA, not applicable, n<3 samples.
